# Supplementary material for: Poly(heptazine imide) ligand exchange enables remarkable low catalyst loadings in heterogeneous metallaphotocatalysis
Source: Nat Commun. 2023 Mar 17;14:1501. doi: 10.1038/s41467-023-37113-8 (PMC10023668; doi:10.1038/s41467-023-37113-8)
Supplement: Supplementary file 1 — Supplementary Information [file 41467_2023_37113_MOESM1_ESM.pdf]

Supplementary information

Poly(Heptazine Imide) Ligand Exchange Enables Remarkable Low Catalyst Loadings in Heterogeneous Metallaphotocatalysis

Liuzhuang Xing,† Qian Yang,† Chen Zhu,† Yilian Bai, Yurong Tang,* Magnus Rueping,* and Yunfei Cai*

Table of Contents

1 Supplementary Methods S2

1.1 General methods S3

1.2 Preparation of L_nNi-PHI catalysts S3

1.3 Structure characterization of L_nNi-PHI S8

1.4 Preliminary optimization on the structure of bpyNi-PHI by DFT S14

1.5 Preparation and characterization of Ni-PHI S15

1.6 Optimization of the reaction conditions and control experiments S17

1.7 General procedures (GPs) for bpyNi-PHI catalyzed cross-couplings S22

1.8 Catalyst recycling S24

1.9 Scale-up experiments S24

1.10 Structure characterization of the recovered bpyNi-PHI S26

1.11 Characterization date for the products S27

1.12 ^{1}HNMR spectra of $L_nNiCl_2(H_2O)_n$ S60

1.13 ^{1}H NMR spectra of L_nNi in L_nNi-PHI after acid treatment S63

1.14 NMR spectra of products S66

2 Supplementary References S198

1 Supplementary Methods

1.1 General methods

All reactions were carried out using oven-dried glassware and magnetic stirring under an inert atmosphere (N_2) unless otherwise stated. All chemical were obtained from commercial supplier and were used without further purification unless otherwise stated. All solvents were dried and distilled under N_2 prior to use. Solvents for chromatography were of technical grade and distilled prior to use. Analytical thin layer chromatography was carried out using silica gel GF254, visualized under UV light (at 254 nm). All NMR spectra were measured at room temperature using a Bruker 500 (500 MHz for ^{1}H, 126 MHz for ^{13}C) or a Bruker 400 (400 MHz for ^{1}H, 101 MHz for ^{13}C, 377 MHz for ^{19}F and 162 MHz for ^{31}P) NMR spectrometer in $CDCl_3$, DMSO-*d6*, and MeOH-*d4* solutions with internal solvent signals (for ^{1}H and ^{13}C) as reference (7.26, 77.2 for $CDCl_3$, 2.50 and 39.5 for DMSO-*d6*, and 3.31, 49.0 for MeOH-*d4*). All chemical shifts are reported in δ-scale as parts per million [ppm] (multiplicity, coupling constant *J*, number of protons) relative to the solvent residual peaks as the internal standard. The following abbreviations were used to express the multiplicities: s = singlet; d = doublet; t = triplet; q = quartet; m = multiplet; dd = doublet of doublets; dt = doublet of triplets; br = broad. Melting points were measured using a melting point apparatus in open glass capillaries.

High resolution mass spectra were acquired on a SolariX 15.0 T FT-MS (ESI). The light-promoted reactions were carried out by using standard blue LEDs with 28 blue LED beads (EPISTAR, 1 W LED beads and wavelength 460 ± 5 nm, in Supplementary Figure 19a and S20), the output power at 3 cm distance from the light source 19 mW/cm^2. Irradiance of the LED modules was measured using CEL-NP2000 Optical Power and Energy Meter equipped. Fourier Transform Infrared (FTIR) spectra from 4000 to 400 cm^{-1} were recorded in KBr discs on a Nicolet iS50 FTIR spectrometer. XRD patterns of the samples were recorded using X' Pert Pro X-ray diffractometer (Philips) with Cu Kα radiation (λ = 1.54 Å). Transmission electron microscopy (TEM) images and EDX elemental mappings were obtained on a Talos F200S transmission electron microscope. Aberration-corrected high-angle annular dark field scanning transmission electron microscopy (HAADF-STEM) images were conducted on a double-corrected Titan ETEM G2 80-300 transmission electron microscopy instrument at voltage of 300 kV. X-ray photoelectron spectroscopy (XPS) was performed with a ESCALAB250Xi electron spectrometer using monochromatic Al Ka radiation. Diffuse-reflectance UV-vis (DRUV-vis) absorption spectra of the samples in solid state has been acquired using Shimadzu UV-3600. Inductively coupled plasma optical emission spectrometry (ICP-OES) data was obtained from iCAP 6300 Duo. Photoluminescence spectras and lifetimes were recorded on an Edinburg Instruments FLS1000 spectrofluorometer at room temperature with a 450 W Xenon lamp and a 375 nm nanosecond Pulsed Diode laser (EPL), respectively. BET surface area was determined using N_2 adsorption-desorption isotherm. This was measured by BET surface area analyzer instrument (Quantachrome NovaWin) at 77 K. Degassing was performed under N_2 atmosphere. XAFS and EXAFS data were collected in fluorescence mode on the X-ray Absorption

Spectroscopy beamline at the Shanghai Synchrotron (200 mA, 3.0 GeV). The powder samples were homogeneously mixed with cellulose to obtain a metal concentration around 1000 ppm. A Si (111) single crystal was used to monochromatize the X-ray beam. XANES and EXAFS data reduction and analysis were processed by Athena software.

Photoelectrochemical measurements were performed with a Zahner Ennium electrochemical workstation in a conventional three electrode cell, using a platinum wire as the counter electrode and Ag/$AgNO_3$ (0.01 M $AgNO_3$, 0.1 M NBu_4PF_6, MeCN) as reference electrode (RE). The supporting electrolyte was 0.1 M Bu_4NPF_6/MeCN solution. The working electrode was prepared on indium-tin oxide (ITO) glass that was cleaned by sonication in deionized (DI) water for 30 min and dried at 353 K. 3 mg sample was dispersed in 1 mL DI water with sonication to get the slurry. The slurry and nafion (5 wt %, 30 μL) were spread onto a 1.5 cm × 1.5 cm pretreated ITO glass and air-drying.

Mott-Schottky plots were carried out in 0.2 M Na_2SO_4 aqueous solution as the electrolyte at frequencies of 1000, 1500, and 2000 Hz. The measurement processes were performed in 0.2 M Na_2SO_4 aqueous solution, Ag/AgCl electrode (saturated KCl solution) and platinum wire were used as reference electrode and counter electrode, respectively. The preparation of the working electrode was consistent with the aforementioned process.

1.2 Preparation of L_nNi-PHI Catalysts

Preparation of K-PHI as photocatalyst carrier: Melamine (1 g) was thoroughly grinded with NH_4Cl (3 g) and KCl (10 g). The mixture was heated to 550 °C at a rate of 2.3 °C min^{-1} and kept at this temperature for 4 h under nitrogen flow (90 mL/min). After cooling to room temperature, the solid mixture was washed with DI water (5 × 9 mL) then dried overnight at 60 °C to obtain final product K-PHI (0.5 g). According to the ICP-OES results, the content of K in K-PHI was determined to be 12 wt %. The structure of the prepared K-PHI has been confirmed by a series techniques and characterization results are in agreement with our reported earlier data.[1]

Preparation of bipyridyl-Ni(II) complexes[2]: To a round-bottom flask, $NiCl_2 \cdot 6(H_2O)$ (1.0 g, 4.2 mmol) was dissolved in 20 mL of ethanol and heated to reflux. 2,2'-bipyridine (bpy) solution (688 mg, 4.4 mmol) in 5 mL of ethanol was added dropwise to the refluxing solution. After 12 h, a pale green precipitate formed and was filtered off, washed with ethanol (3 × 5 mL) and tetrahydrofuran (3 × 5 mL), and dried in vacuo to afford **(bpy)$NiCl_2(H_2O)_n$** as a pale green solid (1 g, 83% yield determined by ^{1}H-NMR with trimethyl benzene-1,3,5-tricarboxylate as an internal standard). **^{1}H NMR** (400 MHz, DMSO-*d6*) δ 156.61 (br, 2H), 59.71 (s, 2H), 42.86 (s, 2H), 14.90 (s, 2H). The same synthetic procedure as **(bpy)$NiCl_2(H_2O)_n$** was performed for the preparation of other Ni complexes. The chemical structures of different ligands are shown below:

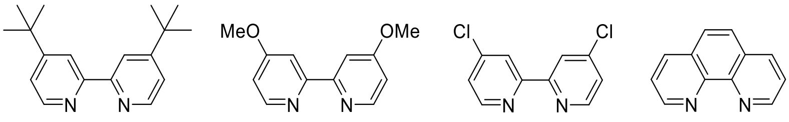

dtbpy dOMebpy dClbpy phen

(dtbpy)NiCl$_2$(H$_2$O)$_n$: pale green solid, 1.4 g, 85% yield. **^{1}H NMR** (400 MHz, DMSO-*d6*) δ 156.51 (br, 2H), 57.57 (s, 2H), 40.16 (s, 2H), 1.49 (s, 18H).

(dOMebpy)NiCl$_2$(H$_2$O)$_n$: pale green solid, 1.3 g, 90% yield. **^{1}H NMR** (400 MHz, DMSO-*d6*) δ 154.26 (br, 2H), 54.72 (s, 2H), 38.00 (s, 2H), 3.77 (s, 6H).

(dClbpy)NiCl$_2$(H$_2$O)$_n$: pale green solid, 1.3 g, 90% yield. **^{1}H NMR** (400 MHz, DMSO-*d6*) δ 149.38 (br, 2H), 54.42 (s, 2H), 36.99 (s, 2H).

(phen)NiCl$_2$(H$_2$O)$_n$: pale green solid, 1.2 g, 96% yield. **^{1}H NMR** (400 MHz, DMSO-*d6*) δ 157.64 (br, 2H), 45.56 (s, 2H), 23.77 (s, 2H), 17.56 (s, 2H).

Preparation of L$_n$Ni-PHI: To a 20 mL oven-dried sealed tube equipped with a magnetic stir bar was added (bpy)NiCl$_2$(H$_2$O)$_n$ (40 mg) and K-PHI (200 mg). It was capped with a rubber septum, evacuated and backfilled with nitrogen three times. Then, DMF (5 mL) was added via syringe. The mixture was stirred under nitrogen at room temperature for 72 h and centrifuged. The resulting solid was successively washed with DMF (2 × 5 mL) with sonication and separation by centrifugation, deionized water (2 × 5 mL) with sonication and separation by centrifugation, and MeCN (2 × 5 mL) with sonication and separation by centrifugation. The resulting powder was dried at 50 °C under vacuum for 12 h to yield bpyNi-PHI as dark yellow powder. (average yield per batch: ~202 mg). Other L$_n$Ni-PHI catalysts including dtbpyNi-PHI, dOMebpyNi-PHI, dClbpyNi-PHI and phenNi-PHI were prepared using the corresponding bipyridyl-Ni(II) complexes instead of (bpy)NiCl$_2$(H$_2$O)$_n$ via the same procedure as bpyNi-PHI. According to the ICP-OES results, the content of Ni in L$_n$Ni-PHI was determined to be 0.29 mmol/g (1.68 wt %) for bpyNi-PHI, 0.18 mmol/g (1.04 wt %) for dtbpyNi-PHI, 0.24 mmol/g (1.39 wt %) for dOMebpyNi-PHI, 0.21 mmol/g (1.22 wt %) for dClbpyNi-PHI, 0.25 mmol/g (1.45 wt %) for phenNi-PHI, respectively. The scale-up synthesis was also performed using 2.0 g of K-PHI and 0.4 g of (bpy)NiCl$_2$(H$_2$O)$_n$ in DMF (50 mL) to produce 2.1 g of bpyNi-PHI with 1.53 wt % Ni content.

Supplementary Table 1. Ni leaching in different solvent.

solvent	Ni in supernatant[a] (μg/mL)	Ni leaching (%)
DMF	2.70	3.21
MeCN	1.40	1.67
DMSO	3.01	3.56
MeOH	1.09	1.30
DCM	0.49	0.58
THF	0.60	0.71
H_2O	4.11	4.89

Procedure: The bpyNi-PHI (10 mg, 1.68 wt % Ni) was dispersed in the corresponding solvent (2 mL) and stired at room temperature for 24 h. Then the supernatant was collected by centrifugation. [a]Determined by ICP-OES.

Supplementary Table 2. The change of Ni content in L_nNi-PHI before and after acid treatment.

L_nNi-PHI	Ni in fresh catalyst (mmol)	Ni in solid after acid treatment[a] (mmol)	Ni in combined solution after acid treatment[a] (mmol)
bpyNi-PHI	0.029	0	0.029
dtbpyNi-PHI	0.018	0	0.017
dOMebpyNi-PHI	0.024	0	0.024
dClbpyni-PHI	0.021	0	0.022
phenNi-PHI	0.025	0	0.024

[a]The L_nNi-PHI (100 mg) was treated with 1.5 M HCl in a mixed H_2O:MeOH (1:1, v:v) solvent (4 mL) at room temperature for 20 min and then centrifuged. The resulted solid was washed with 1.5 M HCl in a mixed H_2O:MeOH (1:1, v:v) solvent (4 mL) and separated by centrifugation (2x). The combined solution was concentrated in vacuo and further determined by ICP-OES.

Supplementary Table 3. The content of Ni, K and L_nNi in L_nNi-PHI.

L_nNi-PHI[a]	Ni (mmol/g)[b]	K (mmol/g)[b]	L_nNi (mmol/g)[c]	L_nNi (wt %)[c]
bpyNi-PHI (batch 1)	0.29	2.4	0.28	6.0
bpyNi-PHI (batch 2)	0.28	2.3	0.27	5.8
bpyNi-PHI (gram-scale)[d]	0.26	2.5	0.25	5.4
dtbpyNi-PHI (batch 1)	0.18	2.8	0.18	5.9
dtbpyNi-PHI (batch 2)	0.20	2.7	0.19	6.2
dOMebpyNi-PHI (batch 1)	0.24	2.7	0.23	6.3
dOMebpyNi-PHI (batch 2)	0.20	2.6	0.22	6.0
dClbpyni-PHI (batch 1)	0.21	2.6	0.20	5.6
dClbpyni-PHI (batch 2)	0.20	2.7	0.21	5.9
phenNi-PHI (batch 1)	0.25	2.6	0.26	6.2
phenNi-PHI (batch 2)	0.25	2.5	0.25	6.0

[a]The catalyst preparation was performed on 200 mg scale. [b]Determined by ICP-OES. [c]Determined by ^{1}H NMR analysis (trimethyl benzene-1,3,5-tricarboxylate as an internal standard) of Ni complex in the combined solution of L_nNi-PHI after treatment with 1.5 M HCl in a mixed H_2O:MeOH (1:1, v:v) solvent. [d]The catalyst prepartion was performed on 2 g scale.

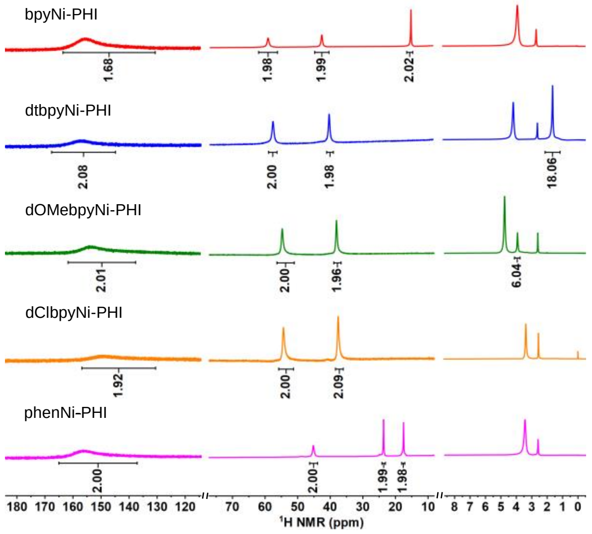

Supplementary Figure 1. ^{1}H NMR spectra (DMSO-*d*6, 400 MHz) of nickel complexes in L_nNi-PHI after treatment with 1.5 M HCl in a mixed H_2O:MeOH (1:1, v:v) solvent.

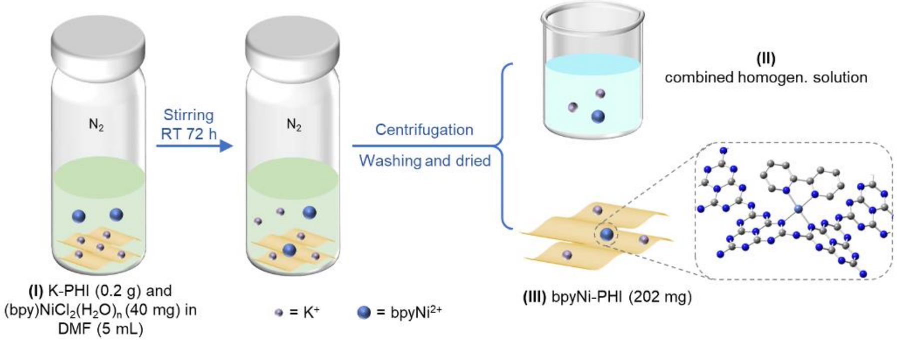

Supplementary Figure 2. Schematic illustration for the synthesis of bpyNi-PHI (6.0 wt % Nibpy).

Supplementary Table 4. The changes of K, Ni and Nibpy contents during the preparation of bpyNi-PHI (6.0 wt % Nibpy).

sample[a]	K (mmol)[b]	Ni (mmol)[b]	Nibpy (mmol)[c]
K-PHI + $(bpy)NiCl_2(H_2O)_n$ (**I**)	0.62	0.13	0.13
Combined homogen. solution (**II**)	0.12	0.072	0.075
bpyNi-PHI (**III**)	0.49	0.058	0.056[d]

[a]Corresponding to the sample shown in Supplementary Figure 2. [b]Determined by ICP-OES. [c]Determined by ^{1}H NMR analysis with trimethyl benzene-1,3,5-tricarboxylate as an internal standard. [d]Determined by ^{1}H NMR analysis (trimethyl benzene-1,3,5-tricarboxylate as an internal standard) of Ni complex in the combined solution of bpyNi-PHI after treatment with 1.5 M HCl in a mixed H_2O:MeOH (1:1, v:v) solvent.

Preparation of bpyNi-PHI bearing different Nibpy loadings: To a 20 mL oven-dried flask equipped with a magnetic stir bar was added $(bpy)NiCl_2(H_2O)_n$ (100 mg or 20 mg) and K-PHI (200 mg). It was capped with a rubber septum, evacuated and backfilled with nitrogen three times. Then, DMF (40 mL) was added via syringe. The mixture was stirred under nitrogen at room temperature for 72 h and centrifuged. The resulting solid was successively washed with DMF (2 × 5 mL) with sonication and separation by centrifugation, deionized water (2 × 5 mL) with sonication and separation by centrifugation, and MeCN (2 × 5 mL) with sonication and separation by centrifugation. The resulting powder was dried at 50 °C under vacuum for 12 h to yield bpyNi-PHI (2.5 wt % Nibpy) or bpyNi-PHI (1.5 wt % Nibpy).

Preparation of bpyNi-CN: For comparison, bpyNi-CN catalysts including bpyNi-g-C_3N_4, bpyNi-mpg-CN and bpyNi-CCN were prepared via the same procedure as bpyNi-PHI with graphite carbon nitride (g-C_3N_4),[3] mesoporous graphitic carbon nitride (mpg-CN),[4] and crystalline carbon nitride (CCN)[5] as carbon nitride precursors. According to the ICP-OES results, the content of Ni in bpyNi-CN was determined to be 0.05 wt % for bpyNi-g-C_3N_4, 0.23 wt % for bpyNi-mpg-CN and 0.21 wt % for bpyNi-CCN, respectively.

1.3 Structure characterization of L_nNi-PHI

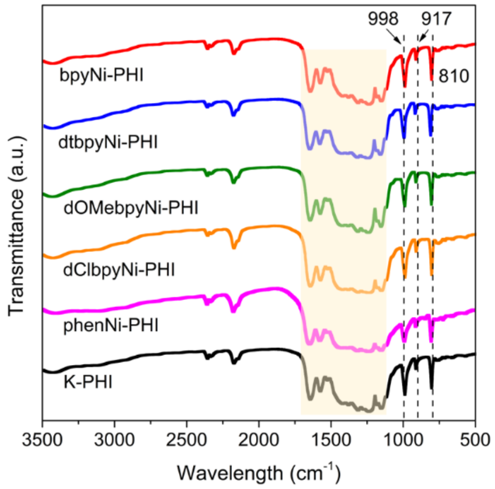

Supplementary Figure 3. FTIR spectra of L_nNi-PHI and K-PHI.

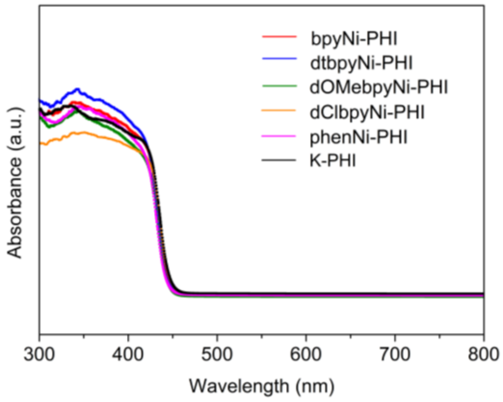

Supplementary Figure 4. UV-vis spectra of L_nNi-PHI and K-PHI.

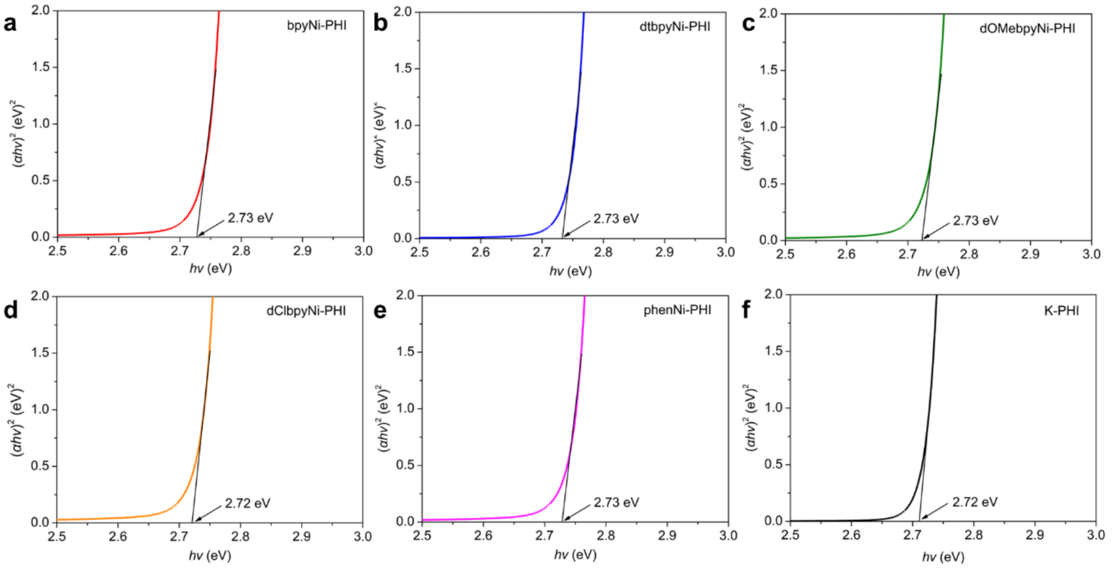

Supplementary Figure 5. Tauc plots of bpyNi-PHI (a), dtbpyNi-PHI (b), dOMebpyNi-PHI (c), dClbpyNi-PHI (d) phenNi-PHI (e) and K-PHI (f).

The band gaps of L_nNi-PHI were calculated from the curve of photon energy by $(ahv)^2$. As can be seen from Supplementary Figure 5, the values of bpyNi-PHI, dtbpyNi-PHI, dOMebpyNi-PHI, dClbpyNi-PHI, phenNi-PHI and K-PHI are 2.73 eV, 2.73 eV, 2.73 eV, 2.72 eV, 2.73 eV and 2.72 eV, respectively.

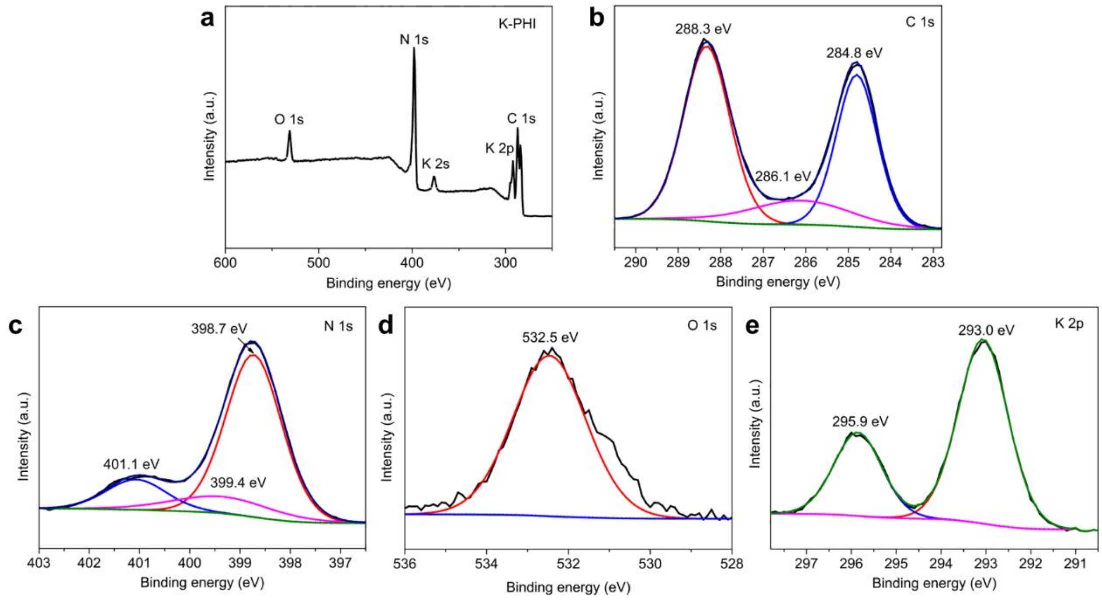

Supplementary Figure 6. XPS spectra of K-PHI: (a) survey spectrum, (b) C 1s, (c) N 1s, (d) O 1s and (e) K 2p high-resolution spectra.

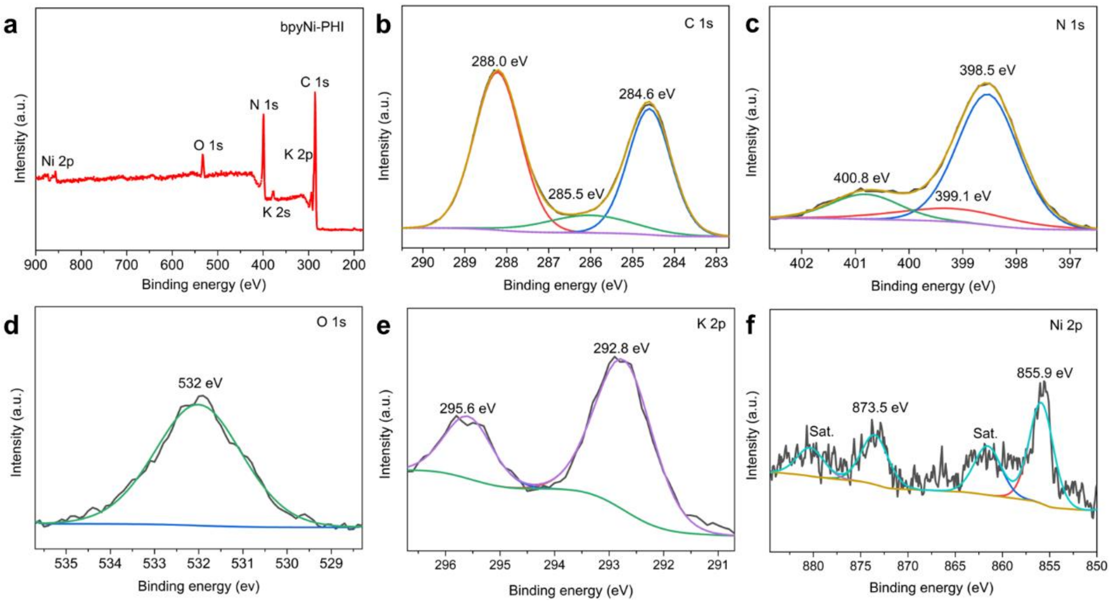

Supplementary Figure 7. XPS spectra of bpyNi-PHI: (a) survey spectrum, (b) C 1s, (c) N 1s, (d) O 1s, (e) K 2p and (f) Ni 2p high-resolution spectra.

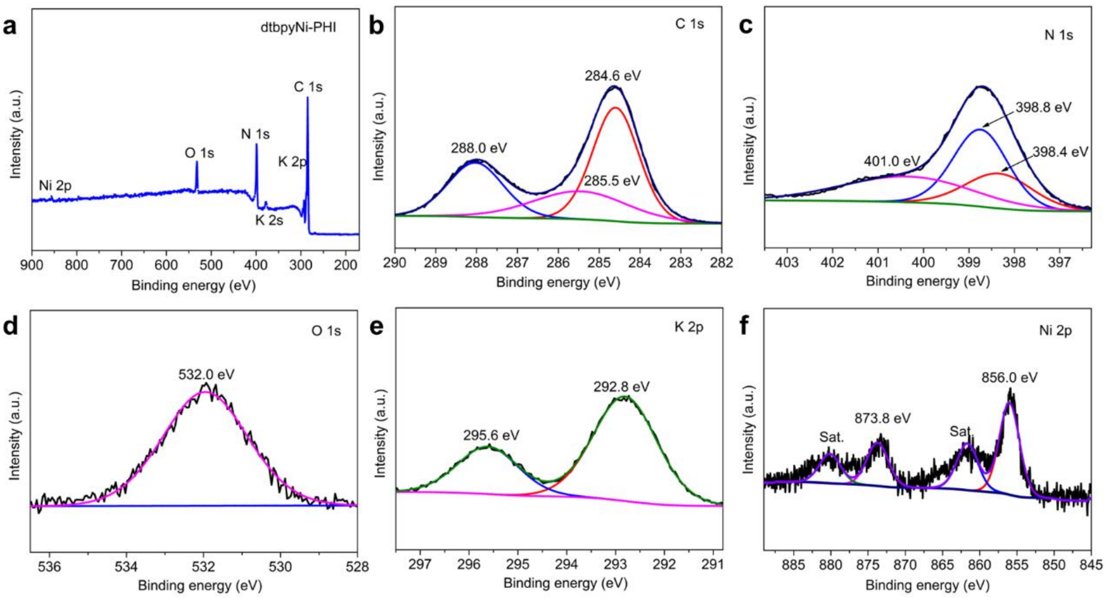

Supplementary Figure 8. XPS spectra of dtbpyNi-PHI: (a) survey spectrum, (b) C 1s, (c) N 1s, (d) O 1s, (e) K 2p and (f) Ni 2p high-resolution spectra.

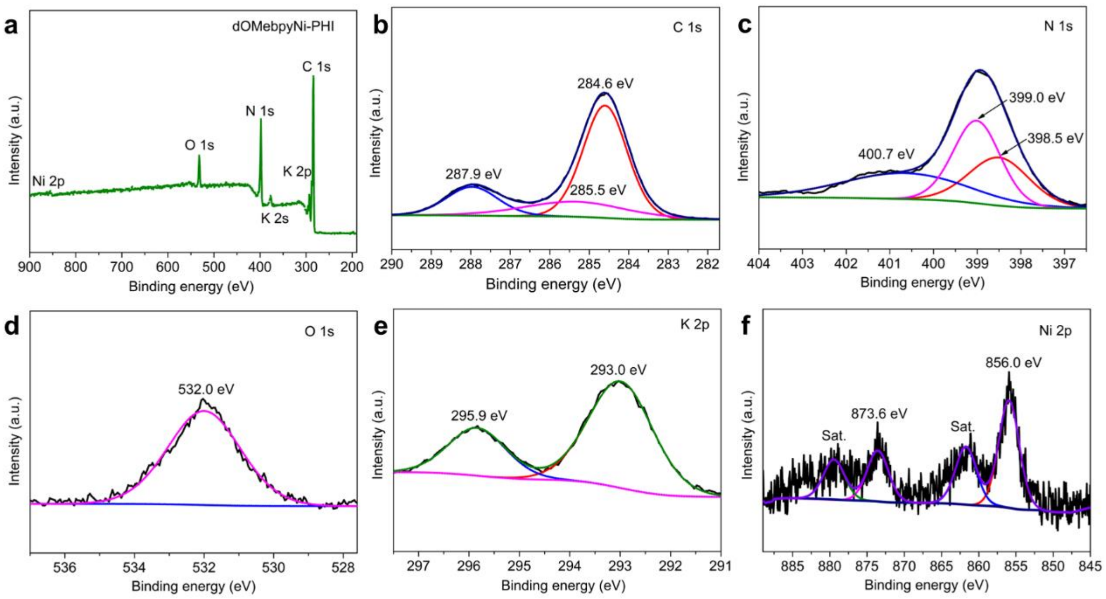

Supplementary Figure 9. XPS spectra of dOMebpy-PHI: (a) survey spectrum, (b) C 1s, (c) N 1s, (d) O 1s, (e) K 2p and (f) Ni 2p high-resolution spectra.

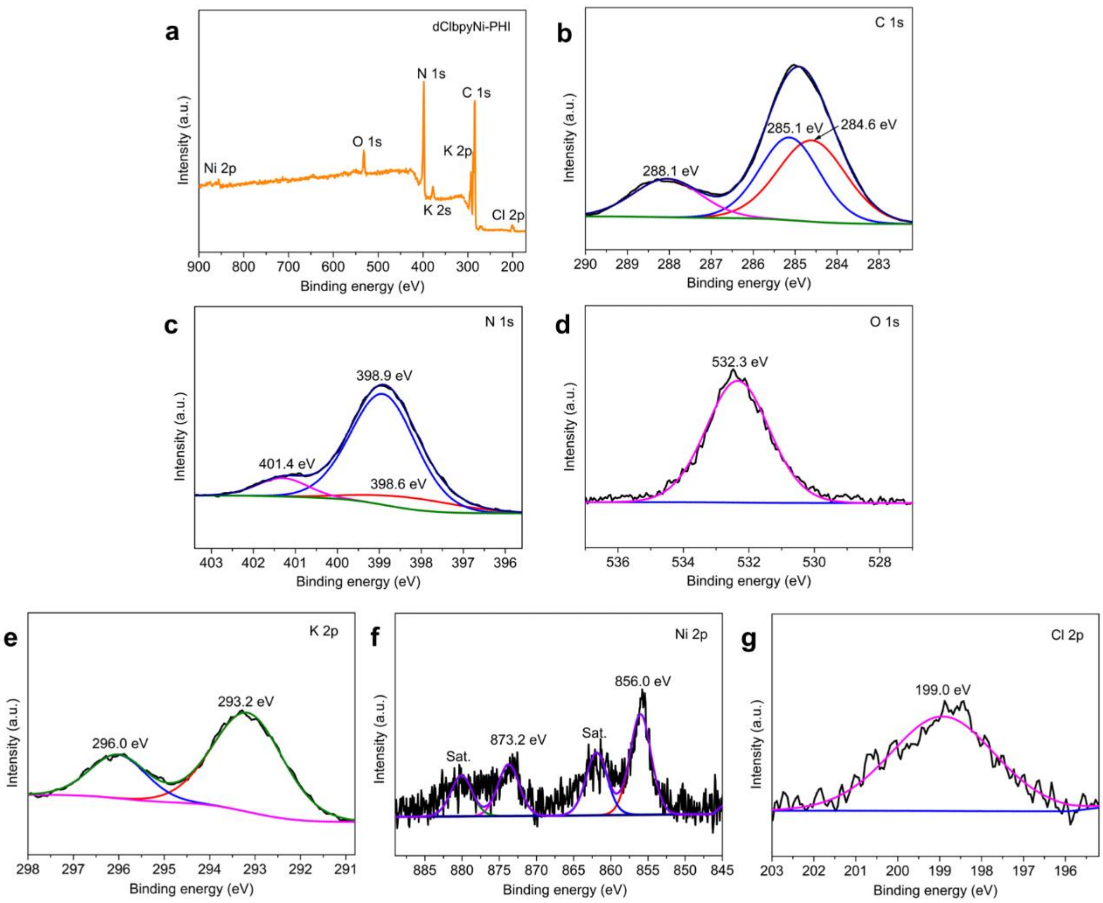

Supplementary Figure 10. XPS spectra of dClbpyNi-PHI: (a) survey spectrum, (b) C 1s, (c) N 1s, (d) O 1s, (e) K, (f) Ni 2p and (g) Cl 2p high-resolution spectra.

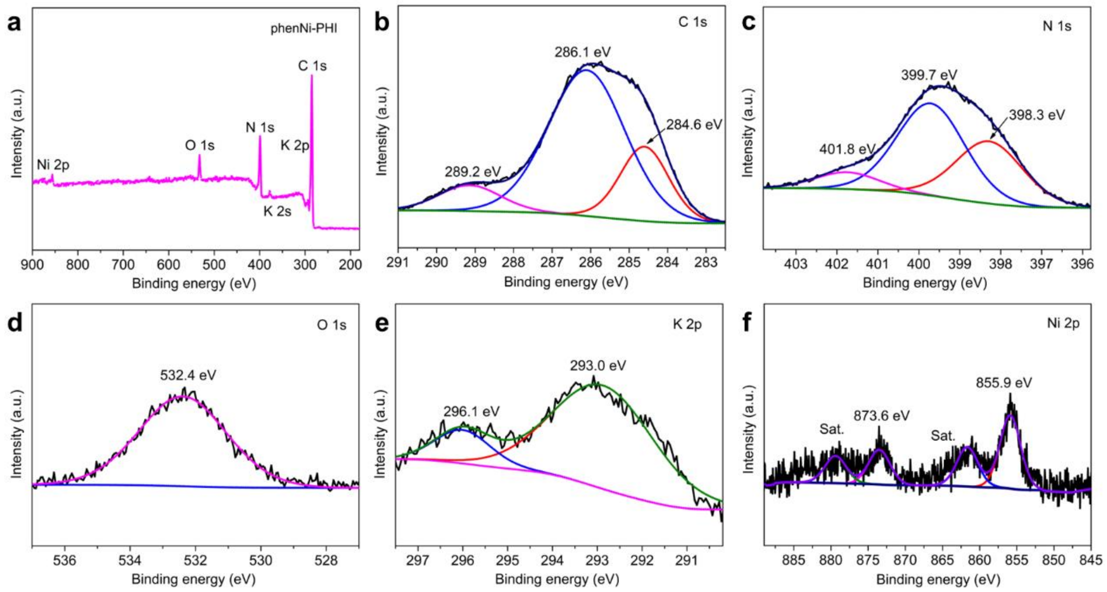

Supplementary Figure 11. XPS spectra of phenNi-PHI: (a) survey spectrum, (b) C 1s, (c) N 1s, (d) O 1s, (e) K 2p and (f) Ni 2p high-resolution spectra.

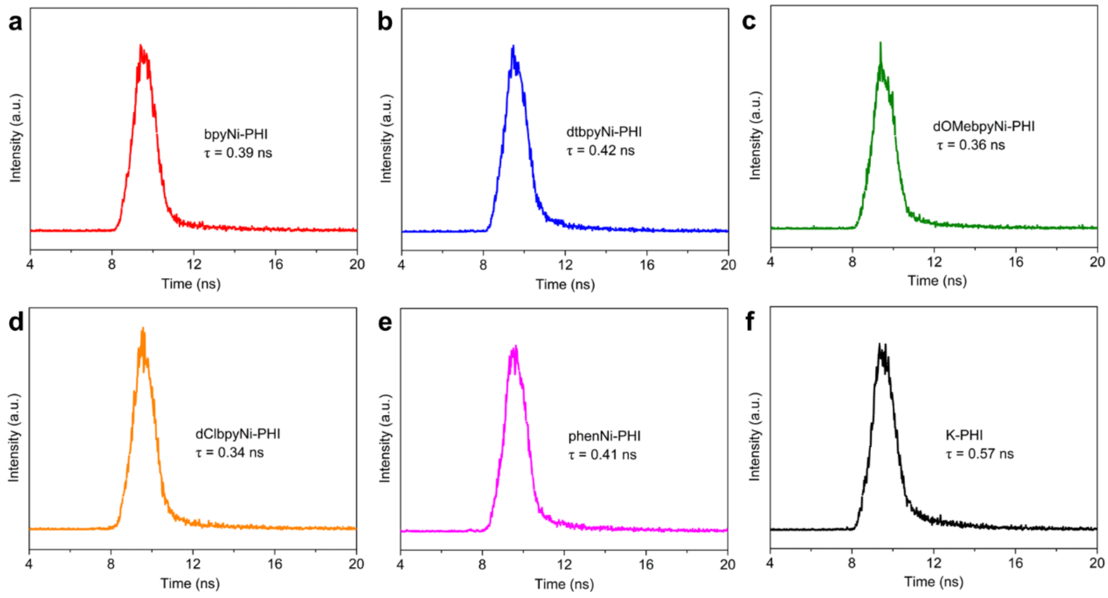

Supplementary Figure 12. Time-resolved fluorescence decay spectra of bpyNi-PHI (a), dtbpyNi-PHI (b), dOMebpyNi-PHI (c), dClbpyNi-PHI (d), phenNi-PHI (e) and K-PHI (f).

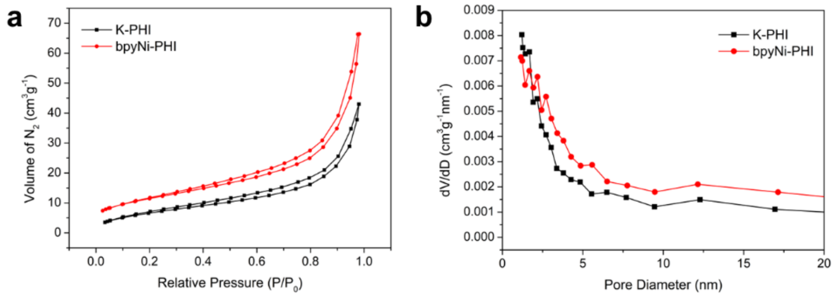

Supplementary Figure 13. N_2 adsorption/desorption isotherm(a) and BJH pore size distribution from the N_2 adsorption branch (b) of K-PHI and bpyNi-PHI at 77 K.

Supplementary Table 5. BET specific surface area, pore diameter and total pore volume.

Sample	Specific surface area (m^2g^{-1})	Pore diameter (nm)	Total pore volume (ccg^{-1})
K-PHI	25.8	10.3	0.066
bpyNi-PHI	41.3	9.94	0.103

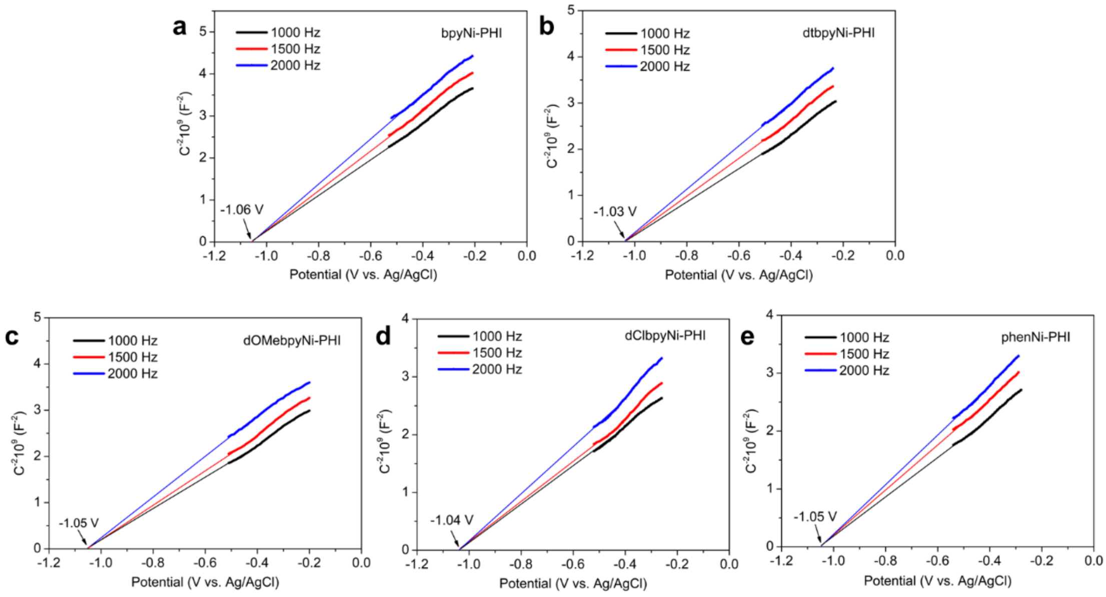

Supplementary Figure 14. Mott-Schottky plots of bpyNi-PHI (a), dtbpyNi-PHI (b), dOMebpyNi-PHI (c), dClbpyNi-PHI (d) and phenNi-PHI (e). Experimental conditions: 0.2 M Na_2SO_4, pH 6.6.

The flat band potentials of the L_nNi-PHI were measured by Mott-Schottky plots at frequencies of 1000, 1500, and 2000 Hz (Supplementary Figure 14). The intercept of the curve on the abscissa is the flat band potential of the sample and its values (vs. Ag/AgCl) are -1.06 V, -1.03 V, -1.05 V, -1.04 V and -1.05 V of bpyNi-PHI, dtbpyNi-PHI, dOMebpyNi-PHI, dClbpyNi-PHI and phenNi-PHI. The slope of the curve is positive, which reveal the sample is n-type semiconductors. For n-type semiconductor, the flat band potential is about 0.3 V below the conduction band (CB) potential.[6-8] According to the equation of:

$$E_{vs\ SCE} = E_{vs\ Ag/AgCl} - 0.042\ V$$

All potentials were converted to the standard calomel electrode (SCE). Therefore, the conduction band (CB) potentials of bpyNi-PHI, dtbpyNi-PHI, dOMebpyNi-PHI, dClbpyNi-PHI and phenNi-PHI are -1.40 V, -1.37 V, -1.39 V, -1.38 V and -1.39 V (vs. SCE), respectively.

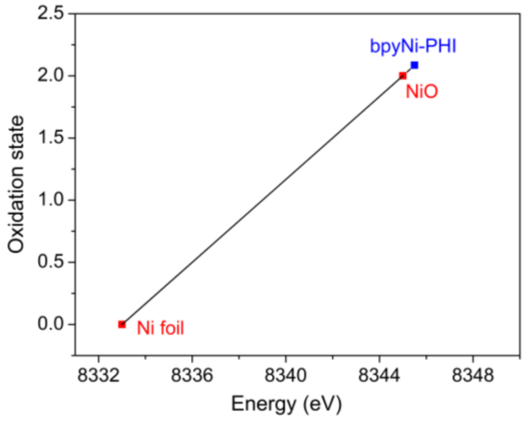

Supplementary Figure 15. The fitted average oxidation states of Ni from XANES spectra.

Supplementary Table 6. Fitting results of Ni K-edge FT-EXAFS curves

Sample	Valence	Path	C.N.	R (Å)	σ^2 ($\times 10^3$ Å^2)	ΔE (eV)	R factor
Ni foil	0	Ni-Ni	12*	2.48±0.01	6.0±0.2	6.8±0.3	0.001
bpyNi-PHI	2.08	Ni-N	4*	2.03±0.02	3.4±5.1	-2.9±10.1	0.018
		Ni-O	2*	2.12±0.03	1.8±5.4	0.4±9.8	

C.N: coordination numbers; R: bond distance; σ^2: Debye-Waller factors; ΔE: the inner potential correction. R factor: goodness of fit. *fitting with fixed parameter.

The obtained XAFS data was processed in Athena (version 0.9.26) for background, pre-edge line and post-edge line calibrations. Then Fourier transformed fitting was carried out in Artemis (version 0.9.26). The k^3 weighting, k-range of 3 - 12 Å^{-1} and R range of 1 - 3 Å were used for the fitting. The four parameters, coordination number, bond length, Debye-Waller factor and E_0 shift (CN, R, σ^2, ΔE_0) were fitted without anyone was fixed, constrained, or correlated.

1.4 Preliminary optimization on the structure of bpyNi-PHI by DFT

DFT calculations have been performed with the Gaussian 16, Revision B.01.[9] Geometry optimizations and frequency calculations of the ground state structure of a simplified model for the bpy-$Ni^{2+}(H_2O)_2PHI^{2-}$ system have been performed at the PBE-D3/(def2-TZVP for Ni, def2-SVP for other atoms) level of theory.[10, 11]

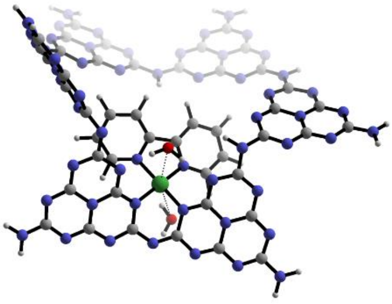

Supplementary Figure 16. bpy-$Ni^{2+}(H_2O)_2$-PHI^{2-} after DFT optimization.

1.5 Preparation and characterization of Ni-PHI

Preparation of Ni-PHI: The cation exchange method was also applied to prepare Ni-PHI by mixing $NiCl_2·6H_2O$ (40 mg) and K-PHI (200 mg) in DMF (5 mL) and stirring for 72 h. The average yield per batch: ~203 mg. According to the ICP-OES results, the content of Ni in Ni-PHI was determined to be 3.2 wt %.

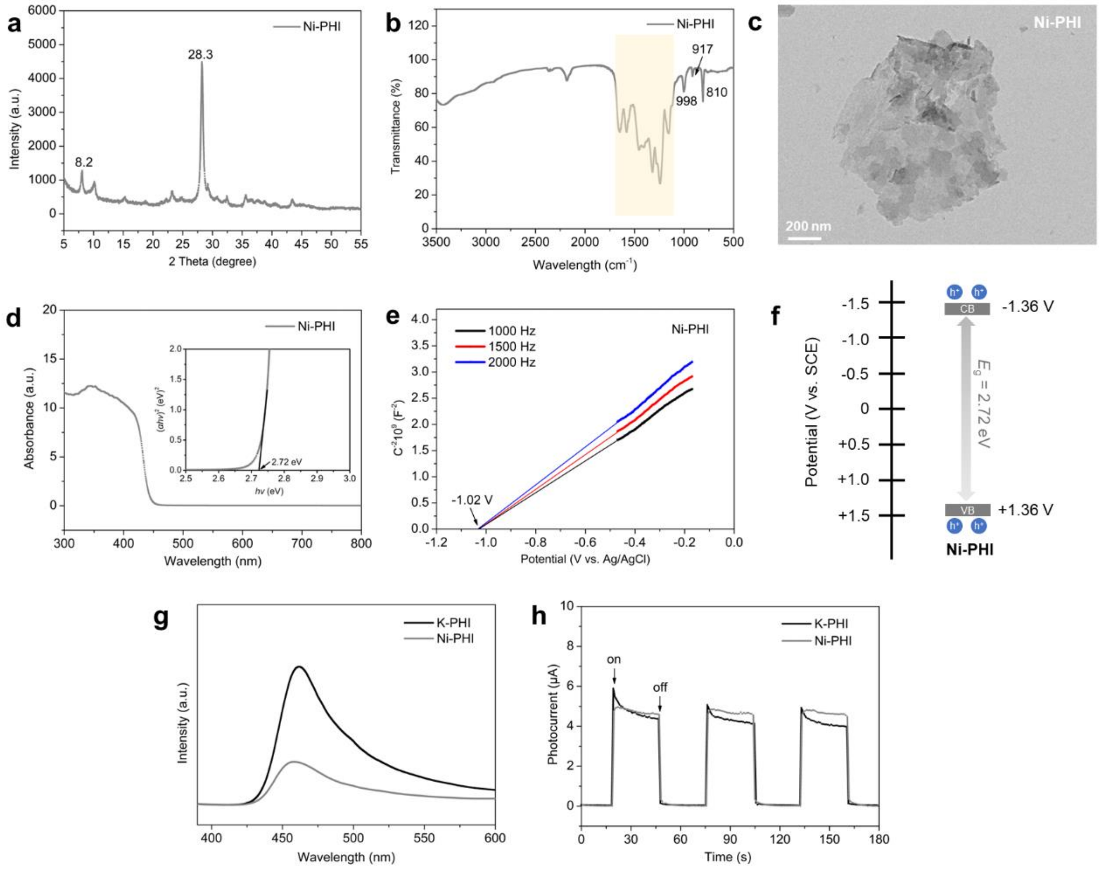

Supplementary Figure 17. Structural characterization of Ni-PHI: (a) XRD pattern, (b) FTIR spectra, (c) TEM image, (d) UV-vis spectra (inset: Tauc plots), (e) Mott-Schottky plot, (f) band structures, (g) steady-state PL emission spectra, (h) transient photocurrent responses (no bias potential vs Ag/AgCl).

XRD pattern (Supplementary Figure 17a), FTIR spectra (Supplementary Figure 17b) and UV-vis spectra (Supplementary Figure 17d) of Ni-PHI are similar to K-PHI, suggesting that Ni deposition did not affect the optical properties and composition of the PHI-based material. TEM images (Supplementary Figure 17c) suggest that Ni-PHI is a layered structure with nanometer-sized domains. Combining optical band gaps and Mott−Schottky results (Supplementary Figure 17e), the conduction band (CB) and valence band (VB) potentials of Ni-PHI were determined to be –1.36 V and +1.36 (vs SCE), respectively (Supplementary Figure 17f). The photoluminescence (PL) spectra of Ni-PHI display reduced emission (Supplementary Figure 17g), indicative of efficient electron transfer or energy transfer from the emissive state to Ni(II), producing the Ni(I) or excited Ni(II) species. The Ni-PHI exhibit prompt and reproducible photocurrent response under visible light illumination, further

demonstrating good charge separation and migration in Ni-PHI (Supplementary Figure 17h).

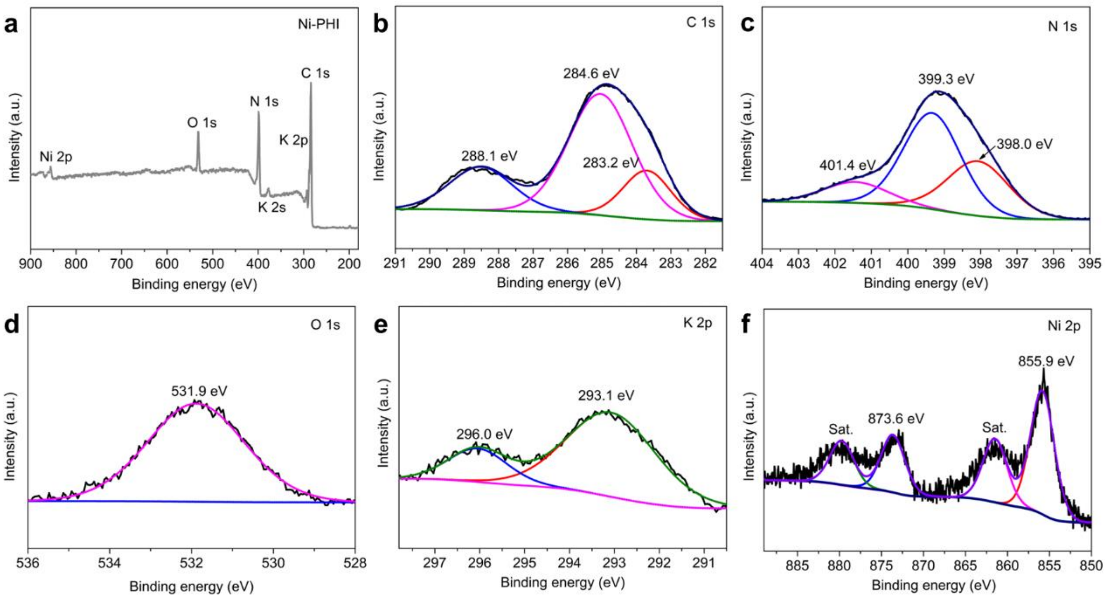

Supplementary Figure 18. XPS spectra of Ni-PHI: (a) survey spectrum, (b) C 1s, (c) N 1s, (d) O 1s, (e) K 2p and (f) Ni 2p high-resolution spectra.

The X-ray photoelectron spectra (XPS) indicate that Ni-PHI comprise elements of C, N, O, K and Ni. The binding energy located at 855.9 (Ni 2p3/2) and 873.6 eV (Ni 2p1/2) are assigned to Ni^{2+}.[12, 13]

1.6 Optimization of the reaction conditions and control experiments

Supplementary Table 7. Screening of catalyst and control experiments for C−P coupling

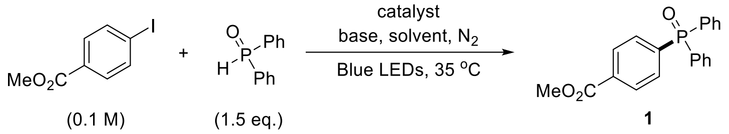

entry[a]	catalyst	base	solvent	time (h)	yield (%)[b]
1	bpyNi-PHI (10 mg, 6.0 wt % Nibpy)	$^{i}Pr_2NH$	DMF	42	97 (96)[c]
2	bpyNi-PHI (10 mg, 6.0 wt % Nibpy)	TMG	DMF	42	58
3	bpyNi-PHI (10 mg, 6.0 wt % Nibpy)	Et_3N	DMF	42	65
4	bpyNi-PHI (10 mg, 6.0 wt % Nibpy)	K_3PO_4	DMF	42	15
5	bpyNi-PHI (10 mg, 6.0 wt % Nibpy)	$^{i}Pr_2NH$	MeCN	42	76
6	bpyNi-PHI (10 mg, 6.0 wt % Nibpy)	$^{i}Pr_2NH$	DMSO	42	85
7	dtbpyNi-PHI (10 mg, 5.9 wt % Nidtbpy)	$^{i}Pr_2NH$	DMF	42	96 (94)[c]
8	dOMebpyNi-PHI (10 mg, 6.3 wt % NidOMebpy)	$^{i}Pr_2NH$	DMF	42	97 (96)[c]
9	phenNi-PHI (10 mg, 6.2 wt % Niphen)	$^{i}Pr_2NH$	DMF	42	96 (95)[c]
10	dClbpyNi-PHI (10 mg, 5.6 wt % NidClbpy)	$^{i}Pr_2NH$	DMF	48	93 (92)[c]
11		$^{i}Pr_2NH$	DMF	42	0
12	bpyNi-PHI (10 mg, 6.0 wt % Nibpy)		DMF	42	4
13[d]	bpyNi-PHI (10 mg, 6.0 wt % Nibpy)	$^{i}Pr_2NH$	DMF	42	trace
14	Ni-PHI (10 mg, 3.2 wt % Ni)	$^{i}Pr_2NH$	DMF	48	7
15	K-PHI (10 mg) + $NiCl_2$ (1.4 mol%)	$^{i}Pr_2NH$	DMF	48	6
16	K-PHI (10 mg) + (bpy)$NiCl_2(H_2O)_n$ (1.4 mol%)	$^{i}Pr_2NH$	DMF	42	31
17	bpyNi-PHI (10 mg, 2.5 wt % Nibpy)	$^{i}Pr_2NH$	DMF	42	82
18	bpyNi-PHI (10 mg, 1.5 wt % Nibpy)	$^{i}Pr_2NH$	DMF	42	66
19	bpyNi-PHI (2 mg, 6.0 wt % Nibpy)	$^{i}Pr_2NH$	DMF	72	92
20	K-PHI (2 mg) + (bpy)$NiCl_2(H_2O)_n$ (0.28 mol%)	$^{i}Pr_2NH$	DMF	72	15

[a]Reaction conditions: methyl 4-iodobenzoate (0.2 mmol, 1.0 eq.), diphenylphosphine oxide (0.3 mmol, 1.5 eq.), the corresponding catalyst, base (0.6 mmol, 3.0 eq.) in solvent (2 mL) under N_2 atmosphere and blue LEDs irradiation (24 W, 460 ± 5 nm) without extra heating (at 35 ± 5 °C) for the indicated time. [b]Determined by ^{1}H NMR analysis using 1,3,5-trimethoxybenzene as an internal standard. [c]Isolated yield in parenthesis. [d]In dark.

Note: Different batches of L_nNi-PHI catalysts (Supplementary Table 3) shows similar catalytic activity for the reaction (+/-5% based on ^{1}H-NMR with internal standard).

Supplementary Table 8. Screening of catalyst and control experiments for C−N coupling

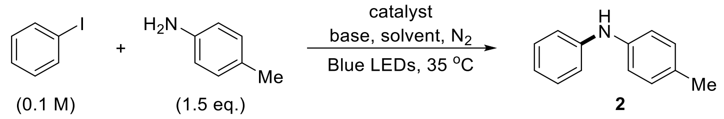

entry[a]	catalyst	base	solvent	time (h)	yield (%)[b]
1	bpyNi-PHI (10 mg, 6.0 wt % Nibpy)	TMG	MeCN	12	96 (95)[c]
2	bpyNi-PHI (10 mg, 6.0 wt % Nibpy)	DBU	MeCN	12	6
3	bpyNi-PHI (10 mg, 6.0 wt % Nibpy)	DIPEA	MeCN	12	9
4	bpyNi-PHI (10 mg, 6.0 wt % Nibpy)	$^{i}Pr_2NH$	MeCN	12	38
5	bpyNi-PHI (10 mg, 6.0 wt % Nibpy)	TMG	DMF	12	52
6	bpyNi-PHI (10 mg, 6.0 wt % Nibpy)	TMG	DMSO	12	32
7	dtbpyNi-PHI (10 mg, 5.9 wt % Nidtbpy)	TMG	MeCN	12	94 (92)[c]
8	dOMebpyNi-PHI (10 mg, 6.3 wt % NidOMebpy)	TMG	MeCN	12	93 (91)[c]
9	phenNi-PHI (10 mg, 6.2 wt % Niphen)	TMG	MeCN	12	96 (94)[c]
10	dClbpyNi-PHI (10 mg, 5.6 wt % NidClbpy)	TMG	MeCN	18	95 (92)[c]
11		TMG	MeCN	12	0
12	bpyNi-PHI (10 mg, 6.0 wt % Nibpy)		MeCN	12	trace
13[d]	bpyNi-PHI (10 mg, 6.0 wt % Nibpy)	TMG	MeCN	12	0
14	Ni-PHI (10 mg, 3.2 wt % Ni)	TMG	MeCN	18	8
15	K-PHI (10 mg) + $NiCl_2$ (1.4 mol%)	TMG	MeCN	18	9
16	K-PHI (10 mg) + $(bpy)NiCl_2(H_2O)_n$ (1.4 mol%)	TMG	MeCN	12	45
17	bpyNi-PHI (2 mg, 6.0 wt % Nibpy)	TMG	MeCN	36	90
18	K-PHI (2 mg) + $(bpy)NiCl_2(H_2O)_n$ (0.28 mol%)	TMG	MeCN	36	10

[a]Reaction conditions: iodobenzene (0.2 mmol, 1.0 eq.), *p*-toluidine (0.3 mmol, 1.5 eq.), the corresponding catalyst, base (0.4 mmol, 2.0 eq.) in solvent (2 mL) under N_2 atmosphere and blue LEDs irradiation (24 W, 460 ± 5 nm) without extra heating (at 35 ± 5 °C) for the indicated time. [b]Determined by ^1H NMR analysis using 1,3,5-trimethoxybenzene as an internal standard. [c]Isolated yield in parenthesis. [d]In dark.

Supplementary Table 9. Screening of catalyst and control experiments for C−O coupling of aryl iodide with alcohol

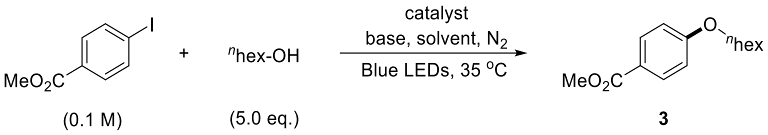

entry[a]	catalyst	base	solvent	time (h)	yield (%)[b]
1	bpyNi-PHI (10 mg, 6.0 wt % Nibpy)	TMG	MeCN	12	86 (85)[c]
2	bpyNi-PHI (10 mg, 6.0 wt % Nibpy)	Et_3N	MeCN	12	30
3	bpyNi-PHI (10 mg, 6.0 wt % Nibpy)	$^{i}Pr_2NH$	MeCN	12	48
4	bpyNi-PHI (10 mg, 6.0 wt % Nibpy)	TMG	DMF	12	46
5	bpyNi-PHI (10 mg, 6.0 wt % Nibpy)	TMG	DMSO	12	35
6	dtbpyNi-PHI (10 mg, 5.9 wt % Nidtbpy)	TMG	MeCN	12	86 (85)[c]
7	dOMebpyNi-PHI (10 mg, 6.3 wt % NidOMebpy)	TMG	MeCN	12	84 (82)[c]
8	phenNi-PHI (10 mg, 6.2 wt % Niphen)	TMG	MeCN	12	82 (80)[c]
9	dClbpyNi-PHI (10 mg, 5.6 wt % NidClbpy)	TMG	MeCN	16	80 (78)[c]
10		TMG	MeCN	12	0
11	bpyNi-PHI (10 mg, 6.0 wt % Nibpy)		MeCN	12	trace
12[d]	bpyNi-PHI (10 mg, 6.0 wt % Nibpy)	TMG	MeCN	12	0
13	Ni-PHI (10 mg, 3.2 wt % Ni)	TMG	MeCN	16	10
14	K-PHI (10 mg) + $NiCl_2$ (1.4 mol%)	TMG	MeCN	16	9
15	K-PHI (10 mg) + (bpy)$NiCl_2(H_2O)_n$ (1.4 mol%)	TMG	MeCN	12	48
16	bpyNi-PHI (0.5 mg, 6.0 wt % Nibpy)	TMG	MeCN	40	85
17	K-PHI (0.5 mg) + (bpy)$NiCl_2(H_2O)_n$ (0.07 mol%)	TMG	MeCN	40	5

[a]Reaction conditions: methyl 4-iodobenzoate (0.2 mmol, 1.0 eq.), 1-hexanol (1.0 mmol, 5.0 eq.), the corresponding catalyst, TMG (0.4 mmol, 2.0 eq.) in solvent (2 mL) under N_2 atmosphere and blue LEDs irradiation (24 W, 460 ± 5 nm) without extra heating (at 35 ± 5 °C) for the indicated time. [b]Determined by ^{1}H NMR analysis using 1,3,5-trimethoxybenzene as an internal standard. [c]Isolated yield in parenthesis. [d]In dark.

Supplementary Table 10. Screening of catalyst and control experiments for C−O coupling of aryl iodide with carboxylic acid

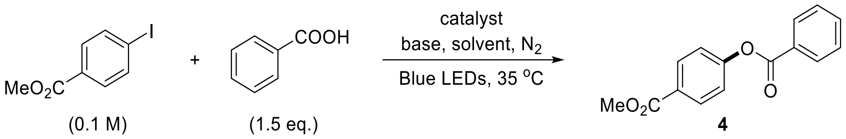

entry[a]	catalyst	base	solvent	time (h)	yield (%)[b]
1	bpyNi-PHI (10 mg, 6.0 wt % Nibpy)	BIPA	DMSO	10	87 (86)[c]
2	bpyNi-PHI (10 mg, 6.0 wt % Nibpy)	TMG	DMSO	10	25
3	bpyNi-PHI (10 mg, 6.0 wt % Nibpy)	$^{i}Pr_2NH$	DMSO	10	45
4	bpyNi-PHI (10 mg, 6.0 wt % Nibpy)	BIPA	DMF	10	65
5	bpyNi-PHI (10 mg, 6.0 wt % Nibpy)	BIPA	MeCN	10	40
6	dtbpyNi-PHI (10 mg, 5.9 wt % Nidtbpy)	BIPA	DMSO	10	87 (85)[c]
7	dOMebpyNi-PHI (10 mg, 6.3 wt % NidOMebpy)	BIPA	DMSO	10	82 (80)[c]
8	phenNi-PHI (10 mg, 6.2 wt % Niphen)	BIPA	DMSO	10	85 (84)[c]
9	dClbpyNi-PHI (10 mg, 5.6 wt % NidClbpy)	BIPA	DMSO	16	80 (77)[c]
10		BIPA	DMSO	10	0
11	bpyNi-PHI (10 mg, 6.0 wt % Nibpy)		DMSO	10	0
12[d]	bpyNi-PHI (10 mg, 6.0 wt % Nibpy)	BIPA	DMSO	10	trace
13	Ni-PHI (10 mg, 3.2 wt % Ni)	BIPA	DMSO	16	0
14	K-PHI (10 mg) + $NiCl_2$ (1.4 mol%)	BIPA	DMSO	16	0
15	K-PHI (10 mg) + (bpy)$NiCl_2(H_2O)_n$ (1.4 mol%)	BIPA	DMSO	10	23
16	bpyNi-PHI (0.5 mg, 6.0 wt % Nibpy)	BIPA	DMSO	40	80
17	K-PHI (0.5 mg) + (bpy)$NiCl_2(H_2O)_n$ (0.07 mol%)	BIPA	DMSO	40	5

[a]Reaction conditions: methyl 4-iodobenzoate (0.2 mmol, 1.0 eq.), benzoic acid (0.3 mmol, 1.5 eq), the corresponding catalyst, base (0.6 mmol, 3.0 eq) in solvent (2 mL) under N_2 atmosphere and blue LEDs irradiation (24 W, 460 ± 5 nm) without extra heating (at 35 ± 5 °C) for the indicated time. [b]Determined by ^{1}H NMR analysis using 1,3,5-trimethoxybenzene as an internal standard. [c]Isolated yield in parenthesis. [d]In dark.

Supplementary Table 11. Screening of catalyst and control experiments for C−S coupling

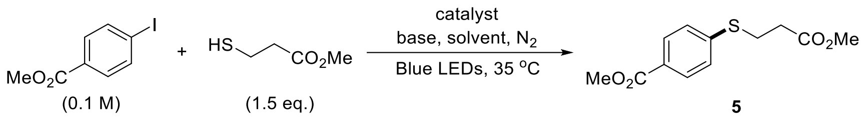

entry[a]	catalyst system	base	solvent	time (h)	yield (%)[b]
1	bpyNi-PHI (10 mg, 6.0 wt % Nibpy)	$^{i}Pr_2NH$	MeCN	6	98 (96)[c]
2	bpyNi-PHI (10 mg, 6.0 wt % Nibpy)	TMG	MeCN	6	trace
3	bpyNi-PHI (10 mg, 6.0 wt % Nibpy)	$^{i}Pr_2NH$	DMF	6	56
4	bpyNi-PHI (10 mg, 6.0 wt % Nibpy)	$^{i}Pr_2NH$	DMSO	6	45
5	dtbpyNi-PHI (10 mg, 5.9 wt % Nidtbpy)	$^{i}Pr_2NH$	MeCN	6	96 (94)[c]
6	dOMebpyNi-PHI (10 mg, 6.3 wt % NidOMebpy)	$^{i}Pr_2NH$	MeCN	6	95 (93)[c]
7	phenNi-PHI (10 mg, 6.2 wt % Niphen)	$^{i}Pr_2NH$	MeCN	6	98 (96)[c]
8	dClbpyNi-PHI (10 mg, 5.6 wt % NidClbpy)	$^{i}Pr_2NH$	MeCN	9	93 (90)[c]
9		$^{i}Pr_2NH$	MeCN	6	trace
10	bpyNi-PHI (10 mg, 6.0 wt % Nibpy)		MeCN	6	0
11[d]	bpyNi-PHI (10 mg, 6.0 wt % Nibpy)	$^{i}Pr_2NH$	MeCN	6	trace
12	Ni-PHI (10 mg, 3.2 wt % Ni)	$^{i}Pr_2NH$	MeCN	9	<5
13	K-PHI (10 mg) + $NiCl_2$ (1.4 mol%)	$^{i}Pr_2NH$	MeCN	9	<5
14	K-PHI (10 mg) + $(bpy)NiCl_2(H_2O)_n$ (1.4 mol%)	$^{i}Pr_2NH$	MeCN	6	46
15	bpyNi-PHI (1 mg, 6.0 wt % Nibpy)	$^{i}Pr_2NH$	MeCN	36	95
16	K-PHI (1 mg) + $(bpy)NiCl_2(H_2O)_n$ (0.14 mol%)	$^{i}Pr_2NH$	MeCN	36	9

[a]Reaction conditions: methyl 4-iodobenzoate (0.2 mmol, 1.0 eq.), methyl 3-mercaptopropanoate (0.3 mmol, 1.5 eq.), the corresponding catalyst, base (0.6 mmol, 3.0 eq.) in solvent (2 mL) under N_2 atmosphere and blue LEDs irradiation (24 W, 460 ± 5 nm) without extra heating (at 35 ± 5 °C) for the indicated time. [b]Determined by ^{1}H NMR analysis using 1,3,5-trimethoxybenzene as an internal standard. [c]Isolated yield in parenthesis. [d]In dark.

1.7 General procedures (GPs) for bpyNi-PHI catalyzed cross-couplings

bpyNi-PHI based heterogeneous photocatalytic C−P couplings (GP 1)

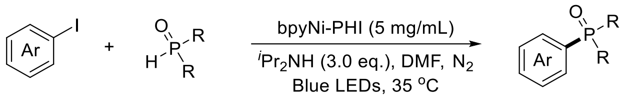

To a 10 mL oven-dried sealed tube equipped with a magnetic stir bar was added the corresponding aryl iodide (0.2 mmol, 1.0 eq.), H-phosphine oxide (0.3 mmol, 1.5 eq.) and bpyNi-PHI (10 mg, 6.0 wt % Nibpy). Then, dry DMF (2 mL) and $^{i}Pr_2NH$ (0.6 mmol, 3.0 eq.) were added. The tube was closed with a rubber septum and the reaction mixture was degassed by three cycles vacuum/N_2 of "freeze-pump-thaw". The reaction mixture was stirred and irradiated by blue LEDs (24 W, 460 ± 5 nm) without extra heating (35 ± 5 °C) for the indicated time. In each case, the blue LEDs was placed 3 cm from the reaction tube (Supplementary Figure 19a). An independent fan was used to maintain the temperature inside the irradiation reaction system. Upon completion, the reaction mixture was diluted with deionized water (5 mL) and extracted with ethyl acetate (3 × 5 mL). The combined organic layer was washed with brine, dried over anhydrous Na_2SO_4, and concentrated. Finally, the crude residue was purified by silica gel column chromatography. For comparison, two 40 W Kessil PR lamp (50% power, 456 nm) were used as alternative light sources (Supplementary Figure 19b), similar yield of methyl 4-(diphenylphosphoryl)benzoate (**1**, 48 h, 63.2 mg, 94%,) as obtained.

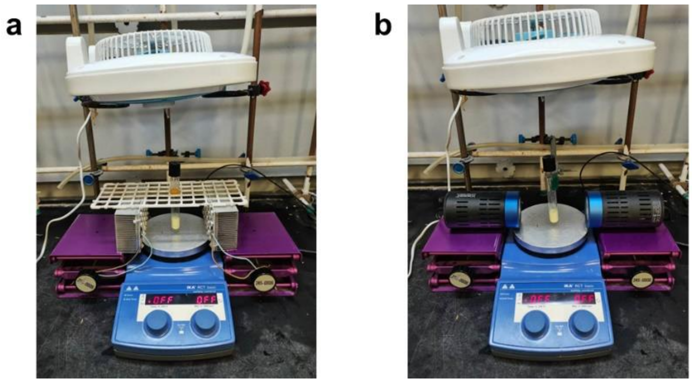

Supplementary Figure 19. Photograph for the photochemical reaction set-up: (a) Blue LED, (b) Kessil PR lamp.

bpyNi-PHI based heterogeneous photocatalytic C−N couplings (GP 2)

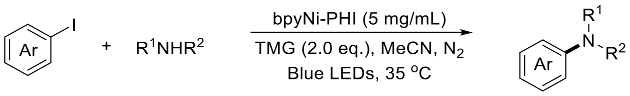

To a 10 mL oven-dried sealed tube equipped with a magnetic stir bar was added the corresponding aryl iodide (0.2 mmol, 1.0 eq.), amine (0.3 mmol, 1.5 eq.), and bpyNi-PHI (10 mg, 6.0 wt % Nibpy). Then, dry MeCN (2 mL) and TMG (0.4 mmol, 2.0 eq.) were added. The tube was closed with a rubber septum and the reaction mixture was degassed by three cycles vacuum/N_2 of "freeze-pump-thaw". After that the reaction mixture was stirred and irradiated by blue LEDs (24 W, 460 ± 5 nm) without extra heating (35 ± 5 °C) for the indicated time. An independent fan was used

to maintain the temperature inside the irradiation reaction system. In each case, the blue LEDs was placed 3 cm from the reaction tube (Supplementary Figure 19a). Upon completion, the reaction mixture was concentrated under reduced pressure to evaporate the solvent, and the crude residue was purified by silica gel column chromatography.

bpyNi-PHI based heterogeneous photocatalytic C−O couplings

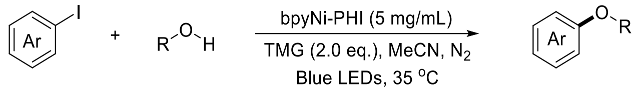

GP 3: To a 10 mL oven-dried sealed tube equipped with a magnetic stir bar was added the corresponding aryl iodide (0.2 mmol, 1.0 eq.), alcohol (1.0 mmol, 5.0 eq.), and bpyNi-PHI (10 mg, 6.0 wt % Nibpy). Then, dry MeCN (2 mL) and TMG (0.4 mmol, 2.0 eq.) were added. The tube was closed with a rubber septum and the reaction mixture was degassed by three cycles vacuum/N_2 of "freeze-pump-thaw". The reaction mixture was stirred and irradiated by blue LEDs (24 W, 460 ± 5 nm) without extra heating (35 ± 5 ºC) for the indicated time. In each case, the blue LEDs was placed 3 cm from the reaction tube (Supplementary Figure 19a). An independent fan was used to maintain the temperature inside the irradiation reaction system. Upon completion, the reaction mixture was concentrated under reduced pressure to evaporate the solvent, and the crude residue was purified by silica gel column chromatography.

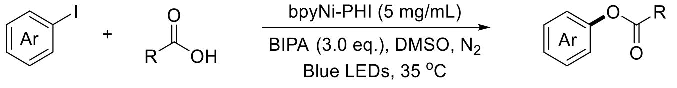

GP 4: To a 10 mL oven-dried sealed tube equipped with a magnetic stir bar was added the corresponding aryl iodide (0.2 mmol, 1.0 eq., if solid), carboxylic acid (0.3 mmol, 1.5 eq., if solid), and bpyNi-PHI (10 mg, 6.0 wt % Nibpy). Then, dry DMSO (2 mL) and BIPA (0.6 mmol, 3.0 eq.) were added. The tube was closed with a rubber septum and the reaction mixture was degassed by three cycles vacuum/N_2 of "freeze-pump-thaw". After that the reaction mixture was stirred and irradiated by blue LEDs (24 W, 460 ± 5 nm) without extra heating (35 ± 5 ºC) for the indicated time. In each case, the blue LEDs was placed 3 cm from the reaction tube (Supplementary Figure 19a). An independent fan was used to maintain the temperature inside the irradiation reaction system. Upon completion, the reaction mixture was diluted with deionized water (5 mL) and extracted with ethyl acetate (3 × 5 mL). The combined organic layer was washed with brine, dried over anhydrous Na_2SO_4, and concentrated. Finally, the crude residue was purified by silica gel column chromatography.

bpyNi-PHI based heterogeneous photocatalytic C−S couplings (GP 5)

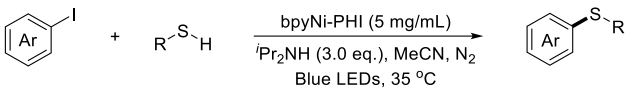

To a 10 mL oven-dried sealed tube equipped with a magnetic stir bar was added the corresponding aryl iodide (0.2 mmol, 1.0 eq.), thiol or thiolacid (0.3 mmol, 1.5 eq.), and bpyNi-PHI (10 mg, 6.0 wt % Nibpy). Then, dry MeCN (2 mL) and $^{i}Pr_2NH$ (0.6 mmol, 3.0 eq.) were added. The tube was closed with a rubber septum and the reaction mixture was degassed by three cycles vacuum/N_2 of "freeze-pump-thaw". After that the reaction mixture was stirred and irradiated by blue LEDs (24 W, 460 ± 5 nm) without extra heating (35 ± 5 °C) for the indicated time. In each case, the blue LEDs was placed 3 cm from the reaction tube (Supplementary Figure 19a). An independent fan was used to maintain the temperature inside the irradiation reaction system. Upon completion, the reaction mixture was concentrated under reduced pressure to evaporate the solvent, and the crude residue was purified by silica gel column chromatography.

1.8 Catalyst recycling

After completion of the reaction, the reaction mixture was centrifuged at 10000 rpm for 10 min to separate bpyNi-PHI and the liquid mixture. The recovered bpyNi-PHI was washed with MeCN (2 × 1 mL), centrifuged and dried in a high vacuum. Then the recovered bpyNi-PHI was suspended in reaction solvent (0.5 mL) and transferred to a new reaction tube. The falcon tube was washed with reaction solvent (2 × 0.5 mL) to ensure the complete transfer of the heterogeneous material and used for the next cycle. Afterward, the corresponding reactants and solvent (0.5 mL) were added. The tube was closed with a rubber septum and the reaction mixture was degassed by three cycles vacuum/N_2 of "freeze-pump-thaw". Then the reaction mixture was stirred and irradiated by blue LEDs (4 × 24 W, 460 ± 5 nm) without extra heating (35 ± 5 °C) for the indicated time. An independent fan was used to maintain the temperature inside the irradiation reaction system. More than 78% of the material can be recovered after the fifth cycle.

1.9 Scale-up experiments

Gram-scale synthesis of methyl 4-(diphenylphosphoryl)benzoate (1)

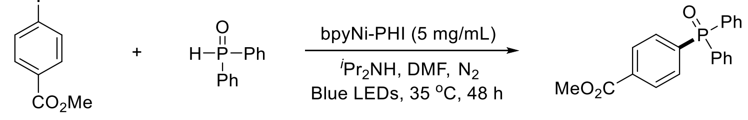

4 mmol 6 mmol **1**, 1.21 g, 90%

To a 100 mL oven-dried Schlenk tube equipped with a magnetic stir bar was added methyl 4-iodobenzoate (4.0 mmol, 1.0 eq.), diphenylphosphine oxide (6.0 mmol, 1.5 eq.) and bpyNi-PHI (200 mg, 6.0 wt % Nibpy). Then, dry DMF (40 mL) and $^{i}Pr_2NH$ (12 mmol, 3.0 eq.) were added. The resulting mixture was degassed via 'freeze-pump-thaw'procedure (3 times). Then the reaction mixture was stirred and irradiated by blue LEDs (4 × 24 W, 460 ± 5 nm) without extra heating (35 ± 5 °C) for

48 h. The blue LEDs was placed 5 cm from the reaction tube (Supplementary Figure 20). An independent fan was used to maintain the temperature inside the irradiation reaction system. Upon completion, the reaction mixture was diluted with deionized water (50 mL) and extracted with ethyl acetate (3 × 50 mL). The combined organic layer was washed with brine, dried over anhydrous Na_2SO_4, and concentrated. Finally, the crude residue was purified by silica gel column chromatography (petroleum ether/ethyl acetate 1/1) to afford product **1** (1.21 g, 90% yield).

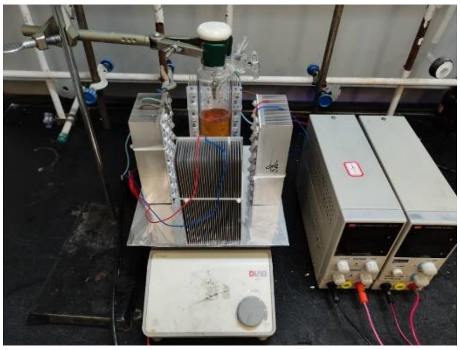

Supplementary Figure 20. Photograph for the photochemical reaction set-up in batch

Gram-scale synthesis of Fluoxetine (118)

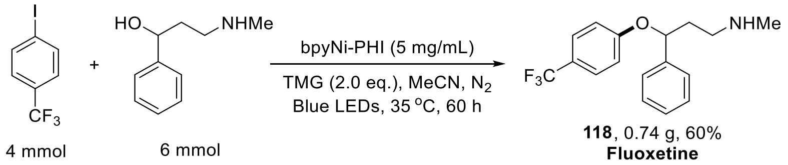

To a 100 mL oven-dried Schlenk tube equipped with a magnetic stir bar was added 1-iodo-4-(trifluoromethyl) benzene (4 mmol, 1.0 eq.), racemic 3-(methylamino)-1-phenylpropan-1-ol (6 mmol, 1.5 eq.) and bpyNi-PHI (200 mg, 6.0 wt % Nibpy). Then, dry MeCN (40 mL) and TMG (8 mmol, 2.0 eq.) were added. The resulting mixture was degassed via 'freeze-pump-thaw' procedure (3 times). Then the Schlenk tube was irradiated by blue LEDs (4 × 24 W, 460 ± 5 nm) and the reaction mixture was stirred without extra heating (35 ± 5 °C) for 60 h. The blue LEDs was placed 5 cm from the reaction tube (Supplementary Figure 20). An independent fan was used to maintain the temperature inside the irradiation reaction system. Upon completion, the solvent was removed in vacuo, and the residue was purified by silica gel column chromatography (petroleum ether/ethyl acetate 5/1) to afford product **118** (0.74 g, 60% yield).

1.10 Structure characterization of the recovered bpyNi-PHI

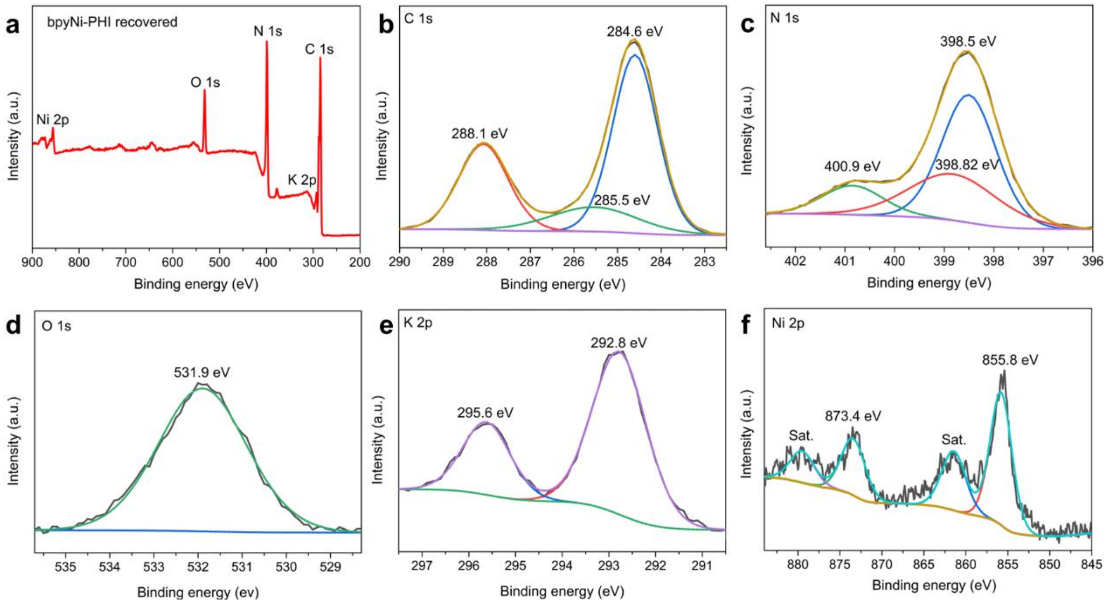

Supplementary Figure 21. XPS spectra of recovered bpyNi-PHI: (a) survey; (b) C 1s; (c) N 1s; (d) O 1s; (e) K 2p; (f) Ni 2p.

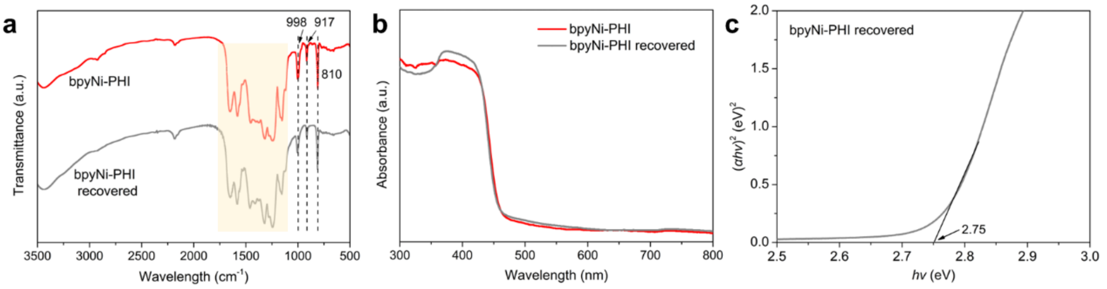

Supplementary Figure 22. (a) FTIR spectra and (b) UV-vis spectra of bpyNi-PHI and recovered bpyNi-PHI, (c) Tauc plots of recovered bpyNi-PHI.

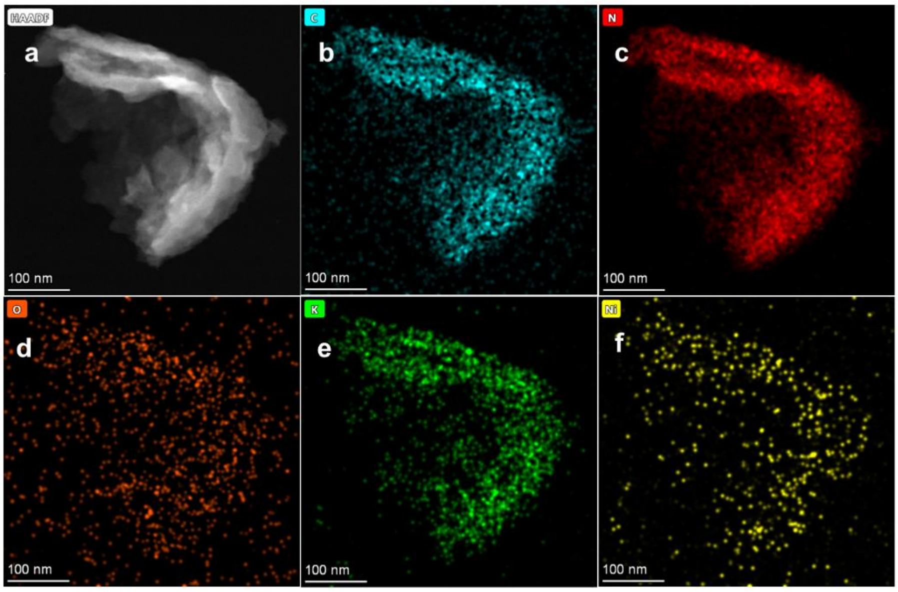

Supplementary Figure 23. (a-f) TEM image of recovered bpyNi-PHI with corresponding elemental mapping. The colors of blue, red, orange, green and yellow represent the elemental components of C, N, O, K and Ni respectively.

1.11 Characterization data for the products

Methyl 4-(diphenylphosphoryl)benzoate[14] (1)

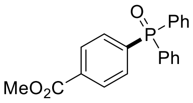

1

According to **GP1** starting from methyl 4-iodobenzoate (0.2 mmol, 1.0 eq.), diphenylphosphine oxide (0.3 mmol, 1.5 eq.), $^{i}Pr_2NH$ (0.6 mmol, 3.0 eq.) and bpyNi-PHI (10 mg, 6.0 wt % Nibpy) in DMF (2 mL) for 42 h, the product **1** was isolated as white solid after flash chromatography (petroleum ether/ethyl acetate 1/1), 64.5 mg (96% yield). **^{1}H NMR** (400 MHz, $CDCl_3$) δ 8.15 – 8.09 (m, 2H), 7.78 (dd, *J* = 11.7, 8.1 Hz, 2H), 7.72 – 7.62 (m, 4H), 7.60 – 7.53 (m, 2H), 7.52 – 7.44 (m, 4H), 3.93 (s, 3H). **^{13}C NMR** (101 MHz, $CDCl_3$) δ 166.2, 137.6 (d, *J* = 100.7 Hz), 133.2 (d, *J* = 2.9 Hz), 132.3 (d, *J* = 2.8 Hz), 132.1 (d, *J* = 10.2 Hz), 132.0 (d, *J* = 10.0 Hz), 131.8 (d, *J* = 104.7 Hz), 129.4 (d, *J* = 12.2 Hz), 128.7 (d, *J* = 12.3 Hz), 52.5. **^{31}P NMR** (162 MHz, $CDCl_3$) δ 28.4.

4-methyl-*N*-phenylaniline[15] (2)

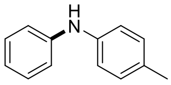

2

According to **GP2** starting from iodobenzene (0.2 mmol, 1.0 eq.), *p*-toluidine (0.3 mmol, 1.5 eq.), TMG (0.4 mmol, 2.0 eq.) and bpyNi-PHI (10 mg, 6.0 wt % Nibpy) in MeCN (2 mL) for 12 h, the product **2** was isolated as white solid after flash chromatography (petroleum ether/ethyl acetate 30/1), 34.8 mg (95% yield). **^{1}H NMR** (500 MHz, $CDCl_3$) δ 7.22 (t, *J* = 7.7 Hz, 2H), 7.07 (d, *J* = 8.0 Hz, 2H), 7.02 – 6.95 (m, 4H), 6.87 (t, *J* = 7.4 Hz, 1H), 5.57 (br, 1H), 2.29 (s, 3H). **^{13}C NMR** (101 MHz, $CDCl_3$) δ 144.0, 140.3, 131.0, 129.9, 129.3, 120.3, 119.0, 116.9, 20.7.

Methyl 4-(hexyloxy)benzoate[16] (3)

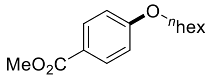

3

According to **GP3** starting from methyl 4-iodobenzoate (0.2 mmol, 1.0 eq.), hexan-1-ol (1 mmol, 5.0 eq.), TMG (0.4 mmol, 2.0 eq.) and bpyNi-PHI (10 mg, 6.0 wt % Nibpy) in MeCN (2 mL) for 12 h, the product **3** was isolated as pale yellow solid after flash chromatography (petroleum ether/ethyl acetate 20/1), 40.2 mg (85% yield). **^{1}H NMR** (500 MHz, $CDCl_3$) δ 7.98 (d, *J* = 8.9 Hz, 2H), 6.90 (d, *J* = 8.9 Hz, 2H), 4.00 (t, *J* = 6.6 Hz, 2H), 3.88 (s, 3H), 1.84 – 1.75 (m, 2H), 1.49 – 1.42 (m, 2H), 1.38 – 1.31 (m, 4H), 0.93 – 0.88 (m, 3H). **^{13}C NMR** (126 MHz, $CDCl_3$) δ 166.9, 163.0, 131.6, 122.3, 114.1, 68.2, 51.8, 31.6, 29.1, 25.7, 22.6, 14.0.

Methyl 4-(benzoyloxy)benzoate[17] (**4**)

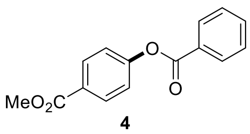

4

According to **GP4** starting from methyl 4-iodobenzoate (0.2 mmol, 1.0 eq.), benzoic acid (0.3 mmol, 1.5 eq.), BIPA (0.6 mmol, 3.0 eq.) and bpyNi-PHI (10 mg, 6.0 wt % Nibpy) in DMSO (2 mL) for 10 h, the product **4** was isolated as white solid after flash chromatography (petroleum ether/ethyl acetate 10/1), 44.1 mg (86% yield). **^{1}H NMR** (400 MHz, $CDCl_3$) δ 8.20 (d, *J* = 6.9 Hz, 2H), 8.13 (d, *J* = 8.7 Hz, 2H), 7.65 (t, *J* = 7.4 Hz, 1H), 7.52 (t, *J* = 7.8 Hz, 2H), 7.31 (d, *J* = 8.8 Hz, 2H), 3.93 (s, 3H). **^{13}C NMR** (101 MHz, $CDCl_3$) δ 165.3, 163.6, 153.6, 132.9, 130.2, 129.2, 128.1, 127.6, 126.7, 120.7, 51.2.

Methyl 4-((3-methoxy-3-oxopropyl)thio)benzoate[16] (5)

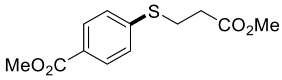

5

According to **GP5** starting from methyl 4-iodobenzoate (0.2 mmol, 1.0 eq.), methyl 3-mercaptopropanoate (0.3 mmol, 1.5 eq.), ^{i}Pr$_2$NH (0.6 mmol, 3.0 eq.) and bpyNi-PHI (10 mg, 6.0 wt % Nibpy) in MeCN (2 mL) for 6 h, the product **5** was isolated as white solid after flash chromatography (petroleum ether/ethyl acetate 5/1), 48.8 mg (96% yield). **^{1}H NMR** (400 MHz, $CDCl_3$) δ 7.94 (d, *J* = 8.3 Hz, 2H), 7.32 (d, *J* = 8.3 Hz, 2H), 3.90 (s, 3H), 3.70 (s, 3H), 3.26 (t, *J* = 7.4 Hz, 2H), 2.69 (t, *J* = 7.4 Hz, 2H). **^{13}C NMR** (101 MHz, $CDCl_3$) δ 171.9, 166.7, 142.6, 130.1, 127.3, 127.1, 52.1, 51.9, 33.8, 27.3.

1-(4-(diphenylphosphoryl)phenyl)ethan-1-one[14] (6)

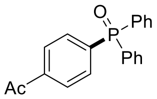

6

According to **GP1** starting from 1-(4-iodophenyl)ethan-1-one (0.2 mmol, 1.0 eq.), diphenylphosphine oxide (0.3 mmol, 1.5 eq.), (0.6 mmol, 3.0 eq.) and bpyNi-PHI (10 mg, 6.0 wt % Nibpy) in DMF (2 mL) for 48 h, the product **6** was isolated as white solid after flash chromatography (petroleum ether/ethyl acetate 1/1), 54.4 mg (85% yield). **^{1}H NMR** (400 MHz, $CDCl_3$) δ 8.03 (dd, *J* = 8.4, 2.4 Hz, 2H), 7.80 (dd, *J* = 11.5, 8.3 Hz, 2H), 7.70 – 7.63 (m, 4H), 7.61 – 7.55 (m, 2H), 7.53 – 7.45 (m, 4H), 2.63 (s, 3H). **^{13}C NMR** (101 MHz, $CDCl_3$) δ 197.6, 139.5 (d, *J* = 2.7 Hz), 137.7 (d, *J* = 100.8 Hz), 132.4 (d, *J* = 9.9 Hz), 132.3 (d, *J* = 2.8 Hz), 132.0 (d, *J* = 10.0 Hz), 131.8 (d, *J* = 104.9 Hz), 128.7 (d, *J* = 12.3 Hz), 128.1 (d, *J* = 12.1 Hz), 26.8. **^{31}P NMR** (162 MHz, $CDCl_3$) δ 28.3.

4-(diphenylphosphoryl)benzonitrile[14] **(7)**

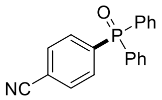

7

According to **GP1** starting from 4-iodobenzonitrile (0.2 mmol, 1.0 eq.), diphenylphosphine oxide (0.3 mmol, 1.5 eq.), $^{i}Pr_2NH$ (0.6 mmol, 3.0 eq.) and bpyNi-PHI (10 mg, 6.0 wt % Nibpy) in DMF (2 mL) for 24 h, the product **7** was isolated as white solid after flash chromatography (petroleum ether/ethyl acetate 1/1), 54.6 mg (90% yield). **^{1}H NMR** (400 MHz, $CDCl_3$) δ 7.83 – 7.72 (m, 4H), 7.69 – 7.56 (m, 6H), 7.55 – 7.45 (m, 4H). **^{13}C NMR** (101 MHz, $CDCl_3$) δ 138.5 (d, J = 99.2 Hz), 132.6 (d, J = 13.6 Hz), 132.6, 132.1, 132.0, 131.2 (d, J = 105.4 Hz), 128.8 (d, J = 12.4 Hz), 117.9 (d, J = 1.5 Hz), 115.6 (d, J = 3.0 Hz). **^{31}P NMR** (162 MHz, $CDCl_3$) δ 27.8.

(4-chlorophenyl)diphenylphosphine oxide[18] **(8)**

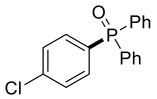

8

According to **GP1** starting from 1-chloro-4-iodobenzene (0.2 mmol, 1.0 eq.), diphenylphosphine oxide (0.3 mmol, 1.5 eq.), $^{i}Pr_2NH$ (0.6 mmol, 3.0 eq.) and bpyNi-PHI (10 mg, 6.0 wt % Nibpy) in DMF (2 mL) for 48 h, the product **8** was isolated as white solid after flash chromatography (petroleum ether/ethyl acetate 2/1), 50.0 mg (80% yield). **^{1}H NMR** (400 MHz, $CDCl_3$) δ 7.70 – 7.52 (m, 8H), 7.52 – 7.41 (m, 6H). **^{13}C NMR** (101 MHz, $CDCl_3$) δ 138.6 (d, J = 3.3 Hz), 133.5 (d, J = 10.7 Hz), 132.2 (d, J = 2.8 Hz), 132.1 (d, J = 105.0 Hz), 132.0 (d, J = 10.1 Hz), 131.2 (d, J = 104.6 Hz), 128.9 (d, J = 12.7 Hz), 128.6 (d, J = 12.3 Hz). **^{31}P NMR** (162 MHz, $CDCl_3$) δ 28.5.

(4-bromophenyl)diphenylphosphine oxide[18] **(9)**

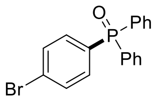

9

According to **GP1** starting from 1-bromo-4-iodobenzene (0.2 mmol, 1.0 eq.), diphenylphosphine oxide (0.3 mmol, 1.5 eq.), $^{i}Pr_2NH$ (0.6 mmol, 3.0 eq.) and bpyNi-PHI (10 mg, 6.0 wt % Nibpy) in DMF (2 mL) for 48 h, the product **9** was isolated as white solid after flash chromatography (petroleum ether/ethyl acetate 2/1), 50.0 mg (70% yield). **^{1}H NMR** (400 MHz, $CDCl_3$) δ 7.69 – 7.64 (m, 3H), 7.64 – 7.60 (m, 3H), 7.59 – 7.51 (m, 4H), 7.50 – 7.44 (m, 4H). **^{13}C NMR** (101 MHz, $CDCl_3$) δ 133.6 (d, J = 10.6 Hz), 132.2 (d, J = 2.8 Hz), 132.0 (d, J = 104.9 Hz), 132.0 (d, J = 9.9 Hz), 131.8 (d, J = 12.4 Hz), 130.1 (d, J = 224.3 Hz), 128.7 (d, J = 12.2 Hz), 127.2 (d, J = 3.4 Hz). **^{31}P NMR** (162 MHz, $CDCl_3$) δ 28.5.

Triphenylphosphine oxide[18] **(10)**

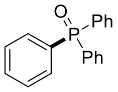

10

According to **GP1** starting from iodobenzene (0.2 mmol, 1.0 eq.), diphenylphosphine oxide (0.3 mmol, 1.5 eq.), $^{i}Pr_2NH$ (0.6 mmol, 3.0 eq.) and bpyNi-PHI (10 mg, 6.0 wt % Nibpy) in DMF (2 mL) for 48 h, the product **10** was isolated as white solid after flash chromatography (petroleum ether/ethyl acetate 1/1), 50.1 mg (90% yield). **^{1}H NMR** (400 MHz, $CDCl_3$) δ 7.70 – 7.63 (m, 6H), 7.58 – 7.52 (m, 3H), 7.49 – 7.42 (m, 6H). **^{13}C NMR** (101 MHz, $CDCl_3$) δ 132.4 (d, J = 104.6 Hz) 132.11 (d, J = 10.0 Hz), 131.97 (d, J = 2.6 Hz), 128.52 (d, J = 12.2 Hz). **^{31}P NMR** (162 MHz, $CDCl_3$) δ 29.3.

Diphenyl(*p*-tolyl)phosphine oxide[14] **(11)**

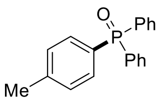

11

According to **GP1** starting from 1-iodo-4-methylbenzene (0.2 mmol, 1.0 eq.), diphenylphosphine oxide (0.3 mmol, 1.5 eq.), $^{i}Pr_2NH$ (0.6 mmol, 3.0 eq.) and bpyNi-PHI (10 mg, 6.0 wt % Nibpy) in DMF (2 mL) for 48 h, the product **11** was isolated as white solid after flash chromatography (petroleum ether/ethyl acetate 2/1), 55.0 mg (94% yield). **^{1}H NMR** (400 MHz, $CDCl_3$) δ 7.70 – 7.63 (m, 4H), 7.59 – 7.50 (m, 4H), 7.48 – 7.39 (m, 4H), 7.27 – 7.23 (m, 2H), 2.39 (s, 3H). **^{13}C NMR** (101 MHz, $CDCl_3$) δ 142.5 (d, *J* = 2.7 Hz), 132.8 (d, *J* = 104.0 Hz), 132.1 (d, *J* = 10.2 Hz), 132.1 (d, *J* = 10.0 Hz), 131.8 (d, *J* = 2.7 Hz), 129.3 (d, *J* = 12.6 Hz), 129.2 (d, *J* = 106.6 Hz), 128.4 (d, *J* = 12.1 Hz), 21.6 (d, *J* = 1.4 Hz). **^{31}P NMR** (162 MHz, $CDCl_3$) δ 29.2.

(4-methoxyphenyl)diphenylphosphine oxide[14] **(12)**

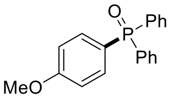

12

According to **GP1** starting from 1-iodo-4-methoxybenzene (0.2 mmol, 1.0 eq.), diphenylphosphine oxide (0.3 mmol, 1.5 eq.), $^{i}Pr_2NH$ (0.6 mmol, 3.0 eq.) and bpyNi-PHI (10 mg, 6.0 wt % Nibpy) in DMF (2 mL) for 48 h, the product **12** was isolated as colorless oil after flash chromatography (petroleum ether/ethyl acetate 1/1), 50.6 mg (82% yield). **^{1}H NMR** (400 MHz, $CDCl_3$) δ 7.71 – 7.61 (m, 4H), 7.63 – 7.49 (m, 4H), 7.48 – 7.42 (m, 4H), 6.97 (dd, *J* = 8.8, 2.3 Hz, 2H), 3.84 (s, 3H). **^{13}C NMR** (101 MHz, $CDCl_3$) δ 162.5, 134.0 (d, *J* = 11.5 Hz), 133.0 (d, *J* = 104.1 Hz), 132.1 (d, *J* = 9.9 Hz), 131.8, 128.5 (d, *J* = 12.0 Hz), 123.6 (d, *J* = 110.3 Hz), 114.1 (d, *J* = 13.4 Hz), 55.4. **^{31}P NMR** (162 MHz, $CDCl_3$) δ 29.1.

(4-hydroxyphenyl)diphenylphosphine oxide[19] **(13)**

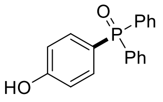

13

According to **GP1** starting from 4-iodophenol (0.2 mmol, 1.0 eq.), diphenylphosphine oxide (0.3 mmol, 1.5 eq.), $^{i}Pr_2NH$ (0.6 mmol, 3.0 eq.) and bpyNi-PHI (10 mg, 6.0 wt % Nibpy) in DMF (2 mL) for 48 h, the product **13** was isolated as white solid after flash chromatography (petroleum ether/ethyl acetate 1/1), 47.1mg (80% yield). **^{1}H NMR** (400 MHz, CD_3OD) δ 7.70 – 7.61 (m, 6H), 7.60 – 7.52 (m, 4H), 7.51 – 7.42 (m, 2H), 6.95 (dd, *J* = 8.7, 2.4 Hz, 2H). **^{13}C NMR** (101 MHz, CD_3OD) δ 161.6 (d, *J* = 2.8 Hz), 133.8 (d, *J* = 11.6 Hz), 132.1 (d, *J* = 2.8 Hz), 131.9 (d, *J* = 105.9 Hz), 131.7 (d, *J* = 10.1 Hz), 128.5 (d, *J* = 12.3 Hz), 120.2 (d, *J* = 113.7 Hz), 115.5 (d, *J* = 13.5 Hz). **^{31}P NMR** (162 MHz, CD_3OD) δ 33.0.

(4-aminophenyl)diphenylphosphine oxide[19] **(14)**

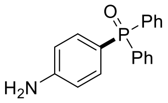

14

According to **GP1** starting from 4-iodoaniline (0.2 mmol, 1.0 eq.), diphenylphosphine oxide (0.3 mmol, 1.5 eq.), $^{i}Pr_2NH$ (0.6 mmol, 3.0 eq.) and bpyNi-PHI (10 mg, 6.0 wt % Nibpy) in DMF (2 mL) for 48 h, the product **14** was isolated as white solid after flash chromatography(petroleum ether/ethyl acetate 1/1), 54.0 mg (92% yield). **^{1}H NMR** (400 MHz, $CDCl_3$) δ 7.71 – 7.61 (m, 4H), 7.55 – 7.48 (m, 2H), 7.47 – 7.35 (m, 6H), 6.68 (dd, *J* = 8.6, 2.4 Hz, 2H), 4.06 (s, 2H). **^{13}C NMR** (101 MHz, $CDCl_3$) δ149.9 (d, *J* = 2.8 Hz), 133.8 (d, *J* = 11.2 Hz), 133.3 (d, *J* = 104.3 Hz), 132.1 (d, *J* = 9.8 Hz), 131.6 (d, *J* = 2.7 Hz), 128.4 (d, *J* = 12.0 Hz), 119.9 (d, *J* = 113.7 Hz),

114.3 (d, J = 13.2 Hz). **^{31}P NMR** (162 MHz, $CDCl_3$) δ 29.6.

Diphenyl(m-tolyl)phosphine oxide[20] (15)

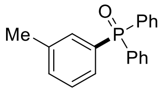

15

According to **GP1** starting from 1-iodo-3-methylbenzene (0.2 mmol, 1.0 eq.), diphenylphosphine oxide (0.3 mmol, 1.5 eq.), $^{i}Pr_2NH$ (0.6 mmol, 3.0 eq.) and bpyNi-PHI (10 mg, 6.0 wt % Nibpy) in DMF (2 mL) for 48 h, the product **15** was isolated as white solid after flash chromatography (petroleum ether/ethyl acetate 1/1), 51.4 mg (88% yield). **^{1}H NMR** (400 MHz, $CDCl_3$) δ 7.71 – 7.62 (m, 4H), 7.61 – 7.50 (m, 3H), 7.48 – 7.41 (m, 4H), 7.41 – 7.29 (m, 3H), 2.35 (s, 3H). **^{13}C NMR** (101 MHz, $CDCl_3$) δ 138.5 (d, J = 12.0 Hz), 133.2, 132.8 (d, J = 2.7 Hz), 132.5 (d, J = 9.4 Hz), 132.1 (d, J = 9.8 Hz), 131.9 (d, J = 2.7 Hz), 131.8, 129.2 (d, J = 10.2 Hz), 128.5 (d, J = 12.1 Hz), 128.3 (d, J = 12.9 Hz), 21.4. ^{31}P NMR (162 MHz, $CDCl_3$) δ 29.3.

(2-methoxyphenyl)diphenylphosphine oxide[21] (16)

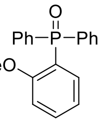

16

According to **GP1** starting from 1-iodo-2-methoxybenzene(0.2 mmol, 1.0 eq.), diphenylphosphine oxide (0.3 mmol, 1.5 eq.), $^{i}Pr_2NH$ (0.6 mmol, 3.0 eq.) and bpyNi-PHI (10 mg, 6.0 wt % Nibpy) in DMF (2 mL) for 60 h, the product **16** was isolated as white solid after flash chromatography (petroleum ether/ethyl acetate 1/1), 46.0 mg (74% yield). **^{1}H NMR** (400 MHz, $CDCl_3$) δ 7.82 – 7.66 (m, 5H), 7.57 – 7.48 (m, 3H), 7.47 – 7.36 (m, 4H), 7.11 – 7.05 (m, 1H), 6.92 (dd, J = 8.3, 5.2 Hz, 1H), 3.56 (s, 3H). **^{13}C NMR** (101 MHz, $CDCl_3$) δ 160.9 (d, J = 3.3 Hz), 135.0 (d, J = 7.1 Hz), 134.3 (d, J = 2.1 Hz), 133.2 (d, J = 107.5 Hz), 131.8 (d, J = 10.2 Hz), 131.5 (d, J = 2.8 Hz), 128.1 (d, J = 12.4 Hz), 121.0 (d, J = 11.7 Hz), 120.3 (d, J = 102.2 Hz), 111.4 (d, J = 6.5 Hz), 55.3. **^{31}P NMR** (162 MHz, $CDCl_3$) δ 27.4.

Naphthalen-1-yldiphenylphosphine oxide[21] (17)

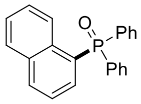

17

According to **GP1** starting from 1-iodonaphthalene (0.2 mmol, 1.0 eq.), diphenylphosphine oxide (0.3 mmol, 1.5 eq.), $^{i}Pr_2NH$ (0.6 mmol, 3.0 eq.) and bpyNi-PHI (10 mg, 6.0 wt % Nibpy) in DMF (2 mL) for 48 h, the product **17** was isolated as white solid after flash chromatography (petroleum ether/ethyl acetate 1/1), 63.0 mg (96% yield). **^{1}H NMR** (400 MHz, $CDCl_3$) δ 8.59 (d, J = 8.4 Hz, 1H), 8.01 (d, J = 8.1 Hz, 1H), 7.88 (d, J = 8.1 Hz, 1H), 7.73 – 7.64 (m, 4H), 7.59 – 7.41 (m, 8H), 7.40 – 7.27 (m, 2H). **^{13}C NMR** (101 MHz, $CDCl_3$) δ 133.9 (d, J = 17.9 Hz), 133.9 (d, J = 2.3 Hz), 133.7 (d, J = 3.2 Hz), 133.3 (d, J = 1.9 Hz), 132.8 (d, J = 99.5 Hz), 132.1 (d, J = 9.8 Hz), 131.9 (d, J = 2.9 Hz), 128.9 (d, J = 102.1 Hz), 128.8 (d, J = 1.4 Hz), 128.6 (d, J = 12.1 Hz), 127.6 (d, J = 5.8 Hz), 127.0 (d, J = 85.9 Hz), 124.2 (d, J = 14.3 Hz). **^{31}P NMR** (162 MHz, $CDCl_3$) δ 32.4.

Diphenyl(pyridin-4-yl)phosphine oxide[14] (18)

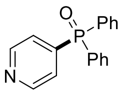

18

According to **GP1** starting from 4-iodopyridine (0.2 mmol, 1.0 eq.), diphenylphosphine oxide (0.3 mmol, 1.5 eq.), $^{i}Pr_2NH$ (0.6 mmol, 3.0 eq.) and bpyNi-PHI (10 mg, 6.0 wt % Nibpy) in DMF (2 mL) for 48 h, the product **18** was isolated as white solid after flash chromatography (petroleum ether/ethyl acetate 1/1), 52.5 mg (94% yield). **^{1}H NMR** (400 MHz, $CDCl_3$) δ 8.76 (s, 2H), 7.67 (dd, *J* = 12.2, 7.1 Hz, 4H), 7.63 – 7.56 (m, 3H), 7.55 – 7.47 (m, 5H). **^{13}C NMR** (101 MHz, $CDCl_3$) δ 150.0 (d, *J* = 9.4 Hz), 142.1 (d, *J* = 96.8 Hz), 132.6 (d, *J* = 2.8 Hz), 132.0 (d, *J* = 10.0 Hz), 130.9 (d, *J* = 105.5 Hz), 128.8 (d, *J* = 12.3 Hz), 125.7 (d, *J* = 7.8 Hz). **^{31}P NMR** (162 MHz, $CDCl_3$) δ 27.0.

(1*H*-indol-5-yl)diphenylphosphine oxide[21] (19)

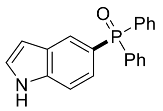

19

According to **GP1** starting from 5-iodo-1*H*-indole (0.2 mmol, 1.0 eq.), diphenylphosphine oxide (0.3 mmol, 1.5 eq.), $^{i}Pr_2NH$ (0.6 mmol, 3.0 eq.) and bpyNi-PHI (10 mg, 6.0 wt % Nibpy) in DMF (2 mL) for 48 h, the product **19** was isolated as white solid after flash chromatography (dichloromethane/methanol 20/1), 52.0 mg (82% yield). **^{1}H NMR** (400 MHz, $CDCl_3$) δ 10.36 (s, 1H), 7.84 (d, *J* = 13.3 Hz, 1H), 7.69 (dd, *J* = 11.9, 7.5 Hz, 4H), 7.55 – 7.35 (m, 7H), 7.34 – 7.28 (m, 1H), 7.19 (s, 1H), 6.46 (s, 1H). **^{13}C NMR** (101 MHz, $CDCl_3$) δ 138.1 (d, *J* = 2.5 Hz), 133.3 (d, *J* = 103.9 Hz), 132.2 (d, *J* = 9.9 Hz), 131.7 (d, *J* = 2.6 Hz), 128.4 (d, *J* = 12.0 Hz), 127.6 (d, *J* = 15.0 Hz), 126.4, 126.0 (d, *J* = 12.0 Hz), 124.4 (d, *J* = 11.9 Hz), 121.1 (d, *J* = 110.1 Hz), 112.1 (d, *J* = 13.8 Hz), 102.7. **^{31}P NMR** (162 MHz, $CDCl_3$) δ 32.6.

(2-chloro-3-methylpyridin-4-yl)diphenylphosphine oxide (20)

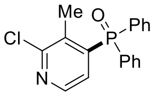

20

According to **GP1** starting from 2-chloro-4-iodo-3-methylpyridine (0.2 mmol, 1.0 eq.), diphenylphosphine oxide (0.3 mmol, 1.5 eq.), $^{i}Pr_2NH$ (0.6 mmol, 3.0 eq.) and bpyNi-PHI (10 mg, 6.0 wt % Nibpy) in DMF (2 mL) for 48 h, the product **20** was isolated as white solid after flash chromatography (dichloromethane/methanol 20/1), 57.7 mg (88% yield). mp = 139–140 °C. **^{1}H NMR** (400 MHz, $CDCl_3$) δ 8.24 (t, *J* = 4.4 Hz, 1H), 7.69 – 7.58 (m, 6H), 7.57 – 7.40 (m, 4H), 6.85 (dd, *J* = 12.7, 4.8 Hz, 1H), 2.52 (s, 3H). **^{13}C NMR** (101 MHz, $CDCl_3$) δ 154.2 (d, *J* = 12.1 Hz), 146.5 (d, *J* = 12.1 Hz), 143.4 (d, *J* = 94.4 Hz), 136.4 (d, *J* = 7.7 Hz), 132.7 (d, *J* = 2.8 Hz), 131.7 (d, *J* = 9.9 Hz), 130.8 (d, *J* = 105.7 Hz), 129.0 (d, *J* = 12.4 Hz), 125.7 (d, *J* = 11.0 Hz), 18.6 (d, *J* = 4.7 Hz). **^{31}P NMR** (162 MHz, $CDCl_3$) δ 29.8. **HRMS (ESI)** m/z calcd. For $C_{18}H_{16}ClNOP$ $[M+H]^+$ 328.0653, found 328.0650.

Methyl 4-(diethoxyphosphoryl)benzoate[22] (21)

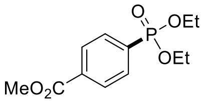

21

According to **GP1** starting from methyl 4-iodobenzoate (0.2 mmol, 1.0 eq.), diethyl phosphonate (0.3 mmol, 1.5 eq.), $^{i}Pr_2NH$ (0.6 mmol, 3.0 eq.) and bpyNi-PHI (10 mg, 6.0 wt % Nibpy) in DMF (2 mL) for 48 h, the product **21** was isolated as colorless oil after flash chromatography (petroleum ether/ethyl acetate 1/1), 49.0 mg (90% yield). **^{1}H NMR** (400 MHz, $CDCl_3$) δ 8.12 (dd, J = 8.3, 3.8 Hz, 2H), 7.89 (dd, J = 13.0, 8.3 Hz, 2H), 4.28 – 4.04 (m, 4H), 3.95 (s, 3H), 1.33 (t, J = 7.1 Hz, 6H). **^{13}C NMR** (101 MHz, $CDCl_3$) δ 166.2, 133.5 (d, J = 3.3 Hz), 133.3 (d, J = 186.7 Hz), 131.8 (d, J = 10.0 Hz), 129.4 (d, J = 15.0 Hz), 62.4 (d, J = 5.5 Hz), 52.5, 16.3 (d, J = 6.4 Hz). **^{31}P NMR** (162 MHz, $CDCl_3$) δ 17.0.

Diphenylamine[15] (22)

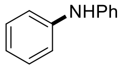

22

According to **GP2** starting from iodobenzene (0.2 mmol, 1.0 eq.), aniline (0.3 mmol, 1.5 eq.), TMG (0.4 mmol, 2.0 eq.) and bpyNi-PHI (10 mg, 6.0 wt % Nibpy) in MeCN (2 mL) for 12 h, the product **22** was isolated as white solid after flash chromatography (petroleum ether/ethyl acetate 30/1), 30.8 mg (91% yield). **^{1}H NMR** (400 MHz, $CDCl_3$) δ 7.32 – 7.23 (m, 4H), 7.13 – 7.04 (m, 4H), 6.98 – 6.90 (m, 2H), 5.69 (br, 1H). **^{13}C NMR** (101 MHz, $CDCl_3$) δ 143.2, 129.4, 121.0, 117.9.

4-methyl-*N*-phenylaniline[15] (23)

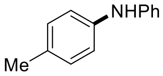

23

According to **GP2** starting from 1-iodo-4-methylbenzene(0.2 mmol, 1.0 eq.), aniline (0.3 mmol, 1.5 eq.), TMG (0.4 mmol, 2.0 eq.) and bpyNi-PHI (10 mg, 6.0 wt % Nibpy) in MeCN (2 mL) for 12 h, the product **23** was isolated as white solid after flash chromatography (petroleum ether/ethyl acetate 30/1), 34.1 mg (93% yield). **^{1}H NMR** (500 MHz, $CDCl_3$) δ 7.22 (t, J = 7.7 Hz, 2H), 7.07 (d, J = 8.0 Hz, 2H), 7.02 – 6.95 (m, 4H), 6.87 (t, J = 7.4 Hz, 1H), 5.57 (br, 1H), 2.29 (s, 3H). **^{13}C NMR** (126 MHz, $CDCl_3$) δ 144.0, 140.4, 131.0, 129.9, 129.4, 120.4, 119.0, 116.9, 20.7.

4-methoxy-*N*-phenylaniline[15] (24)

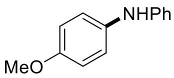

24

According to **GP2** starting from 1-iodo-4-methoxybenzene (0.2 mmol, 1.0 eq.), aniline (0.3 mmol, 1.5 eq.), TMG (0.4 mmol, 2.0 eq.) and bpyNi-PHI (10 mg, 6.0 wt % Nibpy) in MeCN (2 mL) for 12 h, the product **24** was isolated as white solid after flash chromatography (petroleum ether/ ethyl acetate 30/1), 37.1 mg (93% yield). **^{1}H NMR** (500 MHz, $CDCl_3$) δ 7.22 – 7.17 (m, 2H), 7.06 (d, J = 8.9 Hz, 2H), 6.89 (d, J = 7.7 Hz, 2H), 6.88 – 6.79 (m, 3H), 5.47 (br, 1H), 3.78 (s, 3H). **^{13}C NMR** (126 MHz, $CDCl_3$) δ 155.3, 145.2, 135.8, 129.4, 122.3, 119.6, 115.7, 114.7, 55.6.

4-bromo-*N*-phenylaniline[23] (25)

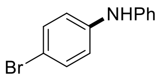

25

According to **GP2** starting from 1-bromo-4-iodobenzene (0.2 mmol, 1.0 eq.), aniline (0.3 mmol, 1.5 eq.), TMG (0.4 mmol, 2.0 eq.) and bpyNi-PHI (10 mg, 6.0 wt % Nibpy) in MeCN (2 mL) for 12 h, the product **25** was isolated as white solid after flash chromatography (petroleum ether/ethyl acetate 10/1), 42.7 mg (86% yield). **^{1}H NMR** (400 MHz, $CDCl_3$) δ 7.38 – 7.31 (m, 2H), 7.31 – 7.26 (m, 2H), 7.08 – 7.02 (m, 2H), 6.99 – 6.91 (m, 3H), 5.68 (br, 1H). **^{13}C NMR** (101 MHz, $CDCl_3$) δ 142.4, 132.2, 129.5, 121.7, 119.0, 118.3, 112.6.

***N*-phenyl-4-(trifluoromethyl)aniline[24] (26)**

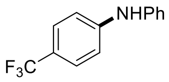

26

According to **GP2** starting from 1-iodo-4-(trifluoromethyl)benzene (0.2 mmol, 1.0 eq.), aniline (0.3 mmol, 1.5 eq.), TMG (0.4 mmol, 2.0 eq.) and bpyNi-PHI (10 mg, 6.0 wt % Nibpy) in MeCN (2 mL) for 12 h, the product **26** was isolated as pale yellow solid after flash chromatography (petroleum ether/ethyl acetate 10/1), 39.4 mg (83% yield). **^{1}H NMR** (400 MHz, $CDCl_3$) δ 7.51 – 7.42 (m, 2H), 7.38 – 7.29 (m, 2H), 7.18 – 7.12 (m, 2H), 7.09 – 7.00 (m, 3H), 5.91 (br, 1H). **^{13}C NMR** (101 MHz, $CDCl_3$) δ 146.8, 141.2, 129.6, 126.7 (q, J = 3.7 Hz), 124.6 (q, J = 270.7 Hz), 123.0, 121.7 (q, J = 32.7 Hz), 120.1, 115.3. **^{19}F NMR** (377 MHz, $CDCl_3$) δ -61.4.

4-(phenylamino)benzonitrile[23] (27)

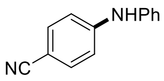

27

According to **GP2** starting from 4-iodobenzonitrile (0.2 mmol, 1.0 eq.), aniline (0.3 mmol, 1.5 eq.), TMG (0.4 mmol, 2.0 eq.) and bpyNi-PHI (10 mg, 6.0 wt % Nibpy) in MeCN (2 mL) for 12 h, the product **27** was isolated as brown solid after flash chromatography (petroleum ether/ethyl acetate 5/1), 33.0 mg (85% yield). **^{1}H NMR** (400 MHz, $CDCl_3$) δ 7.50 – 7.44 (m, 2H), 7.38 – 7.31 (m, 2H), 7.21 – 7.14 (m, 2H), 7.14 – 7.08 (m, 1H), 7.01 – 6.94 (m, 2H), 6.14 (br, 1H). **^{13}C NMR** (101 MHz, $CDCl_3$) δ 148.1, 140.0, 133.8, 129.7, 124.0, 121.3, 120.0, 114.9, 101.5.

Methyl 4-(phenylamino)benzoate[15] (28)

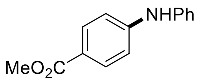

28

According to **GP2** starting from methyl 4-iodobenzoate (0.2 mmol, 1.0 eq.), aniline (0.3 mmol, 1.5 eq.), TMG (0.4 mmol, 2.0 eq.) and bpyNi-PHI (10 mg, 6.0 wt % Nibpy) in MeCN (2 mL) for 12 h, the product **28** was isolated as white solid after flash chromatography (petroleum ether/ethyl acetate 10/1), 36.4 mg (80% yield). **^{1}H NMR** (500 MHz, $CDCl_3$) δ 7.91 (d, J = 9.0 Hz, 2H), 7.33 (t, J = 7.9 Hz, 2H), 7.16 (d, J = 7.9 Hz, 2H), 7.06 (t, J = 7.5 Hz, 1H), 6.98 (d, J = 9.0 Hz, 2H), 6.10 (br, 1H), 3.86 (s, 3H). **^{13}C NMR** (126 MHz, $CDCl_3$) δ 167.1, 148.2, 140.9, 131.5, 129.5, 123.1, 121.1, 120.5, 114.6, 51.8.

1-(4-(phenylamino)phenyl)ethan-1-one[15] (29)

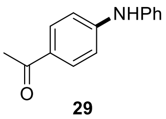

29

According to **GP2** starting from 1-(4-iodophenyl)ethan-1-one (0.2 mmol, 1.0 eq.), aniline (0.3 mmol, 1.5 eq.), TMG (0.4 mmol, 2.0 eq.) and bpyNi-PHI (10 mg, 6.0 wt % Nibpy) in MeCN (2 mL) for 12 h, the product **29** was isolated as white solid after flash chromatography (petroleum ether/ethyl acetate 5/1), 35.1 mg (83% yield). **^{1}H NMR** (500 MHz, $CDCl_3$) δ 7.86 (d, J = 8.5 Hz, 2H), 7.33 (t, J = 7.7 Hz, 2H), 7.18 (d, J = 7.9 Hz, 2H), 7.07 (t, J = 7.4 Hz, 1H), 6.99 (d, J = 8.5 Hz, 2H), 6.29 (br, 1H), 2.52 (s, 3H). **^{13}C NMR** (126 MHz, $CDCl_3$) δ 196.6, 148.5, 140.7, 130.7, 129.6, 129.0, 123.4, 120.7, 114.4, 26.2.

3-bromo-*N*-phenylaniline[25] (30)

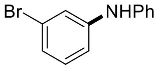

30

According to **GP2** starting from 1-bromo-3-iodobenzene (0.2 mmol, 1.0 eq.), aniline (0.3 mmol, 1.5 eq.), TMG (0.4 mmol, 2.0 eq.) and bpyNi-PHI (10 mg, 6.0 wt % Nibpy) in MeCN (2 mL) for 12 h, the product **30** was isolated as white solid after flash chromatography (petroleum ether/ethyl acetate 20/1), 40.7 mg (82% yield). **^{1}H NMR** (400 MHz, $CDCl_3$) δ 7.33 – 7.27 (m, 2H), 7.19 (t, J = 2.1 Hz, 1H), 7.13 – 7.06 (m, 3H), 7.03 – 6.96 (m, 2H), 6.95 – 6.91 (m, 1H), 5.69 (br, 1H). **^{13}C NMR** (101 MHz, $CDCl_3$) δ 145.0, 141.9, 130.6, 129.5, 123.4, 123.1, 122.2, 119.6, 119.0, 115.6.

2-methoxy-*N*-phenylaniline[23] (31)

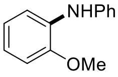

31

According to **GP2** starting from 1-iodo-2-methoxybenzene(0.2 mmol, 1.0 eq.), aniline (0.3 mmol, 1.5 eq.), TMG (0.4 mmol, 2.0 eq.) and bpyNi-PHI (10 mg, 6.0 wt % Nibpy) in MeCN (2 mL) for 12 h, the product **31** was isolated as brown oil after flash chromatography (petroleum ether/ethyl acetate 20/1), 32.3 mg (81% yield). **^{1}H NMR** (400 MHz, $CDCl_3$) δ 7.32 – 7.26 (m, 3H), 7.17 – 7.11 (m, 2H), 6.97 – 6.83 (m, 4H), 6.14 (br, 1H), 3.89 (s, 3H). **^{13}C NMR** (101 MHz, $CDCl_3$) δ 148.3, 142.7, 133.0, 129.3, 121.1, 120.8, 119.9, 118.6, 114.7, 110.5, 55.6.

2-chloro-3-methyl-*N*-phenylpyridin-4-amine (32)

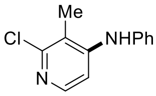

32

According to **GP2** starting from 2-chloro-4-iodo-3-methylpyridine (0.2 mmol, 1.0 eq.), aniline (0.3 mmol, 1.5 eq.), TMG (0.4 mmol, 2.0 eq.) and bpyNi-PHI (10 mg, 6.0 wt % Nibpy) in MeCN (2 mL) for 12 h, the product **32** was isolated as brown solid after flash chromatography (petroleum ether/dichloromethane 1/1), 31.5 mg (72% yield). mp = 165–166 °C. **^{1}H NMR** (400 MHz, $CDCl_3$) δ 7.92 (d, J = 5.7 Hz, 1H), 7.43 – 7.36 (m, 2H), 7.22 – 7.15 (m, 3H), 6.85 (d, J = 5.7 Hz, 1H), 5.90 (br, 1H), 2.34 (s, 3H). **^{13}C NMR** (101 MHz, $CDCl_3$) δ 152.0, 151.4, 146.9, 139.4, 129.7, 125.0, 122.9, 115.9, 106.8, 13.5. **HRMS (ESI)** m/z calcd. For $C_{12}H_{12}ClN_2$ $[M+H]^+$ 219.0684, found 219.0686.

N-phenylpyridin-3-amine[26] (33)

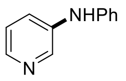

33

According to **GP2** starting from 3-iodopyridine (0.2 mmol, 1.0 eq.), aniline (0.3 mmol, 1.5 eq.), TMG (0.4 mmol, 2.0 eq.) and bpyNi-PHI (10 mg, 6.0 wt % Nibpy) in MeCN (2 mL) for 12 h, the product **33** was isolated as yellow solid after flash chromatography (petroleum ether/ethyl acetate 5/1), 23.8 mg (70% yield). **^{1}H NMR** (500 MHz, $CDCl_3$) δ 8.40 – 8.34 (m, 1H), 8.15 (d, *J* = 4.6 Hz, 1H), 7.41 (d, *J* = 6.9 Hz, 1H), 7.29 (t, *J* = 7.7 Hz, 2H), 7.16 (dd, *J* = 8.3, 4.7 Hz, 1H), 7.08 (d, *J* = 7.9 Hz, 2H), 6.99 (t, *J* = 7.4 Hz, 1H), 5.94 (br, 1H). **^{13}C NMR** (126 MHz, $CDCl_3$) δ 142.0, 141.9, 140.1, 139.9, 129.6, 123.8, 123.4, 122.1, 118.4.

N-phenyl-1*H*-indol-5-amine[23] (34)

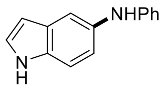

34

According to **GP2** starting from 5-iodo-1*H*-indole (0.2 mmol, 1.0 eq.), aniline (0.3 mmol, 1.5 eq.), TMG (0.4 mmol, 2.0 eq.) and bpyNi-PHI (10 mg, 6.0 wt % Nibpy) in MeCN (2 mL) for 12 h, the product **34** was isolated as brown solid after flash chromatography (petroleum ether/ethyl acetate 5/1), 37.5 mg (90% yield). **^{1}H NMR** (400 MHz, $CDCl_3$) δ 8.08 (br, 1H), 7.44 – 7.40 (m, 1H), 7.32 (d, *J* = 8.6 Hz, 1H), 7.24 – 7.16 (m, 3H), 7.03 (dd, *J* = 8.6, 2.1 Hz, 1H), 6.93 (d, *J* = 7.4 Hz, 2H), 6.80 (t, *J* = 7.3 Hz, 1H), 6.51 – 6.45 (m, 1H), 5.61 (br, 1H). **^{13}C NMR** (101 MHz, $CDCl_3$) δ 146.3, 135.2, 132.7, 129.3, 128.6, 125.0, 119.0, 118.3, 115.3, 113.2, 111.6, 102.4.

4-methyl-*N*-phenylbenzenesulfonamide[27] (35)

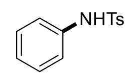

35

According to **GP2** starting from iodobenzene (0.2 mmol, 1.0 eq.), 4-methylbenzenesulfonamide (0.3 mmol, 1.5 eq.), TMG (0.4 mmol, 2.0 eq.) and bpyNi-PHI (10 mg, 6.0 wt % Nibpy) in MeCN (2 mL) for 16 h, the product **35** was isolated as white solid after flash chromatography (petroleum ether/ethyl acetate 5/1), 46.0 mg (93% yield). **^{1}H NMR** (400 MHz, $CDCl_3$) δ 7.69 (d, *J* = 8.1 Hz, 2H), 7.25 – 7.17 (m, 5H), 7.12 – 7.03 (m, 3H), 2.36 (s, 3H). **^{13}C NMR** (126 MHz, $CDCl_3$) δ 143.9, 136.7, 136.1, 129.7, 129.3, 127.3, 125.2, 121.5, 21.6.

4-methyl-*N*-(p-tolyl)benzenesulfonamide[28] (36)

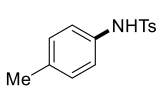

36

According to **GP2** starting from 1-iodo-4-methylbenzene (0.2 mmol, 1.0 eq.), 4-methylbenzenesulfonamide (0.3 mmol, 1.5 eq.), TMG (0.4 mmol, 2.0 eq.) and bpyNi-PHI (10 mg, 6.0 wt % Nibpy) in MeCN (2 mL) for 16 h, the product **36** was isolated as pale yellow solid after flash chromatography (petroleum ether/ethyl acetate 5/1), 49.7 mg (95% yield). **^{1}H NMR** (400 MHz, $CDCl_3$) δ 7.64 (d, *J* = 8.4 Hz, 2H), 7.21 (d, *J* = 8.1 Hz, 2H), 7.02 (d, *J* = 8.3 Hz, 2H), 6.98 – 6.93 (m, 2H), 6.77 (br, 1H), 2.37 (s, 3H), 2.26 (s, 3H). **^{13}C NMR** (101 MHz, $CDCl_3$) δ 143.7, 136.1, 135.4, 133.8, 129.9, 129.6, 127.3, 122.3, 21.5, 20.9.

***N*-(4-bromophenyl)-4-methylbenzenesulfonamide[28] (37)**

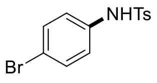

37

According to **GP2** starting from 1-bromo-4-iodobenzene (0.2 mmol, 1.0 eq.), 4-methylbenzenesulfonamide (0.3 mmol, 1.5 eq.), TMG (0.4 mmol, 2.0 eq.) and bpyNi-PHI (10 mg, 6.0 wt % Nibpy) in MeCN (2 mL) for 16 h, the product **37** was isolated as white solid after flash chromatography (petroleum ether/ethyl acetate 5/1), 58.7 mg (90% yield). **^{1}H NMR** (400 MHz, $CDCl_3$) δ 7.67 (d, *J* = 8.3 Hz, 2H), 7.36 – 7.31 (m, 2H), 7.24 (d, *J* = 8.1 Hz, 2H), 7.19 (s, 1H), 7.02 – 6.93 (m, 2H), 2.38 (s, 3H). **^{13}C NMR** (101 MHz, $CDCl_3$) δ 144.2, 135.7, 135.7, 132.4, 129.8, 127.3, 123.0, 118.5, 21.6.

4-methyl-N-(4-(trifluoromethyl)phenyl)benzenesulfonamide[28] (38)

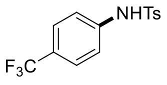

38

According to **GP2** starting from 1-iodo-4-(trifluoromethyl)benzene (0.2 mmol, 1.0 eq.), 4-methylbenzenesulfonamide(0.3 mmol, 1.5 eq.), TMG (0.4 mmol, 2.0 eq.) and bpyNi-PHI (10 mg, 6.0 wt % Nibpy) in MeCN (2 mL) for 16 h, the product **38** was isolated as white solid after flash chromatography (dichloromethane/ethyl acetate 20/1), 56.8 mg (90% yield). **^{1}H NMR** (400 MHz, $CDCl_3$) δ 7.78 – 7.71 (m, 2H), 7.53 – 7.44 (m, 3H), 7.28 (s, 1H), 7.26 (s, 1H), 7.19 (d, *J* = 8.5 Hz, 2H), 2.39 (s, 3H). **^{13}C NMR** (101 MHz, $CDCl_3$) δ 144.6, 139.9, 135.7, 130.0, 127.3, 126.7 (q, *J* = 3.8 Hz), 126.6 (q, *J* = 33.0 Hz). 123.9 (q, *J* = 271.6 Hz), 119.6, 21.6. **^{19}F NMR** (377 MHz, $CDCl_3$) δ -62.2.

4-methyl-*N*-(thiophen-3-yl)benzenesulfonamide[28] (39)

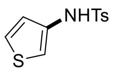

39

According to **GP2** starting from 3-iodothiophene(0.2 mmol, 1.0 eq.), 4-methylbenzenesulfonamide (0.3 mmol, 1.5 eq.), TMG (0.4 mmol, 2.0 eq.) and bpyNi-PHI (10 mg, 6.0 wt % Nibpy) in MeCN (2 mL) for 16 h, the product **39** was isolated as brown solid after flash chromatography (dichloromethane/ethyl acetate 50/1), 43.1 mg (85% yield). **^{1}H NMR** (400 MHz, $CDCl_3$) δ 7.68 – 7.62 (m, 2H), 7.24 (d, *J* = 8.1 Hz, 2H), 7.18 (dd, *J* = 5.1, 3.2 Hz, 1H), 6.87 (dd, *J* = 3.2, 1.5 Hz, 1H), 6.83 (dd, *J* = 5.1, 1.4 Hz, 1H), 6.78 (br, 1H), 2.39 (s, 3H). **^{13}C NMR** (101 MHz, $CDCl_3$) δ 144.0, 135.9, 134.2, 129.6, 127.3, 125.6, 123.4, 114.2, 21.6.

4-methoxy-*N*-phenylaniline[15] (40)

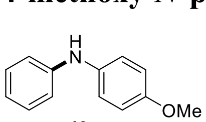

40

According to **GP2** starting from iodobenzene (0.2 mmol, 1.0 eq.), 4-methoxyaniline (0.3 mmol, 1.5 eq.), TMG (0.4 mmol, 2.0 eq.) and bpyNi-PHI (10 mg, 6.0 wt % Nibpy) in MeCN (2 mL) for 12 h, the product **40** was isolated as white solid after flash chromatography (petroleum ether/ ethyl acetate 30/1), 34.3 mg (86% yield). **^{1}H NMR** (500 MHz, $CDCl_3$) δ 7.25 – 7.17 (m, 2H), 7.09 – 7.04 (m, 2H), 6.90 (d, *J* = 7.7 Hz, 2H), 6.87 – 6.80 (m, 3H), 5.48 (br, 1H), 3.79 (s, 3H). **^{13}C NMR** (126 MHz, $CDCl_3$) δ 155.3, 145.2, 135.8, 129.4, 122.3, 119.6, 115.7, 114.7, 55.6.

4-chloro-*N*-phenylaniline[25] (41)

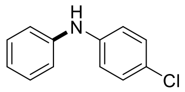

41

According to **GP2** starting from iodobenzene (0.2 mmol, 1.0 eq.), 4-chloroaniline (0.3 mmol, 1.5 eq.), TMG (0.4 mmol, 2.0 eq.) and bpyNi-PHI (10 mg, 6.0 wt % Nibpy) in MeCN (2 mL) for 12 h, the product **41** was isolated as pale yellow solid after flash chromatography (petroleum ether/ethyl acetate 20/1), 35.0 mg (86% yield). **^{1}H NMR** (400 MHz, $CDCl_3$) δ 7.32 – 7.23 (m, 2H), 7.23 – 7.18 (m, 2H), 7.07 – 7.02 (m, 2H), 7.01 – 6.92 (m, 3H), 5.66 (br, 1H). **^{13}C NMR** (101 MHz, $CDCl_3$) δ 142.7, 141.9, 129.5, 129.3, 125.5, 121.5, 118.8, 118.1.

Methyl 4-(phenylamino)benzoate[15] (42)

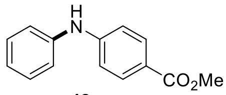

42

According to **GP2** starting from iodobenzene (0.2 mmol, 1.0 eq.), methyl 4-aminobenzoate (0.3 mmol, 1.5 eq.), TMG (0.4 mmol, 2.0 eq.) and bpyNi-PHI (10 mg, 6.0 wt % Nibpy) in MeCN (2 mL) for 16 h, the product **42** was isolated as white solid after flash chromatography (petroleum ether/ethyl acetate 10/1), 41.4 mg (91% yield). **^{1}H NMR** (500 MHz, $CDCl_3$) δ 7.91 (d, J = 9.0 Hz, 2H), 7.33 (t, J = 7.9 Hz, 2H), 7.16 (d, J = 7.9 Hz, 2H), 7.06 (t, J = 7.5 Hz, 1H), 6.98 (d, J = 9.0 Hz, 2H), 6.10 (br, 1H), 3.86 (s, 3H). **^{13}C NMR** (126 MHz, $CDCl_3$) δ 167.1, 148.2, 140.9, 131.5, 129.5, 123.1, 121.1, 120.5, 114.6, 51.8.

1-(3-(phenylamino)phenyl)ethan-1-one[29] (43)

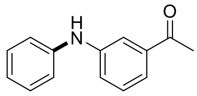

43

According to **GP2** starting from iodobenzene (0.2 mmol, 1.0 eq.), 1-(3-aminophenyl)ethan-1-one (0.3 mmol, 1.5 eq.), TMG (0.4 mmol, 2.0 eq.) and bpyNi-PHI (10 mg, 6.0 wt % Nibpy) in MeCN (2 mL) for 16 h, the product **43** was isolated as yellow solid after flash chromatography (petroleum ether/ethyl acetate 5/1), 33.8 mg (80% yield). **^{1}H NMR** (400 MHz, $CDCl_3$) δ 7.64 – 7.59 (m, 1H), 7.49 – 7.44 (m, 1H), 7.35 – 7.27 (m, 3H), 7.26 – 7.23 (m, 1H), 7.12 – 7.06 (m, 2H), 7.00 – 6.94 (m, 1H), 5.88 (br, 1H), 2.57 (s, 3H). **^{13}C NMR** (101 MHz, $CDCl_3$) δ 198.3, 143.9, 142.3, 138.4, 129.5, 121.9, 121.6, 120.8, 118.5, 116.5, 26.8.

Phenyl(2-(phenylamino)phenyl)methanone[30] (44)

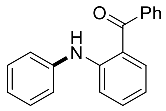

44

According to **GP2** starting from iodobenzene (0.2 mmol, 1.0 eq.), (2-aminophenyl)(phenyl)methanone (0.3 mmol, 1.5 eq.), TMG (0.4 mmol, 2.0 eq.) and bpyNi-PHI (10 mg, 6.0 wt % Nibpy) in MeCN (2 mL) for 16 h, the product **44** was isolated as yellow oil after flash chromatography (petroleum ether/ethyl acetate 10/1), 50.8 mg (93% yield). **^{1}H NMR** (400 MHz, $CDCl_3$) δ 10.12 (br, 1H), 7.73 – 7.66 (m, 2H), 7.58 – 7.51 (m, 2H), 7.50 – 7.44 (m, 2H), 7.39 – 7.31 (m, 4H), 7.31 – 7.27 (m, 2H), 7.13 – 7.03 (m, 1H), 6.71 – 6.66 (m, 1H). **^{13}C NMR** (101 MHz, $CDCl_3$) δ 199.2, 148.1, 140.6, 139.9, 135.0, 134.3, 131.4, 129.4, 129.4, 128.2, 123.6, 122.2, 119.8, 116.6, 114.7.

2-bromo-*N*-phenylaniline[31] (45)

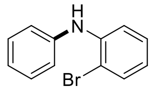

45

According to **GP2** starting from iodobenzene (0.2 mmol, 1.0 eq.), 2-bromoaniline (0.3 mmol, 1.5 eq.), TMG (0.4 mmol, 2.0 eq.) and bpyNi-PHI (10 mg, 6.0 wt % Nibpy) in MeCN (2 mL) for 16 h, the product **45** was isolated as yellowish oil after flash chromatography (petroleum ether/ethyl acetate 20/1), 42.2 mg (85% yield). **^{1}H NMR** (400 MHz, $CDCl_3$) δ 7.52 (dd, *J* = 8.0, 1.5 Hz, 1H), 7.35 – 7.29 (m, 2H), 7.26 – 7.22 (m, 1H), 7.19 – 7.11 (m, 3H), 7.06 – 7.00 (m, 1H), 6.80 – 6.64 (m, 1H), 6.08 (br, 1H). **^{13}C NMR** (101 MHz, $CDCl_3$) δ 141.6, 141.5, 133.0, 129.5, 128.1, 122.7, 120.9, 120.3, 115.8, 112.2.

(2-(phenylamino)phenyl)methanol[32] (46)

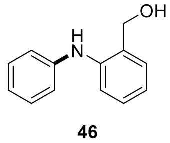

46

According to **GP2** starting from iodobenzene (0.2 mmol, 1.0 eq.), (2-aminophenyl)methanol (0.3 mmol, 1.5 eq.), TMG (0.4 mmol, 2.0 eq.) and bpyNi-PHI (10 mg, 6.0 wt % Nibpy) in MeCN (2 mL) for 12 h, the product **46** was isolated as brown solid after flash chromatography (petroleum ether/ethyl acetate 5/1), 33.1 mg (83% yield). **^{1}H NMR** (400 MHz, $CDCl_3$) δ 7.36 (d, *J* = 8.1, 1H), 7.30 – 7.25 (m, 2H), 7.24 – 7.18 (m, 2H), 7.09 – 7.04 (m, 2H), 6.97 – 6.84 (m, 2H), 4.71 (s, 2H). **^{13}C NMR** (101 MHz, $CDCl_3$) δ 143.2, 143.0, 129.6, 129.4, 129.2, 128.5, 121.0, 120.5, 118.2, 117.1, 64.7.

***N*-phenylnaphthalen-1-amine[15] (47)**

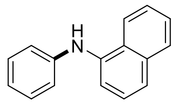

47

According to **GP2** starting from iodobenzene (0.2 mmol, 1.0 eq.), naphthalen-1-amine (0.3 mmol, 1.5 eq.), TMG (0.4 mmol, 2.0 eq.) and bpyNi-PHI (10 mg, 6.0 wt % Nibpy) in MeCN (2 mL) for 12 h, the product **47** was isolated as brown solid after flash chromatography (petroleum ether/ethyl acetate 20/1), 37.3 mg (85% yield). **^{1}H NMR** (400 MHz, $CDCl_3$) δ 8.05 – 8.00 (m, 1H), 7.91 – 7.83 (m, 1H), 7.61 – 7.54 (m, 1H), 7.53 – 7.44 (m, 2H), 7.43 – 7.34 (m, 2H), 7.29 – 7.23 (m, 3H), 7.03 – 6.96 (m, 2H), 6.94 – 6.88 (m, 1H). **^{13}C NMR** (101 MHz, $CDCl_3$) δ 144.8, 138.8, 134.7, 129.4, 128.6, 127.7, 126.1, 126.0, 125.7, 123.0, 121.8, 120.5, 117.4, 115.9.

***N*-phenylpyridin-2-amine[26] (48)**

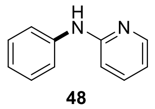

48

According to **GP2** starting from iodobenzene (0.2 mmol, 1.0 eq.), pyridin-2-amine (0.3 mmol, 1.5 eq.), TMG (0.4 mmol, 2.0 eq.) and bpyNi-PHI (10 mg, 6.0 wt % Nibpy) in MeCN (2 mL) for 12 h, the product **48** was isolated as pale yellow solid after flash chromatography (petroleum ether/ethyl acetate 5/1), 23.8 mg (70% yield). **^{1}H NMR** (400 MHz, $CDCl_3$) δ 8.24 – 8.16 (m, 1H), 7.52 – 7.45 (m, 1H), 7.37 – 7.29 (m, 4H), 7.11 – 7.01 (m, 1H), 6.88 (d, *J* = 8.5 Hz, 1H), 6.80 – 6.69 (m, 2H). **^{13}C NMR** (101 MHz, $CDCl_3$) δ 156.0, 148.3, 140.4, 137.8, 129.3, 122.9, 120.4, 115.0, 108.3.

Methyl 4-(isobutylamino)benzoate (49)

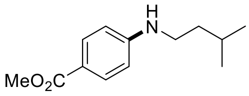

49

According to **GP2** starting from methyl 4-iodobenzoate (0.2 mmol, 1.0 eq.), 3-methylbutan-1-amine (0.3 mmol, 1.5 eq.), TMG (0.4 mmol, 2.0 eq.) and bpyNi-PHI (10 mg, 6.0 wt % Nibpy) in MeCN (2 mL) for 36 h, the product **49** was isolated as white solid after flash chromatography (petroleum ether/ethyl acetate 5/1), 34.0 mg (82% yield), mp = 57–58 °C. **^{1}H NMR** (500 MHz, $CDCl_3$) δ 7.85 (d, *J* = 8.4 Hz, 2H), 6.53 (d, *J* = 8.9 Hz, 2H), 4.11 (s, 1H), 3.84 (s, 3H), 3.15 (t, *J* = 7.3 Hz, 2H), 1.70 (m, 1H), 1.51 (q, *J* = 7.2 Hz, 2H), 0.95 (d, *J* = 6.8 Hz, 6H). **^{13}C NMR** (126 MHz, $CDCl_3$) δ 167.4, 152.3, 131.6, 117.9, 111.3, 51.5, 41.5, 38.2, 26.0, 22.6. **HRMS (ESI)** m/z calcd. For $C_{13}H_{20}NO_2$ $[M+H]^+$ 222.1489, found 222.1490.

Methyl 4-(cyclohexylamino)benzoate[33] (50)

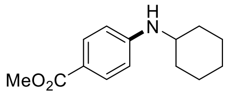

50

According to **GP2** starting from methyl 4-iodobenzoate (0.2 mmol, 1.0 eq.), cyclohexanamine (0.3 mmol, 1.5 eq.), TMG (0.4 mmol, 2.0 eq.) and bpyNi-PHI (10 mg, 6.0 wt % Nibpy) in MeCN (2 mL) for 36 h, the product **50** was isolated as white solid after flash chromatography (petroleum ether/ethyl acetate 20/1), 39.7 mg (85% yield). **^{1}H NMR** (500 MHz, $CDCl_3$) δ 7.83 (d, *J* = 8.8 Hz, 2H), 6.51 (d, *J* = 8.7 Hz, 2H), 4.07 (s, 1H), 3.84 (s, 3H), 3.45 – 3.23 (m, 1H), 2.10 – 2.00 (m, 2H), 1.82 – 1.72 (m, 2H), 1.70 – 1.59 (m, 2H), 1.44 – 1.31 (m, 1H), 1.26 – 1.12 (m, 3H). **^{13}C NMR** (126 MHz, $CDCl_3$) δ 168.1, 151.9, 132.3, 118.2, 112.3, 52.1, 51.9, 33.8, 26.4, 25.6.

Methyl 4-(benzylamino)benzoate[34] (51)

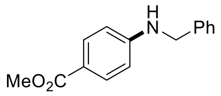

51

According to **GP2** starting from methyl 4-iodobenzoate(0.2 mmol, 1.0 eq.), phenylmethanamine (0.3 mmol, 1.5 eq.), TMG (0.4 mmol, 2.0 eq.) and bpyNi-PHI (10 mg, 6.0 wt % Nibpy) in MeCN (2 mL) for 36 h, the product **51** was isolated as pale yellow solid after flash chromatography (petroleum ether/ethyl acetate 10/1), 40.0 mg (83% yield). **^{1}H NMR** (500 MHz, $CDCl_3$) δ 7.85 (d, *J* = 8.9 Hz, 2H), 7.38 – 7.31 (m, 4H), 7.31 – 7.26 (m, 1H), 6.58 (d, *J* = 8.4 Hz, 2H), 4.52 (br, 1H), 4.40 – 4.34 (m, 2H), 3.83 (s, 3H). **^{13}C NMR** (126 MHz, $CDCl_3$) δ 167.3, 151.8, 138.4, 131.6, 128.8, 127.6, 127.5, 118.7, 111.7, 51.6, 47.7.

Methyl 4-(benzyl(methyl)amino)benzoate[33] (52)

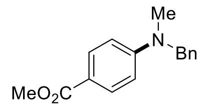

52

According to **GP2** starting from methyl 4-iodobenzoate (0.2 mmol, 1.0 eq.), *N*-methyl-1-phenylmethanamine (0.3 mmol, 1.5 eq.), TMG (0.4 mmol, 2.0 eq.) and bpyNi-PHI (10 mg, 6.0 wt % Nibpy) in MeCN (2 mL) for 48 h, the product **52** was isolated as white solid after flash chromatography (petroleum ether/ethyl acetate 20/1), 16.3 mg (32% yield). **^{1}H NMR** (500 MHz, $CDCl_3$) δ 7.88 (d, *J* = 8.9 Hz, 2H), 7.31 (t, *J* = 7.7 Hz, 2H), 7.25 – 7.22 (m, 1H), 7.17 (d, *J* = 7.5 Hz, 2H), 6.68 (d, *J* = 9.0 Hz, 2H), 4.61 (s, 2H), 3.84 (s, 3H), 3.10 (s, 3H). **^{13}C NMR** (126 MHz, $CDCl_3$) δ 167.4, 152.8, 137.8, 131.4, 128.8, 127.2, 126.5, 117.4, 110.9, 56.0, 51.5, 38.7.

Methyl 4-morpholinobenzoate[35] (53)

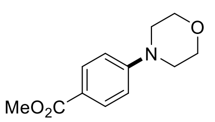

53

According to **GP2** starting from methyl 4-iodobenzoate (0.2 mmol, 1.0 eq.), morpholine (0.3 mmol, 1.5 eq.), TMG (0.4 mmol, 2.0 eq.) and bpyNi-PHI (10 mg, 6.0 wt % Nibpy) in MeCN (2 mL) for 48 h, the product **53** was isolated as white solid after flash chromatography (petroleum ether/ethyl acetate 20/1), 13.7 mg (31% yield). **^{1}H NMR** (500 MHz, $CDCl_3$) δ 7.94 (d, J = 8.7 Hz, 2H), 6.86 (d, J = 8.7 Hz, 2H), 3.90 – 3.80 (m, 7H), 3.33 – 3.25 (m, 4H). **^{13}C NMR** (126 MHz, $CDCl_3$) δ 167.1, 154.2, 131.2, 120.3, 113.5, 66.6, 51.7, 47.7.

***N*-phenyl-4-(trifluoromethyl)benzenesulfonamide[27] (54)**

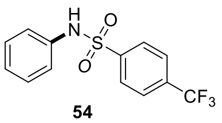

54

According to **GP2** starting from iodobenzene (0.2 mmol, 1.0 eq.), 4-(trifluoromethyl)benzenesulfonamide (0.3 mmol, 1.5 eq.), TMG (0.4 mmol, 2.0 eq.) and bpyNi-PHI (10 mg, 6.0 wt % Nibpy) in MeCN (2 mL) for 16 h, the product **54** was isolated as yellow solid after flash chromatography (petroleum ether/ethyl acetate 5/1), 52.4 mg (87% yield). **^{1}H NMR** (400 MHz, $CDCl_3$) δ 7.91 (d, J = 8.1 Hz, 2H), 7.70 (d, J = 8.1 Hz, 2H), 7.28 – 7.22 (m, 3H), 7.15 (t, J = 7.4 Hz, 1H), 7.10 (d, J = 7.9 Hz, 2H). **^{13}C NMR** (101 MHz, $CDCl_3$) δ 142.5, 135.7, 134.7 (q, J = 33.1 Hz), 129.6, 127.8, 126.3 (q, J = 3.7 Hz), 126.1, 123.1 (q, J = 273.0 Hz), 122.0. **^{19}F NMR** (377 MHz, $CDCl_3$) δ -63.2.

Methyl 4-(cyclopropanesulfonamido)benzoate (55)

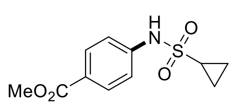

55

According to **GP2** starting from methyl 4-iodobenzoate (0.2 mmol, 1.0 eq.), cyclopropanesulfonamide (0.3 mmol, 1.5 eq.), TMG (0.4 mmol, 2.0 eq.) and bpyNi-PHI (10 mg, 6.0 wt % Nibpy) in MeCN (2 mL) for 16 h, the product **55** was isolated as white solid after flash chromatography (petroleum ether/ethyl acetate 5/1), 40.8 mg (80% yield), mp = 117–118 °C. **^{1}H NMR** (400 MHz, $CDCl_3$) δ 8.01 (d, J = 8.7 Hz, 2H), 7.39 – 7.17 (m, 3H), 3.92 (s, 3H), 2.63 – 2.50 (m, 1H), 1.40 – 1.19 (m, 2H), 1.11 – 0.76 (m, 2H). **^{13}C NMR** (101 MHz, $CDCl_3$) δ 166.5, 141.5, 131.3, 126.2, 119.2, 52.2, 30.5, 5.8. **HRMS (ESI)** m/z calcd. For $C_{11}H_{12}NO_4S$ $[M-H]^-$ 254.0487, found 254.0492.

Methyl 4-((methyl(oxo)(phenyl)-λ^6-sulfanylidene)amino)benzoate[36] (56)

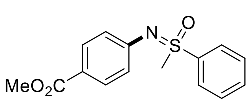

56

According to **GP2** starting from methyl 4-iodobenzoate (0.2 mmol, 1.0 eq.), imino(methyl)(phenyl)- λ^6-sulfanone (0.3 mmol, 1.5 eq.), TMG (0.4 mmol, 2.0 eq.) and bpyNi-PHI (10 mg, 6.0 wt % Nibpy) in MeCN (2 mL) for 16 h, the product **56** was isolated as white solid after flash chromatography (petroleum ether/ethyl acetate 2/1), 44.6 mg (77% yield). **^{1}H NMR** (400 MHz, $CDCl_3$) δ 8.05 – 7.92 (m, 2H), 7.84 – 7.77 (m, 2H), 7.67 – 7.57 (m, 1H), 7.56 – 7.51 (m, 2H), 7.09 – 6.96 (m, 2H), 3.82 (s, 3H), 3.28 (s, 3H). **^{13}C NMR** (101 MHz, $CDCl_3$) δ 167.1, 150.2, 138.8, 133.6, 130.8, 129.7, 128.5, 122.9, 122.5, 51.7, 46.4.

Methyl 4-((diphenylmethylene)amino)benzoate[37] (57)

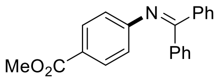

57

According to **GP2** starting from methyl 4-iodobenzoate (0.2 mmol, 1.0 eq.), diphenylmethanimine (0.3 mmol, 1.5 eq.), TMG (0.4 mmol, 2.0 eq.) and bpyNi-PHI (10 mg, 6.0 wt % Nibpy) in MeCN (2 mL) for 16 h, the product **57** was isolated as pale yellow solid after flash chromatography (petroleum ether/ethyl acetate/triethylamine 100/10/1), 51.7 mg (82% yield). **^{1}H NMR** (400 MHz, $CDCl_3$) δ 7.87 – 7.81 (m, 2H), 7.75 (d, J = 7.2 Hz, 2H), 7.49 (t, J = 7.3 Hz, 1H), 7.41 (t, J = 7.5 Hz, 2H), 7.29 – 7.20 (m, 3H), 7.10 (d, J = 5.7 Hz, 2H), 6.78 – 6.71 (m, 2H), 3.84 (s, 3H). **^{13}C NMR** (101 MHz, $CDCl_3$) δ 168.9, 167.0, 155.8, 139.0, 135.6, 131.2, 130.4, 129.5, 129.4, 129.0, 128.3, 128.1, 124.7, 120.6, 51.9.

Methyl 4-benzamidobenzoate[38] (58)

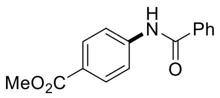

58

According to **GP2** starting from methyl 4-iodobenzoate (0.2 mmol, 1.0 eq.), benzamide (0.3 mmol, 1.5 eq.), TMG (0.4 mmol, 2.0 eq.) and bpyNi-PHI (10 mg, 6.0 wt % Nibpy) in MeCN (2 mL) for 48 h, the product **58** was isolated as white solid after flash chromatography (petroleum ether/ethyl acetate 5/1), 31.1 mg (61% yield). **^{1}H NMR** (400 MHz, $CDCl_3$) δ 8.10 (br, 1H), 8.05 (d, J = 8.7 Hz, 2H), 7.87 (d, J = 7.0 Hz, 2H), 7.75 (d, J = 8.8 Hz, 2H), 7.57 (t, J = 7.4 Hz, 1H), 7.49 (t, J = 7.5 Hz, 2H), 3.91 (s, 3H). **^{13}C NMR** (101 MHz, $CDCl_3$) δ 166.6, 165.9, 142.2, 134.5, 132.2, 130.9, 128.9, 127.1, 125.9, 119.2, 52.1.

Methyl 4-((*tert*-butoxycarbonyl)amino)benzoate[39] (59)

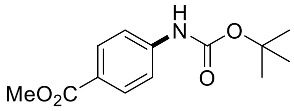

59

According to **GP2** starting from methyl 4-iodobenzoate (0.2 mmol, 1.0 eq.), *tert*-butyl carbamate (0.3 mmol, 1.5 eq.), TMG (0.4 mmol, 2.0 eq.) and bpyNi-PHI (10 mg, 6.0 wt % Nibpy) in MeCN (2 mL) for 24 h, the product **59** was isolated as white solid after flash chromatography (petroleum ether/ethyl acetate 10/1), 33.2 mg (66% yield). **^{1}H NMR** (400 MHz, $CDCl_3$) δ 7.97 (d, J = 8.8 Hz, 2H), 7.43 (d, J = 8.8 Hz, 2H), 6.70 (br, 1H), 3.89 (s, 3H), 1.53 (s, 9H). **^{13}C NMR** (101 MHz, $CDCl_3$) δ 166.7, 152.2, 142.7, 130.9, 124.4, 117.4, 81.2, 51.9, 28.3.

Methyl 4-(((benzyloxy)carbonyl)amino)benzoate[39] (60)

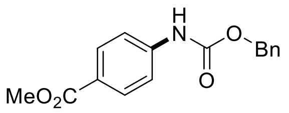

60

According to **GP2** starting from methyl 4-iodobenzoate (0.2 mmol, 1.0 eq.), benzyl carbamate (0.3 mmol, 1.5 eq.), TMG (0.4 mmol, 2.0 eq.) and bpyNi-PHI (10 mg, 6.0 wt % Nibpy) in MeCN (2 mL) for 24 h, the product **60** was isolated as pale yellow solid after flash chromatography (petroleum ether/ethyl acetate 5/1), 34.2 mg (60% yield). **^{1}H NMR** (400 MHz, $CDCl_3$) δ 8.07 – 7.85 (m, 2H), 7.49 – 7.44 (m, 2H), 7.43 – 7.34 (m, 5H), 6.88 (br, 1H), 5.22 (s, 2H), 3.89 (s, 3H). **^{13}C NMR** (101 MHz, $CDCl_3$) δ 166.6, 152.8, 142.0, 135.7, 131.0, 128.7, 128.6, 128.4, 125.0, 117.6, 67.4, 52.0.

1-(4-(hexyloxy)phenyl)ethan-1-one[16] (61)

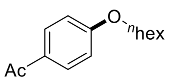

61

According to **GP3** starting from 1-(4-iodophenyl)ethan-1-one (0.2 mmol, 1.0 eq.), hexan-1-ol (1 mmol, 5.0 eq.), TMG (0.4 mmol, 2.0 eq.) and bpyNi-PHI (10 mg, 6.0 wt % Nibpy) in MeCN (2 mL) for 12 h, the product **61** was isolated as colorless oil after flash chromatography (petroleum ether/ethyl acetate 10/1), 40.5 mg (92% yield). **^{1}H NMR** (400 MHz, $CDCl_3$) δ 8.02 – 7.84 (m, 2H), 7.01 – 6.86 (m, 2H), 4.02 (t, *J* = 6.6 Hz, 2H), 2.55 (s, 3H), 1.99 – 1.70 (m, 2H), 1.51 – 1.41 (m, 2H), 1.39 – 1.31 (m, 4H), 0.95 – 0.86 (m, 3H). **^{13}C NMR** (101 MHz, $CDCl_3$) δ 196.8, 163.1, 130.6, 130.1, 114.1, 68.3, 31.5, 29.1, 26.3, 25.7, 22.6, 14.0.

4-(hexyloxy)benzonitrile[16] (62)

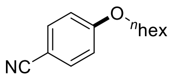

62

According to **GP3** starting from 4-iodobenzonitrile (0.2 mmol, 1.0 eq.), hexan-1-ol (1 mmol, 5.0 eq.), TMG (0.4 mmol, 2.0 eq.) and bpyNi-PHI (10 mg, 6.0 wt % Nibpy) in MeCN (2 mL) for 12 h, the product **62** was isolated as pale yellow solid after flash chromatography (petroleum ether/ethyl acetate 10/1), 38.6 mg (95% yield). **^{1}H NMR** (400 MHz, $CDCl_3$) δ 7.60 – 7.54 (m, 2H), 6.96 – 6.90 (m, 2H), 3.99 (t, *J* = 6.5 Hz, 2H), 1.84 – 1.75 (m, 2H), 1.50 – 1.42 (m, 2H), 1.38 – 1.29 (m, 4H), 0.95 – 0.88 (m, 3H). **^{13}C NMR** (101 MHz, $CDCl_3$) δ 162.5, 134.0, 119.4, 115.2, 103.6, 68.4, 31.5, 28.9, 25.6, 22.6, 14.0.

1-(hexyloxy)-4-(trifluoromethyl)benzene[16] (63)

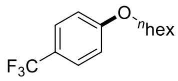

63

According to **GP3** starting from 1-iodo-4-(trifluoromethyl) benzene (0.2 mmol, 1.0 eq.), hexan-1-ol (1 mmol, 5.0 eq.), TMG (0.4 mmol, 2.0 eq.) and bpyNi-PHI (10 mg, 6.0 wt % Nibpy) in MeCN (2 mL) for 24 h, the product **63** was isolated as colorless oil after flash chromatography (petroleum ether), 36.9 mg (75% yield). **^{1}H NMR** (400 MHz, $CDCl_3$) δ 7.53 (d, *J* = 8.7 Hz, 2H), 6.94 (d, *J* = 8.6 Hz, 2H), 3.99 (t, *J* = 6.6 Hz, 2H), 1.84 – 1.75 (m, 2H), 1.52 – 1.40 (m, 2H), 1.40 – 1.29 (m, 4H), 0.97 – 0.85 (m, 3H). **^{13}C NMR** (101 MHz, $CDCl_3$) δ 160.6, δ 127.8 (q, *J* = 3.8 Hz), δ 125.5 (q, *J* = 271.0 Hz), 123.6 (q, *J* = 32.7 Hz), 113.4, 67.2, 30.5, 28.0, 24.6, 21.6, 13.0. **^{19}F NMR** (377 MHz, $CDCl_3$) δ -61.4.

1-bromo-4-(hexyloxy)benzene[16] (64)

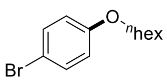

64

According to **GP3** starting from 1-bromo-4-iodobenzene (0.2 mmol, 1.0 eq.), hexan-1-ol (1 mmol, 5.0 eq.), TMG (0.4 mmol, 2.0 eq.) and bpyNi-PHI (10 mg, 6.0 wt % Nibpy) in MeCN (2 mL) for 48 h, the product **64** was isolated as colorless oil after flash chromatography (petroleum ether), 26.7 mg (52% yield, 13/1 ratio of bromo-substituted product **64** vs iodo-substituted product). **^{1}H NMR** (400 MHz, $CDCl_3$) δ 7.35 (d, *J* = 9.0 Hz, 2H), 6.77 (d, *J* = 9.0 Hz, 2H), 3.91 (t, *J* = 6.6 Hz, 2H), 1.81 – 1.70 (m, 2H), 1.49 – 1.39 (m, 2H), 1.38 – 1.28 (m, 4H), 0.93 – 0.88 (m, 3H). **^{13}C NMR** (101 MHz, $CDCl_3$) δ 158.3, 132.2, 116.3, 112.5, 68.3, 31.6, 29.2, 25.7, 22.6, 14.1.

1-(hexyloxy)-4-methylbenzene[40] (65)

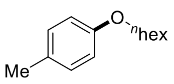

65

According to **GP3** starting from 1-iodo-4-methylbenzene (0.2 mmol, 1.0 eq.), hexan-1-ol (1 mmol, 5.0 eq.), TMG (0.4 mmol, 2.0 eq.) and bpyNi-PHI (10 mg, 6.0 wt % Nibpy) in MeCN (2 mL) for 48 h, the product **65** was isolated as colorless oil after flash chromatography (petroleum ether), 12.7 mg (33% yield). **^{1}H NMR** (400 MHz, $CDCl_3$) δ 7.07 (d, *J* = 8.3 Hz, 2H), 6.79 (d, *J* = 8.6 Hz, 2H), 3.92 (t, *J* = 6.6 Hz, 2H), 2.28 (s, 3H), 1.82 – 1.70 (m, 2H), 1.50 – 1.40 (m, 2H), 1.37 – 1.29 (m, 4H), 0.93 – 0.87 (m, 3H). **^{13}C NMR** (101 MHz, $CDCl_3$) δ 157.0, 129.9, 129.6, 114.4, 68.1, 31.6, 29.3, 25.8, 22.6, 20.5, 14.1.

4-(hexyloxy)pyridine[41] (66)

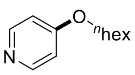

66

According to **GP3** starting from 4-iodopyridine (0.2 mmol, 1.0 eq.), hexan-1-ol (1 mmol, 5.0 eq.), TMG (0.4 mmol, 2.0 eq.) and bpyNi-PHI (10 mg, 6.0 wt % Nibpy) in MeCN (2 mL) for 12 h, the product **66** was isolated as colorless oil after flash chromatography (petroleum ether/ethyl acetate 1/1), 32.6 mg (91% yield). **^{1}H NMR** (400 MHz, $CDCl_3$) δ 8.41 (d, *J* = 5.6 Hz, 2H), 6.79 (d, *J* = 5.9 Hz, 2H), 4.00 (t, *J* = 6.6 Hz, 2H), 1.85 – 1.74 (m, 2H), 1.50 – 1.41 (m, 2H), 1.39 – 1.29 (m, 4H), 0.95 – 0.85 (m, 3H). **^{13}C NMR** (101 MHz, $CDCl_3$) δ 165.1, 151.0, 110.3, 67.9, 31.5, 28.8, 25.6, 22.6, 14.0.

Methyl 4-methoxybenzoate[16] (67)

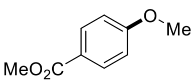

67

According to **GP3** starting from methyl 4-iodobenzoate (0.2 mmol, 1.0 eq.), methanol (1.0 mmol, 5.0 eq.), TMG (0.4 mmol, 2.0 eq.) and bpyNi-PHI (10 mg, 6.0 wt % Nibpy) in MeCN (2 mL) for 12 h, the product **67** was isolated as white solid after flash chromatography (petroleum ether/ethyl acetate 20/1), 31.6 mg (95% yield). **^{1}H NMR** (400 MHz, $CDCl_3$) δ 7.99 (d, *J* = 8.9 Hz, 2H), 6.91 (d, *J* = 8.9 Hz, 2H), 3.88 (s, 3H), 3.85 (s, 3H). **^{13}C NMR** (101 MHz, $CDCl_3$) δ 166.9, 163.3, 131.6, 122.6, 113.6, 55.4, 51.8.

Methyl 4-(methoxy-d3)benzoate[16] (68)

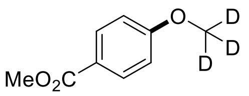

68

According to **GP3** starting from methyl 4-iodobenzoate (0.2 mmol, 1.0 eq.), methan-d_3-ol (1.0 mmol, 5.0 eq.), TMG (0.4 mmol, 2.0 eq.) and bpyNi-PHI (10 mg, 6.0 wt % Nibpy) in MeCN (2 mL) for 12 h, the product **68** was isolated as white solid after flash chromatography (petroleum ether/ethyl acetate 20/1), 31.5 mg (93% yield). **^{1}H NMR** (400 MHz, $CDCl_3$) δ 7.99 (d, *J* = 8.9 Hz, 2H), 6.91 (d, *J* = 9.0 Hz, 2H), 3.88 (s, 3H). **^{13}C NMR** (101 MHz, $CDCl_3$) δ 166.9, 163.3, 131.6, 122.6, 113.6, 51.8.

Methyl 4-(cyclopropylmethoxy)benzoate[42] (69)

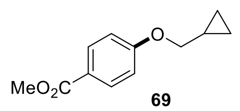

69

According to **GP3** starting from methyl 4-iodobenzoate (0.2 mmol, 1.0 eq.), cyclopropylmethanol (1 mmol, 5.0 eq.), TMG (0.4 mmol, 2.0 eq.) and bpyNi-PHI (10 mg, 6.0 wt % Nibpy) in MeCN (2 mL) for 12 h, the product **69** was isolated as pale yellow solid after flash chromatography (petroleum ether/ethyl acetate 20/1), 35.1 mg (85% yield). **^{1}H NMR** (400 MHz, $CDCl_3$) δ 8.00 – 7.94 (m, 2H), 6.94 – 6.86 (m, 2H), 3.88 (s, 3H), 3.85 (d, J = 6.9 Hz, 2H), 1.33 – 1.23 (m, 1H), 0.70 – 0.63 (m, 2H), 0.39 – 0.33 (m, 2H). **^{13}C NMR** (101 MHz, $CDCl_3$) δ 166.9, 162.8, 131.6, 122.4, 114.1, 72.9, 51.8, 10.1, 3.2.

Methyl 4-(benzyloxy)benzoate[16] (70)

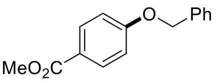

70

According to **GP3** starting from methyl 4-iodobenzoate (0.2 mmol, 1.0 eq.), phenylmethanol (0.6 mmol, 3.0 eq.), TMG (0.4 mmol, 2.0 eq.) and bpyNi-PHI (10 mg, 6.0 wt % Nibpy) in MeCN (2 mL) for 12 h, the product **70** was isolated as white solid after flash chromatography (petroleum ether/ethyl acetate 20/1), 40.7 mg (84% yield). **^{1}H NMR** (400 MHz, $CDCl_3$) δ 7.99 (d, J = 8.9 Hz, 2H), 7.48 – 7.31 (m, 5H), 6.99 (d, J = 8.9 Hz, 2H), 5.12 (s, 2H), 3.88 (s, 3H). **^{13}C NMR** (101 MHz, $CDCl_3$) δ 166.8, 162.5, 136.3, 131.6, 128.7, 128.2, 127.5, 122.8, 114.5, 70.1, 51.9.

Methyl 4-(pyridin-3-ylmethoxy)benzoate (71)

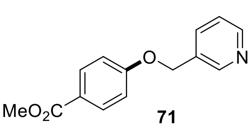

71

According to **GP3** starting from methyl 4-iodobenzoate (0.2 mmol, 1.0 eq.), pyridin-3-ylmethanol (0.6 mmol, 3.0 eq.), TMG (0.4 mmol, 2.0 eq.) and bpyNi-PHI (10 mg, 6.0 wt % Nibpy) in MeCN (2 mL) for 12 h, the product **71** was isolated as white solid after flash chromatography (petroleum ether/ethyl acetate 5/1), 42.8 mg (88% yield). mp = 49–50 °C. **^{1}H NMR** (400 MHz, $CDCl_3$) δ 8.70 (s, 1H), 8.61 (d, J = 3.6 Hz, 1H), 8.12 – 7.94 (m, 2H), 7.78 (d, J = 7.9 Hz, 1H), 7.34 (dd, J = 7.9, 4.7 Hz, 1H), 7.09 – 6.90 (m, 2H), 5.13 (s, 2H), 3.89 (s, 3H). **^{13}C NMR** (101 MHz, $CDCl_3$) δ 166.7, 162.0, 149.7, 149.0, 135.3, 131.9, 131.7, 123.6, 123.3, 114.4, 67.6, 51.9. **HRMS (ESI)** m/z calcd. For $C_{14}H_{23}NO_3$ $[M+H]^+$ 244.0968, found 244.0967.

Methyl 4-(2,2,2-trifluoroethoxy)benzoate[16] (72)

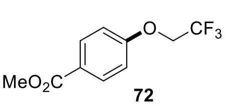

72

According to **GP3** starting from methyl 4-iodobenzoate (0.2 mmol, 1.0 eq.), 2,2,2-trifluoroethan-1-ol (0.6 mmol, 3.0 eq.), TMG (0.4 mmol, 2.0 eq.) and bpyNi-PHI (10 mg, 6.0 wt % Nibpy) in MeCN (2 mL) for 12 h, the product **72** was isolated as white solid after flash chromatography (petroleum ether/ethyl acetate 20/1), 35.1 mg (75% yield). **^{1}H NMR** (400 MHz, $CDCl_3$) δ 8.03 (d, J = 8.9 Hz, 2H), 6.97 (d, J = 8.9 Hz, 2H), 4.41 (q, J = 8.0 Hz, 2H), 3.90 (s, 3H). **^{13}C NMR** (101 MHz, $CDCl_3$) δ 166.4, 160.7, 131.8, 124.5, 123.1 (q, J = 277.9 Hz), 114.4, 65.6 (q, J = 36.1 Hz), 52.0. **^{19}F NMR** (377 MHz, $CDCl_3$) δ -73.9.

Methyl 4-(pent-4-en-1-yloxy)benzoate[43] (73)

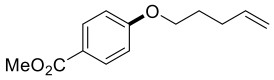

73

According to **GP3** starting from methyl 4-iodobenzoate (0.2 mmol, 1.0 eq.), pent-4-en-1-ol (1 mmol, 5.0 eq.), TMG (0.4 mmol, 2.0 eq.) and bpyNi-PHI (10 mg, 6.0 wt % Nibpy) in MeCN (2 mL) for 12 h, the product **73** was isolated as pale yellow solid after flash chromatography (petroleum ether/ethyl acetate 20/1), 39.6 mg (90% yield). **^{1}H NMR** (400 MHz, $CDCl_3$) δ 7.98 (d, *J* = 9.0 Hz, 2H), 6.90 (d, *J* = 9.0 Hz, 2H), 5.92 – 5.77 (m, 1H), 5.11 – 4.97 (m, 2H), 4.02 (t, *J* = 6.4 Hz, 2H), 3.88 (s, 3H), 2.24 (q, *J* = 7.2 Hz, 2H), 1.94 – 1.85 (m, 2H). **^{13}C NMR** (101 MHz, $CDCl_3$) δ 166.9, 162.9, 137.6, 131.6, 122.4, 115.4, 114.1, 67.3, 51.8, 30.0, 28.3.

Methyl 4-(but-3-yn-1-yloxy)benzoate[44] (74)

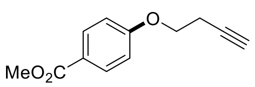

74

According to **GP3** starting from methyl 4-iodobenzoate (0.2 mmol, 1.0 eq.), but-3-yn-1-ol (1 mmol, 5.0 eq.), TMG (0.4 mmol, 2.0 eq.) and bpyNi-PHI (10 mg, 6.0 wt % Nibpy) in MeCN (2 mL) for 12 h, the product **74** was isolated as colorless oil after flash chromatography (petroleum ether/ethyl acetate 20/1), 30.8 mg (92% yield). **^{1}H NMR** (400 MHz, $CDCl_3$) δ 7.99 (d, *J* = 8.8 Hz, 2H), 6.93 (d, *J* = 8.8 Hz, 2H), 4.15 (t, *J* = 7.0 Hz, 2H), 3.89 (s, 3H), 2.74 – 2.74 (m, 2H), 2.08 – 2.02 (m, 1H). **^{13}C NMR** (101 MHz, $CDCl_3$) δ 166.8, 162.2, 131.6, 123.0, 114.2, 80.0, 70.1, 66.0, 51.9, 19.5.

Methyl 4-(2-hydroxyethoxy)benzoate[45] (75)

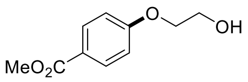

75

According to **GP3** starting from methyl 4-iodobenzoate (0.2 mmol, 1.0 eq.), ethane-1,2-diol (0.6 mmol, 3.0 eq.), TMG (0.4 mmol, 2.0 eq.) and bpyNi-PHI (10 mg, 6.0 wt % Nibpy) in MeCN (2 mL) for 24 h, the product **75** was isolated as white solid after flash chromatography (petroleum ether/ethyl acetate 1/1), 25.1 mg (64% yield). **^{1}H NMR** (400 MHz, $CDCl_3$) δ 8.03 – 7.96 (m, 2H), 6.98 – 6.90 (m, 2H), 4.14 (t, *J* = 5.1, 2H), 4.00 (t, *J* = 5.1, 2H), 3.89 (s, 3H). **^{13}C NMR** (101 MHz, $CDCl_3$) δ 166.8, 162.4, 131.7, 123.0, 114.1, 69.3, 61.3, 51.9.

Methyl 4-isopropoxybenzoate[16] (76)

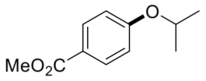

76

According to **GP3** starting from methyl 4-iodobenzoate (0.2 mmol, 1.0 eq.), propan-2-ol (1 mmol, 5.0 eq.), TMG (0.4 mmol, 2.0 eq.) and bpyNi-PHI (10 mg, 6.0 wt % Nibpy) in MeCN (2 mL) for 24 h, the product **76** was isolated as colorless oil after flash chromatography (petroleum ether/ethyl acetate 20/1), 35.0 mg (90% yield). **^{1}H NMR** (400 MHz, $CDCl_3$) δ 7.97 (d, *J* = 8.9 Hz, 2H), 6.88 (d, *J* = 8.9 Hz, 2H), 4.68 – 4.58 (m, 1H), 3.88 (s, 3H), 1.36 (d, *J* = 6.2 Hz, 6H). **^{13}C NMR** (101 MHz, $CDCl_3$) δ 166.9, 161.8, 131.6, 122.1, 115.0, 70.0, 51.8, 21.9.

Methyl 4-(cyclohexyloxy)benzoate[16] (77)

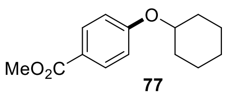

77

According to **GP3** starting from methyl 4-iodobenzoate (0.2 mmol, 1.0 eq.), cyclohexanol (0.6 mmol, 3.0 eq.), TMG (0.4 mmol, 2.0 eq.) and bpyNi-PHI (10 mg, 6.0 wt % Nibpy) in MeCN (2 mL) for 24 h, the product **77** was isolated as white solid after flash chromatography (petroleum ether/ethyl acetate 5/1), 39.8 mg (85% yield). **^{1}H NMR** (400 MHz, $CDCl_3$) δ 7.96 (d, *J* = 8.9 Hz, 2H), 6.89 (d, *J* = 8.9 Hz, 2H), 4.38 – 4.27 (m, 1H), 3.88 (s, 3H), 2.04 – 1.91 (m, 2H), 1.86 – 1.72 (m, 2H), 1.63 – 1.47 (m, 2H), 1.45 – 1.22 (m, 4H). **^{13}C NMR** (101 MHz, $CDCl_3$) δ 166.9, 161.8, 131.6, 122.1, 115.1, 75.4, 51.8, 31.6, 25.5, 23.7.

Methyl 4-hydroxybenzoate[16] (78)

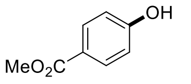

78

According to **GP3** starting from methyl 4-iodobenzoate (0.2 mmol, 1.0 eq.), water (2 mmol, 10.0 eq.), TMG (0.4 mmol, 2.0 eq.) and bpyNi-PHI (10 mg, 6.0 wt % Nibpy) in MeCN (2 mL) for 16 h, the product **78** was isolated as white soild after flash chromatography (petroleum ether/ethyl acetate 5/1), 28.9 mg (95% yield). **^{1}H NMR** (400 MHz, $CDCl_3$) δ 7.95 (d, *J* = 8.7 Hz, 2H), 6.88 (d, *J* = 8.8 Hz, 2H), 3.89 (s, 3H). **^{13}C NMR** (101 MHz, $CDCl_3$) δ 167.3, 160.2, 132.0, 122.4, 115.3, 52.1.

4-(methoxycarbonyl)phenyl furan-3-carboxylate (79)

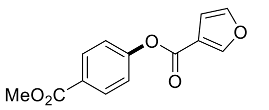

79

According to **GP4** starting from methyl 4-iodobenzoate(0.2 mmol, 1.0 eq.), furan-3-carboxylic acid (0.3 mmol, 1.5 eq.), BIPA (0.6 mmol, 3.0 eq.) and bpyNi-PHI (10 mg, 6.0 wt % Nibpy) in DMSO (2 mL) for 12 h, the product **79** was isolated as pale yellow solid after flash chromatography (petroleum ether/ethyl acetate 10/1), 39.4 mg (80% yield). mp = 123–124 °C. **^{1}H NMR** (400 MHz, $CDCl_3$) δ 8.21 (s, 1H), 8.11 (d, *J* = 8.7 Hz, 2H), 7.54 – 7.48 (m, 1H), 7.29 – 7.26 (m, 2H), 6.89 – 6.84 (m, 1H), 3.93 (s, 3H). **^{13}C NMR** (101 MHz, $CDCl_3$) δ 166.3, 160.8, 154.1, 148.9, 144.2, 131.2, 127.8, 121.7, 118.5, 110.0, 52.2. **HRMS (ESI)** m/z calcd. For $C_{13}H_{11}O_5$ $[M+H]^+$ 247.0601, found 247.0600.

4-(methoxycarbonyl)phenyl 1-methyl-1*H*-indole-2-carboxylate (80)

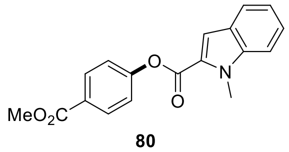

80

According to **GP4** starting from methyl 4-iodobenzoate (0.2 mmol, 1.0 eq.), 1-methyl-1*H*-indole-2-carboxylic acid (0.3 mmol, 1.5 eq.), BIPA (0.6 mmol, 3.0 eq.) and bpyNi-PHI (10 mg, 6.0 wt % Nibpy) in DMSO (2 mL) for 12 h, the product **80** was isolated as pale yellow solid after flash chromatography (petroleum ether/ethyl acetate 10/1), 52.0 mg (84% yield). mp = 132–133 °C. **^{1}H NMR** (400 MHz, $CDCl_3$) δ 8.13 (d, *J* = 8.7 Hz, 2H), 7.73 (d, *J* = 8.0 Hz, 1H), 7.56 (s, 1H), 7.45 – 7.38 (m, 2H), 7.32 (d, *J* = 8.7 Hz, 2H), 7.22 – 7.16 (m, 1H), 4.11 (s, 3H), 3.93 (s, 3H). **^{13}C NMR** (101 MHz, $CDCl_3$) δ 166.4, 160.0, 154.3, 140.3, 131.2, 127.7, 126.3, 125.9, 125.8, 123.0, 121.9, 121.0, 112.1, 110.4, 52.2, 31.7. **HRMS (ESI)** m/z calcd. For $C_{18}H_{16}NO_4$ $[M+H]^+$ 310.1074, found 310.1071.

Methyl 4-(2-methoxyacetoxy)benzoate[47] (81)

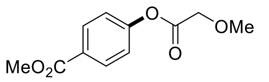

81

According to **GP4** starting from methyl 4-iodobenzoate (0.2 mmol, 1.0 eq.), 2-methoxyacetic acid (0.3 mmol, 1.5 eq.), BIPA (0.6 mmol, 3.0 eq.) and bpyNi-PHI (10 mg, 6.0 wt % Nibpy) in DMSO (2 mL) for 24 h, the product **81** was isolated as white solid after flash chromatography (petroleum ether/ethyl acetate 20/1), 26.9 mg (60% yield). **^{1}H NMR** (400 MHz, $CDCl_3$) δ 8.09 (d, *J* = 8.8 Hz, 2H), 7.21 (d, *J* = 8.8 Hz, 2H), 4.31 (s, 2H), 3.92 (s, 3H), 3.55 (s, 3H). **^{13}C NMR** (101 MHz, $CDCl_3$) δ 168.3, 166.2, 153.7, 131.3, 128.0, 121.4, 69.7, 59.6, 52.2.

1-(*tert*-butyl) 4-(4-(methoxycarbonyl)phenyl) piperidine-1,4-dicarboxylate[17] (82)

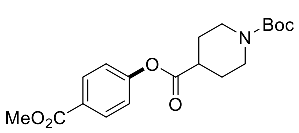

82

According to **GP4** starting from methyl 4-iodobenzoate (0.2 mmol, 1.0 eq.), 1-(*tert*-butoxycarbonyl)piperidine-4-carboxylic acid (0.3 mmol, 1.5 eq.), BIPA (0.6 mmol, 3.0 eq.) and bpyNi-PHI (10 mg, 6.0 wt % Nibpy) in DMSO (2 mL) for 36 h, the product **82** was isolated as white solid after flash chromatography (petroleum ether/ethyl acetate 10/1), 53.1 mg (73% yield). **^{1}H NMR** (400 MHz, $CDCl_3$) δ 8.07 (d, *J* = 8.7 Hz, 2H), 7.15 (d, *J* = 8.7 Hz, 2H), 4.18 – 4.05 (m, 2H), 3.91 (s, 3H), 3.00 – 2.87 (m, 2H), 2.78 – 2.67 (m, 1H), 2.08 – 1.98 (m, 2H), 1.85 – 1.71 (m, 2H), 1.47 (s, 9H). **^{13}C NMR** (101 MHz, $CDCl_3$) δ 172.5, 166.3, 154.7, 154.3, 131.2, 127.8, 121.5, 79.8, 52.2, 43.0 (br), 41.3, 28.4, 27.9.

Methyl 4-((2-phenylacryloyl)oxy)benzoate (83)

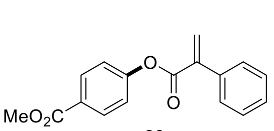

83

According to **GP4** starting from methyl 4-iodobenzoate (0.2 mmol, 1.0 eq.), 2-phenylacrylic acid (0.3 mmol, 1.5 eq.), BIPA (0.6 mmol, 3.0 eq.) and bpyNi-PHI (10 mg, 6.0 wt % Nibpy) in DMSO (2 mL) for 12 h, the product **83** was isolated as white solid after flash chromatography (petroleum ether/ethyl acetate 10/1), 45.7 mg (81% yield). mp = 63–64 °C. **^{1}H NMR** (400 MHz, $CDCl_3$) δ 8.09 (d, *J* = 8.7 Hz, 2H), 7.53 – 7.46 (m, 2H), 7.42 – 7.34 (m, 3H), 7.26 – 7.22 (m, 2H), 6.62 (s, 1H), 6.11 (s, 1H), 3.91 (s, 3H). **^{13}C NMR** (101 MHz, $CDCl_3$) δ 166.3, 164.6, 154.5, 140.6, 136.1, 131.2, 129.1, 128.6, 128.4, 128.3, 127.8, 121.7, 52.2. **HRMS (ESI)** m/z calcd. For $C_{17}H_{15}O_4$ $[M+H]^+$ 283.0965, found 283.0964.

Methyl (*E*)-4-(but-2-enoyloxy)benzoate (84)

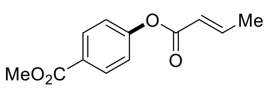

84

According to **GP4** starting from methyl 4-iodobenzoate (0.2 mmol, 1.0 eq.), (*E*)-but-2-enoic acid (0.3 mmol, 1.5 eq.), BIPA (0.6 mmol, 3.0 eq.) and bpyNi-PHI (10 mg, 6.0 wt % Nibpy) in DMSO (2 mL) for 24 h, the product **84** was isolated as white solid after flash chromatography (petroleum ether/ethyl acetate 10/1), 26.4 mg (60% yield). mp = 50–51 °C. **^{1}H NMR** (400 MHz, $CDCl_3$) δ 8.16 – 8.01 (m, 2H), 7.28 – 7.14 (m, 3H), 6.08 – 5.99 (m, 1H), 3.91 (s, 3H), 1.98 (dd, *J* = 7.0, 1.7 Hz, 3H). **^{13}C NMR** (101 MHz, $CDCl_3$) δ 166.4, 164.2, 154.5, 147.8, 131.1, 127.5, 121.7, 121.7, 52.2, 18.3. **HRMS (ESI)** m/z calcd. For $C_{12}H_{13}O_4$ $[M+H]^+$ 221.0808, found 221.0806.

Methyl 4-(cinnamoyloxy)benzoate[17] (85)

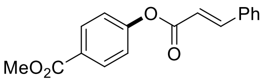

85

According to **GP4** starting from methyl 4-iodobenzoate(0.2 mmol, 1.0 eq.), cinnamic acid (0.3 mmol, 1.5 eq.), BIPA (0.6 mmol, 3.0 eq.) and bpyNi-PHI (10 mg, 6.0 wt % Nibpy) in DMSO (2 mL) for 24 h, the product **85** was isolated as white solid after flash chromatography (petroleum ether/ethyl acetate 10/1), 45.7 mg (81% yield). **^{1}H NMR** (400 MHz, $CDCl_3$) δ 8.10 (d, *J* = 8.7 Hz, 2H), 7.89 (d, *J* = 16.0 Hz, 1H), 7.62 – 7.55 (m, 2H), 7.47 – 7.38 (m, 3H), 7.27 – 7.23 (m, 2H), 6.63 (d, *J* = 16.0 Hz, 1H), 3.92 (s, 3H). **^{13}C NMR** (101 MHz, $CDCl_3$) δ 166.4, 164.8, 154.5, 147.3, 134.0, 131.2, 130.9, 129.1, 128.4, 127.7, 121.7, 116.8, 52.2.

Methyl 3-((4-bromophenyl)thio)propanoate[47] (86)

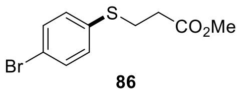

86

According to **GP5** starting from 1-bromo-3-iodobenzene (0.2 mmol, 1.0 eq.), methyl 3-mercaptopropanoate (0.3 mmol, 1.5 eq.), $^{i}Pr_2NH$ (0.6 mmol, 3.0 eq.) and bpyNi-PHI (10 mg, 6.0 wt % Nibpy) in MeCN (2 mL) for 16 h, the product **86** was isolated as white solid after flash chromatography (petroleum ether/ethyl acetate 10/1), 53.9 mg (98% yield). **^{1}H NMR** (400 MHz, $CDCl_3$) δ 7.44 – 7.38 (m, 2H), 7.26 – 7.20 (m, 2H), 3.68 (s, 3H), 3.15 (t, *J* = 7.4 Hz, 2H), 2.62 (t, *J* = 7.4 Hz, 2H). **^{13}C NMR** (101 MHz, $CDCl_3$) δ 172.0, 134.5, 132.1, 131.6, 120.5, 51.9, 34.1, 29.1.

Methyl 3-(phenylthio)propanoate[47] (87)

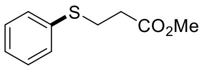

87

According to **GP5** starting from iodobenzene (0.2 mmol, 1.0 eq.), methyl 3-mercaptopropanoate (0.3 mmol, 1.5 eq.), $^{i}Pr_2NH$ (0.6 mmol, 3.0 eq.) and bpyNi-PHI (10 mg, 6.0 wt % Nibpy) in MeCN (2 mL) for 16 h, the product **87** was isolated as colorless oil after flash chromatography (petroleum ether/ethyl acetate 10/1), 38.5 mg (98% yield). **^{1}H NMR** (400 MHz, $CDCl_3$) δ 7.39 – 7.34 (m, 2H), 7.32 – 7.26 (m, 2H), 7.24 – 7.18 (m, 1H), 3.68 (s, 3H), 3.17 (t, *J* = 7.4 Hz, 2H), 2.63 (t, *J* = 7.4 Hz, 2H). **^{13}C NMR** (101 MHz, $CDCl_3$) δ 172.2, 135.2, 130.1, 129.0, 126.6, 51.8, 34.2, 29.1.

Methyl 3-(*p*-tolylthio)propanoate[47] (88)

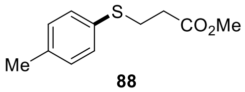

88

According to **GP5** starting from 1-iodo-4-methylbenzene (0.2 mmol, 1.0 eq.), methyl 3-mercaptopropanoate (0.3 mmol, 1.5 eq.), $^{i}Pr_2NH$ (0.6 mmol, 3.0 eq.) and bpyNi-PHI (10 mg, 6.0 wt % Nibpy) in MeCN (2 mL) for 16 h, the product **88** was isolated as colorless oil after flash chromatography (petroleum ether/ethyl acetate 10/1), 40.0 mg (95% yield). **^{1}H NMR** (400 MHz, $CDCl_3$) δ 7.33 – 7.27 (m, 2H), 7.11 (d, *J* = 7.6 Hz, 2H), 3.67 (s, 3H), 3.11 (t, *J* = 7.4 Hz, 2H), 2.60 (t, *J* = 7.4 Hz, 2H), 2.32 (s, 3H). **^{13}C NMR** (101 MHz, $CDCl_3$) δ 172.3, 136.9, 131.3, 131.1, 129.8, 51.8, 34.3, 29.8, 21.0.

Methyl 3-((4-methoxyphenyl)thio)propanoate[47] (89)

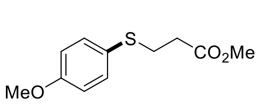

89

According to **GP5** starting from 1-iodo-4-methoxybenzene (0.2 mmol, 1.0 eq.), methyl 3-mercaptopropanoate (0.3 mmol, 1.5 eq.), $^{i}Pr_2NH$ (0.6 mmol, 3.0 eq.) and bpyNi-PHI (10 mg, 6.0 wt % Nibpy) in MeCN (2 mL) for 16 h, the product **89** was isolated as colorless iol after flash chromatography (petroleum ether/ethyl acetate 5/1), 36.2 mg (80% yield). **^{1}H NMR** (400 MHz, $CDCl_3$) δ 7.42 – 7.34 (m, 2H), 6.91 – 6.80 (m, 2H), 3.80 (s, 3H), 3.67 (s, 3H), 3.05 (t, J = 7.4 Hz, 2H), 2.57 (t, J = 7.4 Hz, 2H). **^{13}C NMR** (101 MHz, $CDCl_3$) δ 172.3, 159.4, 134.2, 125.1, 114.7, 55.4, 51.8, 34.4, 31.1.

Methyl 3-((4-hydroxyphenyl)thio)propanoate (90)

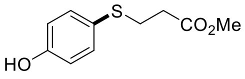

90

According to **GP5** starting from 4-iodophenol (0.2 mmol, 1.0 eq.), methyl 3-mercaptopropanoate (0.3 mmol, 1.5 eq.), $^{i}Pr_2NH$ (0.6 mmol, 3.0 eq.) and bpyNi-PHI (10 mg, 6.0 wt % Nibpy) in MeCN (2 mL) for 16 h, the product **90** was isolated as colorless oil after flash chromatography (petroleum ether/ethyl acetate 5/1), 36.1 mg (85% yield). **^{1}H NMR** (400 MHz, $CDCl_3$) δ 7.35 – 7.29 (m, 2H), 6.81 – 6.75 (m, 2H), 5.65 (s, 1H), 3.68 (s, 3H), 3.04 (t, J = 7.3 Hz, 2H), 2.58 (t, J = 7.3 Hz, 2H). **^{13}C NMR** (101 MHz, $CDCl_3$) δ 172.8, 155.7, 134.5, 124.9, 116.2, 51.9, 34.5, 31.2. **HRMS (ESI)** m/z calcd. For $C_{10}H_{13}O_3S$ $[M+H]^+$ 213.0580, found 213.0578.

Methyl 3-((4-aminophenyl)thio)propanoate[48] (91)

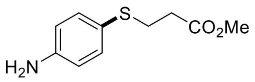

91

According to **GP5** starting from 4-iodoaniline (0.2 mmol, 1.0 eq.), methyl 3-mercaptopropanoate (0.3 mmol, 1.5 eq.), $^{i}Pr_2NH$ (0.6 mmol, 3.0 eq.) and bpyNi-PHI (10 mg, 6.0 wt % Nibpy) in MeCN (2 mL) for 24 h, the product **91** was isolated as brown oil after flash chromatography (petroleum ether/ethyl acetate 2/1), 36.3 mg (86% yield). **^{1}H NMR** (400 MHz, $CDCl_3$) δ 7.55 – 6.96 (m, 2H), 6.78 – 6.45 (m, 2H), 3.66 (s, 3H), 2.99 (t, J = 7.4 Hz, 2H), 2.56 (t, J = 7.4 Hz, 2H). **^{13}C NMR** (101 MHz, $CDCl_3$) δ 172.5, 146.4, 134.9, 121.9, 115.6, 51.7, 34.5, 31.6.

Methyl 3-((3-formylphenyl)thio)propanoate (92)

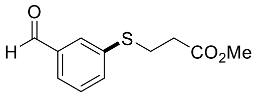

92

According to **GP5** starting from 3-iodobenzaldehyde (0.2 mmol, 1.0 eq.), methyl 3-mercaptopropanoate (0.3 mmol, 1.5 eq.), $^{i}Pr_2NH$ (0.6 mmol, 3.0 eq.) and bpyNi-PHI (10 mg, 6.0 wt % Nibpy) in MeCN (2 mL) for 16 h, the product **92** was isolated as colorless oil after flash chromatography (petroleum ether/ethyl acetate 5/1), 41.3 mg (92% yield). **^{1}H NMR** (400 MHz, $CDCl_3$) δ 9.99 (s, 1H), 7.85 – 7.81 (m, 1H), 7.73 – 7.67 (m, 1H), 7.63 – 7.56 (m, 1H), 7.47 (t, J = 7.7 Hz, 1H), 3.70 (s, 3H), 3.25 (t, J = 7.3 Hz, 2H), 2.67 (t, J = 7.3 Hz, 2H). **^{13}C NMR** (101 MHz, $CDCl_3$) δ 191.7, 171.9, 137.5, 137.0, 135.1, 129.6, 127.8, 52.0, 34.0, 28.5. **HRMS (ESI)** m/z calcd. For $C_{11}H_{13}O_3S$ $[M+H]^+$ 225.0580, found 225.0577.

Methyl 3-(pyridin-4-ylthio)propanoate[49] (93)

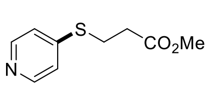

93

According to **GP5** starting from 4-iodopyridine (0.2 mmol, 1.0 eq.), methyl 3-mercaptopropanoate (0.3 mmol, 1.5 eq.), ^{i}Pr$_2$NH (0.6 mmol, 3.0 eq.) and bpyNi-PHI (10 mg, 6.0 wt % Nibpy) in MeCN (2 mL) for 16 h, the product **93** was isolated as colorless oil after flash chromatography (petroleum ether/ethyl acetate 5/1), 32.3 mg (82% yield). **^{1}H NMR** (400 MHz, $CDCl_3$) δ 8.52 – 8.31 (m, 2H), 7.18 – 7.10 (m, 2H), 3.72 (s, 3H), 3.27 (t, J = 7.3 Hz, 2H), 2.73 (t, J = 7.4 Hz, 2H). **^{13}C NMR** (101 MHz, $CDCl_3$) δ 171.7, 149.4, 148.1, 120.8, 52.1, 33.4, 25.6.

Methyl 4-((2-hydroxyethyl)thio)benzoate[17] (94)

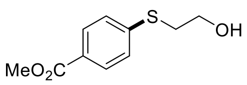

94

According to **GP5** starting from methyl 4-iodobenzoate (0.2 mmol, 1.0 eq.), 2-mercaptoethan-1-ol (0.6 mmol, 3.0 eq.), ^{i}Pr$_2$NH (0.6 mmol, 3.0 eq.) and bpyNi-PHI (10 mg, 6.0 wt % Nibpy) in MeCN (2 mL) for 24 h, the product **94** was isolated as white solid after flash chromatography (petroleum ether/ethyl acetate 5/1), 50.7 mg (70% yield). **^{1}H NMR** (400 MHz, $CDCl_3$) δ 7.94 (d, J = 8.5 Hz, 2H), 7.36 (d, J = 8.7 Hz, 2H), 3.90 (s, 3H), 3.83 (t, J = 6.1 Hz, 2H), 3.21 (t, J = 6.1 Hz, 2H). **^{13}C NMR** (101 MHz, $CDCl_3$) δ 166.7, 142.4, 130.1, 127.4, 127.3, 60.5, 52.1, 35.5.

Methyl 4-(p-tolylthio)benzoate[50] (95)

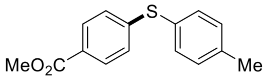

95

According to **GP5** starting from methyl 4-iodobenzoate (0.2 mmol, 1.0 eq.), 4-methylbenzenethiol (0.3 mmol, 1.5 eq.), ^{i}Pr$_2$NH (0.6 mmol, 3.0 eq.) and bpyNi-PHI (10 mg, 6.0 wt % Nibpy) in MeCN (2 mL) for 36 h, the product **95** was isolated as y white solid after flash chromatography (petroleum ether/ethyl acetate 20/1), 43.9 mg (85% yield). **^{1}H NMR** (400 MHz, $CDCl_3$) δ 7.86 (d, J = 8.6 Hz, 2H), 7.39 (d, J = 8.2 Hz, 2H), 7.20 (d, J = 7.8 Hz, 2H), 7.14 (d, J = 8.5 Hz, 2H), 3.87 (s, 3H), 2.38 (s, 3H). **^{13}C NMR** (101 MHz, $CDCl_3$) δ 166.8, 145.5, 139.2, 134.4, 130.5, 130.0, 128.2, 127.0, 126.7, 52.0, 21.3.

Methyl 4-(benzoylthio)benzoate[51] (96)

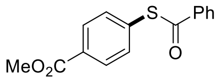

96

According to **GP5** starting from methyl 4-iodobenzoate (0.2 mmol, 1.0 eq.), benzothioic *S*-acid (0.3 mmol, 1.5 eq.), ^{i}Pr$_2$NH (0.6 mmol, 3.0 eq.) and bpyNi-PHI (10 mg, 6.0 wt % Nibpy) in MeCN (2 mL) for 48 h, the product **96** was isolated as brown oil after flash chromatography (petroleum ether/ethyl acetate 20/1), 47.9 mg (88% yield). **^{1}H NMR** (400 MHz, $CDCl_3$) δ 8.11 (d, J = 8.1 Hz, 2H), 8.02 (d, J = 7.7 Hz, 2H), 7.66 – 7.57 (m, 3H), 7.50 (t, J = 7.7 Hz, 2H), 3.94 (s, 3H). **^{13}C NMR** (101 MHz, $CDCl_3$) δ 189.0, 166.5, 136.4, 134.7, 134.0, 133.2, 130.9, 130.2, 128.9, 127.6, 52.3.

(1*R*,2*S*,5*R*)-2-isopropyl-5-methylcyclohexyl 4-methoxybenzoate[52] (97)

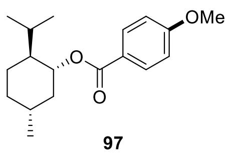

97

According to **GP3** starting from (1*R*,2*S*,5*R*)-2-isopropyl-5-methylcyclohexyl 4-iodobenzoate (0.2 mmol, 1.0 eq.), methanol (1.0 mmol, 5.0 eq.), TMG (0.4 mmol, 2.0 eq.) and bpyNi-PHI (10 mg, 6.0 wt % Nibpy) in MeCN (2 mL) for 12 h, the product **97** was isolated as colorless oil after flash chromatography (petroleum ether/ethyl acetate 20/1), 48.8 mg (84% yield). **^{1}H NMR** (400 MHz, $CDCl_3$) δ 8.06 – 7.92 (m, 2H), 7.01 – 6.85 (m, 2H), 4.90 (td, *J* = 10.9, 4.4 Hz, 1H), 3.85 (s, 3H), 2.14 – 2.09 (m, 1H), 2.00 – 1.9 (m, 1H), 1.76 – 1.67 (m, 2H), 1.62 – 1.47 (m, 2H), 1.18 – 1.04 (m, 2H), 0.97 – 0.87 (m, 7H), 0.79 (d, *J* = 7.0 Hz, 3H). **^{13}C NMR** (101 MHz, $CDCl_3$) δ 165.9, 163.2, 131.5, 123.3, 113.5, 74.4, 55.4, 47.3, 41.1, 34.4, 31.5, 26.5, 23.7, 22.1, 20.8, 16.6.

(1*R*,2*S*,5*R*)-2-isopropyl-5-methylcyclohexyl 4-(benzoyloxy)benzoate (98)

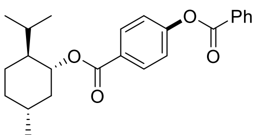

98

According to **GP4** starting from (1*R*,2*S*,5*R*)-2-isopropyl-5-methylcyclohexyl 4-iodobenzoate (0.2 mmol, 1.0 eq.), benzoic acid (0.3 mmol, 1.5 eq.), BIPA (0.6 mmol, 3.0 eq.) and bpyNi-PHI (10 mg, 6.0 wt % Nibpy) in DMSO (2 mL) for 12 h, the product **98** was isolated as colorless oil after flash chromatography (petroleum ether/ethyl acetate 20/1), 57.8 mg (76% yield). **^{1}H NMR** (400 MHz, $CDCl_3$) δ 8.27 – 8.17 (m, 2H), 8.16 – 8.10 (m, 2H), 7.69 – 7.61 (m, 1H), 7.53 (dd, *J* = 8.4, 7.1 Hz, 2H), 7.34 – 7.27 (m, 2H), 4.95 (td, *J* = 10.9, 4.4 Hz, 1H), 2.19 – 2.09 (m, 1H), 1.96 (qt, *J* = 7.0, 3.5 Hz, 1H), 1.78 – 1.69 (m, 2H), 1.63 – 1.50 (m, 2H), 1.19 – 1.05 (m, 2H), 0.98 – 0.88 (m, 7H), 0.81 (d, *J* = 7.0 Hz, 3H). **^{13}C NMR** (101 MHz, $CDCl_3$) δ 165.4, 164.7, 154.5, 133.9, 131.2, 130.3, 129.2, 128.7, 128.5, 121.7, 75.0, 47.3, 41.0, 34.3, 31.5, 26.5, 23.6, 22.1, 20.8, 16.5. **HRMS (ESI)** m/z calcd. For $C_{24}H_{27}O_4$ $[M-H]^-$ 379.1909, found 379.1919.

1-(*tert*-butyl) 2-(4-((((1*R*,2*S*,5*R*)-2-isopropyl-5-methylcyclohexyl)oxy)carbonyl) phenyl) (*S*)-pyrrolidine-1,2-dicarboxylate (99)

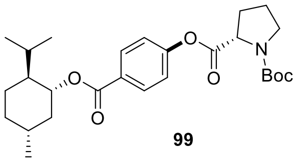

99

According to **GP4** starting from (1*R*,2*S*,5*R*)-2-isopropyl-5-methylcyclohexyl 4-iodobenzoate (0.2 mmol, 1.0 eq.), (*tert*-butoxycarbonyl)-*L*-proline (0.3 mmol, 1.5 eq.), BIPA (0.6 mmol, 3.0 eq.) and bpyNi-PHI (10 mg, 6.0 wt % Nibpy) in DMSO (2 mL) for 12 h, the product **99** was isolated as colorless oil after flash chromatography (dichloromethane / methanol 50/1), 62.5 mg (66% yield). **^{1}H NMR** (400 MHz, $CDCl_3$) rotameric mixture, δ 8.15 – 7.93 (m, 2H), 7.25 – 7.09 (m, 2H), 4.98 – 4.84 (m, 1H), 4.61 – 4.41 (m, 1H), 3.73 – 3.25 (m, 2H), 2.47 – 2.30 (m, 1H), 2.25 – 1.87 (m, 5H), 1.73 (d, *J* = 11.6 Hz, 2H), 1.60 – 1.40 (m, 11H), 1.21 – 1.02 (m, 2H), 1.02 – 0.86 (m, 7H), 0.79 (d, *J* = 6.9, 3H). **^{13}C NMR** (101 MHz, $CDCl_3$) rotameric mixture, resonances for minor rotamer are enclosed in parenthesis δ (171.2) 171.2, (165.3) 165.2, 154.5 (154.3), 154.0 (153.7), (131.2) 131.1, 128.6 (128.4), 121.5 (121.1), 80.3 (80.1), 75.1, (75.0) 59.2 (59.1), 47.3, (46.6) 46.5, 41.0, (34.3) 31.4, 31.0 (30.0), 28.4, 26.5 (24.6), (23.7) 23.6, 22.0, 20.8, 16.5. **HRMS (ESI)** m/z calcd. For $C_{27}H_{40}NO_6$ $[M+H]^+$ 474.2850, found 474.2842.

(1*R*,2*S*,5*R*)-2-isopropyl-5-methylcyclohexyl 4-hydroxybenzoate[53] (100)

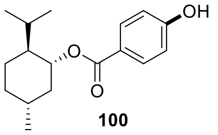

100

According to **GP3** starting from (1*R*,2*S*,5*R*)-2-isopropyl-5-methylcyclohexyl 4-iodobenzoate (0.2 mmol, 1.0 eq.), water (2 mmol, 10.0 eq.), TMG (0.4 mmol, 2.0 eq.) and bpyNi-PHI (10 mg, 6.0 wt % Nibpy) in MeCN (2 mL) for 12 h, the product **100** was isolated as colorless oil after flash chromatography (petroleum ether/ethyl acetate 5/1), 47.5 mg (86% yield). **^{1}H NMR** (400 MHz, $CDCl_3$) δ 8.05 – 7.88 (m, 2H), 7.04 – 6.80 (m, 2H), 6.37 – 6.16 (m, 1H), 4.90 (td, *J* = 10.8, 4.4 Hz, 1H), 2.15 – 2.08 (m, 1H), 1.99 – 1.91 (m, 1H), 1.74 – 1.68 (m, 2H), 1.59 – 1.47 (m, 2H), 1.15 – 1.04 (m, 2H), 0.96 – 0.84 (m, 7H), 0.78 (d, *J* = 6.9 Hz, 3H). **^{13}C NMR** (101 MHz, $CDCl_3$) δ 166.3, 160.0, 131.9, 123.1, 115.2, 74.8, 47.3, 41.0, 34.3, 31.4, 26.5, 23.7, 22.0, 20.8, 16.6.

(1*R*,2*S*,5*R*)-2-isopropyl-5-methylcyclohexyl 4-(phenylamino)benzoate (101)

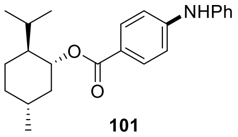

101

According to **GP2** starting from (1*R*,2*S*,5*R*)-2-isopropyl-5-methylcyclohexyl 4-iodobenzoate (0.2 mmol, 1.0 eq.), aniline (0.3 mmol, 1.5 eq.), TMG (0.4 mmol, 2.0 eq.) and bpyNi-PHI (10 mg, 6.0 wt % Nibpy) in MeCN (2 mL) for 12 h, the product **101** was isolated as brown oil after flash chromatography (petroleum ether/ethyl acetate 5/1), 56.2 mg (80% yield). **^{1}H NMR** (400 MHz, $CDCl_3$) δ 7.96 – 7.83 (m, 2H), 7.37 – 7.29 (m, 2H), 7.19 – 7.11 (m, 2H), 7.07 – 7.02 (m, 1H), 7.02 – 6.95 (m, 2H), 6.08 (s, 1H), 4.89 (td, *J* = 10.9, 4.4 Hz, 1H), 2.14 – 2.08 (m, 1H), 2.01 – 1.92 (m, 1H), 1.75 – 1.66 (m, 2H), 1.58 – 1.48 (m, 2H), 1.19 – 1.02 (m, 2H), 0.96 – 0.86 (m, 7H), 0.79 (d, *J* = 6.9 Hz, 3H). **^{13}C NMR** (101 MHz, $CDCl_3$) δ 166.0, 147.9, 141.0, 131.5, 129.5, 123.0, 121.9, 120.3, 114.7, 74.2, 47.4, 41.1, 34.4, 31.5, 26.6, 23.7, 22.1, 20.8, 16.6. **HRMS (ESI)** m/z calcd. For $C_{23}H_{28}NO_2$ $[M-H]^-$ 350.2120, found 350.2128.

(1*R*,2*S*,5*R*)-2-isopropyl-5-methylcyclohexyl 4-((4-methylphenyl)sulfonamido) benzoate (102)

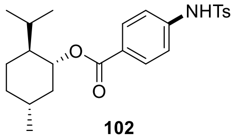

102

According to **GP2** starting from (1*R*,2*S*,5*R*)-2-isopropyl-5-methylcyclohexyl 4-iodobenzoate (0.2 mmol, 1.0 eq.), 4-methylbenzenesulfonamide (0.3 mmol, 1.5 eq.), TMG (0.4 mmol, 2.0 eq.) and bpyNi-PHI (10 mg, 6.0 wt % Nibpy) in MeCN (2 mL) for 16 h, the product **102** was isolated as white solid after flash chromatography (petroleum ether/ethyl acetate 5/1), 72.2 mg (84% yield). mp = 105–106 °C. **^{1}H NMR** (400 MHz, $CDCl_3$) δ 7.91 (d, *J* = 8.7 Hz, 2H), 7.75 (d, *J* = 8.4 Hz, 2H), 7.50 (s, 1H), 7.25 (d, *J* = 7.9 Hz, 2H), 7.14 (d, *J* = 8.7 Hz, 2H), 4.88 (td, *J* = 10.9, 4.4 Hz, 1H), 2.38 (s, 3H), 2.10 – 2.04 (m, 1H), 1.94 – 1.85 (m, 1H), 1.75 – 1.67 (m, 2H), 1.57 – 1.46 (m, 2H), 1.15 – 1.10 (m, 2H), 0.94 – 0.86 (m, 7H), 0.76 (d, *J* = 7.0 Hz, 3H). **^{13}C NMR** (101 MHz, $CDCl_3$) δ 165.5, 144.4, 140.9, 135.9, 131.0, 129.9, 127.3, 126.8, 118.9, 74.9, 47.2, 41.0, 34.3, 31.4, 26.5, 23.6, 22.0, 21.6, 20.8, 16.5. **HRMS (ESI)** m/z calcd. For $C_{24}H_{30}NO_4S$ $[M-H]^-$ 428.1896, found 428.1902.

(1*R*,2*S*,5*R*)-2-isopropyl-5-methylcyclohexyl 4-(diphenylphosphoryl)benzoate (103)

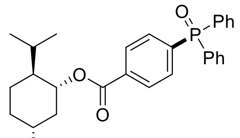

103

According to **GP1** starting from (1*R*,2*S*,5*R*)-2-isopropyl-5-methylcyclohexyl 4-iodobenzoate (0.2 mmol, 1.0 eq.), diphenylphosphine oxide (0.3 mmol, 1.5 eq.), ^{i}Pr$_2$NH (0.6 mmol, 3.0 eq.) and bpyNi-PHI (10 mg, 6.0 wt % Nibpy) in DMF (2 mL) for 48 h, the product **103** was isolated as white solid after flash chromatography (petroleum ether/ethyl acetate 2/1), 78.3 mg (85% yield). mp = 148–149 °C. **^{1}H NMR** (400 MHz, CDCl$_3$) δ 8.12 (dd, *J* = 8.4, 2.5 Hz, 2H), 7.77 (dd, *J* = 11.5, 8.3 Hz, 2H), 7.71 – 7.63 (m, 4H), 7.61 – 7.54 (m, 2H), 7.51 – 7.44 (m, 4H), 4.99 – 4.89 (m, 1H), 2.17 – 2.07 (m, 1H), 1.98 – 1.80 (m, 2H), 1.75 – 1.69 (m, 2H), 1.61 – 1.50 (m, 2H), 1.18 – 1.04 (m, 2H), 0.92 (t, *J* = 7.1 Hz, 6H), 0.79 (d, *J* = 6.9 Hz, 3H). **^{13}C NMR** (101 MHz, CDCl$_3$) δ 165.3, 137.3 (d, *J* = 101.0 Hz), 133.9 (d, *J* = 2.8 Hz), 132.2 (d, *J* = 2.8 Hz), 132.1 (d, *J* = 10.1 Hz), 132.1 (d, *J* = 10.0 Hz), 131.9 (d, *J* = 104.7 Hz), 129.4 (d, *J* = 12.2 Hz), 128.7 (d, *J* = 12.2 Hz), 75.5, 47.2, 40.9, 34.2, 31.5, 26.5, 23.6, 22.0, 20.8, 16.5. **^{31}P NMR** (162 MHz, CDCl$_3$) δ 28.6. **HRMS (ESI)** m/z calcd. For $C_{29}H_{34}O_3P$ [M+H]$^+$ 461.2240, found 461.2235.

(1*R*,2*S*,5*R*)-2-isopropyl-5-methylcyclohexyl 4-((3-methoxy-3-oxopropyl)thio) benzoate (104)

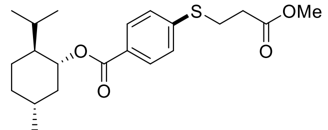

104

According to **GP5** starting from (1*R*,2*S*,5*R*)-2-isopropyl-5-methylcyclohexyl-4-iodobenzoate (0.2 mmol, 1.0 eq.), methyl 3-mercaptopropanoate (0.3 mmol, 1.5 eq.), ^{i}Pr$_2$NH (0.6 mmol, 3.0 eq.) and bpyNi-PHI (10 mg, 6.0 wt % Nibpy) in MeCN (2 mL) for 12 h, the product **104** was isolated as colorless oil after flash chromatography (petroleum ether/ethyl acetate 2/1), 70.4 mg (93% yield). **^{1}H NMR** (400 MHz, CDCl$_3$) δ 7.99 – 7.91 (m, 2H), 7.36 – 7.28 (m, 2H), 4.91 (td, *J* = 10.9, 4.4 Hz, 1H), 3.70 (s, 3H), 3.25 (t, *J* = 7.4 Hz, 2H), 2.68 (t, *J* = 7.4 Hz, 2H), 2.15 – 2.07 (m, 1H), 1.90 – 1.89 (m, 1H), 1.78 – 1.68 (m, 2H), 1.62 – 1.48 (m, 2H), 1.19 – 1.06 (m, 2H), 0.97 – 0.87 (m, 7H), 0.79 (d, *J* = 7.0 Hz, 3H). **^{13}C NMR** (101 MHz, CDCl$_3$) δ 171.9, 165.7, 142.2, 130.1, 128.1, 127.3, 74.9, 52.0, 47.3, 41.0, 34.3, 33.8, 31.5, 27.4, 26.5, 23.7, 22.1, 20.8, 16.6. **HRMS (ESI)** m/z calcd. For $C_{21}H_{31}O_4S$ [M+H]$^+$ 379.1938, found 379.1935.

(*S*)-4-(2-acetamido-3-methoxy-3-oxopropyl)phenyl 4-(diphenylphosphoryl) benzoate (105)

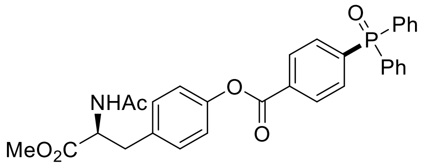

105

According to **GP1** starting from (*S*)-4-(2-acetamido-3-methoxy-3-oxopropyl)phenyl 4-iodobenzoate (0.2 mmol, 1.0 eq.), diphenylphosphine oxide (0.3 mmol, 1.5 eq.), ^{i}Pr$_2$NH (0.6 mmol, 3.0 eq.) and bpyNi-PHI (10 mg, 6.0 wt % Nibpy) in DMF (2 mL) for 36 h, the product **105** was isolated as yellow oil after flash chromatography (petroleum ether/ethyl acetate 1/1), 57.0 mg (75% yield). **^{1}H NMR** (400 MHz, CDCl$_3$) δ 8.30 – 8.23 (m, 2H), 7.84 (dd, *J* = 11.4, 8.1 Hz, 2H), 7.74 – 7.64 (m, 4H), 7.63 – 7.54 (m,

2H), 7.53 – 7.46 (m, 4H), 7.19 – 7.11 (m, 4H), 6.02 (d, *J* = 7.8 Hz, 1H), 4.94 – 4.87 (m, 1H), 3.74 (s, 3H), 3.23 – 3.09 (m, 2H), 2.01 (s, 3H). **^{13}C NMR** (101 MHz, $CDCl_3$) δ 172.0, 169.7, 164.3, 149.8, 138.6 (d, *J* = 100.4 Hz), 133.9, 132.5 (d, *J* = 2.9 Hz), 132.4 (d, *J* = 6.9 Hz), 132.3 (d, *J* = 6.0 Hz), 132.1 (d, *J* = 9.9 Hz), 131.7 (d, *J* = 104.9 Hz), 130.0, 129.9 (d, *J* = 12.1 Hz), 128.7 (d, *J* = 12.1 Hz), 121.7, 53.1, 52.4, 37.3, 23.2. **^{31}P NMR** (162 MHz, $CDCl_3$) δ 28.3. **HRMS (ESI)** m/z calcd. For $C_{31}H_{29}NO_6P$ $[M+H]^+$ 542.1727, found 542.1726.

Methyl 4-(((*tert*-butoxycarbonyl)-*L*-alanyl)oxy)benzoate[56] (106)

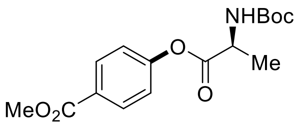

106

According to **GP4** starting from methyl 4-iodobenzoate (0.2 mmol, 1.0 eq.), (*tert*-butoxycarbonyl)-*L*-alanine (0.3 mmol, 1.5 eq.), BIPA (0.6 mmol, 3.0 eq.) and bpyNi-PHI (10 mg, 6.0 wt % Nibpy) in DMSO (2 mL) for 16 h, the product **106** was isolated as white solid after flash chromatography (petroleum ether/ethyl acetate 10/1), 40.1 mg (62% yield). **^{1}H NMR** (400 MHz, $CDCl_3$) δ 8.08 (d, *J* = 8.7 Hz, 2H), 7.19 (d, *J* = 8.7 Hz, 2H), 5.13 – 5.01 (m, 1H), 4.63 – 4.45 (m, 1H), 3.92 (s, 3H), 1.56 (d, *J* = 7.2 Hz, 3H), 1.47 (s, 9H). **^{13}C NMR** (101 MHz, $CDCl_3$) δ 171.6, 166.2, 155.2, 154.1, 131.2, 128.0, 121.4, 80.3, 52.2, 49.5, 28.3, 18.3.

Methyl 4-(((*tert*-butoxycarbonyl)-*L*-phenylalanyl)oxy)benzoate[56] (107)

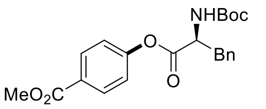

107

According to **GP4** starting from methyl 4-iodobenzoate (0.2 mmol, 1.0 eq.), (*tert*-butoxycarbonyl)-*L*-phenylalanine (0.3 mmol, 1.5 eq.), BIPA (0.6 mmol, 3.0 eq.) and bpyNi-PHI (10 mg, 6.0 wt % Nibpy) in DMSO (2 mL) for 16 h, the product **107** was isolated as white solid after flash chromatography (petroleum ether/ethyl acetate 10/1), 60.0 mg (75% yield). **^{1}H NMR** (400 MHz, $CDCl_3$) δ 8.05 (d, *J* = 8.7 Hz, 2H), 7.37 – 7.26 (m, 3H), 7.26 – 7.20 (m, 2H), 7.06 (d, *J* = 8.7 Hz, 2H), 5.13 (d, *J* = 8.2 Hz, 1H), 4.81 (q, *J* = 6.9 Hz, 1H), 3.90 (s, 3H), 3.23 (d, *J* = 6.4 Hz, 2H), 1.44 (s, 9H). **^{13}C NMR** (101 MHz, $CDCl_3$) δ 170.2, 166.2, 155.2, 153.9, 135.6, 131.2, 129.4, 128.8, 128.0, 127.4, 121.4, 80.3, 54.8, 52.2, 38.3, 28.3.

1-(*tert*-butyl) 2-(4-(methoxycarbonyl)phenyl) (*S*)-pyrrolidine-1,2-dicarboxylate[17] (108)

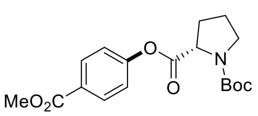

108

According to **GP4** starting from methyl 4-iodobenzoate (0.2 mmol, 1.0 eq.), (*tert*-butoxycarbonyl)-*L*-proline (0.3 mmol, 1.5 eq.), BIPA (0.6 mmol, 3.0 eq.) and bpyNi-PHI (10 mg, 6.0 wt % Nibpy) in DMSO (2 mL) for 16 h, the product **108** was isolated as pale white solid after flash chromatography (petroleum ether/ethyl acetate 10/1), 52.4 mg (75% yield). **^{1}H NMR** (400 MHz, $CDCl_3$) rotameric mixture, δ 8.13 – 8.02 (m, 2H), 7.19 (t, *J* = 9.3 Hz, 2H), 4.53 (dd, *J* = 8.6, 4.3 Hz, 0.44H), 4.46 (dd, *J* = 8.7, 4.4 Hz, 0.56H), 3.95 – 3.88 (m, 3H), 3.68 – 3.41 (m, 2H), 2.47 – 2.30 (m, 1H), 2.23 – 2.11 (m, 1H), 2.11 – 1.91 (m, 1H), 1.51 – 1.43 (m, 9H). **^{13}C NMR** (101 MHz, $CDCl_3$) rotameric mixture, resonances for minor rotamer are enclosed in parenthesis δ (171.2) 171.1, (166.3) 166.2, (154.5) 154.2, 153.7, 131.2 (131.1), 127.8 (127.7), (121.5), 121.1, 80.3 (80.1), 59.2 (59.1), 52.2 (52.2), (46.6) 46.5, 31.0 (30.0), 28.4, (24.5) 23.7.

Methyl (*S*)-4-(3-(*tert*-butoxy)-2-((*tert*-butoxycarbonyl)amino)-3-oxopropoxy) benzoate (109)

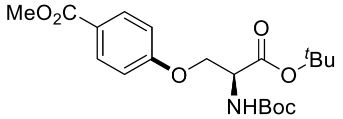

109

According to **GP3** starting from methyl 4-iodobenzoate (0.2 mmol, 1.0 eq.), *tert*-butyl (*tert*-butoxycarbonyl)-*L*-serinate (0.3 mmol, 1.5 eq.), TMG (0.4 mmol, 2.0 eq.) and bpyNi-PHI (10 mg, 6.0 wt % Nibpy) in MeCN (2 mL) for 12 h, the product **109** was isolated as white solid after flash chromatography (petroleum ether/ethyl acetate 5/1), 50.6 mg (64% yield). mp = 112–113 °C. **^{1}H NMR** (400 MHz, $CDCl_3$) δ 8.06 – 7.92 (m, 2H), 6.95 – 6.86 (m, 2H), 5.48 (d, *J* = 8.2 Hz, 1H), 4.98 – 4.61 (m, 1H), 4.39 (dd, *J* = 9.2, 3.1 Hz, 1H), 4.27 (dd, *J* = 9.2, 2.9 Hz, 1H), 3.89 (s, 3H), 1.45 (d, *J* = 7.8 Hz, 18H). **^{13}C NMR** (101 MHz, $CDCl_3$) δ 168.7, 166.7, 162.1, 155.3, 131.6, 123.2, 114.1, 82.8, 80.1, 68.9, 53.9, 51.9, 28.3, 28.0. **HRMS (ESI)** m/z calcd. For $C_{20}H_{30}NO_7$ $[M+H]^+$ 396.2017, found 396.2041.

Methyl (*R*)-4-((2-((*tert*-butoxycarbonyl)amino)-3-methoxy-3-oxopropyl)thio) benzoate (110)

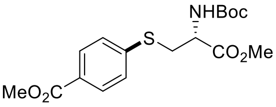

110

According to **GP5** starting from methyl 4-iodobenzoate (0.2 mmol, 1.0 eq.), methyl (*tert*-butoxycarbonyl)-*L*-cysteinate (0.3 mmol, 1.5 eq.), ^{i}Pr$_2$NH (0.6 mmol, 3.0 eq.) and bpyNi-PHI (10 mg, 6.0 wt % Nibpy) in MeCN (2 mL) for 12 h, the product **110** was isolated as colorless oil after flash chromatography (petroleum ether/ethyl acetate 5/1), 48.0 mg (65% yield). **^{1}H NMR** (400 MHz, $CDCl_3$) δ 7.93 (d, *J* = 8.5 Hz, 2H), 7.38 (d, *J* = 8.5 Hz, 2H), 5.36 (d, *J* = 8.0 Hz, 1H), 4.71 – 4.55 (m, 1H), 3.90 (s, 3H), 3.62 (s, 3H), 3.54 – 3.36 (m, 2H), 1.42 (s, 9H). **^{13}C NMR** (101 MHz, $CDCl_3$) δ 170.8, 166.6, 154.9, 142.0, 130.2, 130.0, 128.3, 127.8, 126.2, 80.3, 53.2, 52.6, 52.1, 41.1, 35.6, 28.3. **HRMS (ESI)** m/z calcd. For $C_{17}H_{22}NO_6S$ $[M-H]^-$ 368.1168, found 368.1174.

methyl 4-(((3a*R*,5*R*,5a*S*,8a*S*,8b*R*)-2,2,7,7-tetramethyltetrahydro-5*H*-bis([1,3] dioxolo)[4,5-*b*:4',5'-*d*]pyran-5-yl)methoxy)benzoate (111)

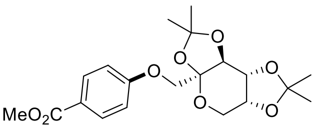

111

According to **GP3** starting from methyl 4-iodobenzoate (0.2 mmol, 1.0 eq.), ((3a*S*,5a*R*,8a*R*,8b*S*)-2,2,7,7-tetramethyltetrahydro-3a*H*-bis([1,3]dioxolo)[4,5-*b*:4',5'-*d*] pyran-3a-yl)methanol (0.3 mmol, 1.5 eq.), TMG (0.4 mmol, 2.0 eq.) and bpyNi-PHI (10 mg, 6.0 wt % Nibpy) in MeCN (2 mL) for 12 h, the product **111** was isolated as yellow oil after flash chromatography (petroleum ether/ethyl acetate 5/1), 51.3 mg (65% yield). mp = 96–97 °C. **^{1}H NMR** (400 MHz, $CDCl_3$) δ 8.04 – 7.93 (m, 2H), 7.01 – 6.86 (m, 2H), 4.65 (dd, *J* = 7.9, 2.6 Hz, 1H), 4.53 (d, *J* = 2.6 Hz, 1H), 4.28 (dd, *J* = 7.7, 1.7 Hz, 1H), 4.22 (d, *J* = 10.3 Hz, 1H), 4.09 (d, *J* = 10.3 Hz, 1H), 3.98 (dd, *J* = 13.0, 1.9 Hz, 1H), 3.88 (s, 3H), 3.80 (d, *J* = 13.0 Hz, 1H), 1.57 (s, 3H), 1.49 (s, 3H), 1.47 (s, 3H), 1.35 (s, 3H). **^{13}C NMR** (101 MHz, $CDCl_3$) δ 166.7, 162.2, 131.6, 123.1, 114.3, 109.0, 101.9, 70.9, 70.1, 70.0, 68.8, 61.2, 51.9, 26.6, 25.9, 25.3, 24.0. **HRMS (ESI)** m/z calcd. For $C_{20}H_{27}O_8$ $[M+H]^+$ 395.1700, found 395.1698.

Methyl 4-((2*S*)-2-((5*S*,6a*S*)-6-(benzyloxy)-2,2-dimethyltetrahydrofuro[2,3-*d*][1,3]dioxol-5-yl)-2-hydroxyethoxy)benzoate (112)

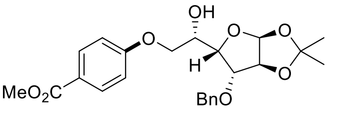

112

According to **GP3** starting from methyl 4-iodobenzoate (0.2 mmol, 1.0 eq.), (*S*)-1-((3a*S*,5*S*,6*R*,6a*S*)-6-(benzyloxy)-2,2-dimethyltetrahydrofuro[2,3-*d*][1,3]dioxol-5-yl)ethane-1,2-diol (0.3 mmol, 1.5 eq.), TMG (0.4 mmol, 2.0 eq.) and bpyNi-PHI (10 mg, 6.0 wt % Nibpy) in MeCN (2 mL) for 12 h, the product **112** was isolated as colorless oil after flash chromatography (petroleum ether/ethyl acetate 2/1), 55.1 mg (62% yield). **^{1}H NMR** (400 MHz, $CDCl_3$) δ 7.97 – 7.89 (m, 2H), 7.25 – 7.10 (m, 3H), 6.99 – 6.91 (m, 4H), 5.94 (d, *J* = 3.7 Hz, 1H), 4.86 – 4.79 (m, 1H), 4.62 (d, *J* = 3.7 Hz, 1H), 4.52 – 4.43 (m, 2H), 4.16 (d, *J* = 11.3 Hz, 1H), 4.06 – 4.00 (m, 2H), 3.96 – 3.90 (m, 1H), 3.90 (s, 3H), 1.53 (s, 3H), 1.34 (s, 3H). **^{13}C NMR** (101 MHz, $CDCl_3$) δ 166.7, 161.4, 136.6, 131.7, 128.3, 128.0, 127.9, 123.3, 115.4, 112.2, 105.3, 81.7, 81.2, 79.5, 74.4, 72.1, 62.3, 51.9, 26.9, 26.3. **HRMS (ESI)** m/z calcd. For $C_{20}H_{29}O_8$ $[M+H]^+$ 445.1857, found 445.1853.

Methyl 4-(((8*R*,9*S*,13*S*,14*S*)-13-methyl-17-oxo-7,8,9,11,12,13,14,15,16,17-decahydro-6H-cyclopenta[*a*]phenanthren-2-yl)oxy)benzoate[55] (113)

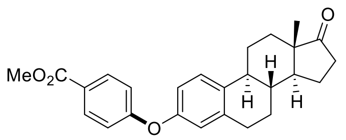

113

According to **GP3** starting from methyl 4-iodobenzoate (0.2 mmol, 1.0 eq.), (8*R*,9*S*,13*S*,14*S*)-3-hydroxy-13-methyl-6,7,8,9,11,12,13,14,15,16-decahydro-17*H*-cyclopenta[*a*]phenanthren-17-one (0.3 mmol, 1.5 eq.), TMG (0.4 mmol, 2.0 eq.) and bpyNi-PHI (10 mg, 6.0 wt % Nibpy) in MeCN (2 mL) for 72 h, the product **113** was isolated as white solid after flash chromatography (petroleum ether/ethyl acetate 5/1), 44.5 mg (55% yield). **^{1}H NMR** (400 MHz, $CDCl_3$) δ 7.99 (d, *J* = 8.8 Hz, 2H), 7.29 (d, *J* = 8.6 Hz, 1H), 6.98 (d, *J* = 8.8 Hz, 2H), 6.87 – 6.82 (m, 1H), 6.81 – 6.77 (m, 1H), 3.90 (s, 3H), 2.93 – 2.85 (m, 2H), 2.52 (dd, *J* = 18.9, 8.6 Hz, 1H), 2.46 – 2.40 (m, 1H), 2.35 – 2.25 (m, 1H), 2.23 – 1.95 (m, 4H), 1.70 – 1.62 (m, 2H), 1.58 – 1.42 (m, 4H), 0.94 (s, 3H). **^{13}C NMR** (101 MHz, $CDCl_3$) δ 166.7, 162.1, 153.4, 138.6, 136.1, 131.6, 126.9, 124.2, 120.2, 117.5, 117.1, 52.0, 50.5, 48.0, 44.2, 38.2, 35.9, 31.6, 29.5, 26.4, 25.9, 21.6, 13.9.

Methyl (*S*)-4-(1-phenylethoxy)benzoate[16] (114)

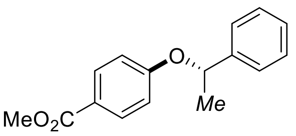

114

According to **GP3** starting from methyl 4-iodobenzoate (0.2 mmol, 1.0 eq.), (*S*)-1-phenylethan-1-ol (0.6 mmol, 3.0 eq.), TMG (0.4 mmol, 2.0 eq.) and bpyNi-PHI (10 mg, 6.0 wt % Nibpy) in MeCN (2 mL) for 12 h, the product **114** was isolated as colorless oil after flash chromatography (petroleum ether/ethyl acetate 10/1), 33.8 mg (66% yield). **^{1}H NMR** (400 MHz, $CDCl_3$) δ 7.89 (d, *J* = 8.5 Hz, 2H), 7.37 – 7.30 (m, 4H), 7.27 – 7.23 (m, 1H), 6.86 (d, *J* = 8.7 Hz, 2H), 5.37 (q, *J* = 6.4 Hz, 1H), 3.84 (s, 3H), 1.65 (d, *J* = 6.8 Hz, 3H). **^{13}C NMR** (101 MHz, $CDCl_3$) δ 165.8, 160.7, 141.4, 130.4, 127.7, 126.7, 124.4, 121.4, 114.4, 75.1, 50.8, 23.4.

Diethyl (4-methoxyphenyl)phosphonate[14] (115)

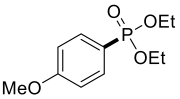

115

According to **GP1** starting from 1-iodo-4-methoxybenzene (0.2 mmol, 1.0 eq.), diethyl phosphonate (0.3 mmol, 1.5 eq.), $^{i}Pr_2NH$ (0.6 mmol, 3.0 eq.) and bpyNi-PHI (10 mg, 6.0 wt % Nibpy) in DMF (2 mL) for 60 h, the product **115** was isolated as colorless oil after flash chromatography (petroleum ether/ethyl acetate 1/1), 42.9 mg (88% yield). **^{1}H NMR** (400 MHz, $CDCl_3$) δ 7.86 – 7.65 (m, 2H), 6.97 (dd, *J* = 8.7, 3.3 Hz, 2H), 4.28 – 3.95 (m, 4H), 3.85 (s, 3H), 1.31 (t, *J* = 7.1 Hz, 6H). **^{13}C NMR** (101 MHz, $CDCl_3$) δ 162.8 (d, *J* = 3.2 Hz), 133.8 (d, *J* = 11.3 Hz), 119.5 (d, *J* = 194.8 Hz), 114.0 (d, *J* = 16.0 Hz), 61.9 (d, *J* = 5.2 Hz), 55.3, 16.3 (d, *J* = 6.5 Hz). **^{31}P NMR** (162 MHz, $CDCl_3$) δ 19.7.

***N*-phenyl-4-(5-(*p*-tolyl)-3-(trifluoromethyl)-1H-pyrazol-1-yl)benzenesulfonamide (116)**

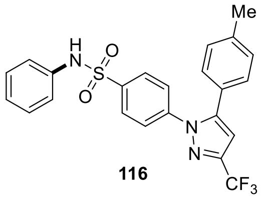

116

According to **GP2** starting from iodobenzene (0.2 mmol, 1.0 eq.), 4-(5-(*p*-tolyl)-3-(trifluoromethyl)-1*H*-pyrazol-1-yl) benzenesulfonamide (0.3 mmol, 1.5 eq.), TMG (0.4 mmol, 2.0 eq.) and bpyNi-PHI (10 mg, 6.0 wt % Nibpy) in MeCN (2 mL) for 12 h, the product **116** was isolated as yellow solid after flash chromatography (petroleum ether/ethyl acetate 5/1), 82.3 mg (90% yield). mp 78–79 °C. **^{1}H NMR** (400 MHz, $CDCl_3$) δ 7.73 (d, *J* = 8.7 Hz, 2H), 7.37 (d, *J* = 8.7 Hz, 2H), 7.25 – 7.21 (m, 2H), 7.16 – 7.10 (m, 4H), 7.08 – 7.00 (m, 4H), 6.71 (s, 1H), 2.36 (s, 3H). **^{13}C NMR** (101 MHz, $CDCl_3$) δ 145.3, 144.1 (q, *J* = 38.6 Hz), 142.7, 139.8, 138.3, 136.0, 129.7, 129.5, 128.7, 128.3, 125.9, 125.6, 125.5, 122.2, 121.1 (q, *J* = 269.2 Hz), 106.3 (q, *J* = 2.1 Hz), 21.3. **^{19}F NMR** (377 MHz, $CDCl_3$) δ -62.4. **HRMS (ESI)** m/z calcd. For $C_{23}H_{19}F_3N_3O_2S$ $[M+H]^+$ 458.1145, found 458.1141.

4-(methoxycarbonyl)phenyl 4-(*N*,*N*-dipropylsulfamoyl)benzoate (117)

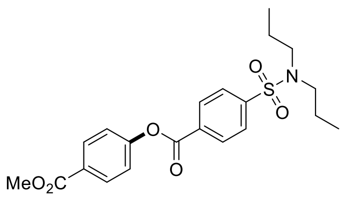

117

According to **GP4** starting from methyl 4-iodobenzoate (0.2 mmol, 1.0 eq.), 4-(*N*,*N*-dipropylsulfamoyl)benzoic acid (0.3 mmol, 1.5 eq.), BIPA (0.6 mmol, 3.0 eq.) and bpyNi-PHI (10 mg, 6.0 wt % Nibpy) in DMSO (2 mL) for 12 h, the product **117** was isolated as yellow solid after flash chromatography (petroleum ether/ethyl acetate 5/1), 65.4 mg (78% yield). mp 70–71 °C. **^{1}H NMR** (400 MHz, $CDCl_3$) δ 8.32 (d, *J* = 8.4 Hz, 2H), 8.14 (d, *J* = 8.7 Hz, 2H), 7.96 (d, *J* = 8.5 Hz, 2H), 7.32 (d, *J* = 8.7 Hz, 2H), 3.94 (s, 3H), 3.21 – 3.07 (m, 4H), 1.62 – 1.52 (m, 4H), 0.89 (t, *J* = 7.4 Hz, 6H). **^{13}C NMR** (101 MHz, $CDCl_3$) δ 166.2, 163.3, 154.2, 145.3, 132.4, 131.3, 130.9, 128.2, 127.3, 121.6, 52.3, 49.9, 21.9, 11.2. **HRMS (ESI)** m/z calcd. For $C_{21}H_{26}NO_6S$ $[M+H]^+$ 420.1475, found 420.1474.

N-methyl-3-phenyl-3-(4-(trifluoromethyl)phenoxy)propan-1-amine[54] (118)

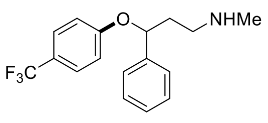

118

According to **GP3** starting from 1-iodo-4-(trifluoromethyl) benzene (0.2 mmol, 1.0 eq.), 3-(methylamino)-1-phenylpropan-1-ol (0.3 mmol, 1.5 eq.), TMG (0.4 mmol, 2.0 eq.) and bpyNi-PHI (10 mg, 6.0 wt % Nibpy) in MeCN (2 mL) for 60 h, the product **118** was isolated as colorless oil after flash chromatography (petroleum ether/ethyl acetate 5/1), 42.1 mg (68% yield). **^{1}H NMR** (400 MHz, $CDCl_3$) δ 7.44 – 7.40 (m, 2H), 7.37 – 7.33 (m, 4H), 7.32 – 7.27 (m, 1H), 6.68 (d, J = 8.6 Hz, 2H), 4.74 (t, J = 6.5 Hz, 1H), 3.53 (t, J = 6.5 Hz, 2H), 2.98 (s, 3H), 2.03 – 1.97 (m, 2H). **^{13}C NMR** (101 MHz, $CDCl_3$) δ 151.2, 144.2, 128.7, 127.9, 126.5 (q, J = 3.7 Hz), 125.7, 125.2 (q, J = 271.3 Hz), 117.4 (q, J = 32.9 Hz), 111.2, 72.4, 49.1, 38.3, 35.7. **^{19}F NMR** (377 MHz, $CDCl_3$) δ -60.8.

1.12 ^{1}H NMR spectra of $L_nNiCl_2(H_2O)_n$

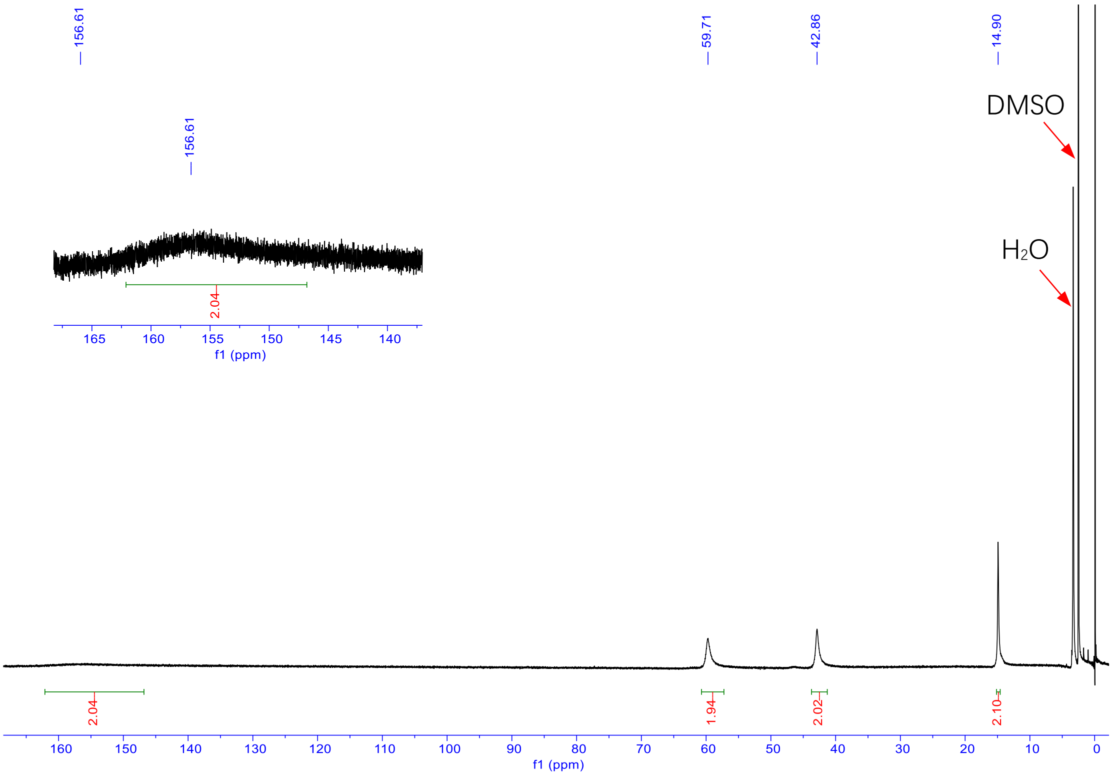

Supplementary Figure 24. ^{1}H NMR (400 MHz, DMSO) spectra of (bpy)$NiCl_2(H_2O)_n$

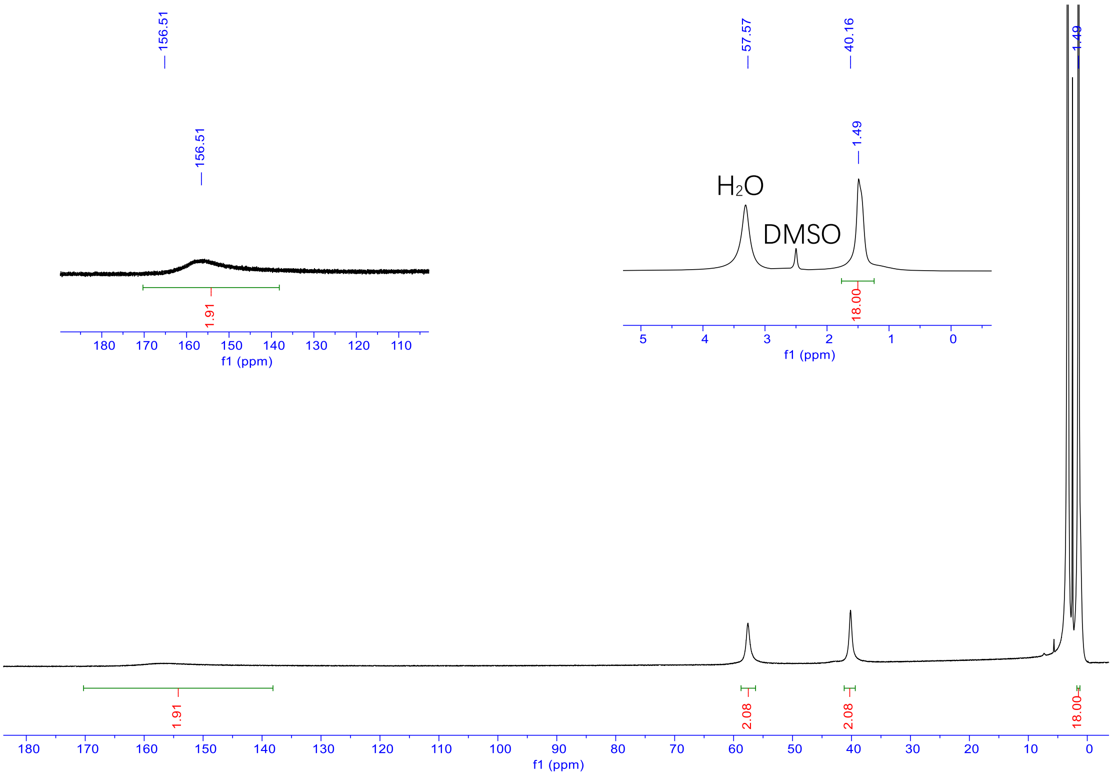

Supplementary Figure 25. ^{1}H NMR (400 MHz, DMSO) spectra of (dtbpy)$NiCl_2(H_2O)_n$

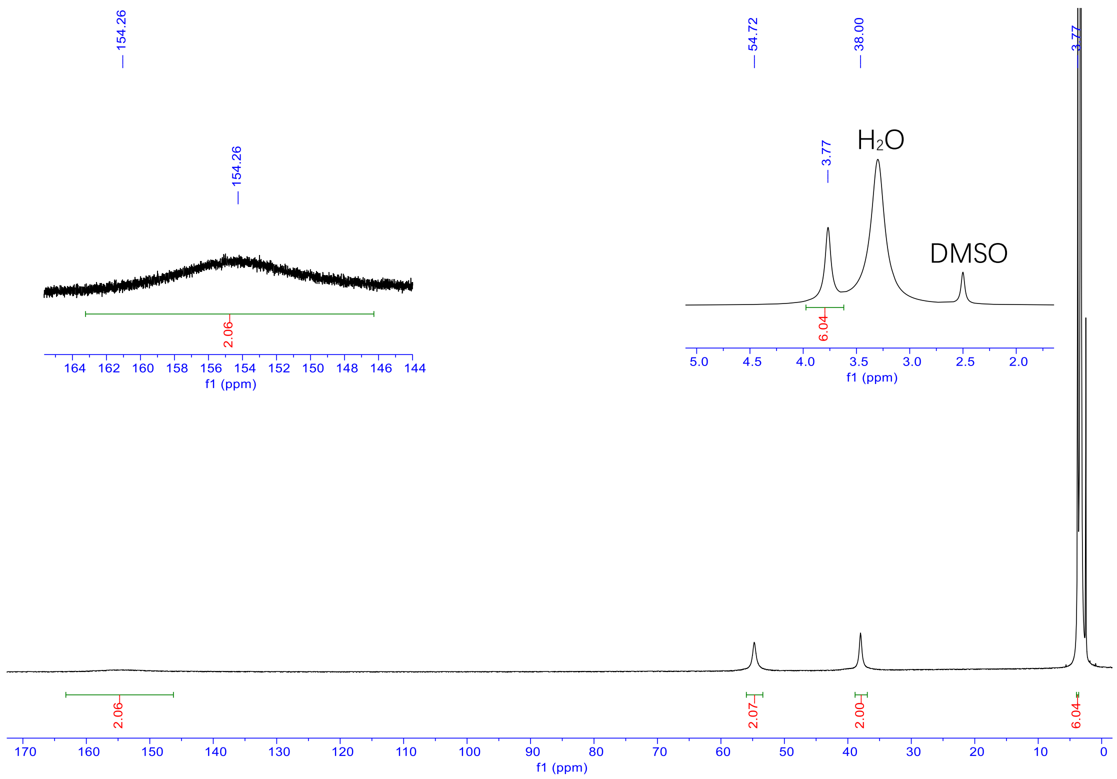

Supplementary Figure 26. ^{1}H NMR (400 MHz, DMSO) spectra of (dOMebpy)$NiCl_2(H_2O)_n$

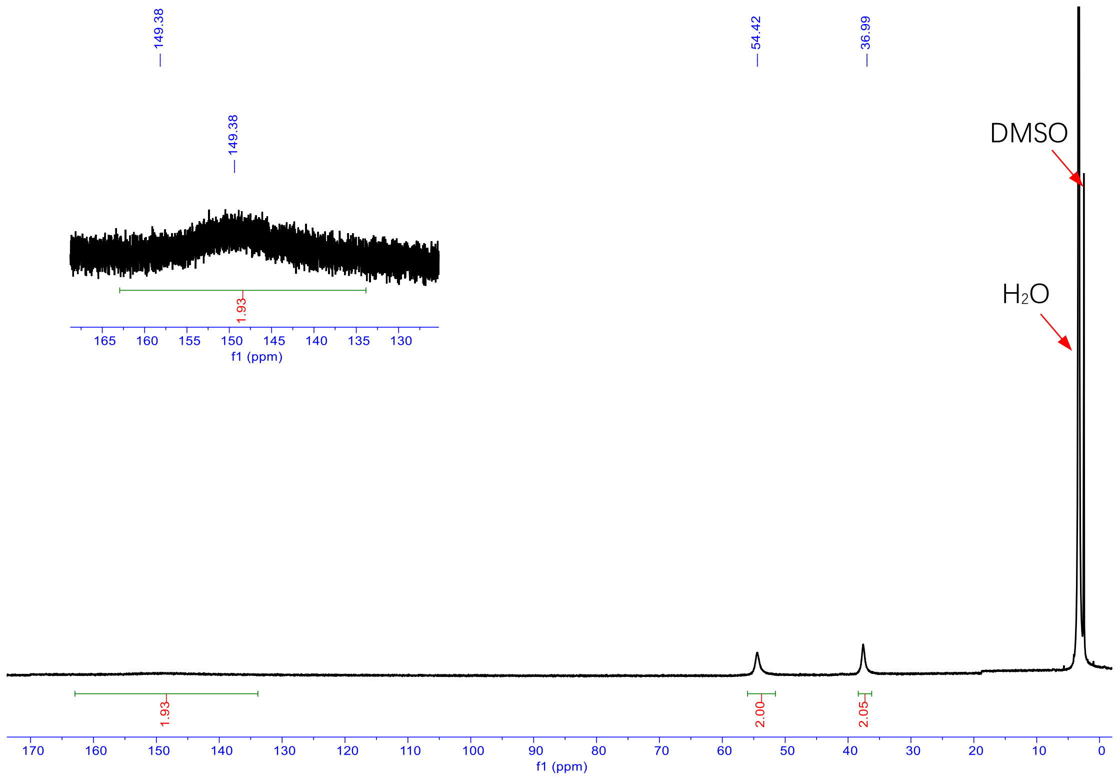

Supplementary Figure 27. ^{1}H NMR (400 MHz, DMSO) spectra of (dClbpy)$NiCl_2(H_2O)_n$

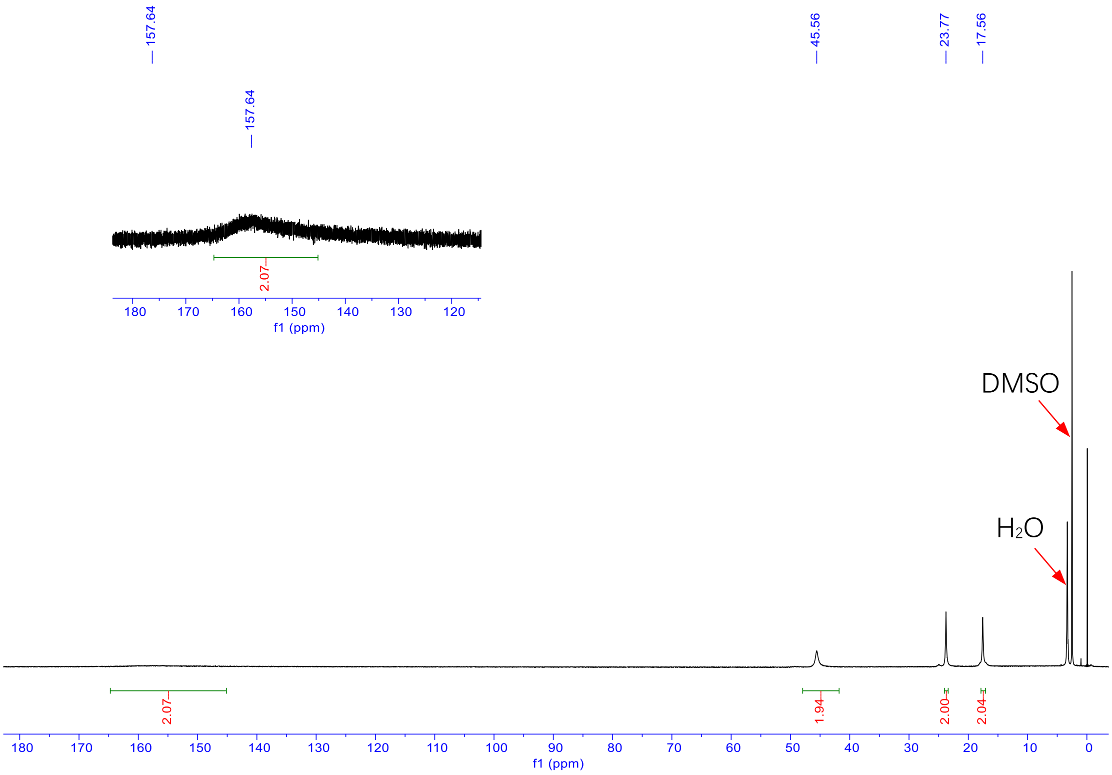

Supplementary Figure 28. ^{1}H NMR (400 MHz, DMSO) spectra of (phen)$NiCl_2(H_2O)_n$

1.13 ^{1}H NMR spectra of LₙNi in LₙNi-PHI after acid treatment

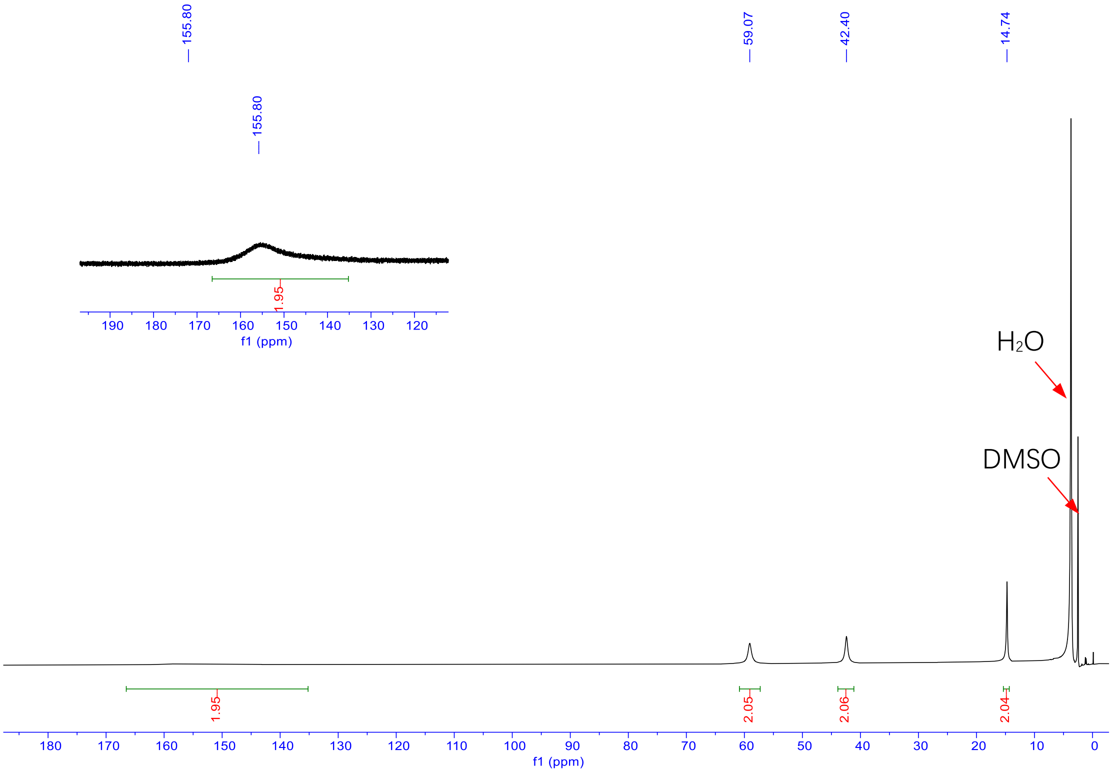

Supplementary Figure 29. ^{1}H NMR (400 MHz, DMSO) spectra of Nibpy in bpyNi-PHI

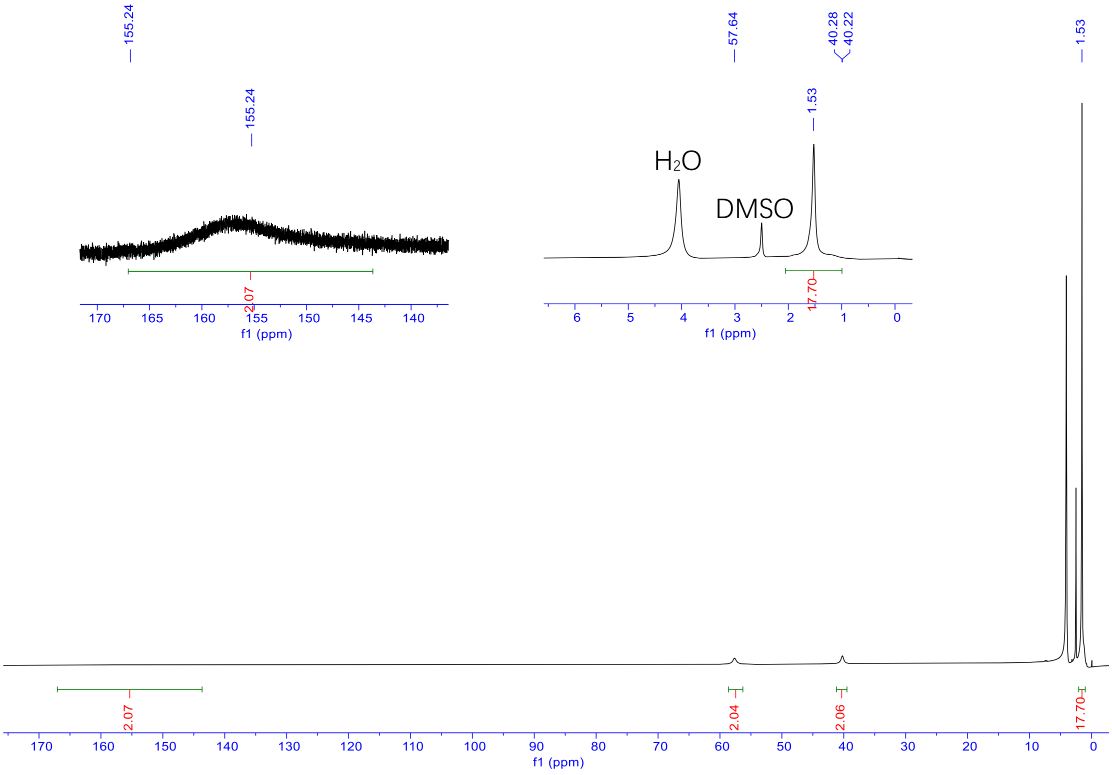

Supplementary Figure 30. ^{1}H NMR (400 MHz, DMSO) spectra of Nidtbpy in dtbpyNi-PHI

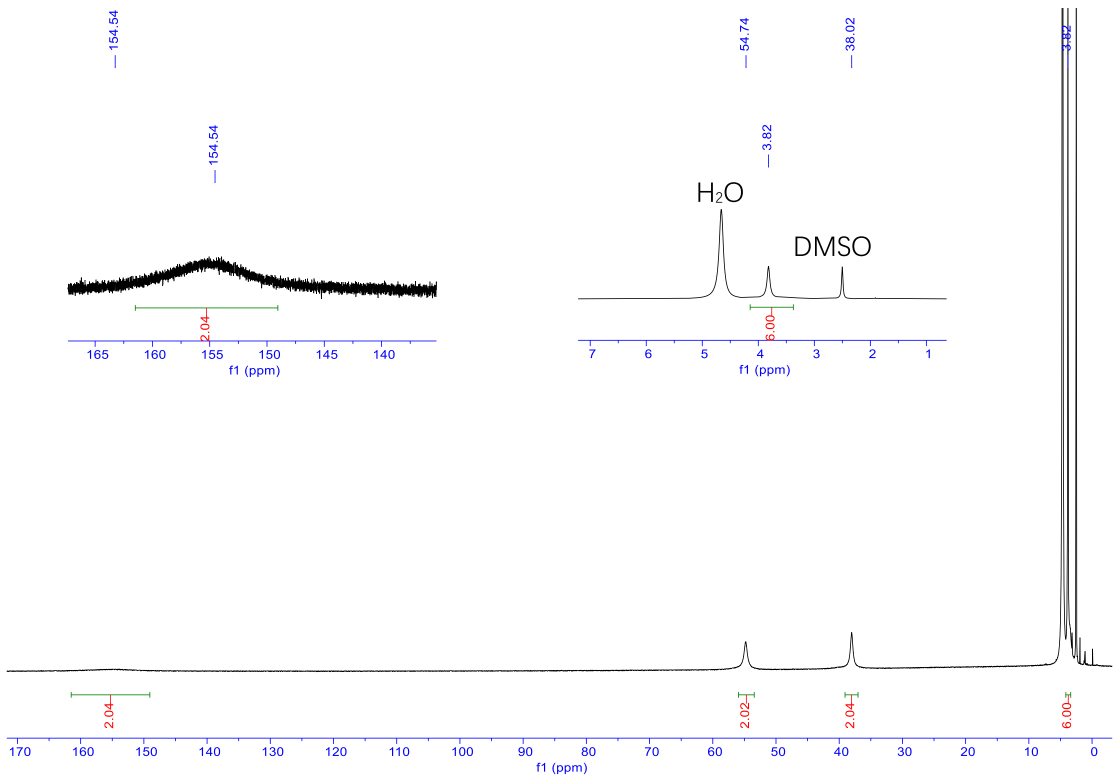

Supplementary Figure 31. ^{1}H NMR (400 MHz, DMSO) spectra of NidOMebpy in dOMebpyNi-PHI

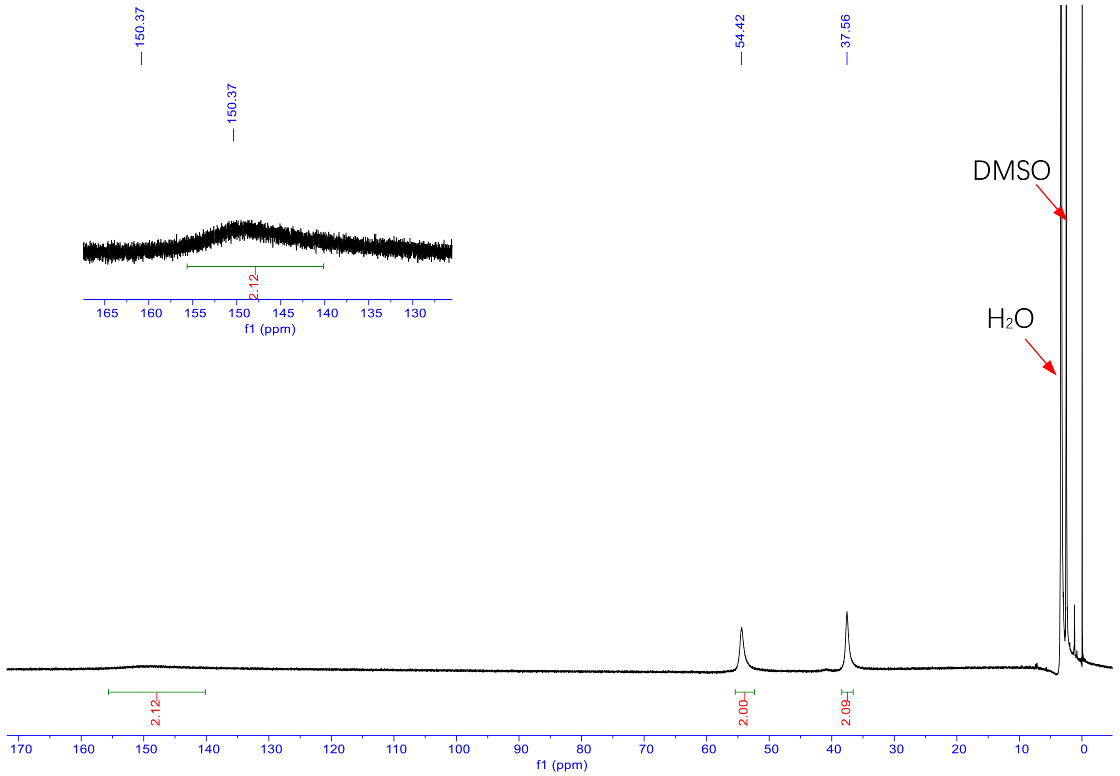

Supplementary Figure 32. ^{1}H NMR (400 MHz, DMSO) spectra of NidClbpy in dClbpyNi-PHI

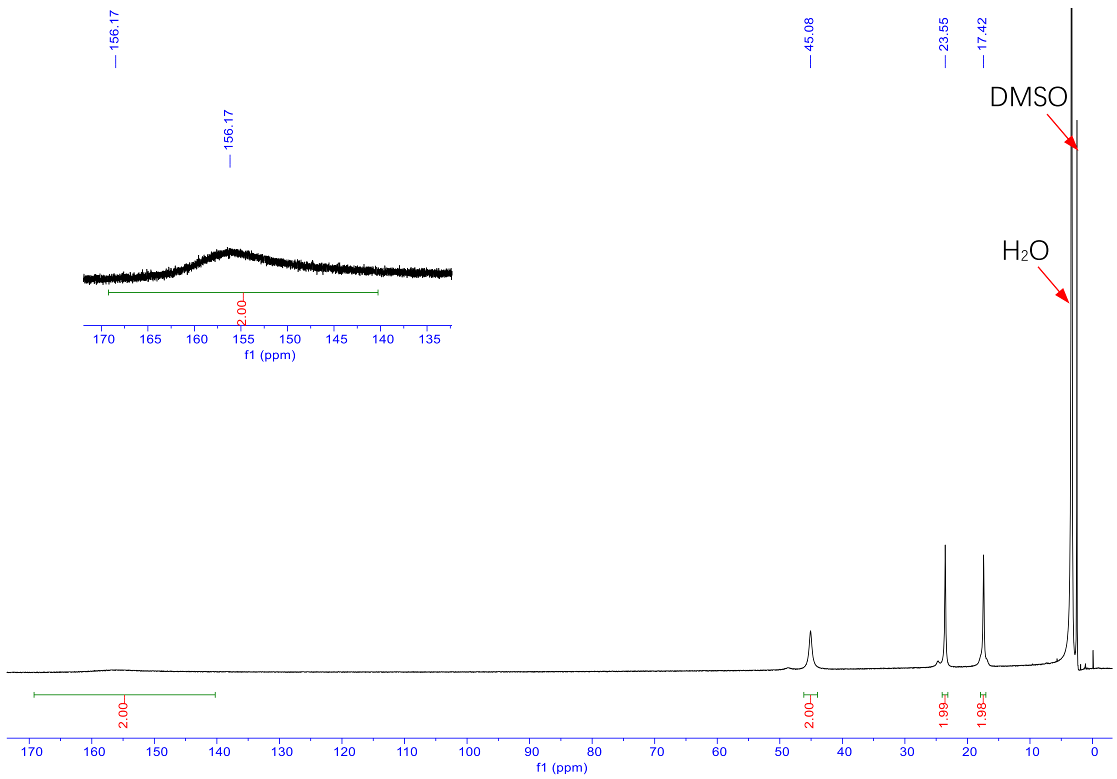

Supplementary Figure 33. ^{1}H NMR (400 MHz, DMSO) spectra of Niphen in phenNi-PHI

1.14 NMR spectra of products

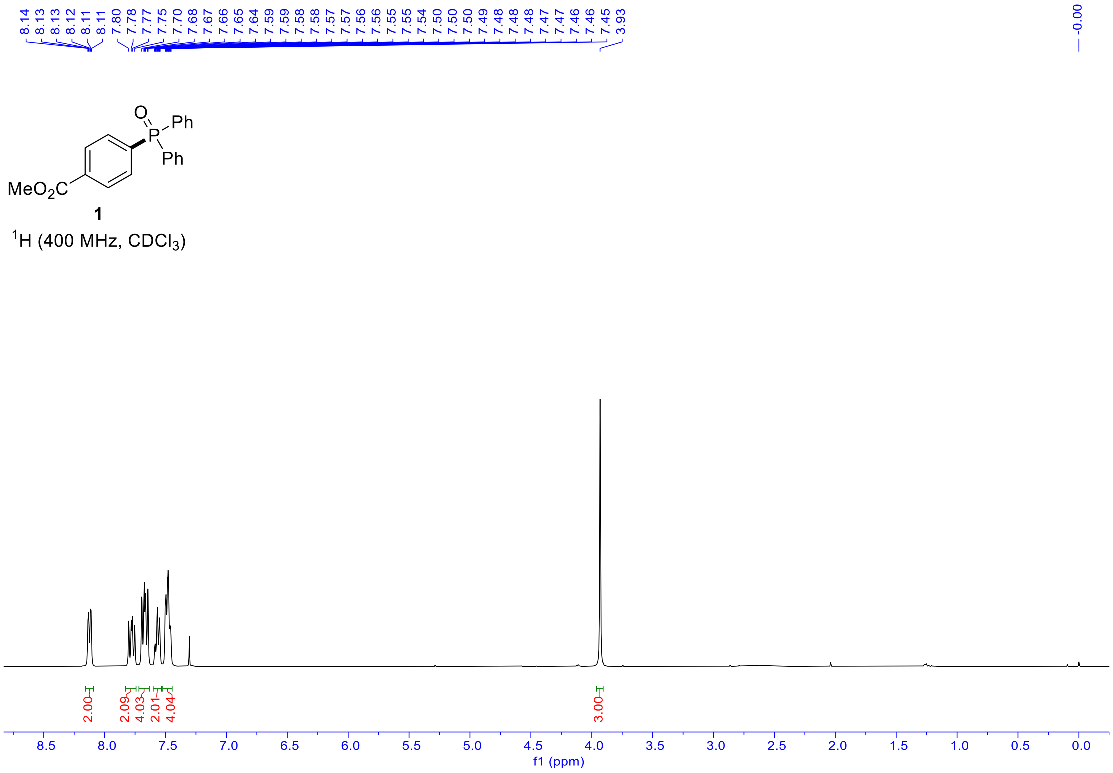

Supplementary Figure 34. ^{1}**H NMR** of compound **1**

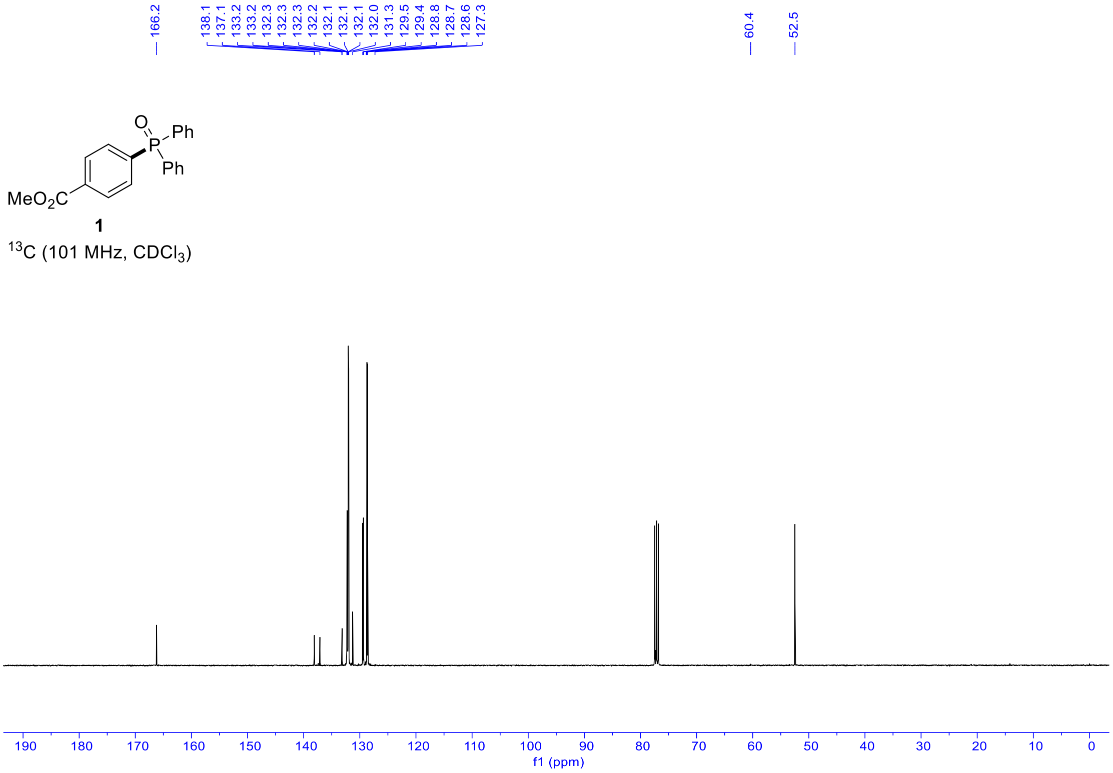

Supplementary Figure 35. ^{13}**C NMR** of compound **1**

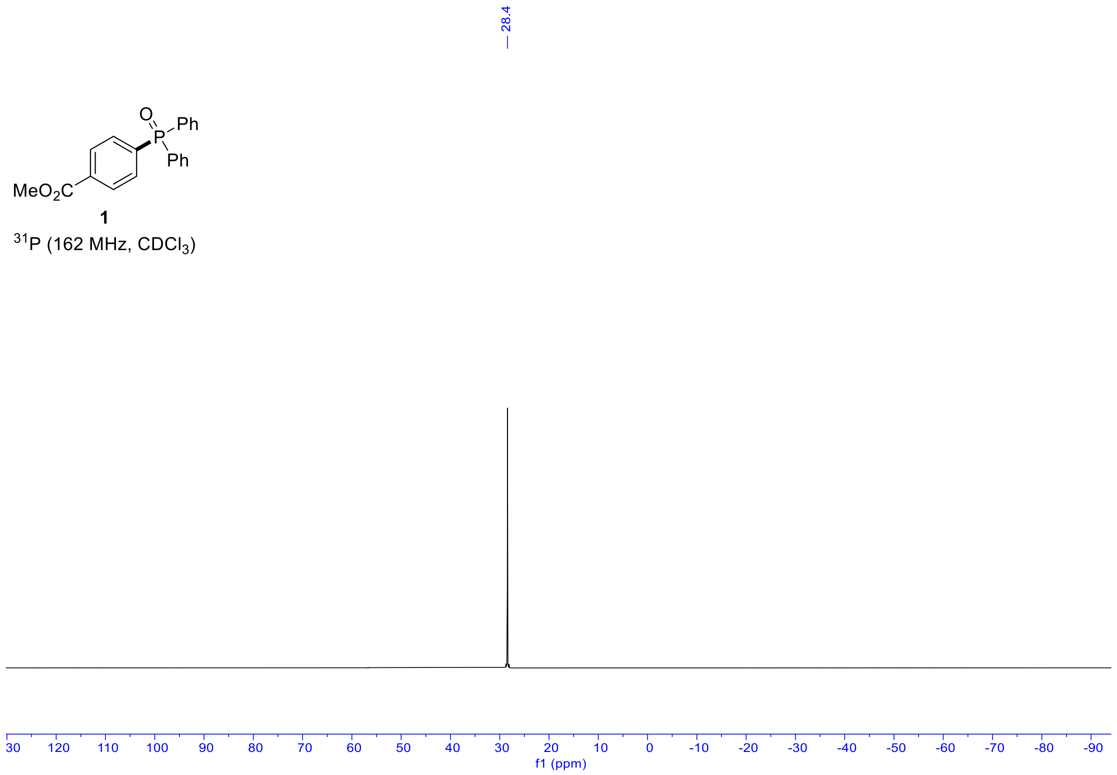

Supplementary Figure 36. ^{31}**P NMR** of compound **1**

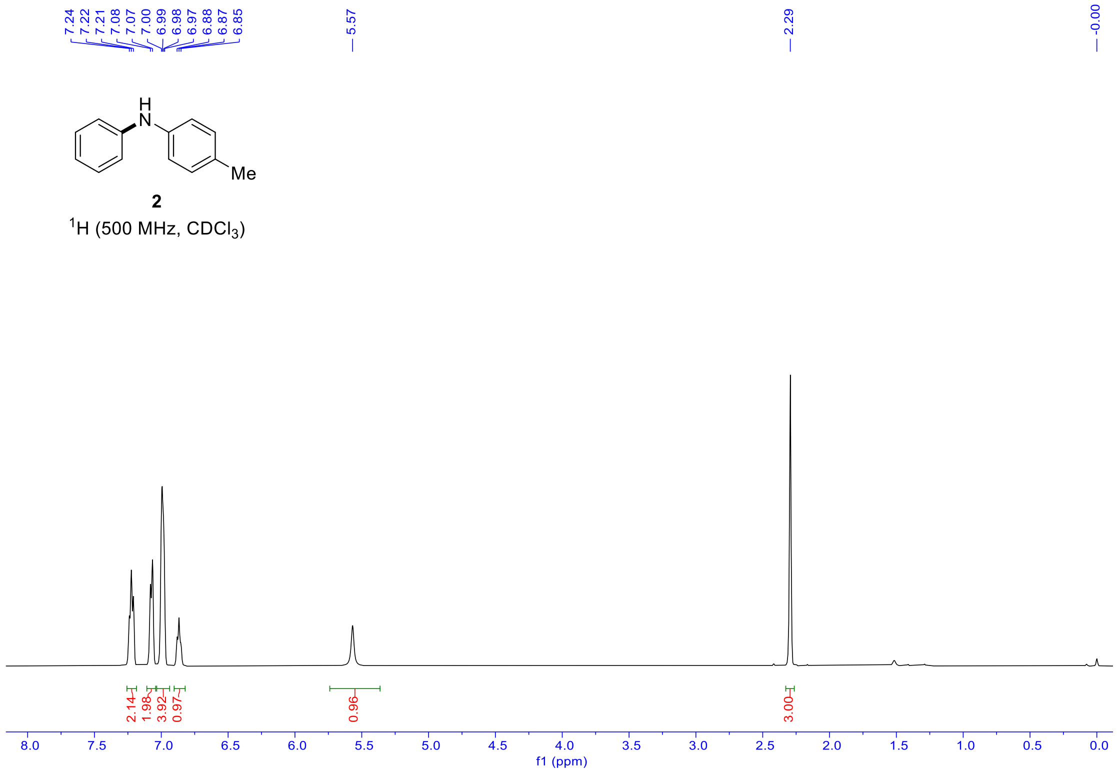

Supplementary Figure 37. ^{1}**H NMR** of compound **2**

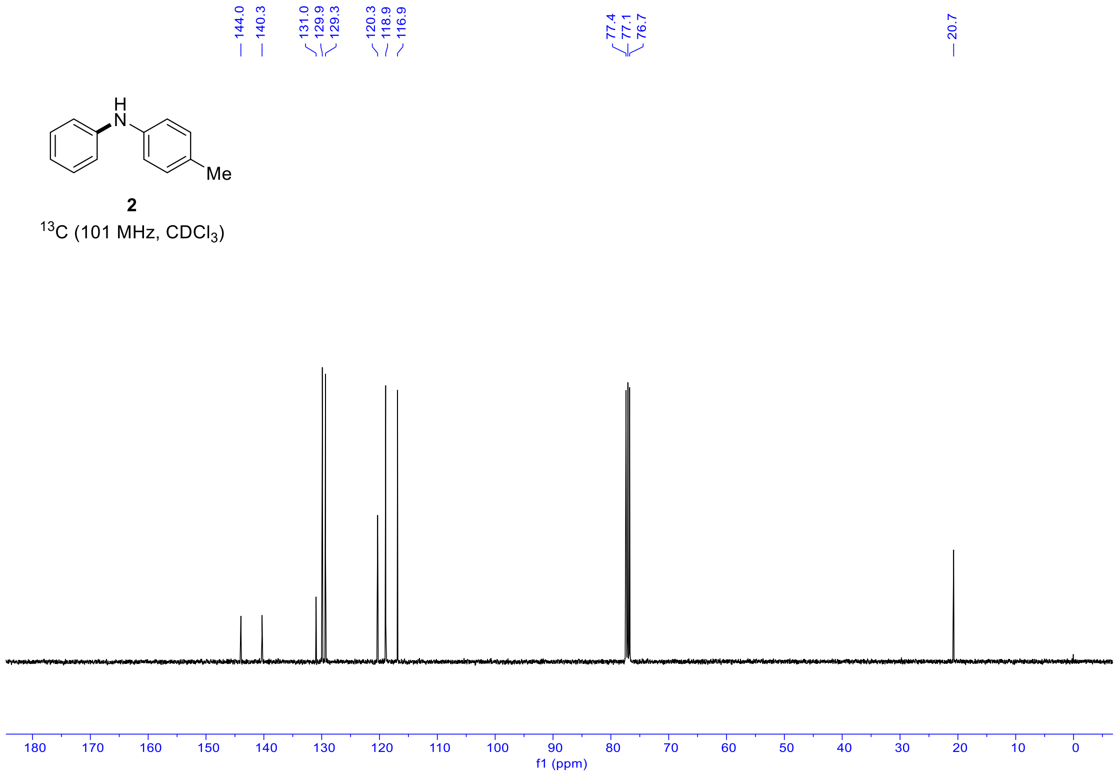

Supplementary Figure 38. ^{13}**C NMR** of compound **2**

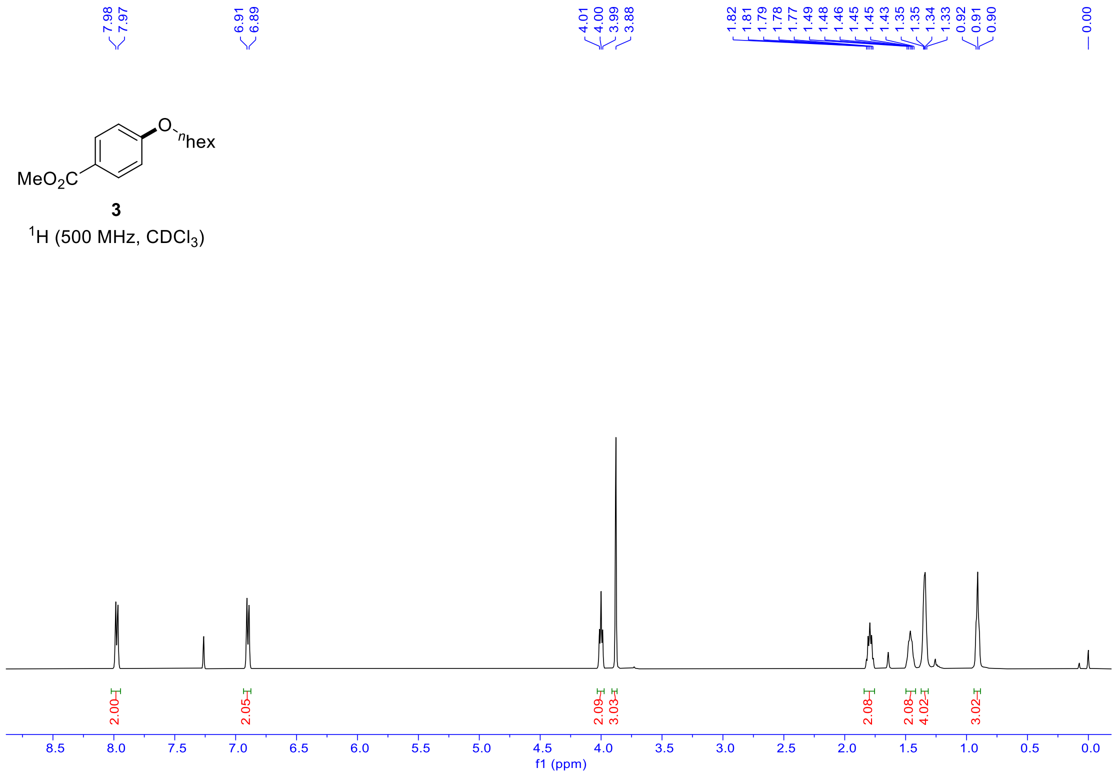

Supplementary Figure 39. ^{1}**H NMR** of compound **3**

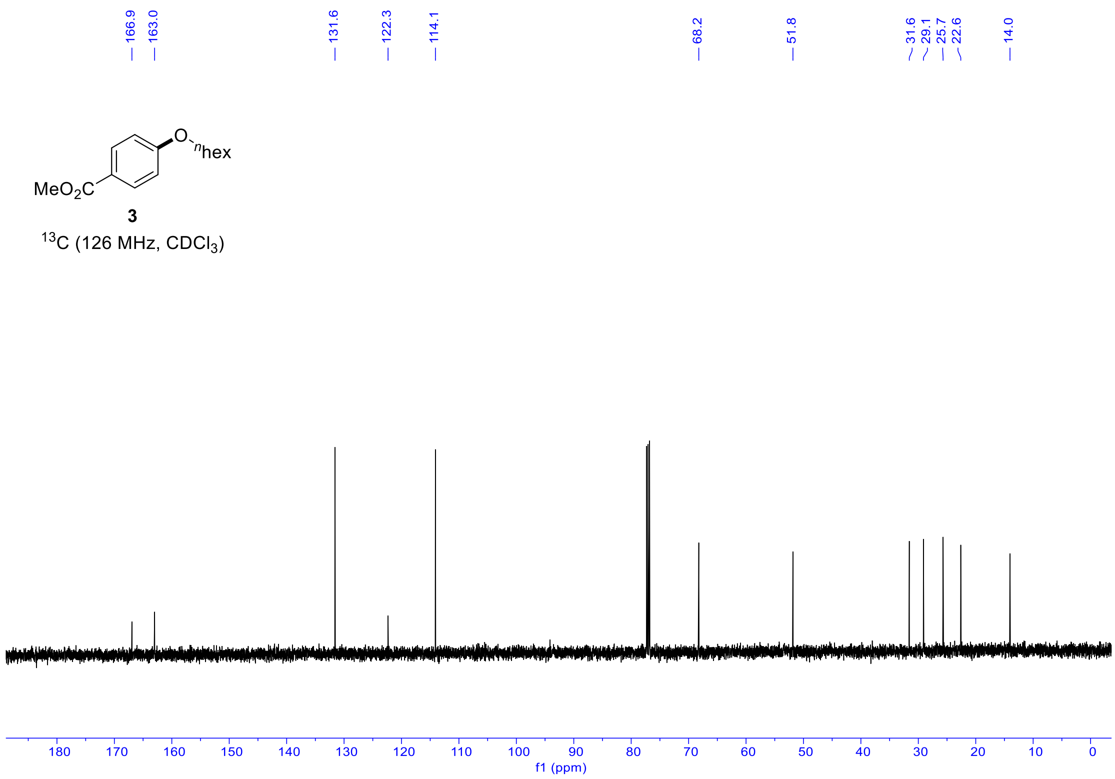

Supplementary Figure 40. ^{13}**C NMR** of compound **3**

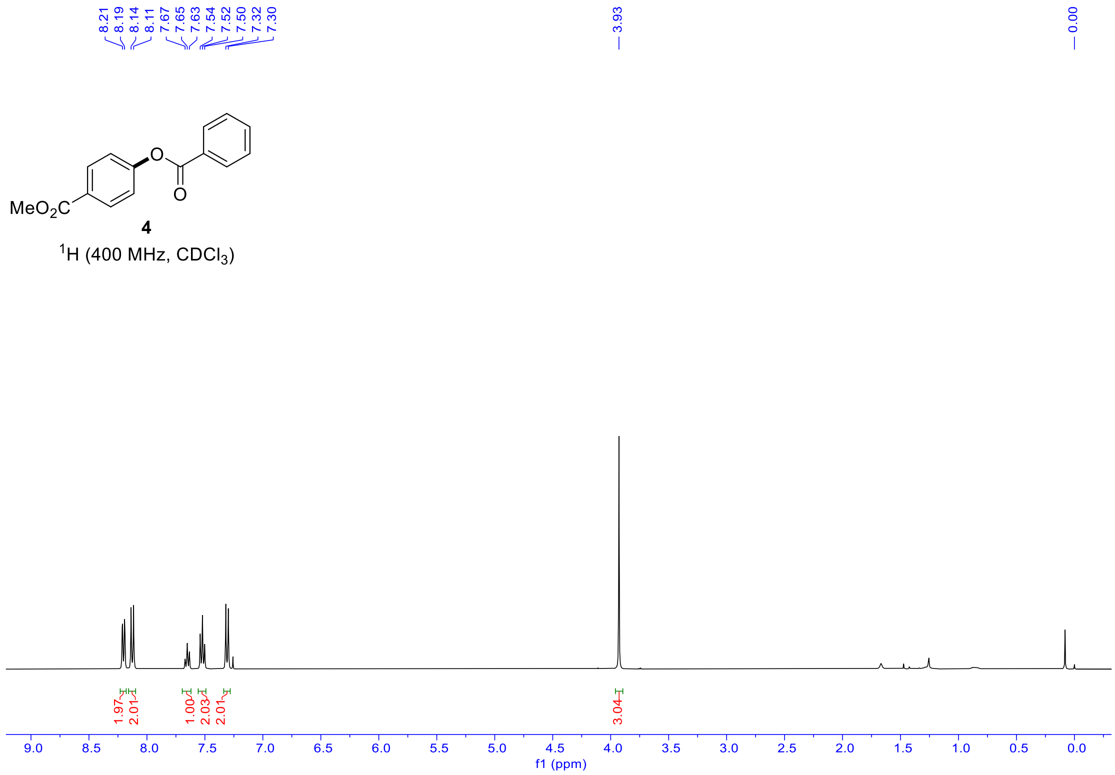

Supplementary Figure 41. ^{1}**H NMR** of compound **4**

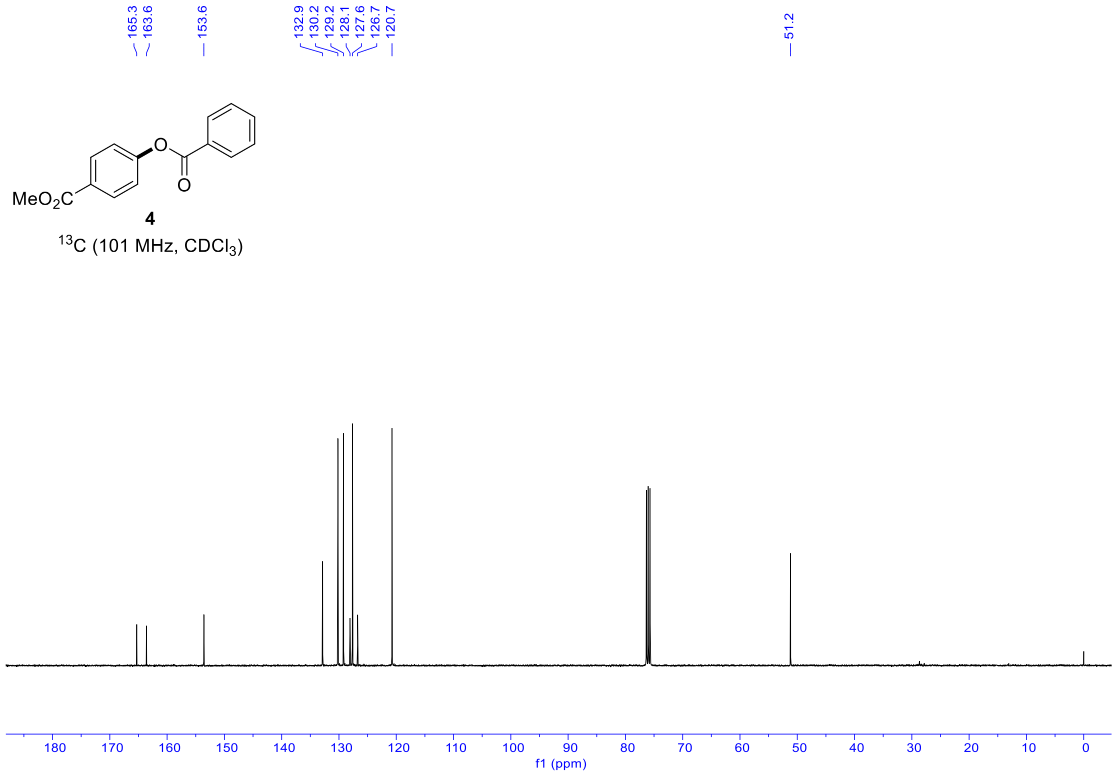

Supplementary Figure 42. ^{13}**C NMR** of compound **4**

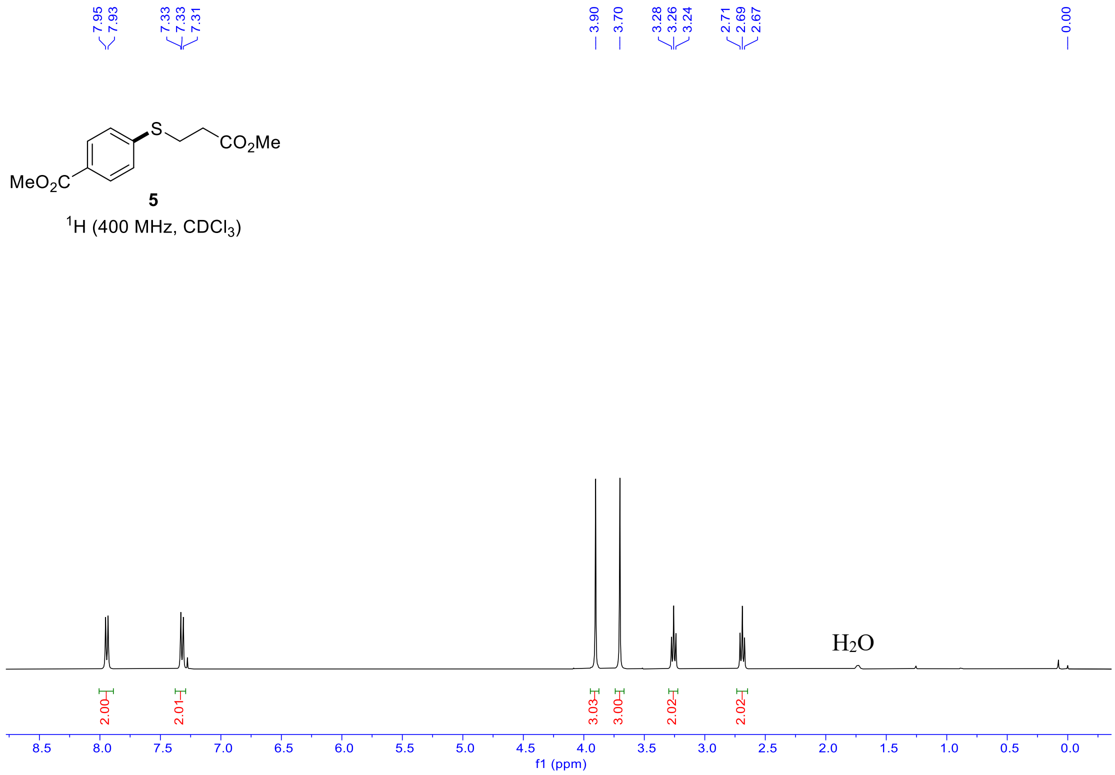

Supplementary Figure 43. ^{1}**H NMR** of compound **5**

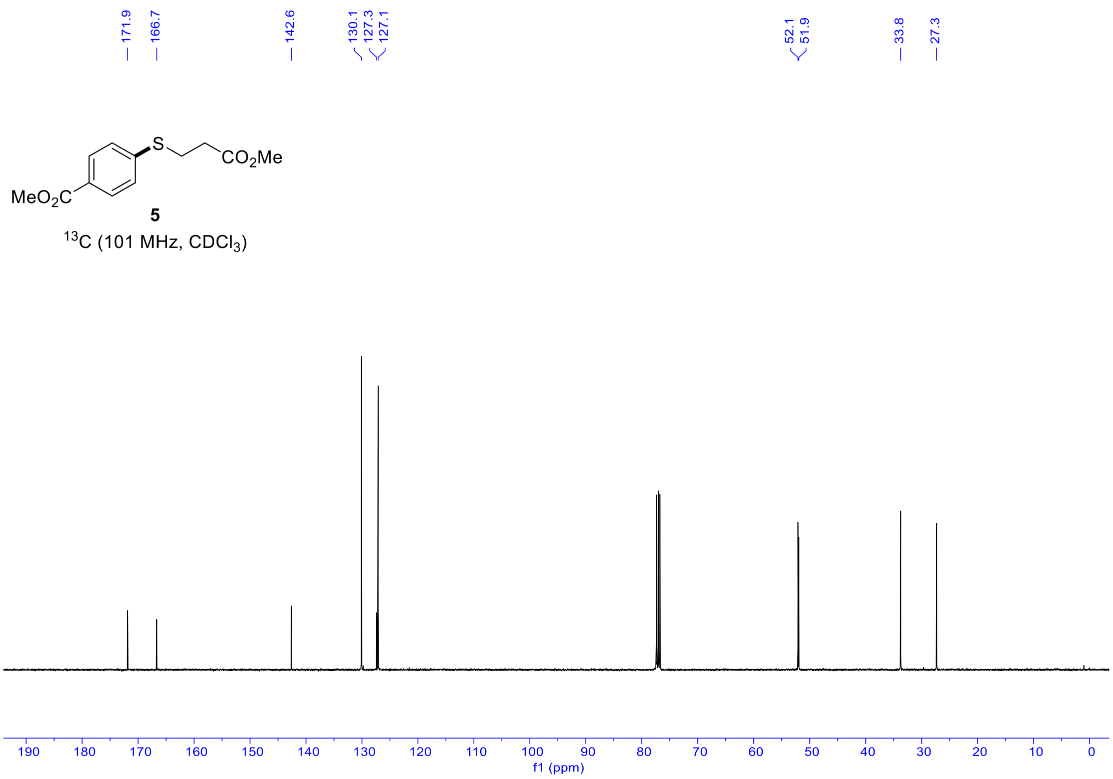

Supplementary Figure 44. ^{13}**C NMR** of compound **5**

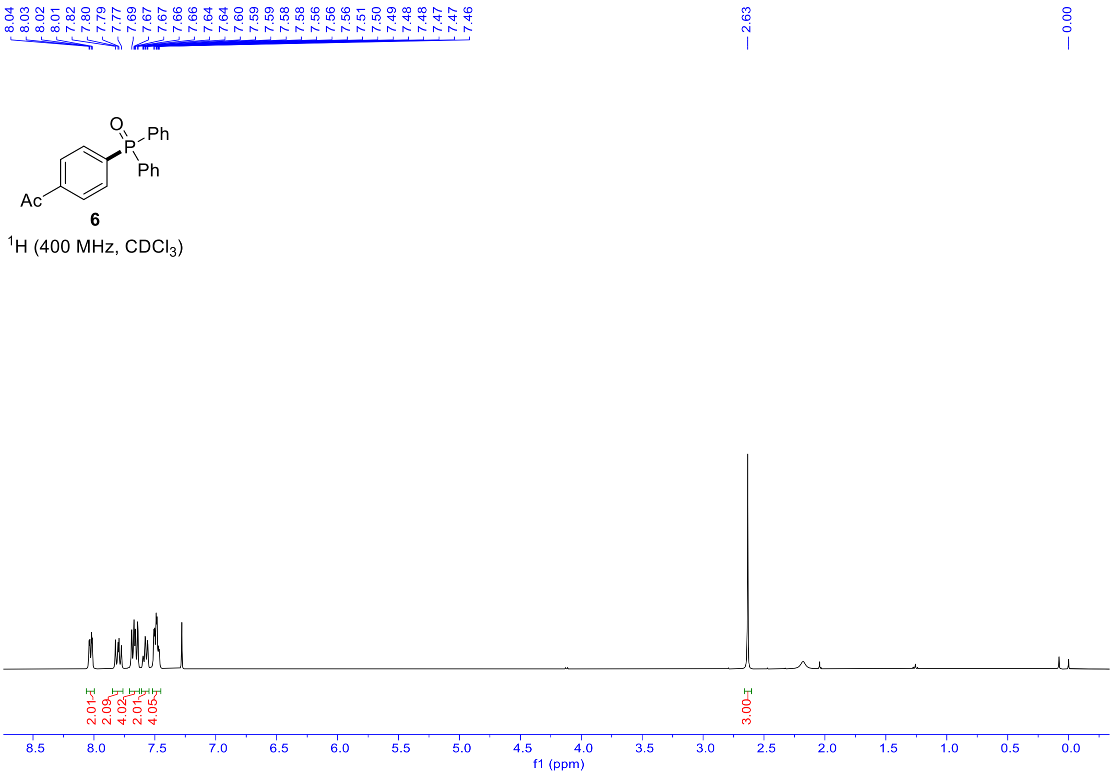

Supplementary Figure 45. ^{1}**H NMR** of compound **6**

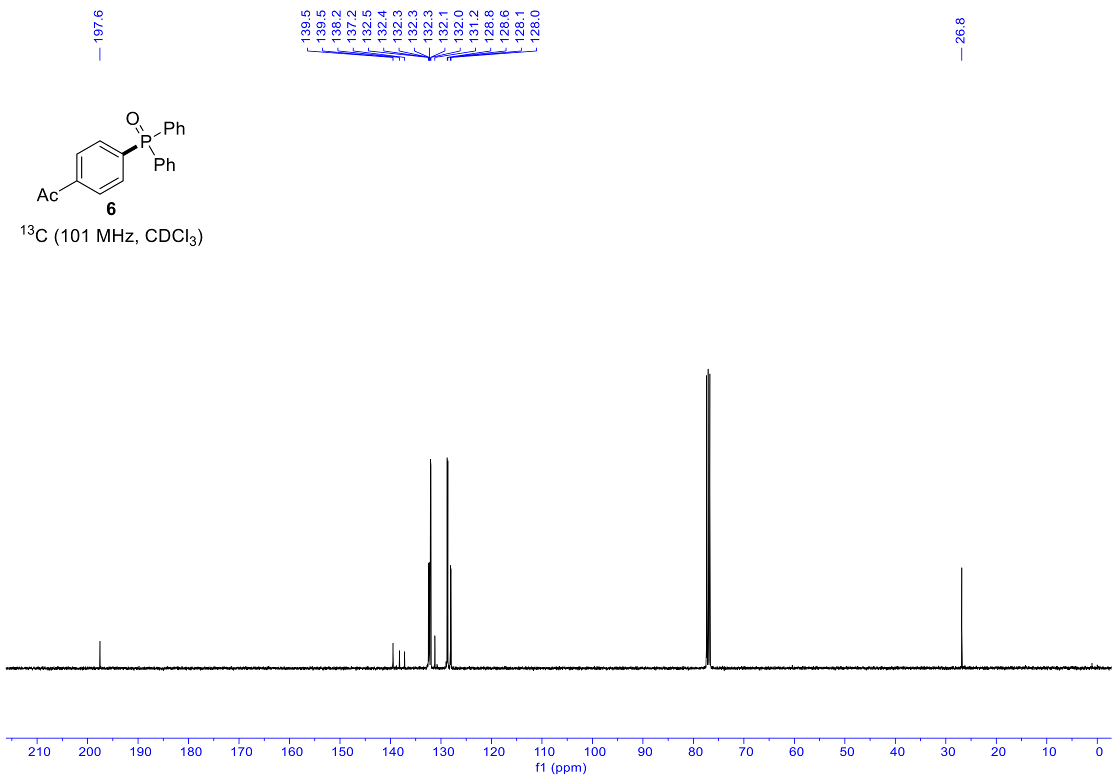

Supplementary Figure 46. ^{13}C NMR of compound **6**

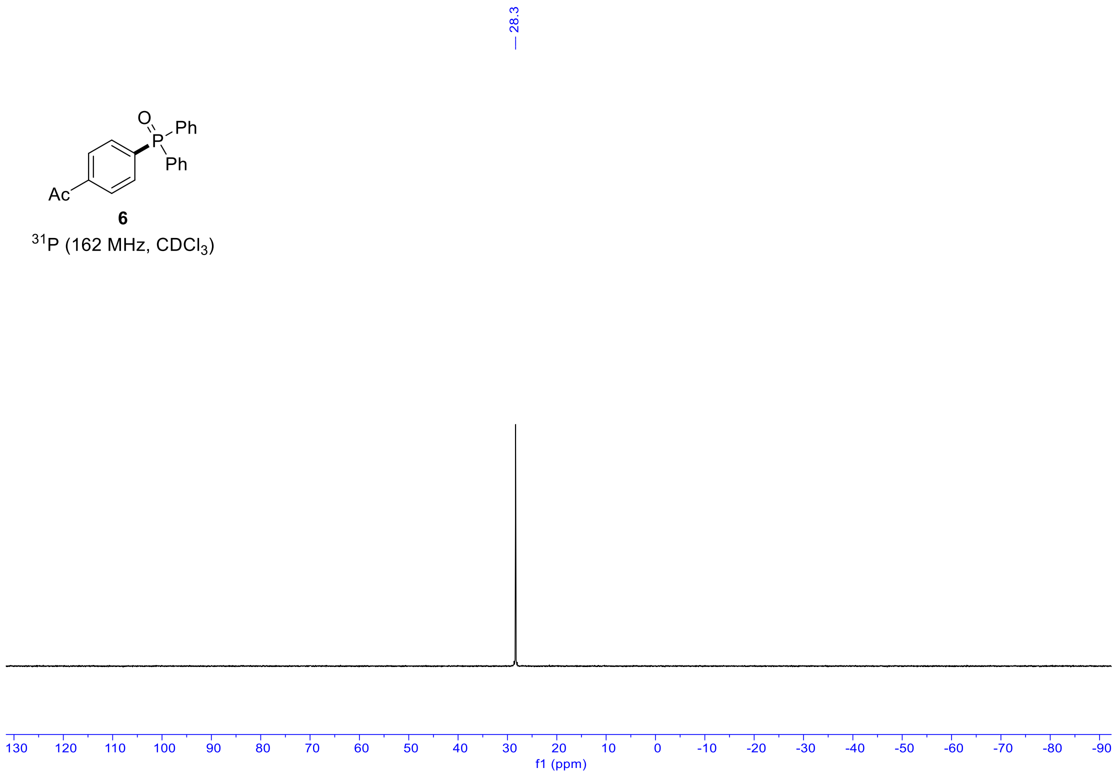

Supplementary Figure 47. ^{31}P NMR of compound **6**

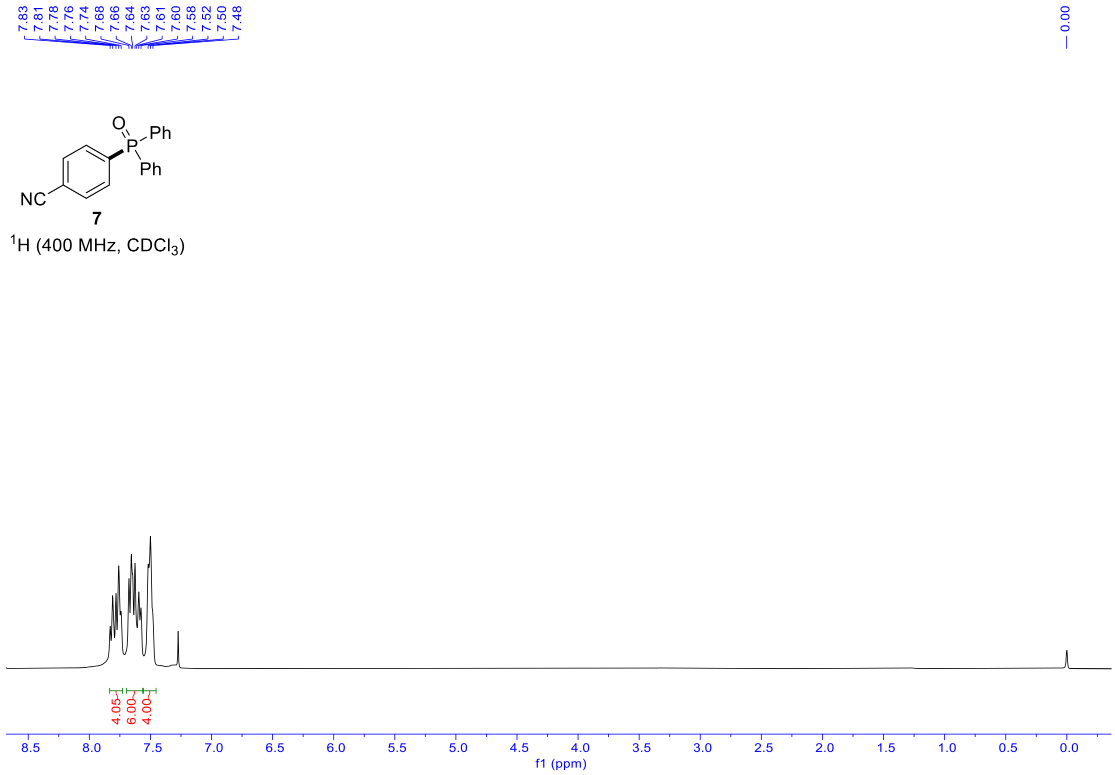

Supplementary Figure 48. **^{1}H NMR** of compound **7**

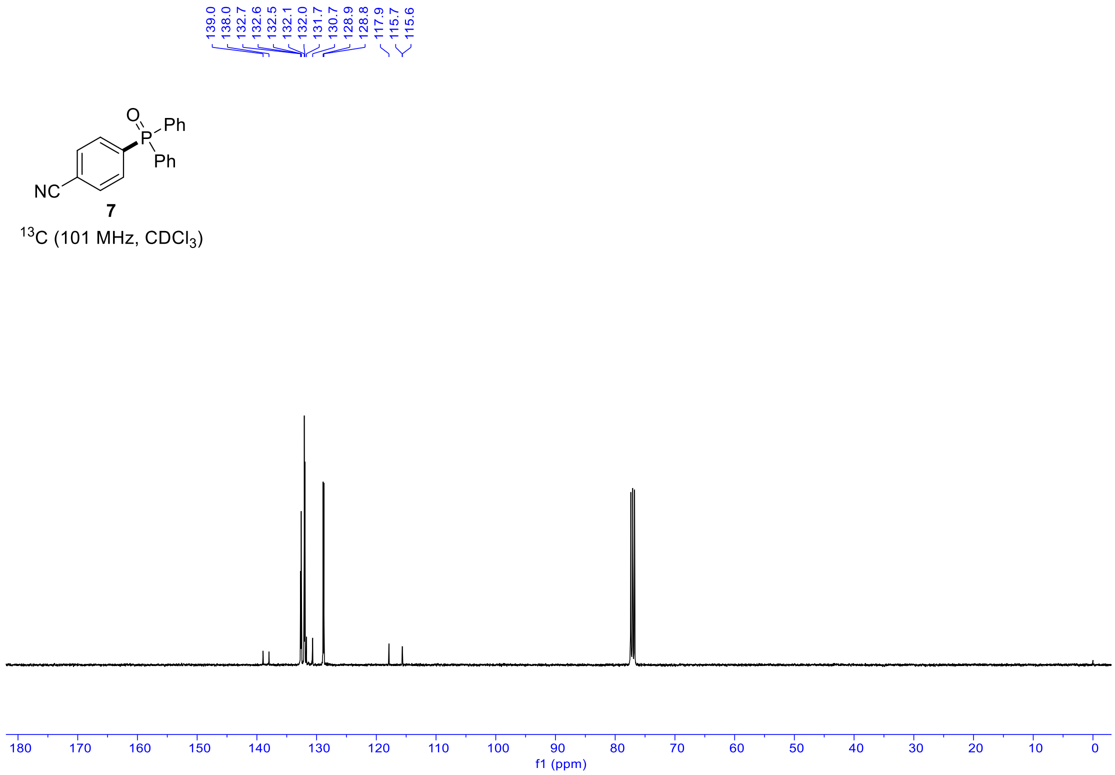

Supplementary Figure 49. **^{13}C NMR** of compound **7**

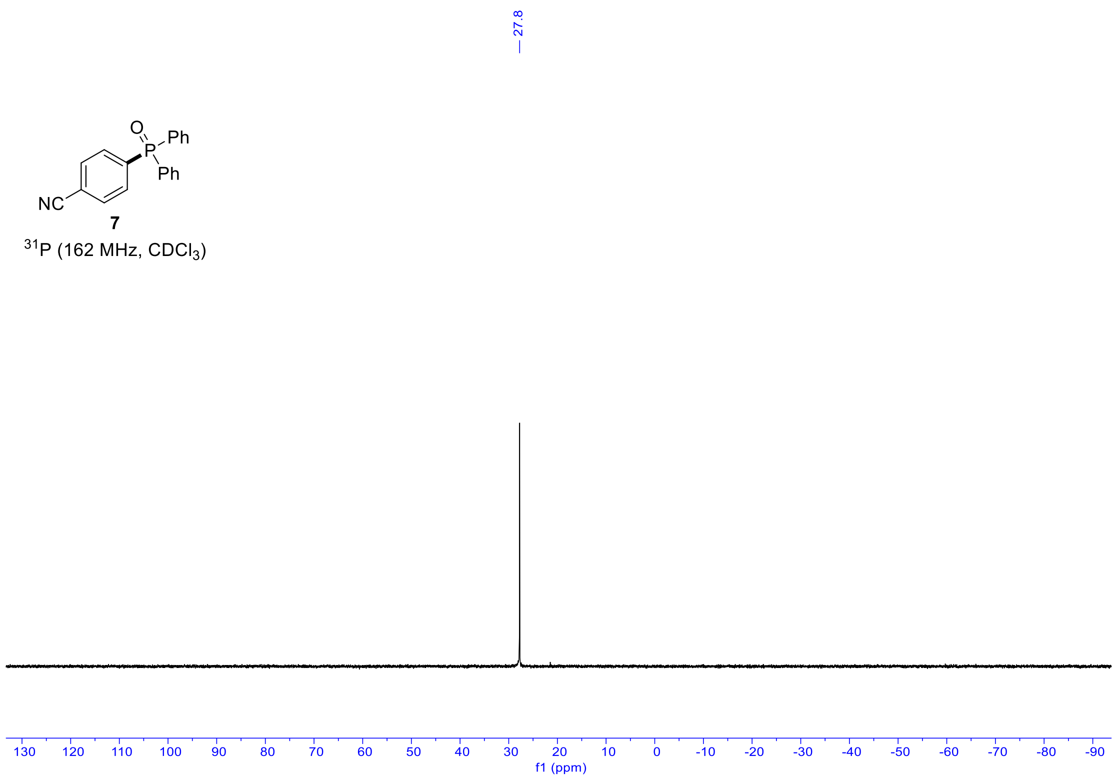

Supplementary Figure 50. ^{31}P NMR of compound **7**

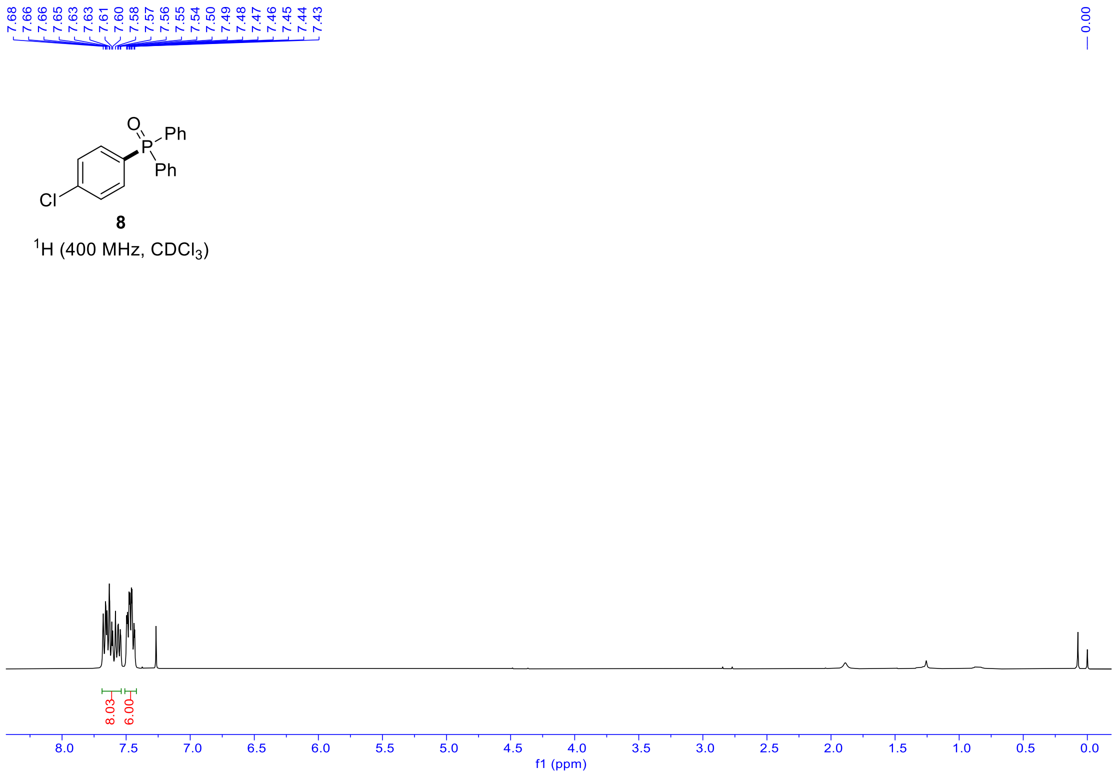

Supplementary Figure 51. ^{1}H NMR of compound **8**

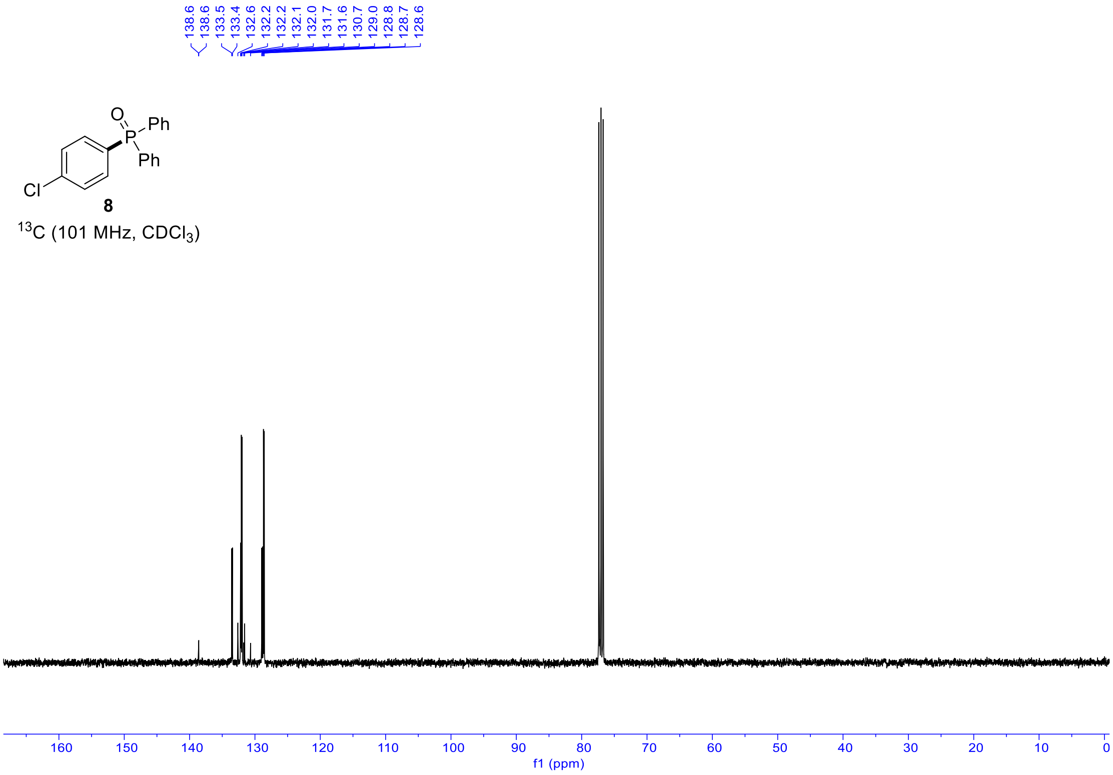

Supplementary Figure 52. ^{13}**C NMR** of compound **8**

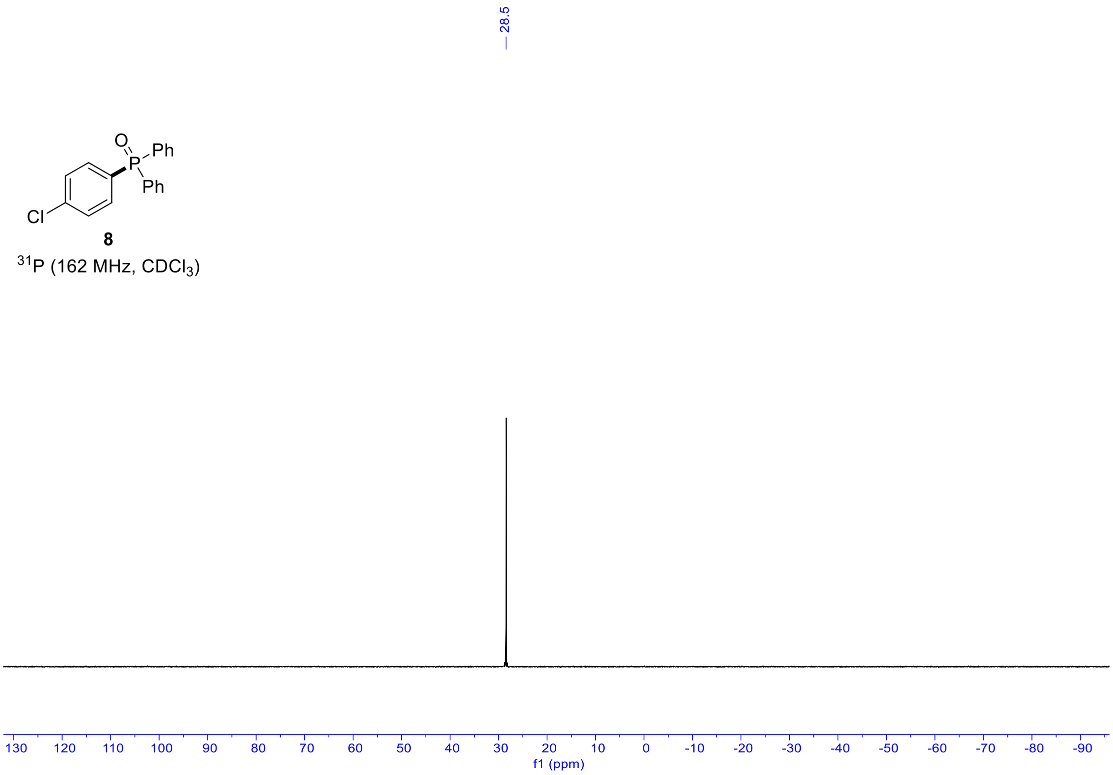

Supplementary Figure 53. ^{31}**P NMR** of compound **8**

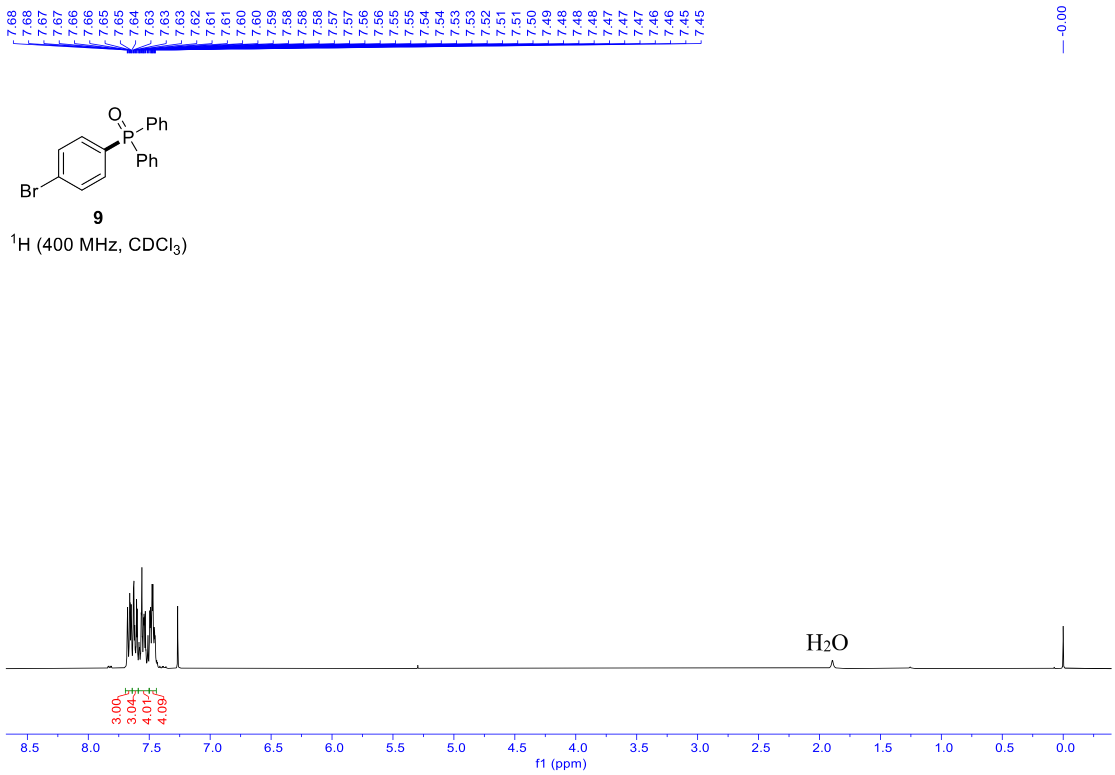

Supplementary Figure 54. ^{1}**H NMR** of compound **9**

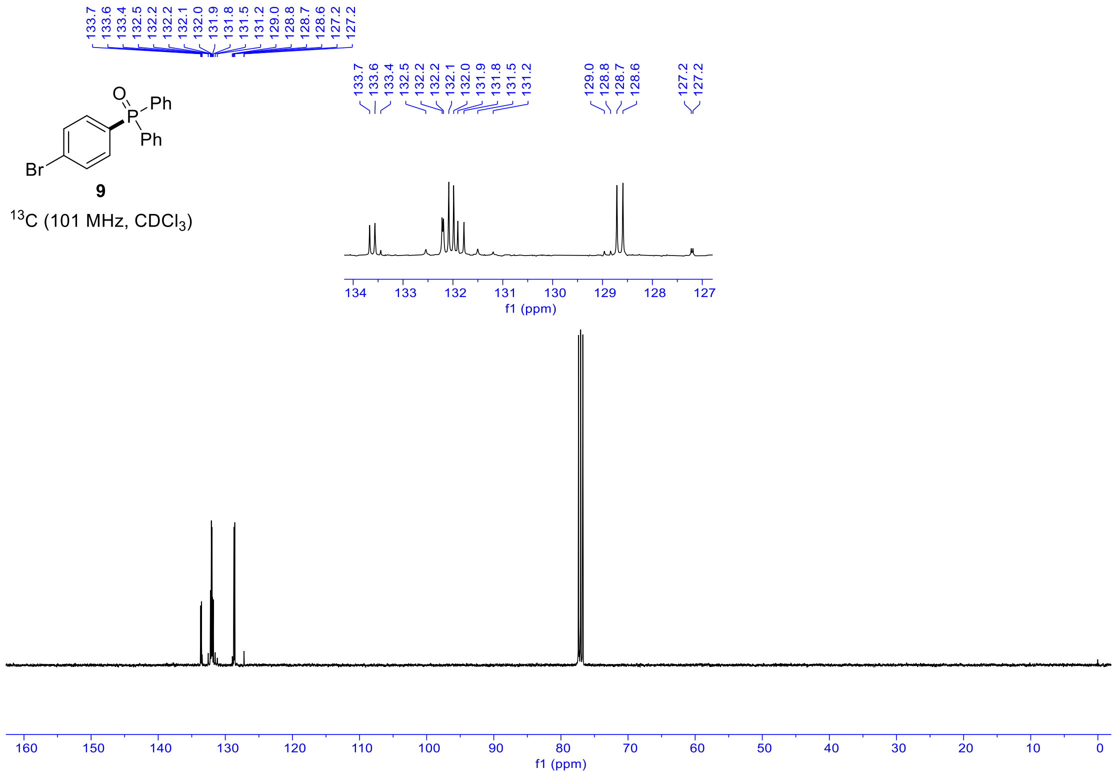

Supplementary Figure 55. ^{13}**C NMR** of compound **9**

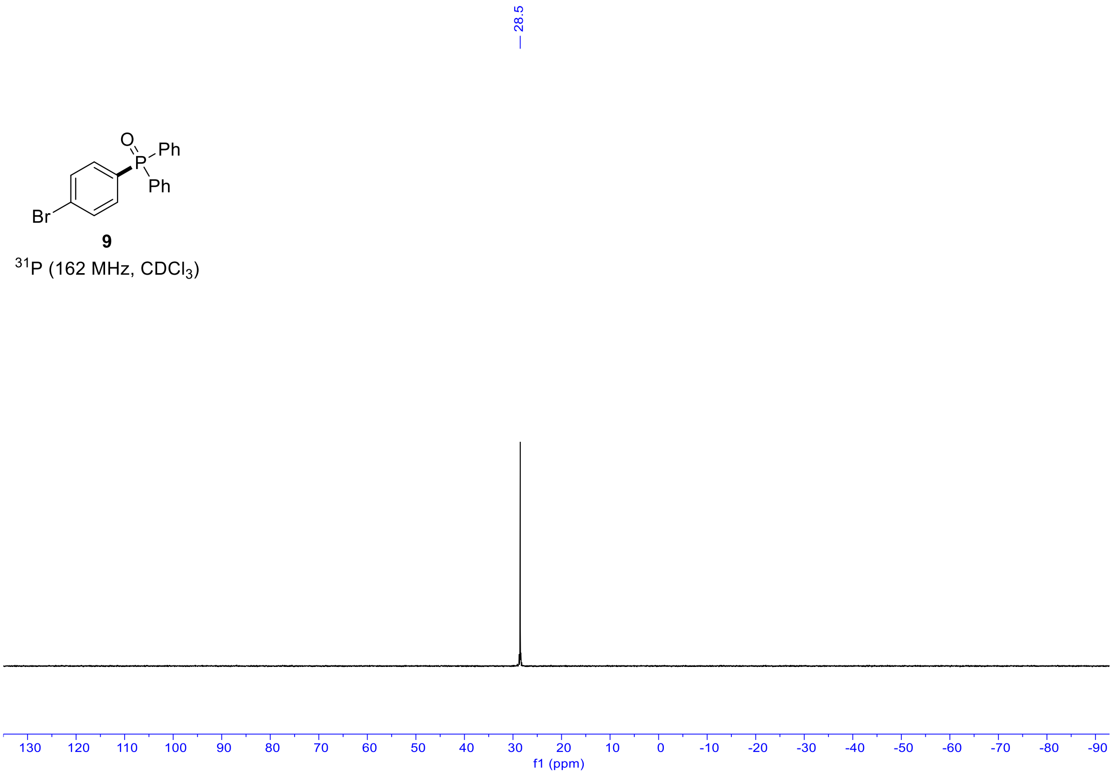

Supplementary Figure 56. ^{31}**P NMR** of compound **9**

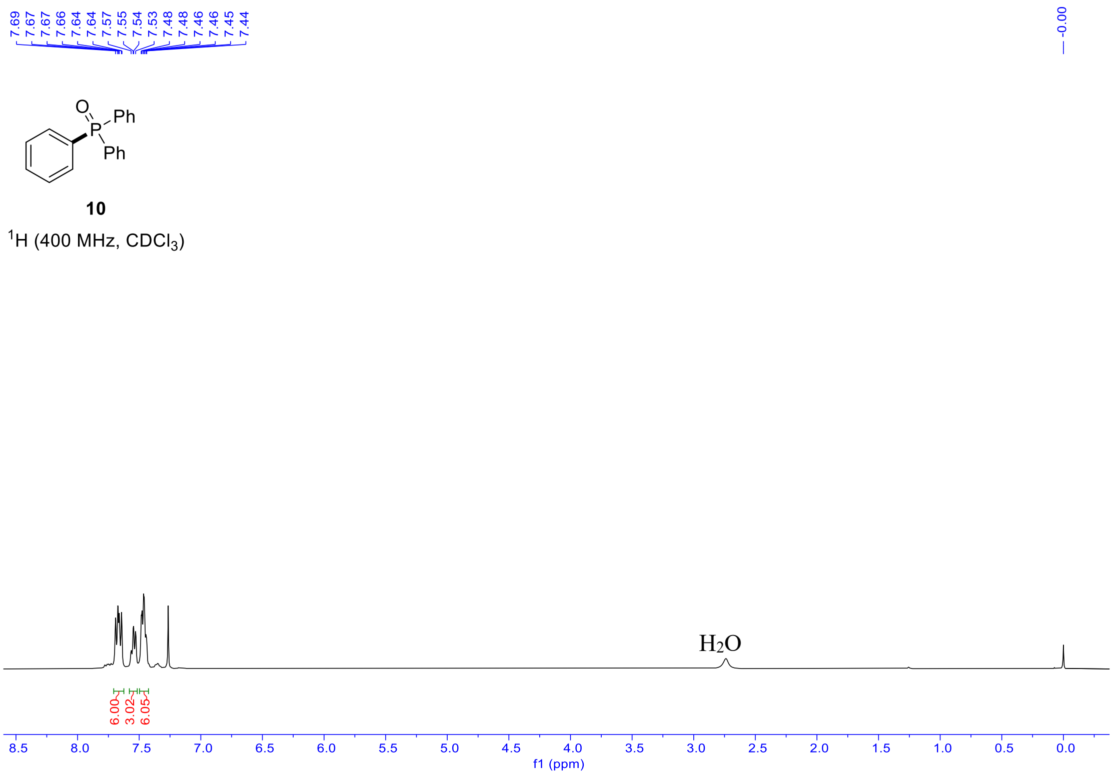

Supplementary Figure 57. ^{1}**H NMR** of compound **10**

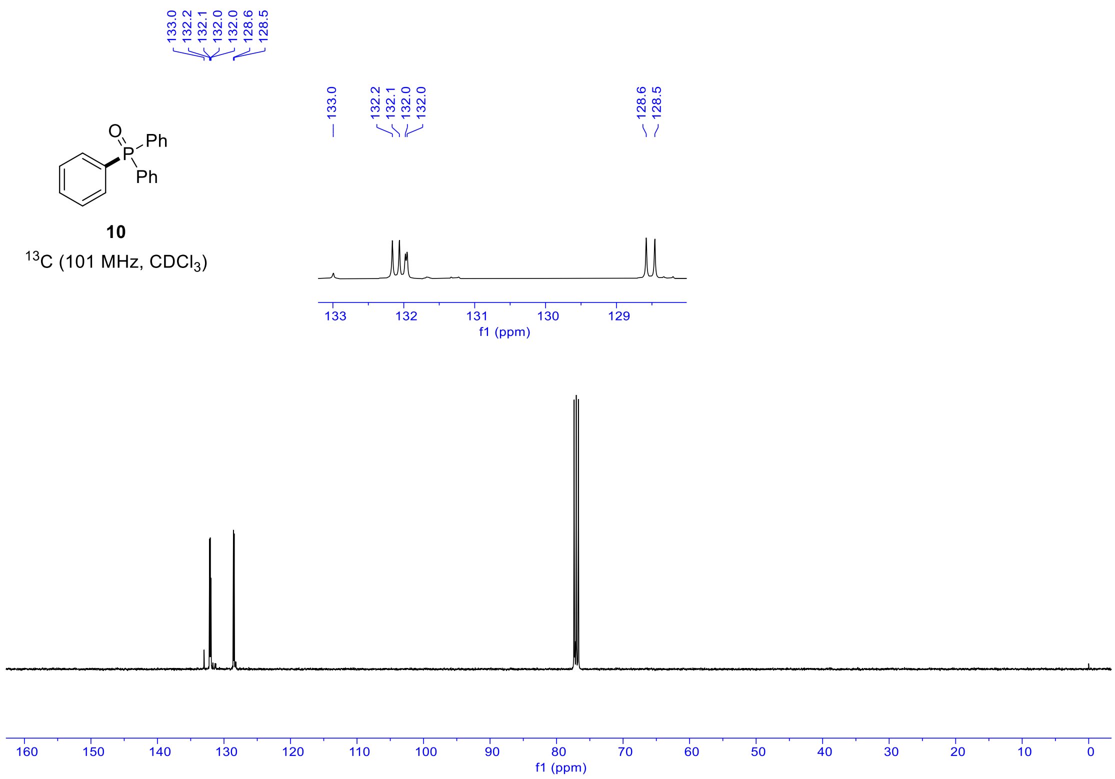

Supplementary Figure 58. ^{13}**C NMR** of compound **10**

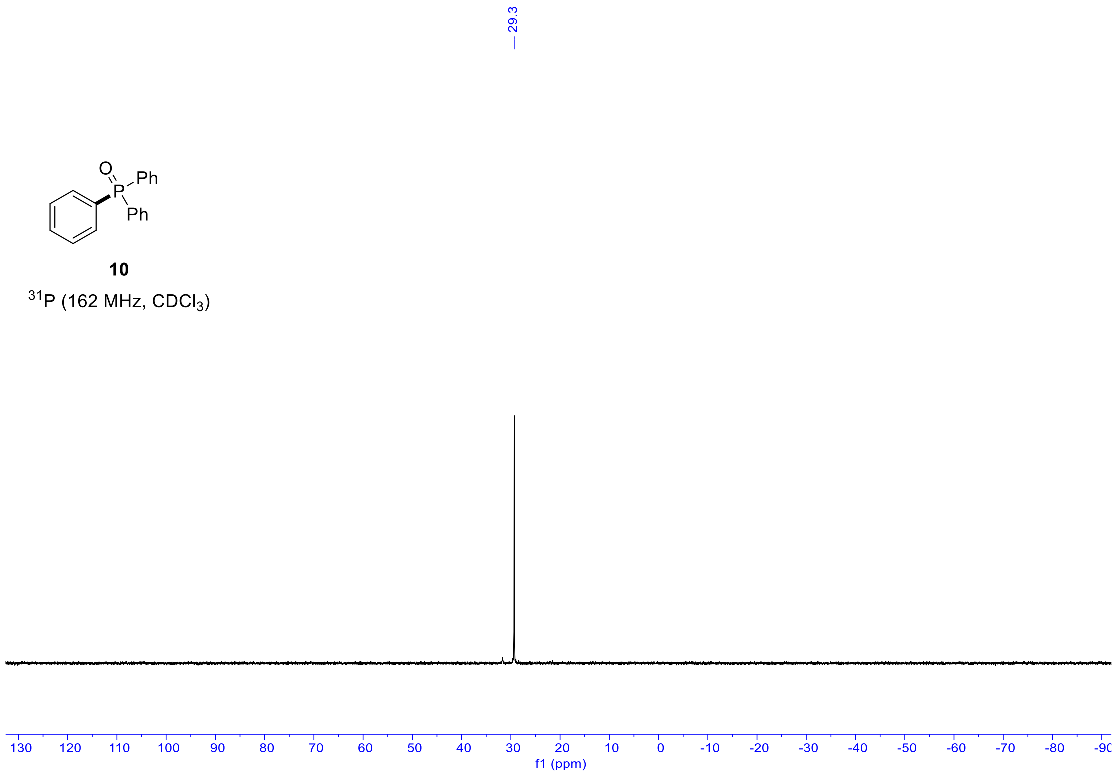

Supplementary Figure 59. ^{31}**P NMR** of compound **10**

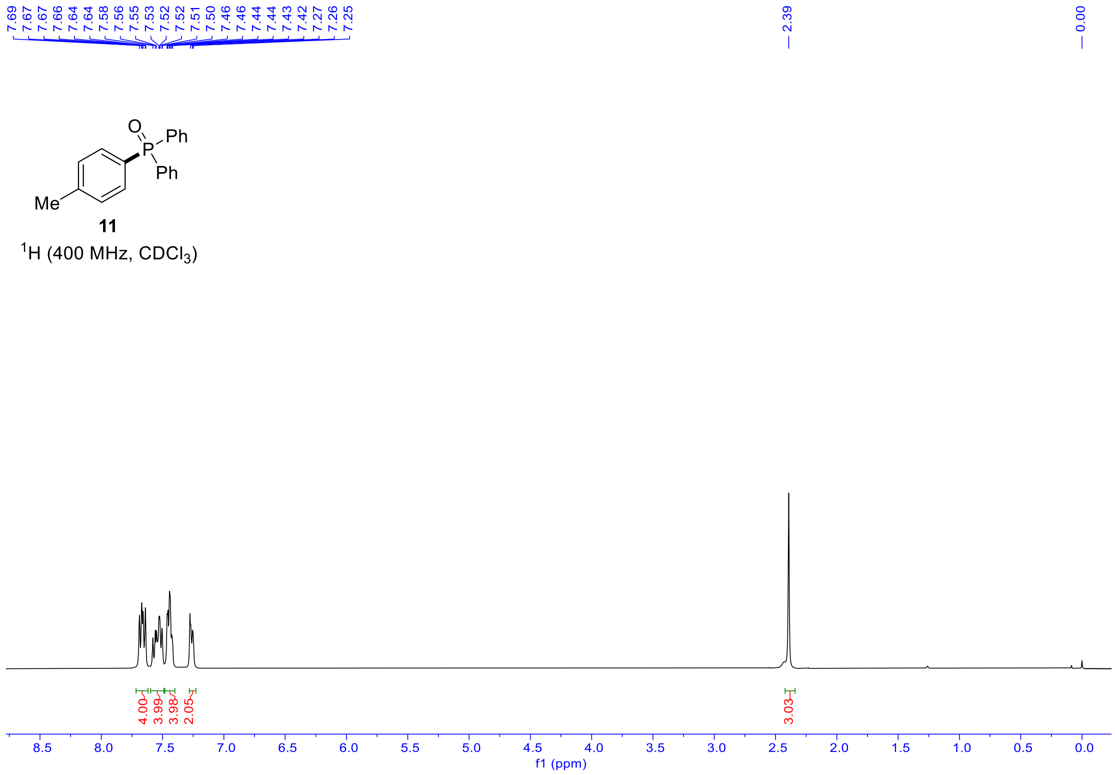

Supplementary Figure 60. ^{1}H NMR of compound **11**

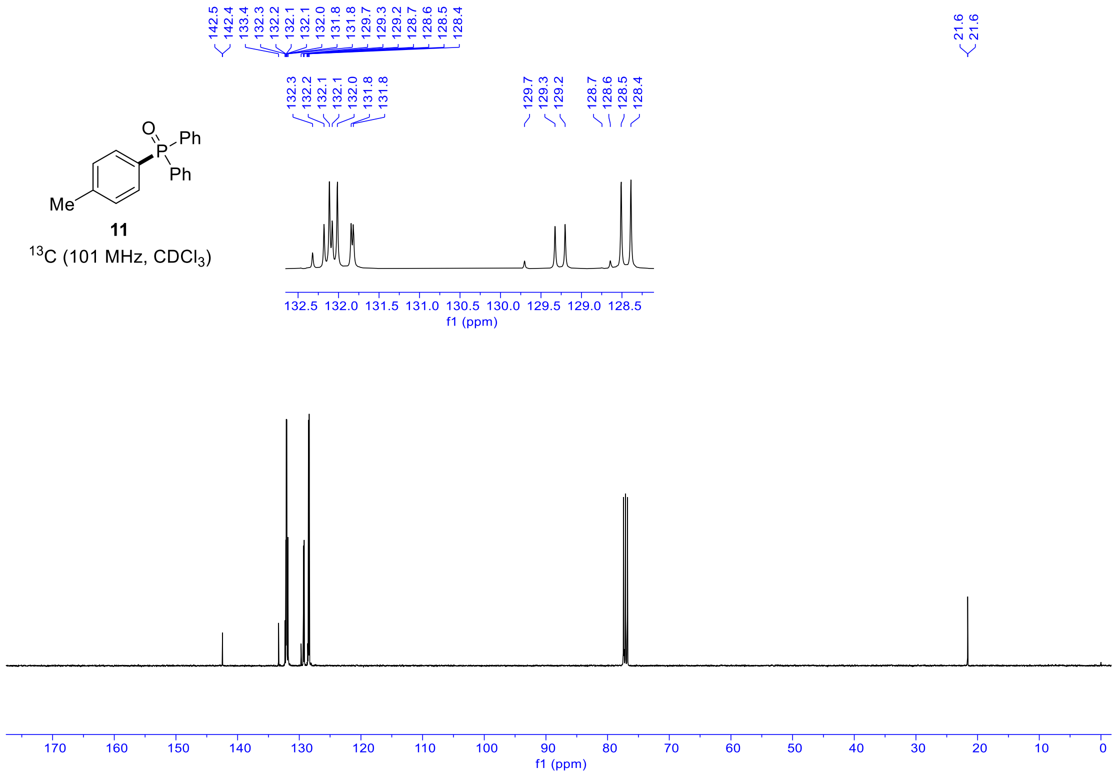

Supplementary Figure 61. ^{13}C NMR of compound **11**

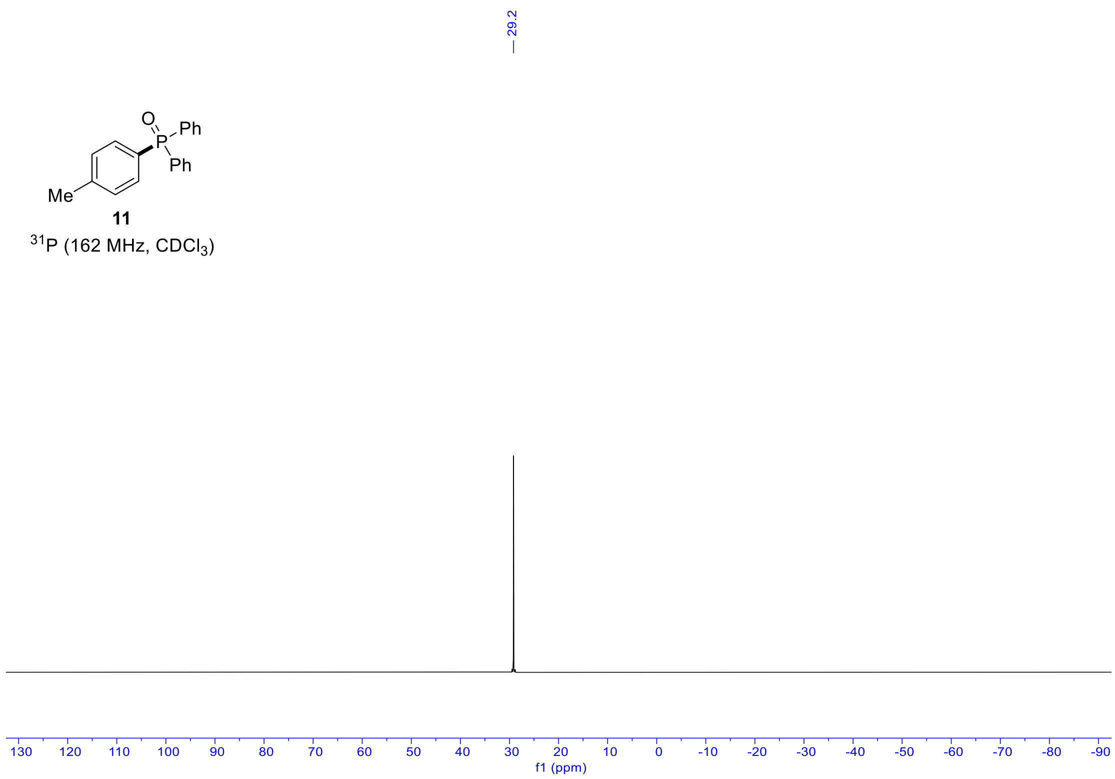

Supplementary Figure 62. **^{31}P NMR** of compound **11**

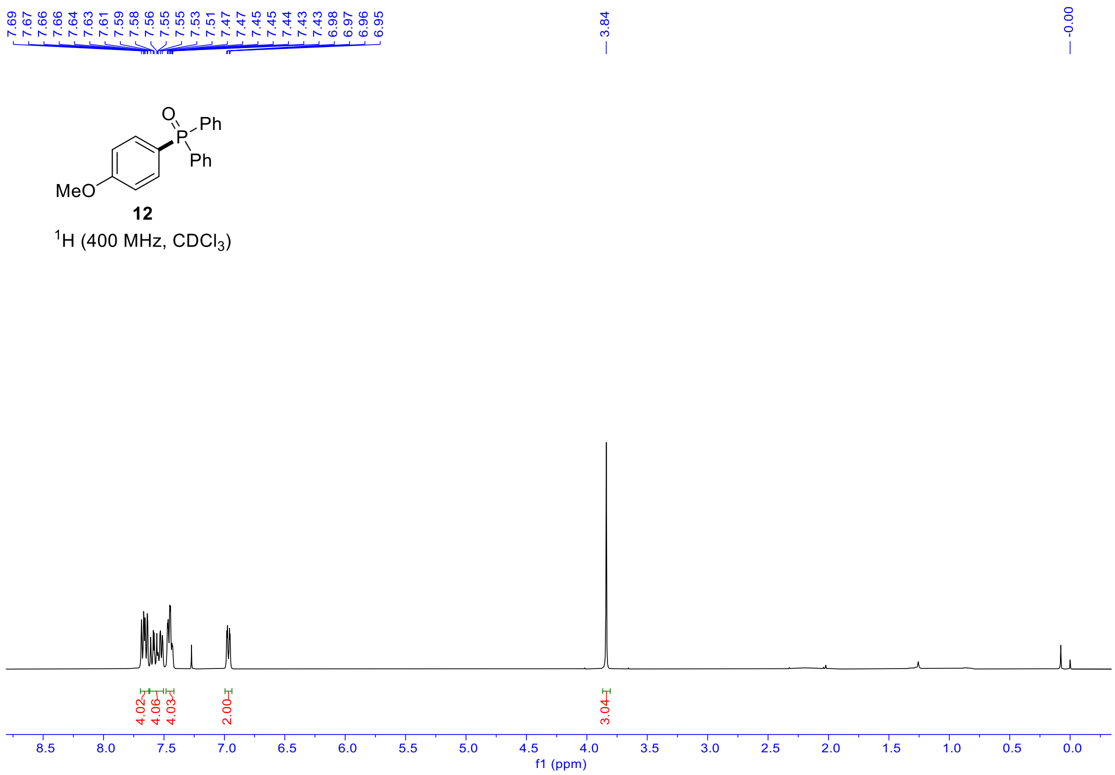

Supplementary Figure 63. **^{1}H NMR** of compound **12**

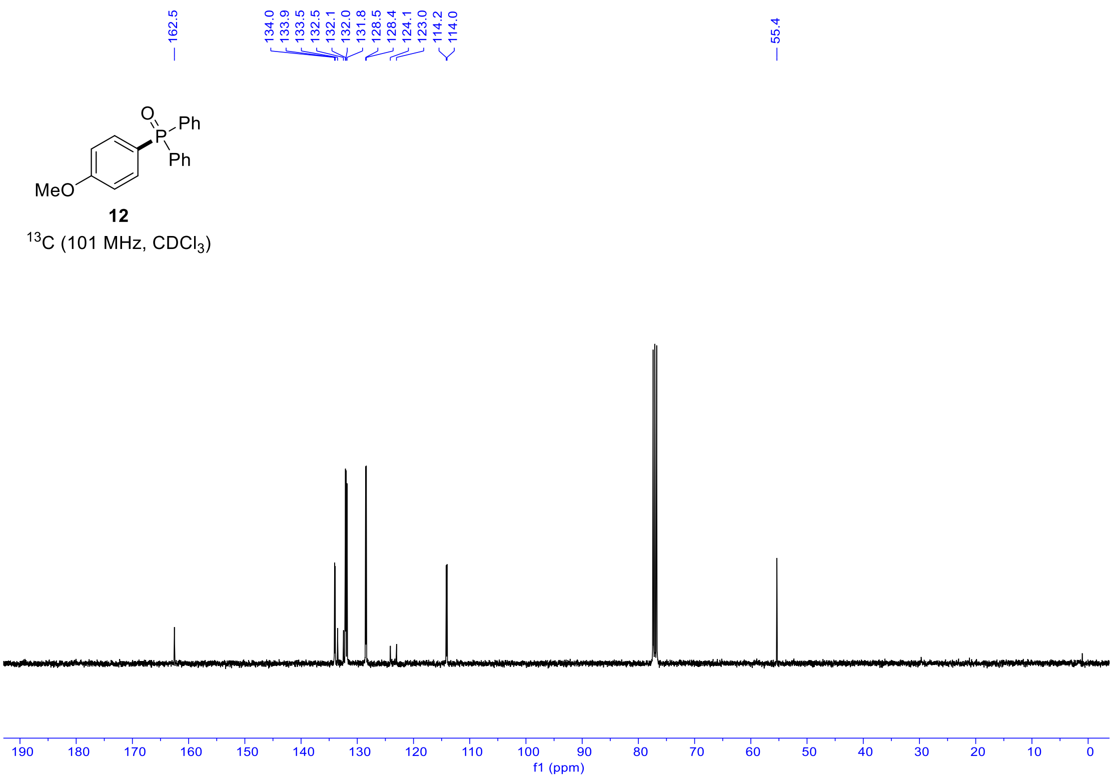

Supplementary Figure 64. ^{13}**C NMR** of compound **12**

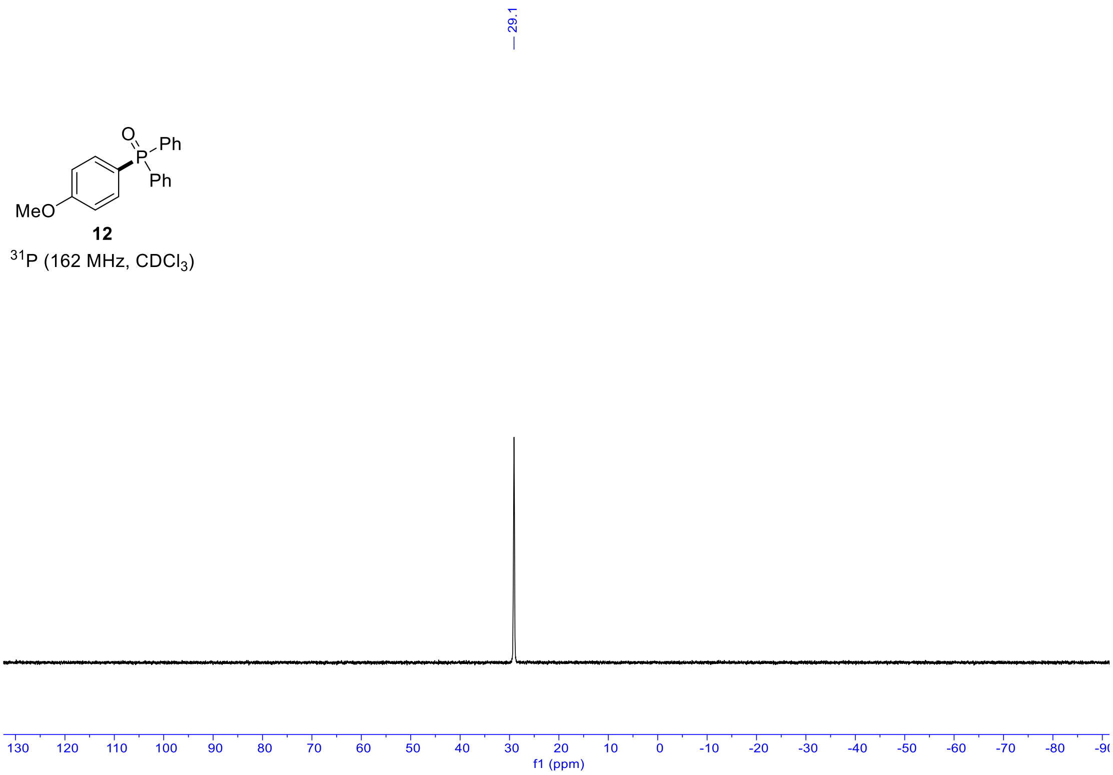

Supplementary Figure 65. ^{31}**P NMR** of compound **12**

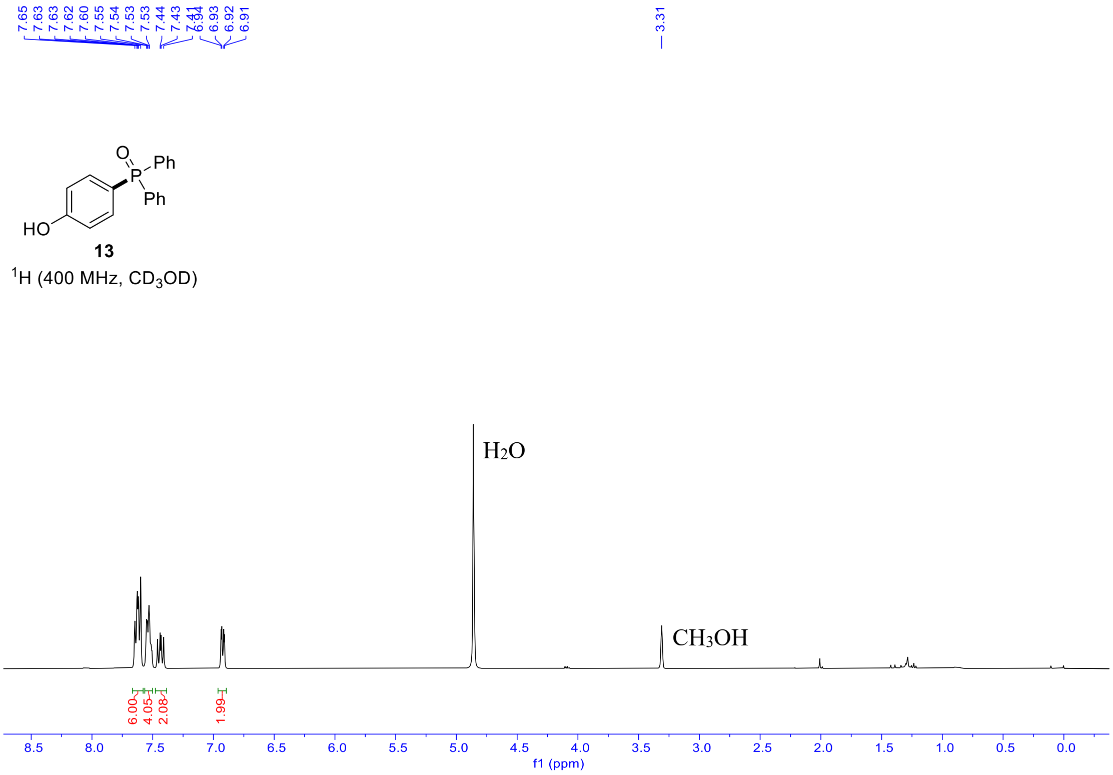

Supplementary Figure 66. **^{1}H NMR** of compound **13**

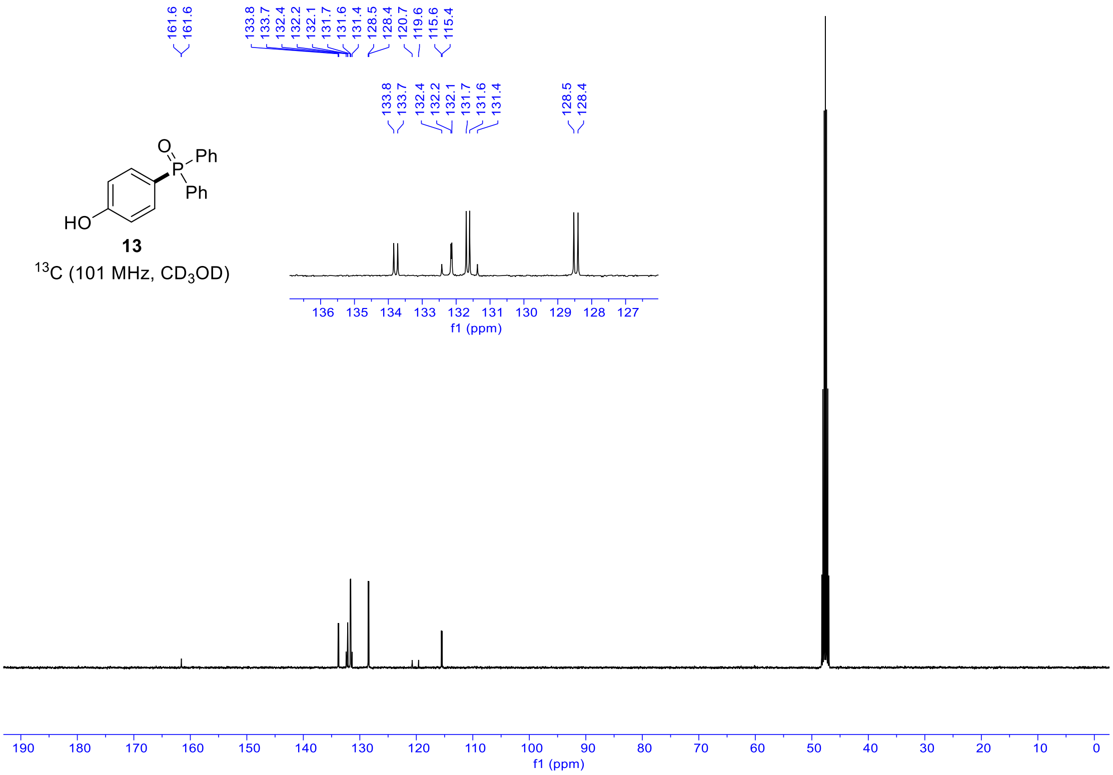

Supplementary Figure 67. **^{13}C NMR** of compound **13**

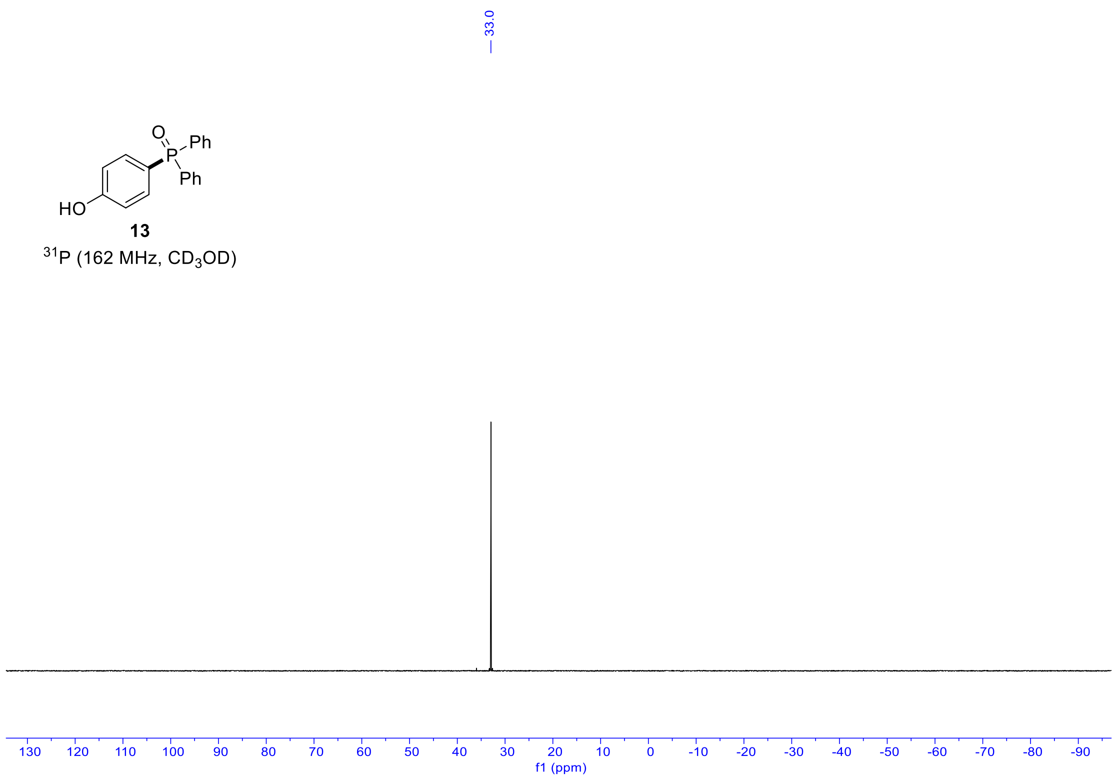

Supplementary Figure 68. ^{31}**P NMR** of compound **13**

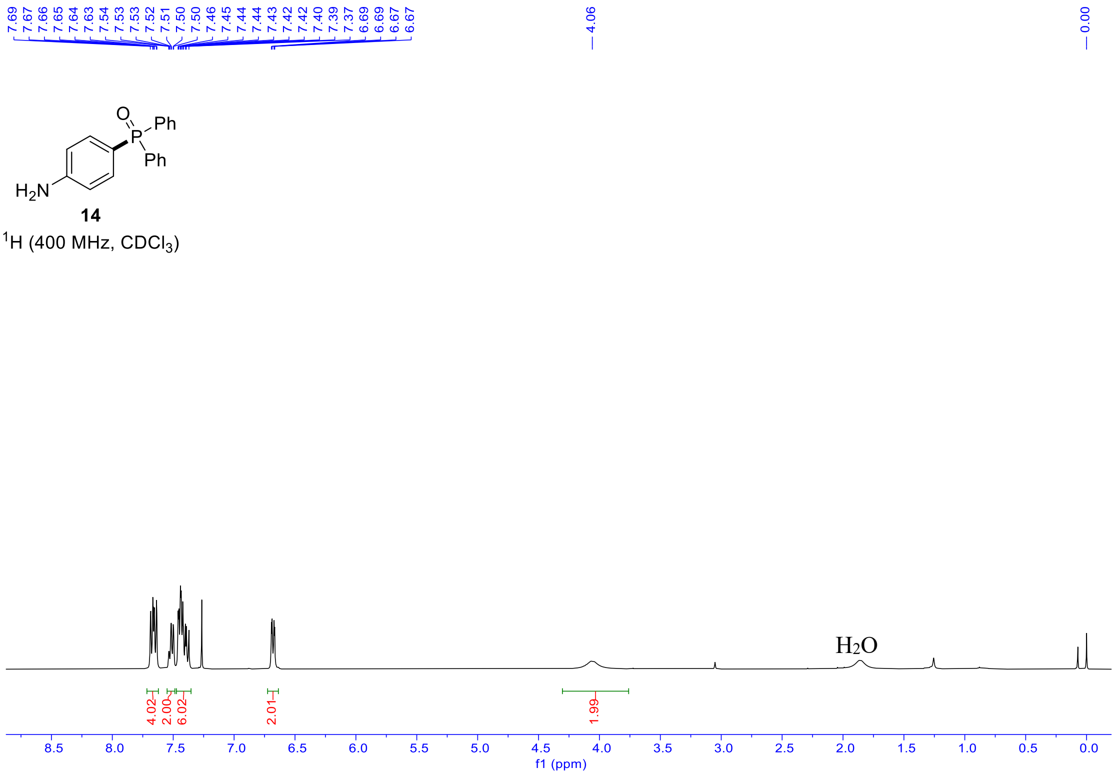

Supplementary Figure 69. ^{1}**H NMR** of compound **14**

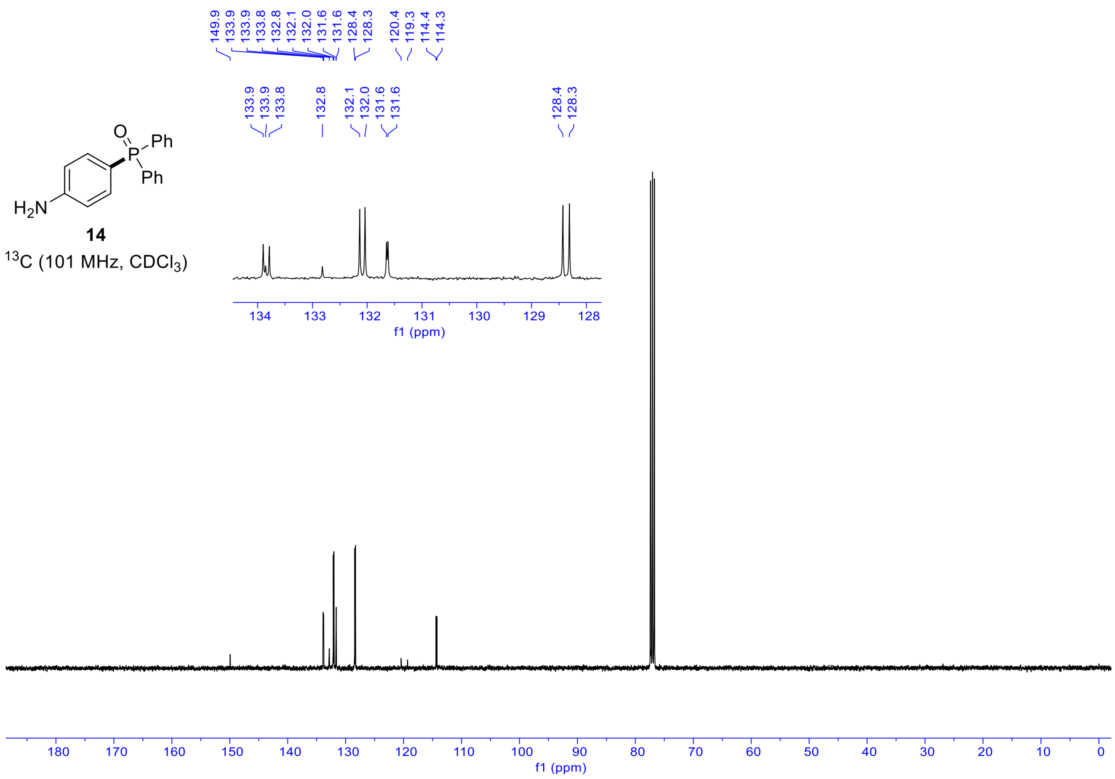

Supplementary Figure 70. ^{13}**C NMR** of compound **14**

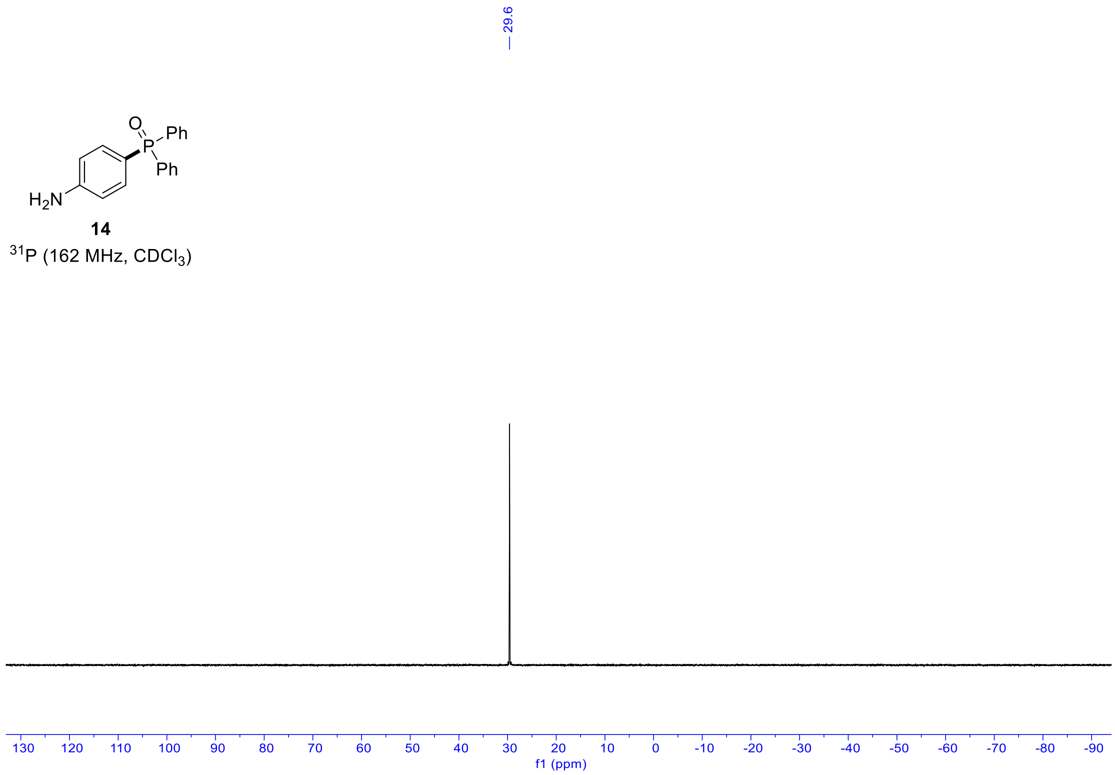

Supplementary Figure 71. ^{31}**P NMR** of compound **14**

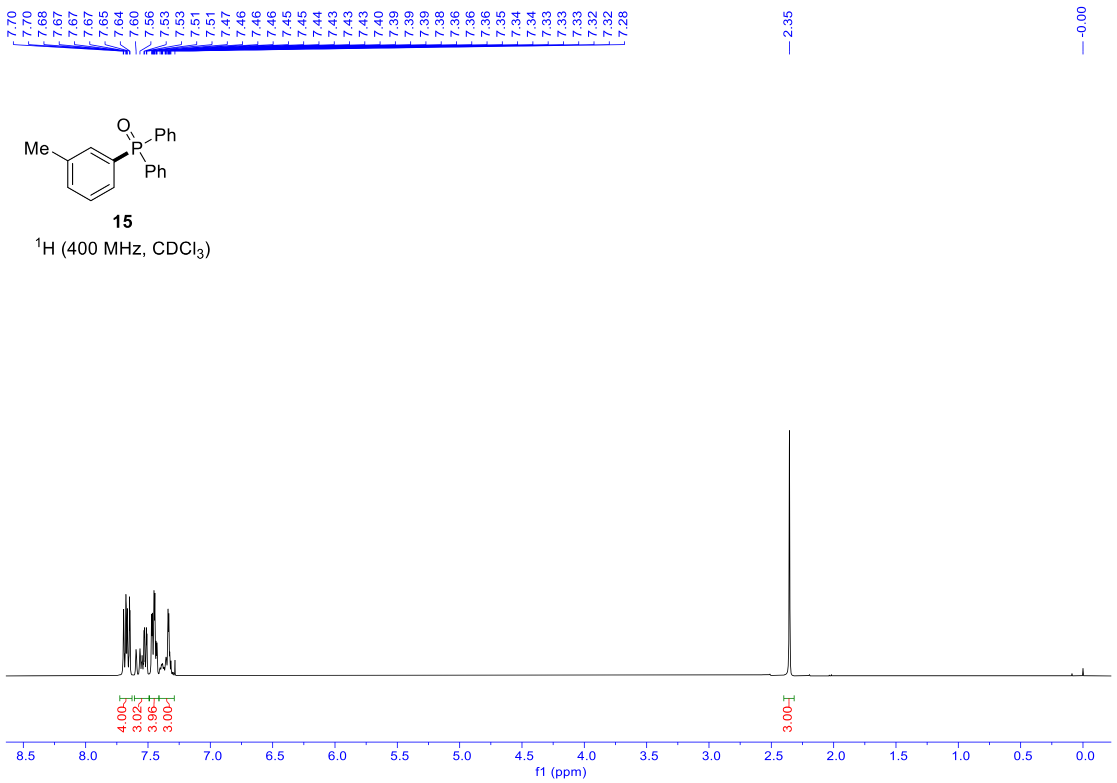

Supplementary Figure 72. ^{1}**H NMR** of compound **15**

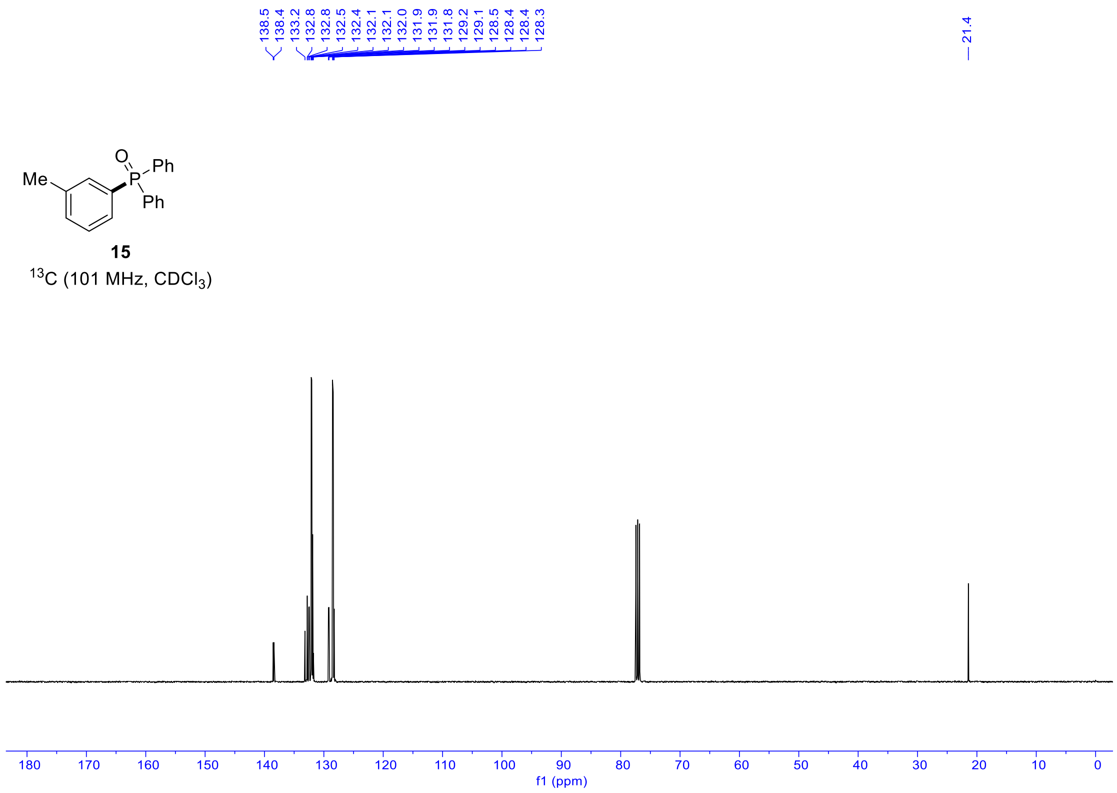

Supplementary Figure 73. ^{13}**C NMR** of compound **15**

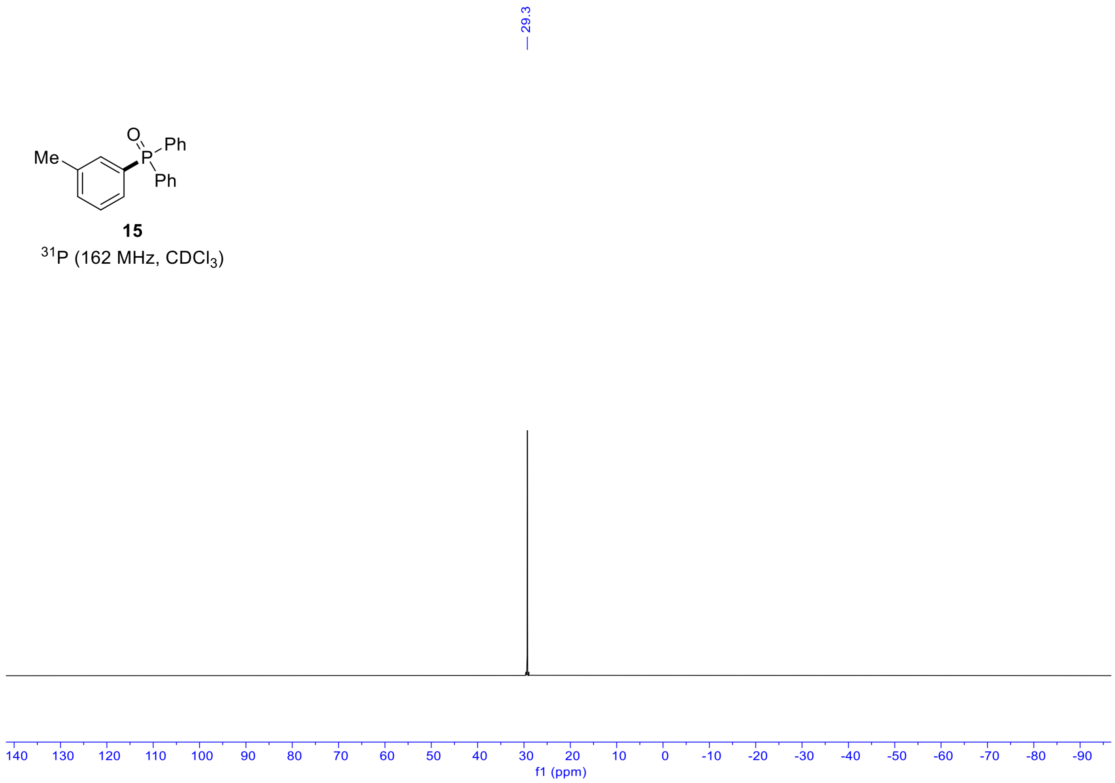

Supplementary Figure 74. **^{31}P NMR** of compound **15**

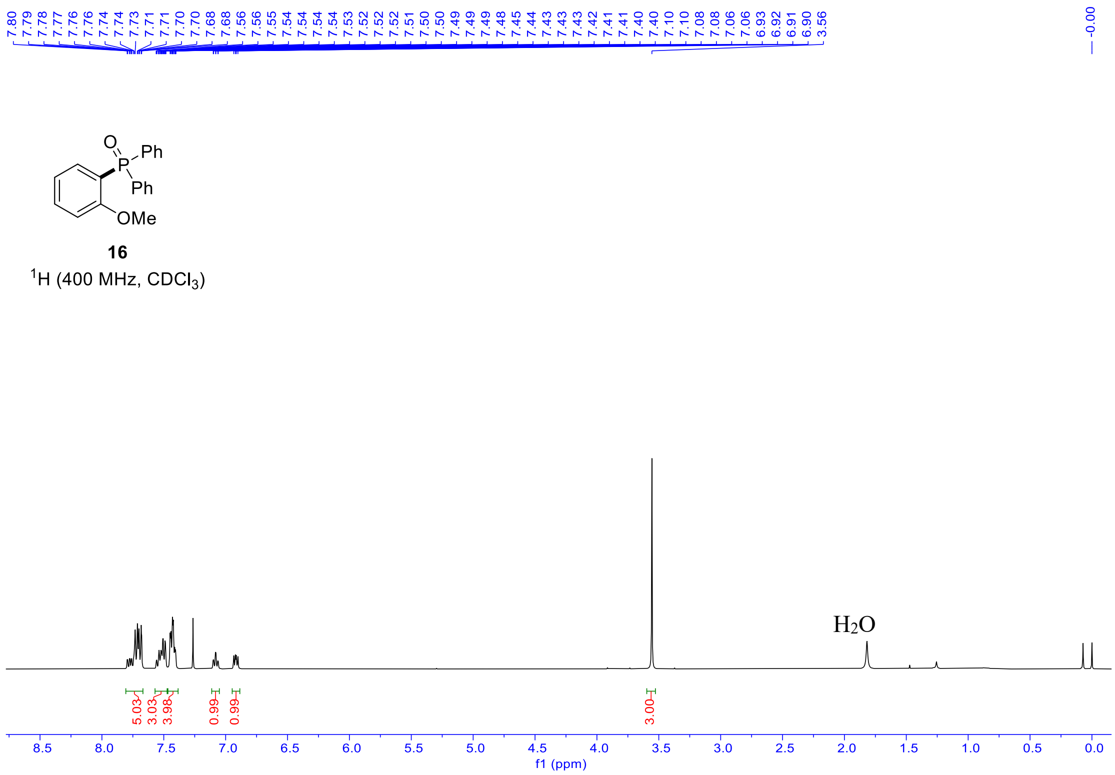

Supplementary Figure 75. **^{1}H NMR** of compound **16**

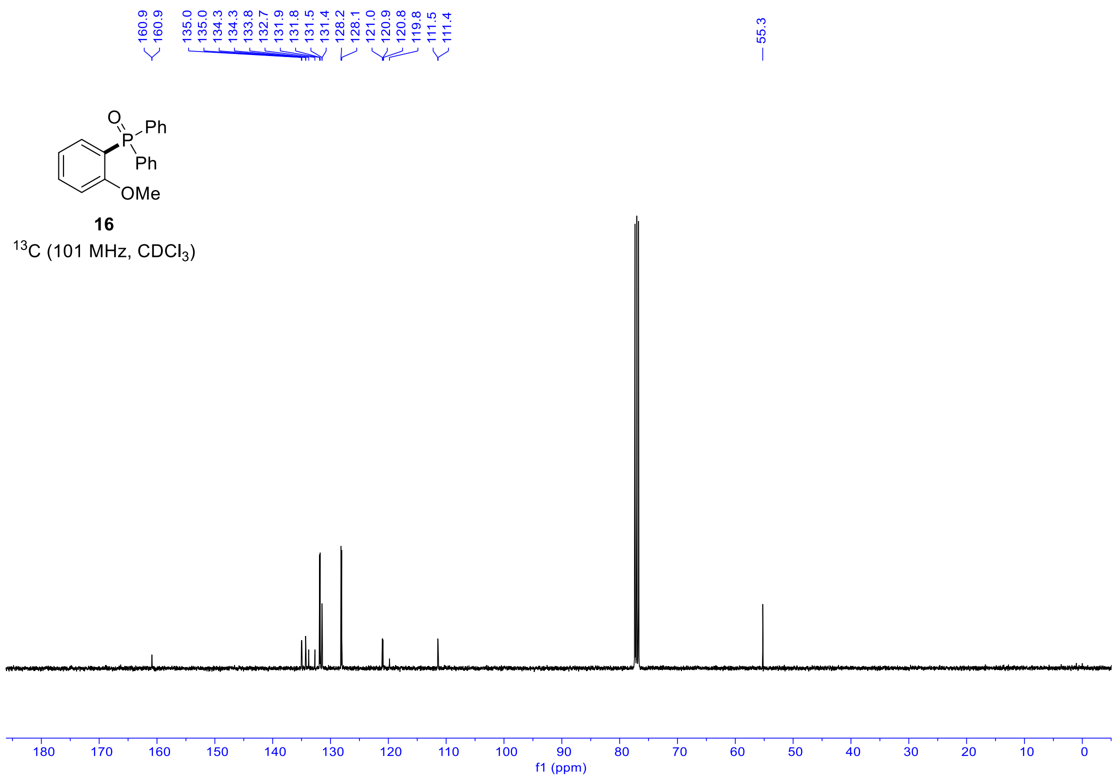

Supplementary Figure 76. ^{13}C NMR of compound **16**

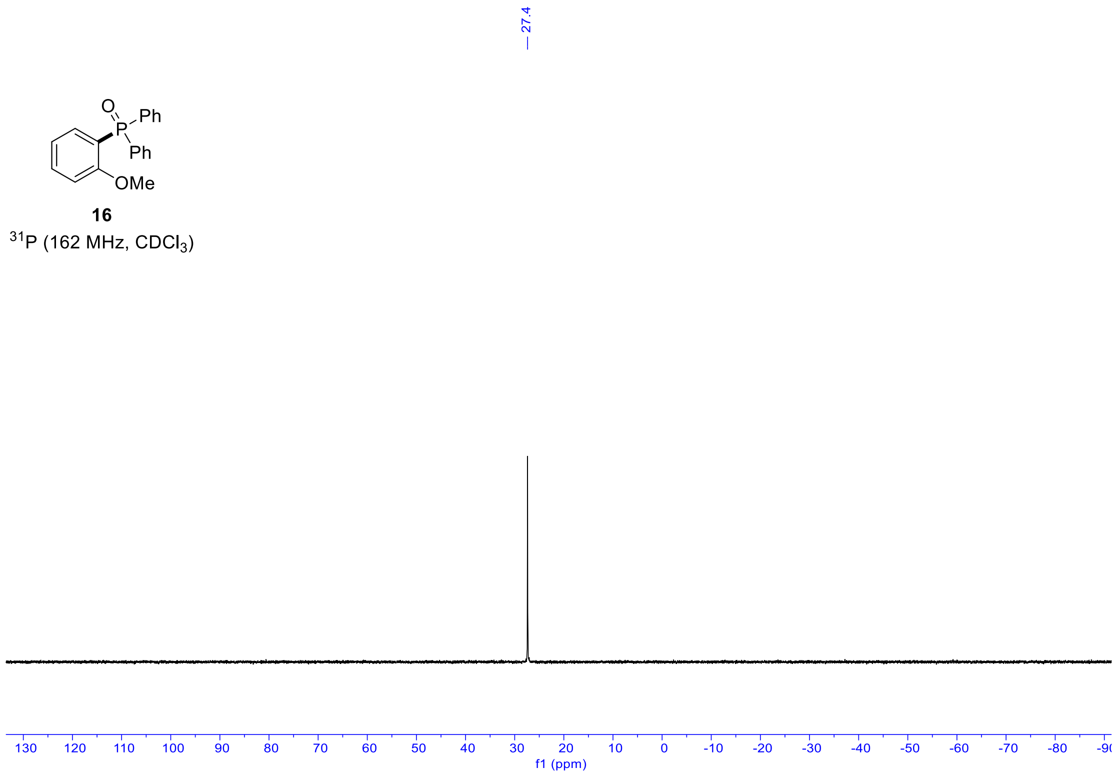

Supplementary Figure 77. ^{31}P NMR of compound **16**

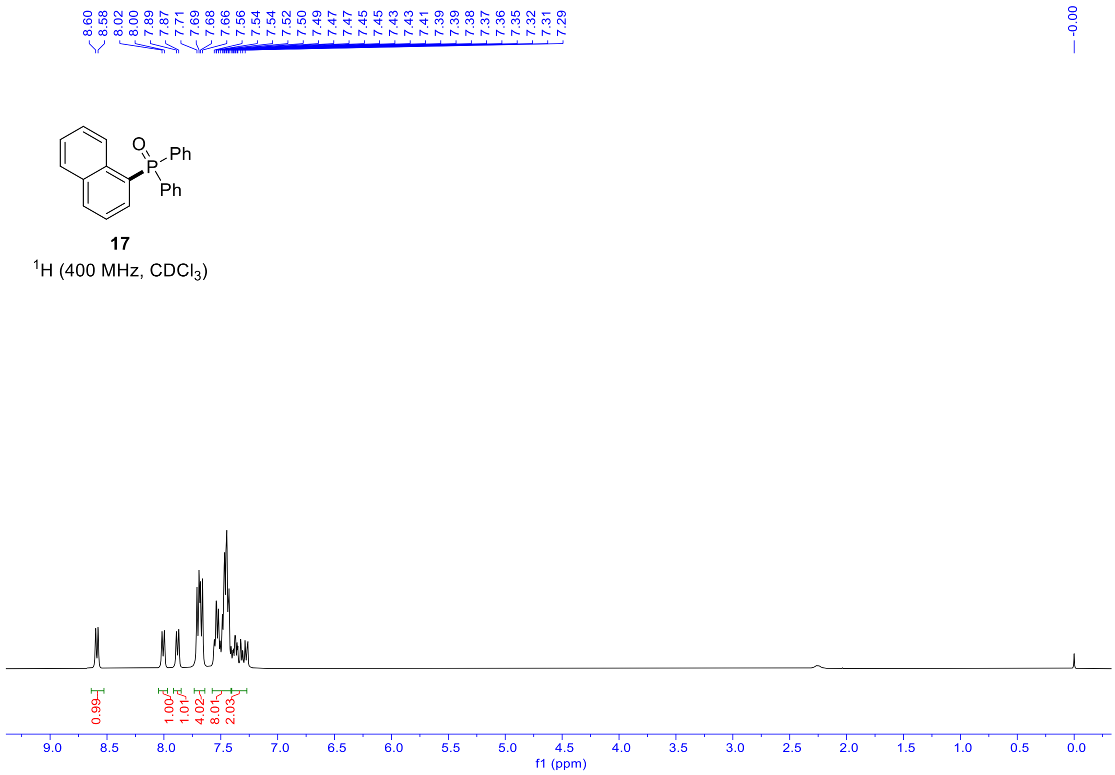

Supplementary Figure 78. ^{1}**H NMR** of compound **17**

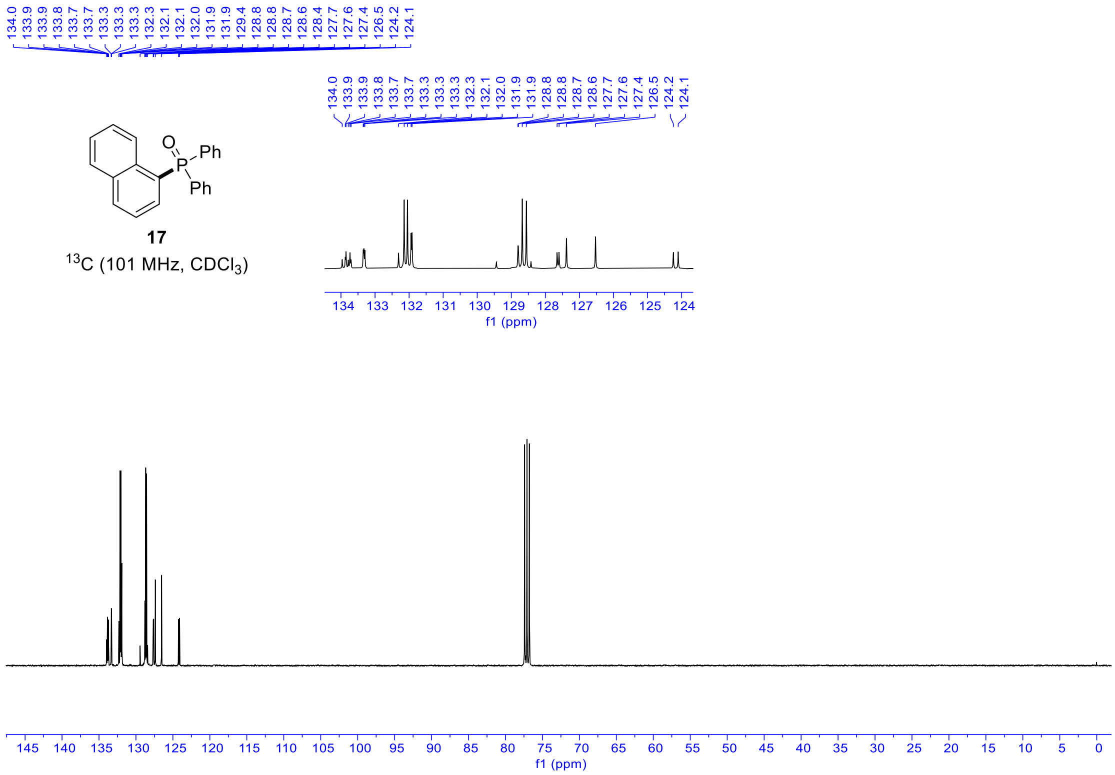

Supplementary Figure 79. ^{13}**C NMR** of compound **17**

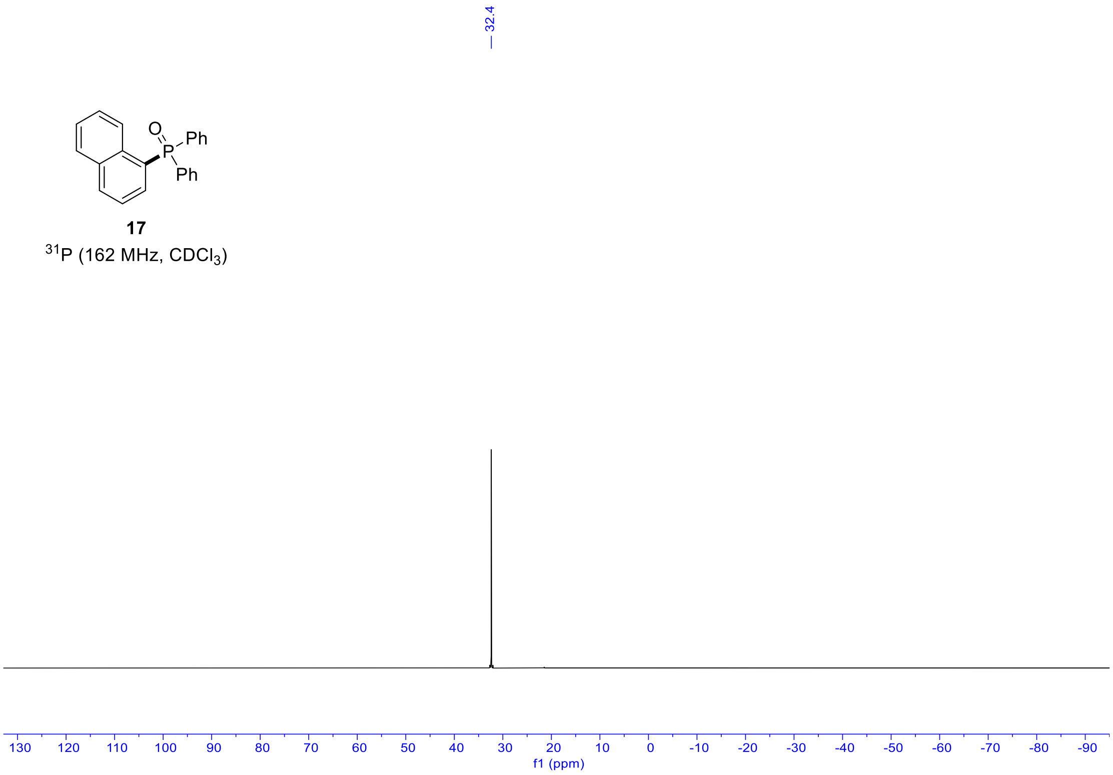

Supplementary Figure 80. ^{31}**P NMR** of compound **17**

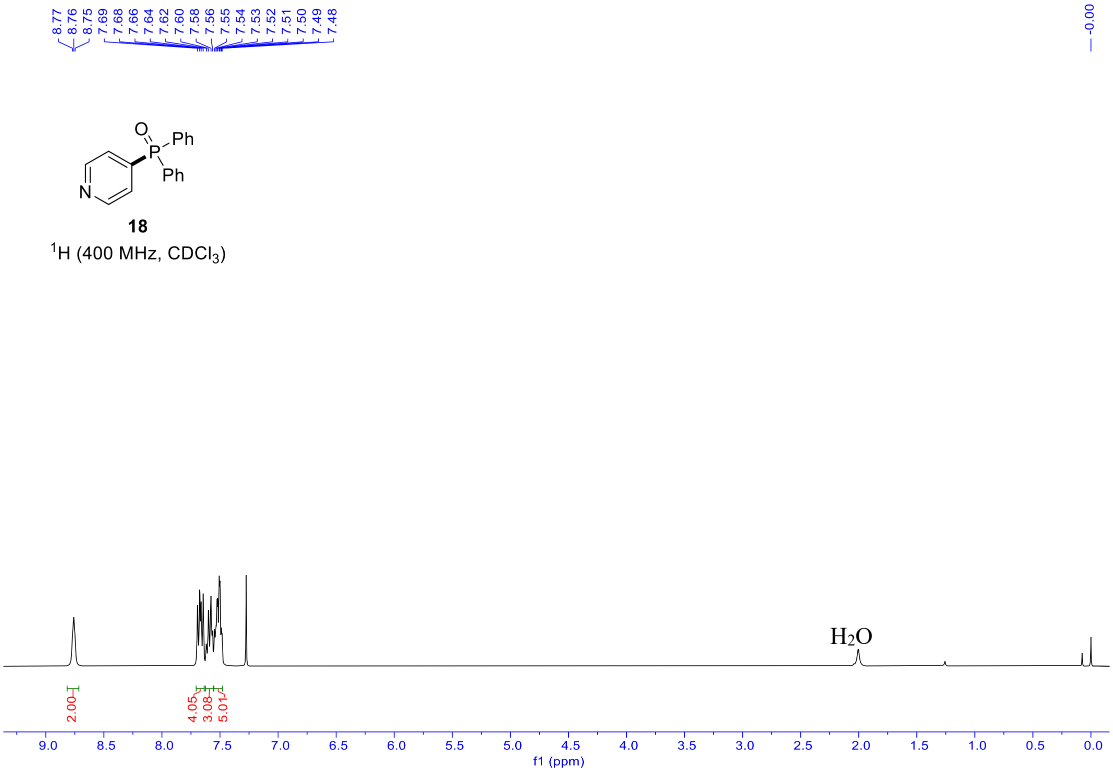

Supplementary Figure 81. ^{1}**H NMR** of compound **18**

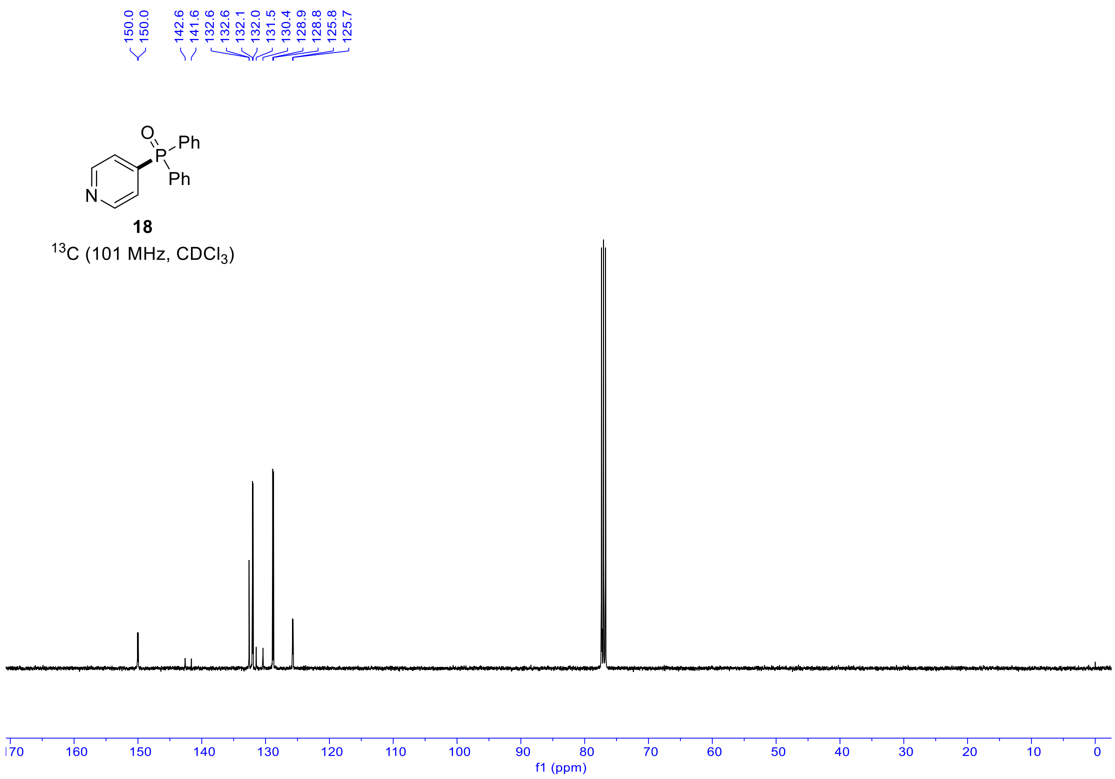

Supplementary Figure 82. ^{13}**C NMR** of compound **18**

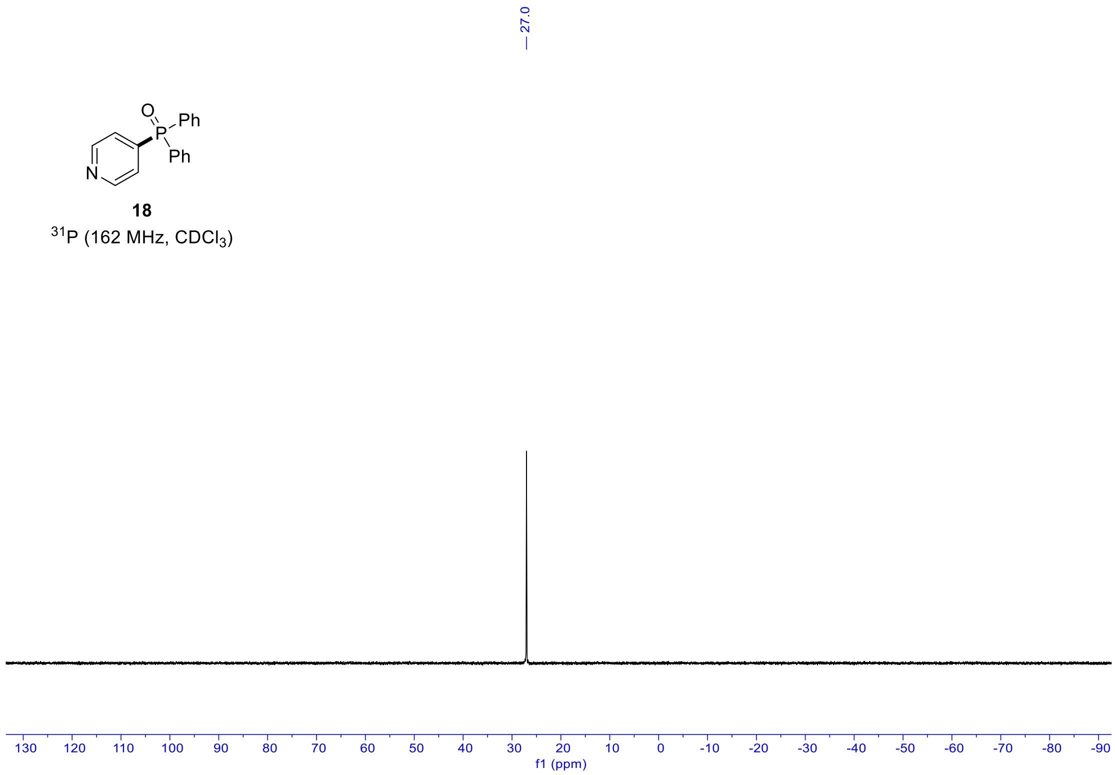

Supplementary Figure 83. ^{31}**P NMR** of compound **18**

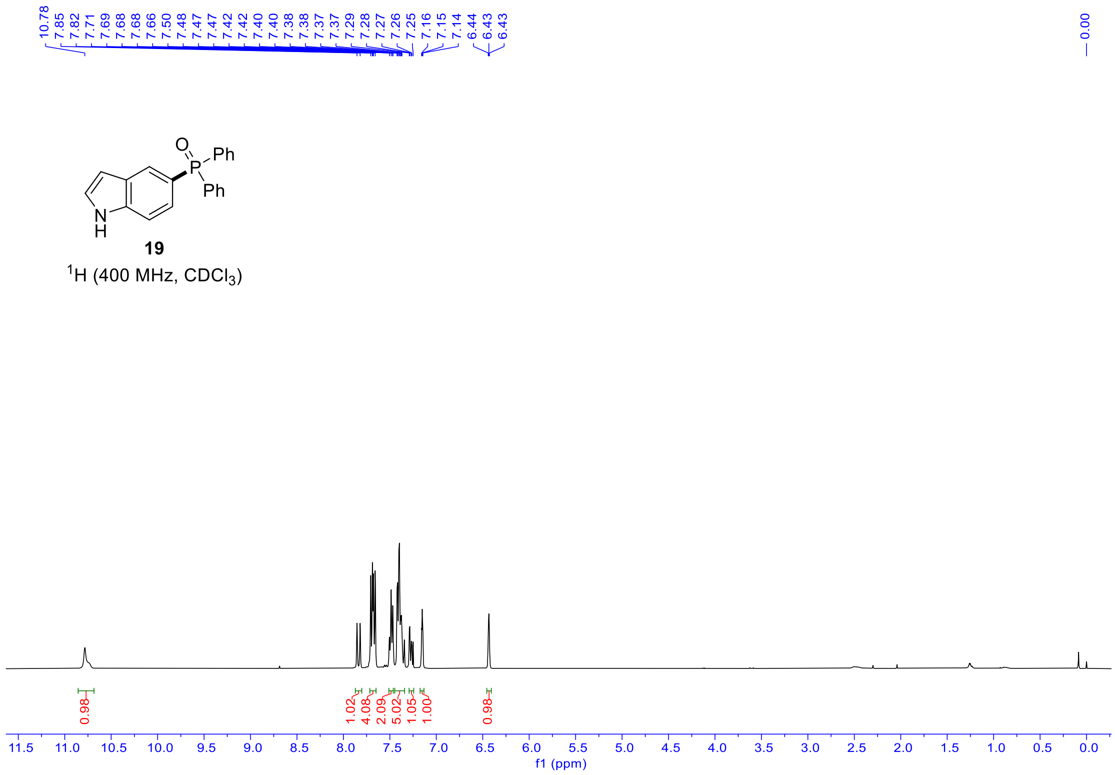

Supplementary Figure 84. **^{1}H NMR** of compound **19**

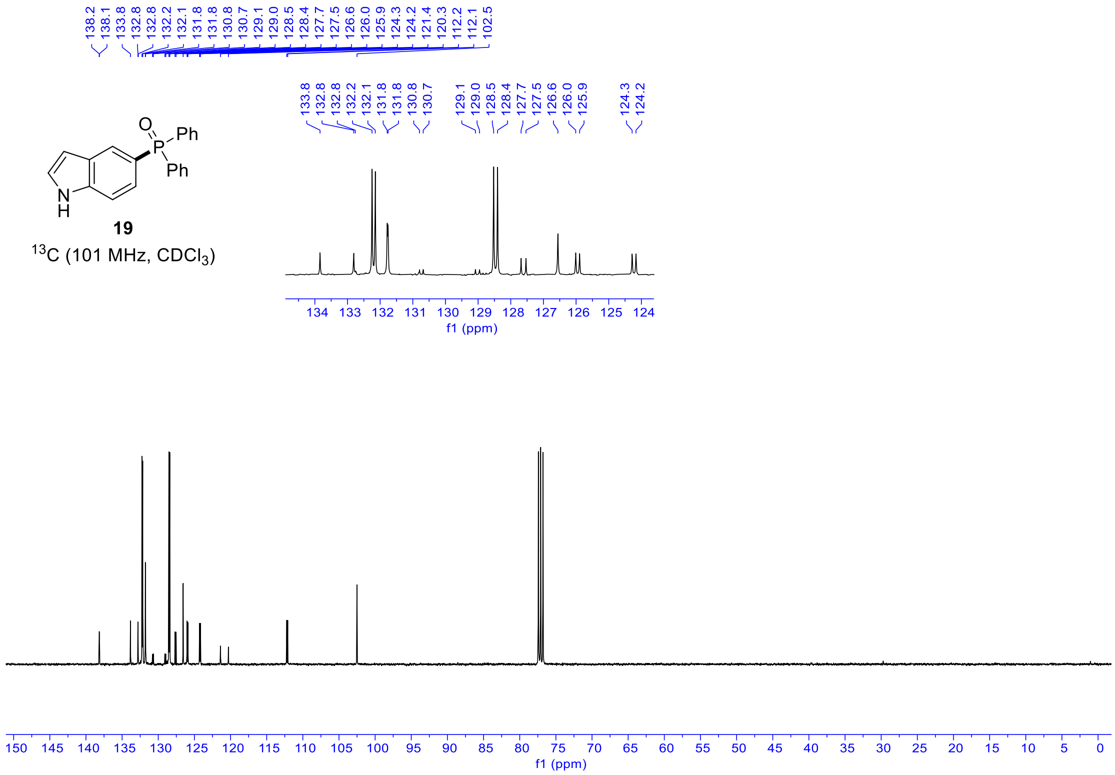

Supplementary Figure 85. **^{13}C NMR** of compound **19**

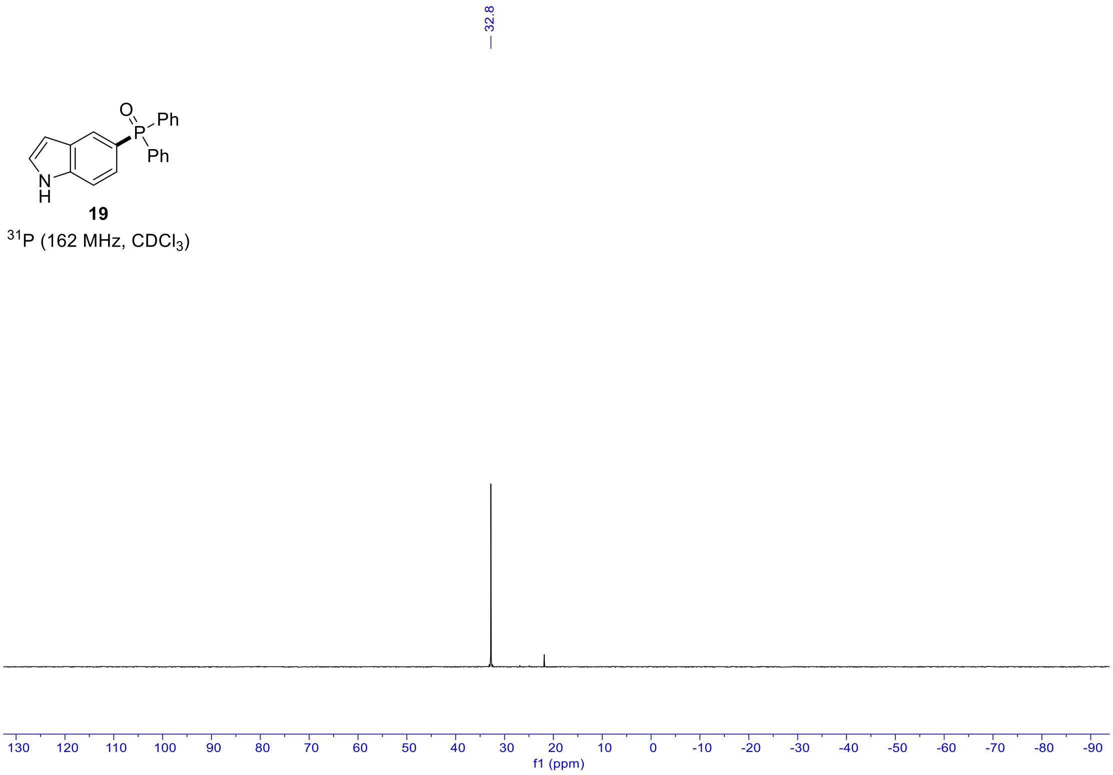

Supplementary Figure 86. ^{31}P NMR of compound **19**

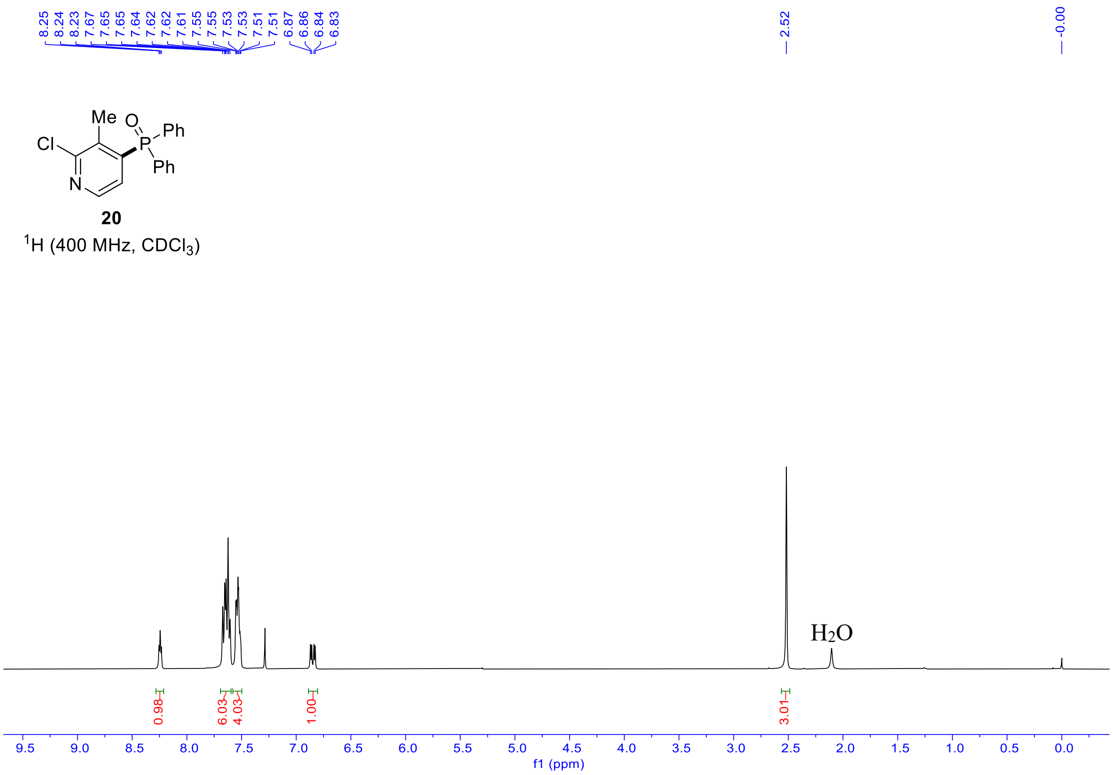

Supplementary Figure 87. ^{1}H NMR of compound **20**

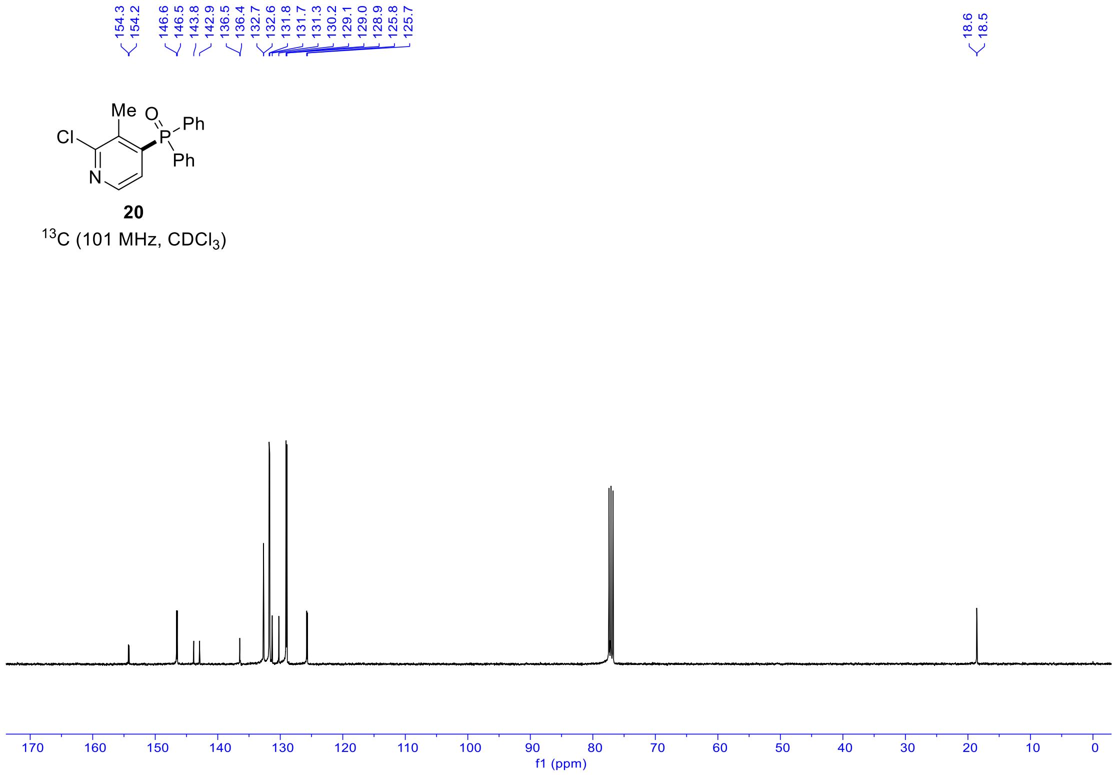

Supplementary Figure 88. ^{13}C NMR of compound **20**

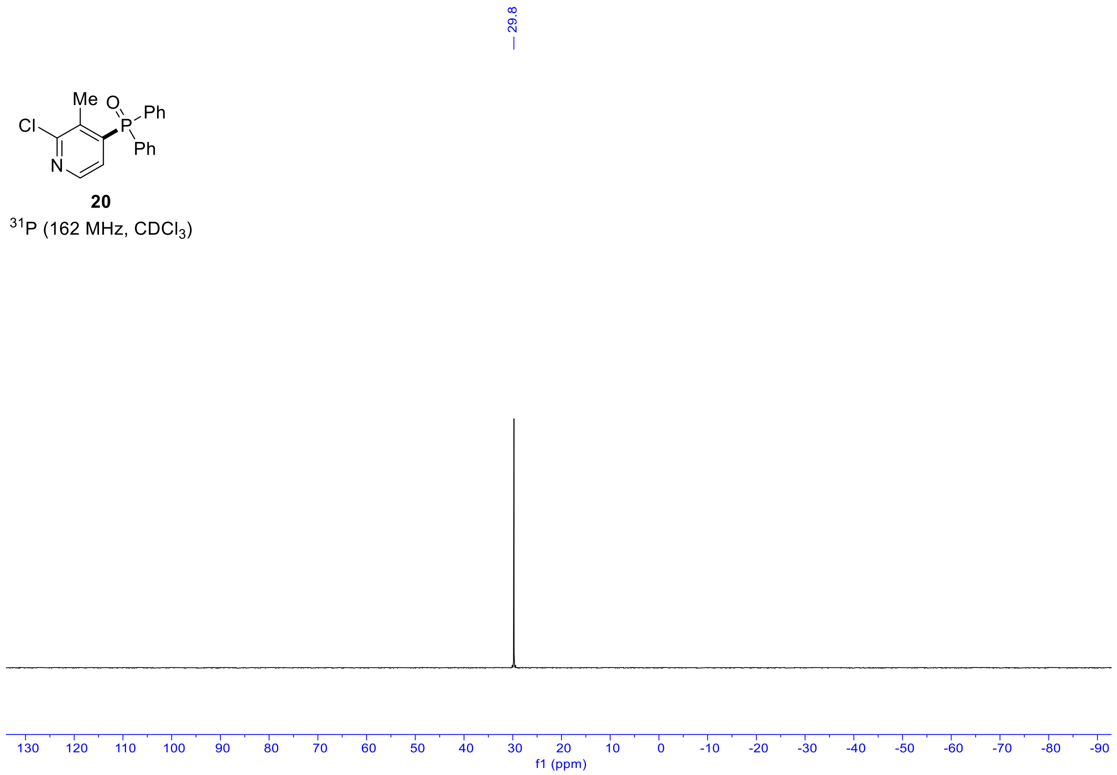

Supplementary Figure 89. ^{31}P NMR of compound **20**

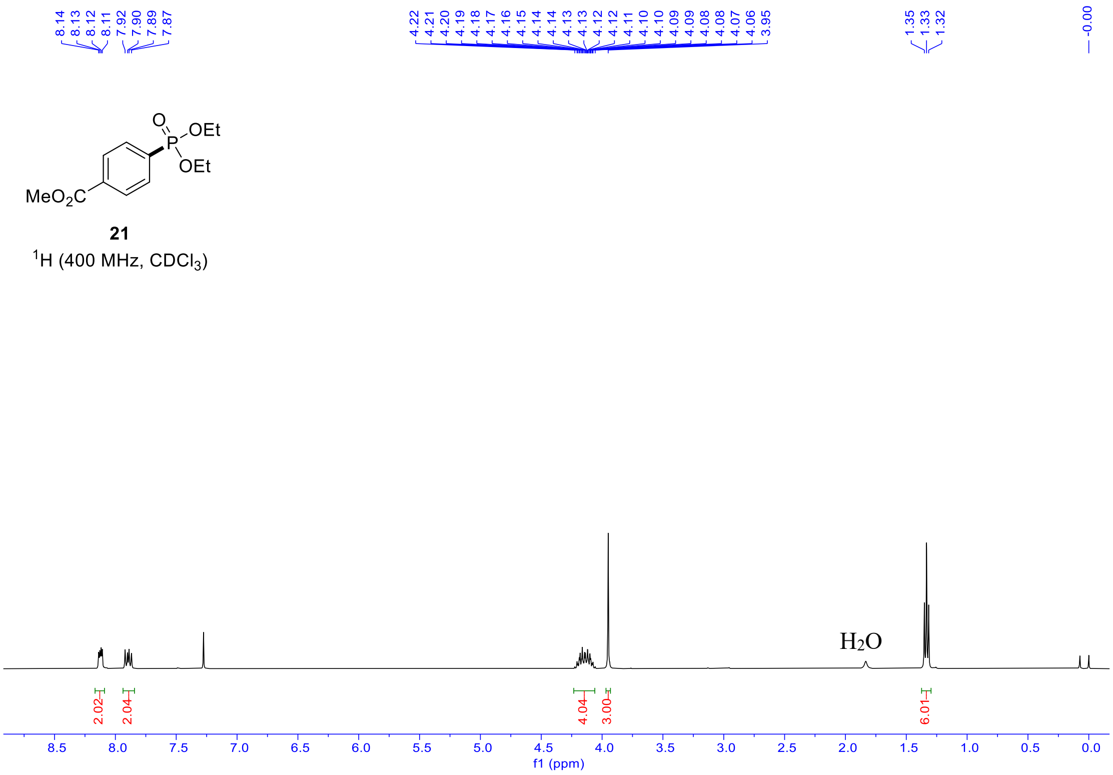

Supplementary Figure 90. ^{1}**H NMR** of compound **21**

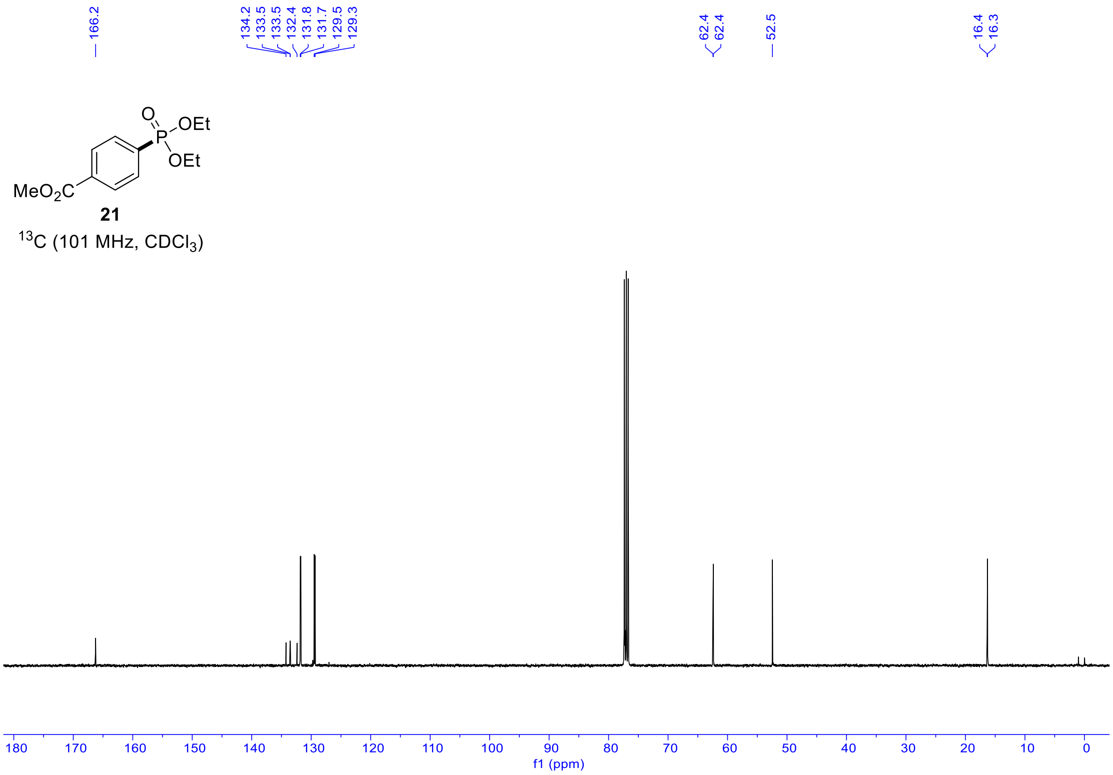

Supplementary Figure 91. ^{13}**C NMR** of compound **21**

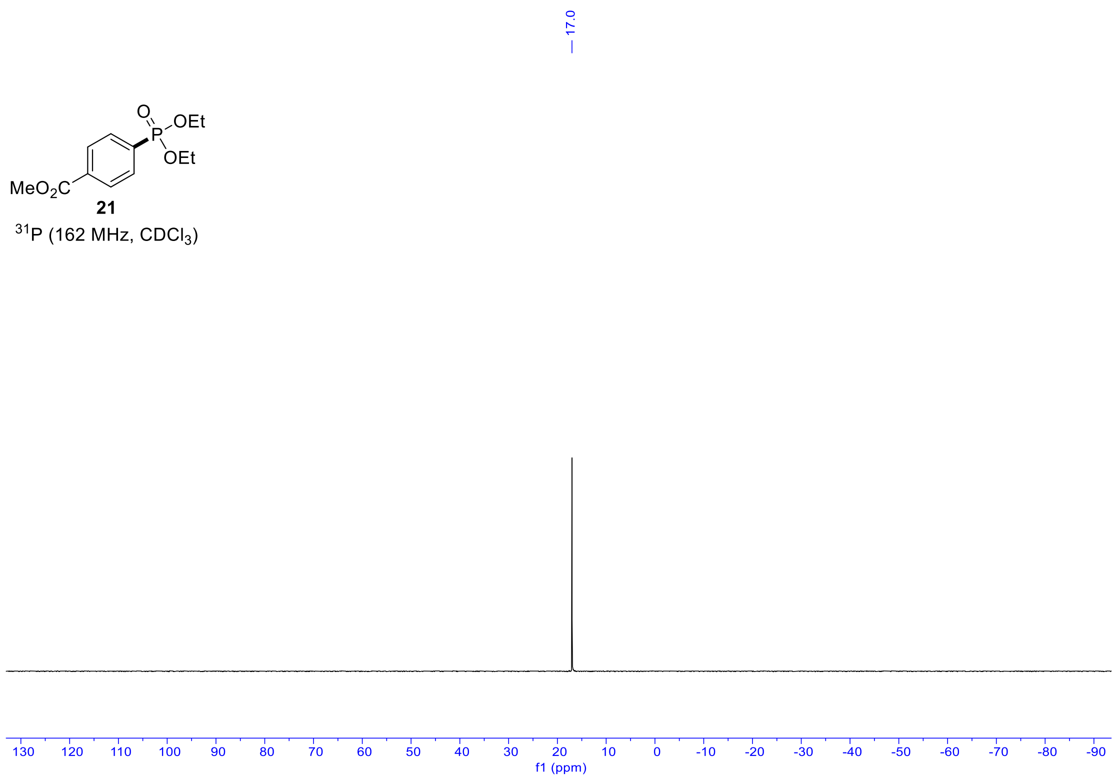

Supplementary Figure 92. ^{31}**P NMR** of compound **21**

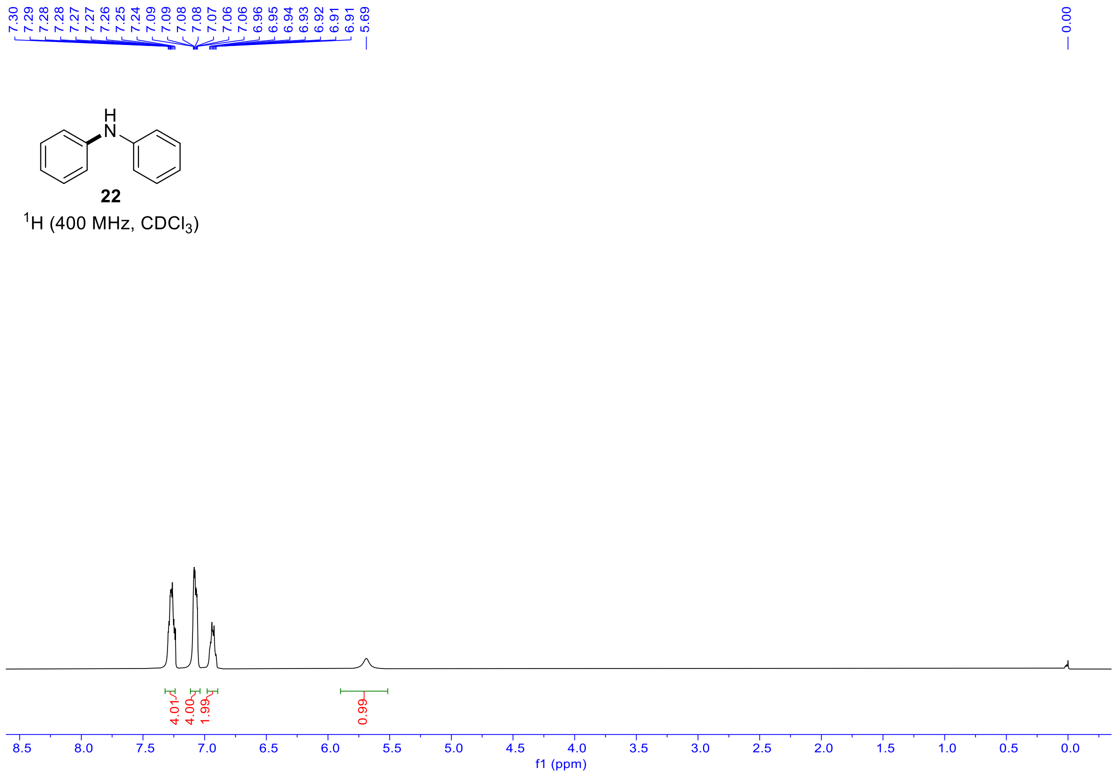

Supplementary Figure 93. ^{1}**H NMR** of compound **22**

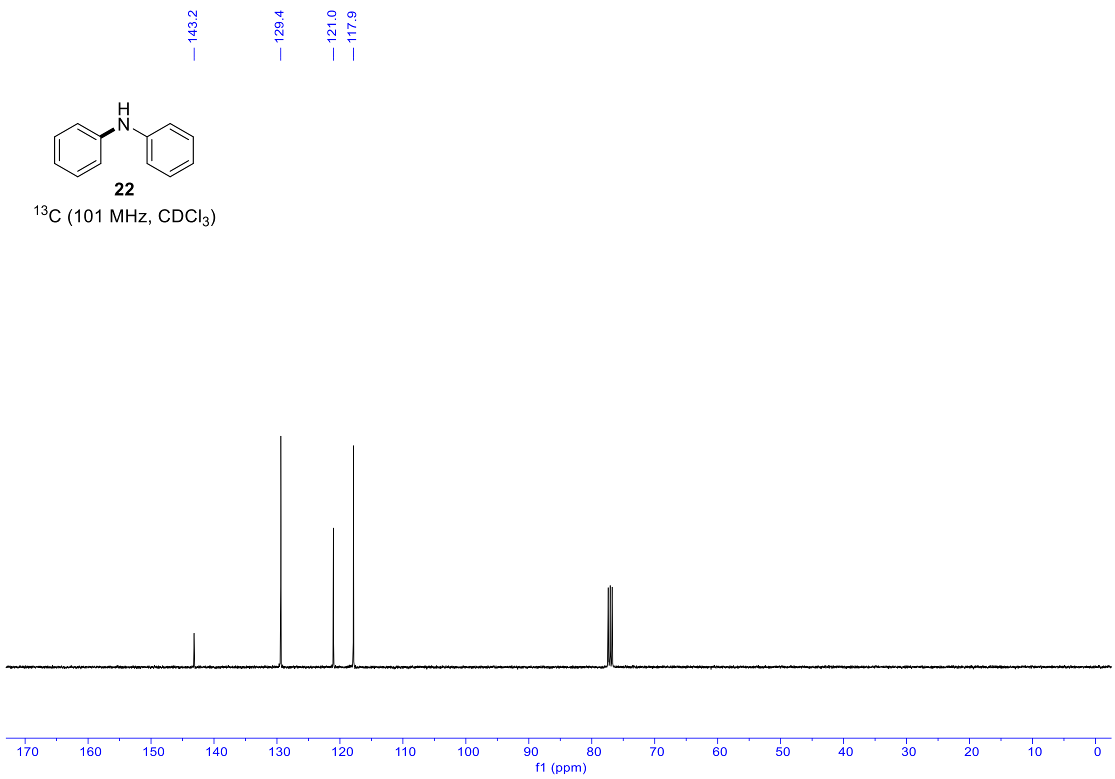

Supplementary Figure 94. ^{13}C **NMR** of compound **22**

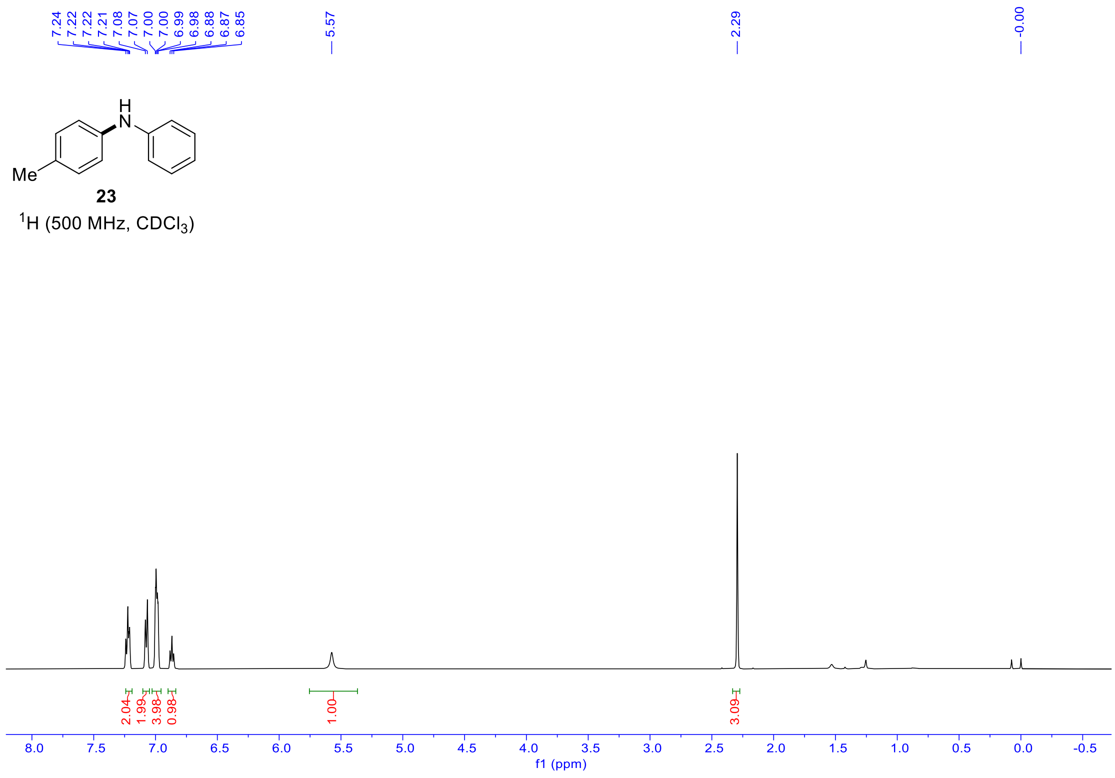

Supplementary Figure 95. ^{1}H **NMR** of compound **23**

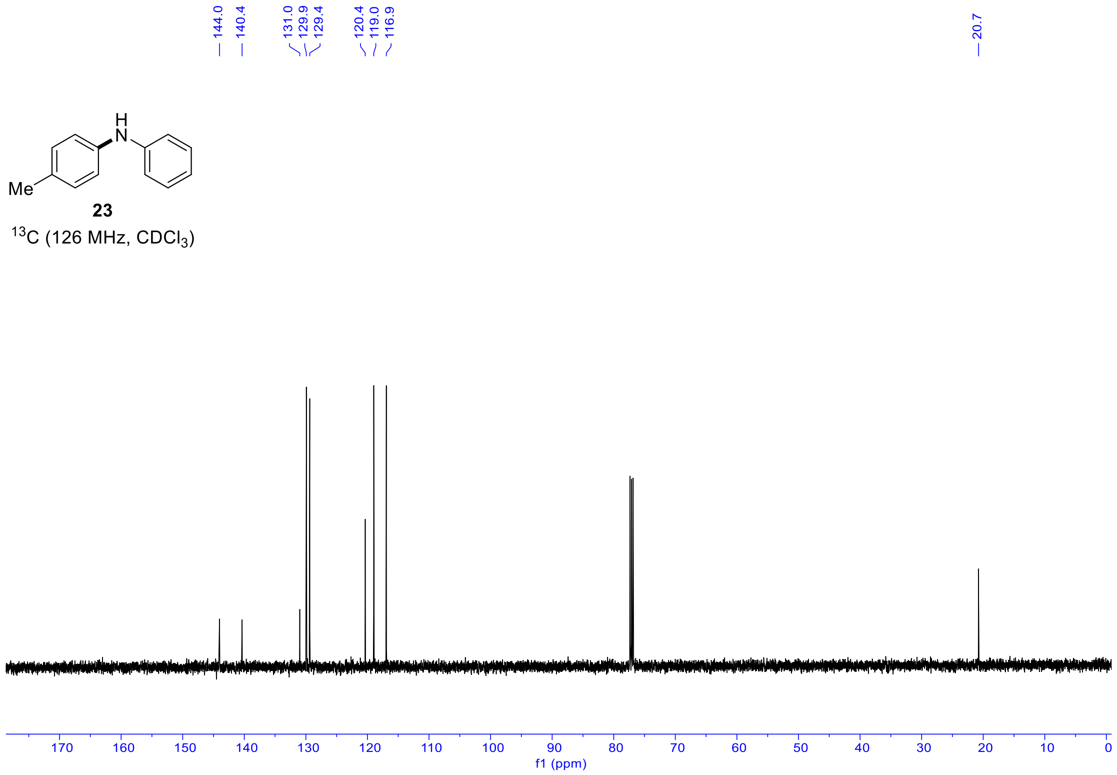

Supplementary Figure 96. ^{13}**C NMR** of compound **23**

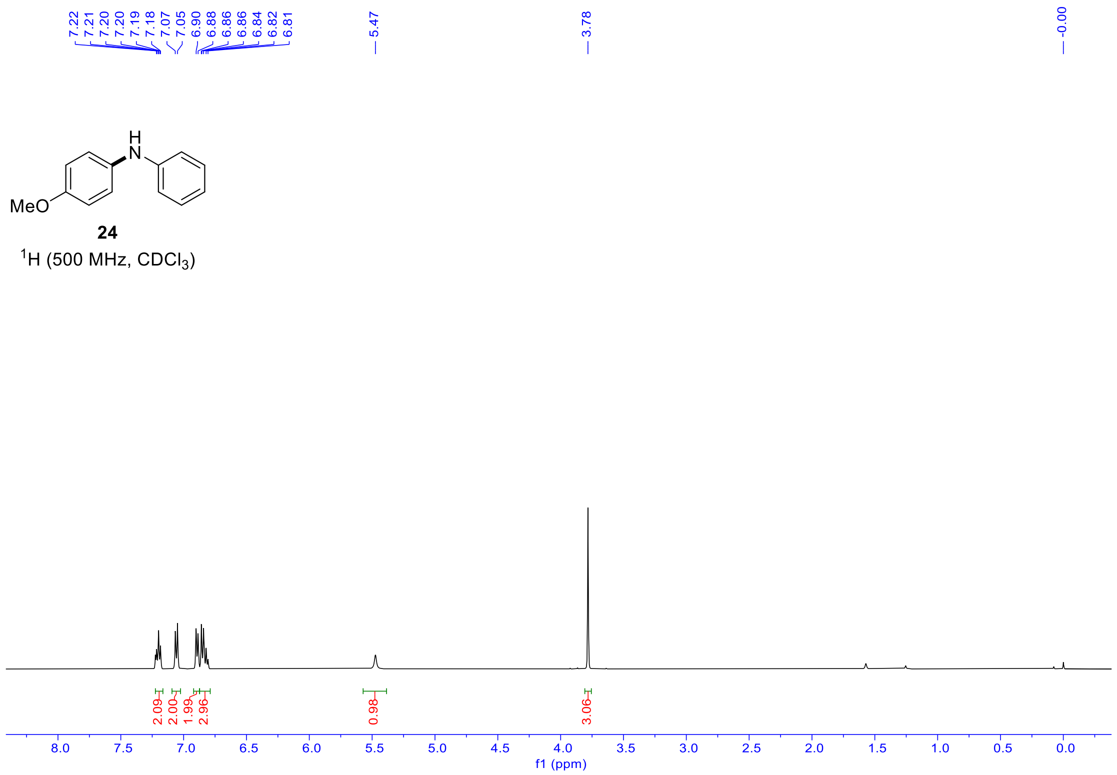

Supplementary Figure 97. ^{1}**H NMR** of compound **24**

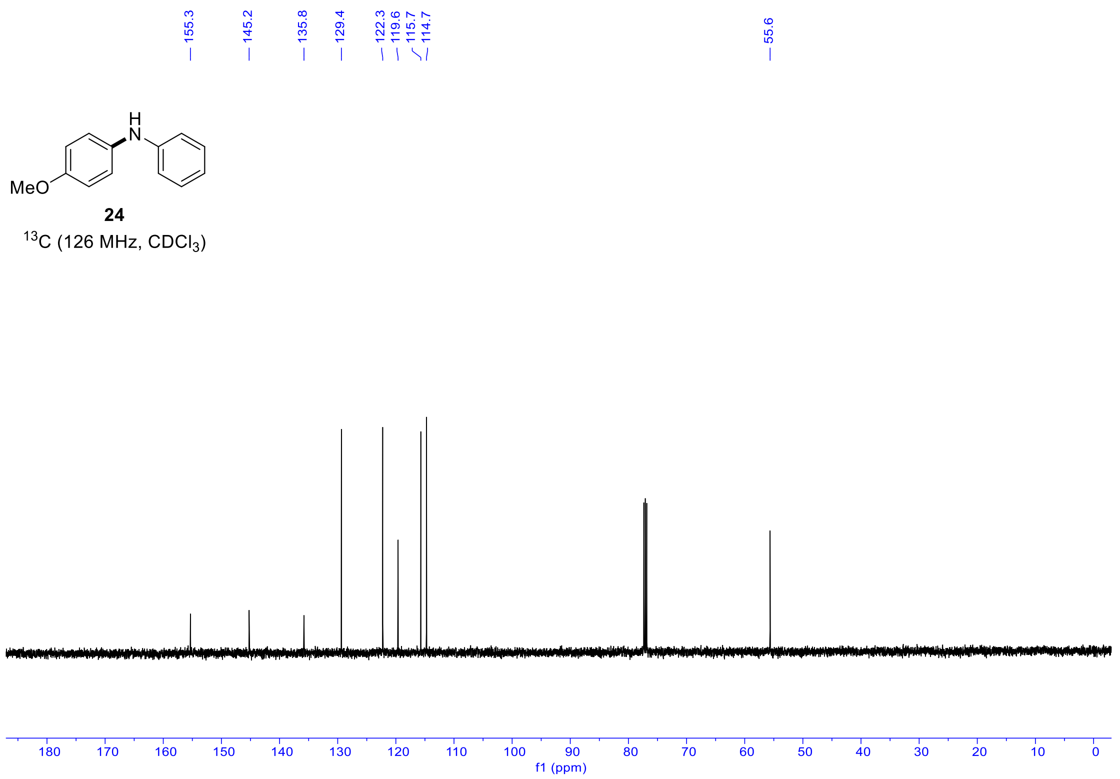

Supplementary Figure 98. ^{13}C NMR of compound **24**

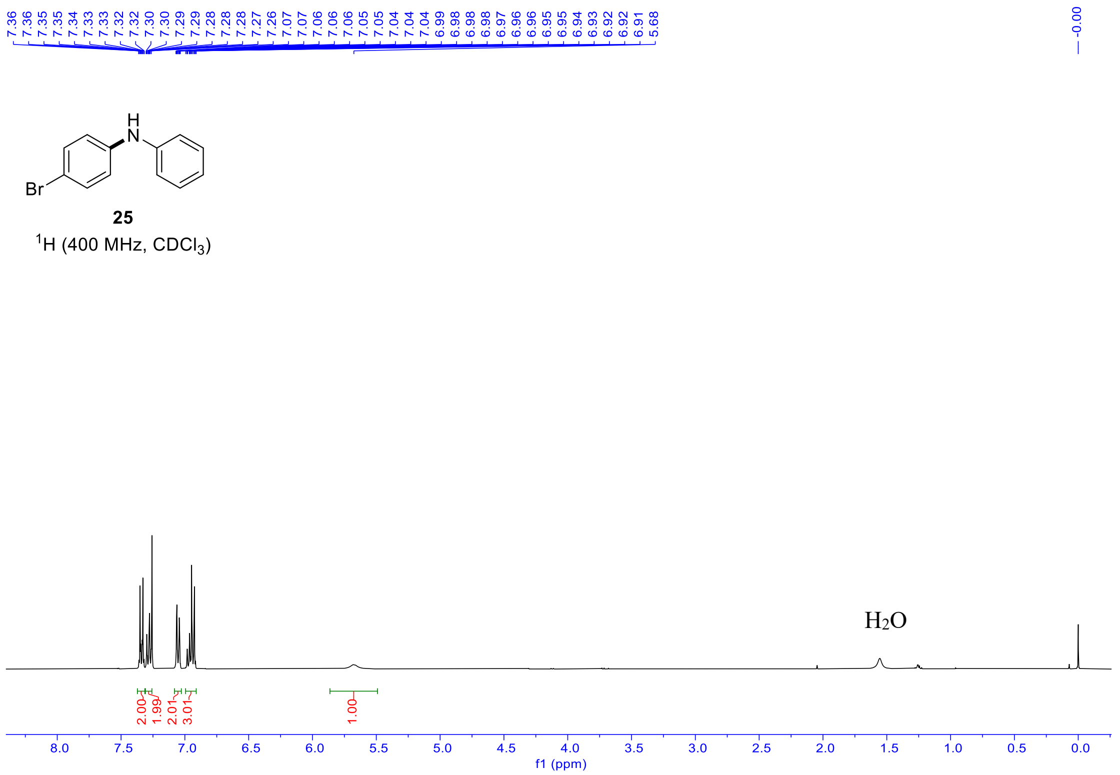

Supplementary Figure 99. ^{1}H NMR of compound **25**

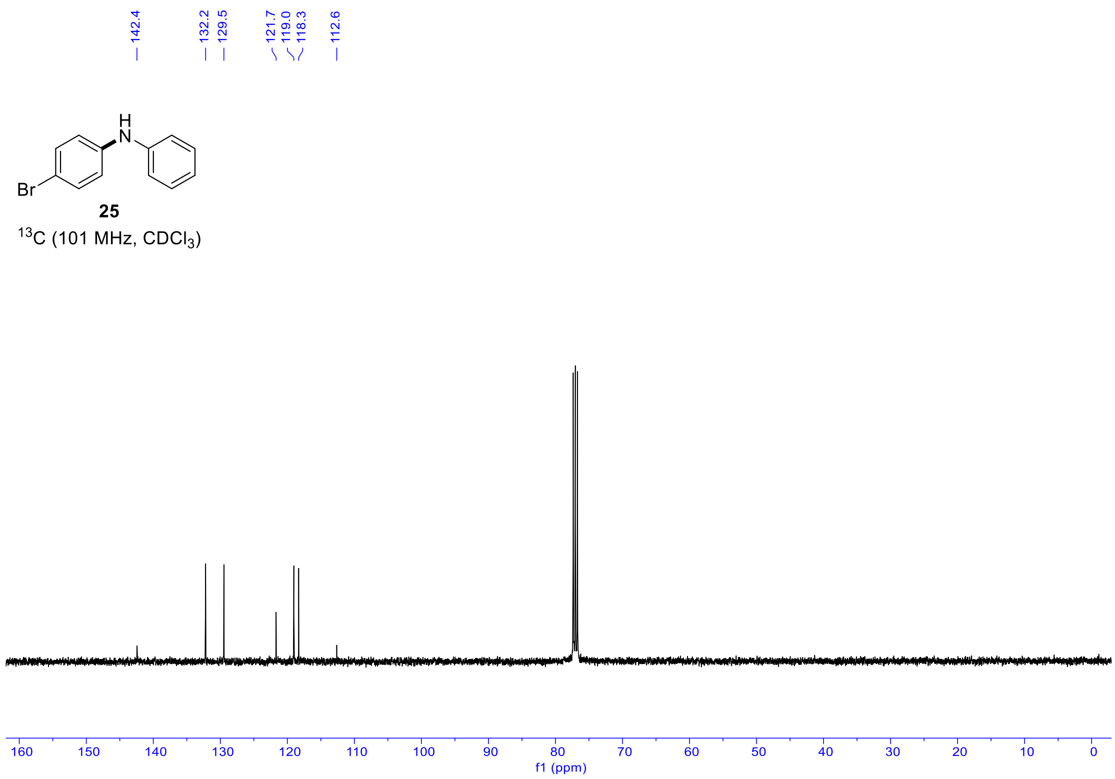

Supplementary Figure 100. ^{13}**C NMR** of compound **25**

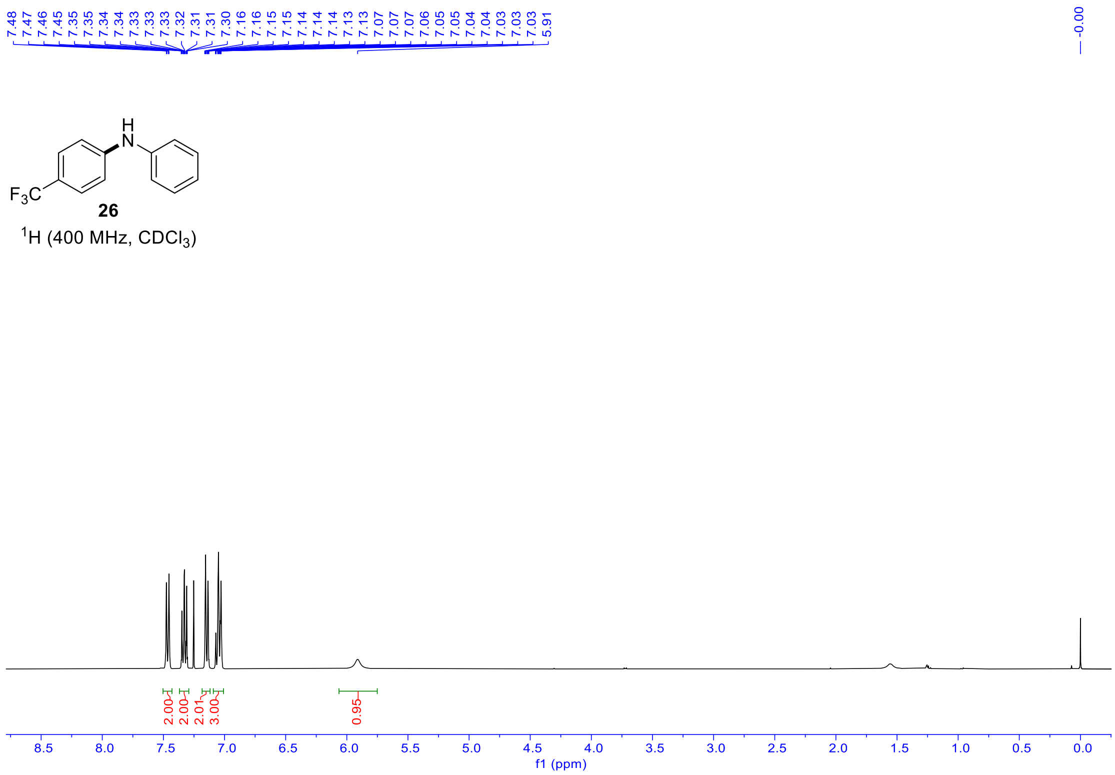

Supplementary Figure 101. ^{1}**H NMR** of compound **26**

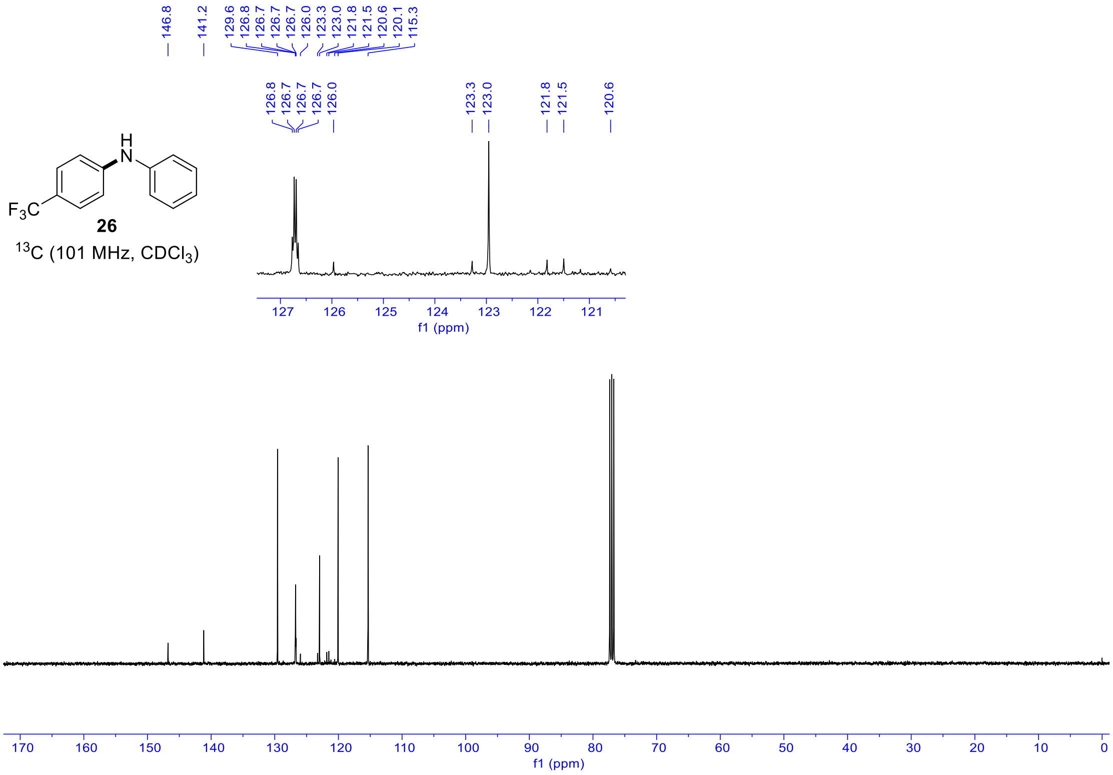

Supplementary Figure 102. ^{13}**C NMR** of compound **26**

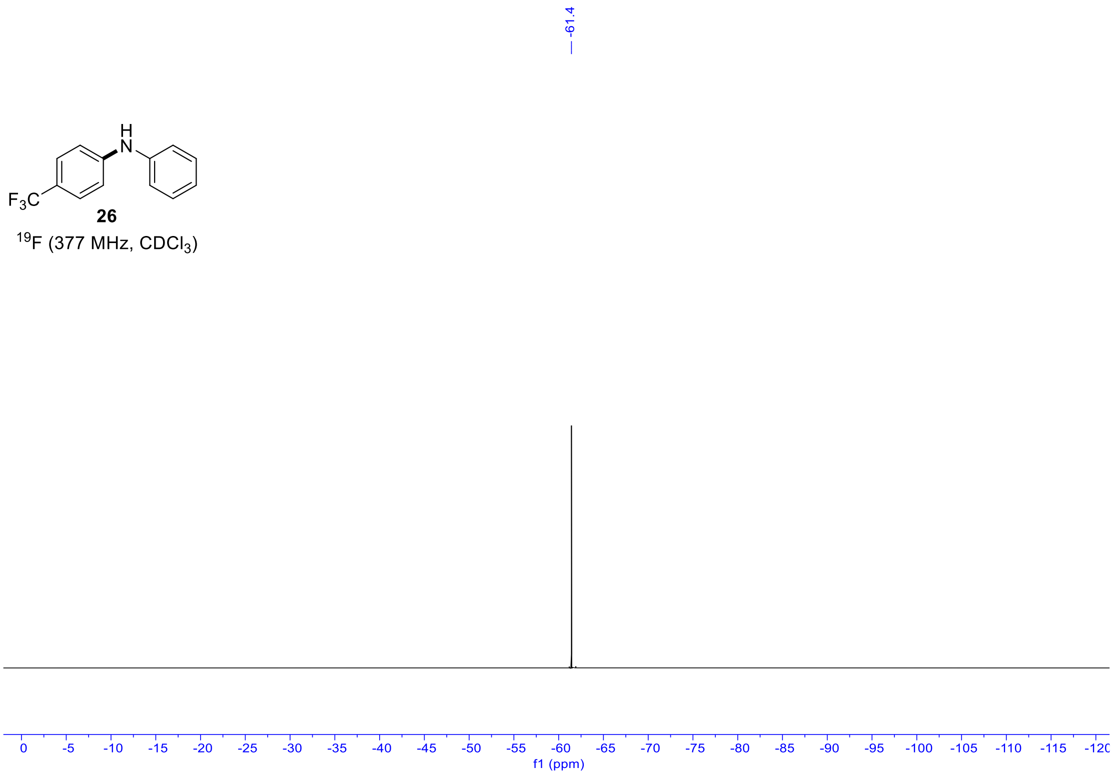

Supplementary Figure 103. ^{19}**F NMR** of compound **26**

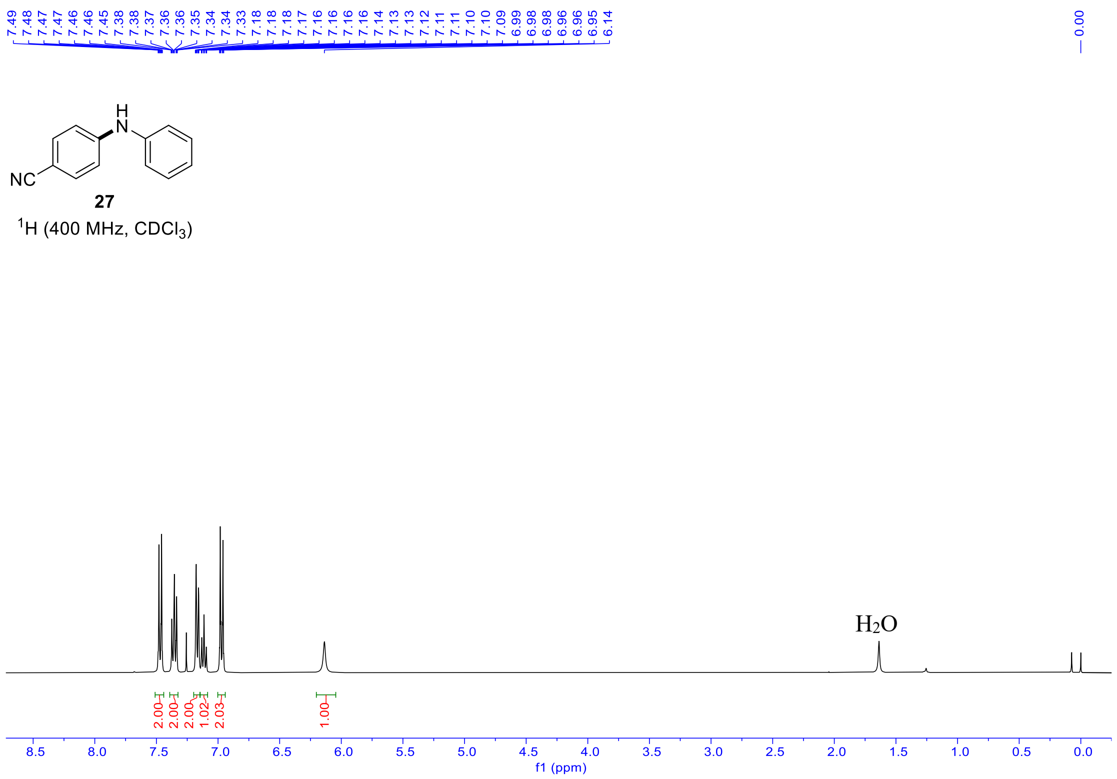

Supplementary Figure 104. ^{1}H NMR of compound **27**

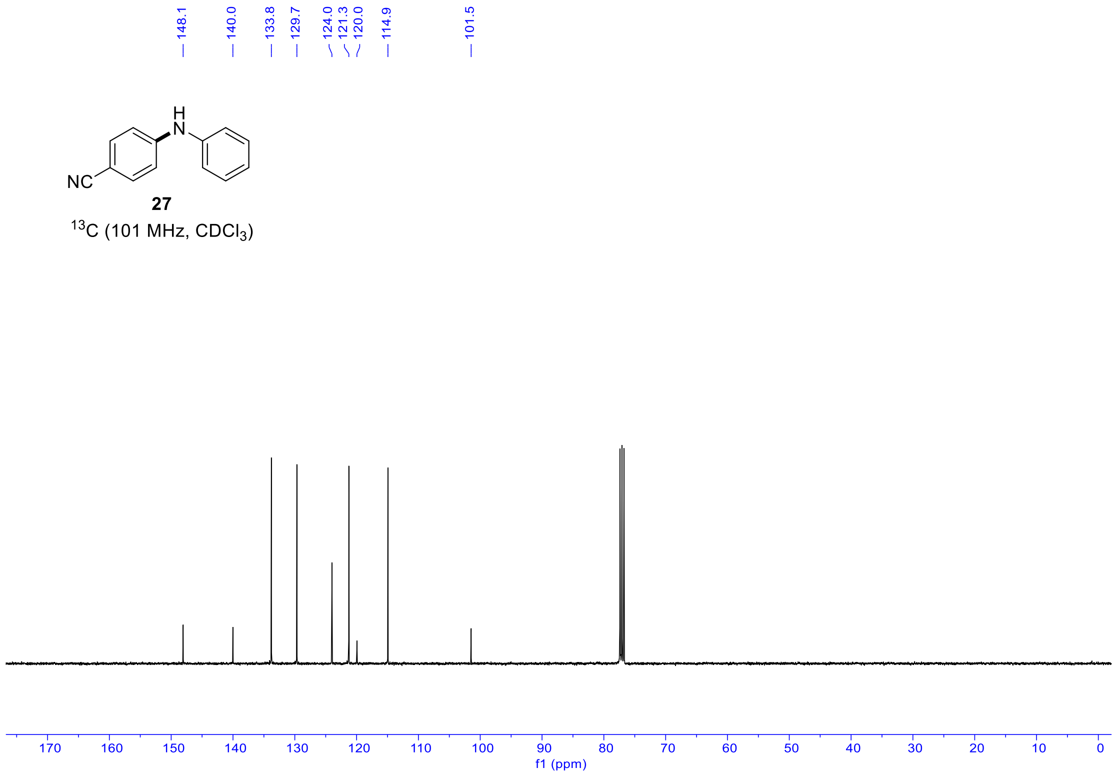

Supplementary Figure 105. ^{13}C NMR of compound **27**

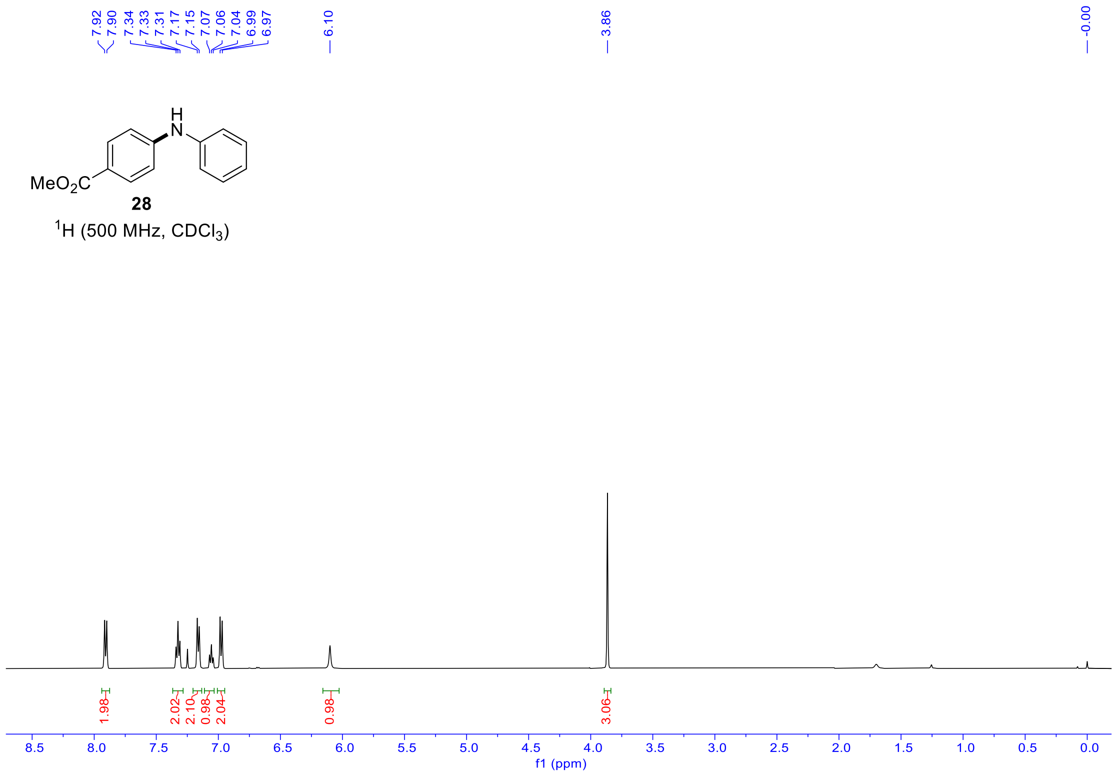

Supplementary Figure 106. ^{1}**H NMR** of compound **28**

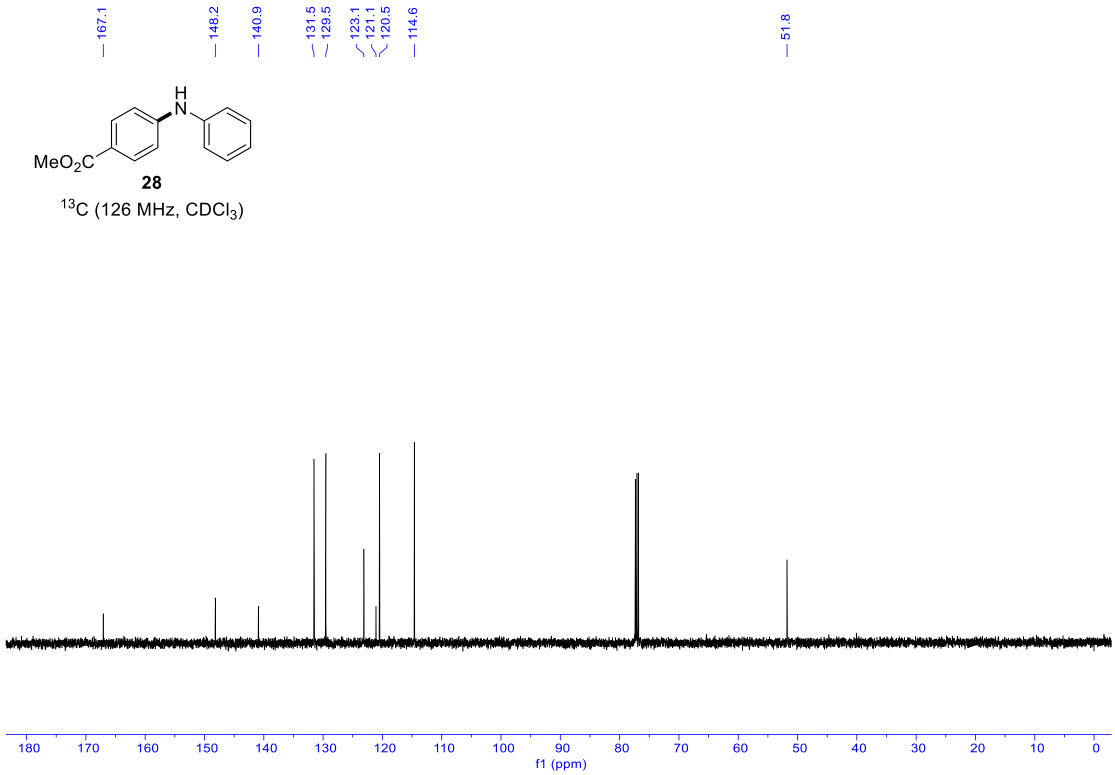

Supplementary Figure 107. ^{13}**C NMR** of compound **28**

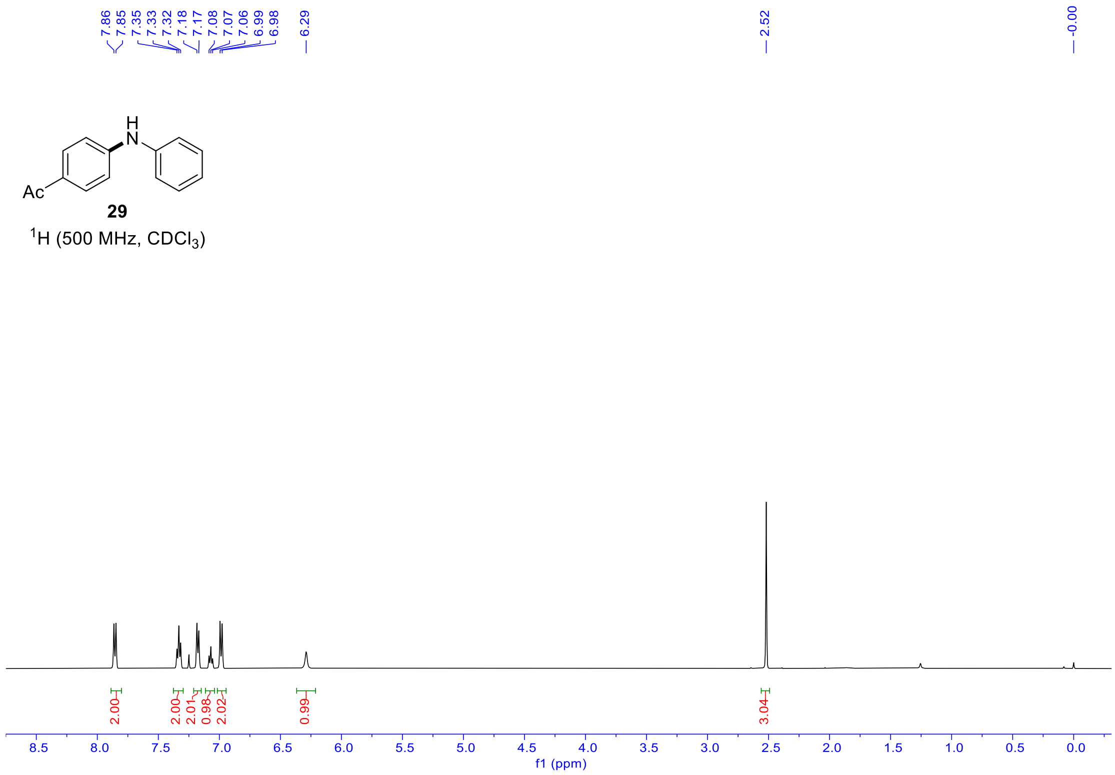

Supplementary Figure 108. **^{1}H NMR** of compound **29**

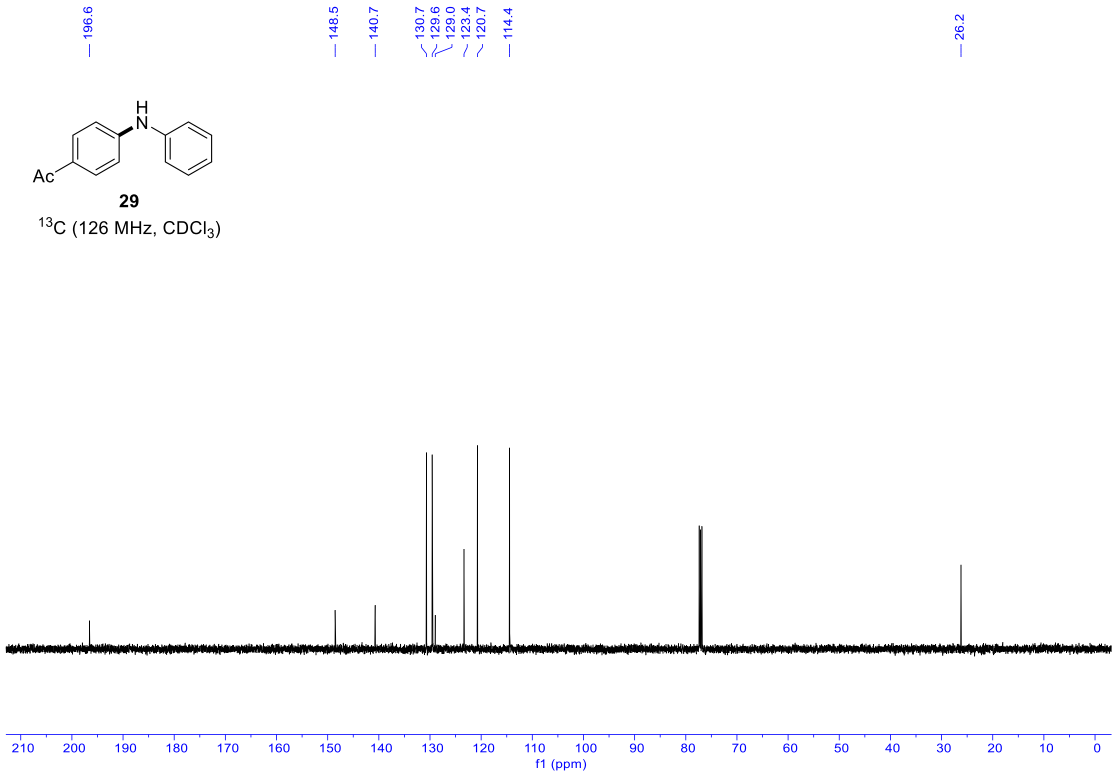

Supplementary Figure 109. **^{13}C NMR** of compound **29**

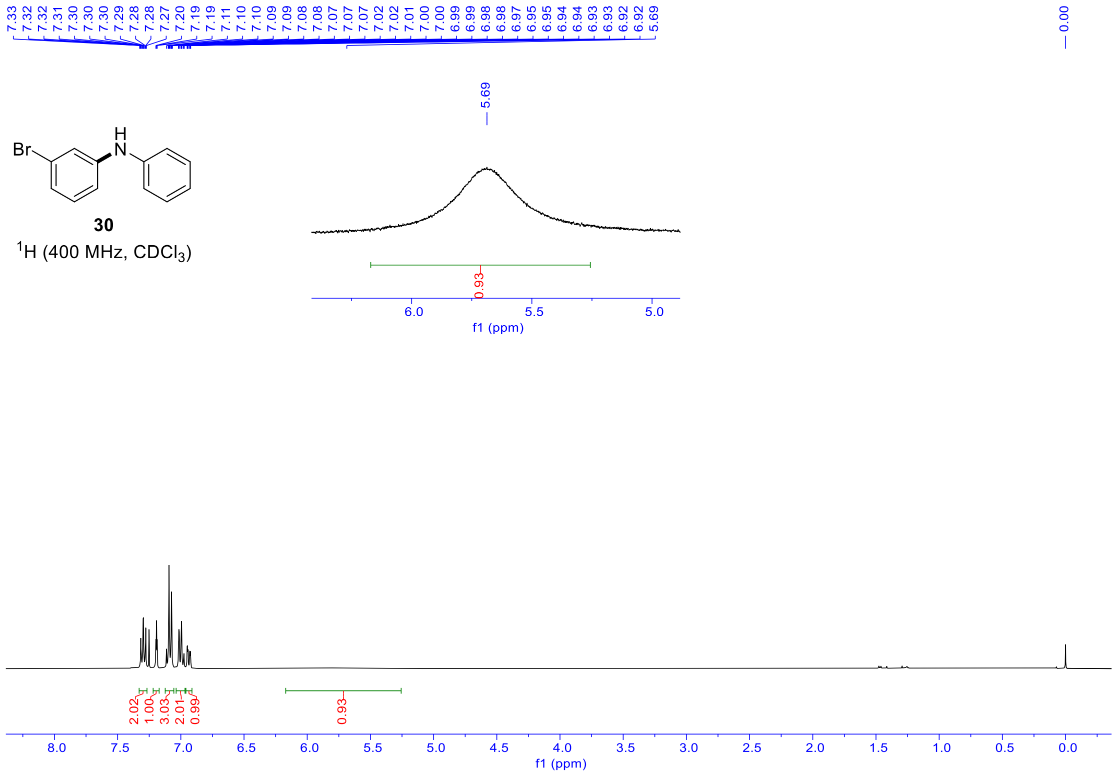

Supplementary Figure 110. ^{1}**H NMR** of compound **30**

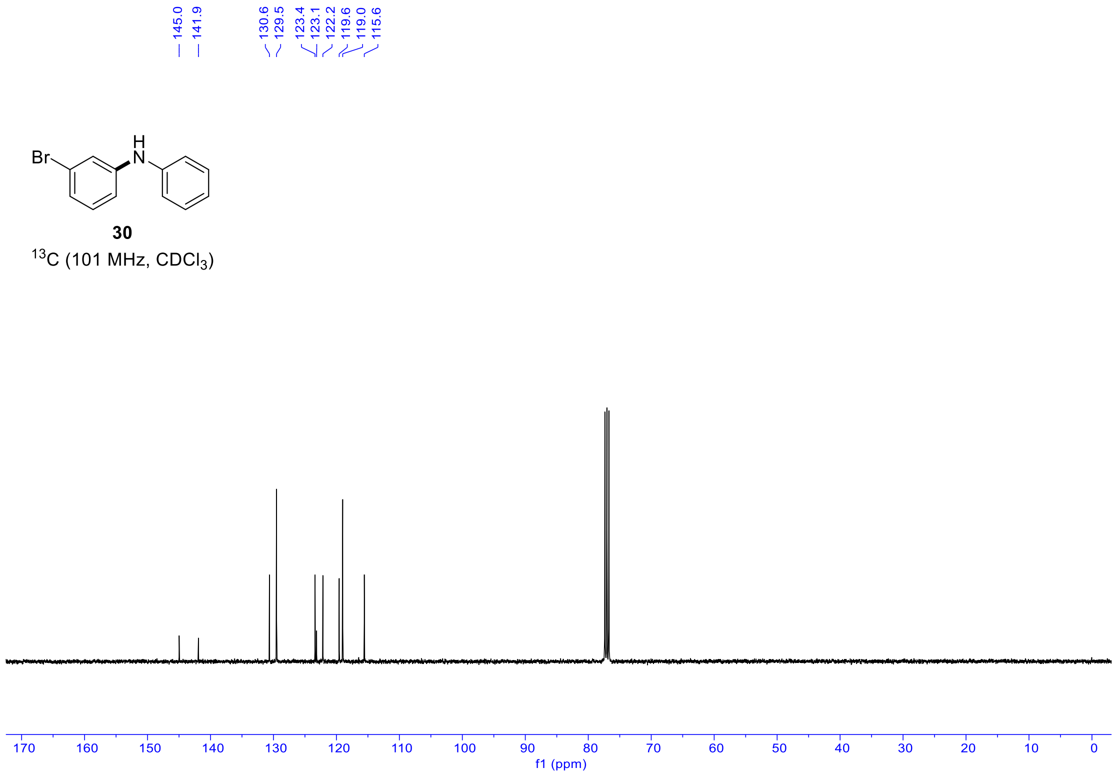

Supplementary Figure 111. ^{13}**C NMR** of compound **30**

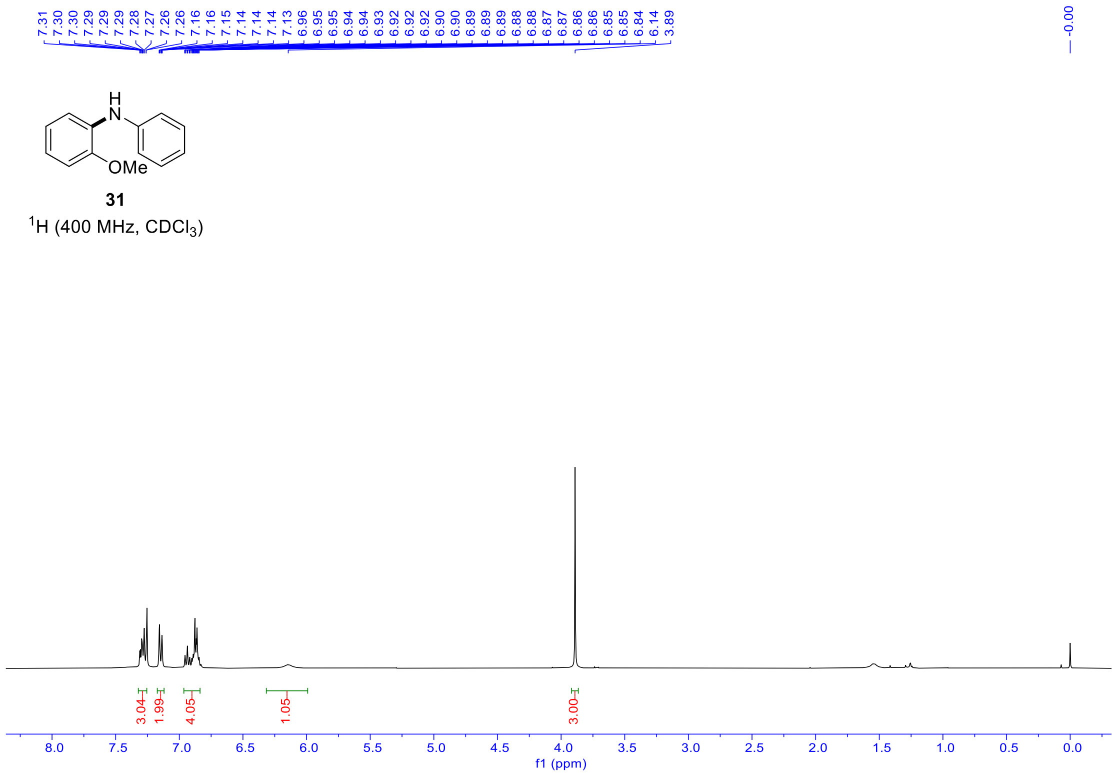

Supplementary Figure 112. ^{1}**H NMR** of compound **31**

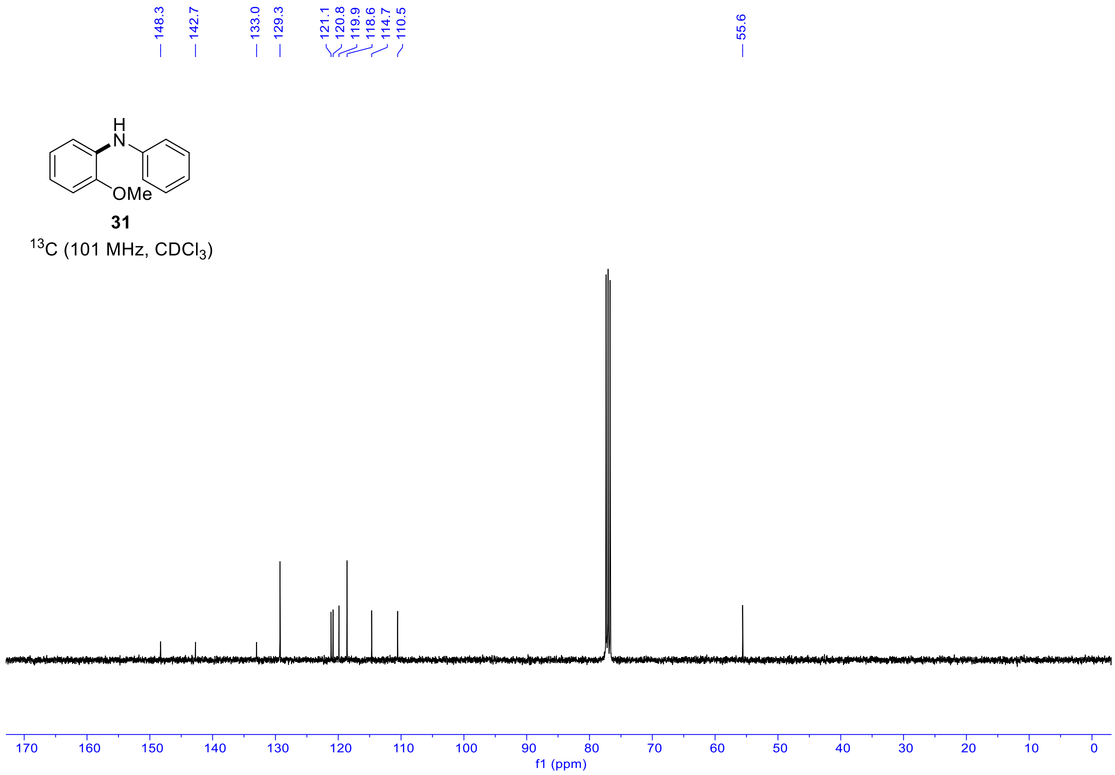

Supplementary Figure 113. ^{13}**C NMR** of compound **31**

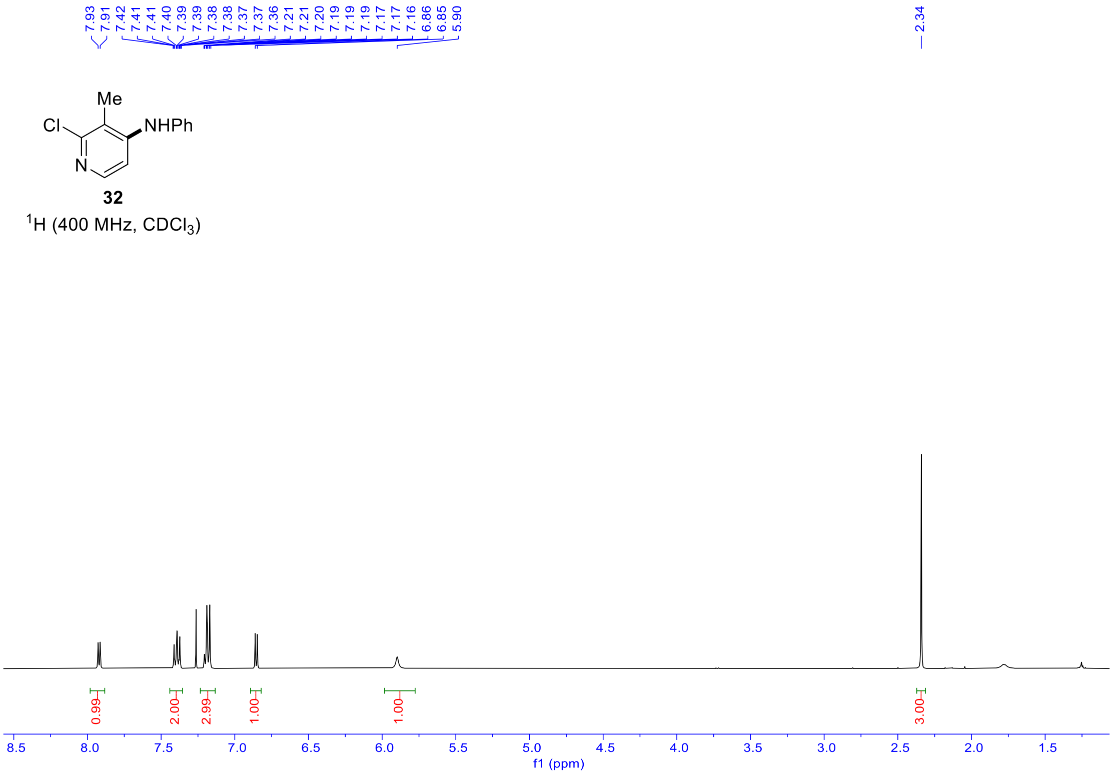

Supplementary Figure 114. ^{1}**H NMR** of compound **32**

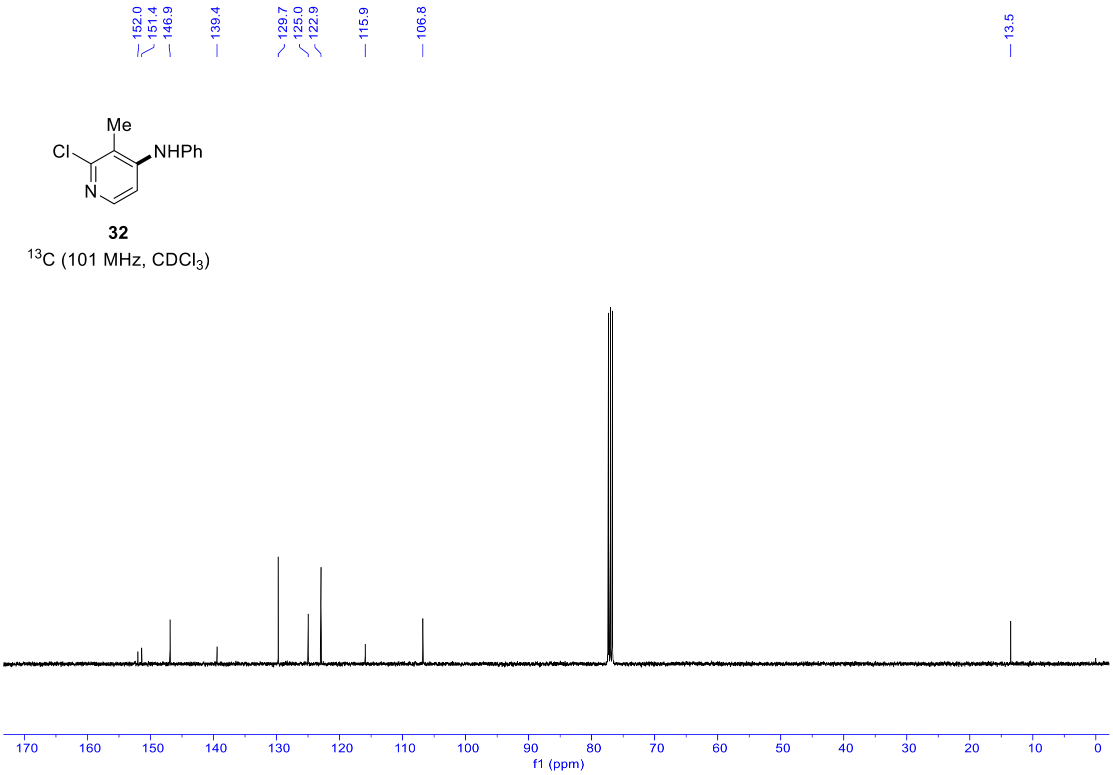

Supplementary Figure 115. ^{13}**C NMR** of compound **32**

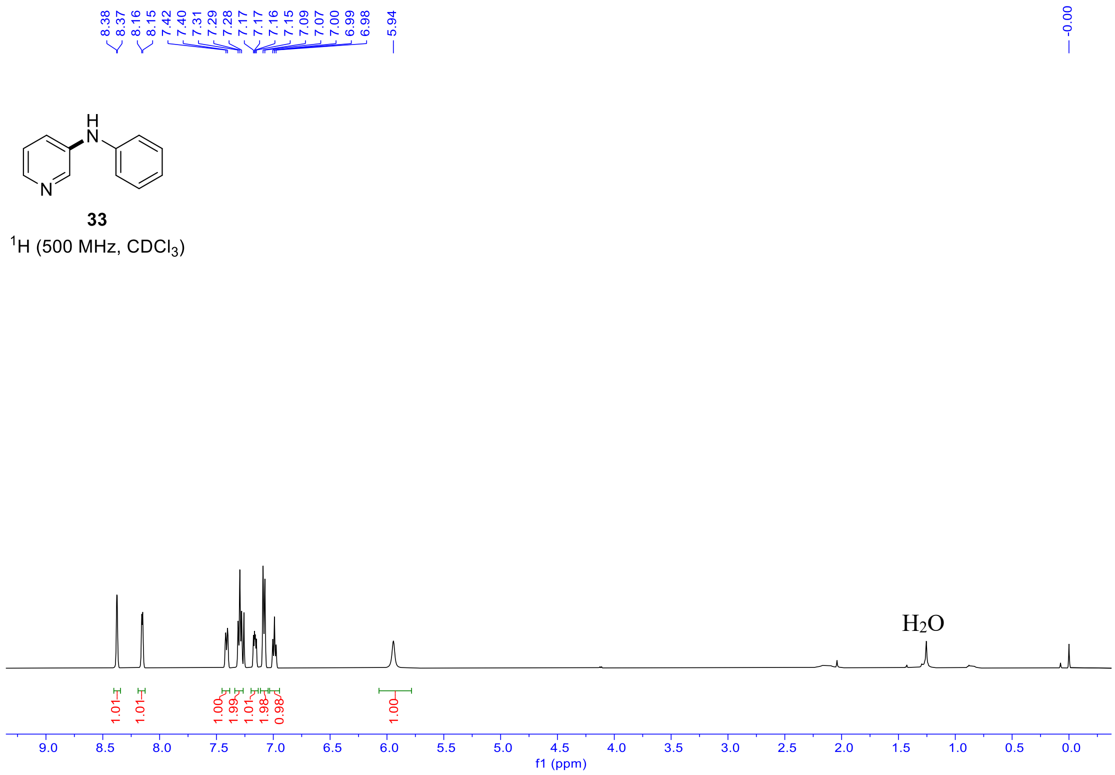

Supplementary Figure 116. ^{1}H NMR of compound **33**

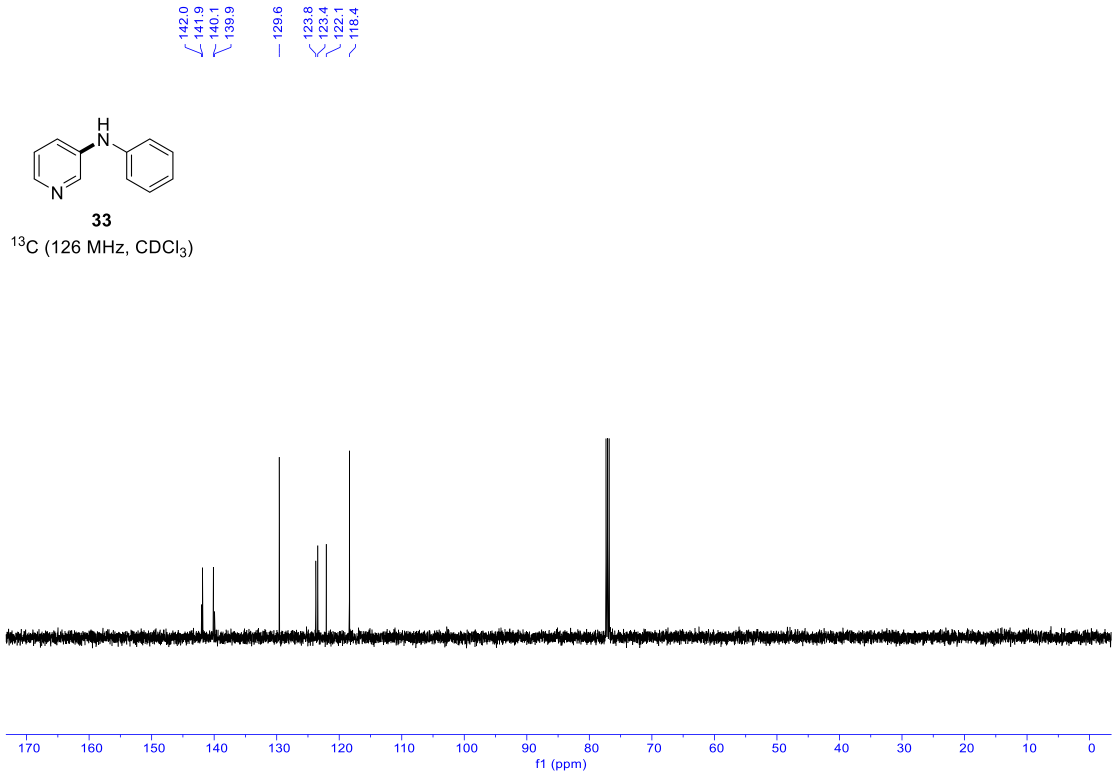

Supplementary Figure 117. ^{13}C NMR of compound **33**

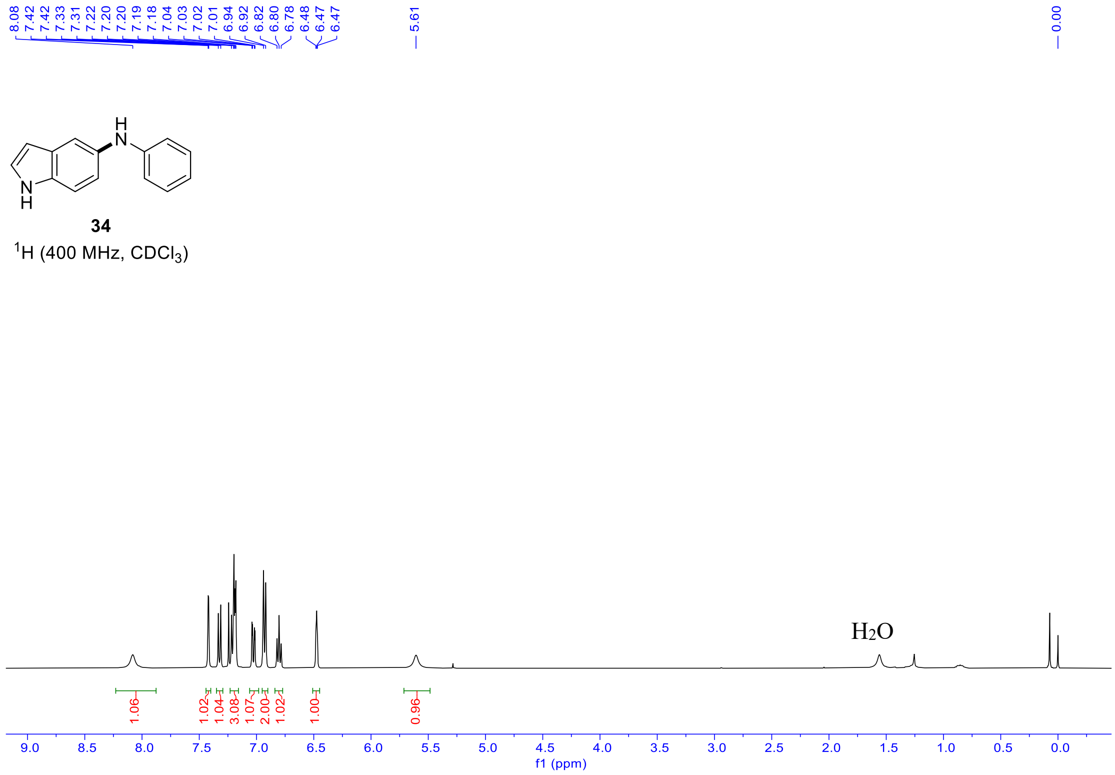

Supplementary Figure 118. ^{1}**H NMR** of compound **34**

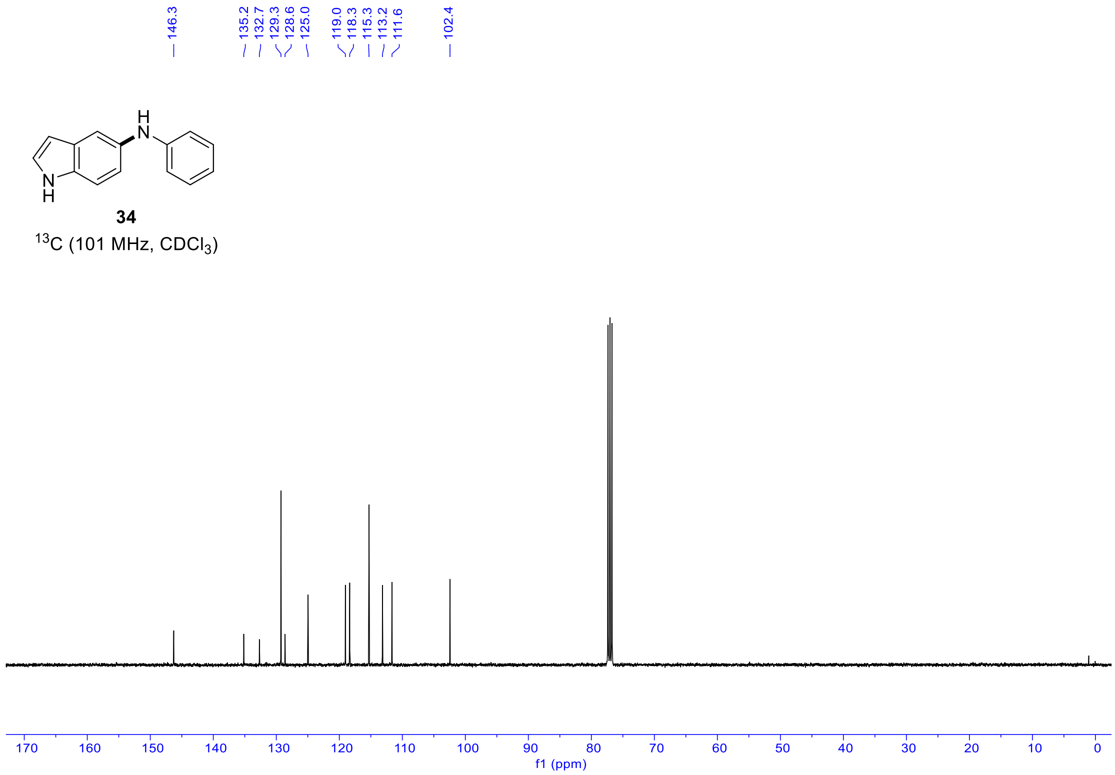

Supplementary Figure 119. ^{13}**C NMR** of compound **34**

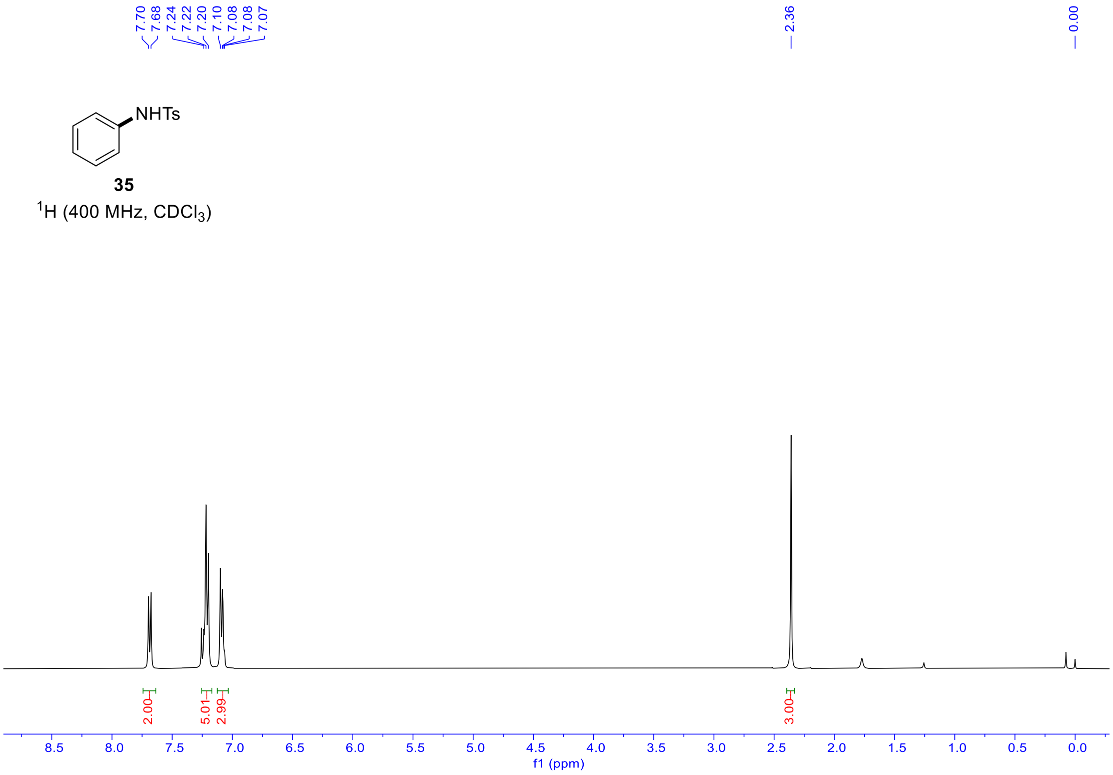

Supplementary Figure 120. ^{1}**H NMR** of compound **35**

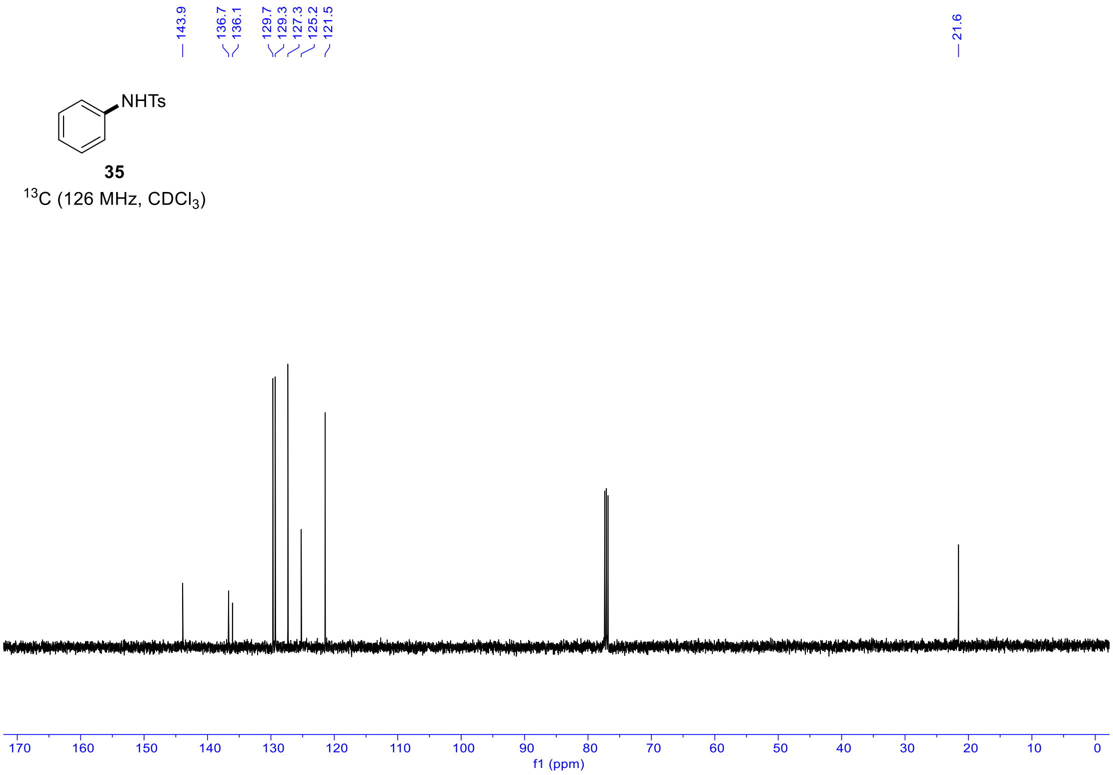

Supplementary Figure 121. ^{13}**C NMR** of compound **35**

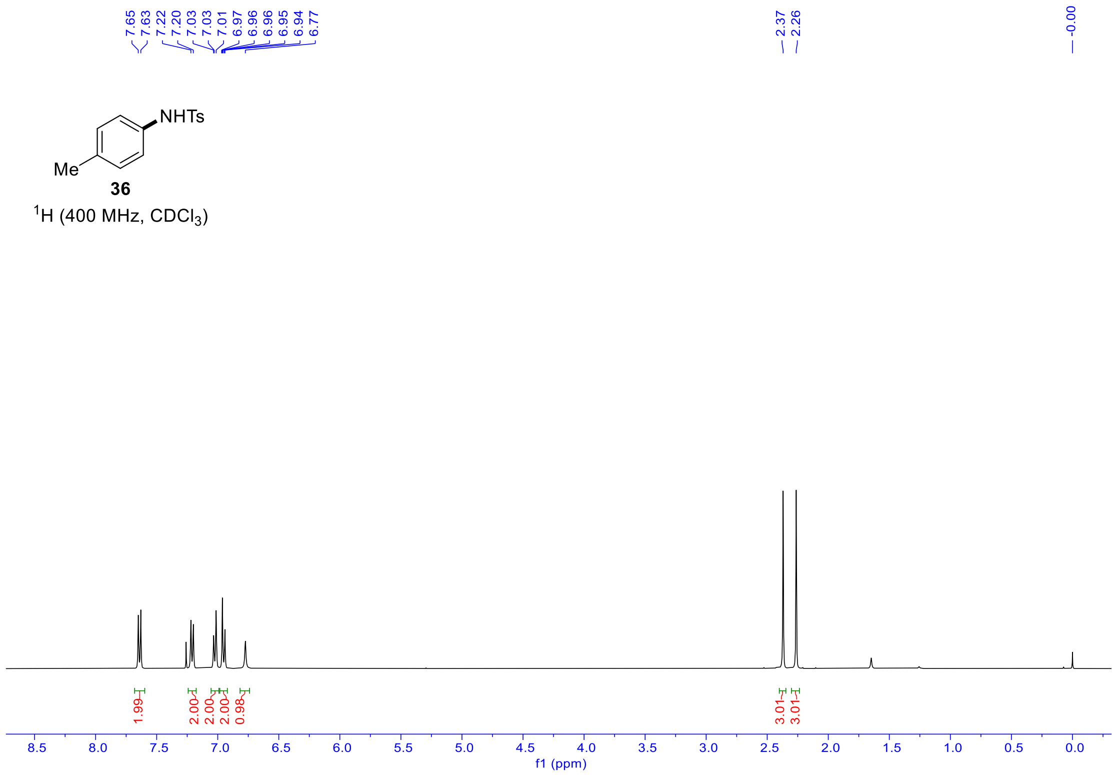

Supplementary Figure 122. ^{1}**H NMR** of compound **36**

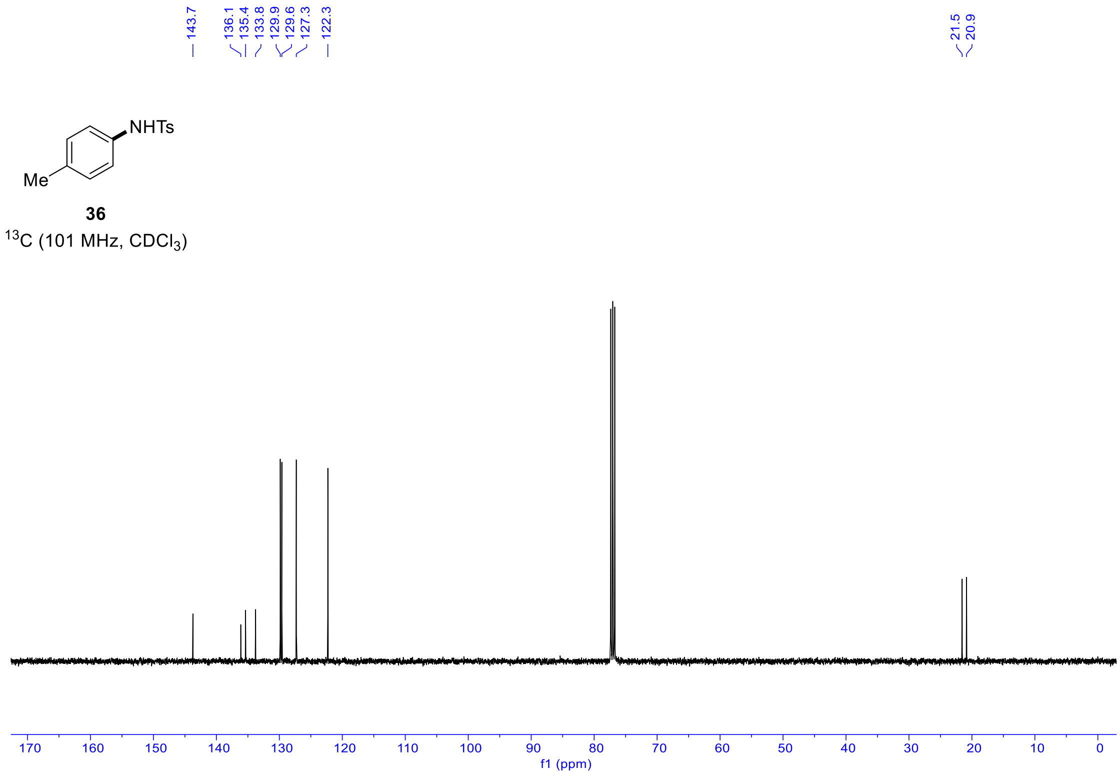

Supplementary Figure 123. ^{13}**C NMR** of compound **36**

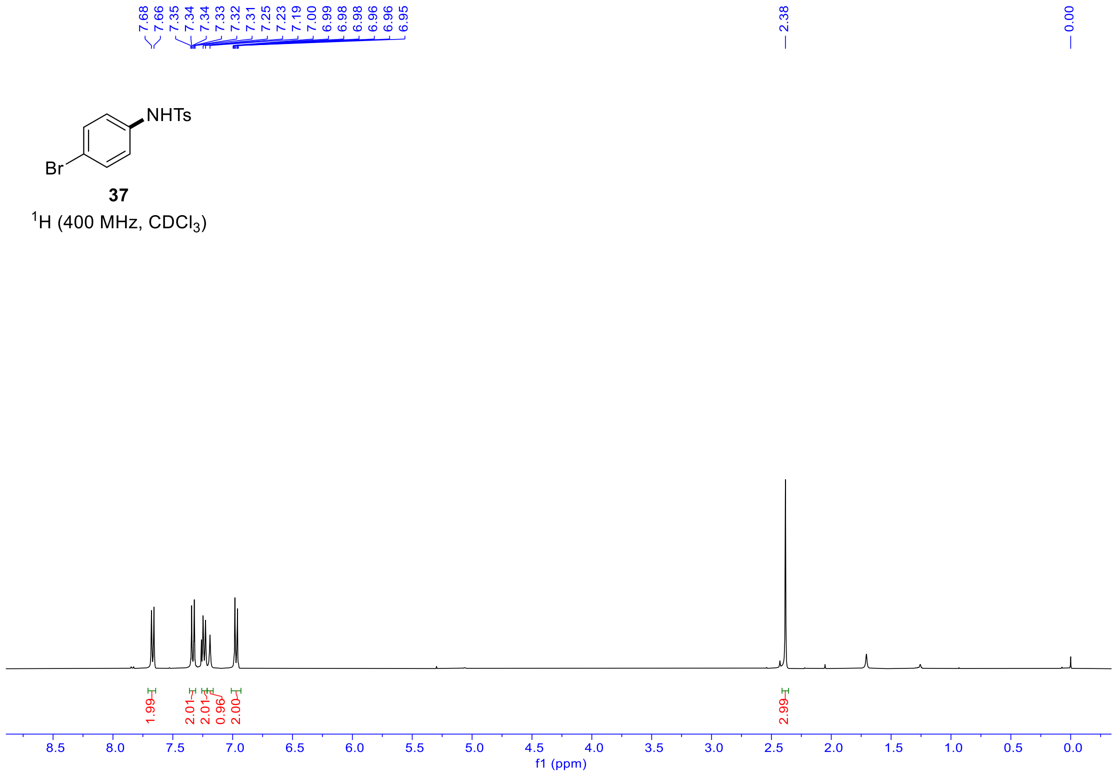

Supplementary Figure 124. ^{1}H NMR of compound **37**

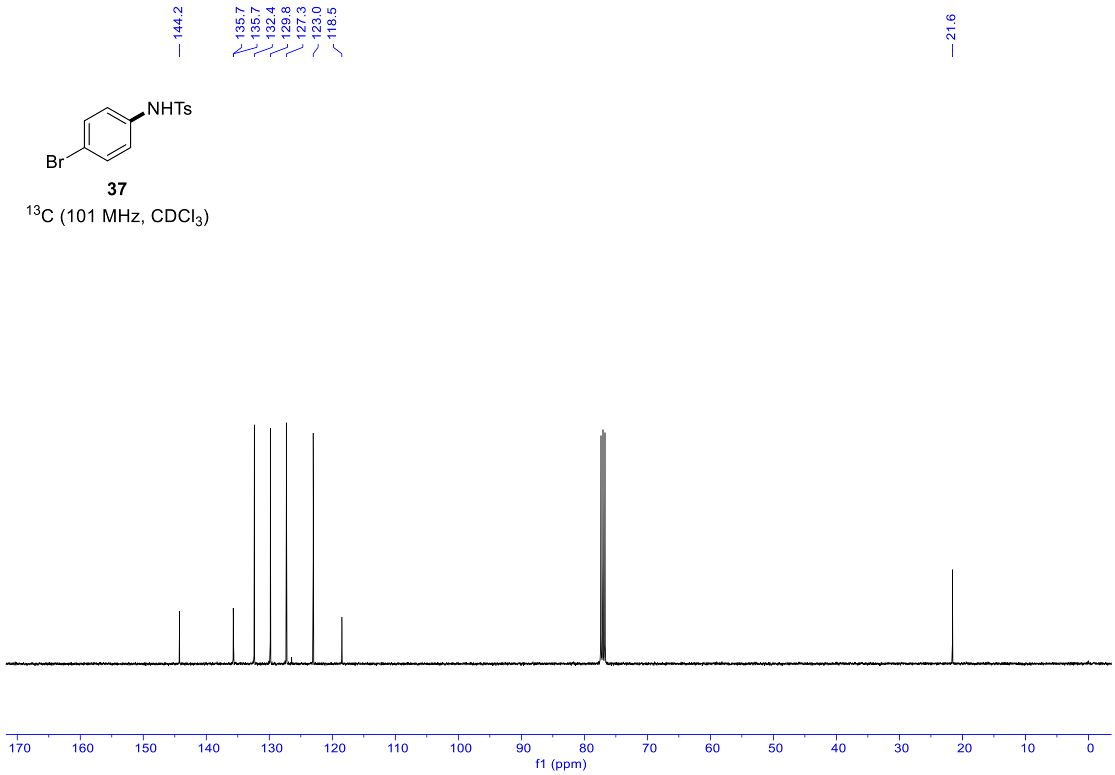

Supplementary Figure 125. ^{13}C NMR of compound **37**

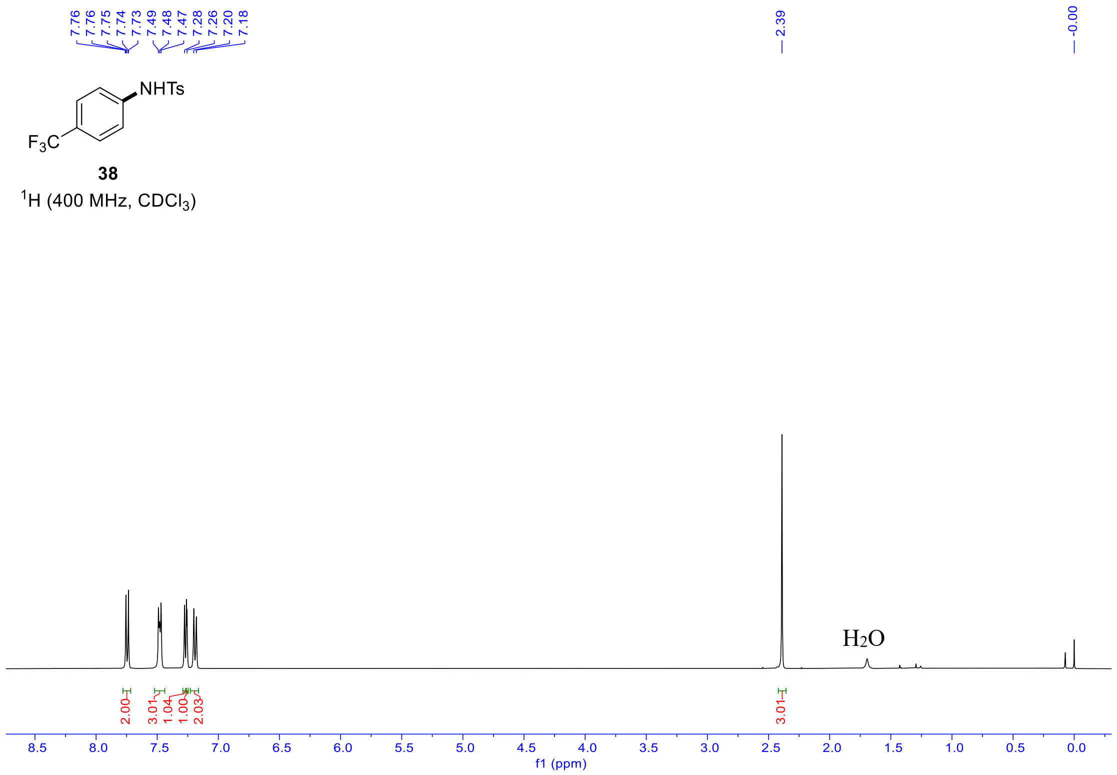

Supplementary Figure 126. ^{1}**H NMR** of compound **38**

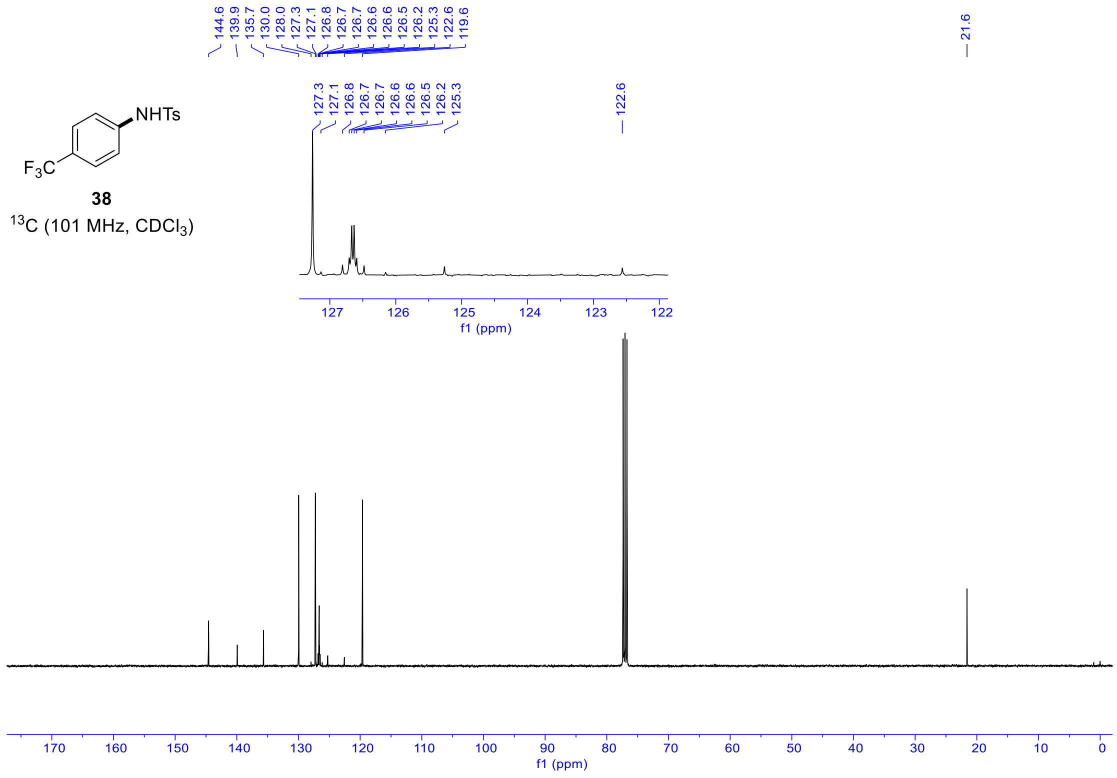

Supplementary Figure 127. ^{13}**C NMR** of compound **38**

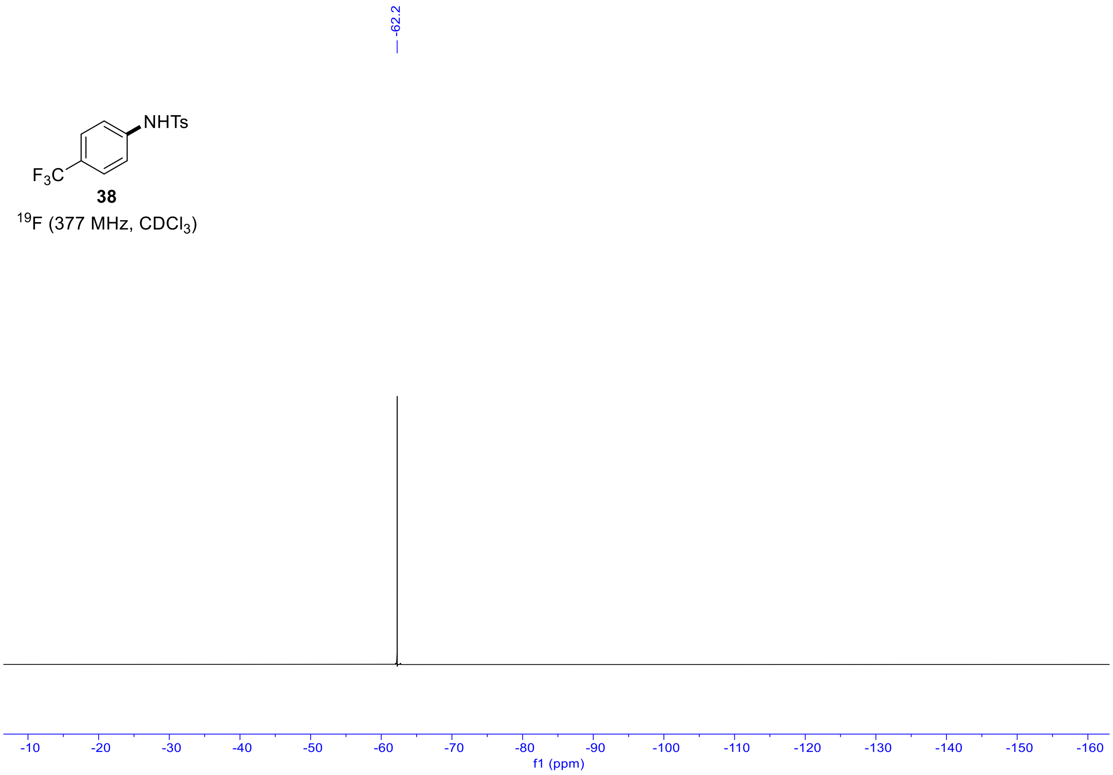

Supplementary Figure 128. **^{19}F NMR** of compound **38**

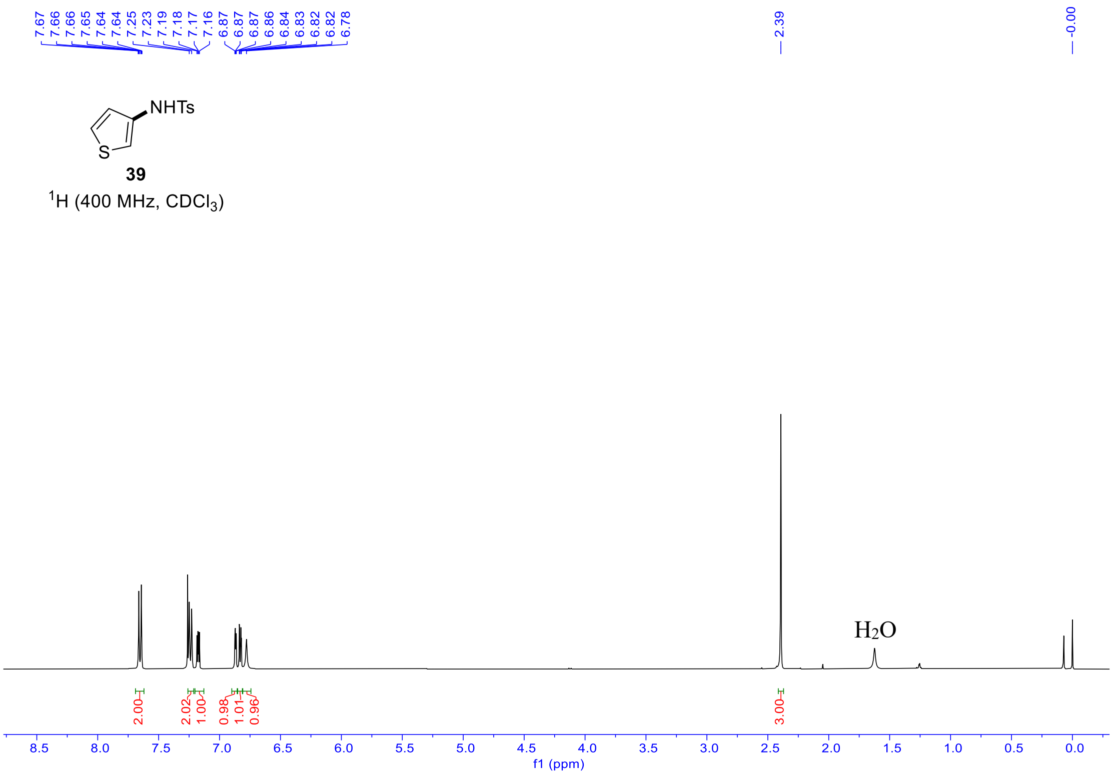

Supplementary Figure 129. **^{1}H NMR** of compound **39**

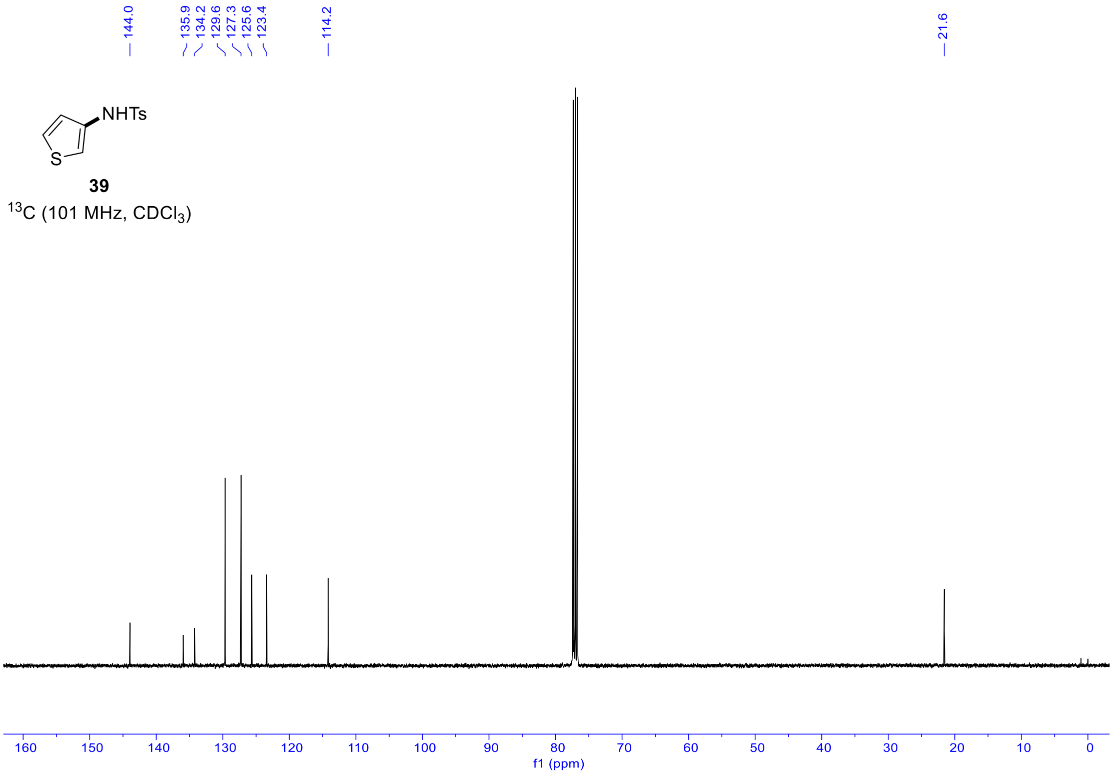

Supplementary Figure 130. ^{13}**C NMR** of compound **39**

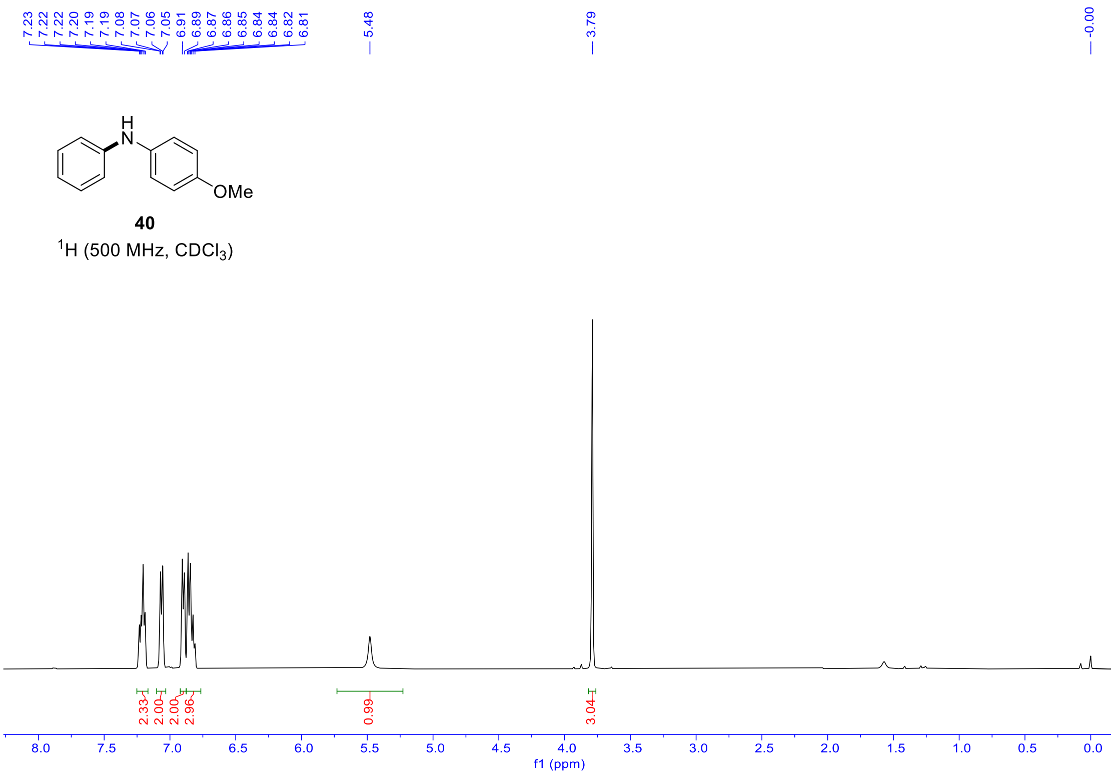

Supplementary Figure 131. ^{1}**H NMR** of compound **40**

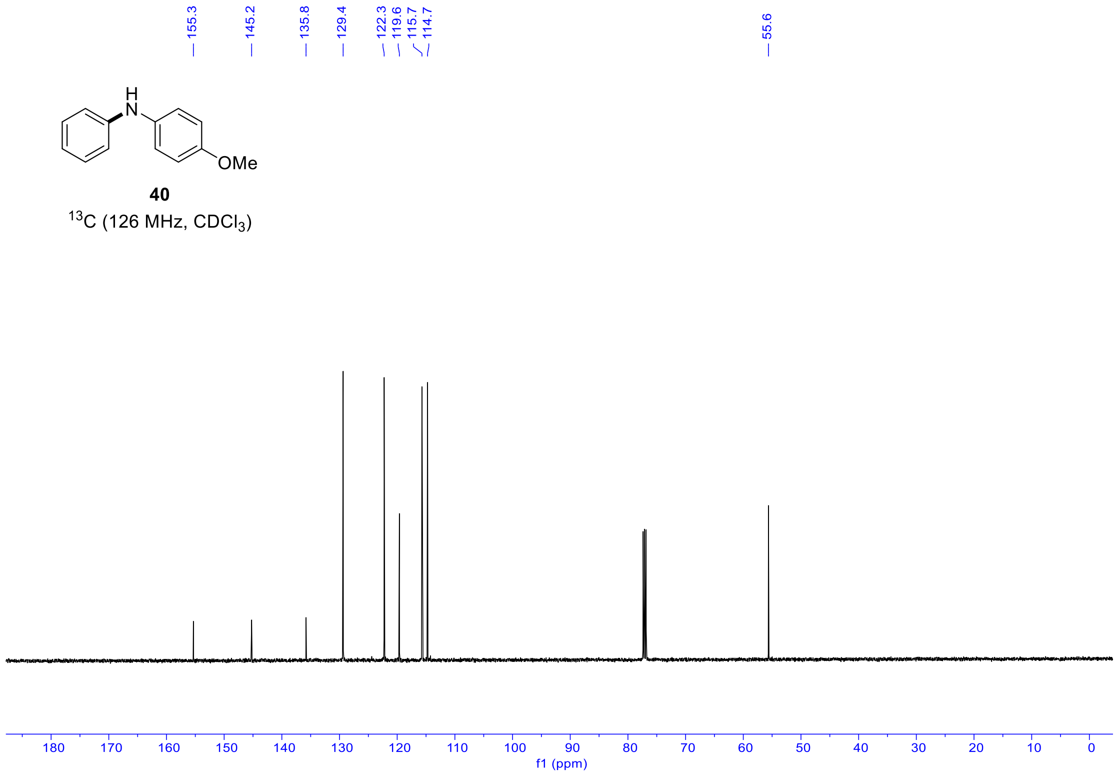

Supplementary Figure 132. ^{13}**C NMR** of compound **40**

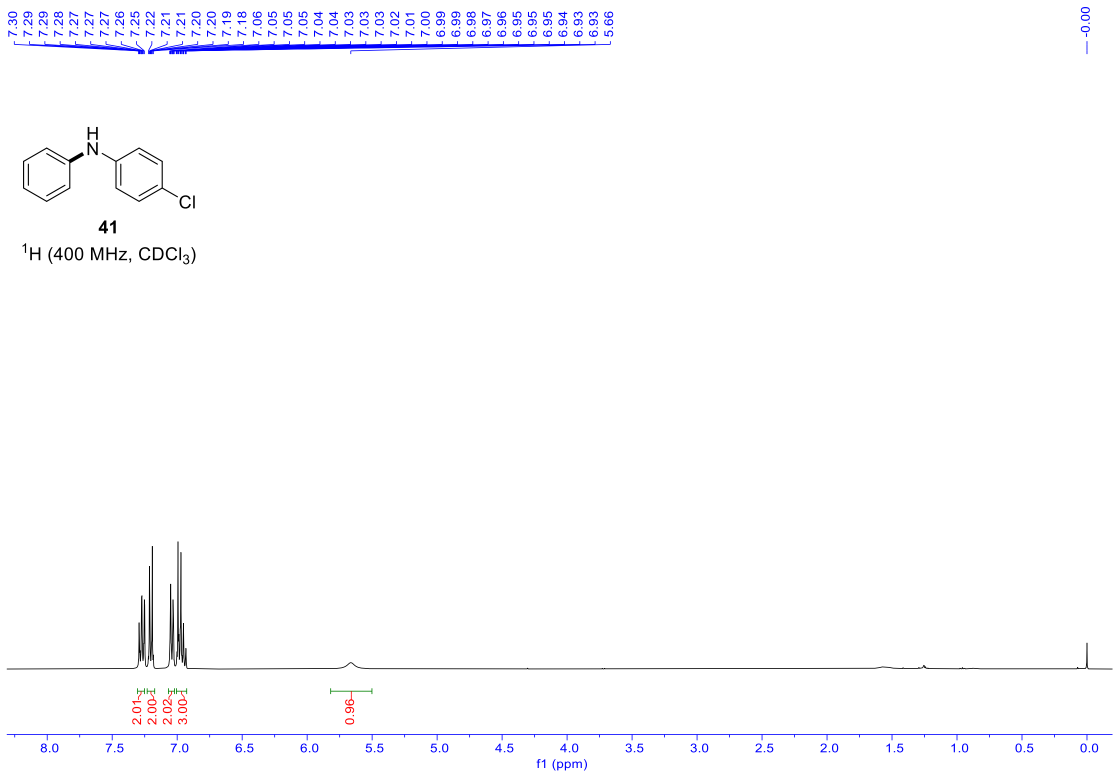

Supplementary Figure 133. ^{1}**H NMR** of compound **41**

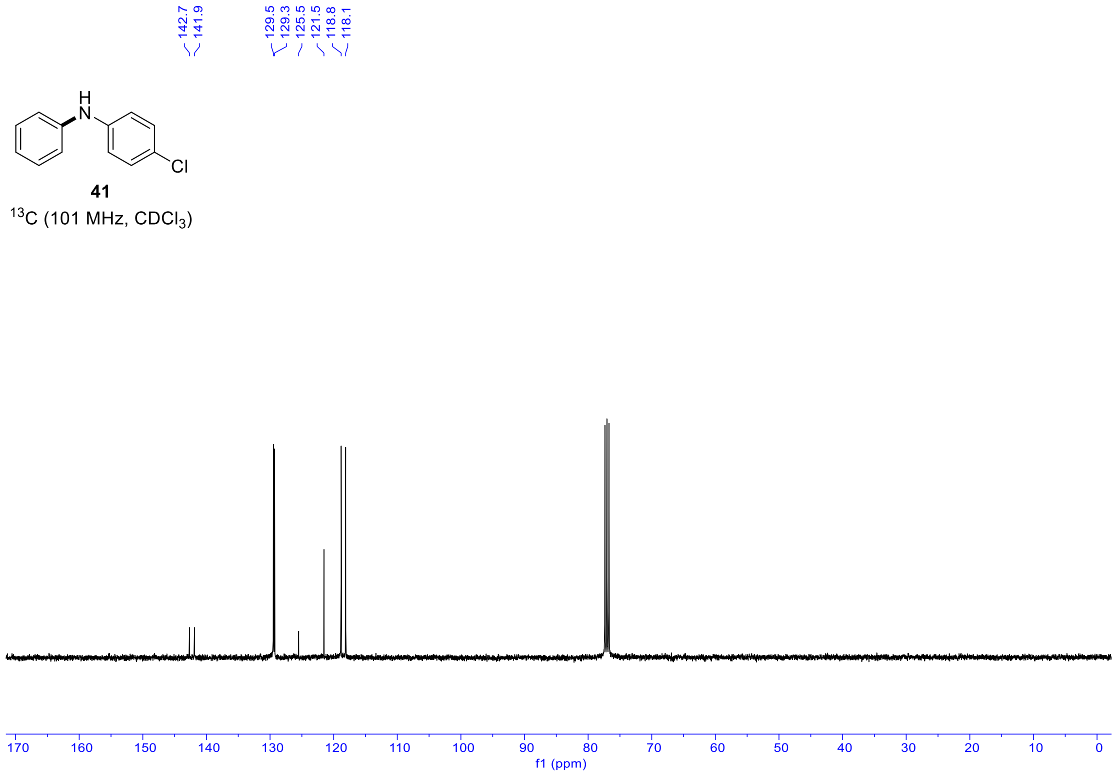

Supplementary Figure 134. ^{13}**C NMR** of compound **41**

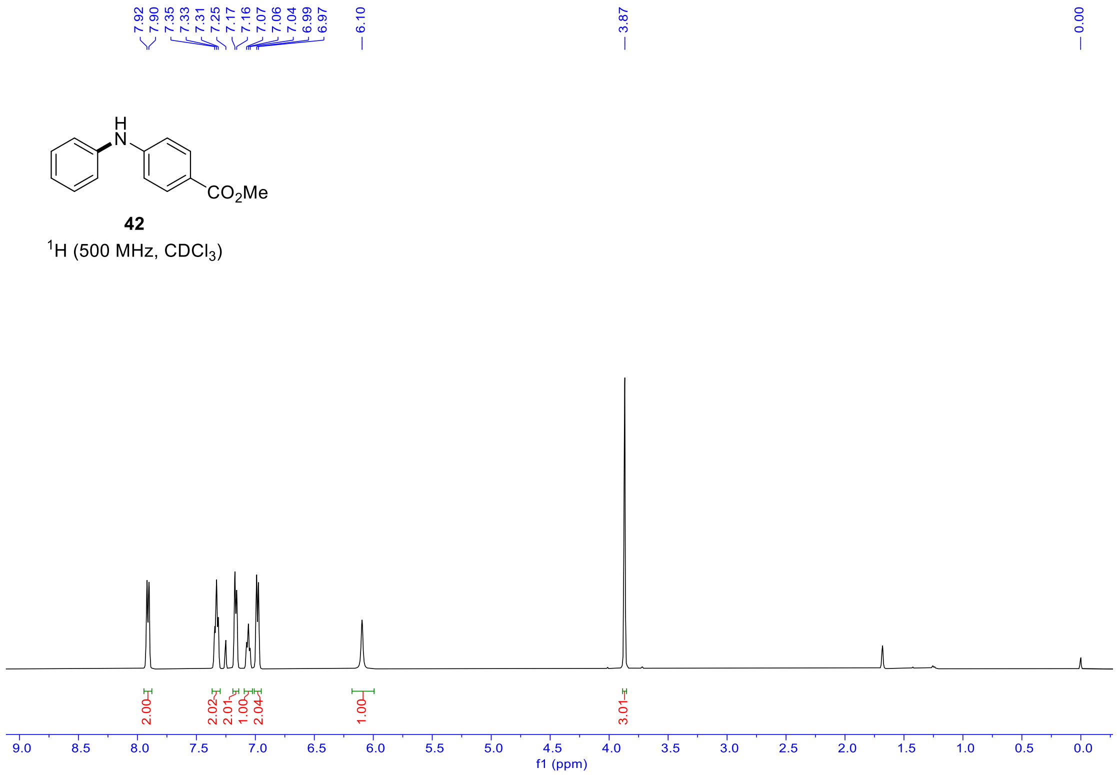

Supplementary Figure 135. ^{1}**H NMR** of compound **42**

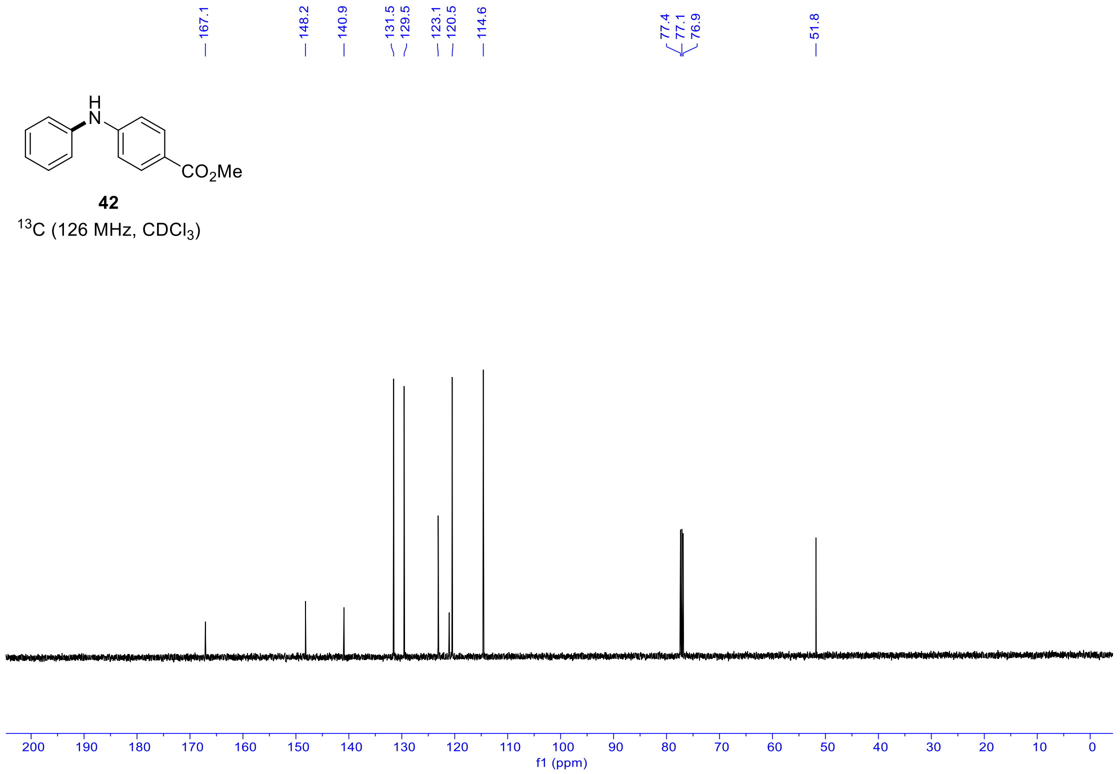

Supplementary Figure 136. ^{13}**C NMR** of compound **42**

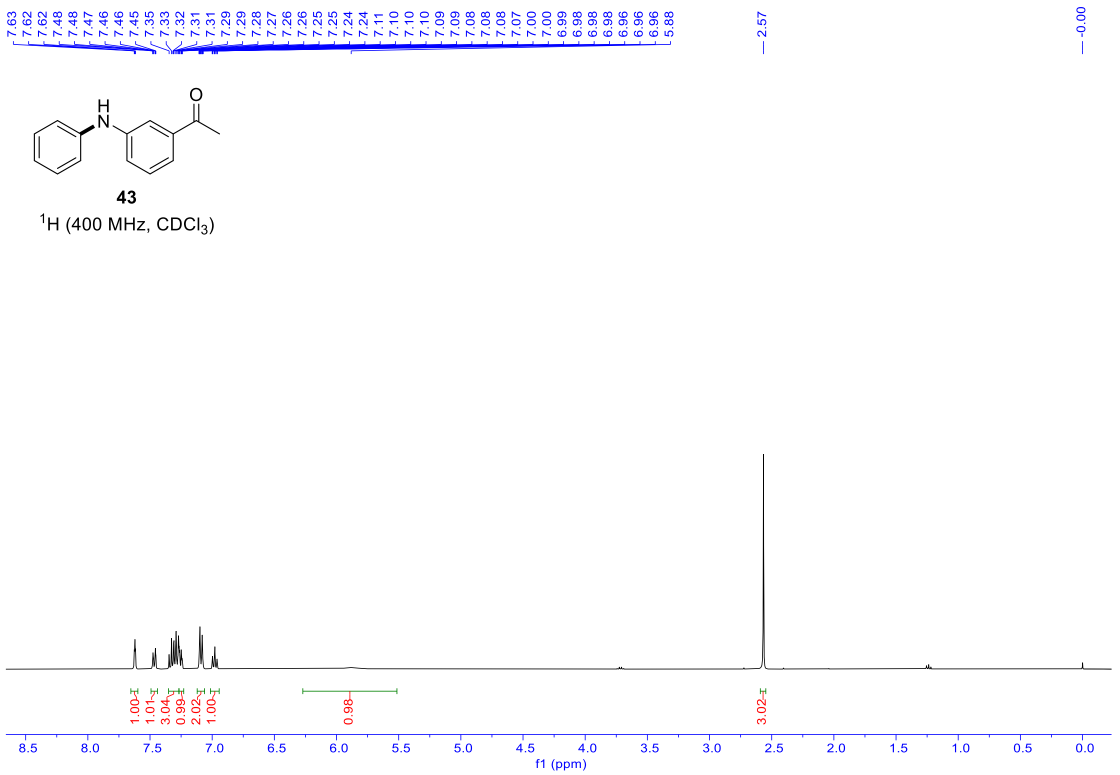

Supplementary Figure 137. ^{1}**H NMR** of compound **43**

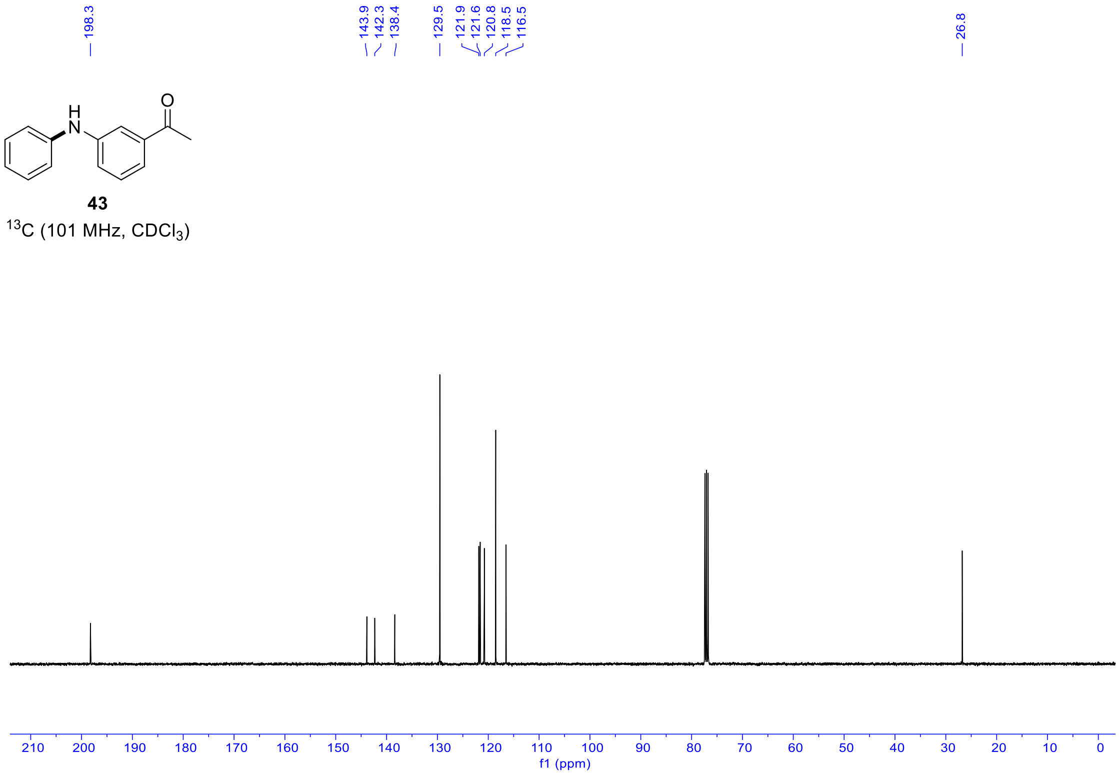

Supplementary Figure 138. ^{13}C NMR of compound **43**

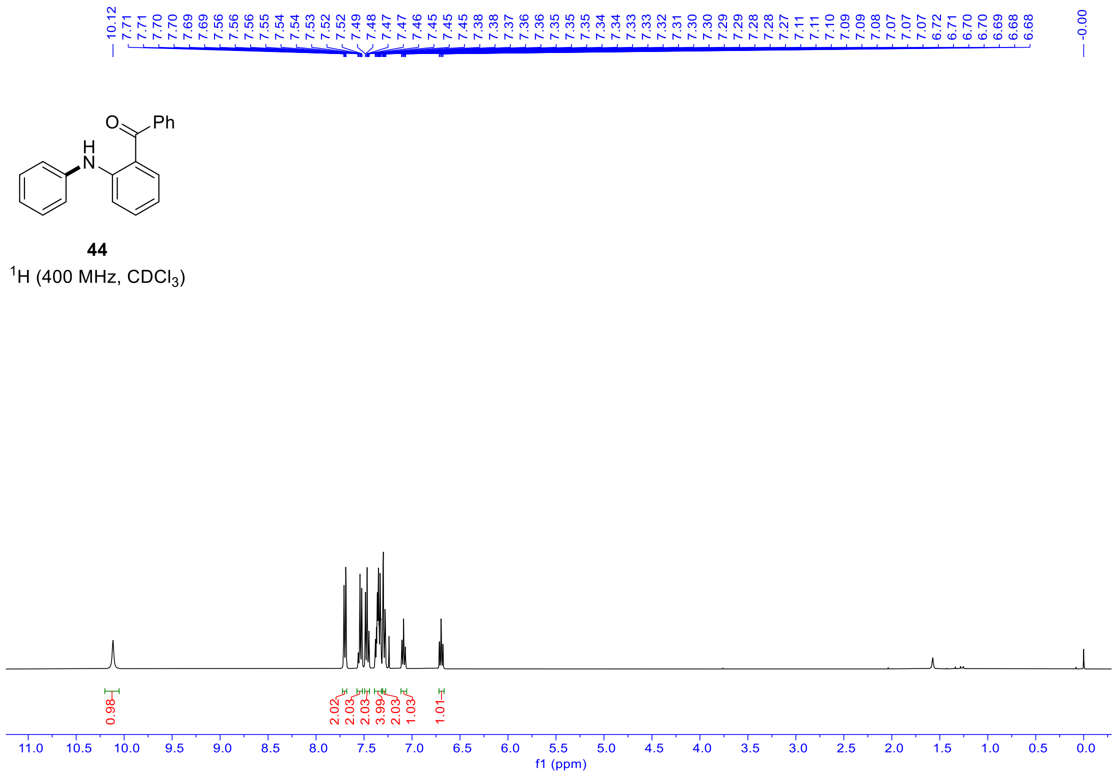

Supplementary Figure 139. ^{1}H NMR of compound **44**

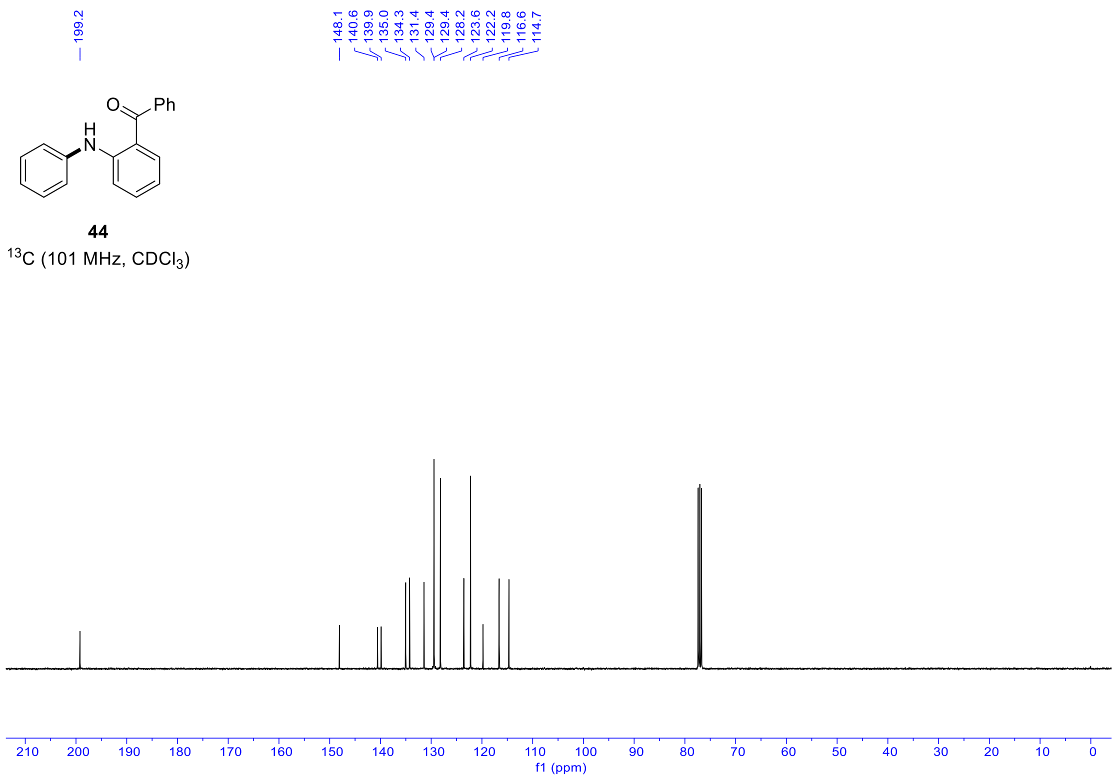

Supplementary Figure 140. ^{13}C NMR of compound **44**

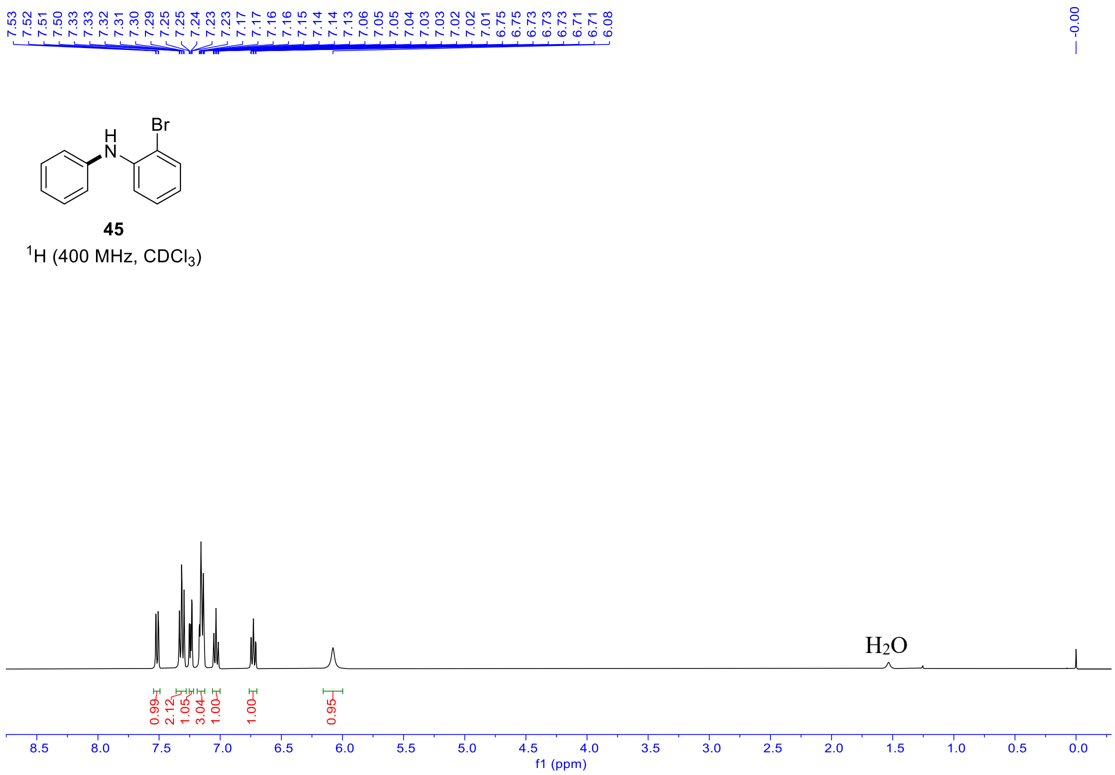

Supplementary Figure 141. ^{1}H NMR of compound **45**

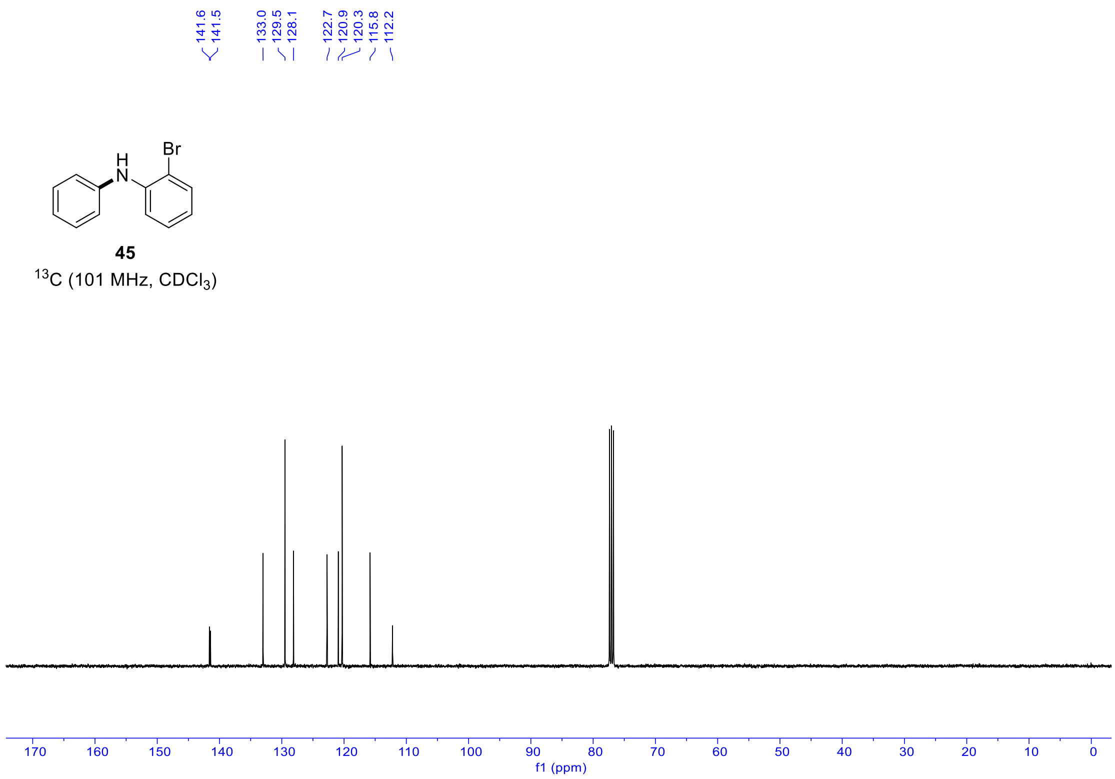

Supplementary Figure 142. ^{13}C NMR of compound **45**

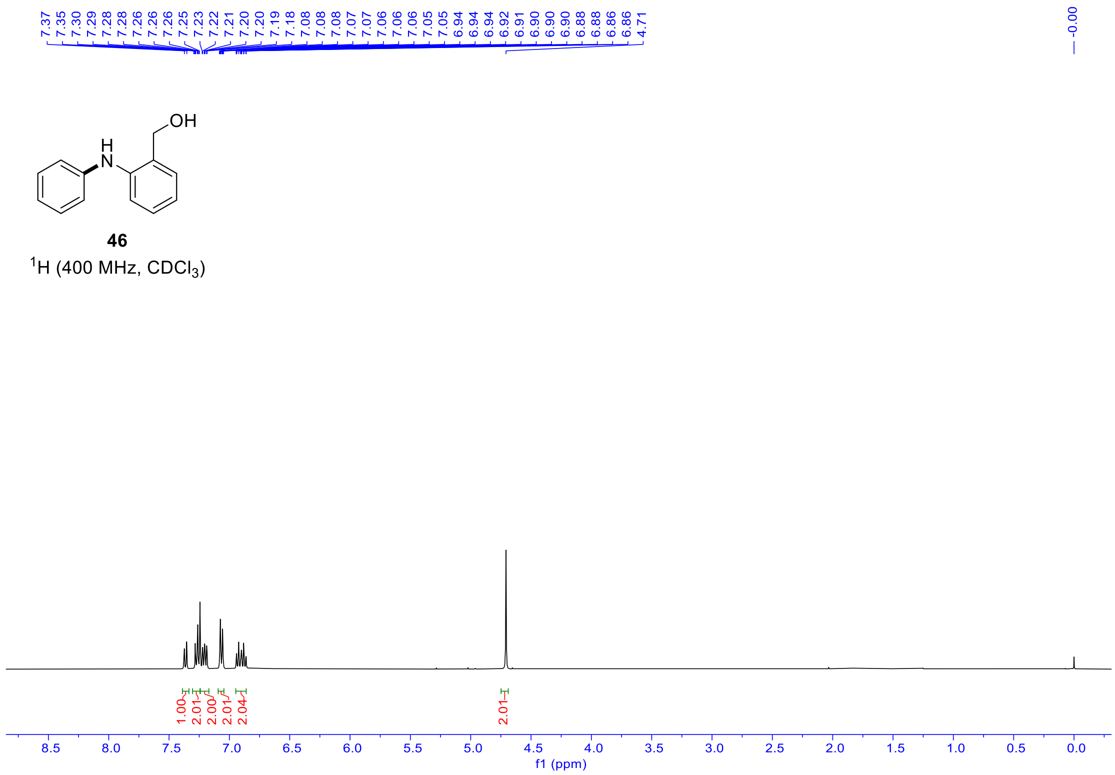

Supplementary Figure 143. ^{1}H NMR of compound **46**

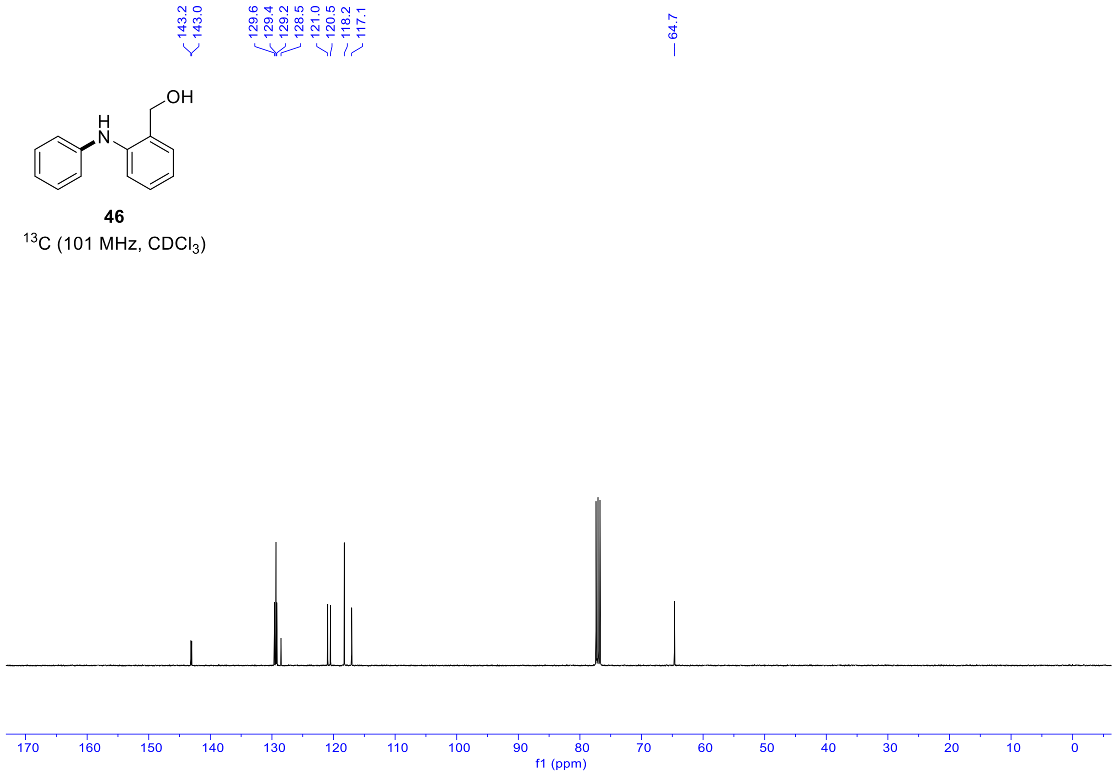

Supplementary Figure 144. ^{13}**C NMR** of compound **46**

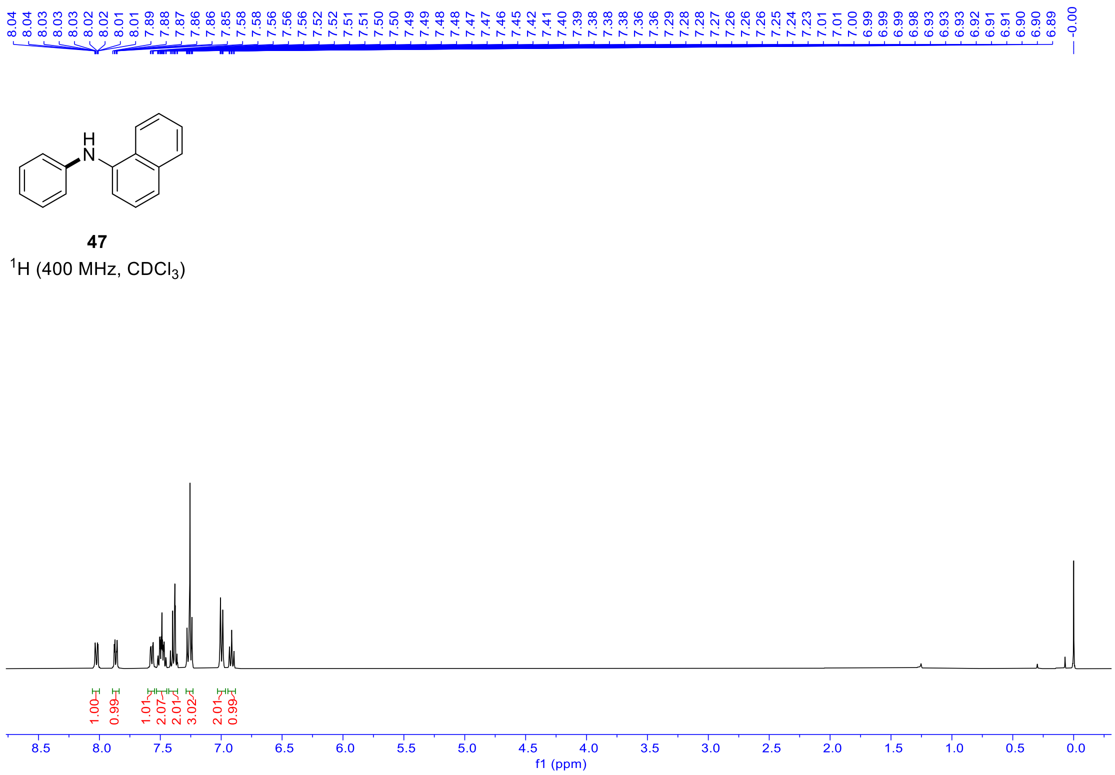

Supplementary Figure 145. ^{1}**H NMR** of compound **47**

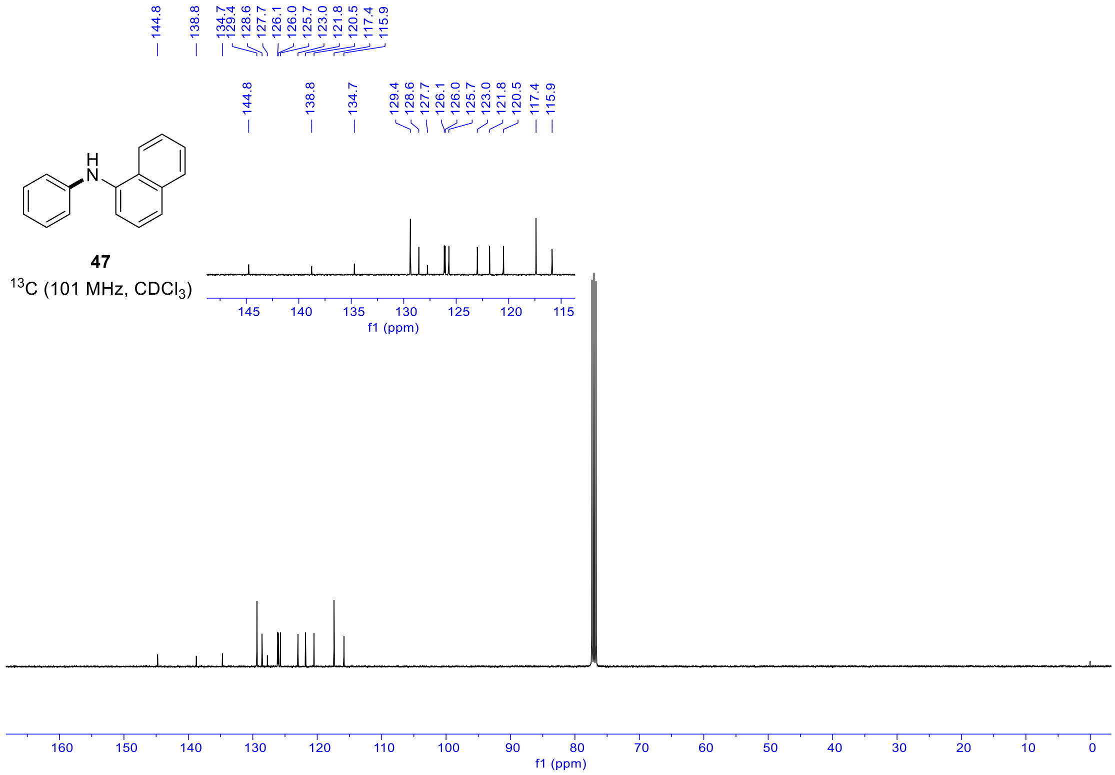

Supplementary Figure 146. **^{13}C NMR** of compound **47**

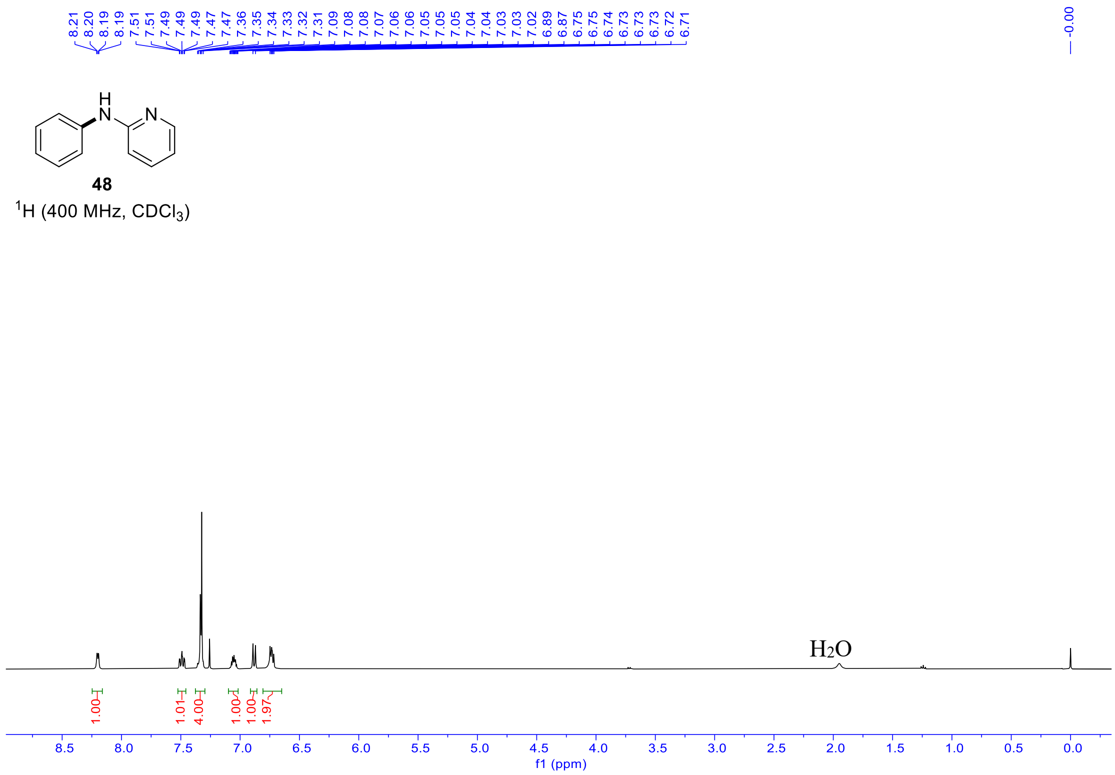

Supplementary Figure 147. **^{1}H NMR** of compound **48**

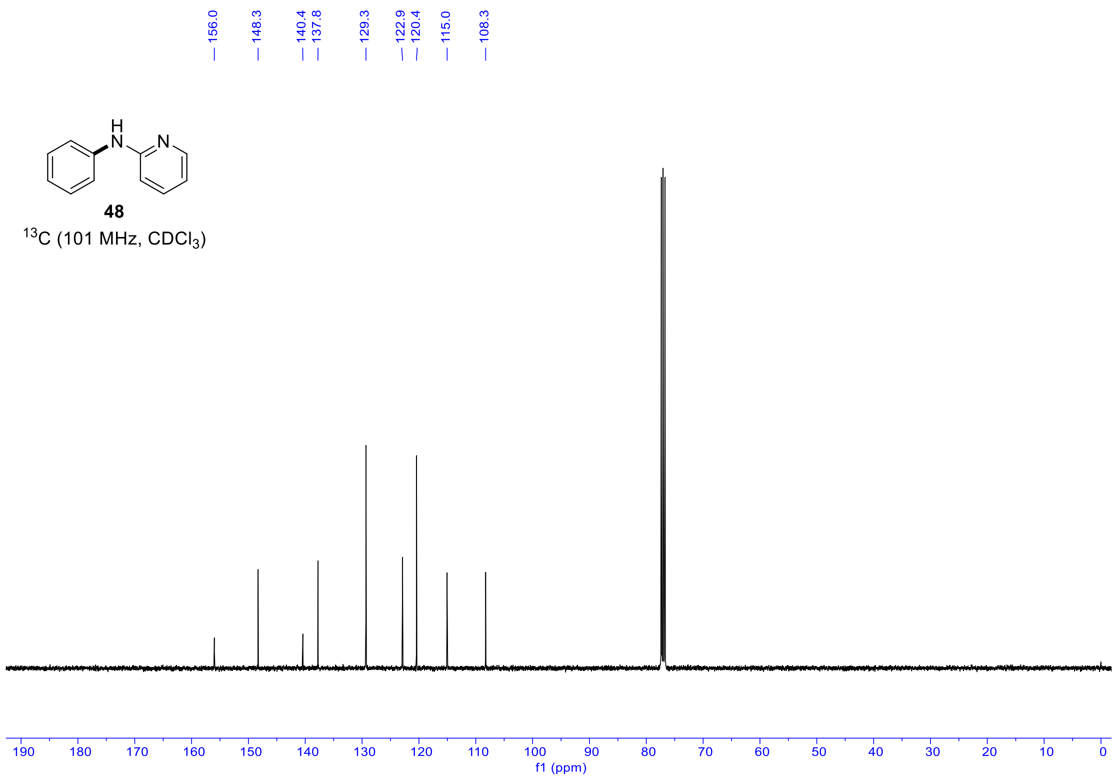

Supplementary Figure 148. ^{13}**C NMR** of compound **48**

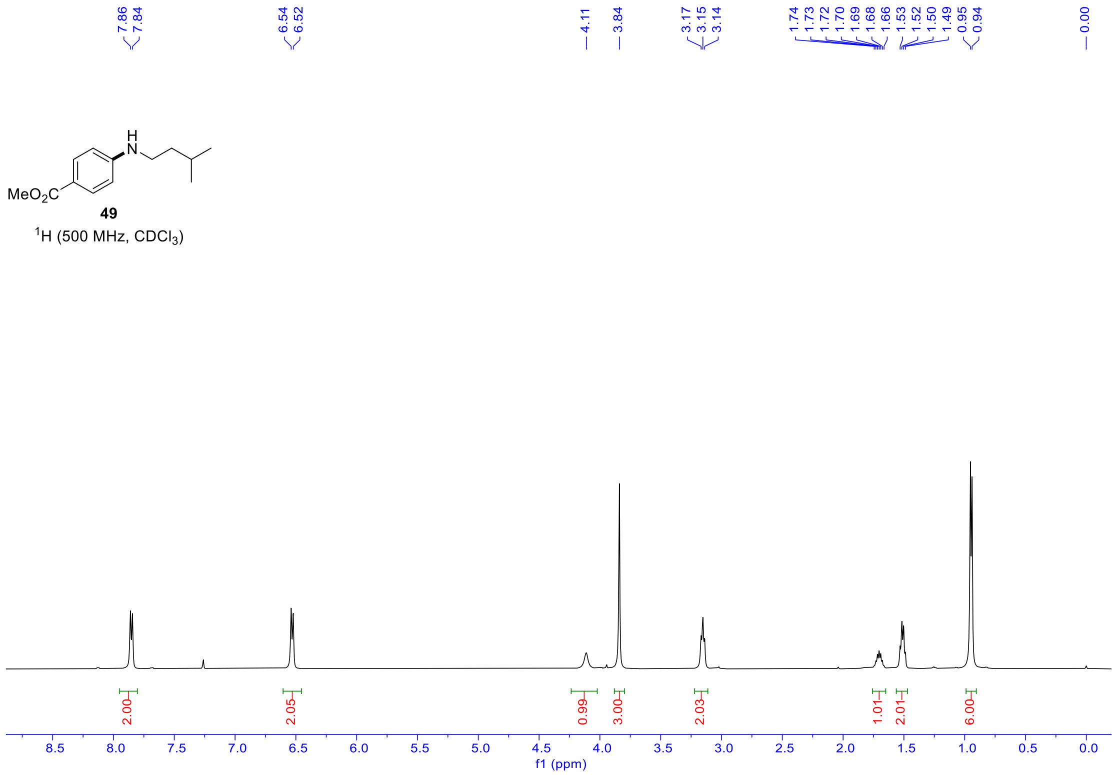

Supplementary Figure 149. ^{1}**H NMR** of compound **49**

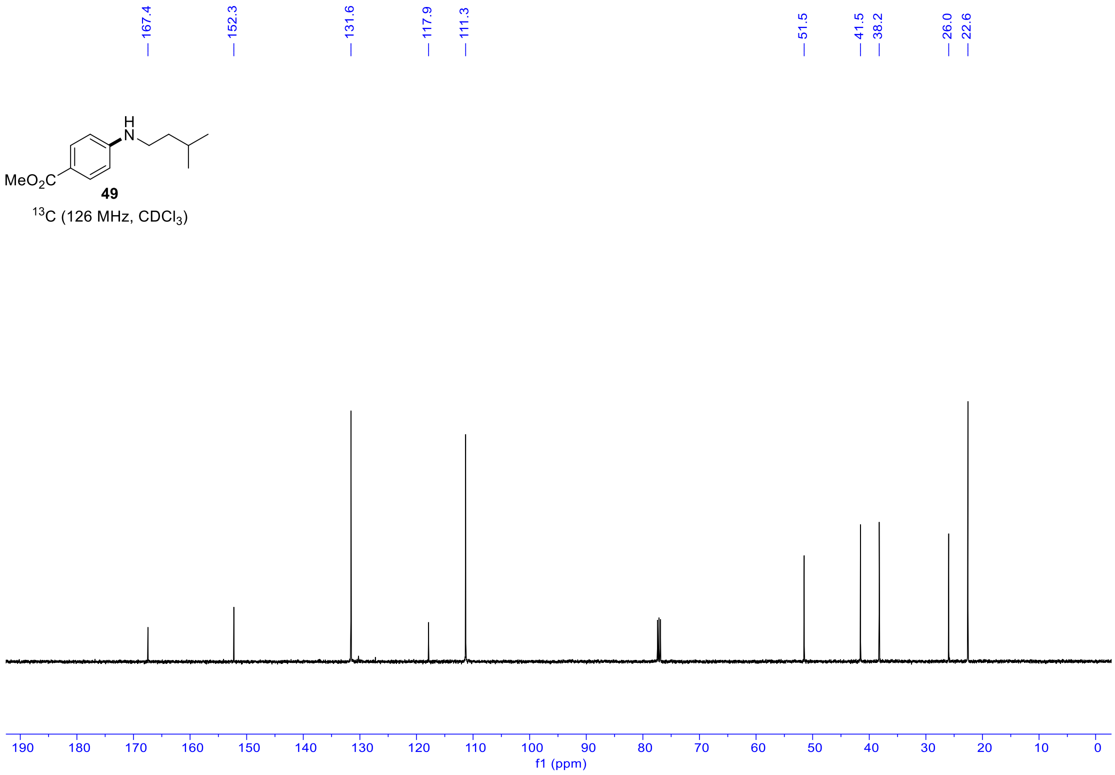

Supplementary Figure 150. ^{13}**C NMR** of compound **49**

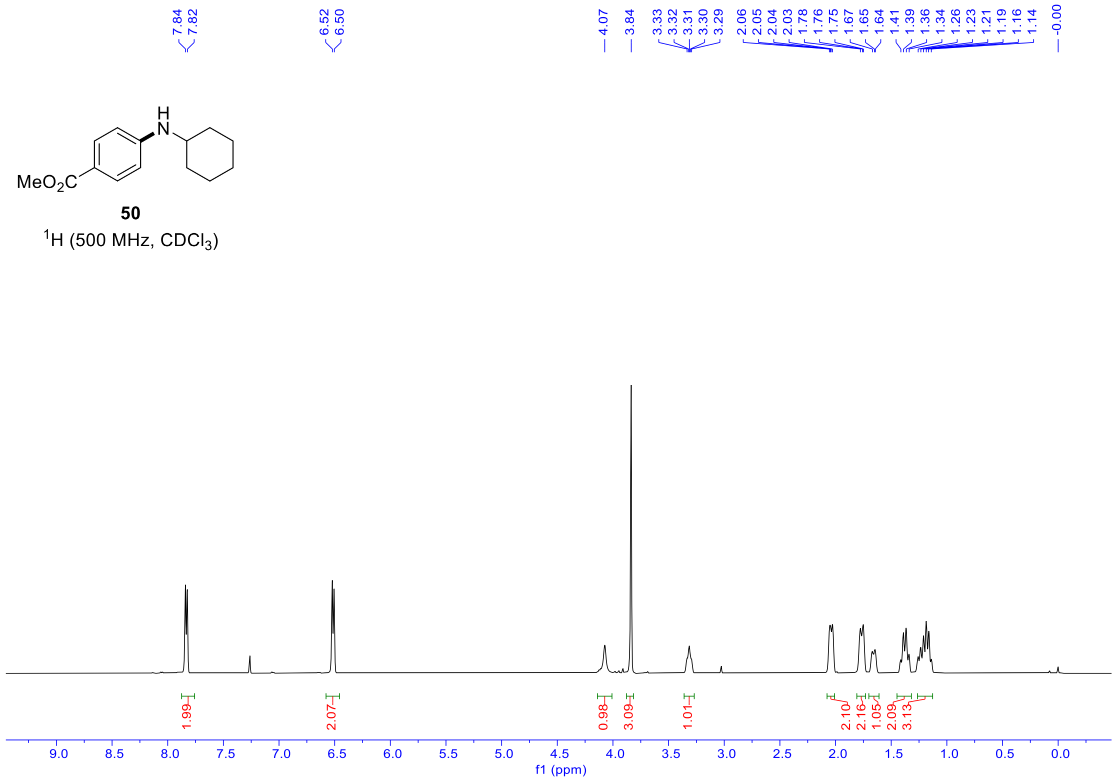

Supplementary Figure 151. ^{1}**H NMR** of compound **50**

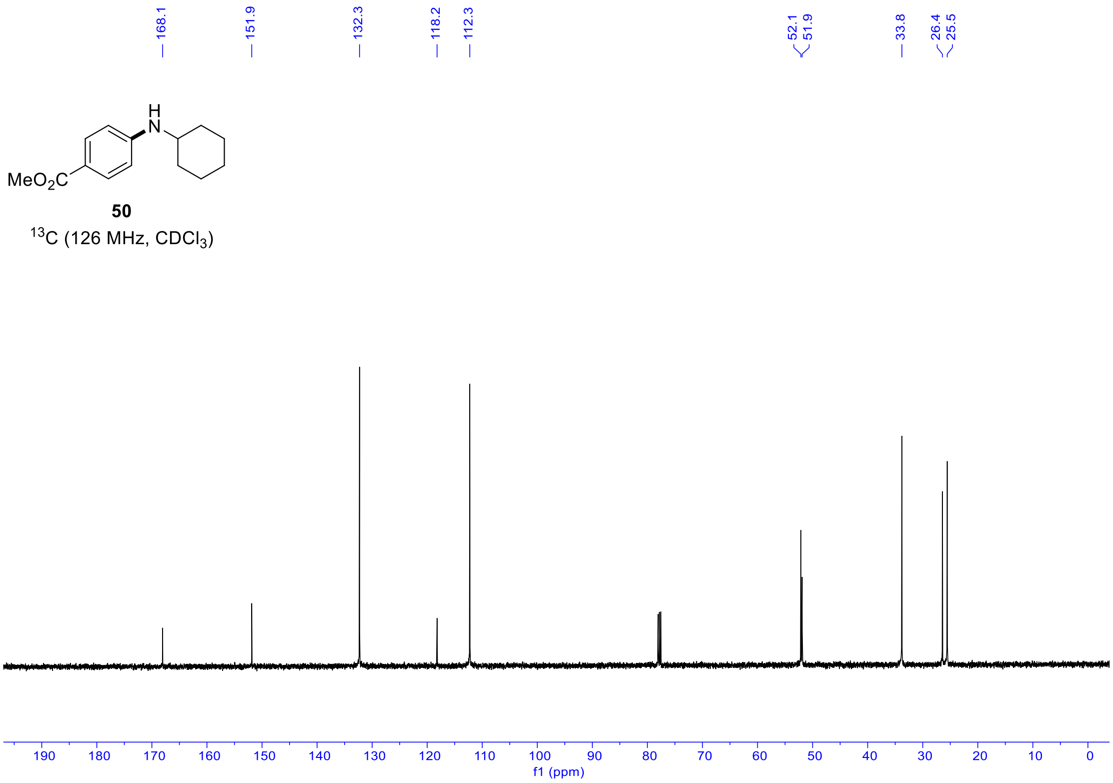

Supplementary Figure 152. ^{13}**C NMR** of compound **50**

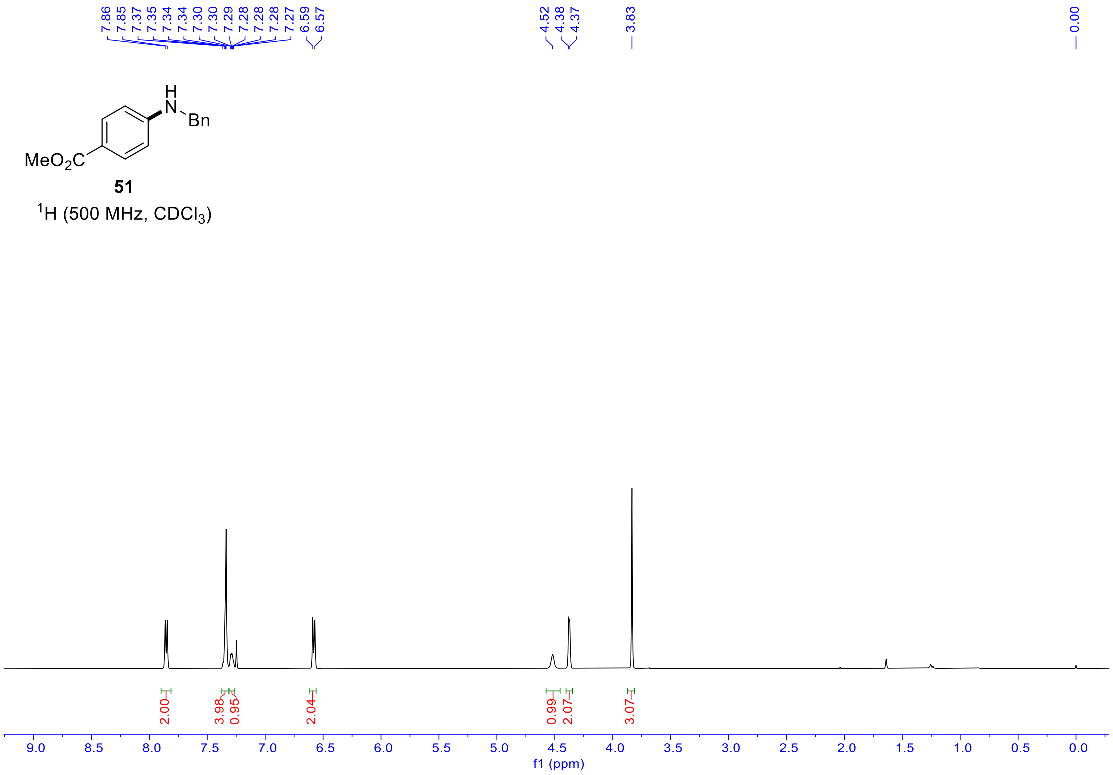

Supplementary Figure 153. ^{1}**H NMR** of compound **51**

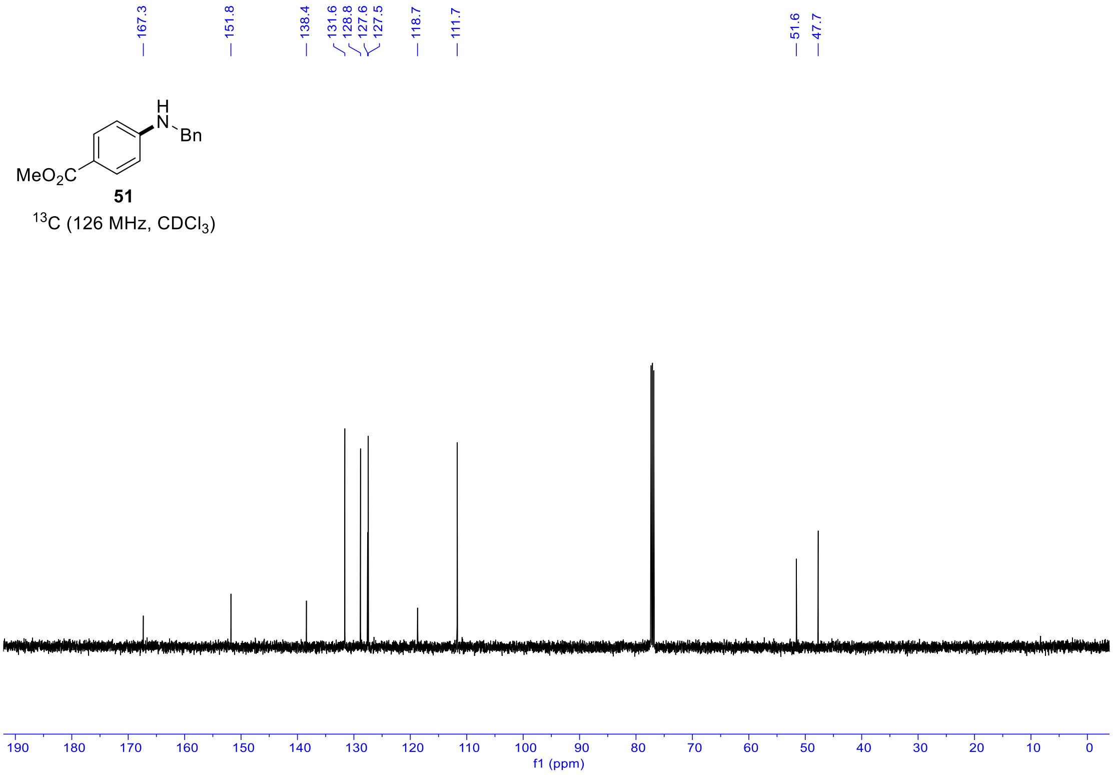

Supplementary Figure 154. ^{13}**C NMR** of compound **51**

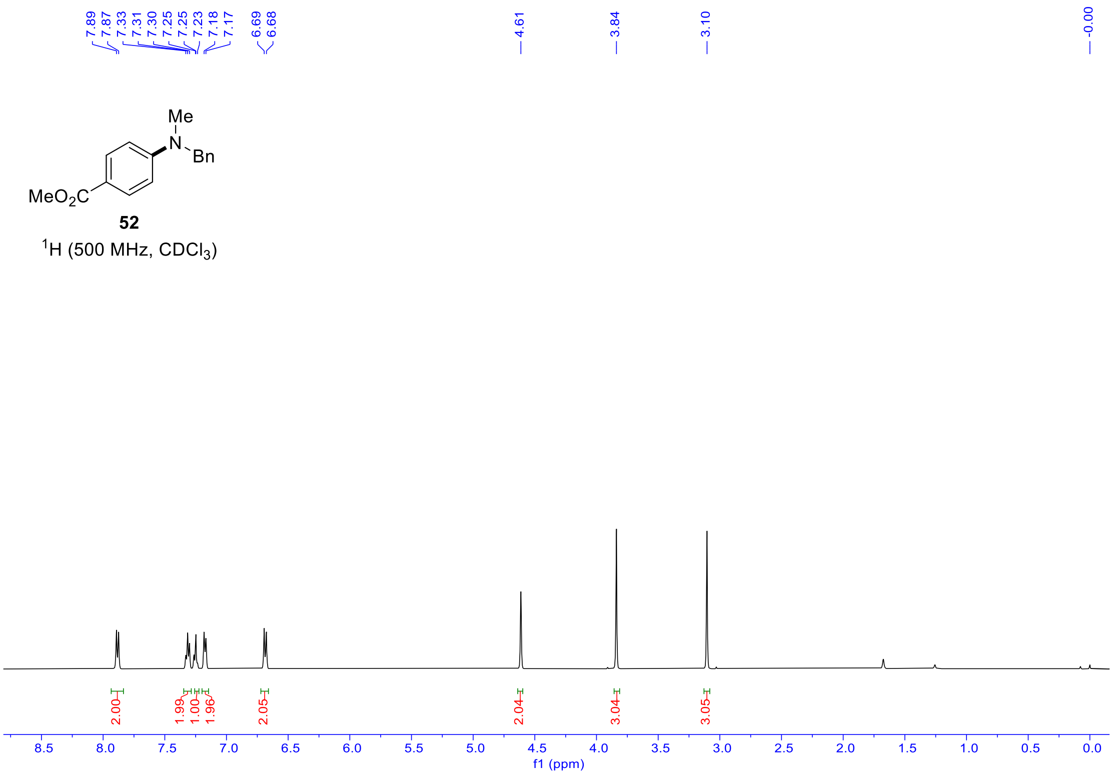

Supplementary Figure 155. ^{1}**H NMR** of compound **52**

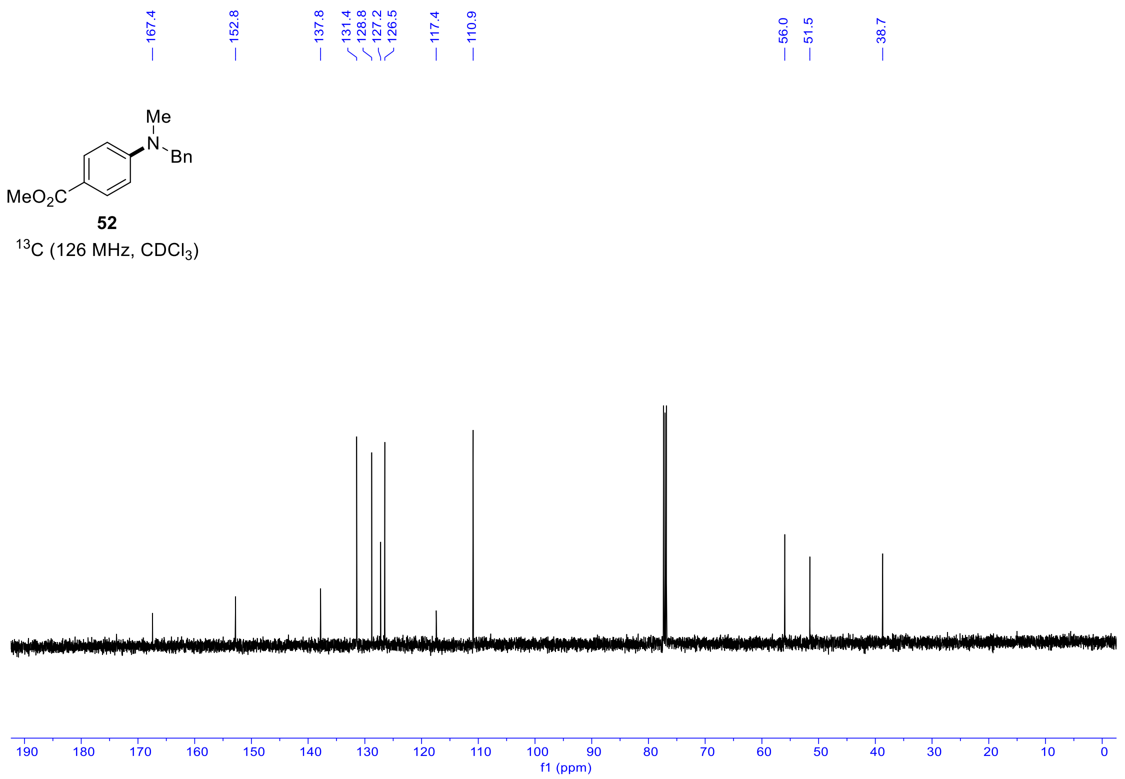

Supplementary Figure 156. ^{13}**C NMR** of compound **52**

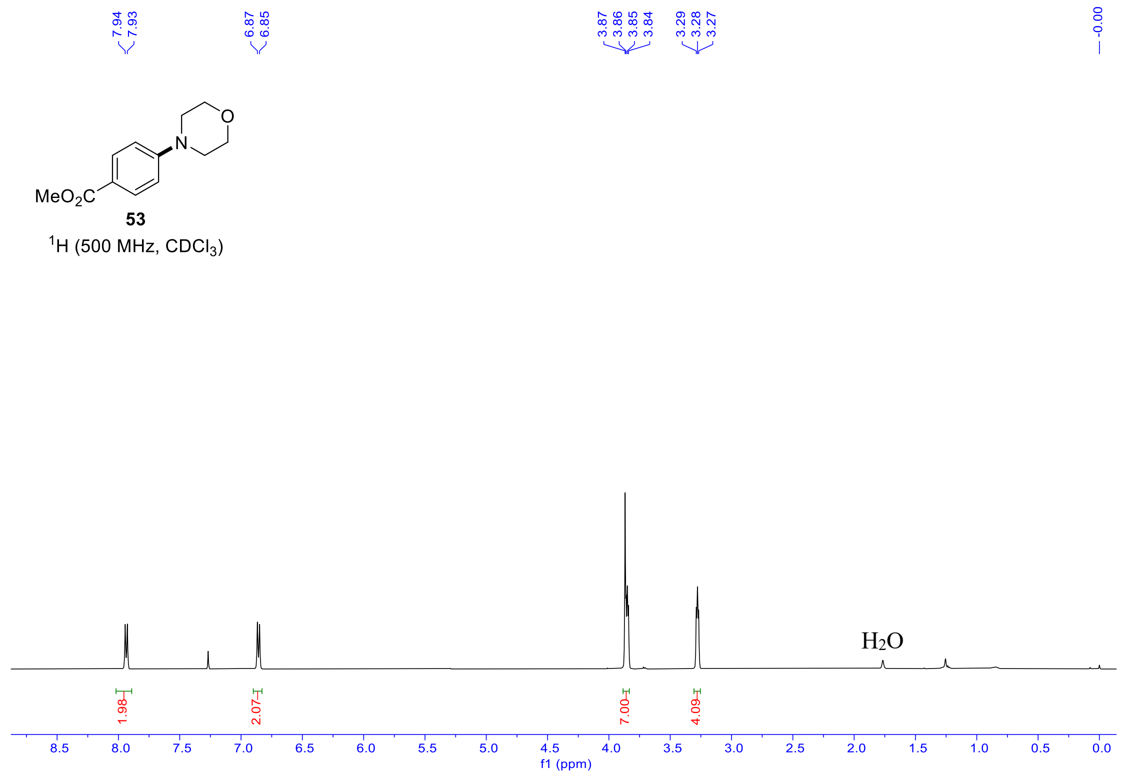

Supplementary Figure 157. ^{1}**H NMR** of compound **53**

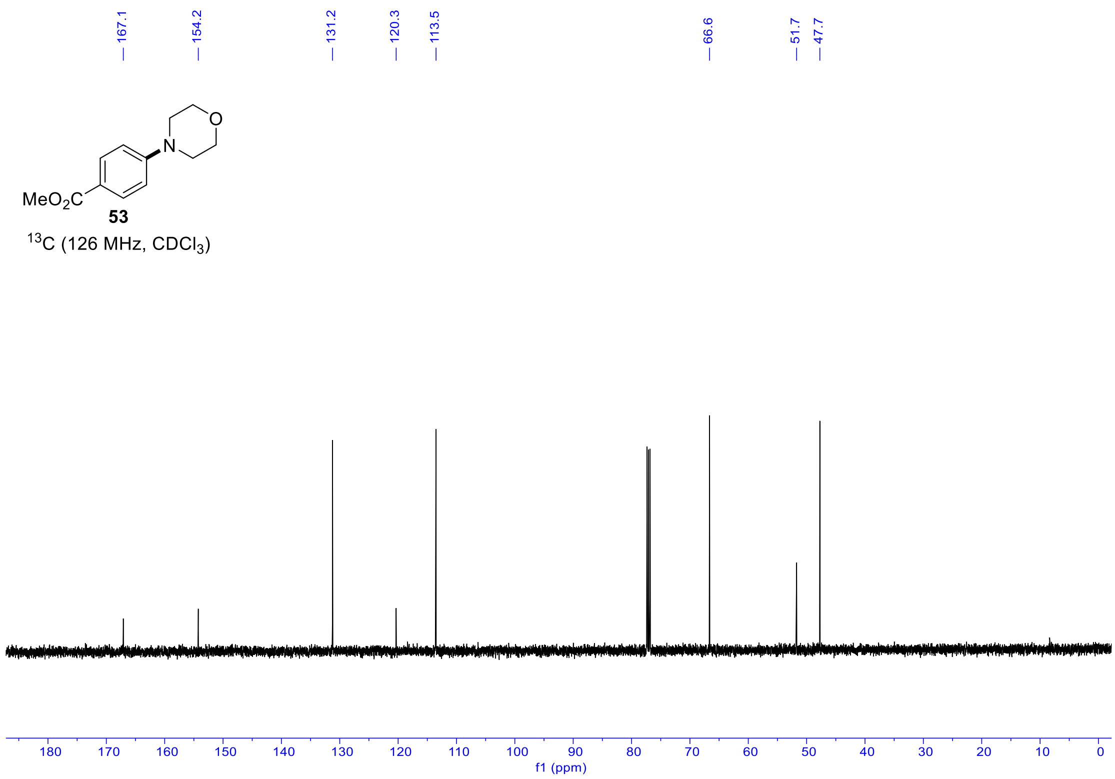

Supplementary Figure 158. ^{13}**C NMR** of compound **53**

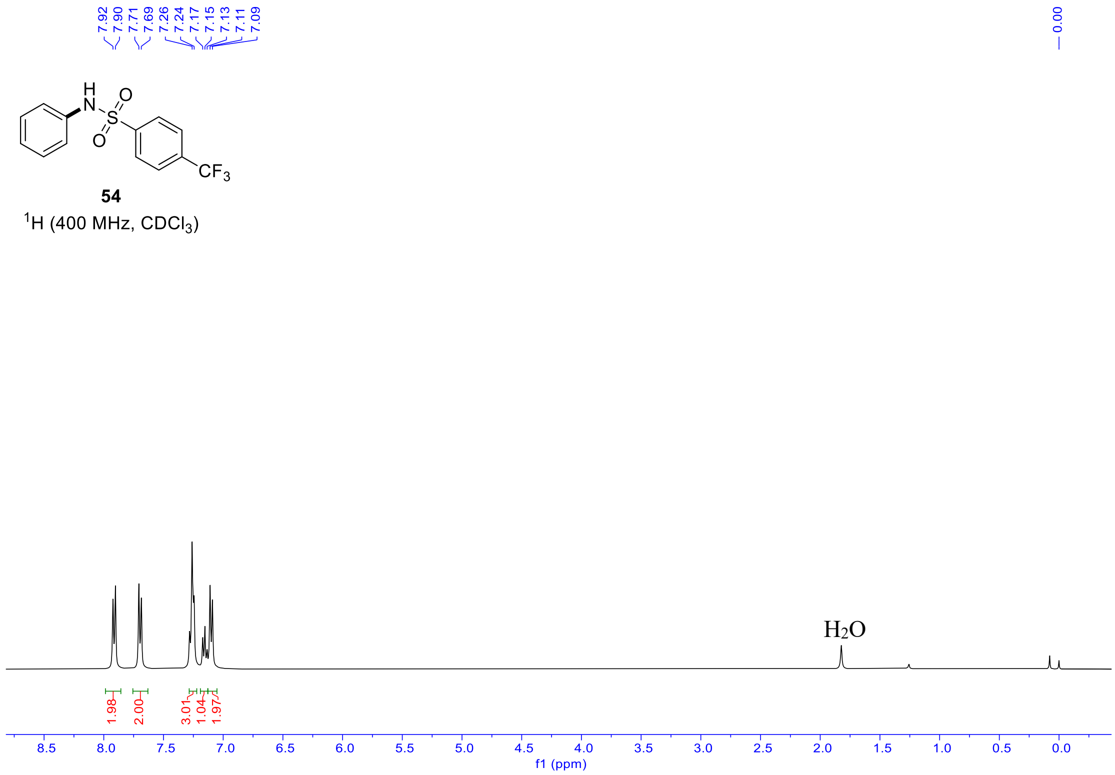

Supplementary Figure 159. ^{1}**H NMR** of compound **54**

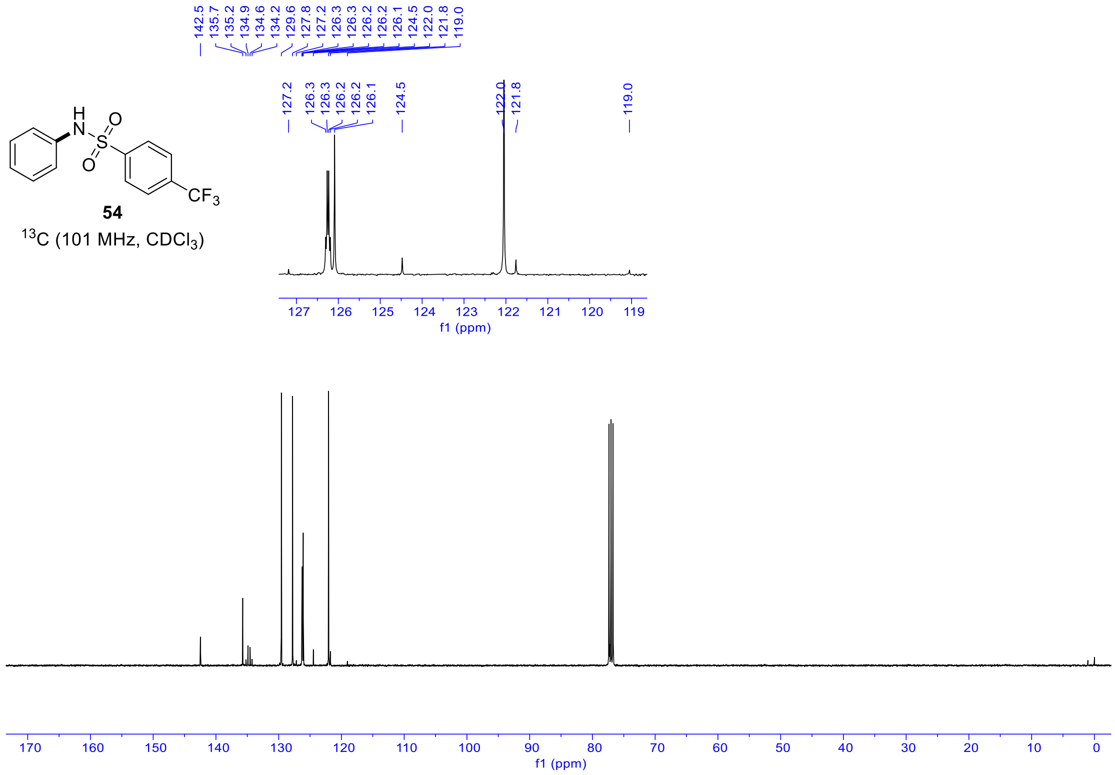

Supplementary Figure 160. ^{13}**C NMR** of compound **54**

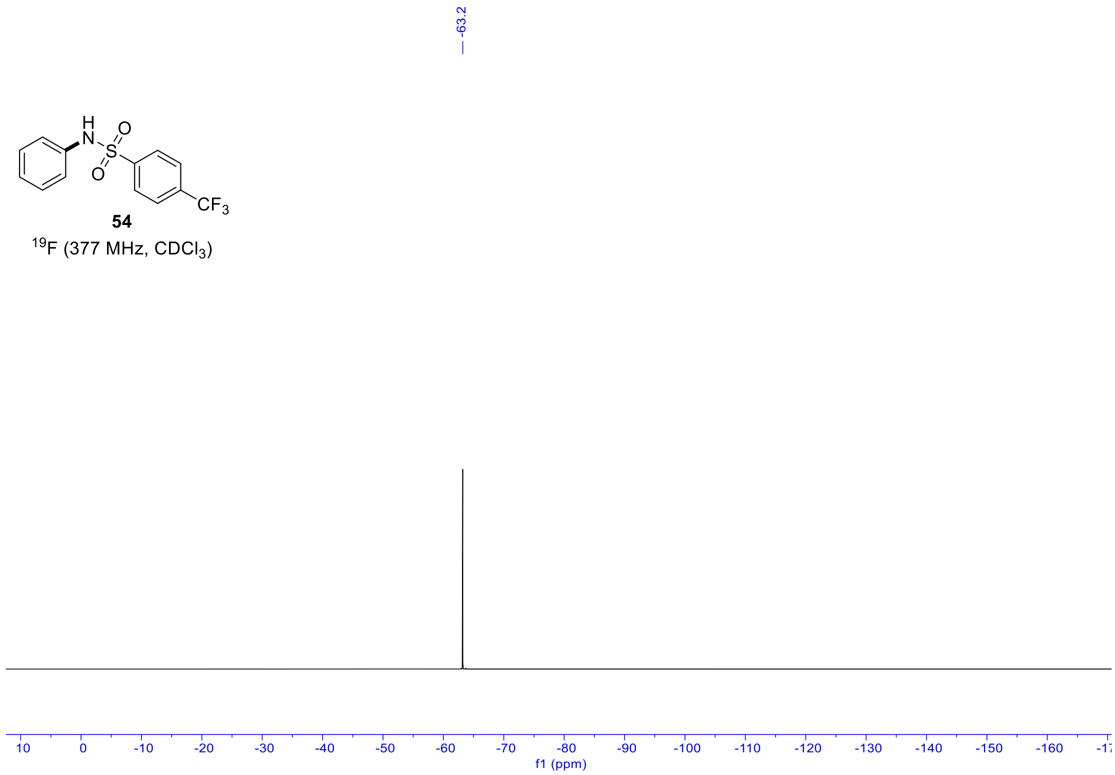

Supplementary Figure 161. ^{19}**F NMR** of compound **54**

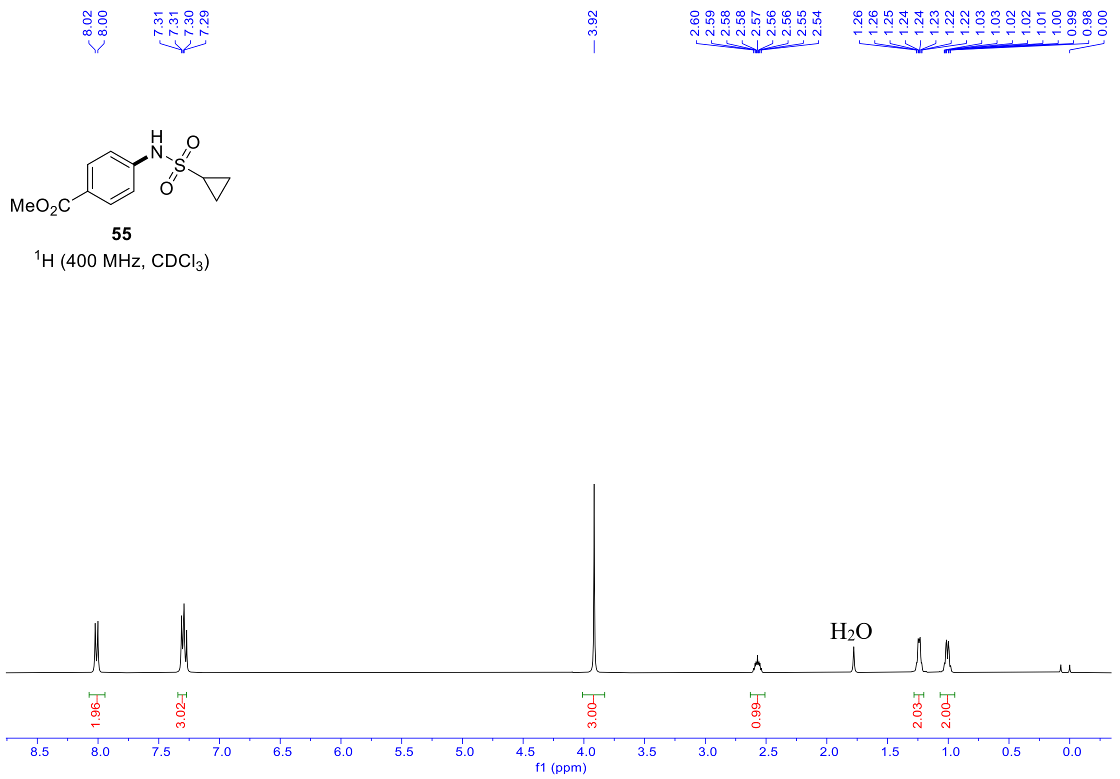

Supplementary Figure 162. ^{1}**H NMR** of compound **55**

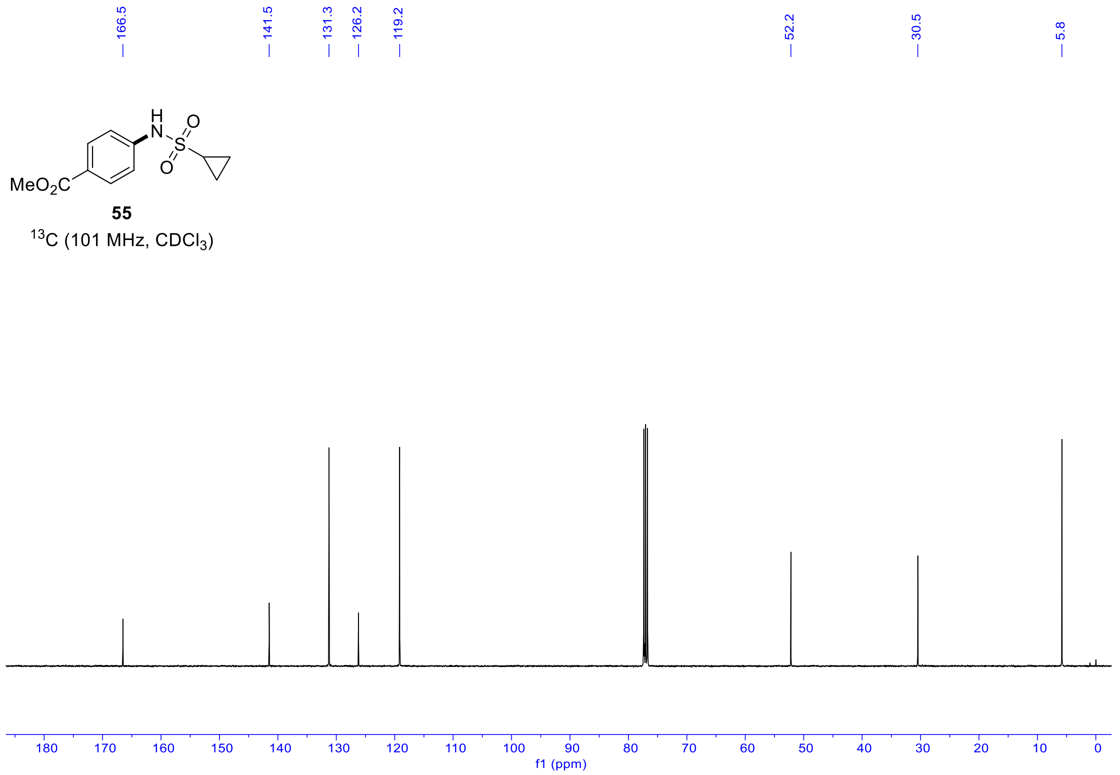

Supplementary Figure 163. ^{13}**C NMR** of compound **55**

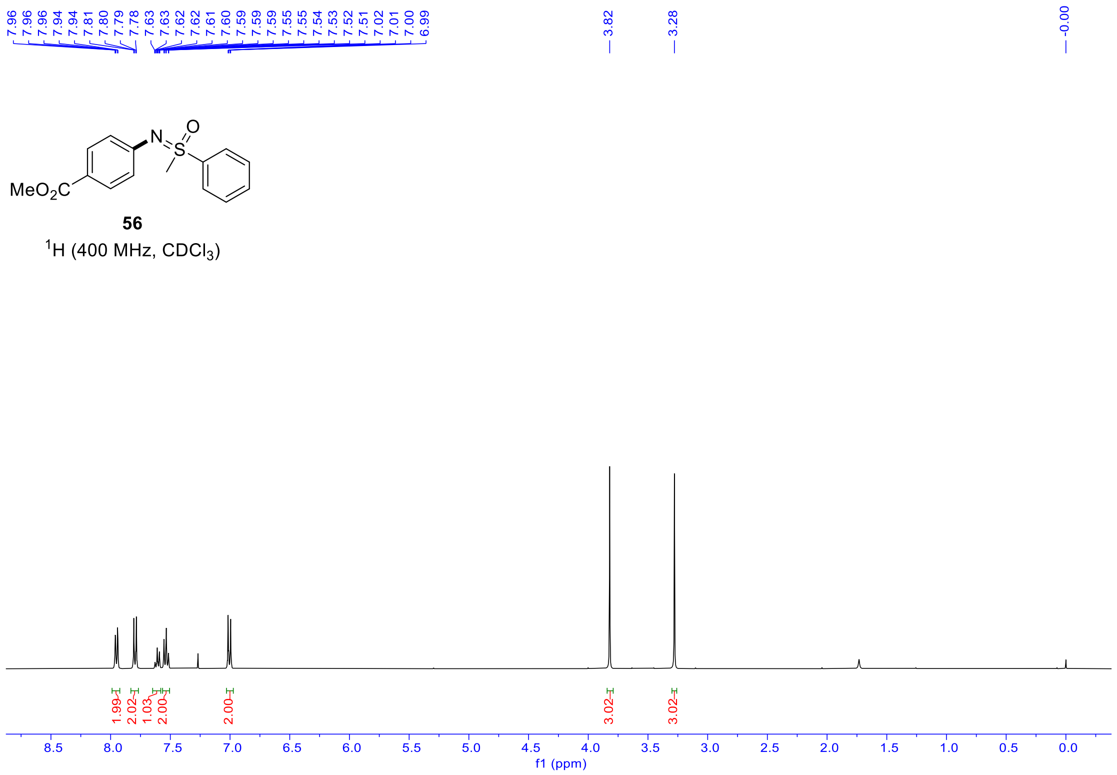

Supplementary Figure 164. ^{1}H NMR of compound **56**

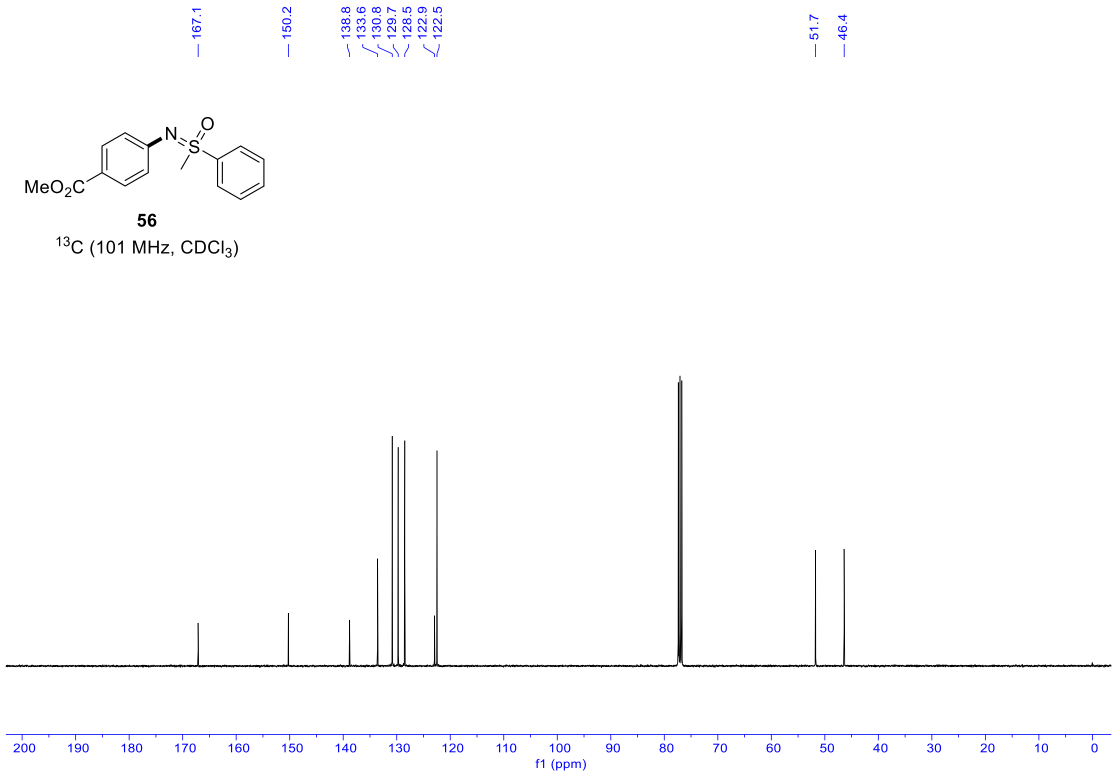

Supplementary Figure 165. ^{13}C NMR of compound **56**

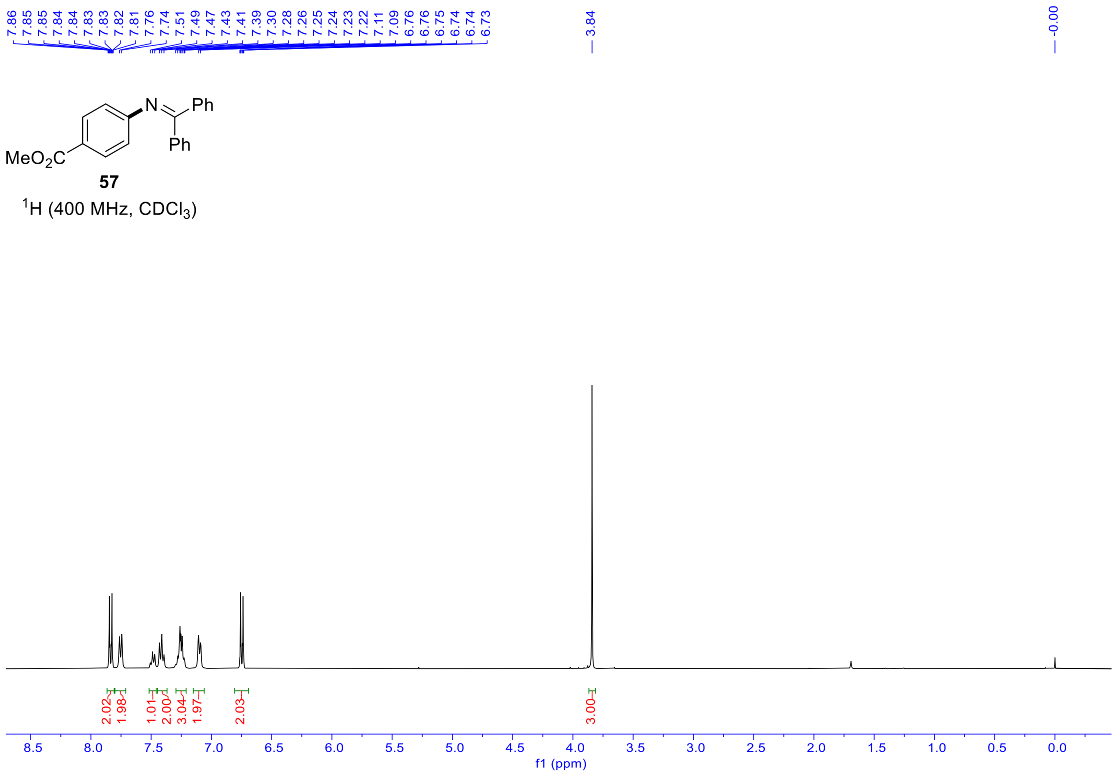

Supplementary Figure 166. ^{1}**H NMR** of compound **57**

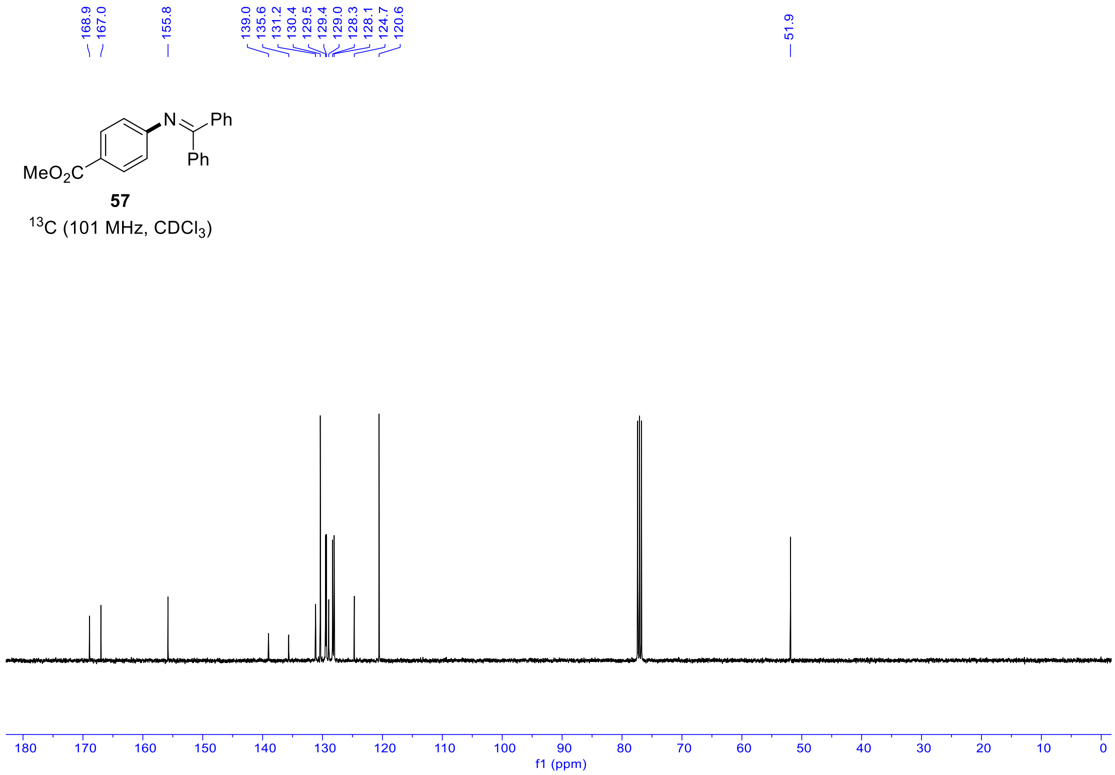

Supplementary Figure 167. ^{13}**C NMR** of compound **57**

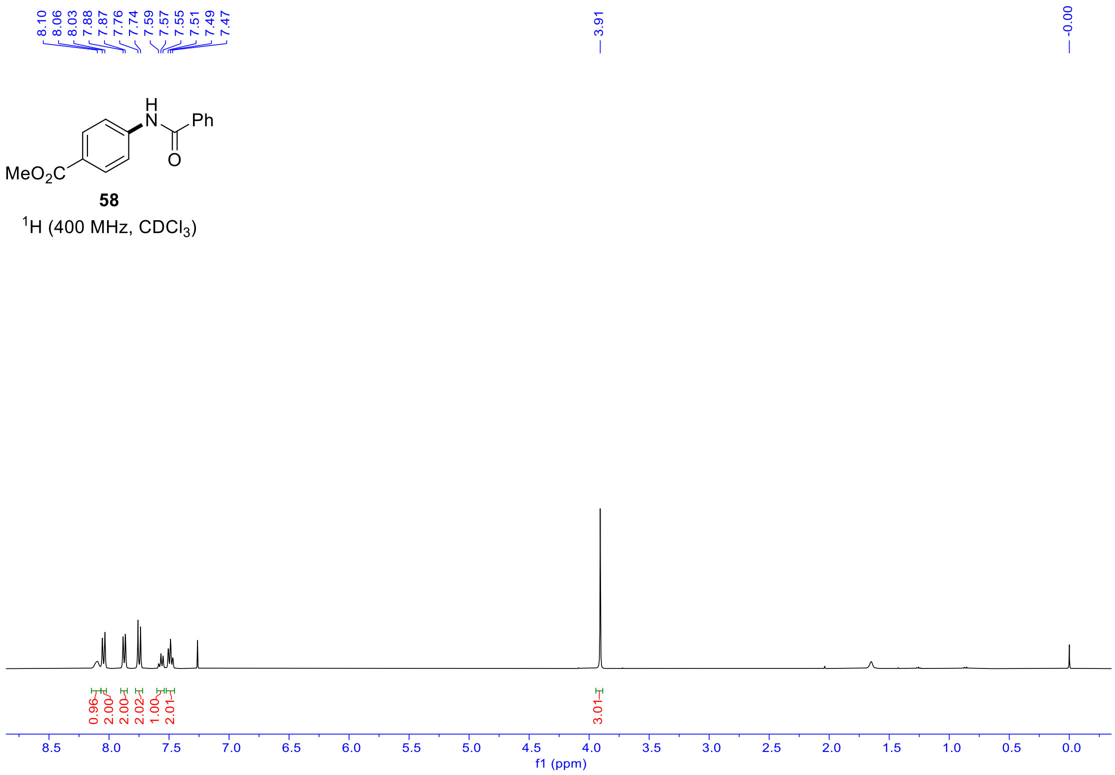

Supplementary Figure 168. ^{1}**H NMR** of compound **58**

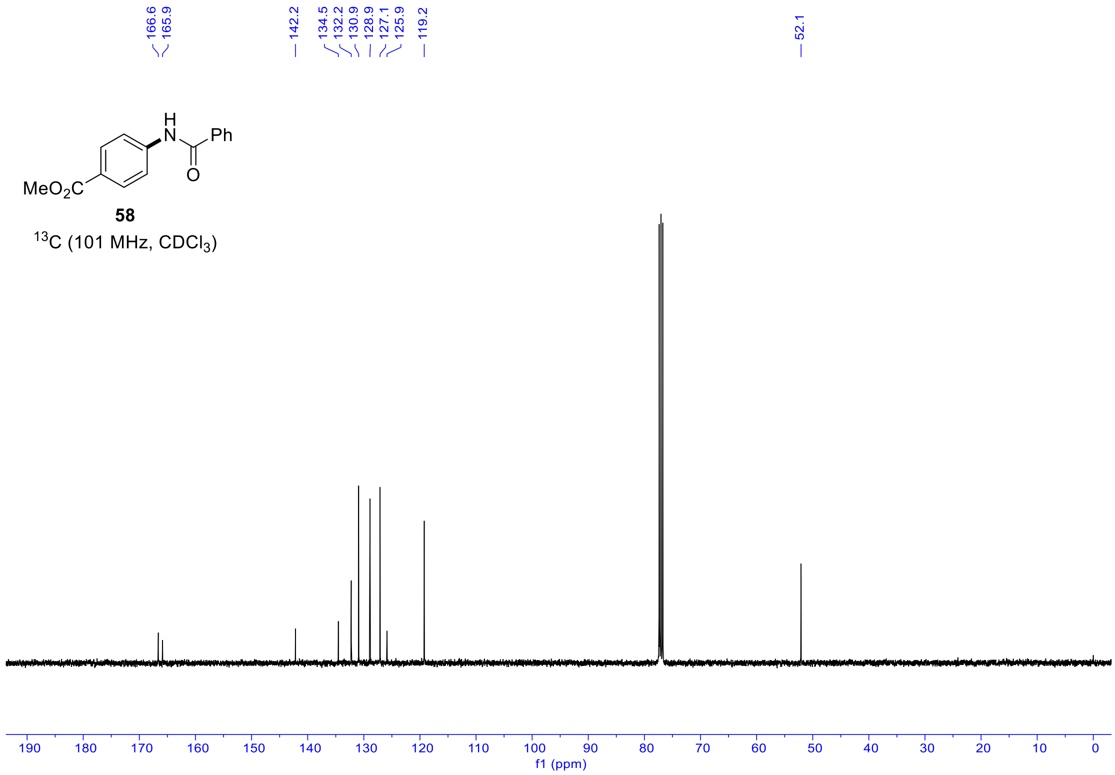

Supplementary Figure 169. ^{13}**C NMR** of compound **58**

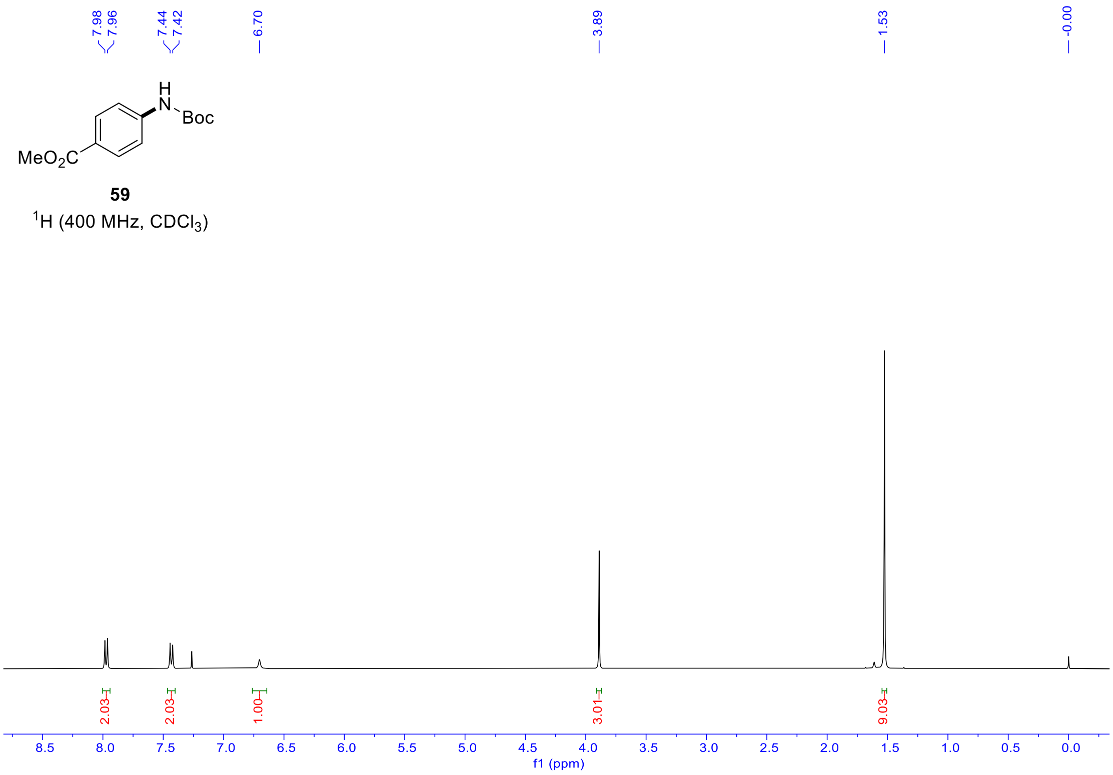

Supplementary Figure 170. ^{1}**H NMR** of compound **59**

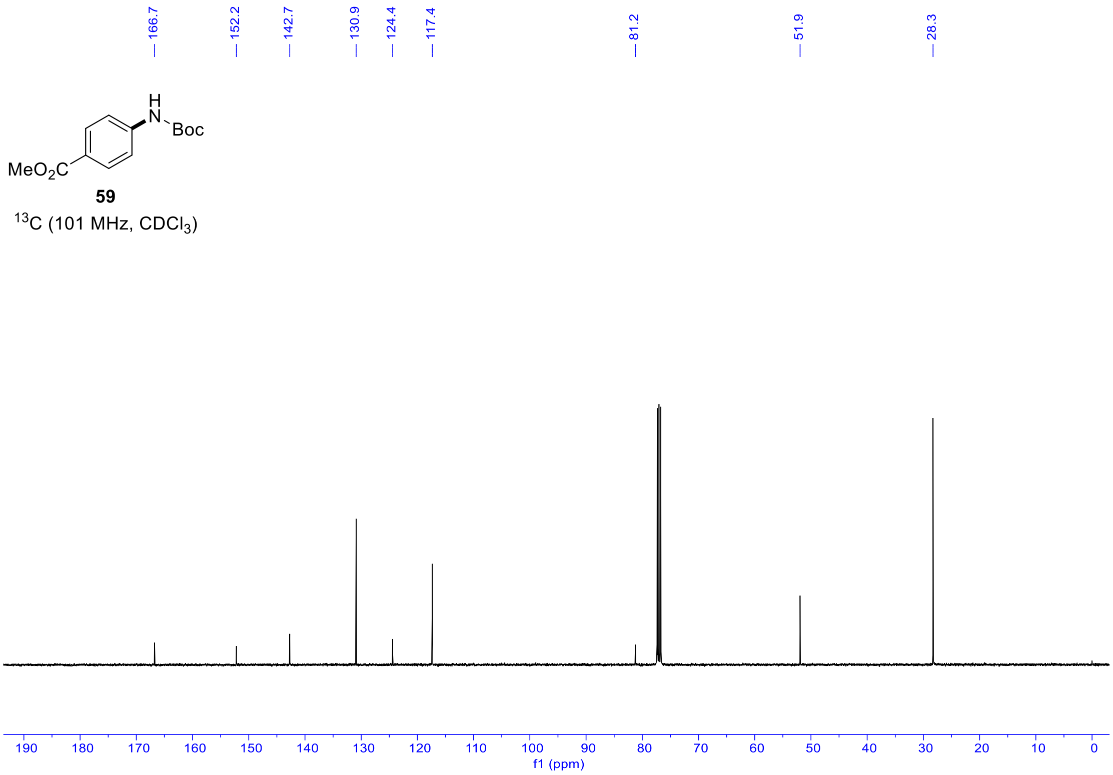

Supplementary Figure 171. ^{13}**C NMR** of compound **59**

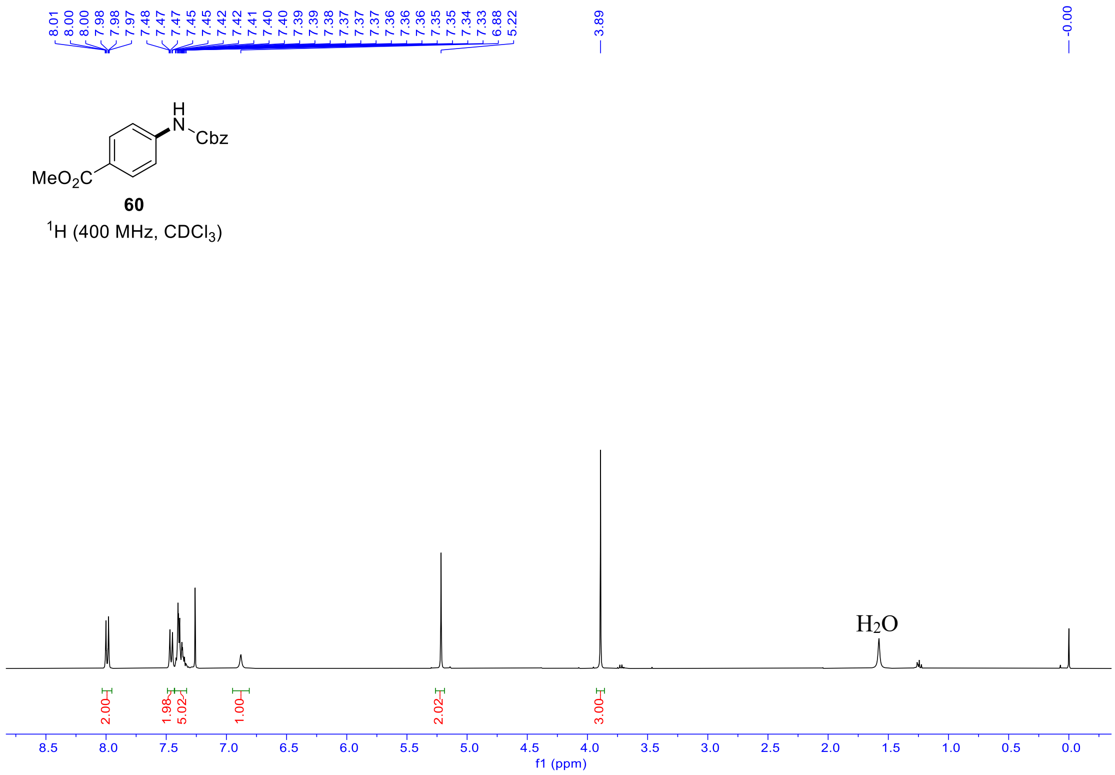

Supplementary Figure 172. ^{1}**H NMR** of compound **60**

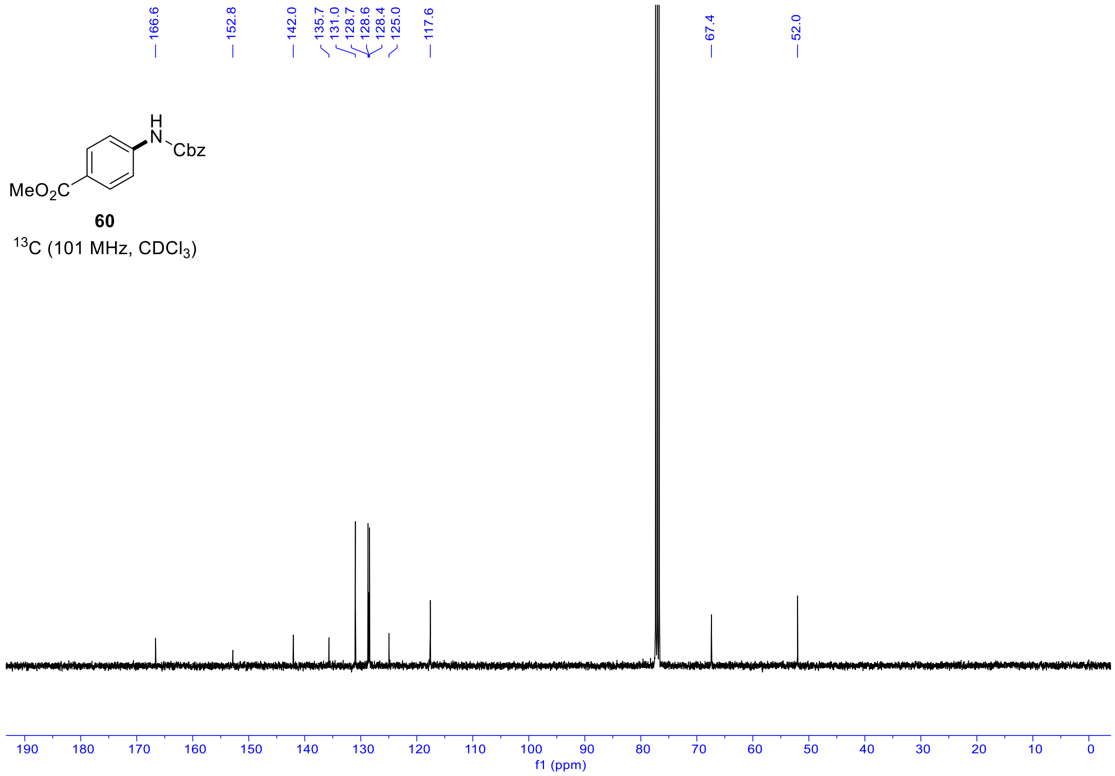

Supplementary Figure 173. ^{13}**C NMR** of compound **60**

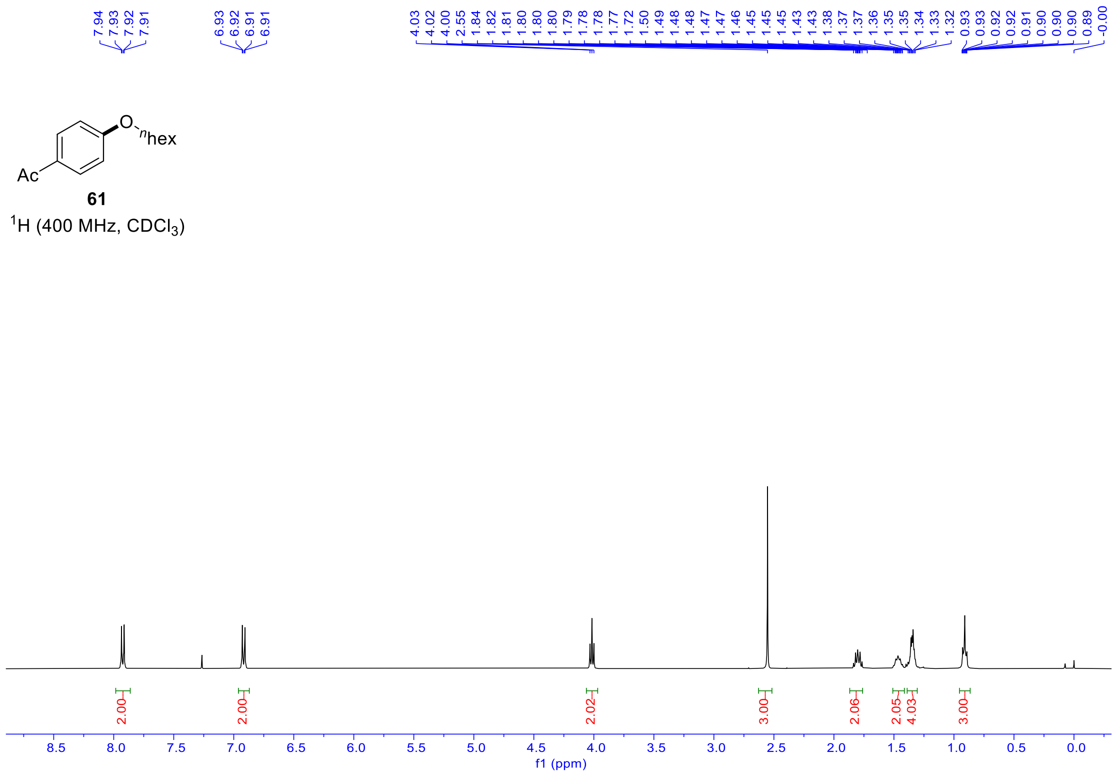

Supplementary Figure 174. ^{1}H NMR of compound **61**

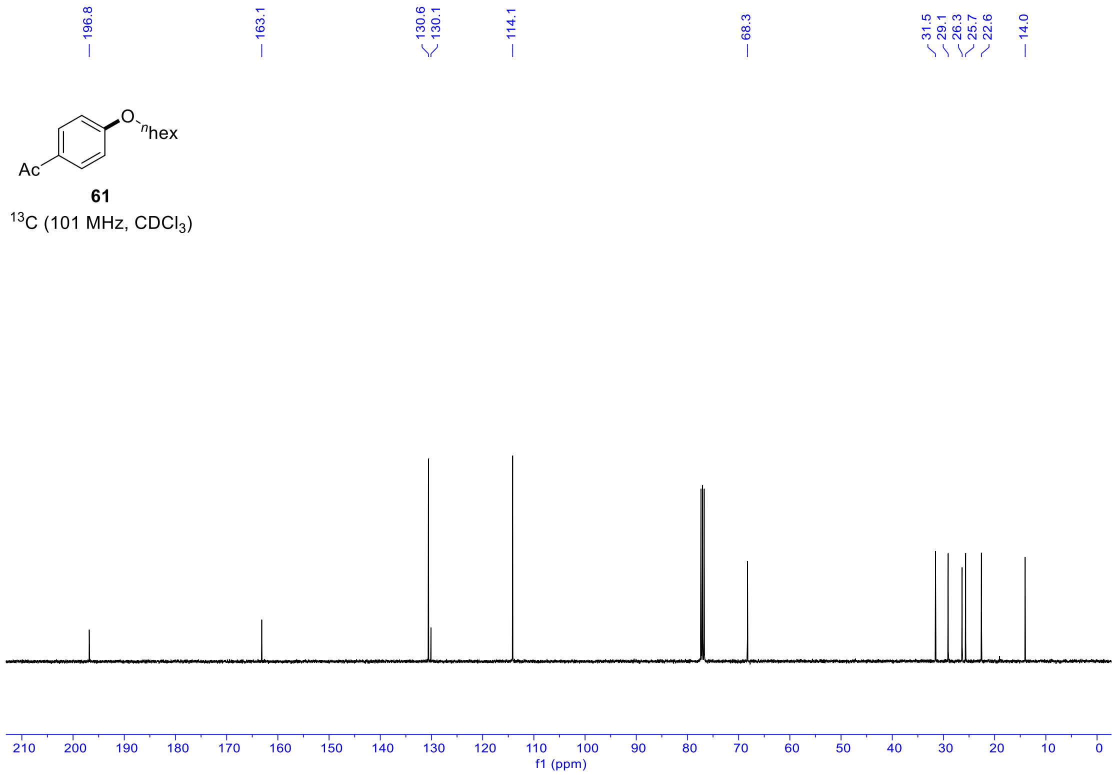

Supplementary Figure 175. ^{13}C NMR of compound **61**

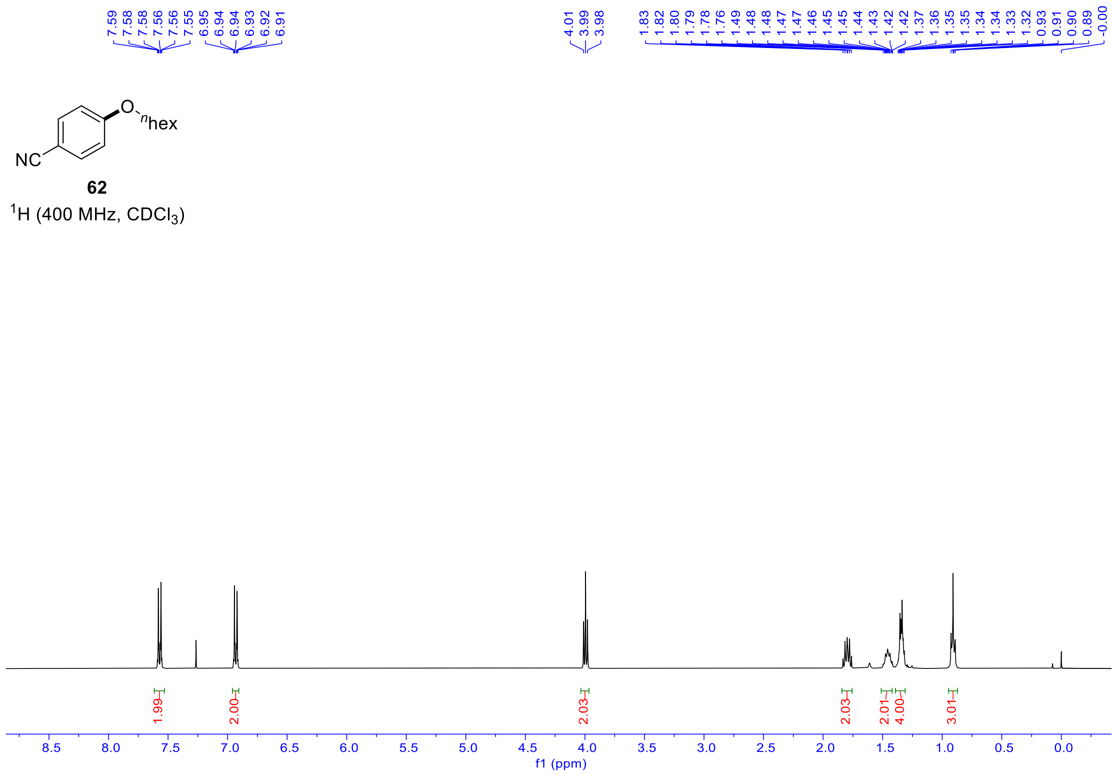

Supplementary Figure 176. ^{1}H NMR of compound **62**

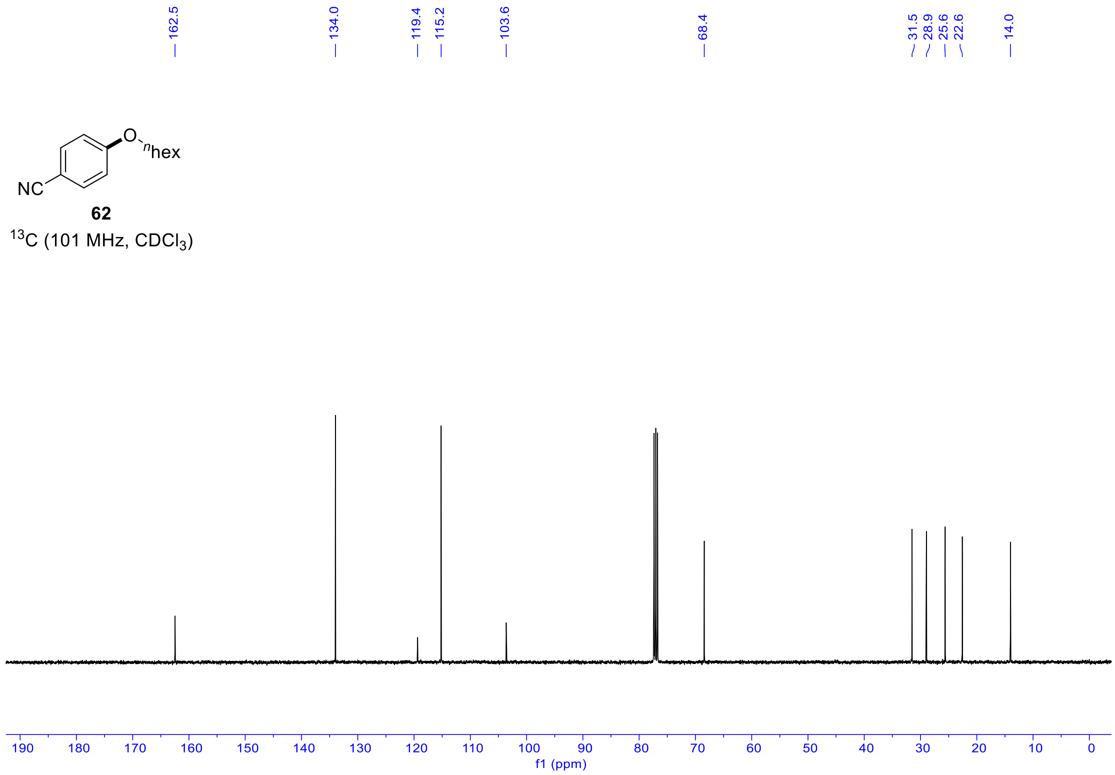

Supplementary Figure 177. ^{13}C NMR of compound **62**

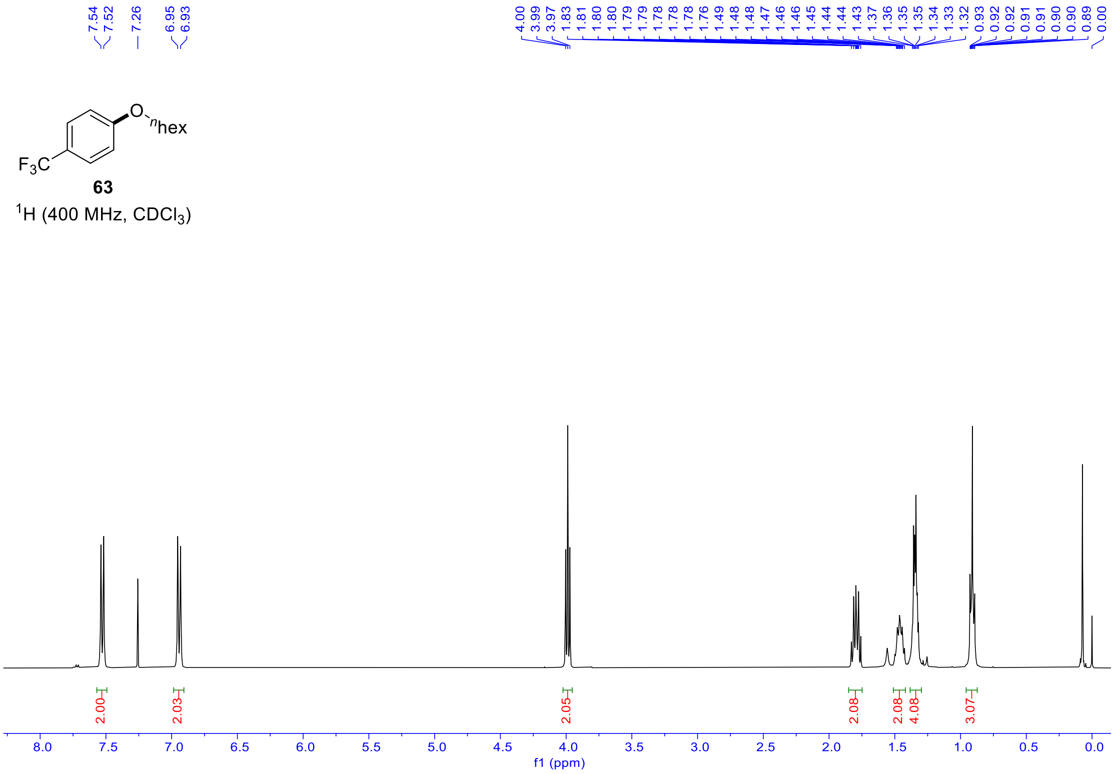

Supplementary Figure 178. **^{1}H NMR** of compound **63**

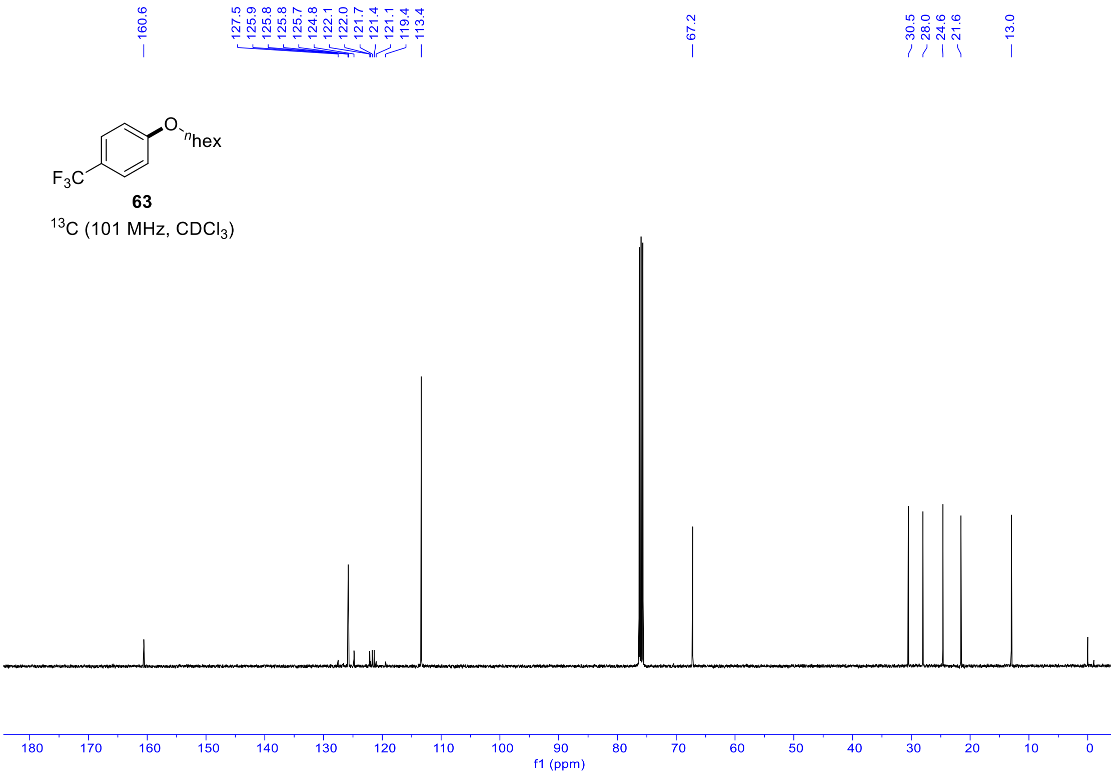

Supplementary Figure 179. **^{13}C NMR** of compound **63**

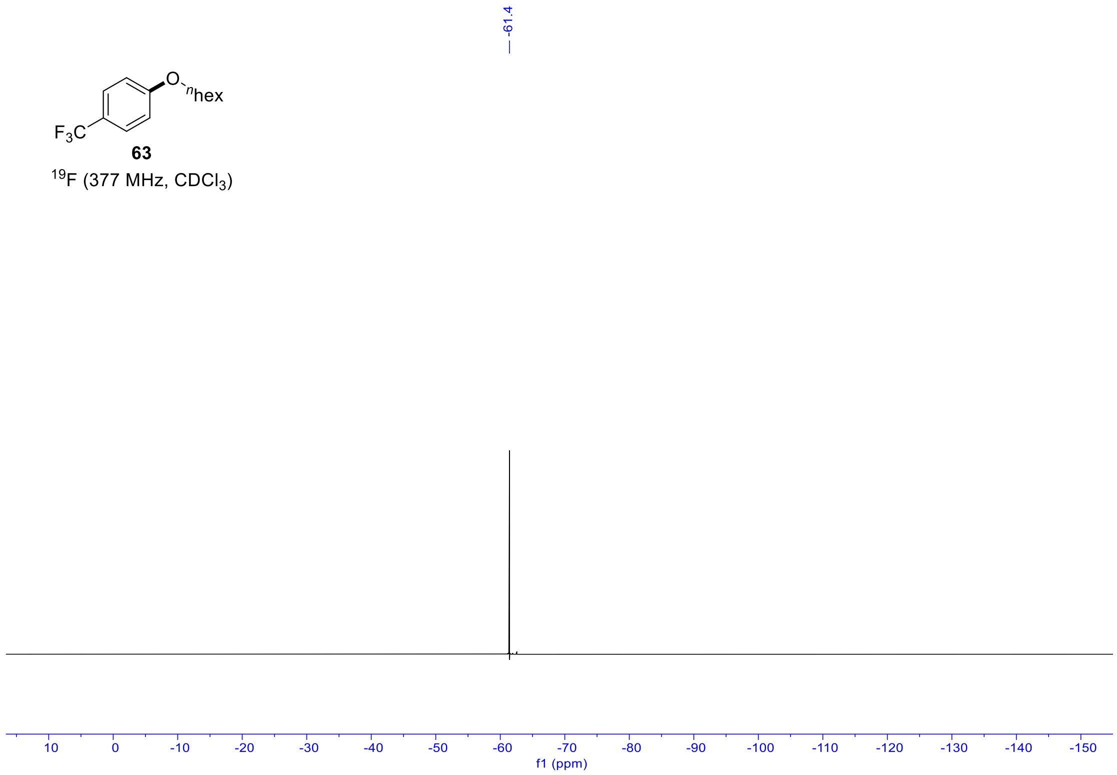

Supplementary Figure 180. ^{19}F NMR of compound **63**

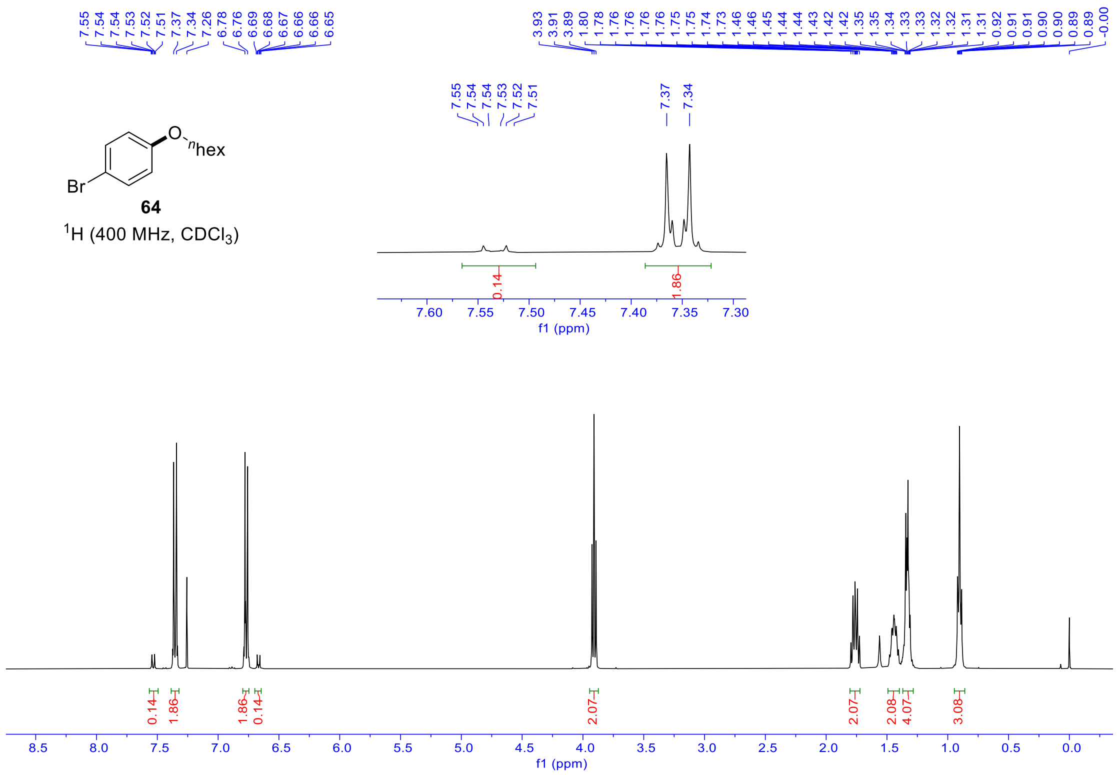

Supplementary Figure 181. ^{1}H NMR of compound **64**

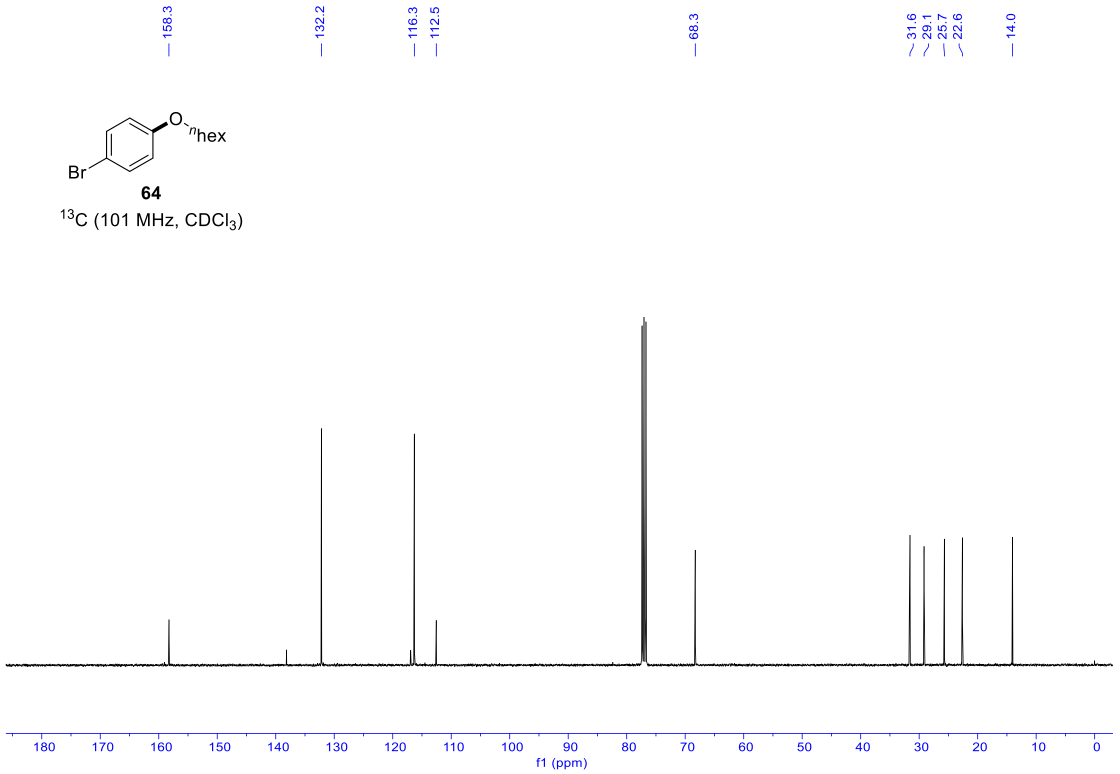

Supplementary Figure 182. ^{13}**C NMR** of compound **64**

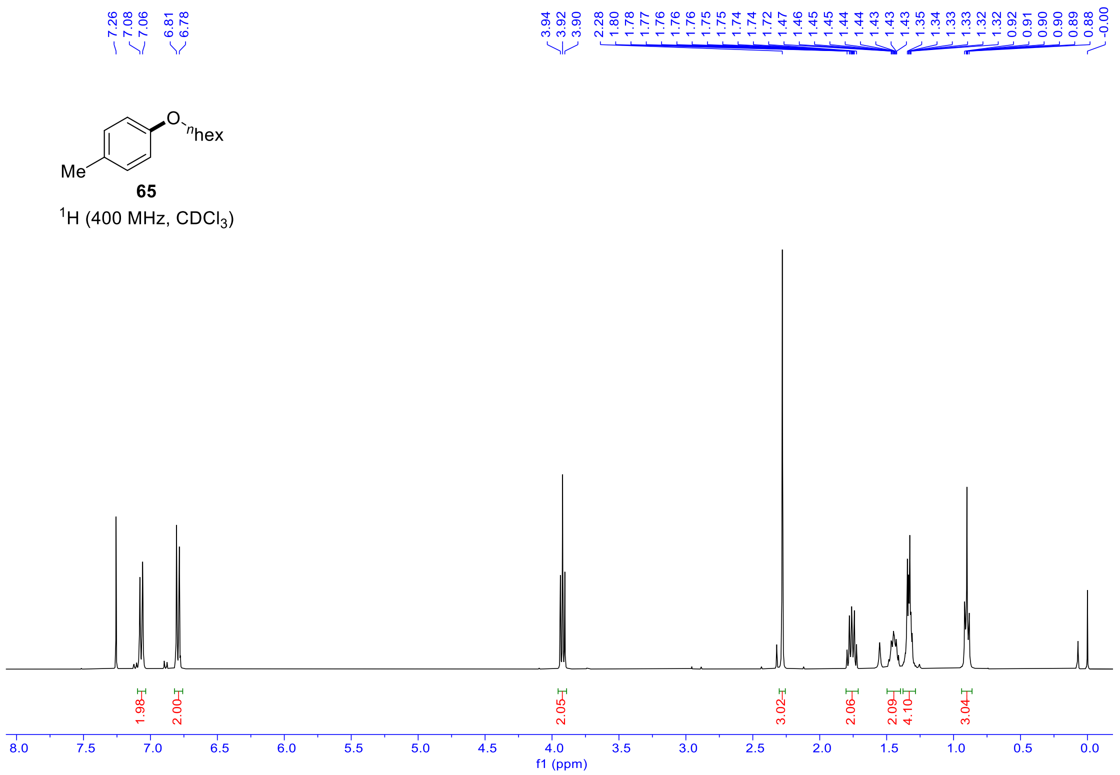

Supplementary Figure 183. ^{1}**H NMR** of compound **65**

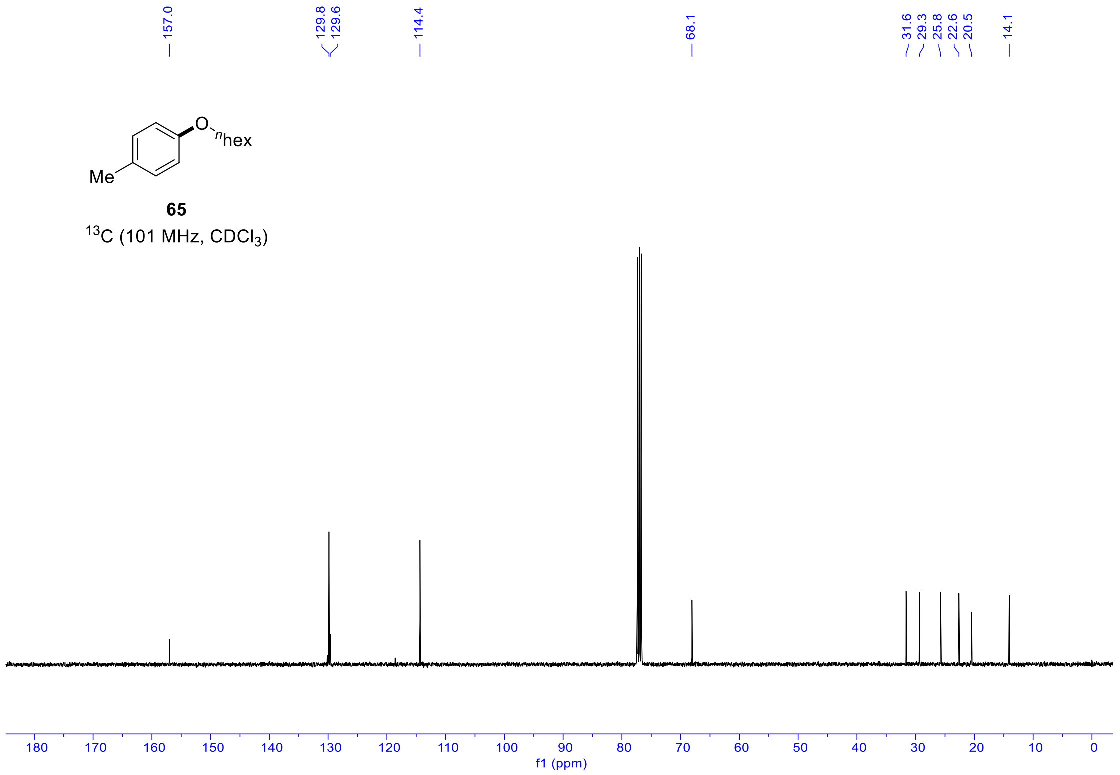

Supplementary Figure 184. ^{13}**C NMR** of compound **65**

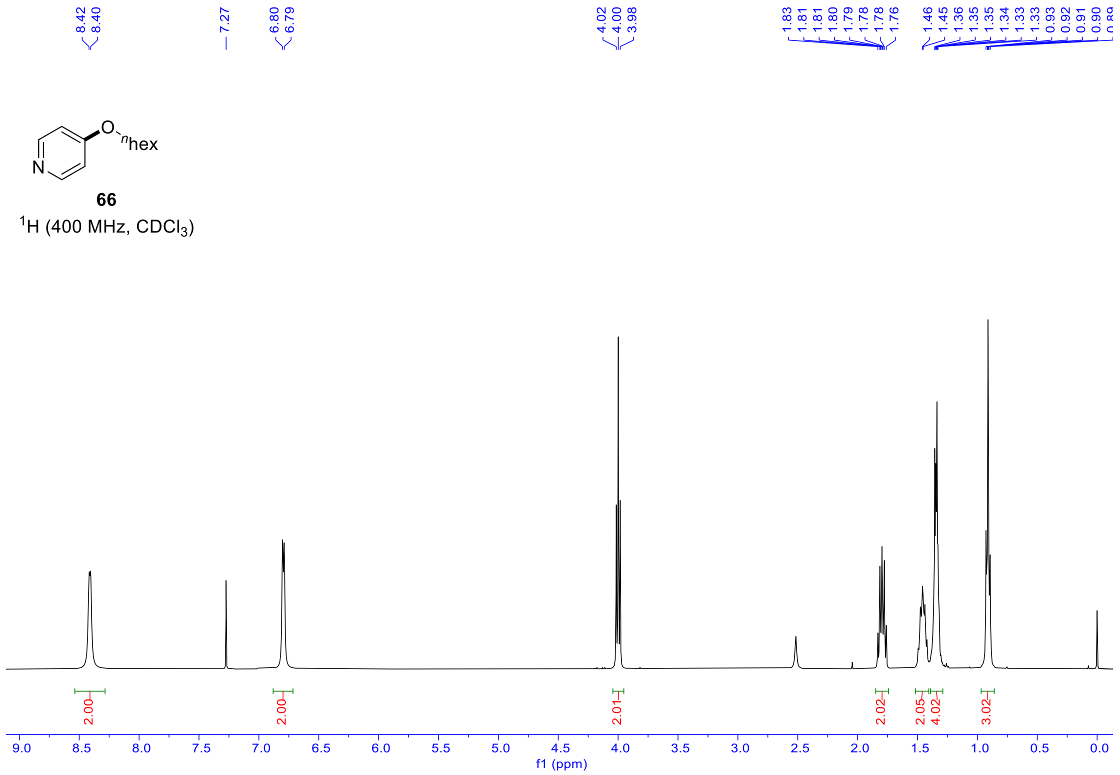

Supplementary Figure 185. ^{1}**H NMR** of compound **66**

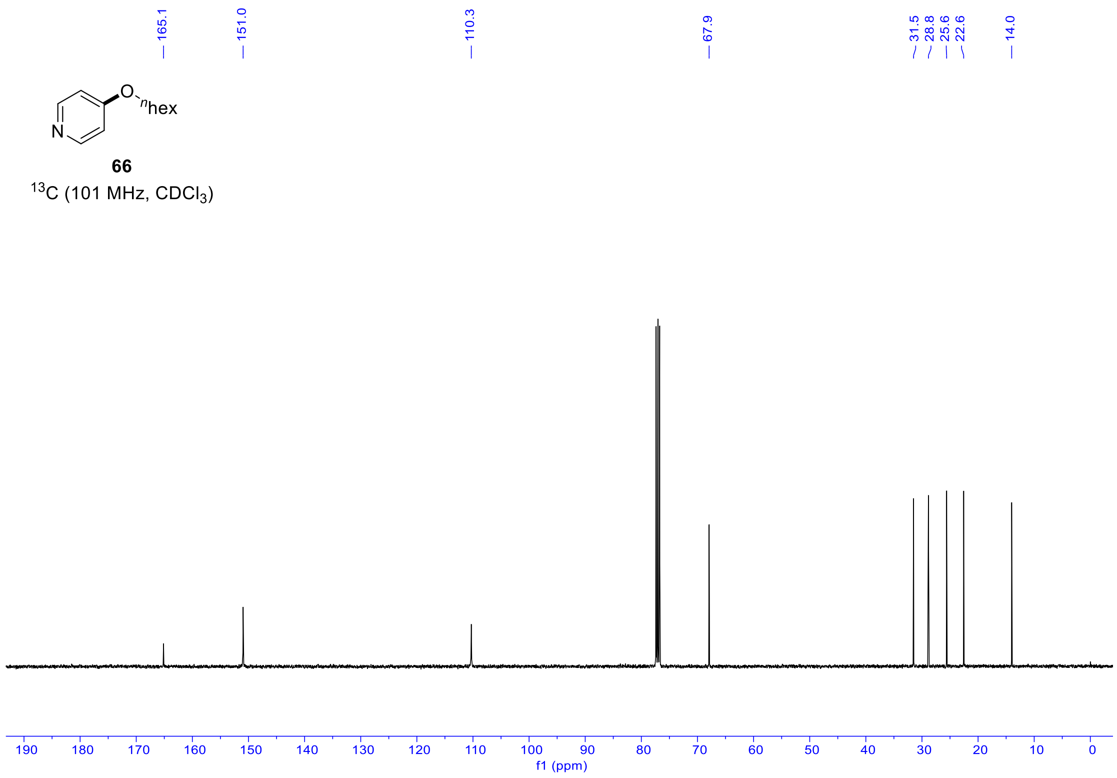

Supplementary Figure 186. ^{13}**C NMR** of compound **66**

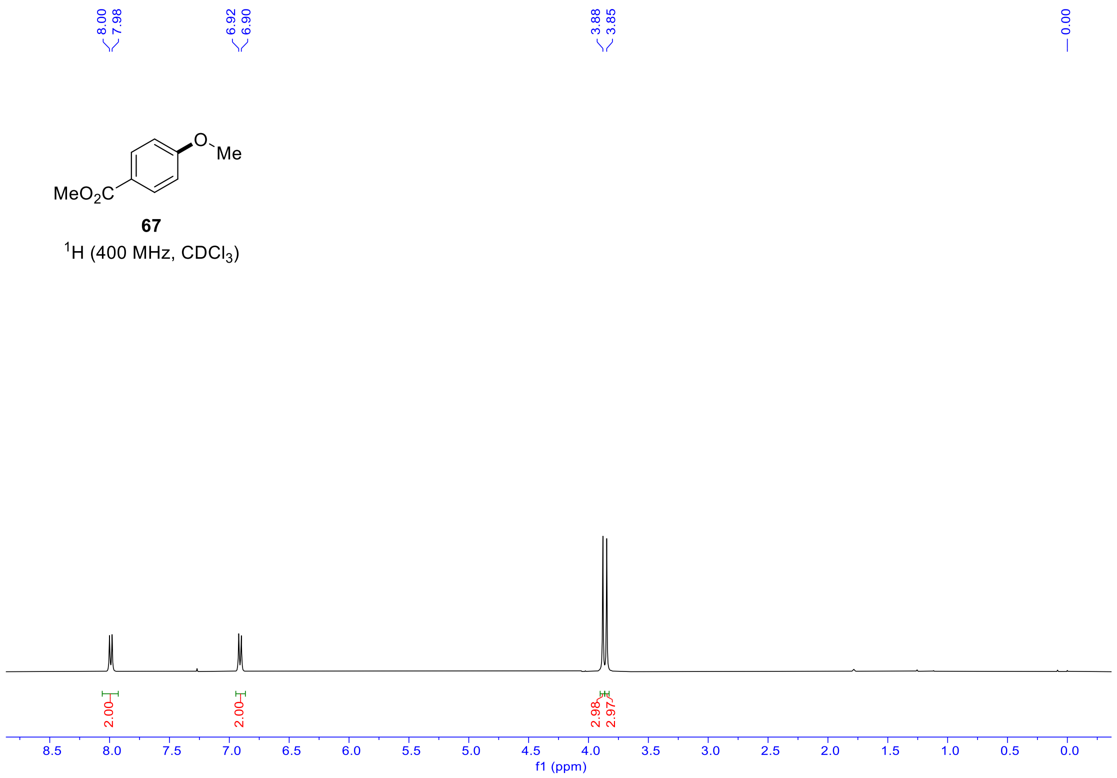

Supplementary Figure 187. ^{13}**C NMR** of compound **67**

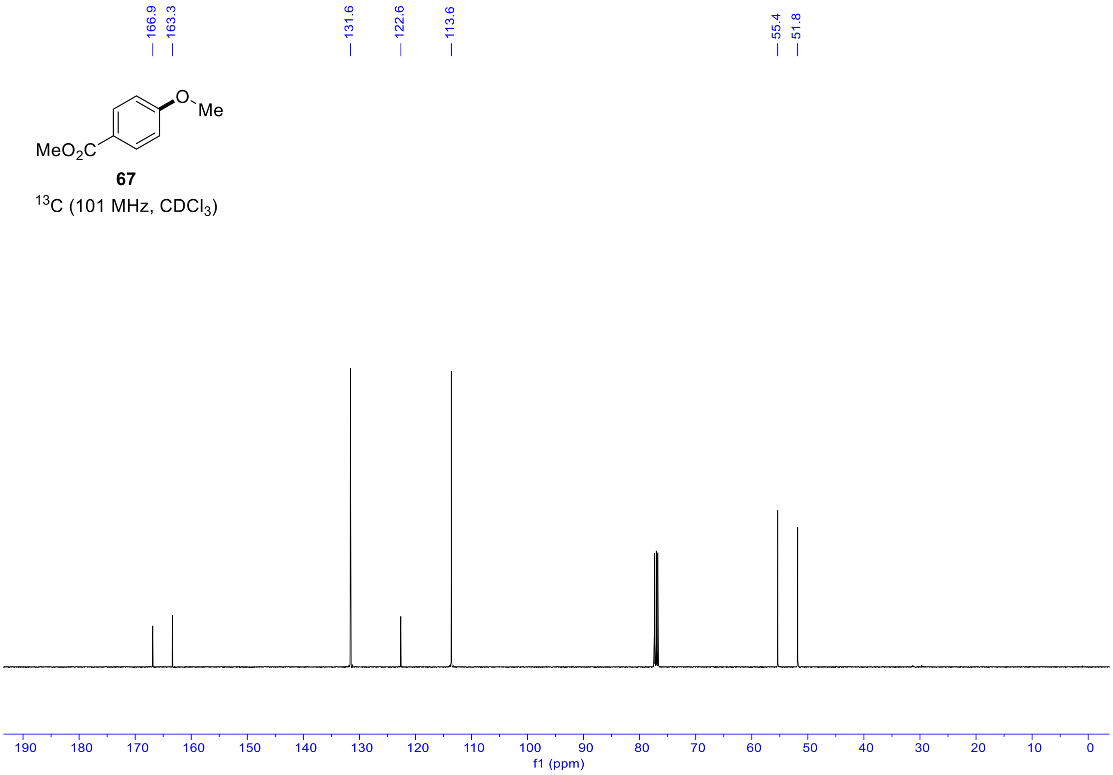

Supplementary Figure 188. ^{13}**C NMR** of compound **67**

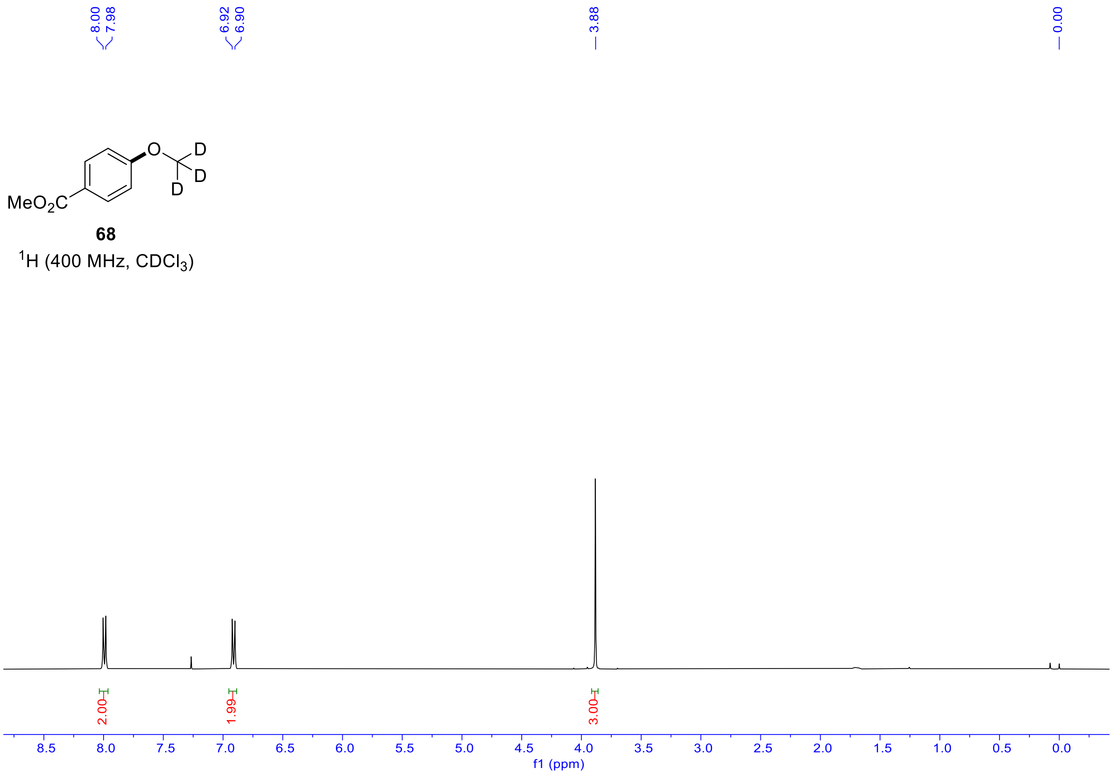

Supplementary Figure 189. ^{1}**H NMR** of compound **68**

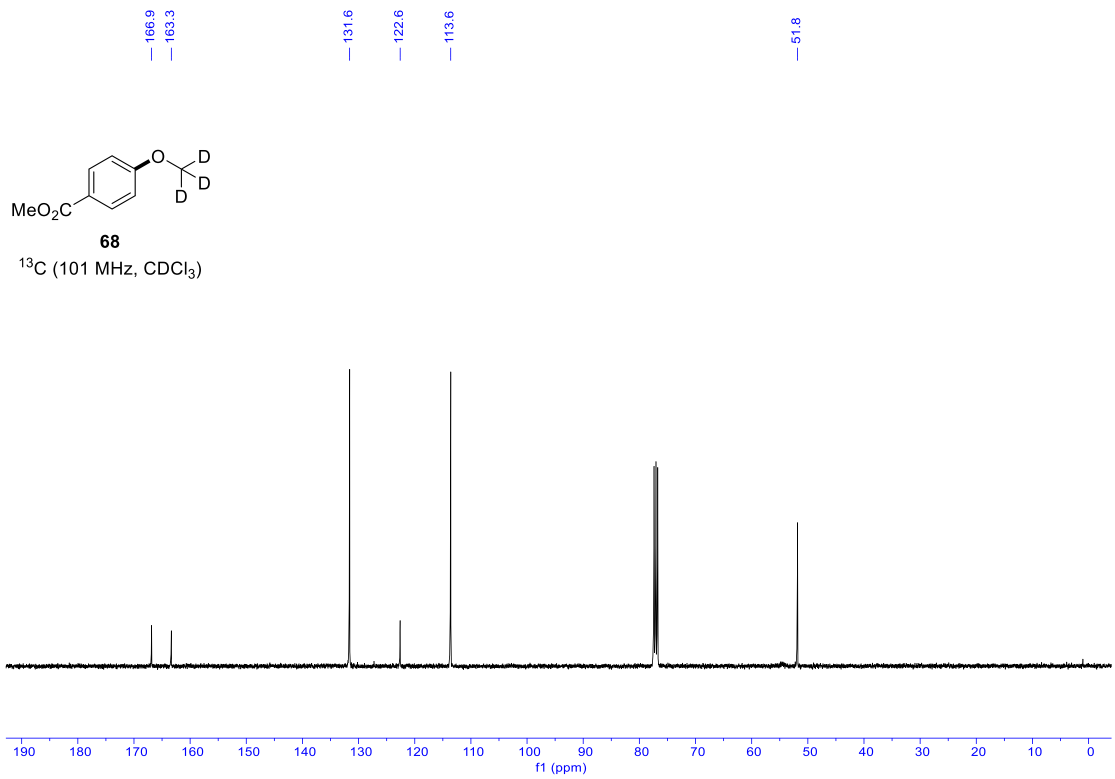

Supplementary Figure 190. ^{13}**C NMR** of compound **68**

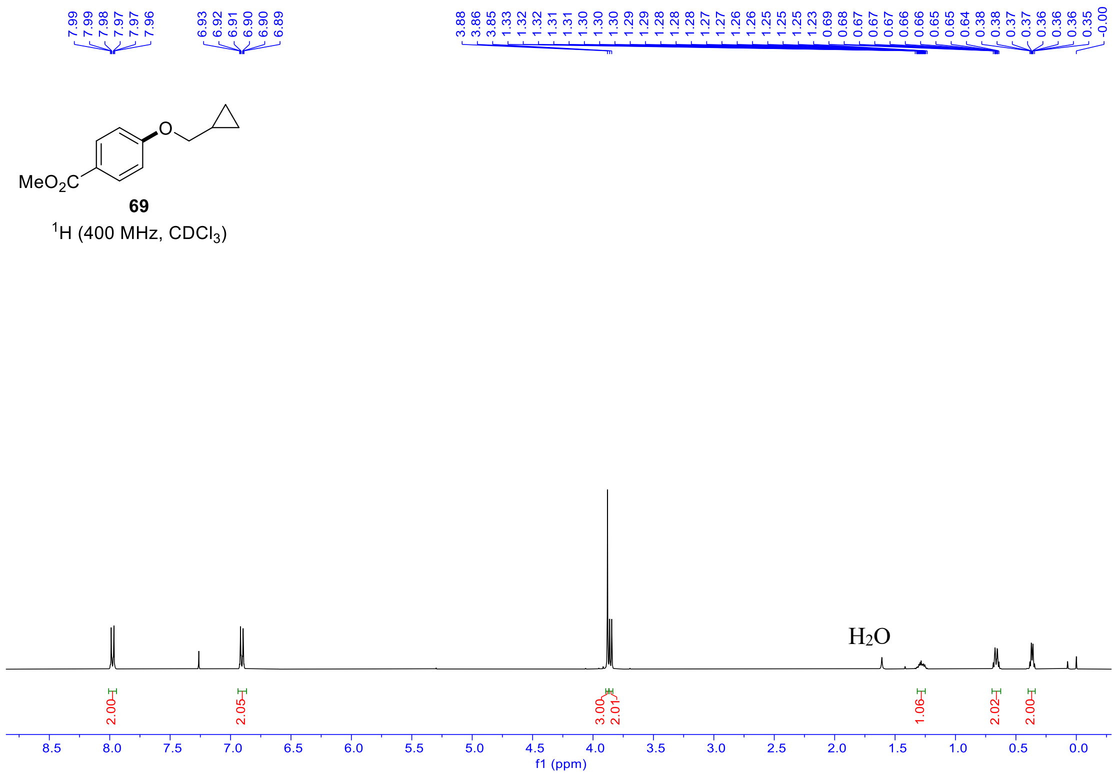

Supplementary Figure 191. ^{1}**H NMR** of compound **69**

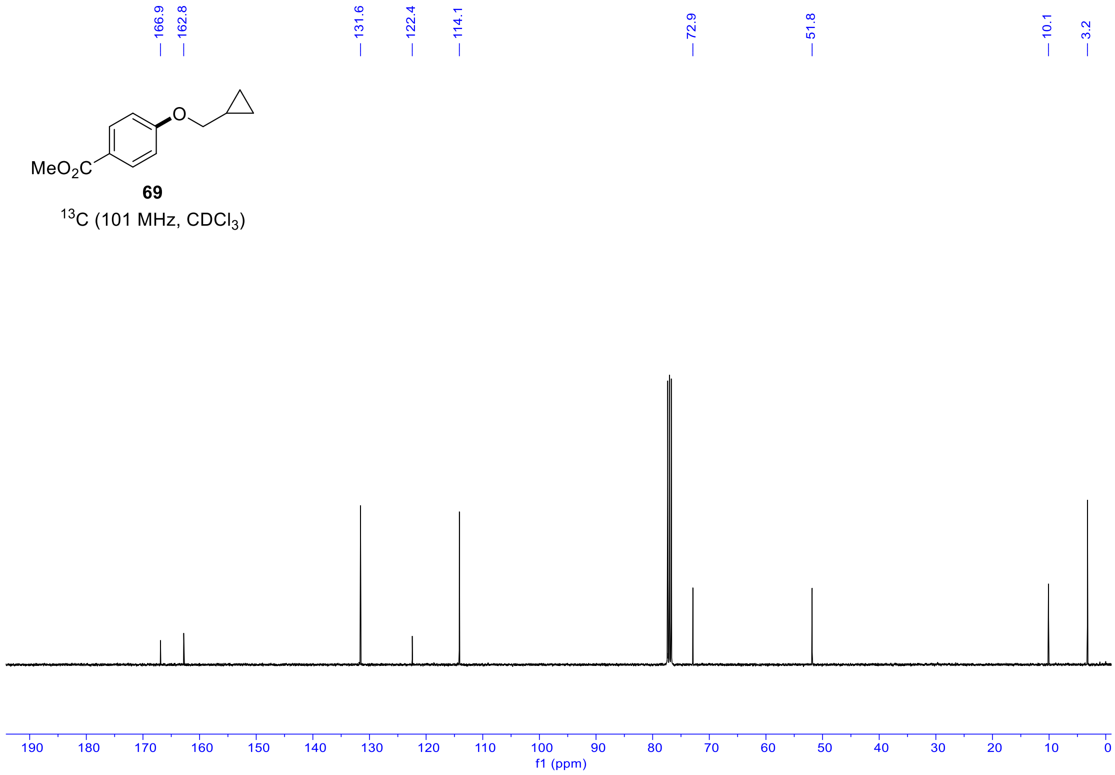

Supplementary Figure 192. ^{13}**C NMR** of compound **69**

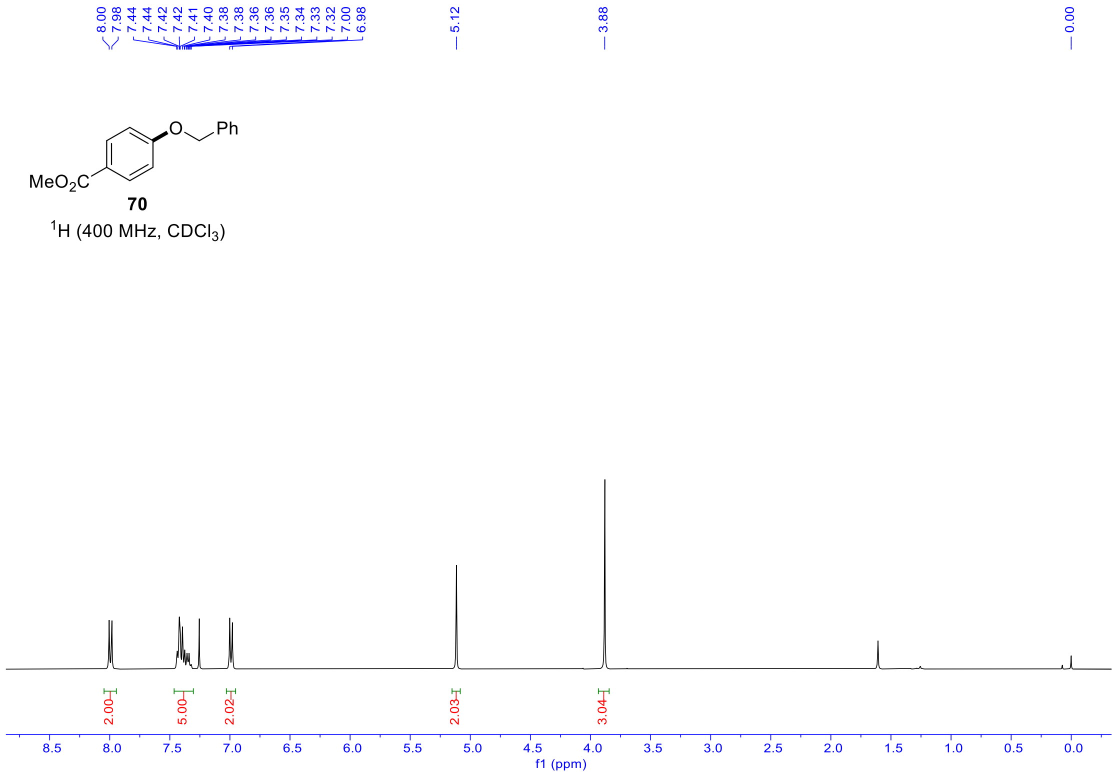

Supplementary Figure 193. ^{1}**H NMR** of compound **70**

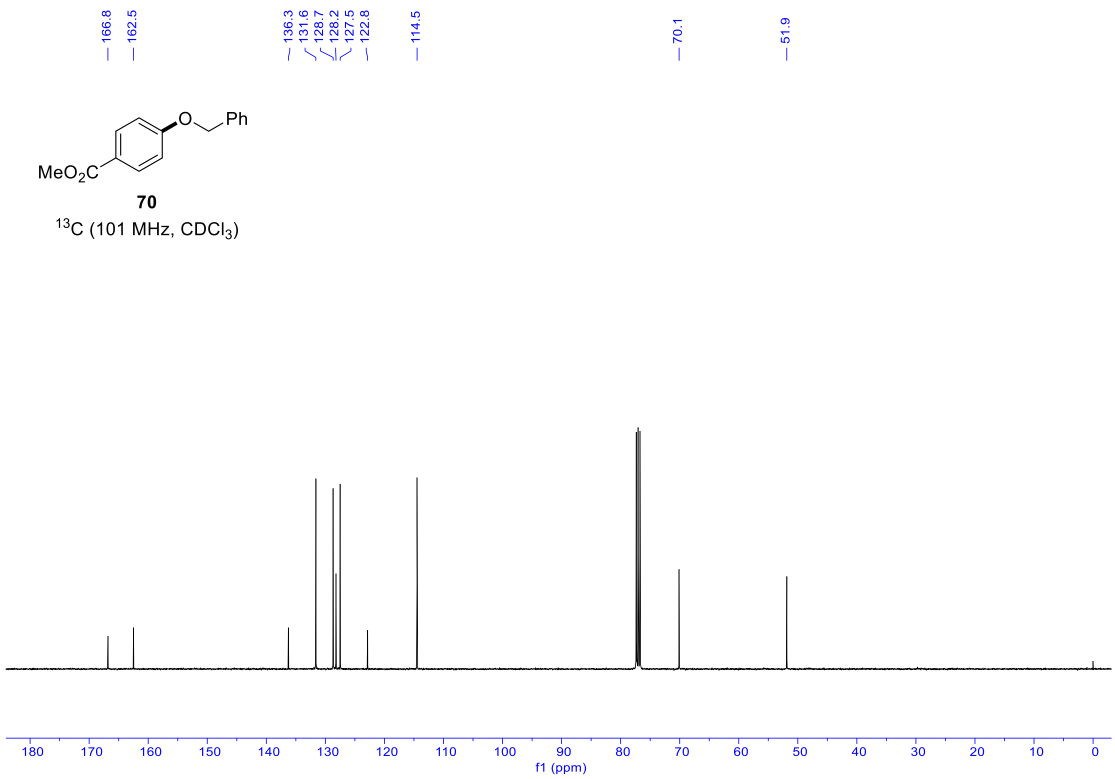

Supplementary Figure 194. ^{13}**C NMR** of compound **70**

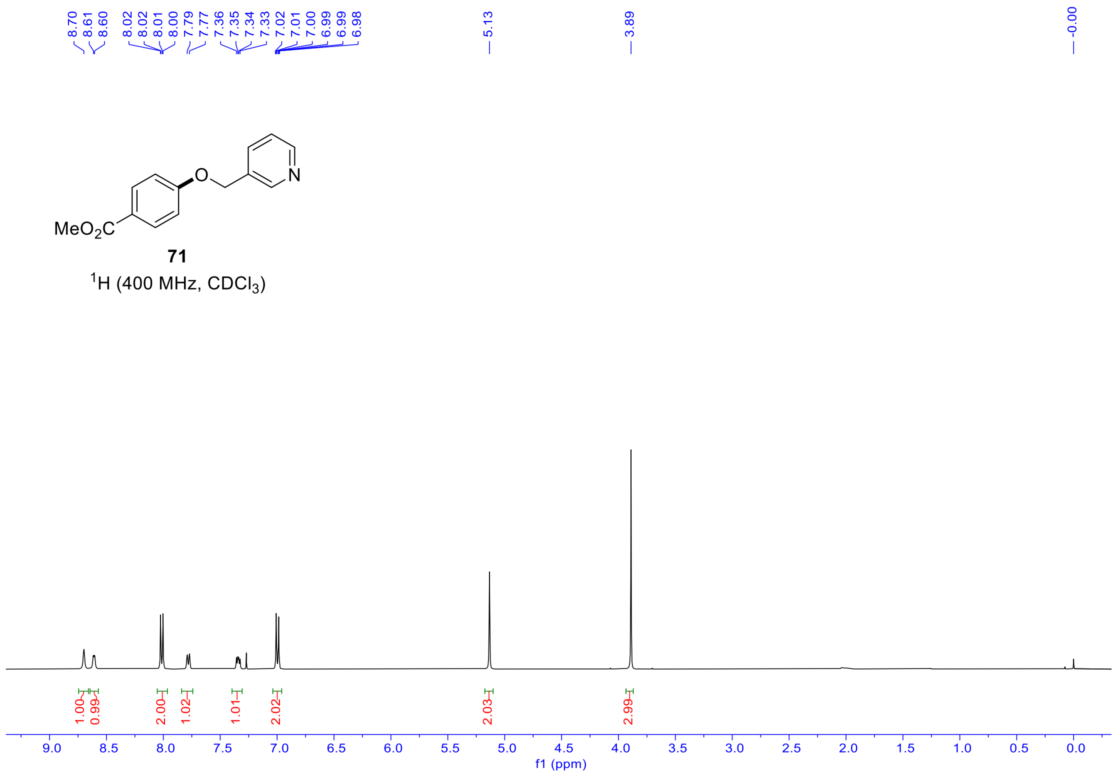

Supplementary Figure 195. ^{1}**H NMR** of compound **71**

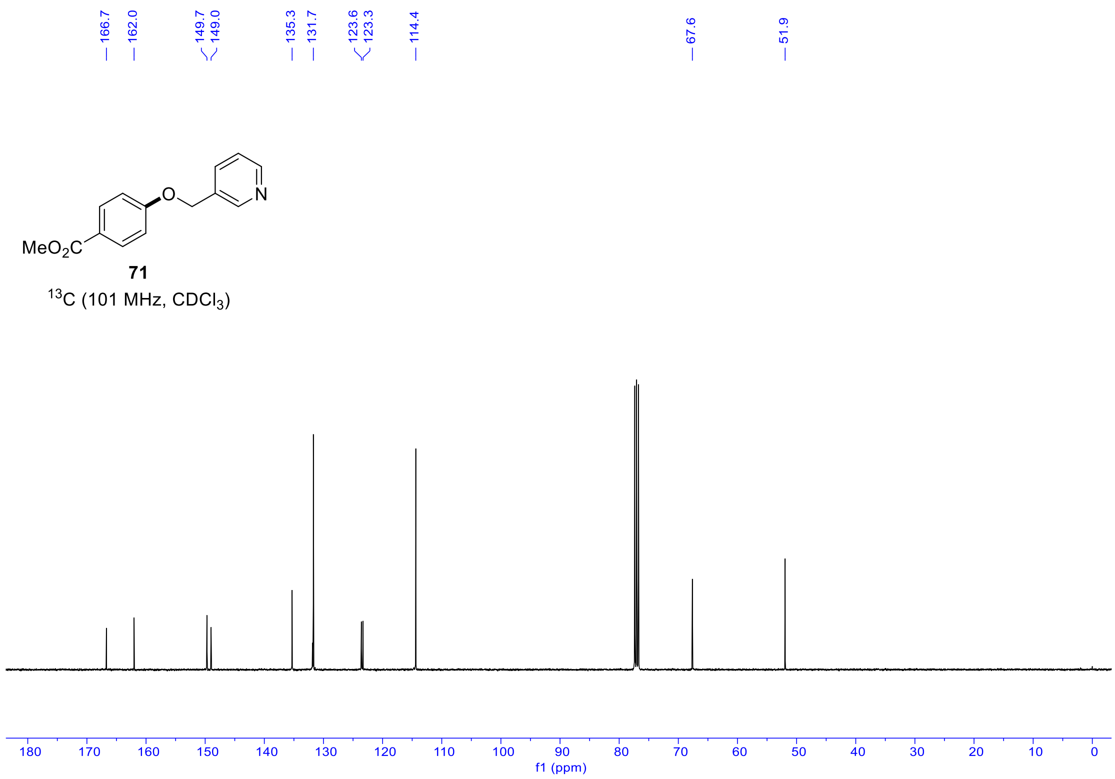

Supplementary Figure 196. ^{13}**C NMR** of compound **71**

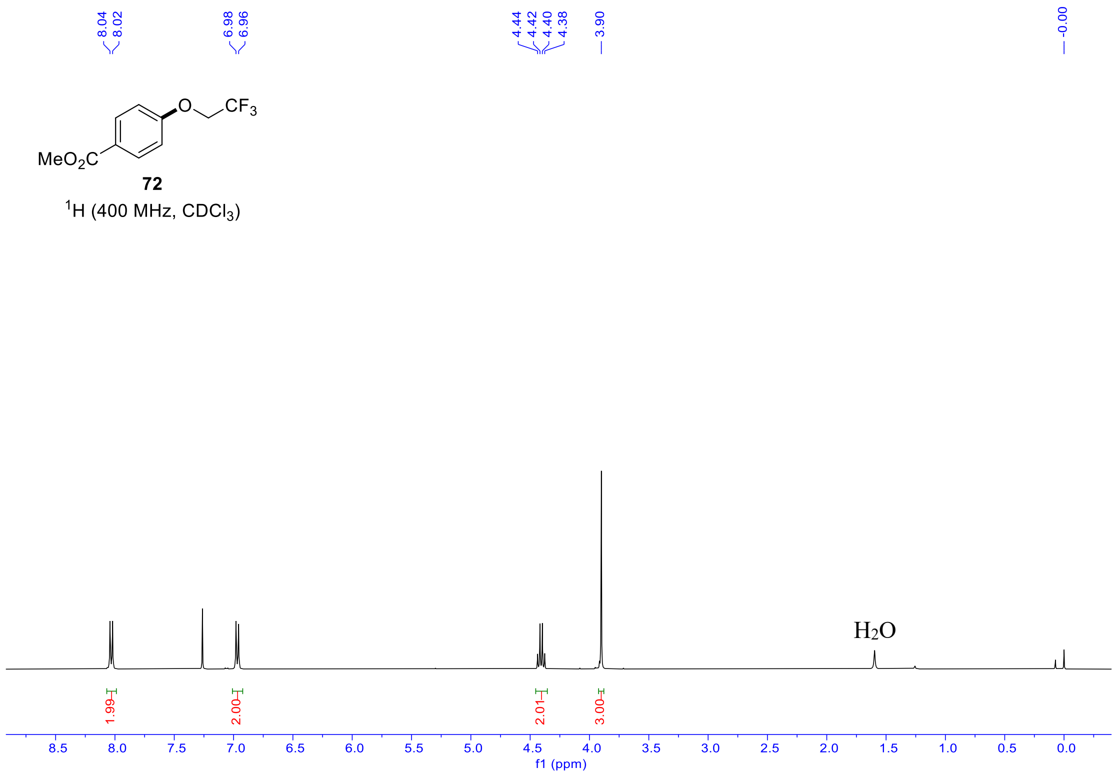

Supplementary Figure 197. ^{1}**H NMR** of compound **72**

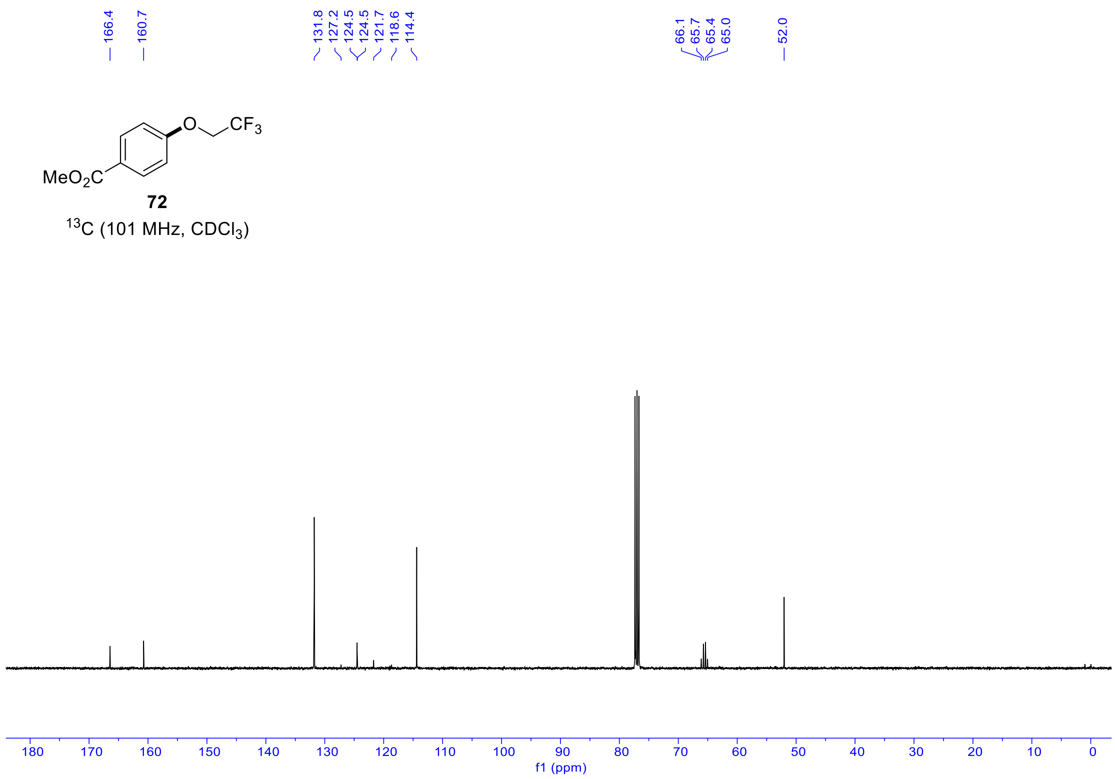

Supplementary Figure 198. ^{13}**C NMR** of compound **72**

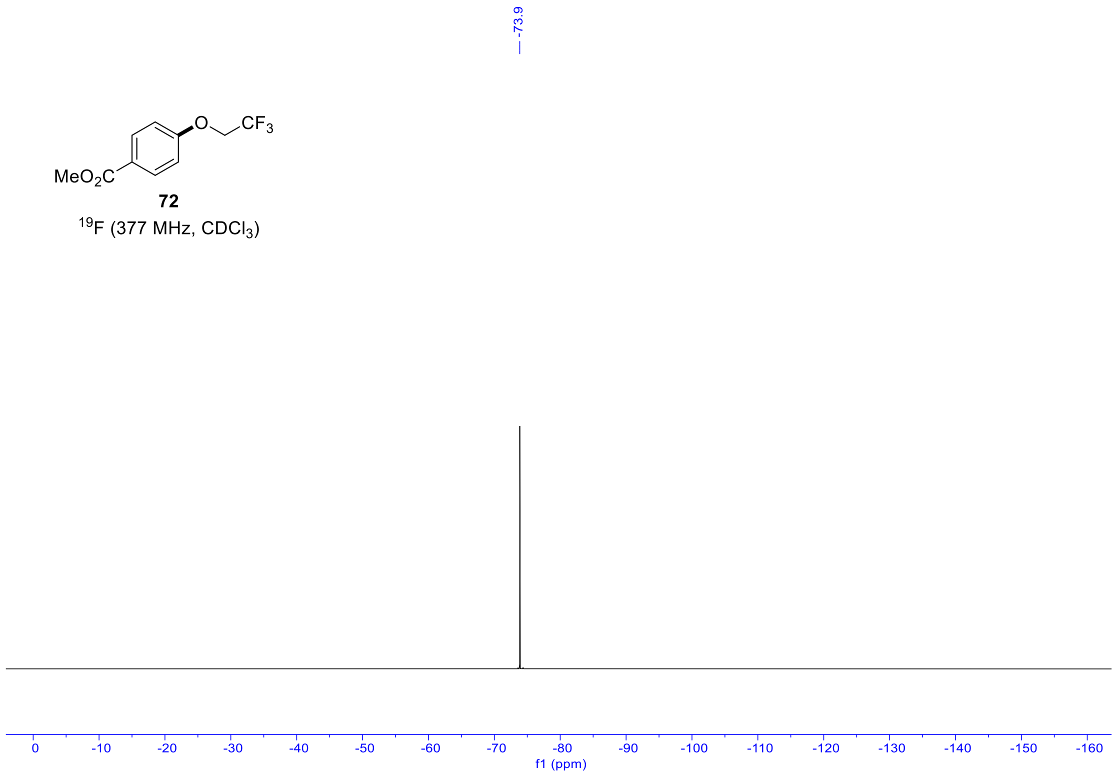

Supplementary Figure 199. ^{19}**F NMR** of compound **72**

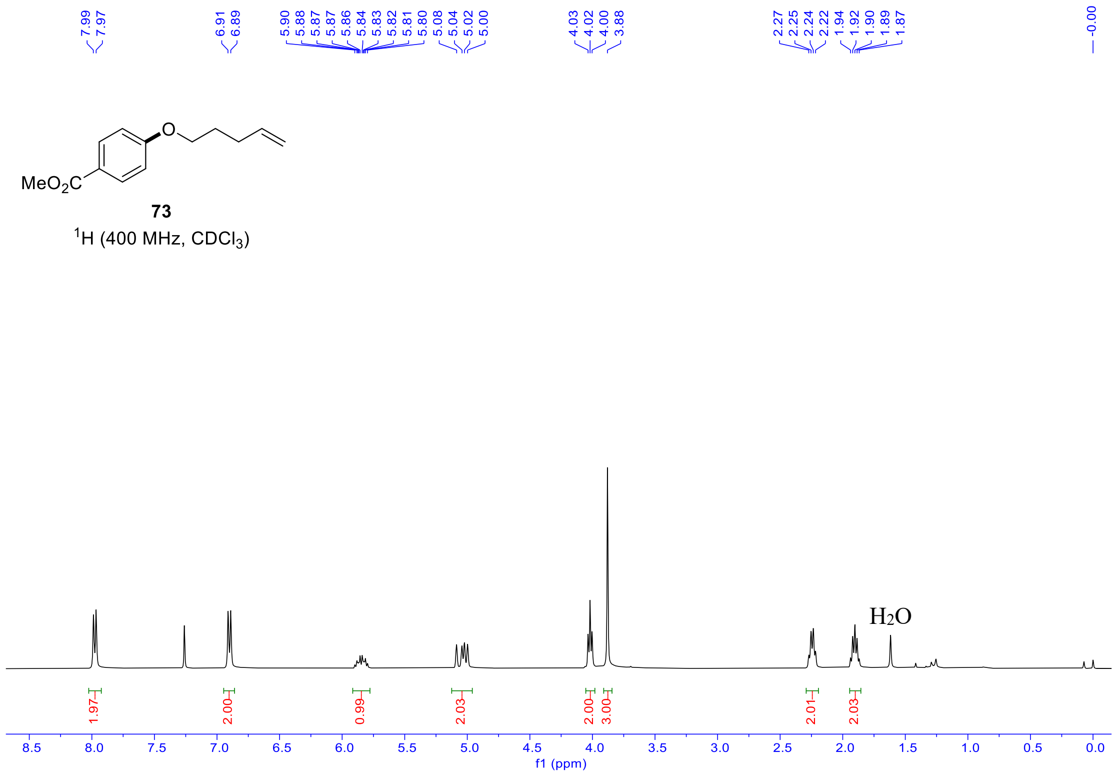

Supplementary Figure 200. ^{1}**H NMR** of compound **73**

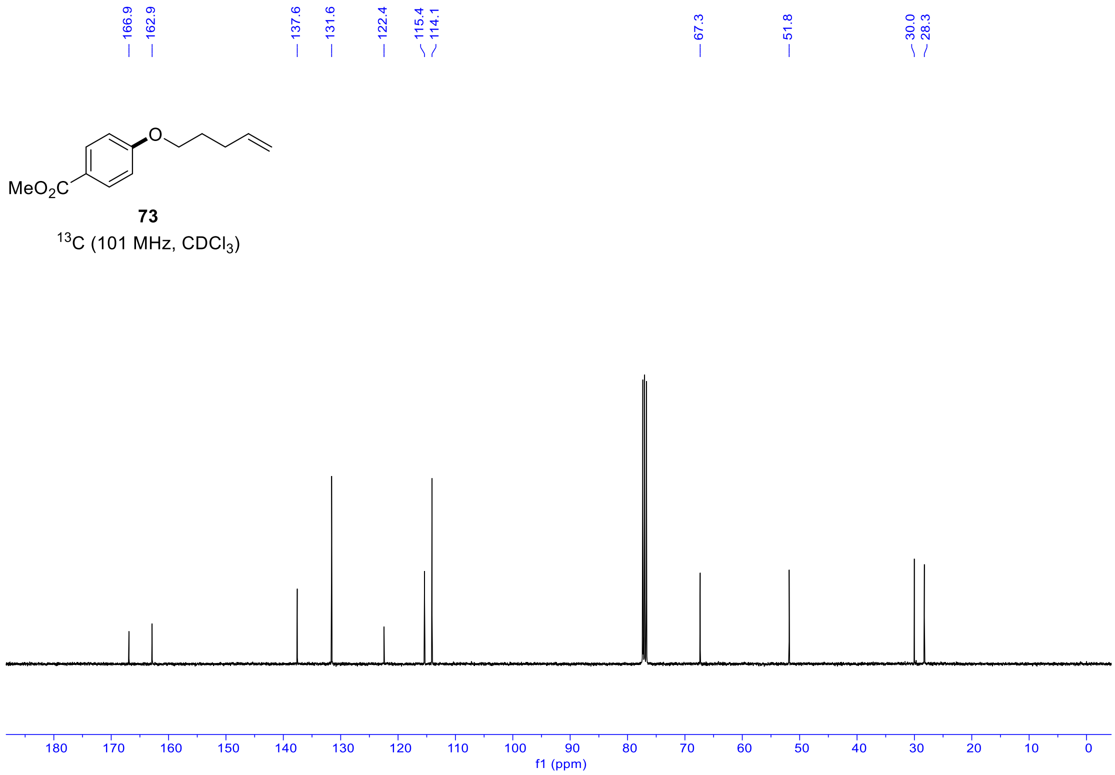

Supplementary Figure 201. ^{13}**C NMR** of compound **73**

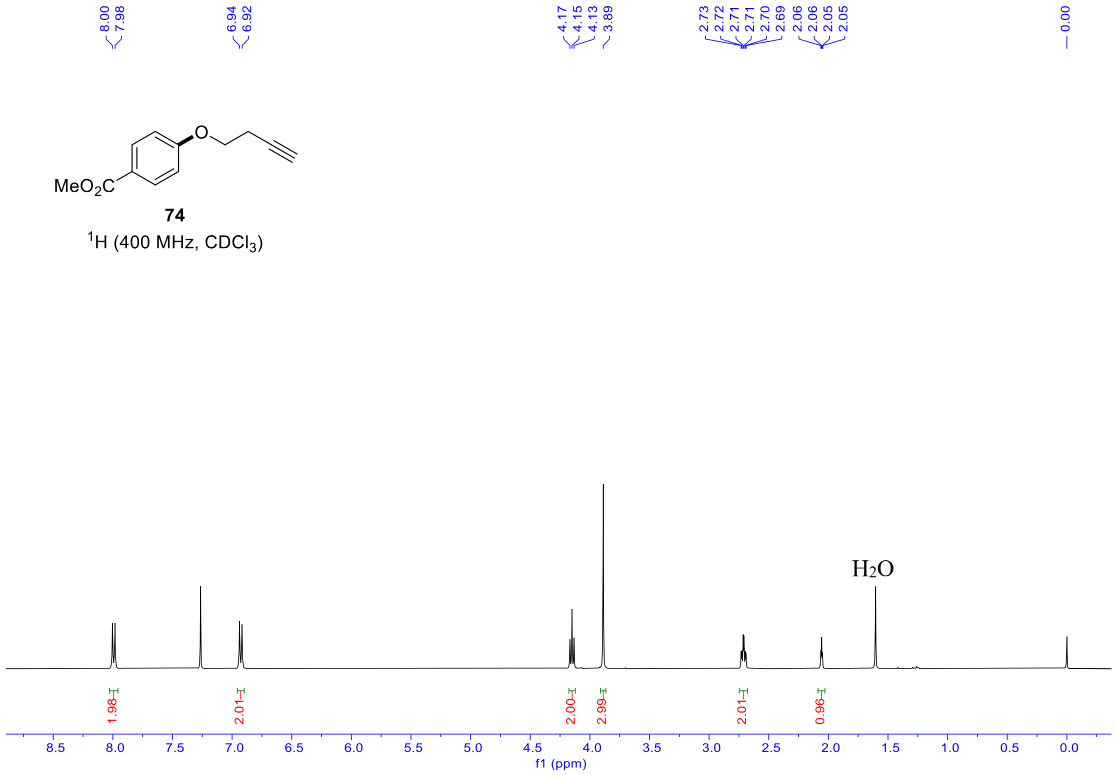

Supplementary Figure 202. ^{1}**H NMR** of compound **74**

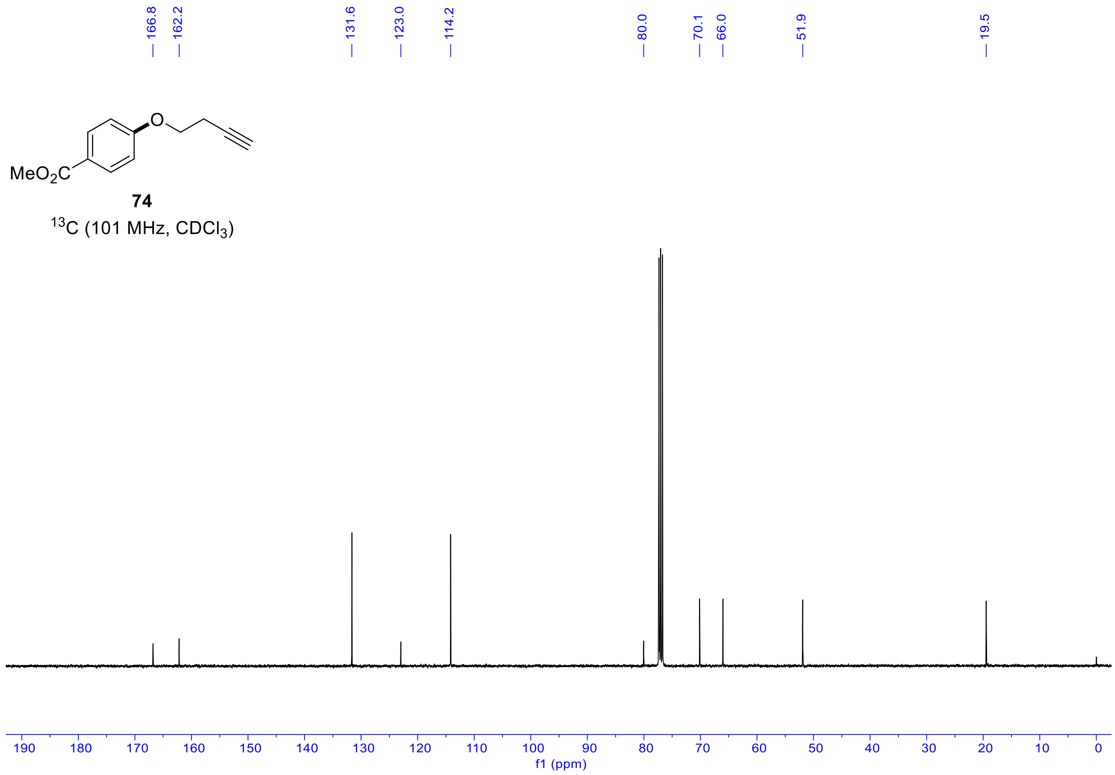

Supplementary Figure 203. ^{13}**C NMR** of compound **74**

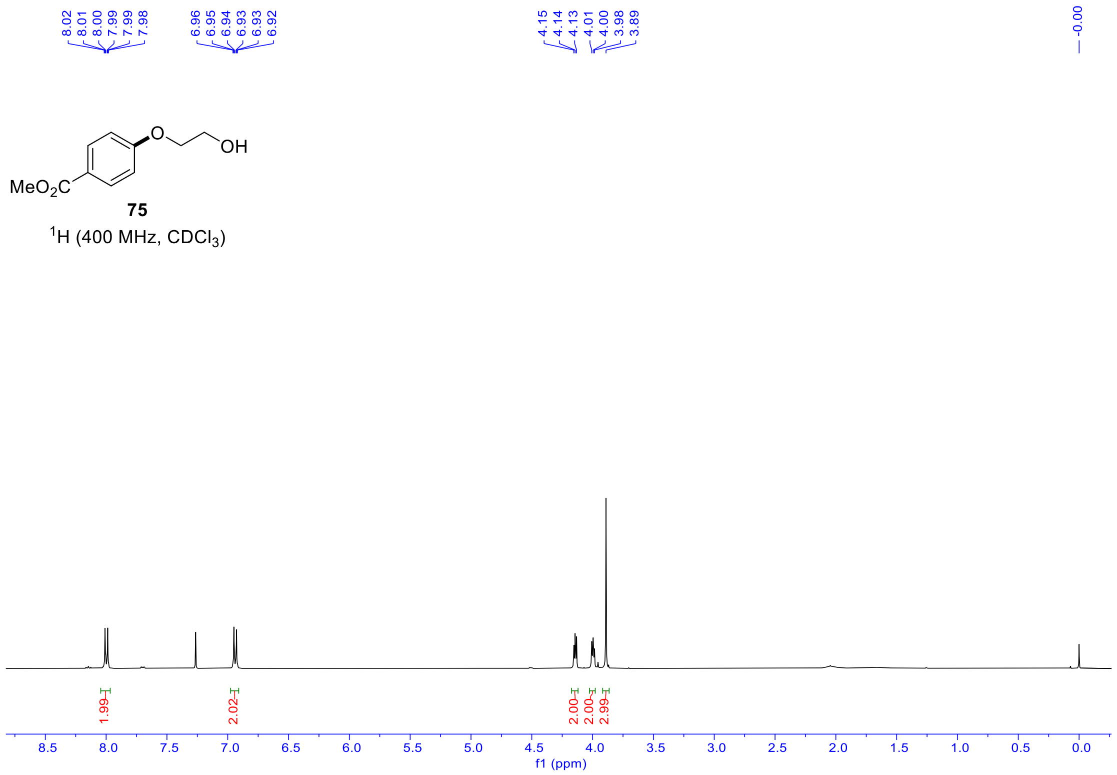

Supplementary Figure 204. ^{1}**H NMR** of compound **75**

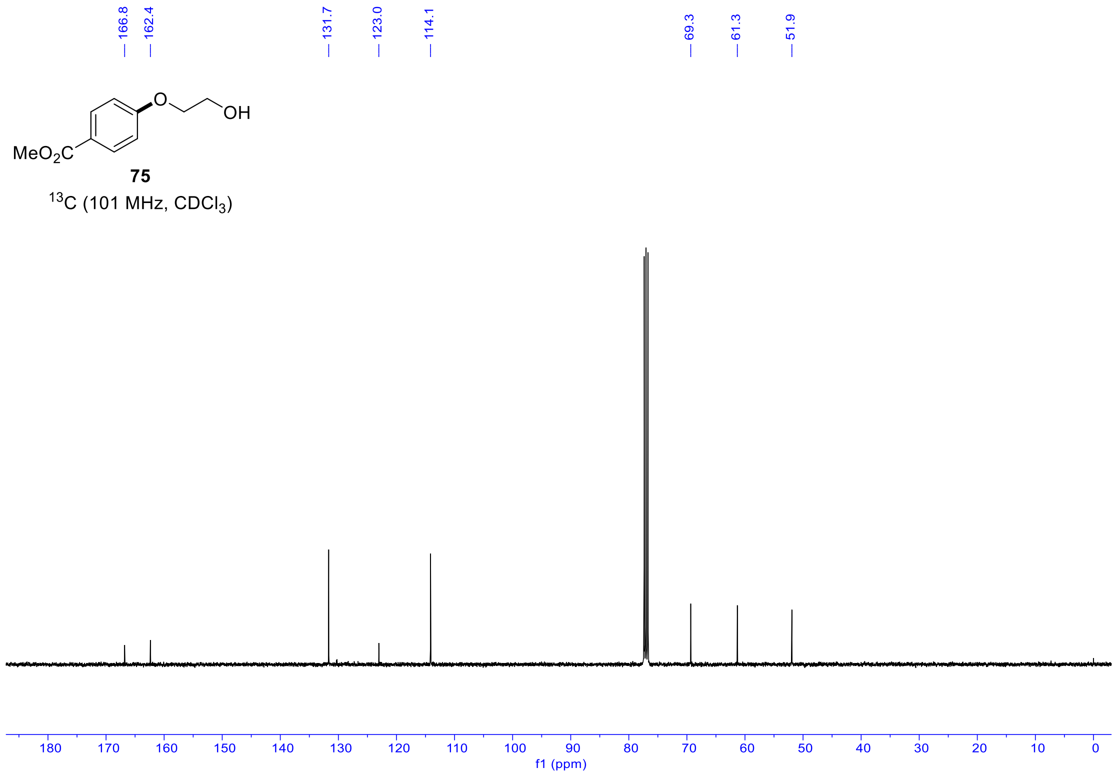

Supplementary Figure 205. ^{13}**C NMR** of compound **75**

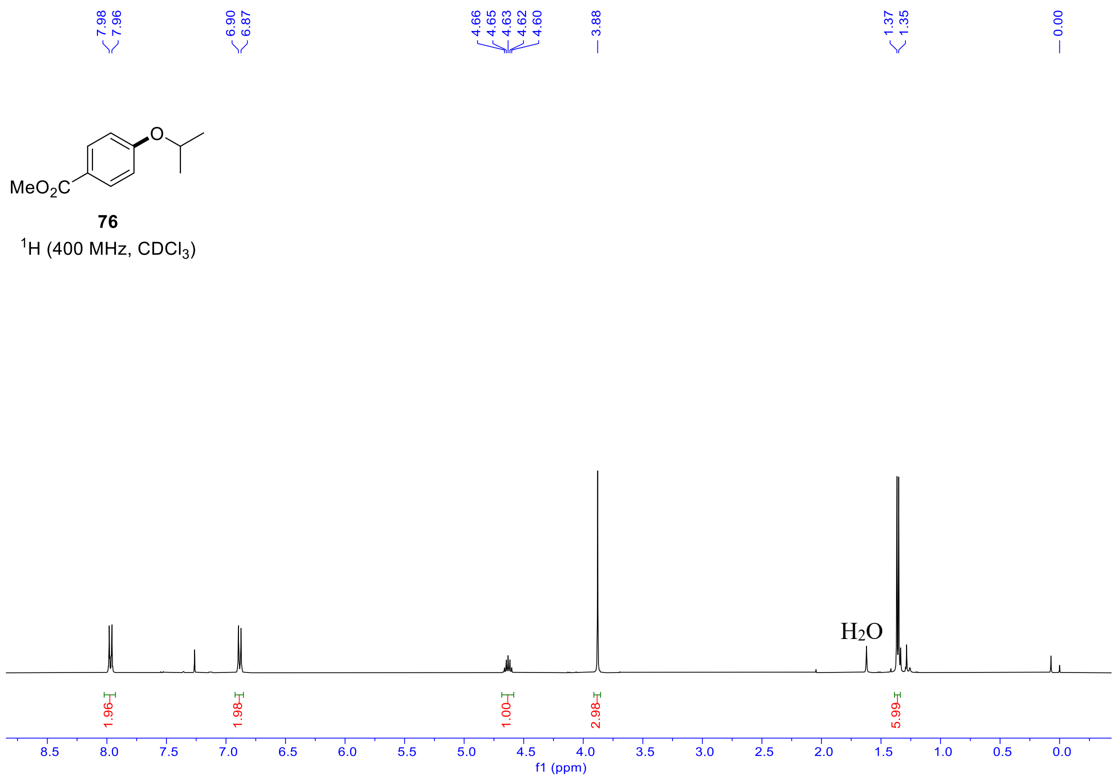

Supplementary Figure 206. ^{1}**H NMR** of compound **76**

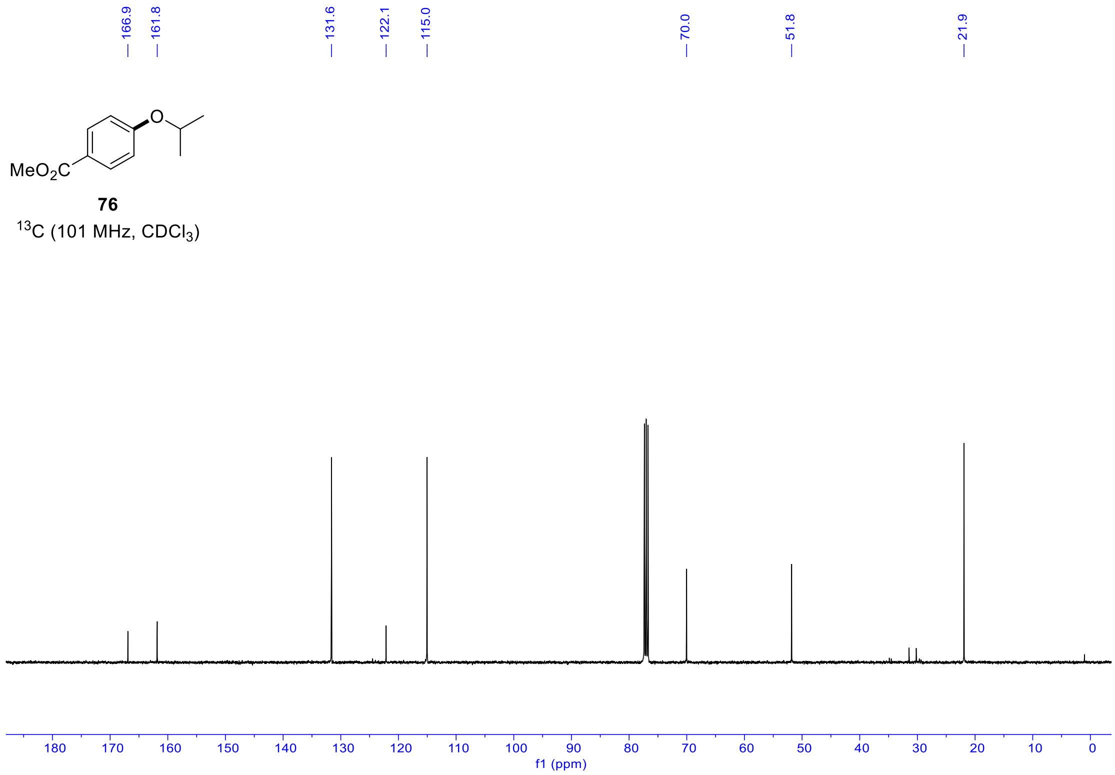

Supplementary Figure 207. ^{13}**C NMR** of compound **76**

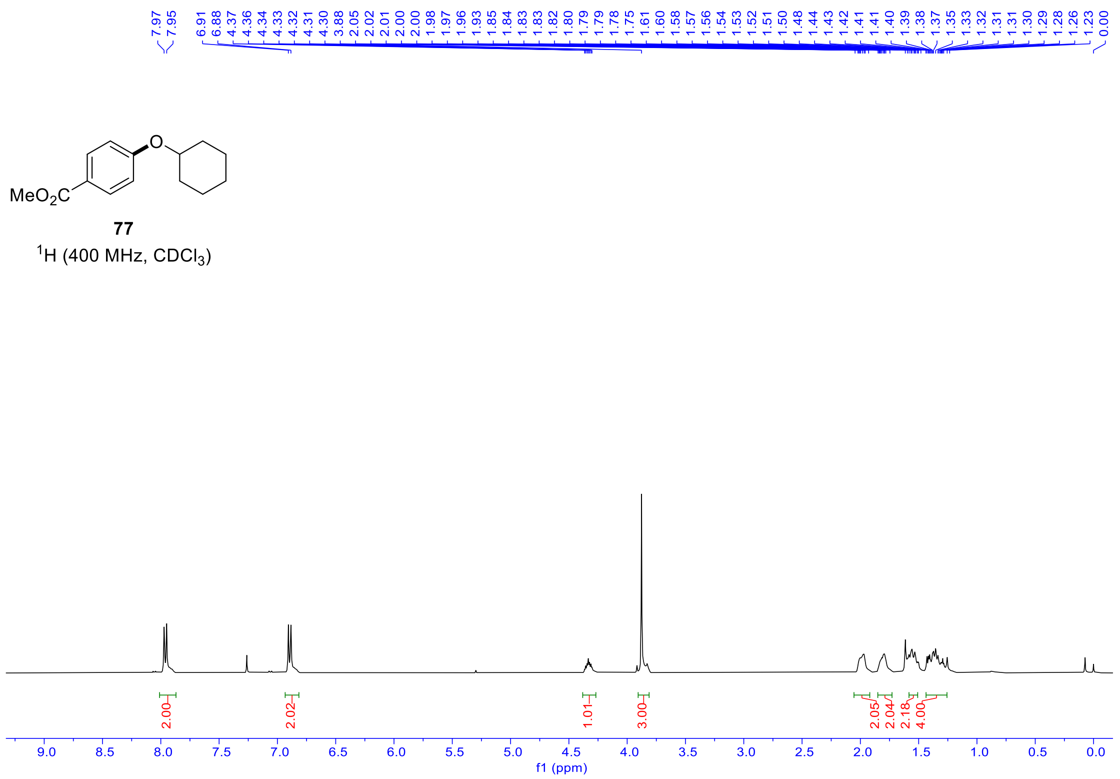

Supplementary Figure 208. ^{1}H NMR of compound **77**

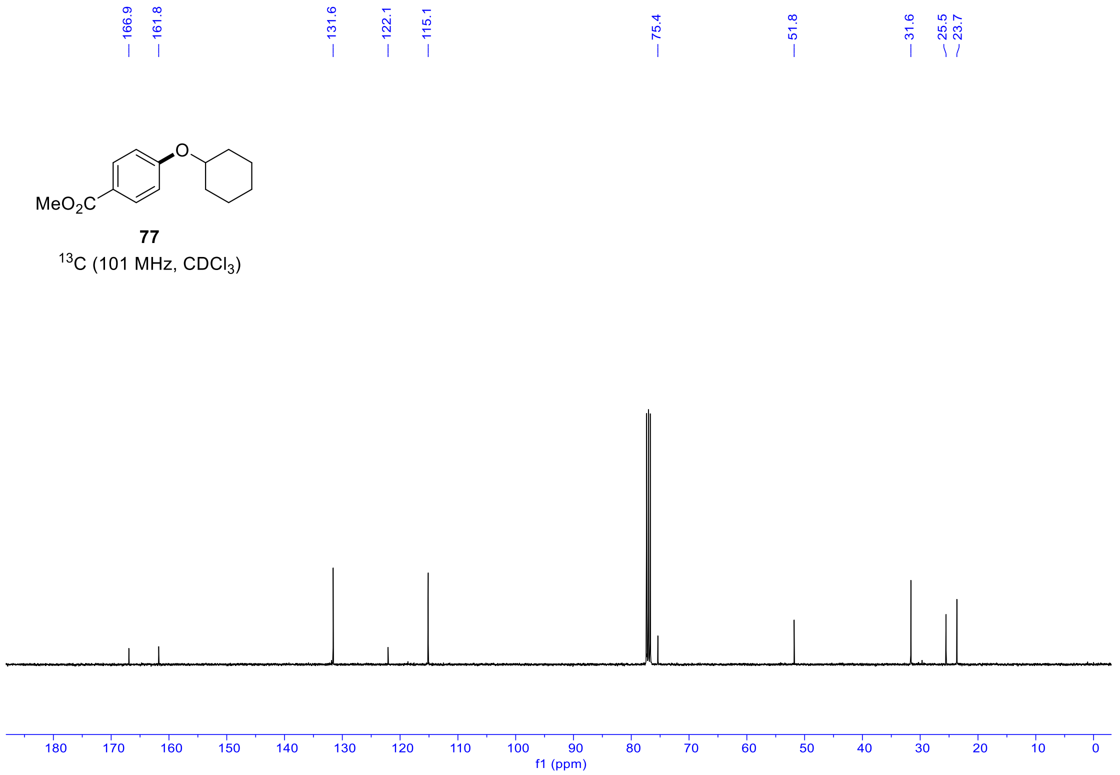

Supplementary Figure 209. ^{13}C NMR of compound **77**

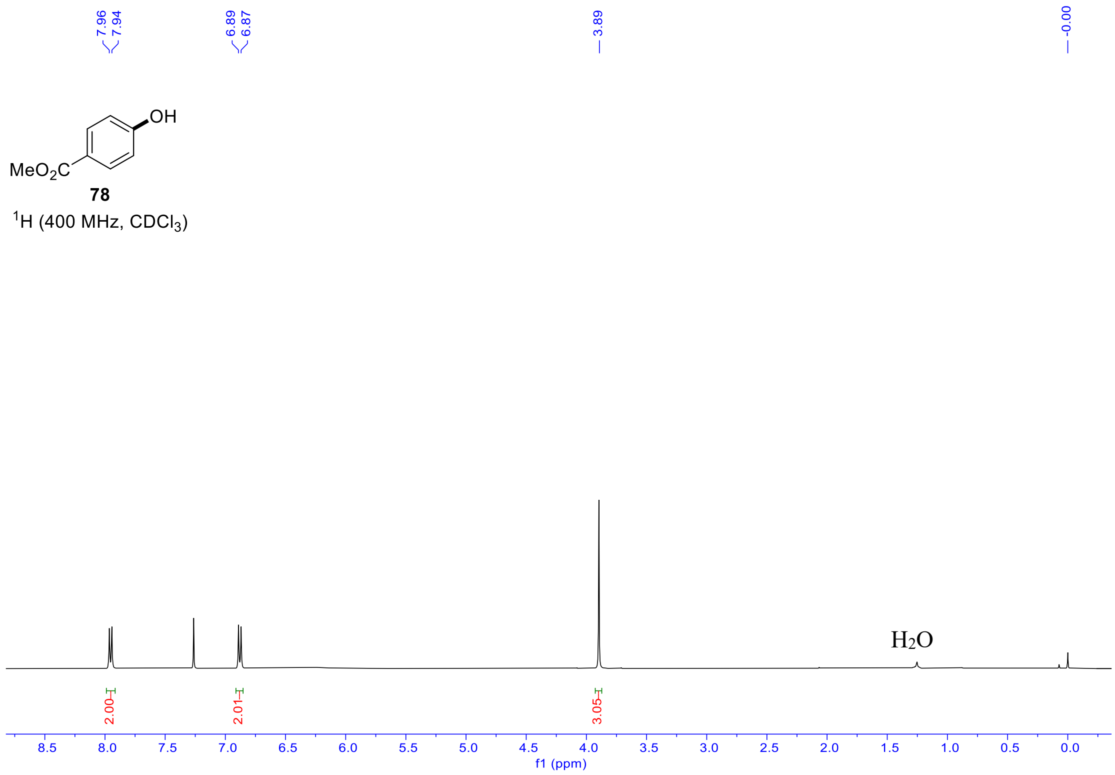

Supplementary Figure 210. ^{1}**H NMR** of compound **78**

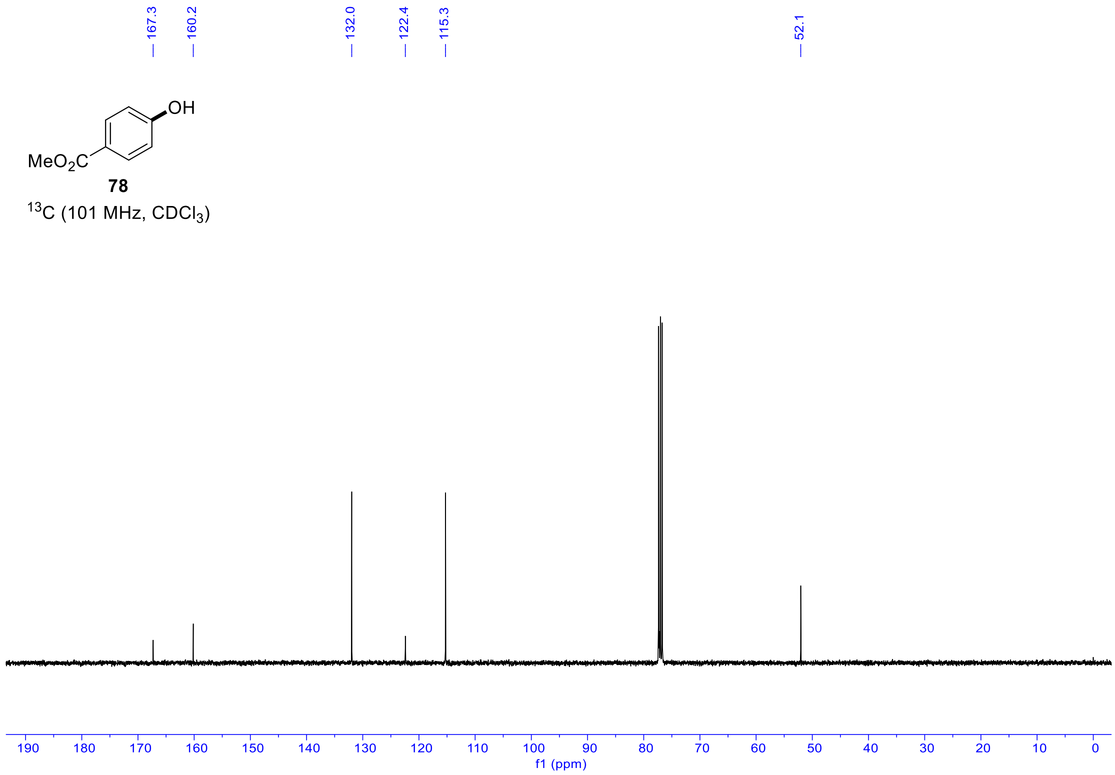

Supplementary Figure 211. ^{13}**C NMR** of compound **78**

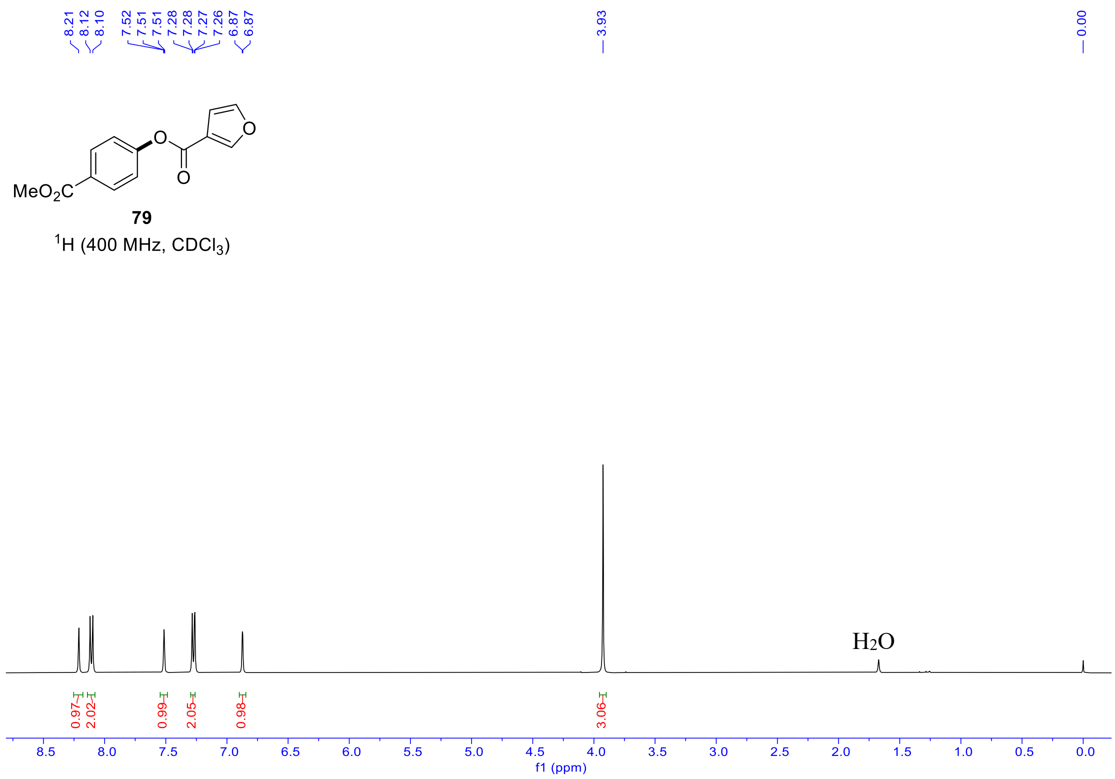

Supplementary Figure 212. ^{1}H NMR of compound **79**

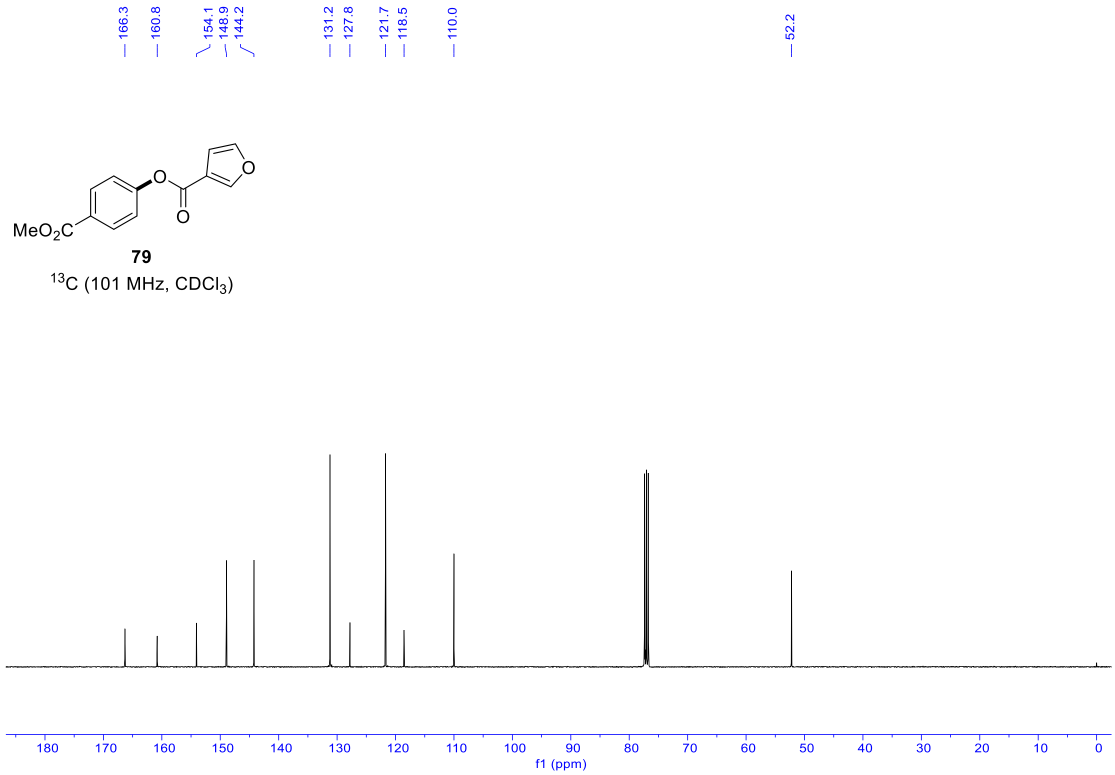

Supplementary Figure 213. ^{13}C NMR of compound **79**

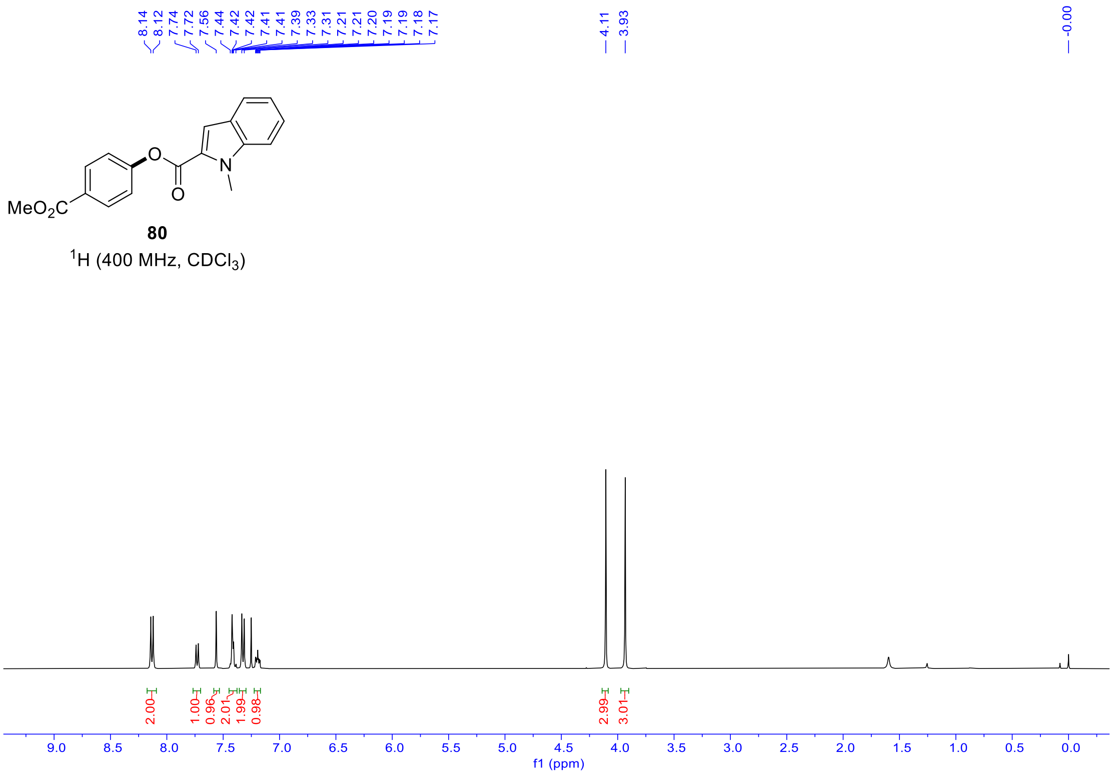

Supplementary Figure 214. ^{1}**H NMR** of compound **80**

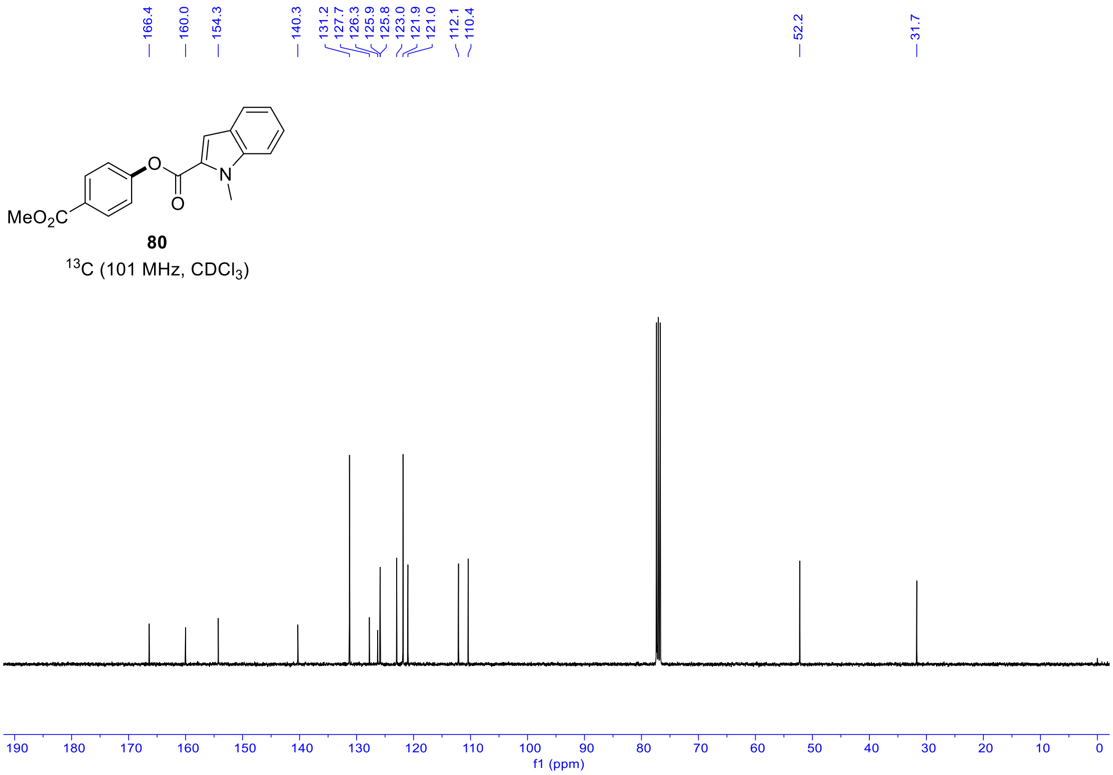

Supplementary Figure 215. ^{13}**C NMR** of compound **80**

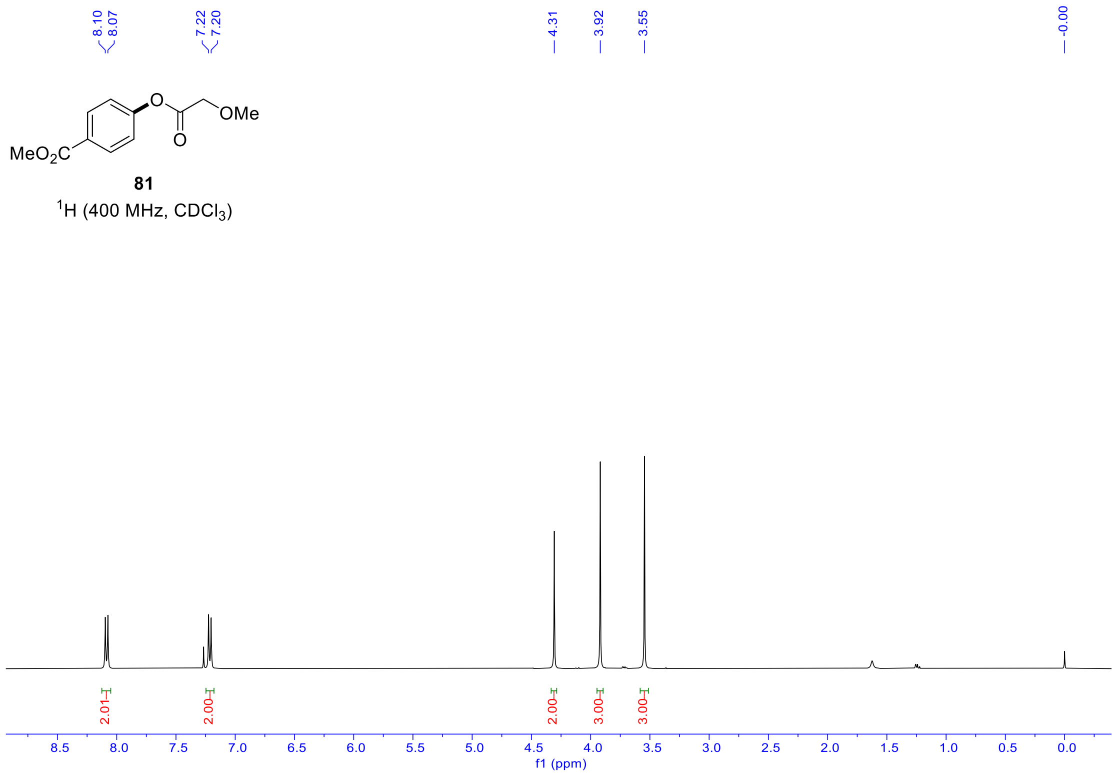

Supplementary Figure 216. ^{1}**H NMR** of compound **81**

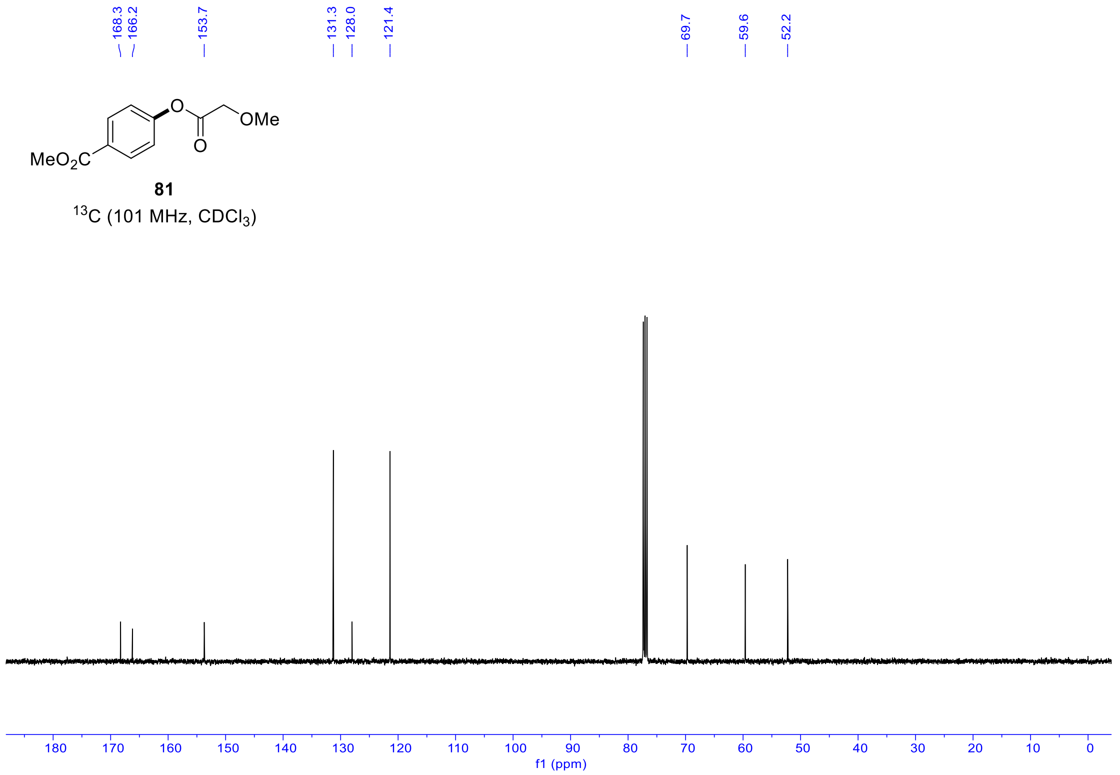

Supplementary Figure 217. ^{13}**C NMR** of compound **81**

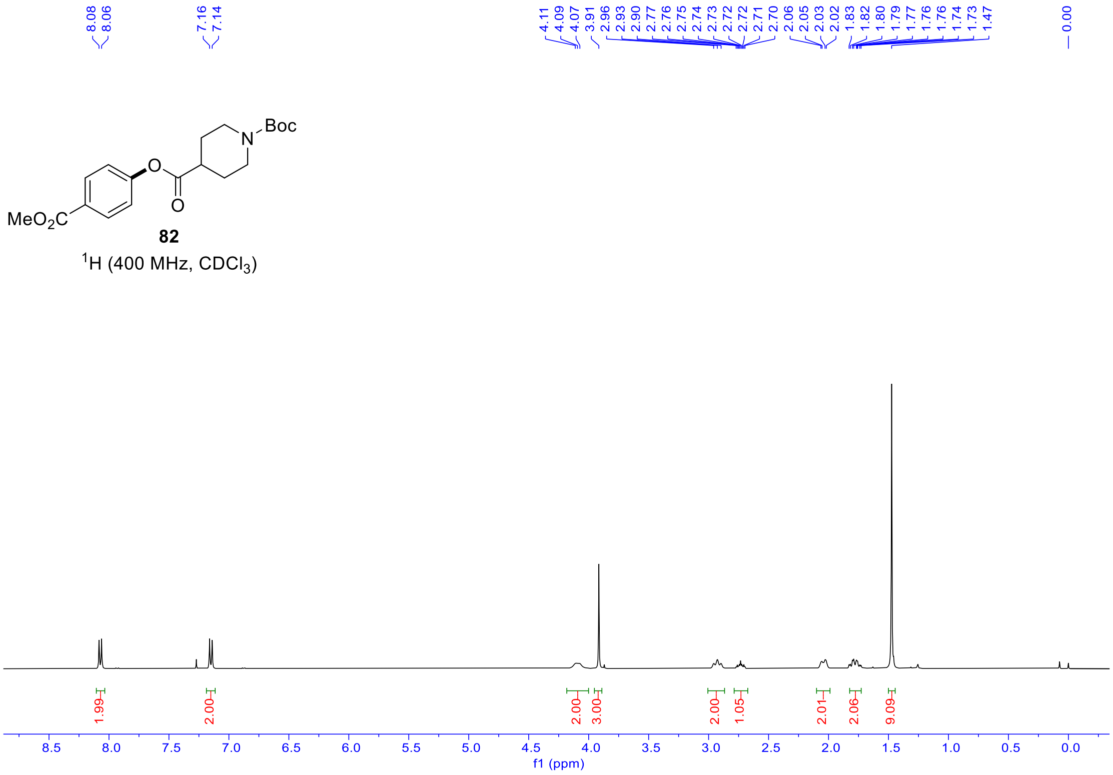

Supplementary Figure 218. ^{1}**H NMR** of compound **82**

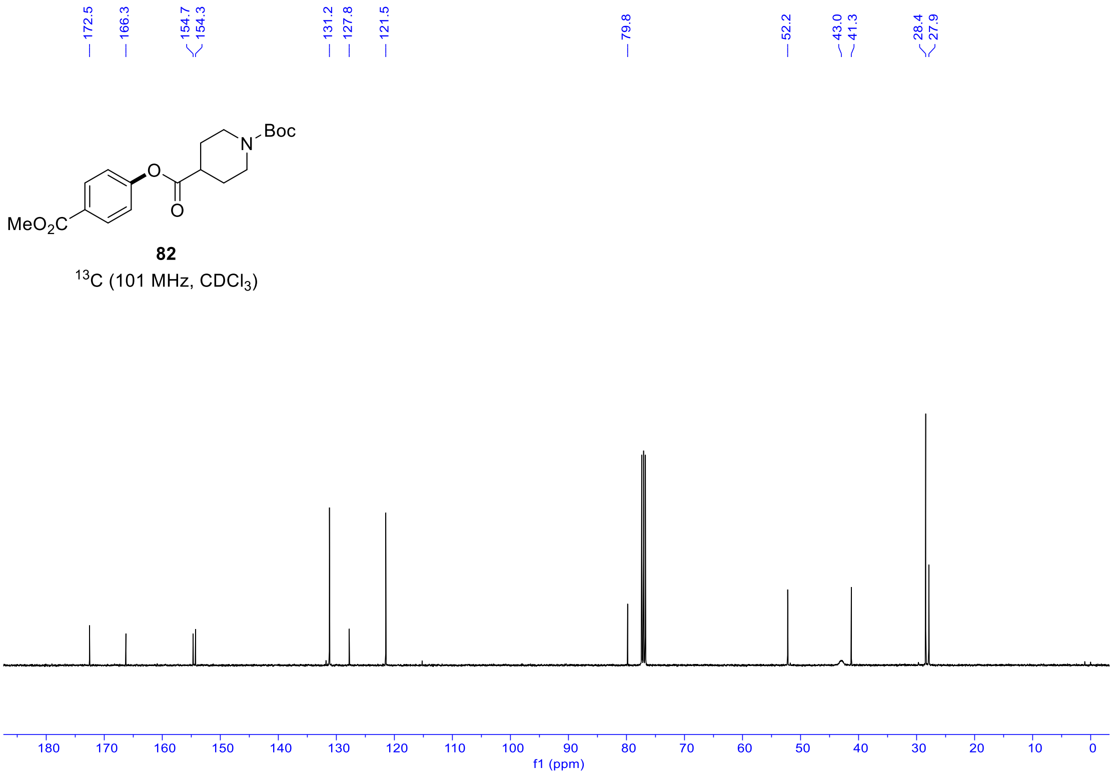

Supplementary Figure 219. ^{13}**C NMR** of compound **82**

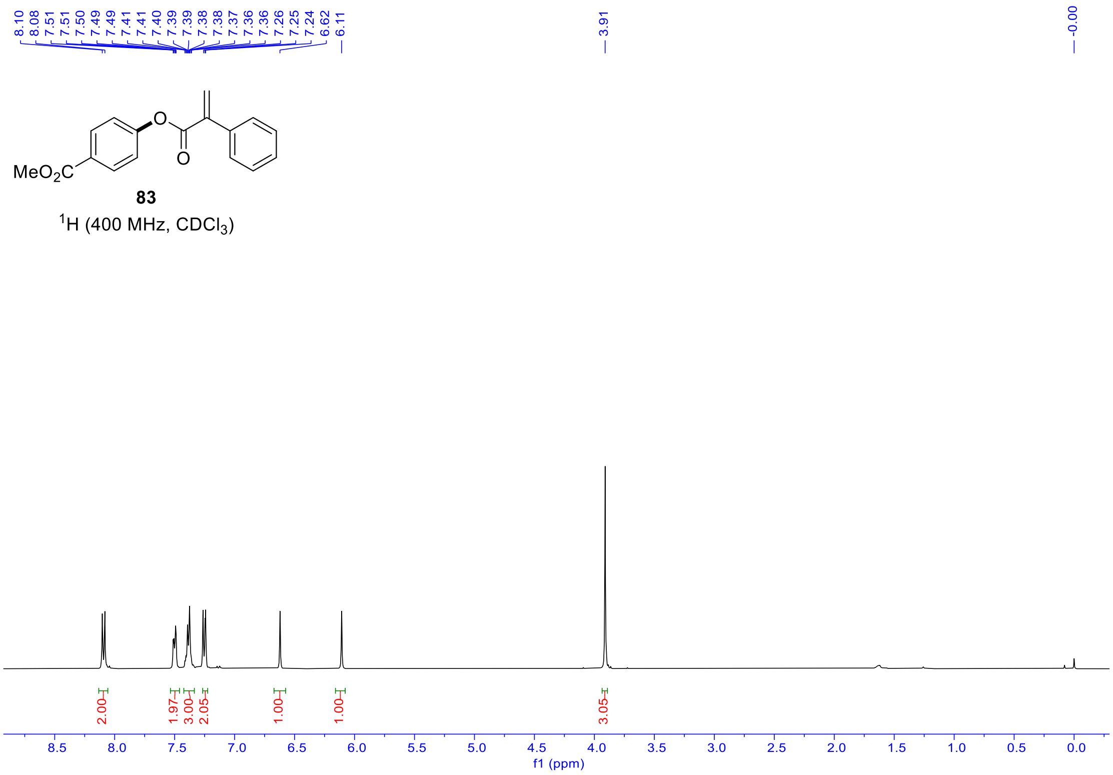

Supplementary Figure 220. ^{1}**H NMR** of compound **83**

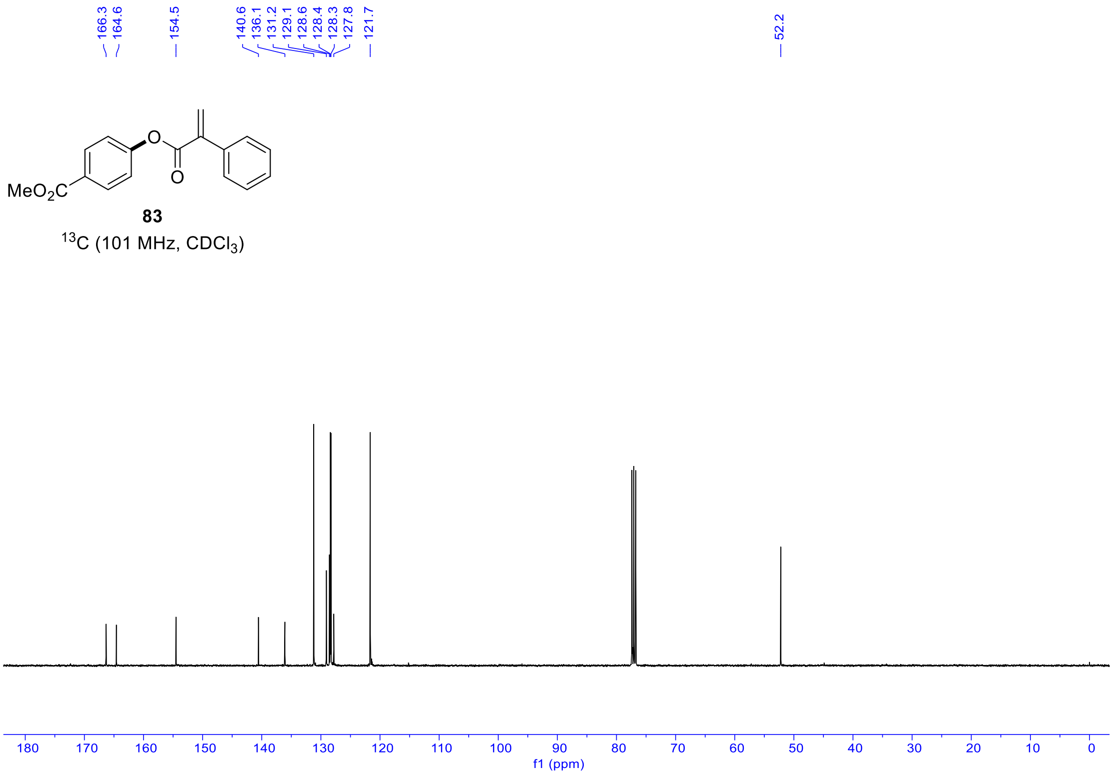

Supplementary Figure 221. ^{13}**C NMR** of compound **83**

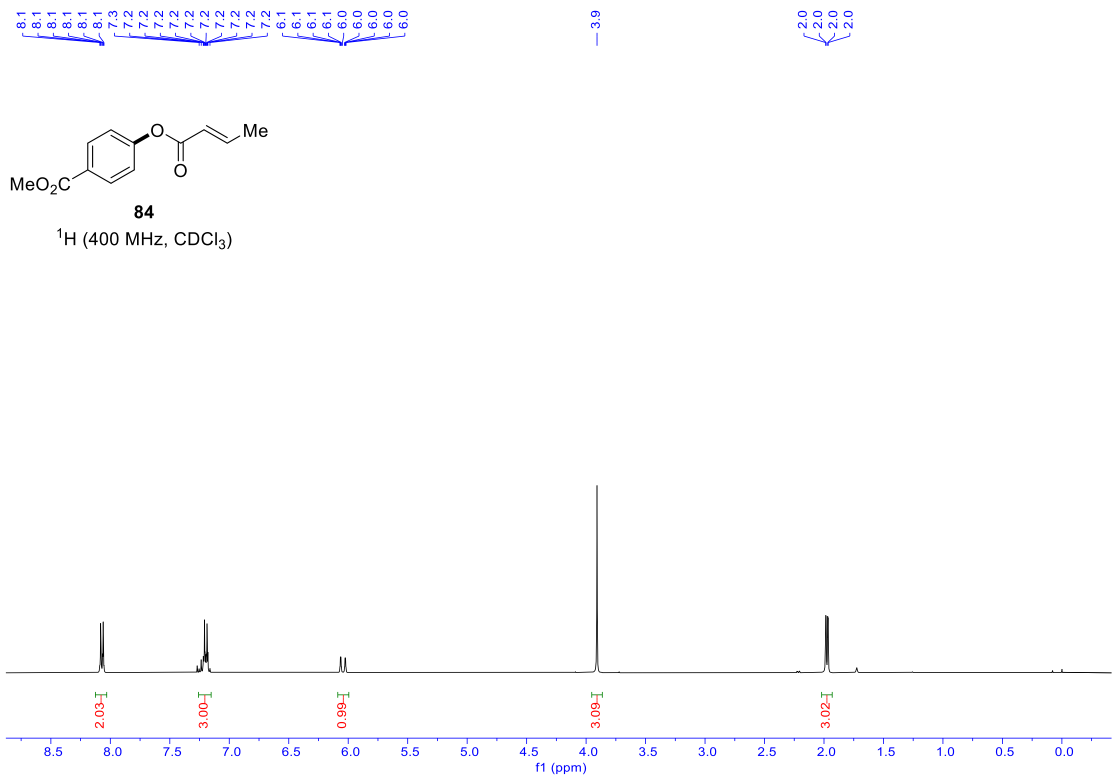

Supplementary Figure 222. ^{1}H NMR of compound **84**

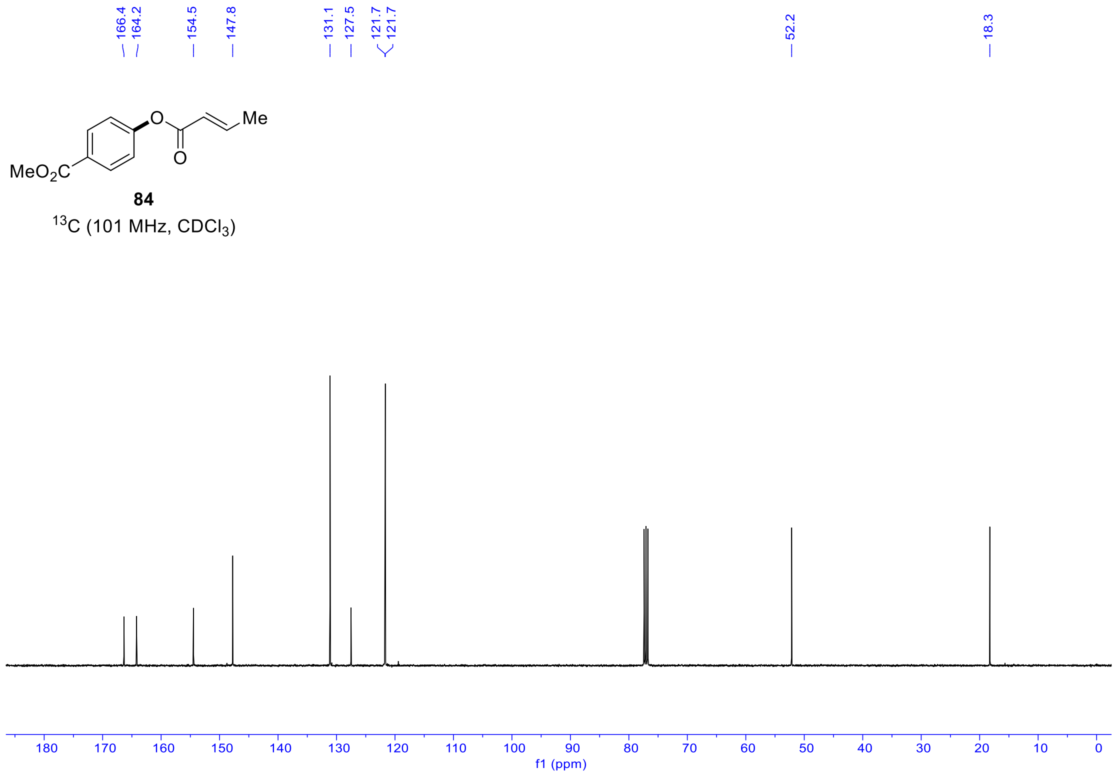

Supplementary Figure 223. ^{13}C NMR of compound **84**

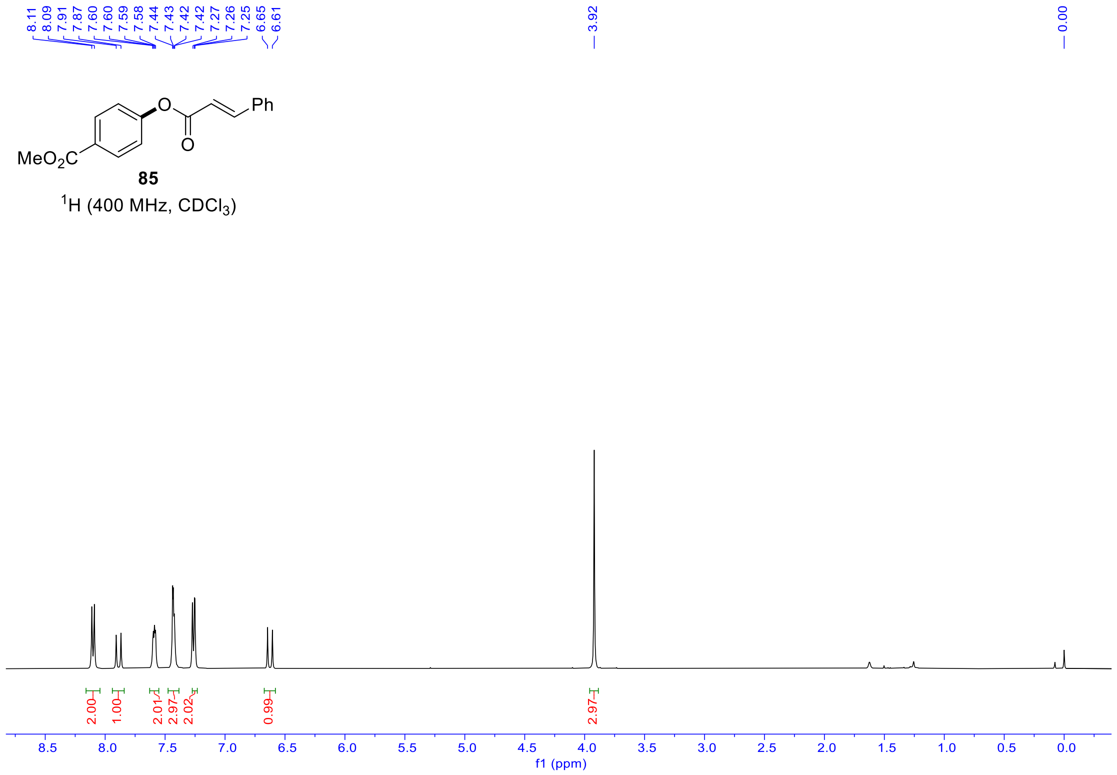

Supplementary Figure 224. ^{1}**H NMR** of compound **85**

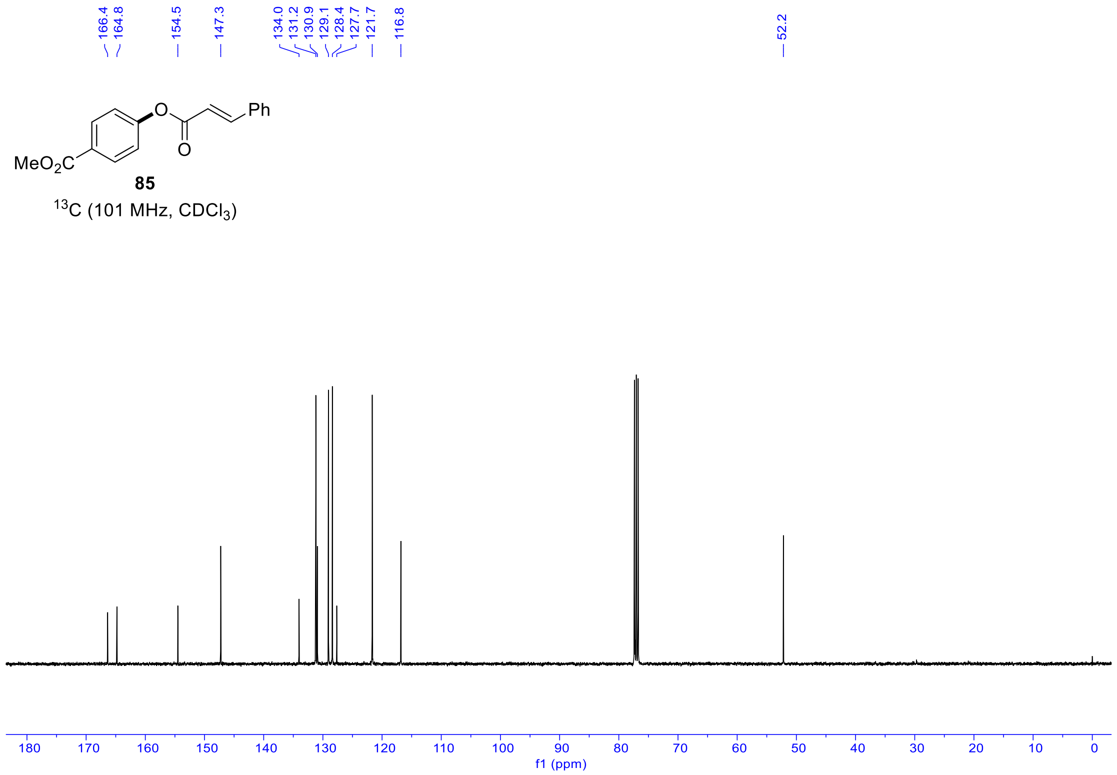

Supplementary Figure 225. ^{13}**C NMR** of compound **85**

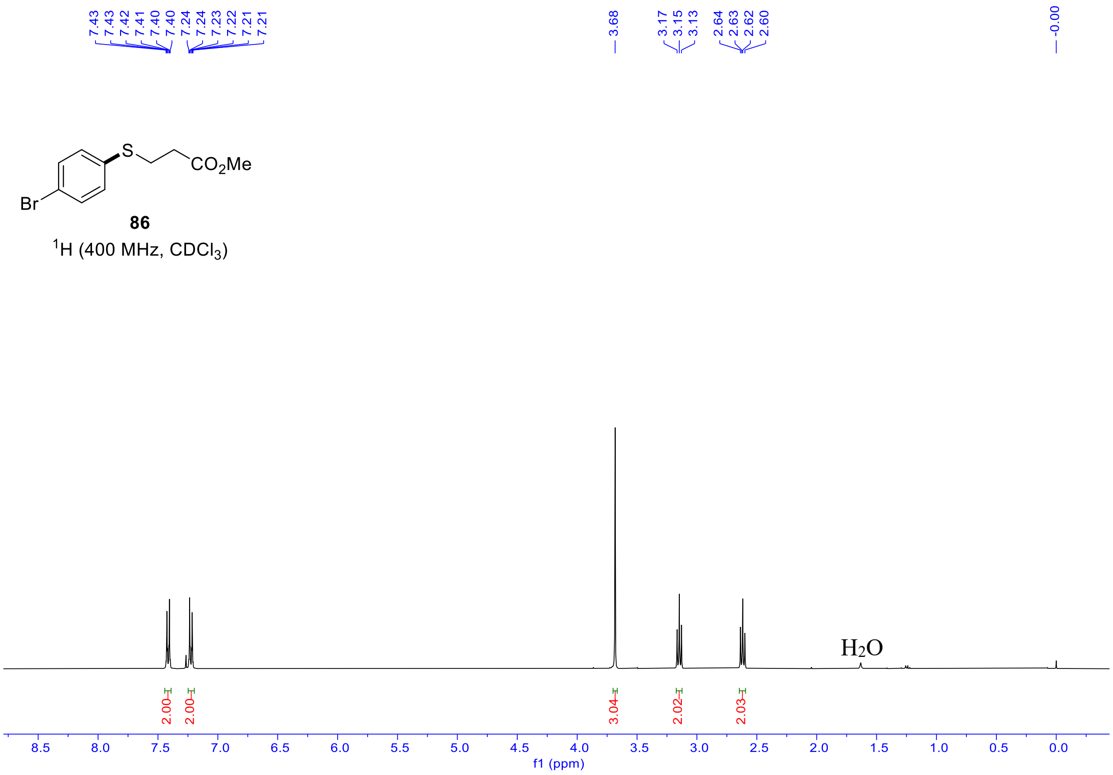

Supplementary Figure 226. ^{1}**H NMR** of compound **86**

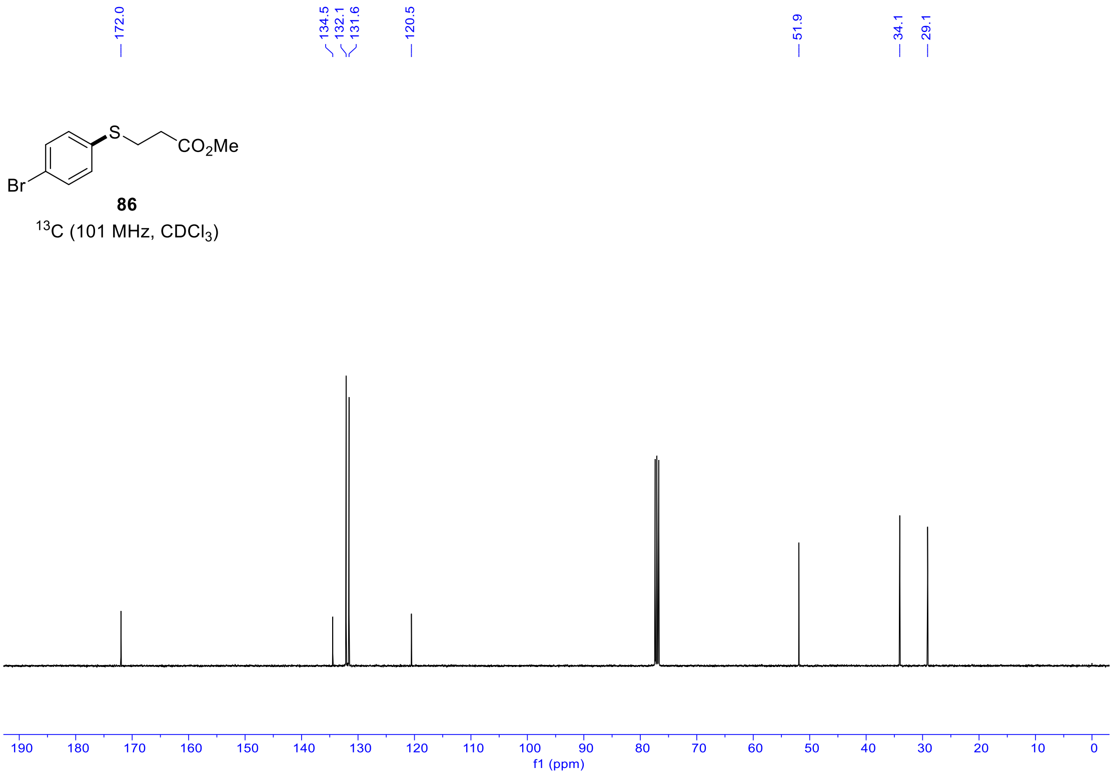

Supplementary Figure 227. ^{13}**C NMR** of compound **86**

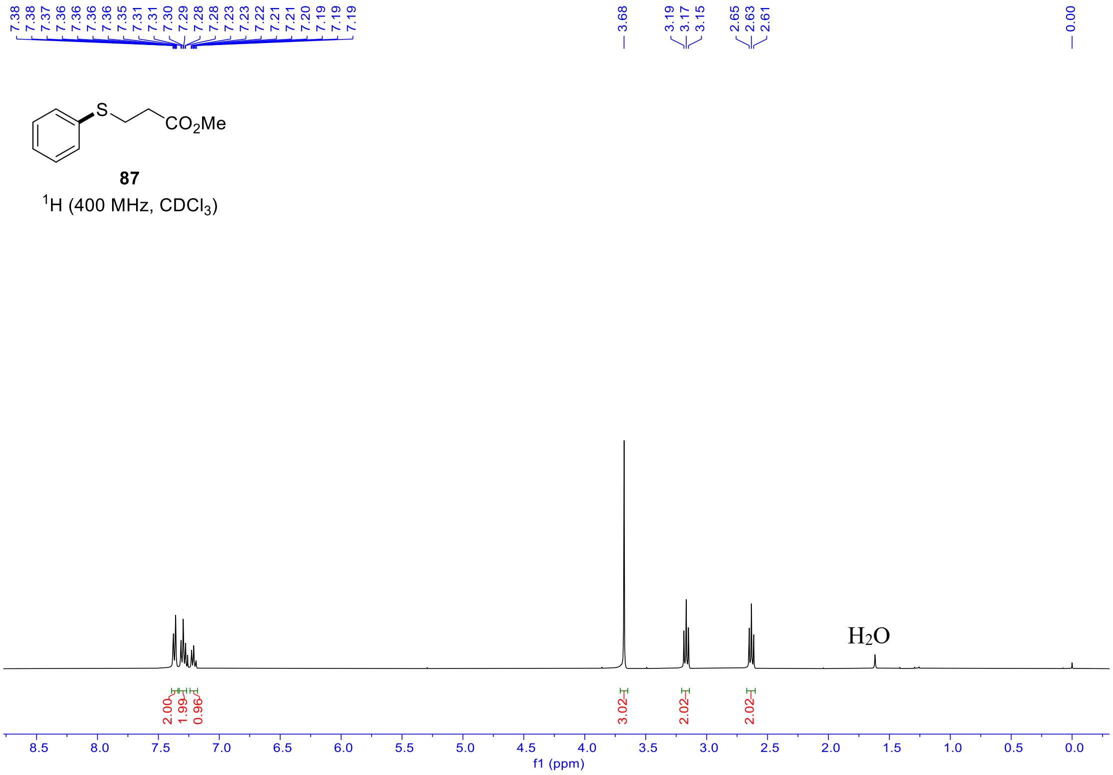

Supplementary Figure 228. ^{1}**H NMR** of compound **87**

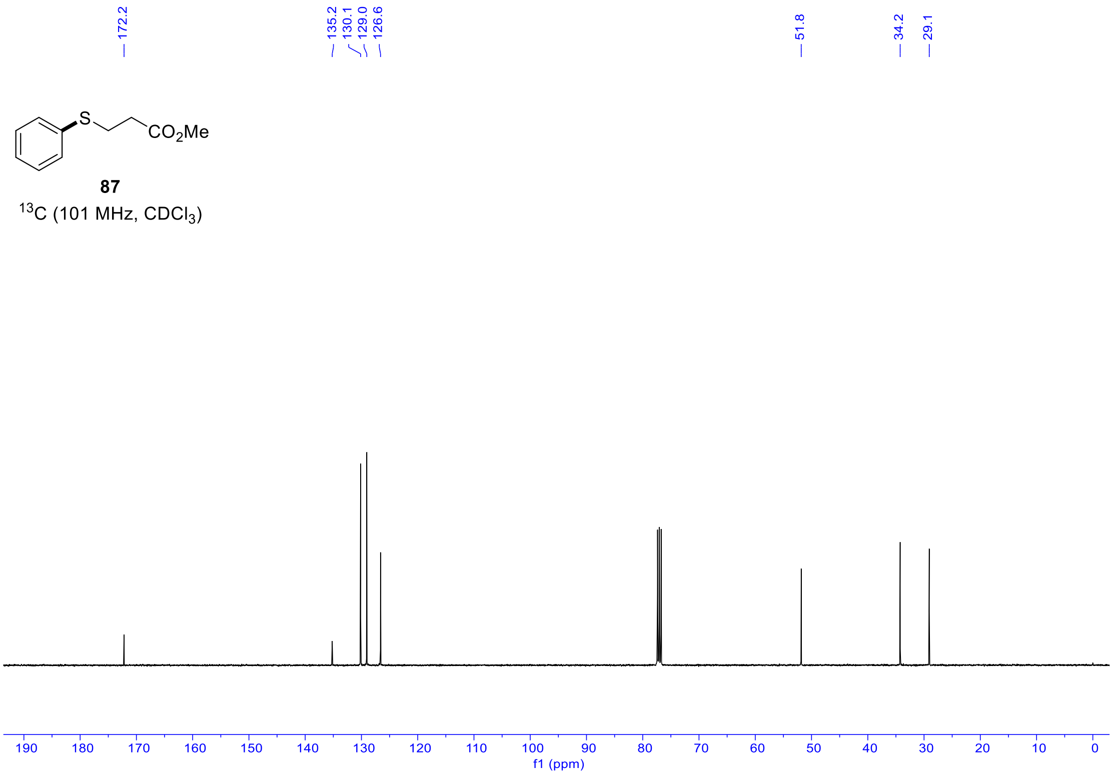

Supplementary Figure 229. ^{13}**C NMR** of compound **87**

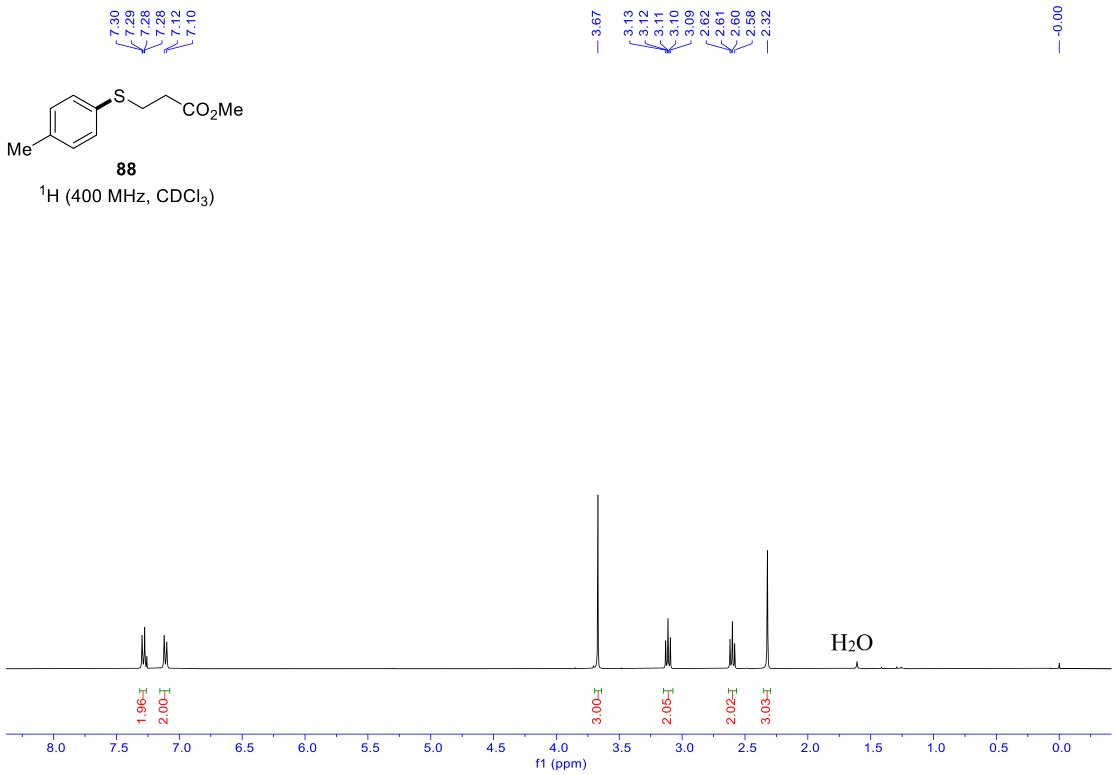

Supplementary Figure 230. ^{1}H NMR of compound **88**

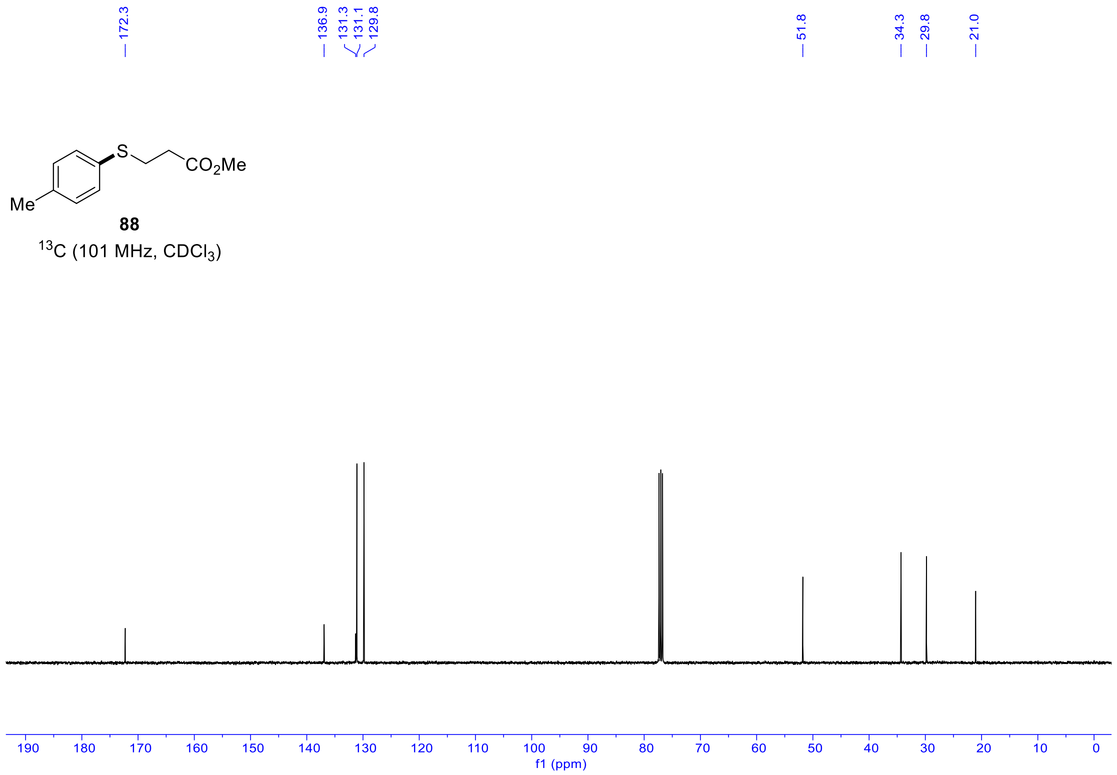

Supplementary Figure 231. ^{13}C NMR of compound **88**

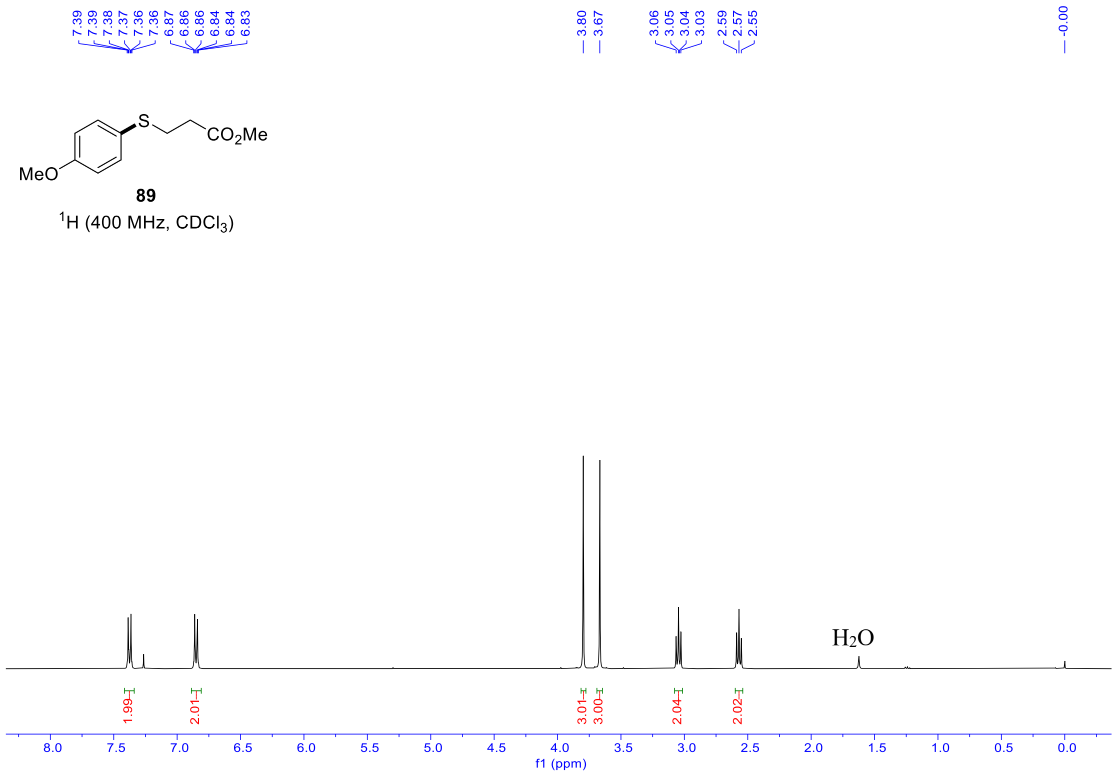

Supplementary Figure 232. ^{1}H NMR of compound **89**

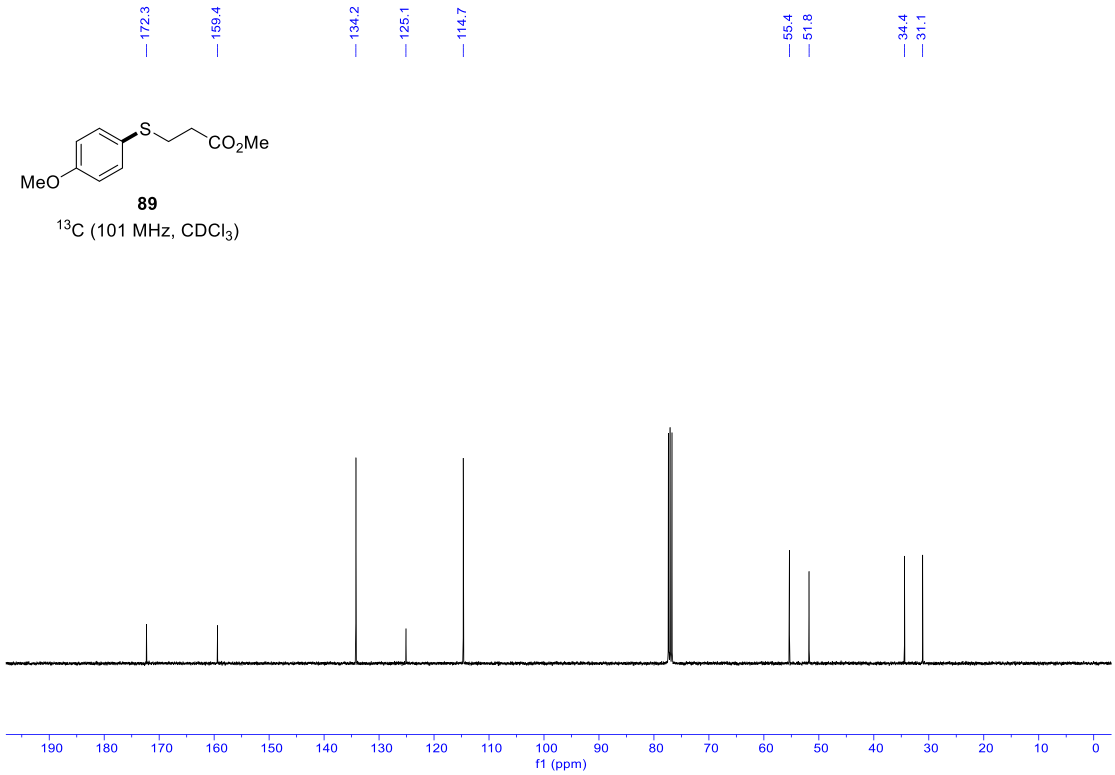

Supplementary Figure 233. ^{13}C NMR of compound **89**

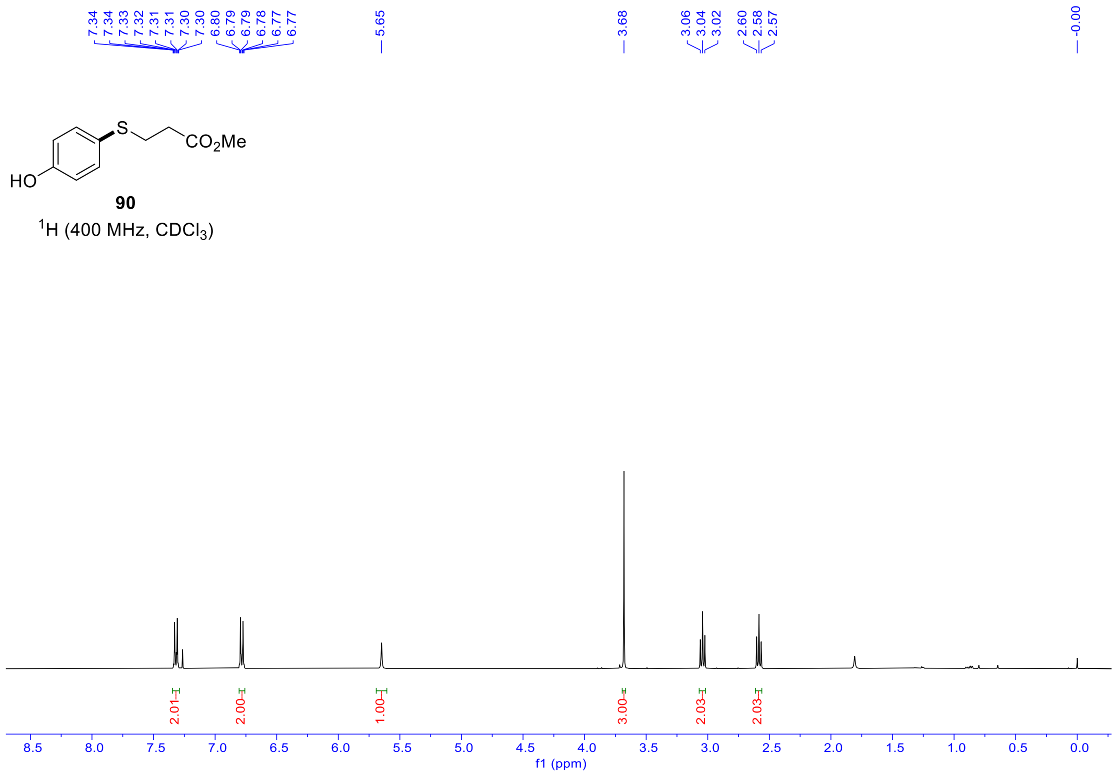

Supplementary Figure 234. ^{1}**H NMR** of compound **90**

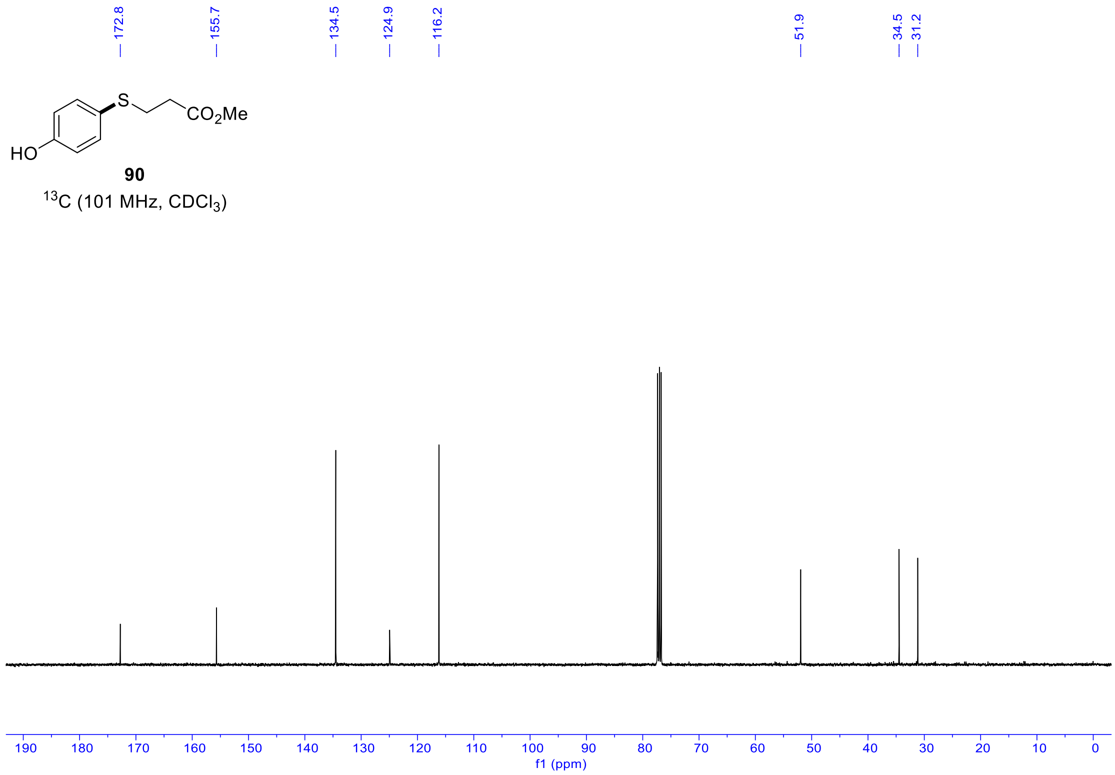

Supplementary Figure 235. ^{13}**C NMR** of compound **90**

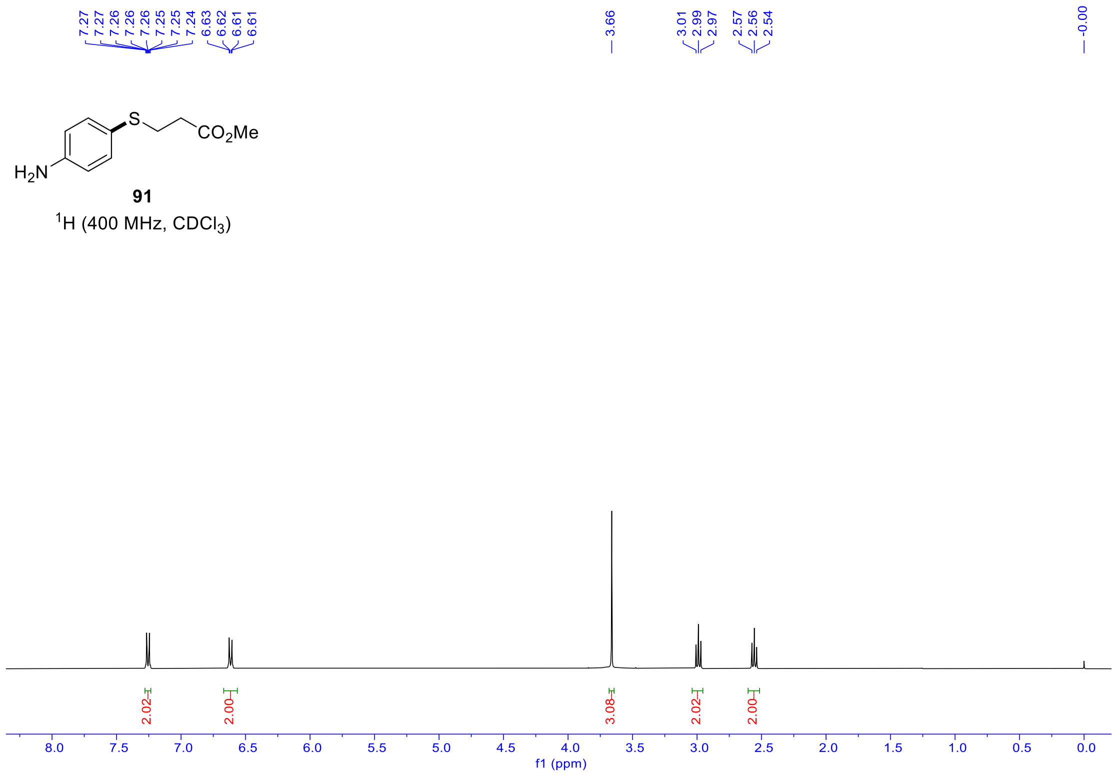

Supplementary Figure 236. ^{1}**H NMR** of compound **91**

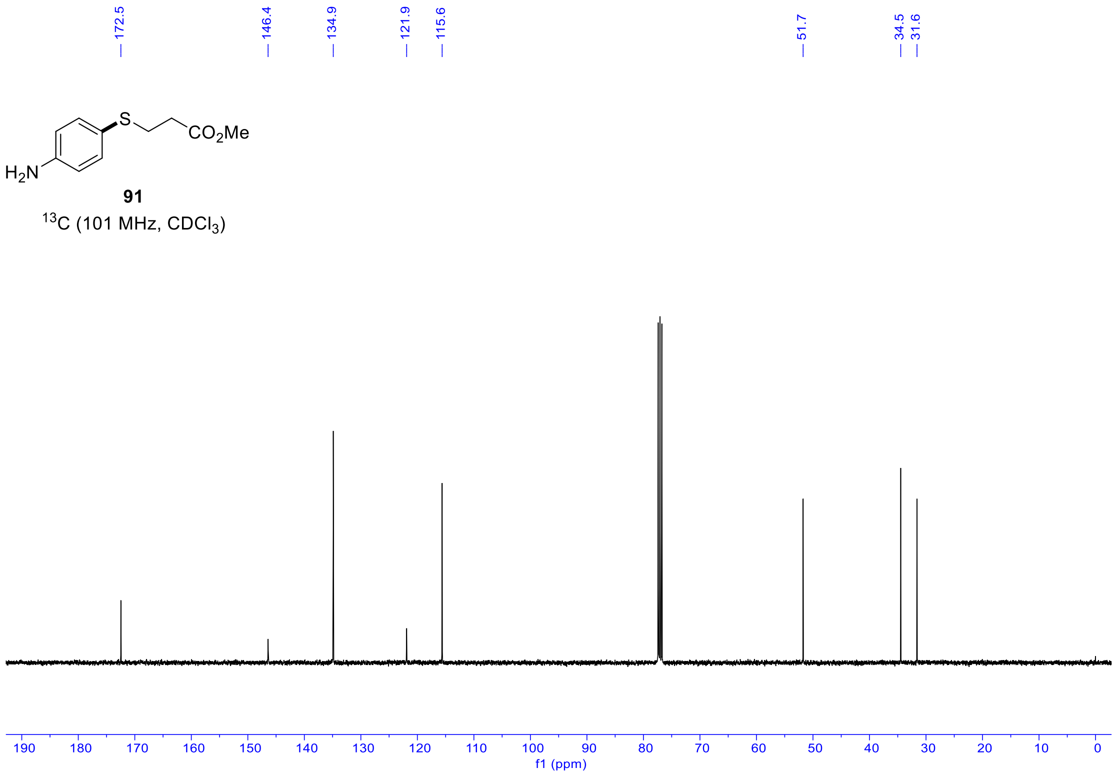

Supplementary Figure 237. ^{13}**C NMR** of compound **91**

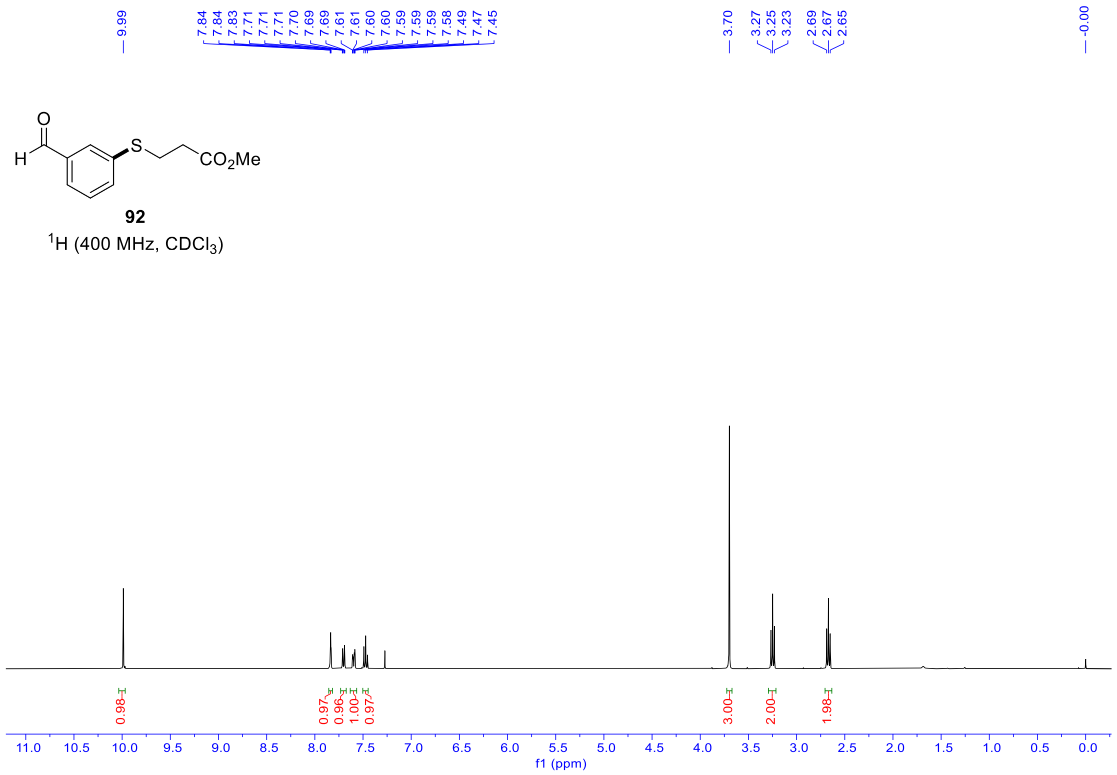

Supplementary Figure 238. **^{1}H NMR** of compound **92**

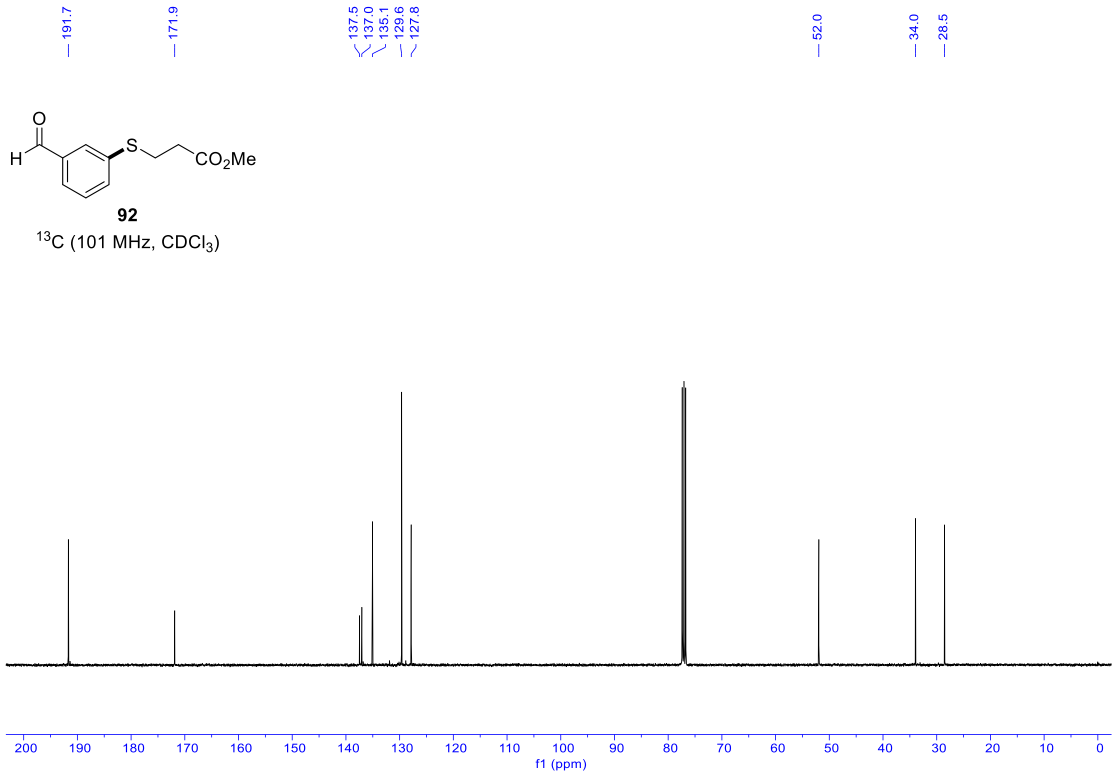

Supplementary Figure 239. **^{13}C NMR** of compound **92**

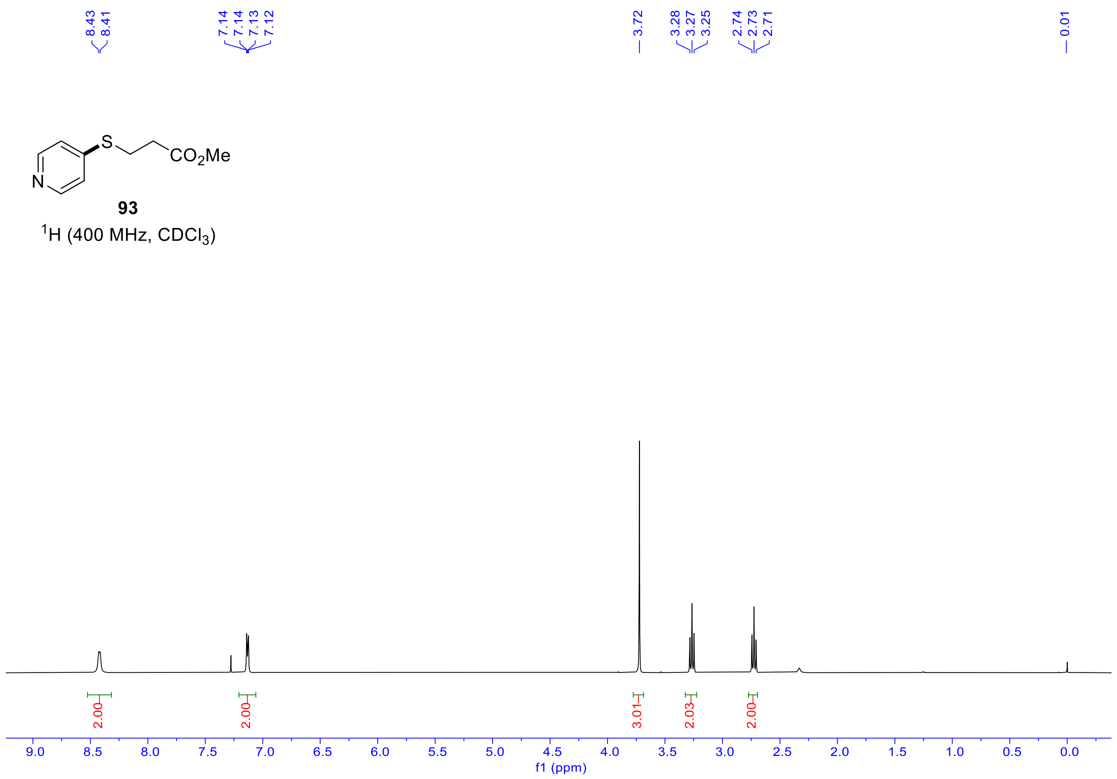

Supplementary Figure 240. **^{1}H NMR** of compound **93**

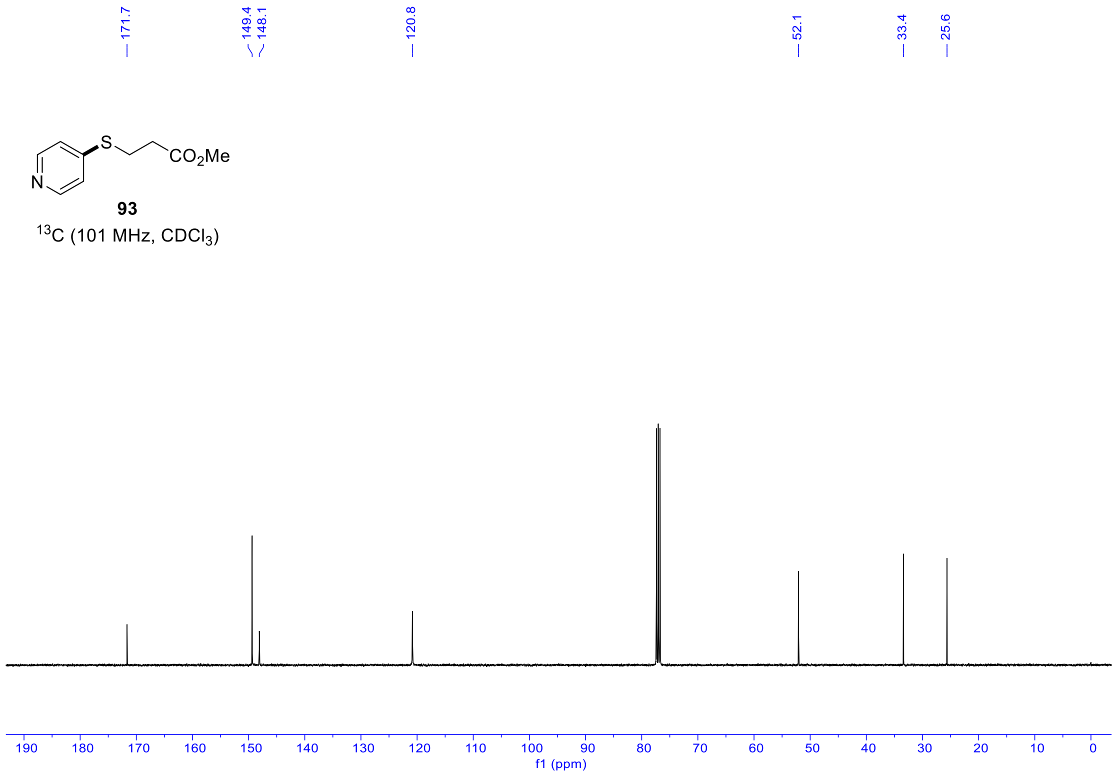

Supplementary Figure 241. **^{13}C NMR** of compound **93**

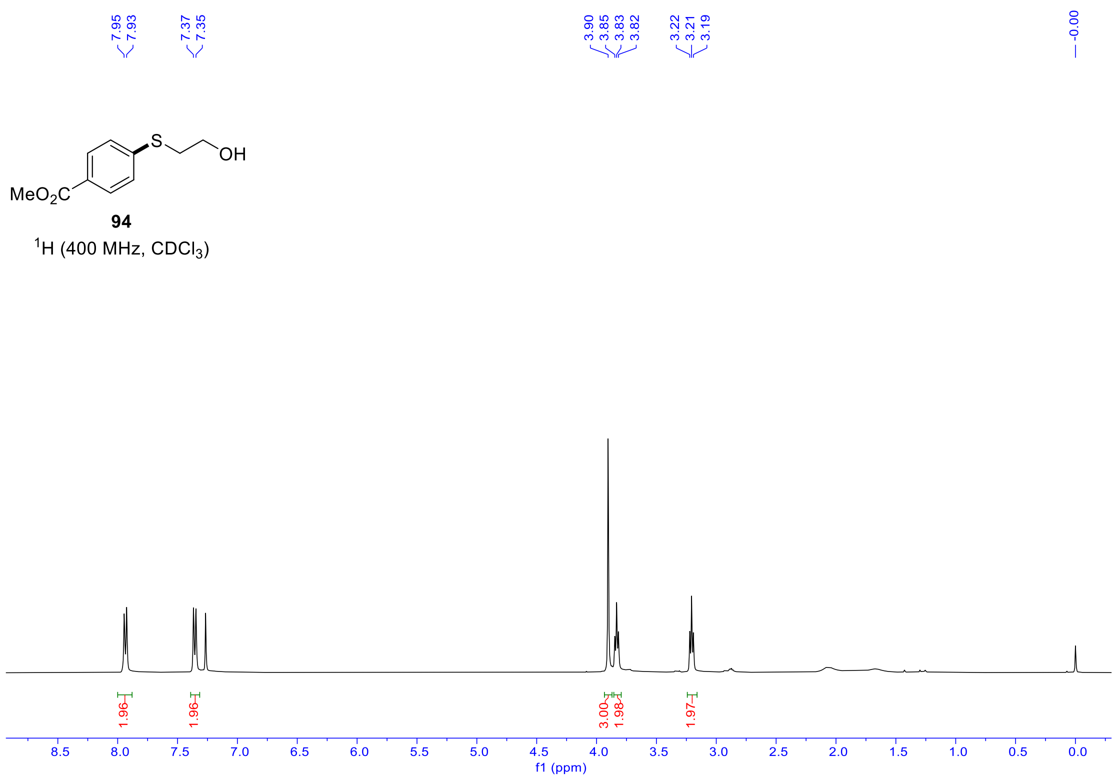

Supplementary Figure 242. ^{1}**H NMR** of compound **94**

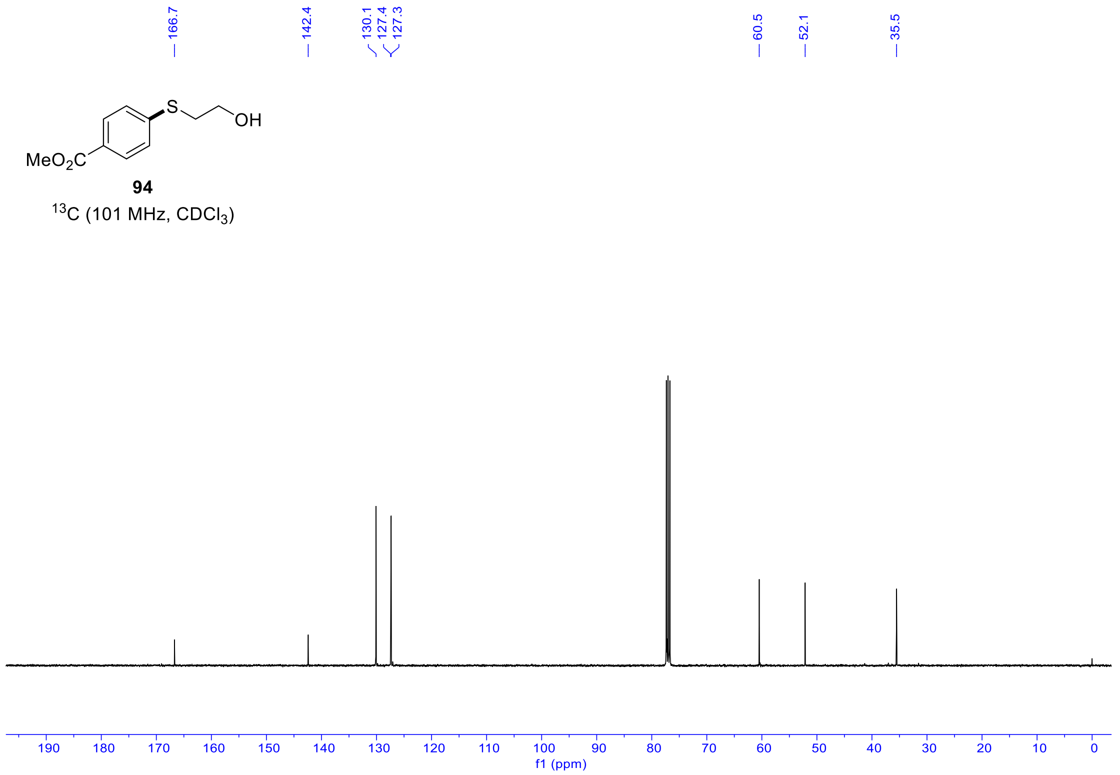

Supplementary Figure 243. ^{13}**C NMR** of compound **94**

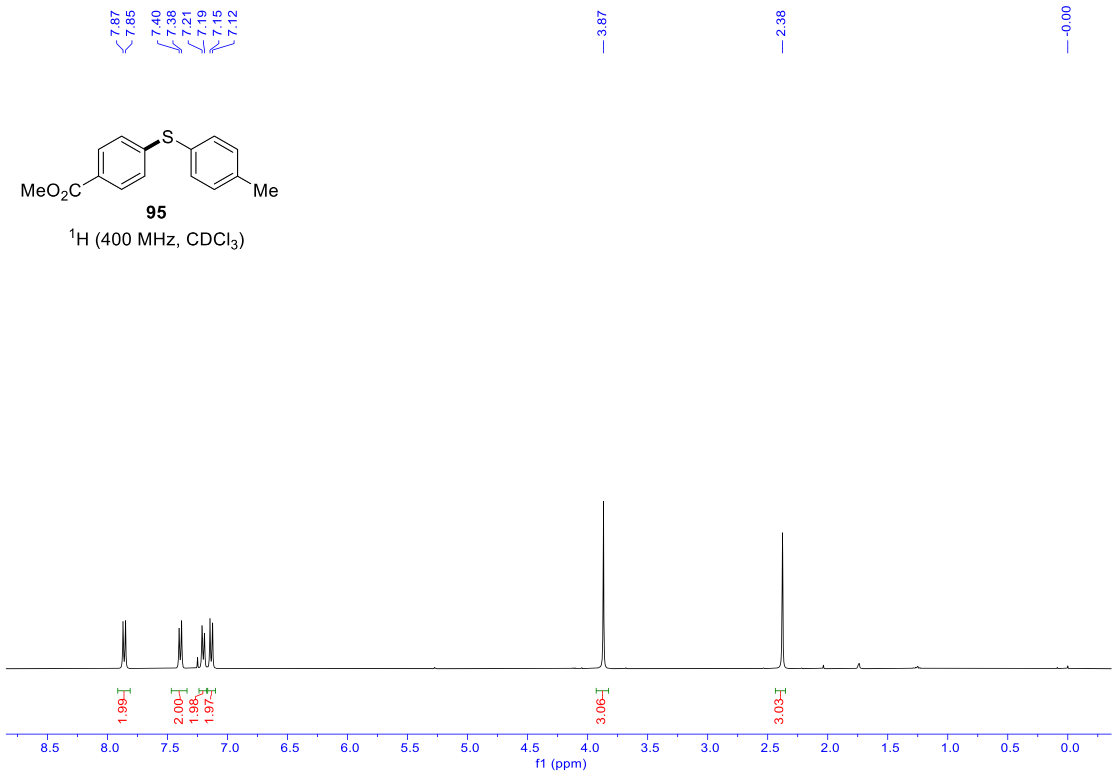

Supplementary Figure 244. ^{1}**H NMR** of compound **95**

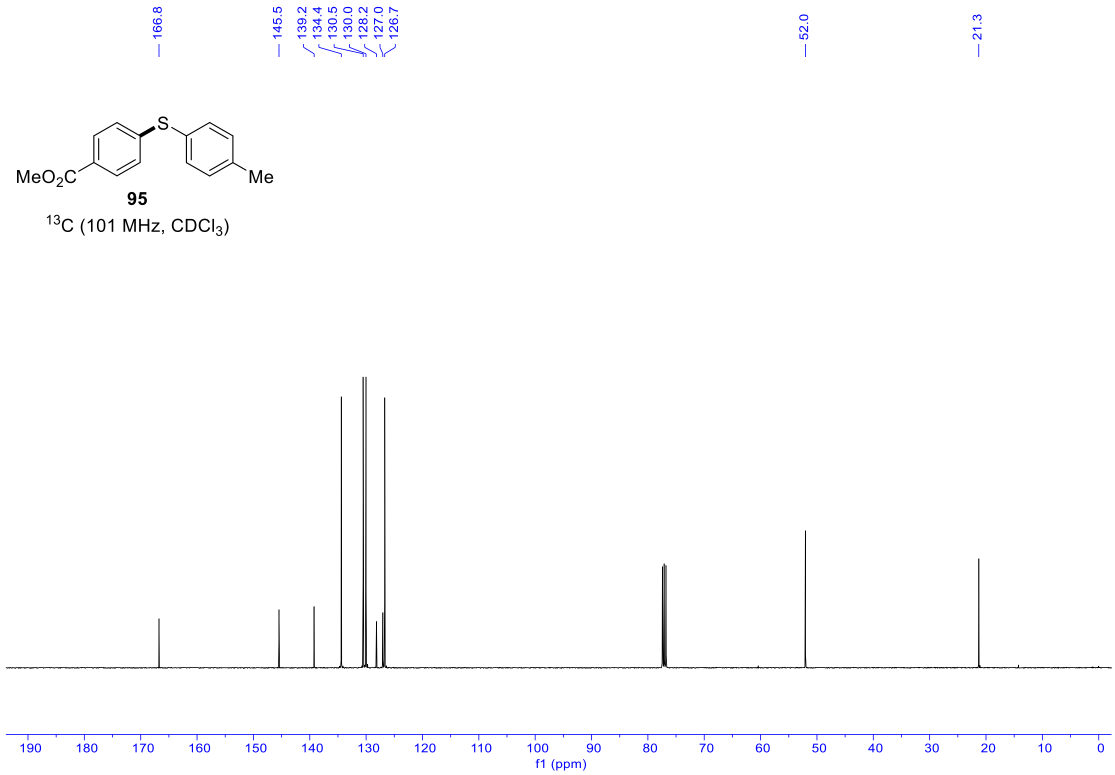

Supplementary Figure 245. ^{13}**C NMR** of compound **95**

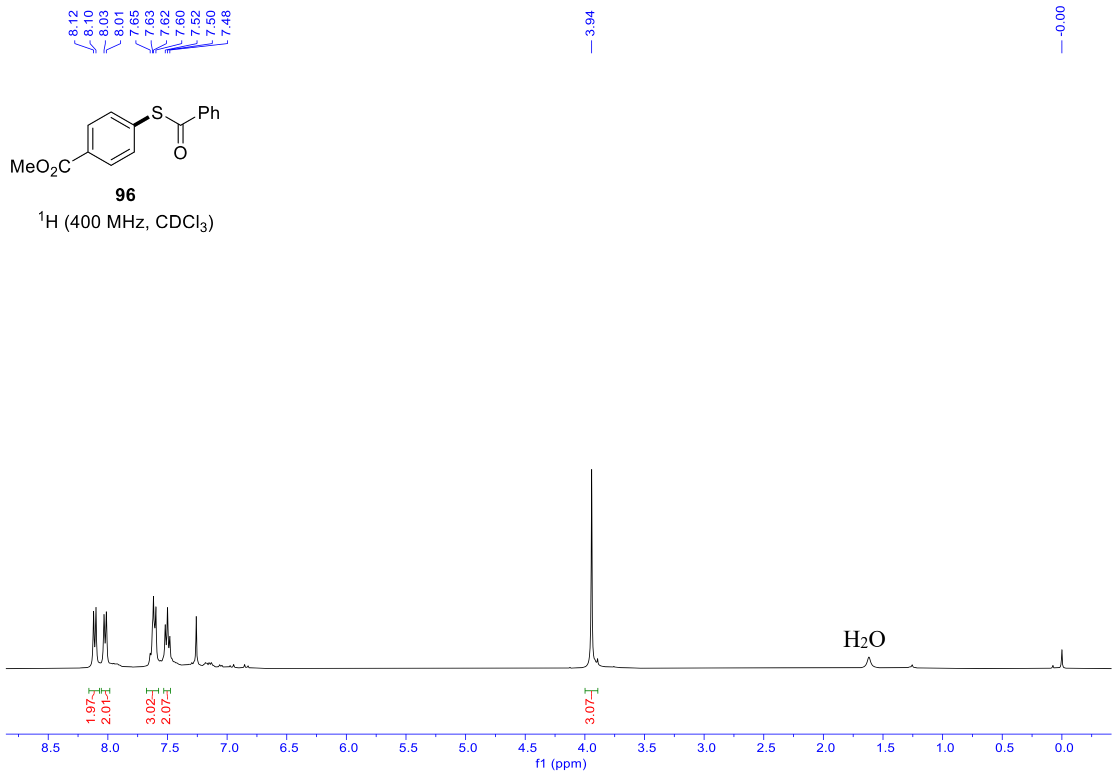

Supplementary Figure 246. ^{1}**H NMR** of compound **96**

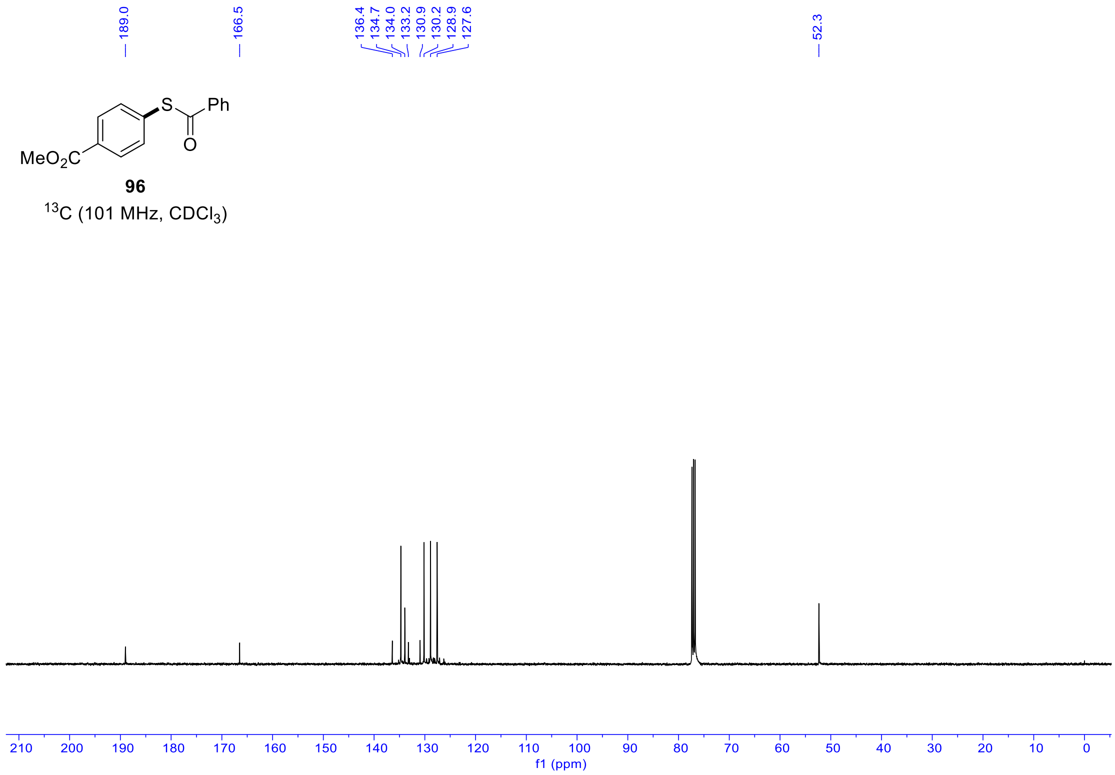

Supplementary Figure 247. ^{13}**C NMR** of compound **96**

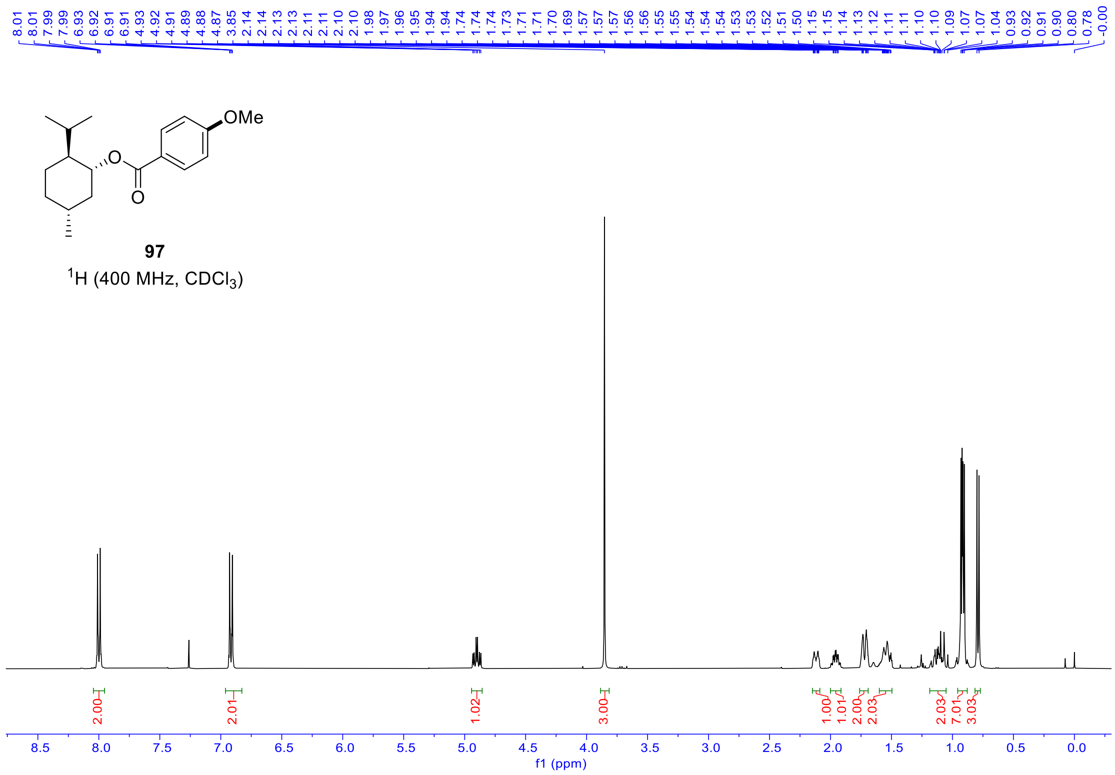

Supplementary Figure 248. ^{1}H NMR of compound **97**

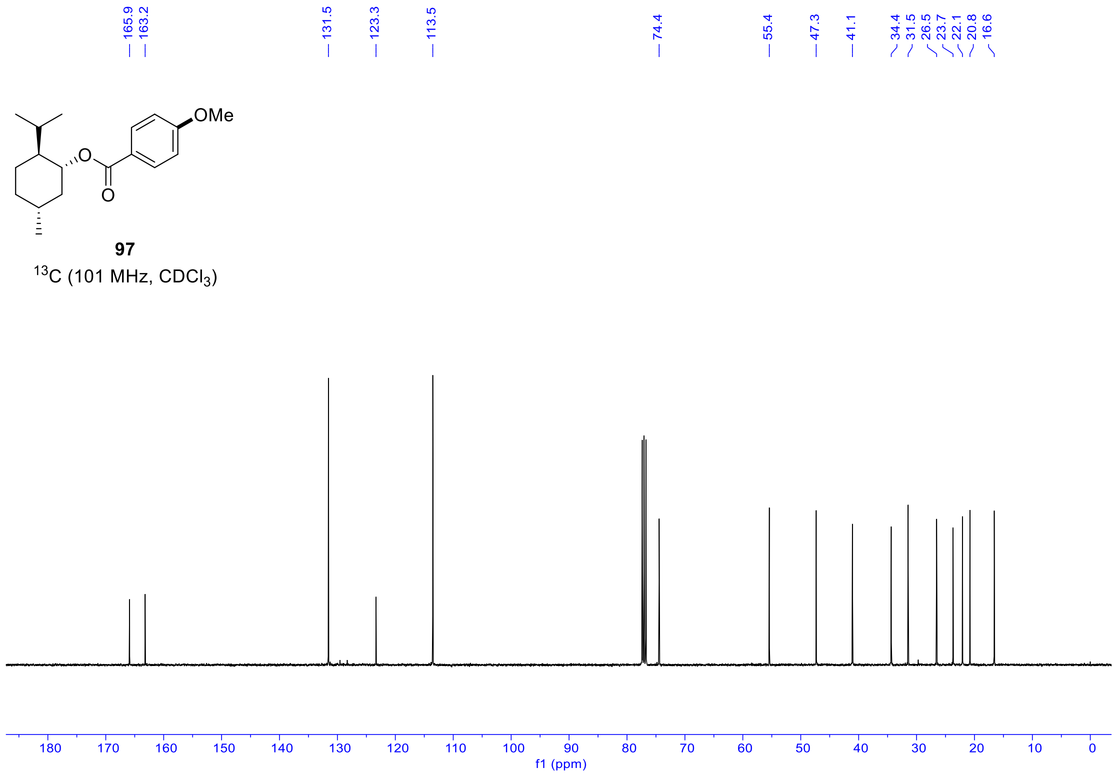

Supplementary Figure 249. ^{13}C NMR of compound **97**

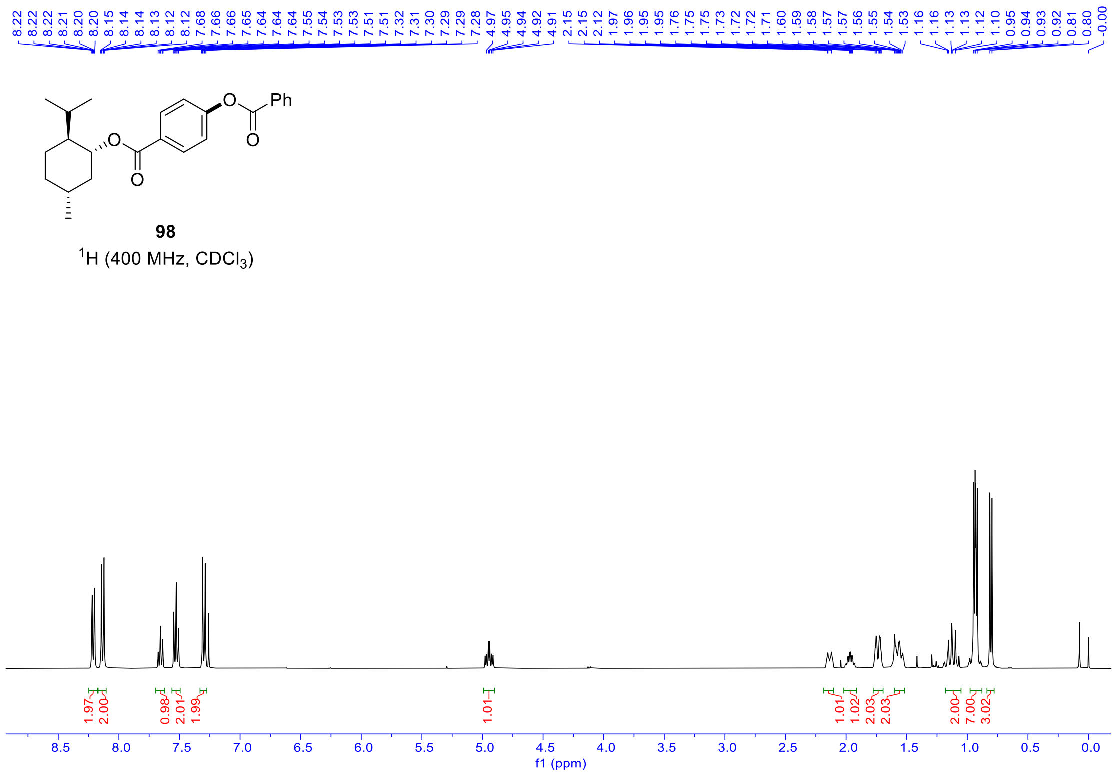

Supplementary Figure 250. ^{1}**H NMR** of compound **98**

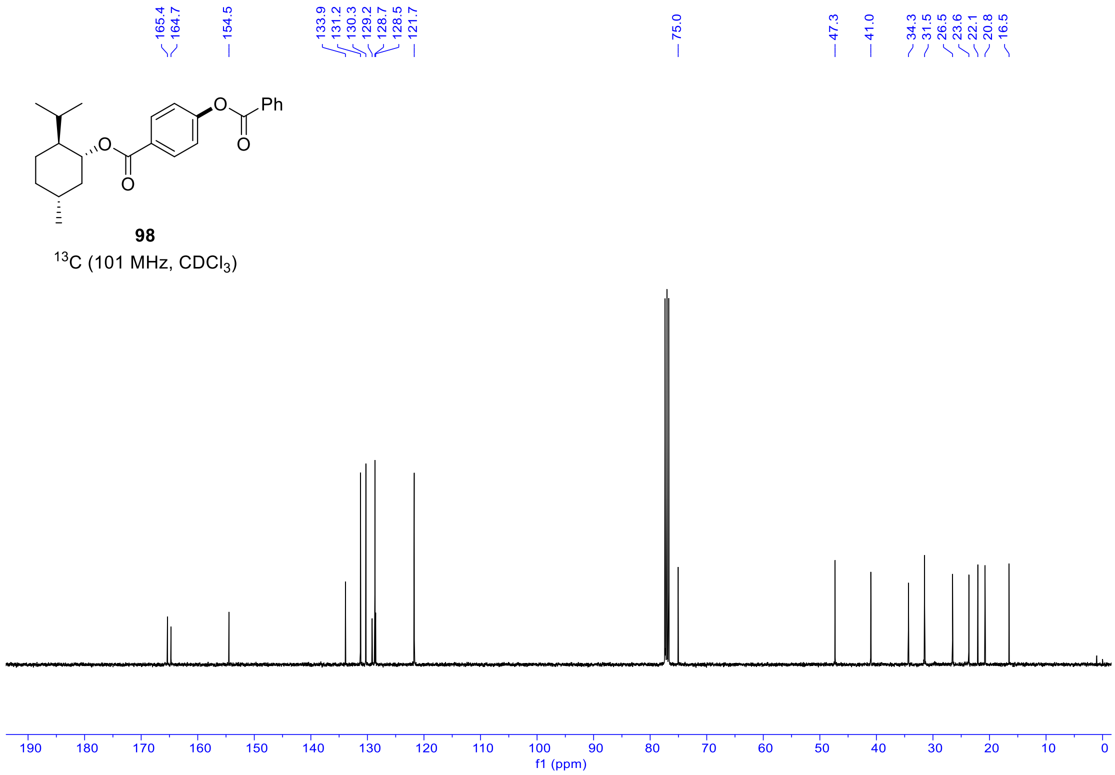

Supplementary Figure 251. ^{1}**H NMR** of compound **98**

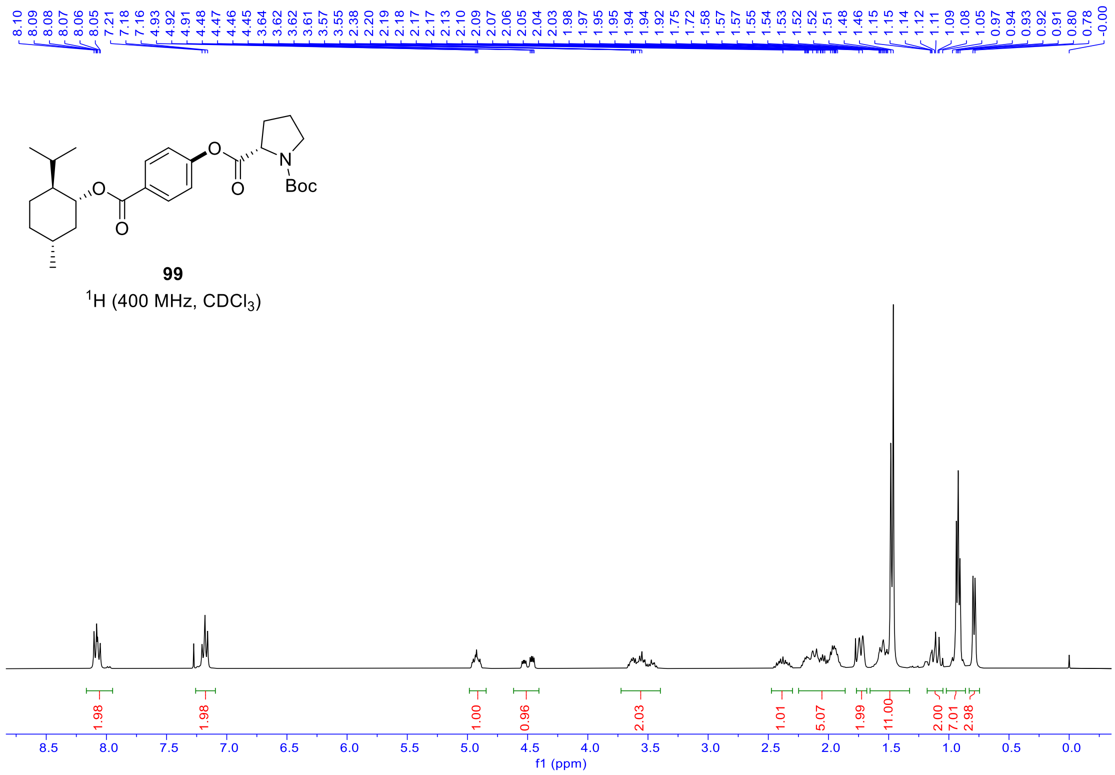

Supplementary Figure 252. ^{1}H NMR of compound **99**

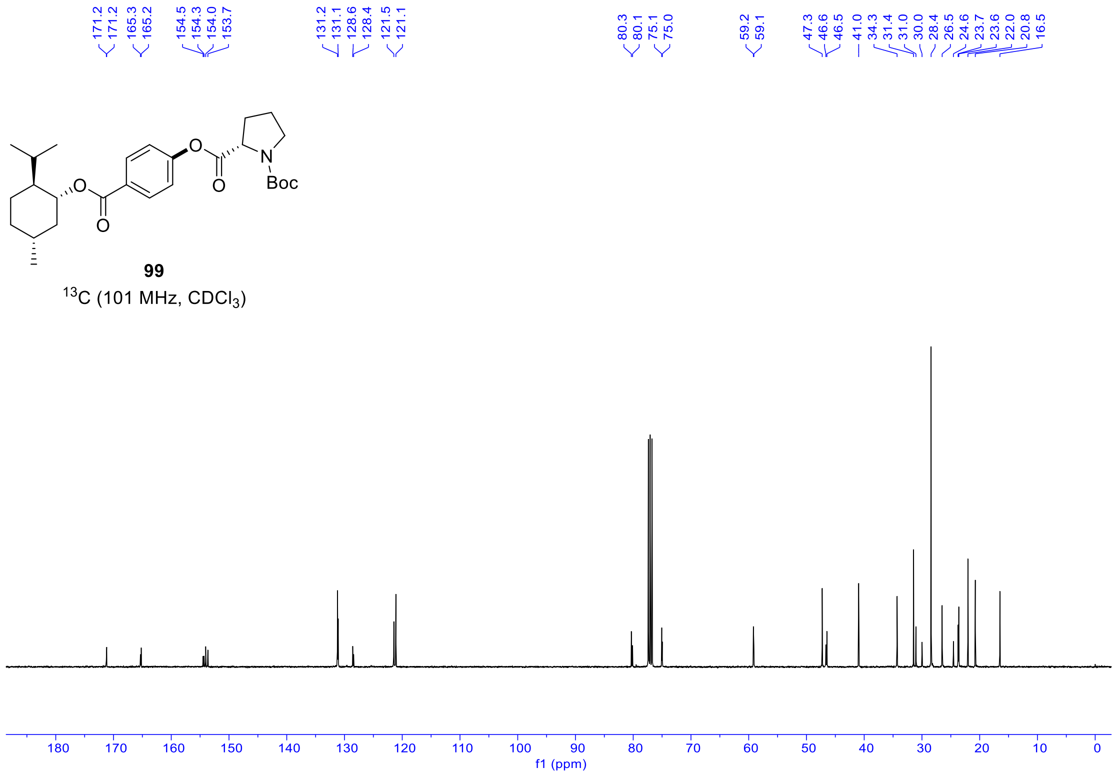

Supplementary Figure 253. ^{13}C NMR of compound **99**

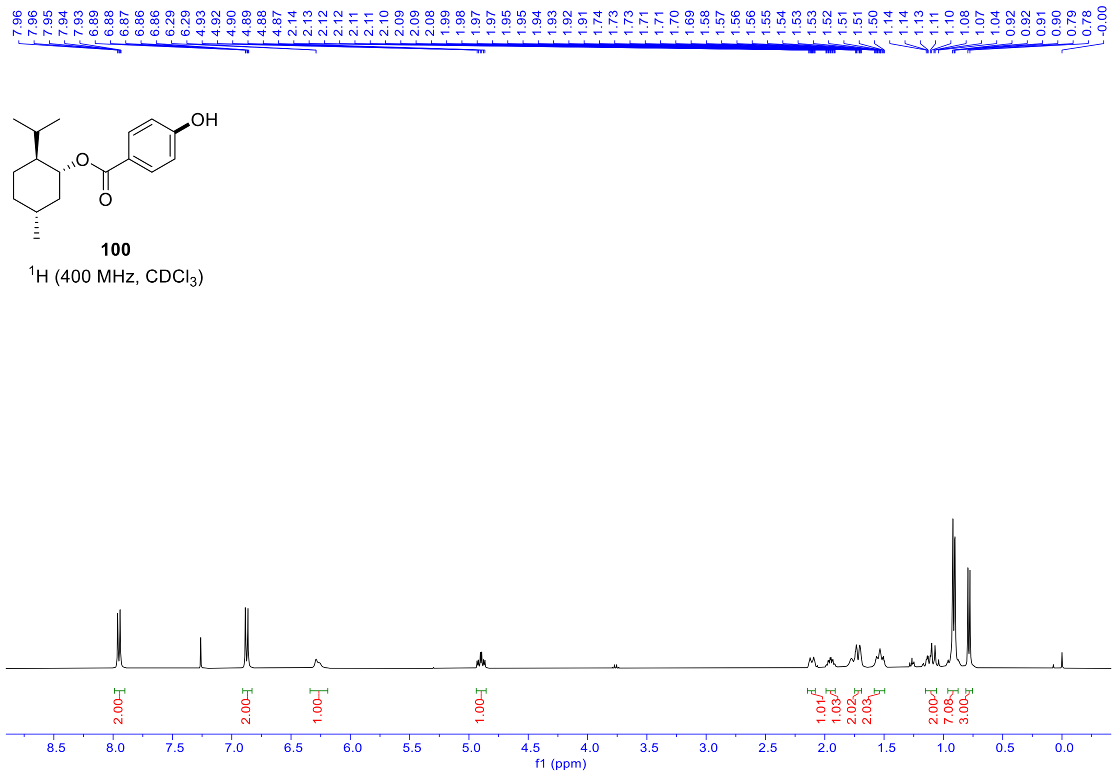

Supplementary Figure 254. ^{1}**H NMR** of compound **100**

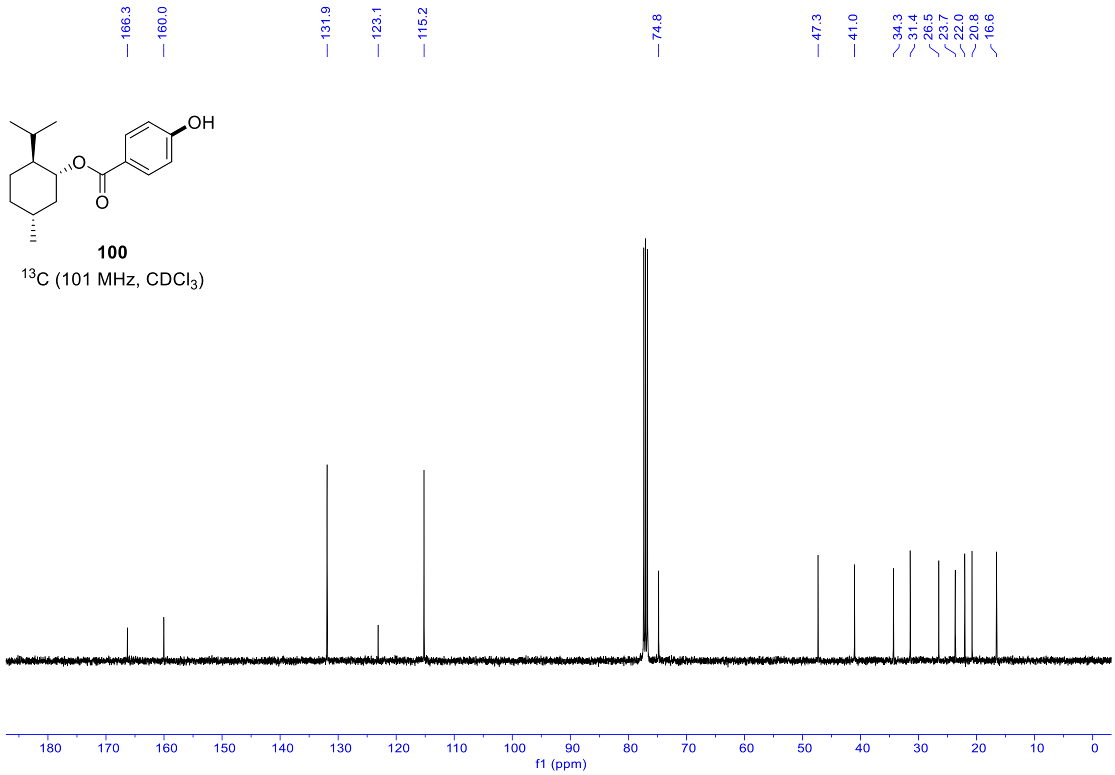

Supplementary Figure 255. ^{13}**C NMR** of compound **100**

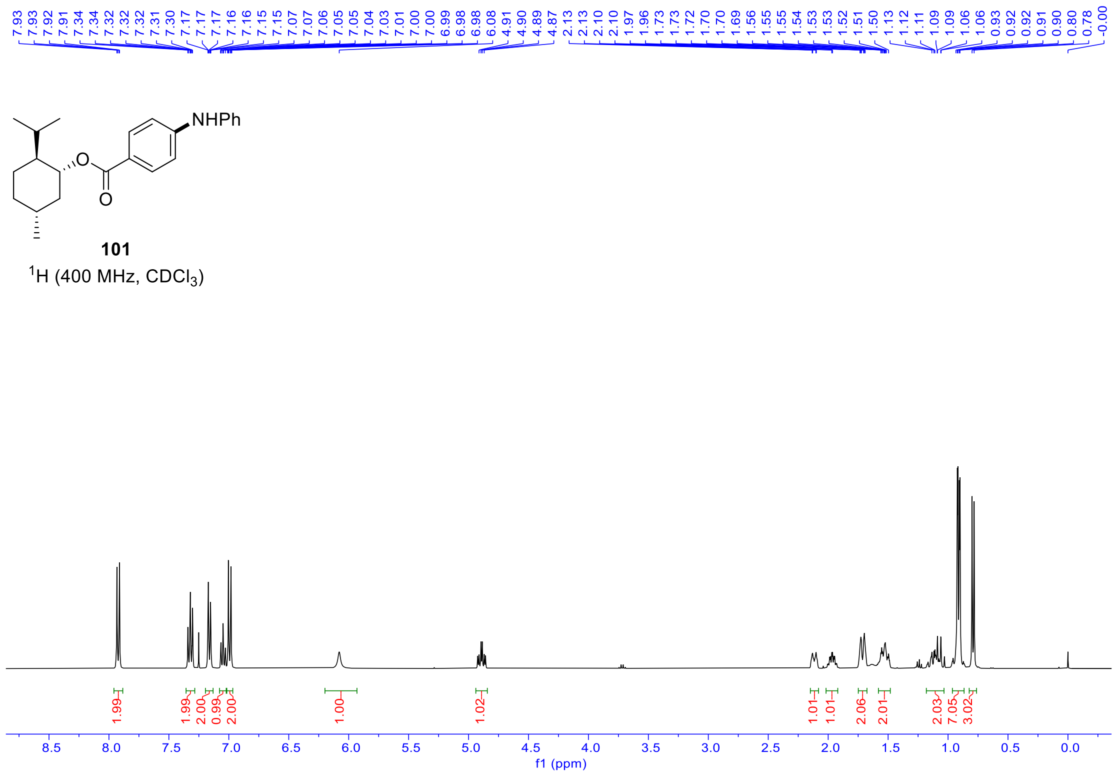

Supplementary Figure 256. ^{1}H NMR of compound 101

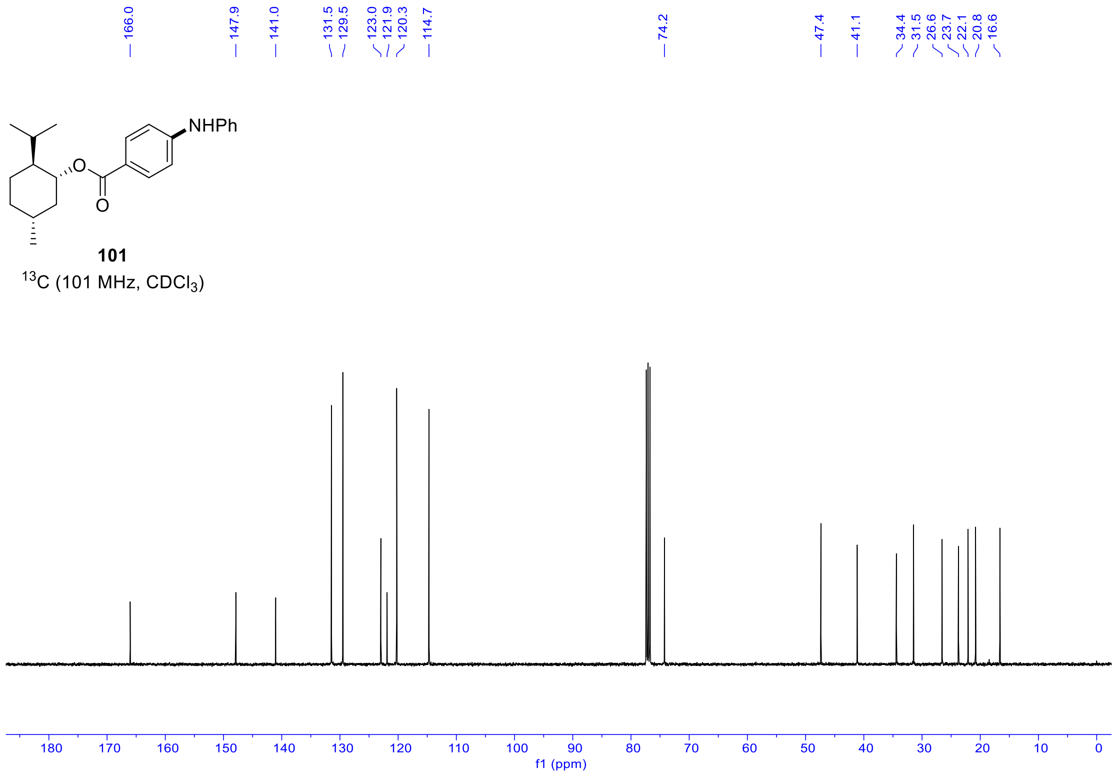

Supplementary Figure 257. ^{13}C NMR of compound 101

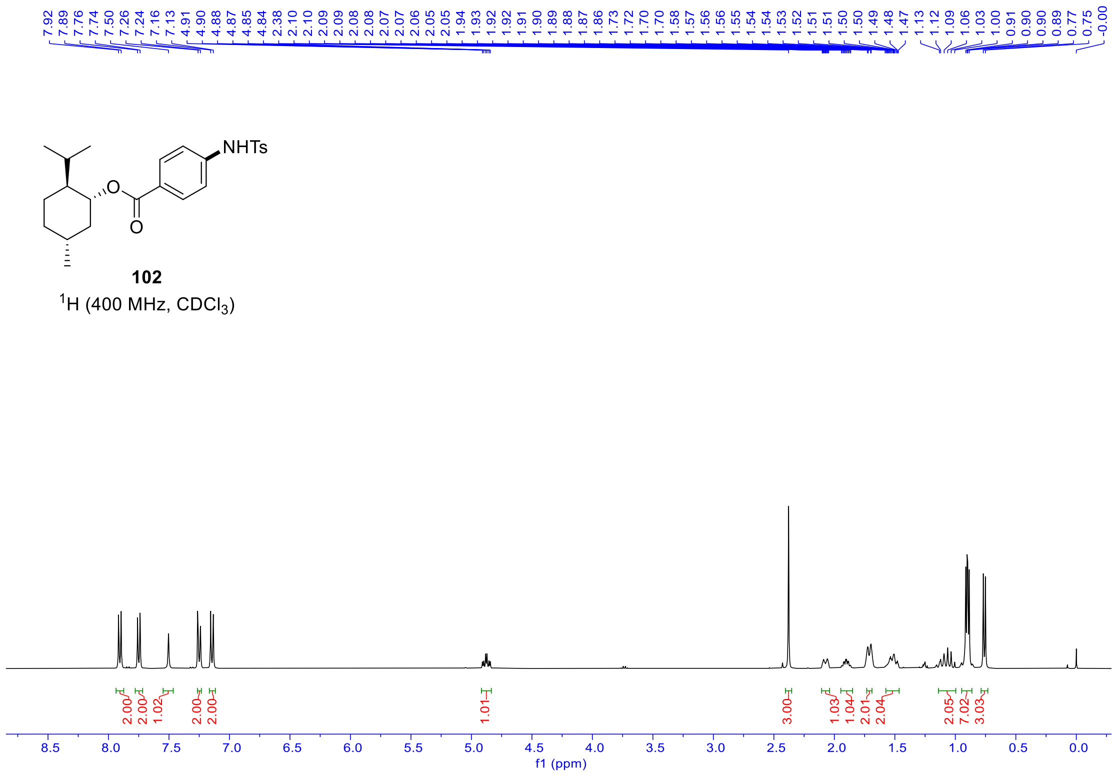

Supplementary Figure 258. ^{1}**H NMR** of compound **102**

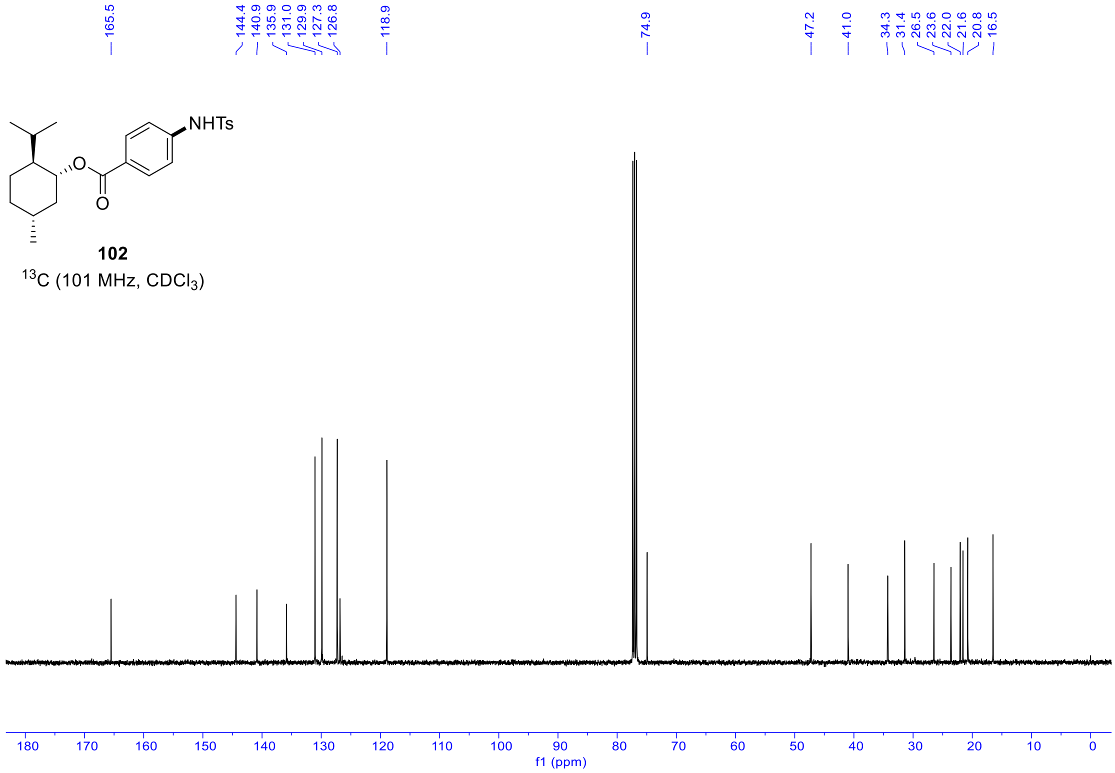

Supplementary Figure 259. ^{13}**C NMR** of compound **102**

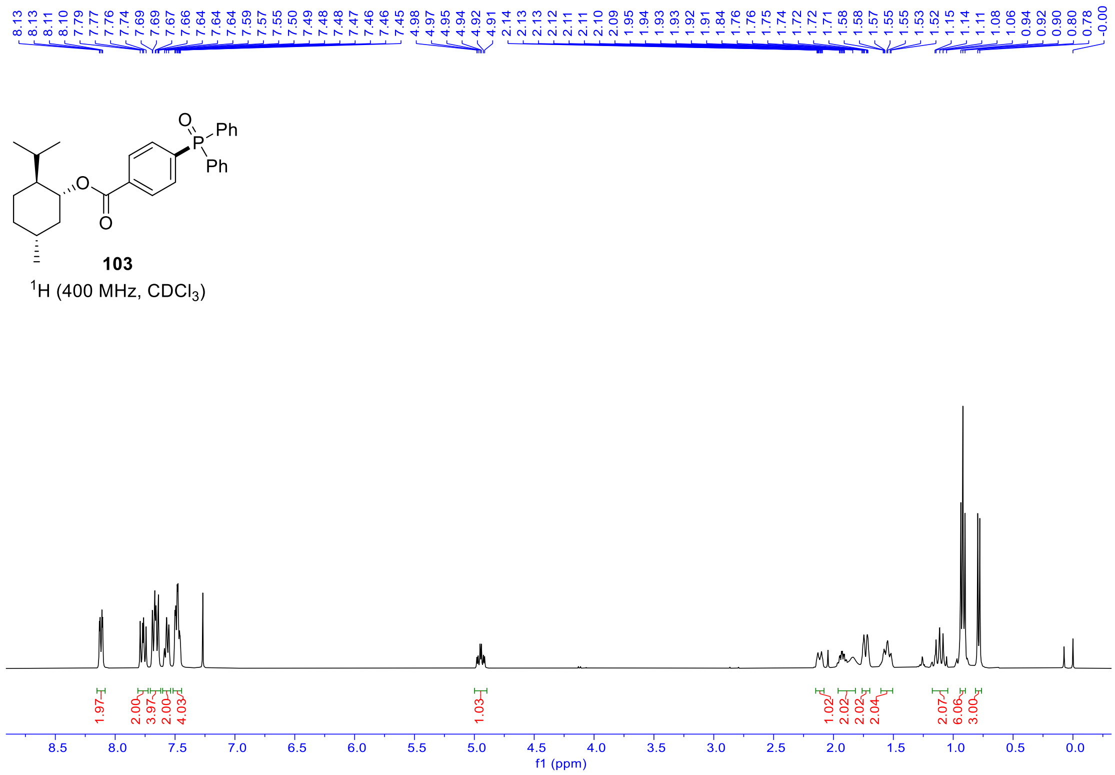

Supplementary Figure 260. ^{1}**H NMR** of compound **103**

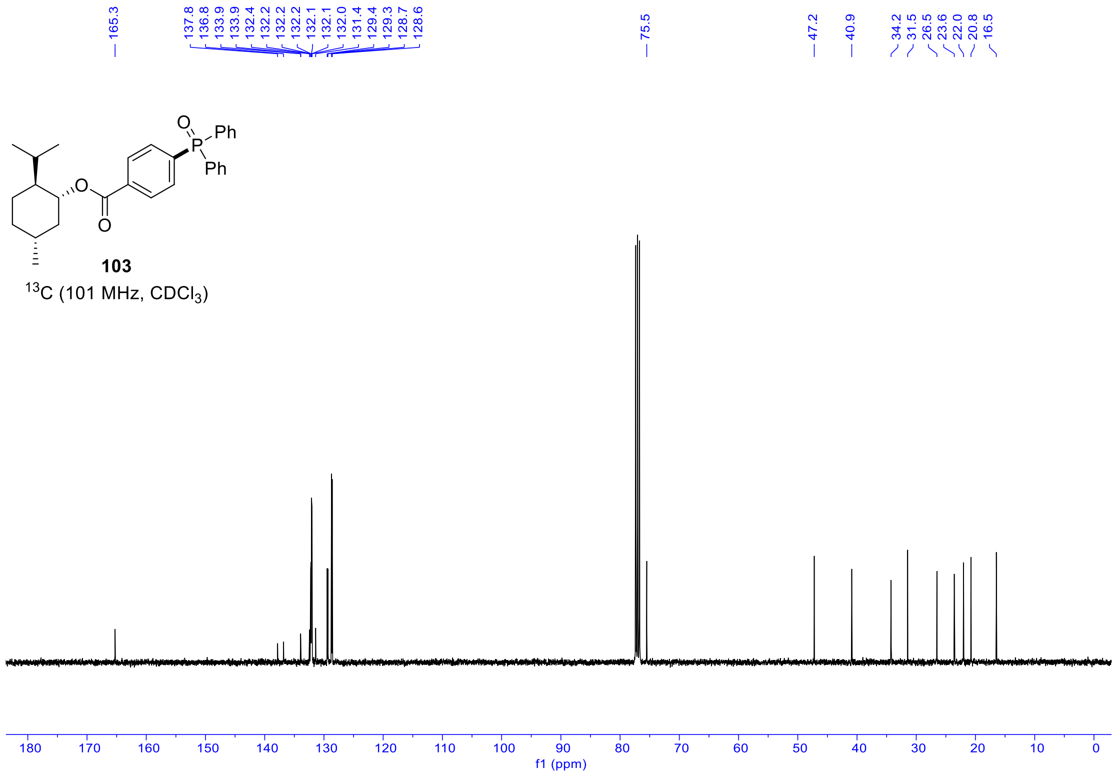

Supplementary Figure 261. ^{13}**C NMR** of compound **103**

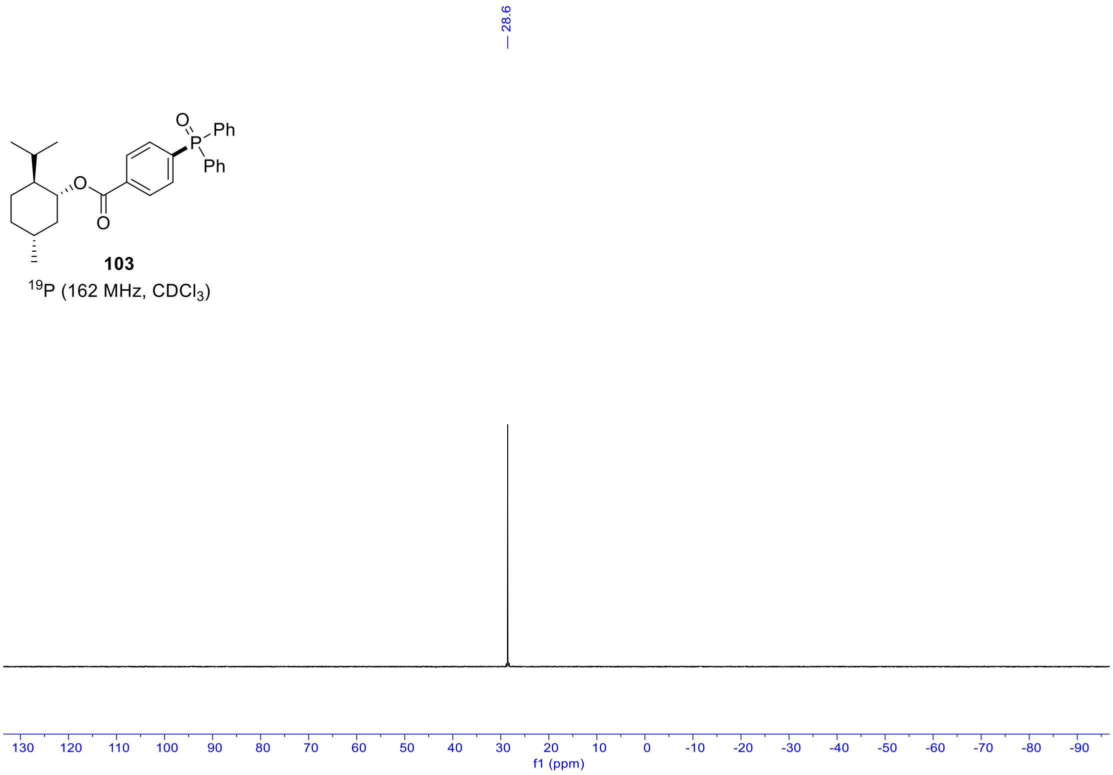

Supplementary Figure 262. ^{31}**P NMR** of compound **103**

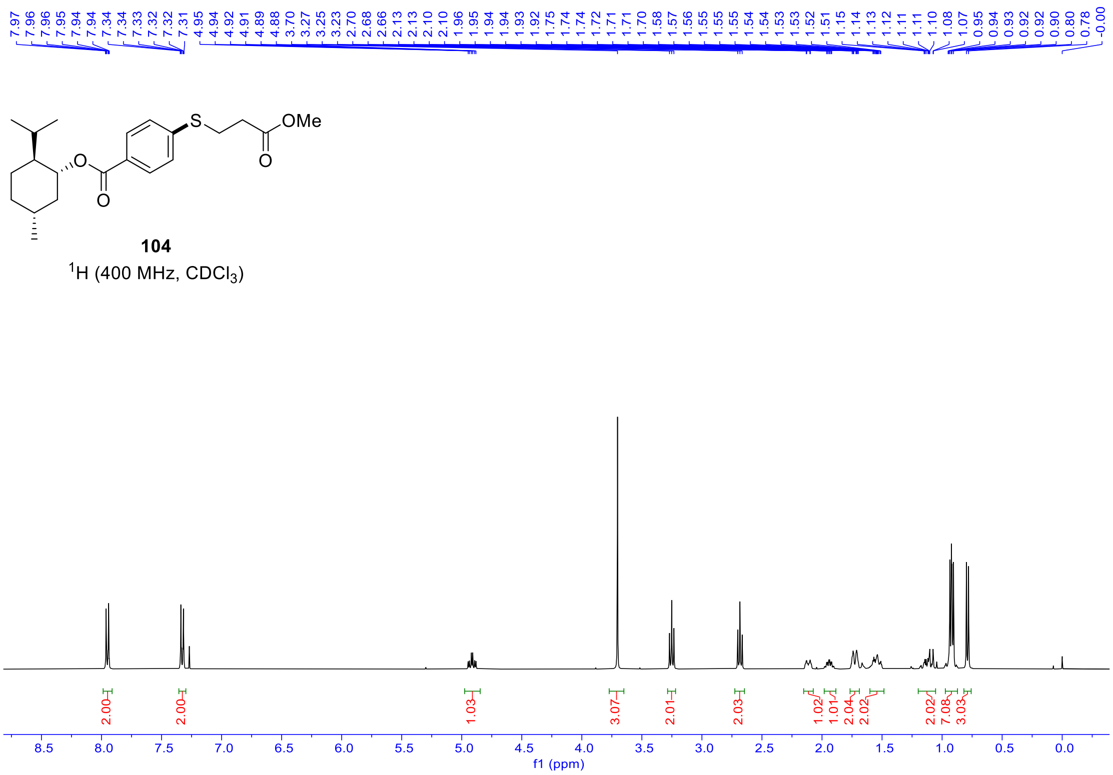

Supplementary Figure 263. ^{1}**H NMR** of compound **104**

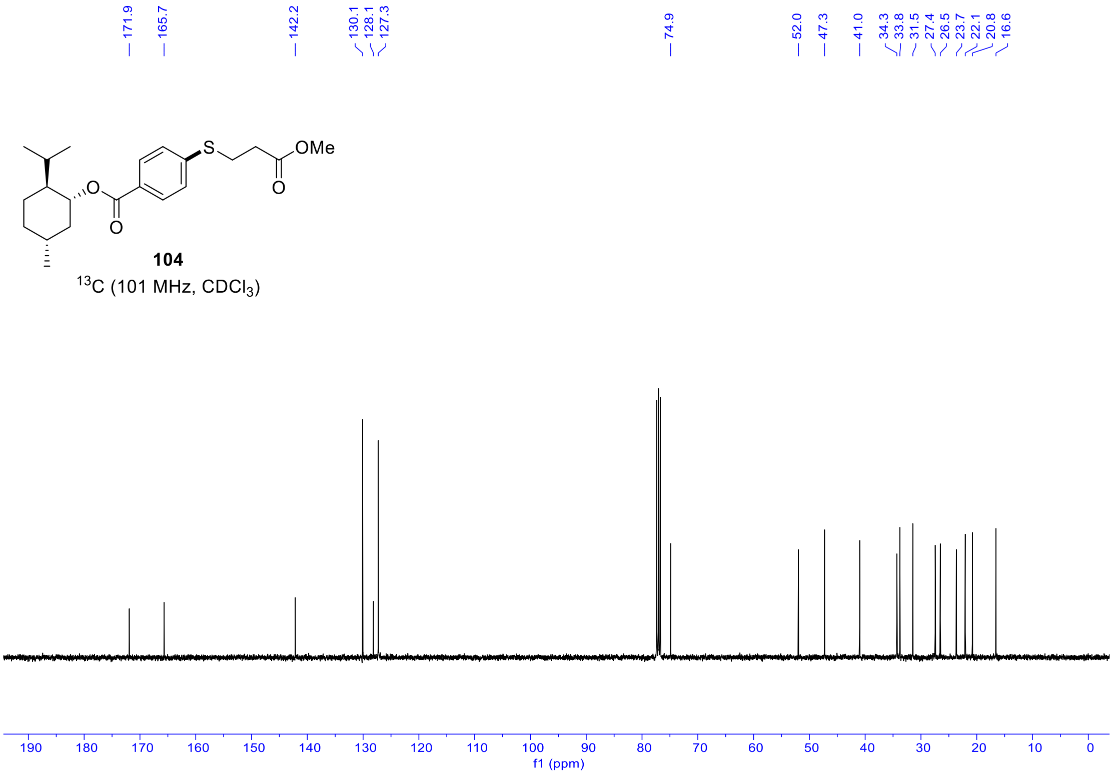

Supplementary Figure 264. **^{13}C NMR** of compound **104**

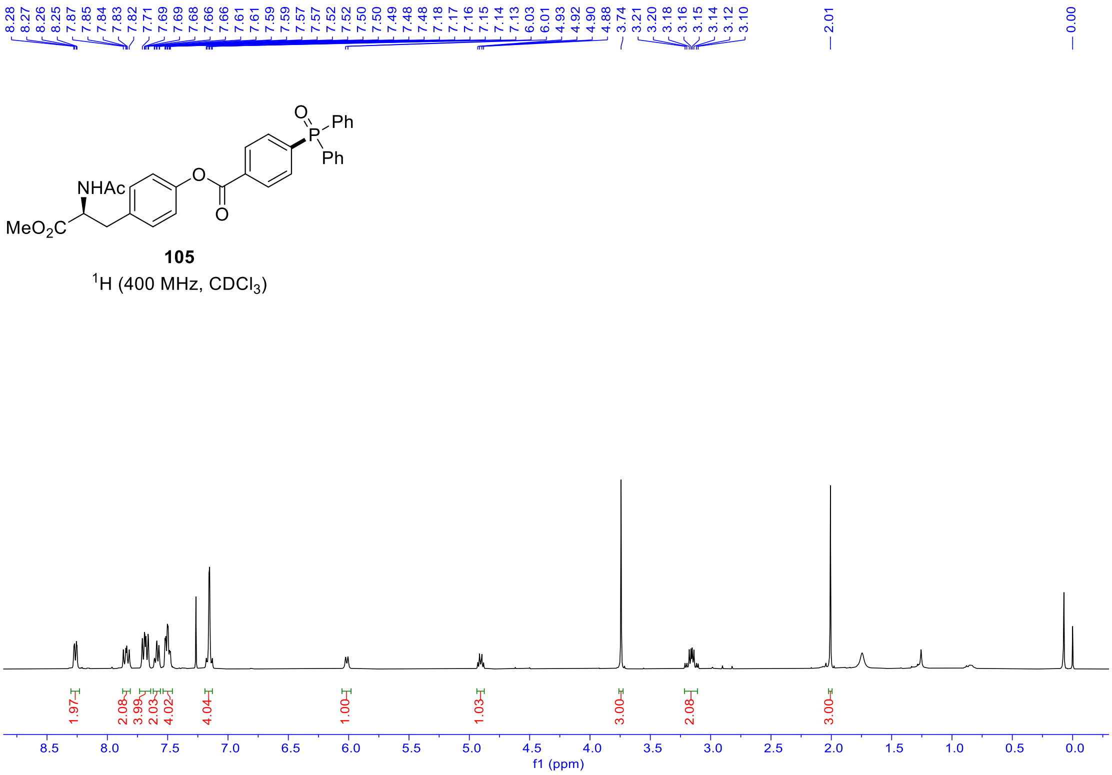

Supplementary Figure 265. **^{1}H NMR** of compound **105**

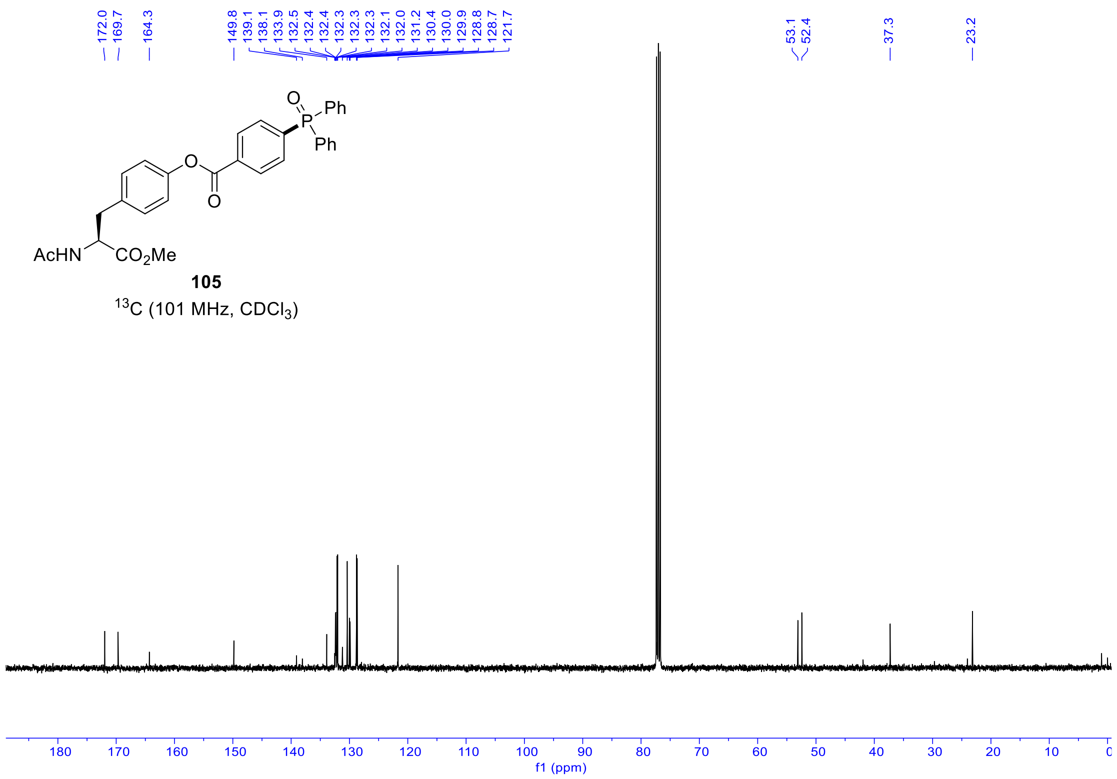

Supplementary Figure 266. ^{13}**C NMR** of compound **105**

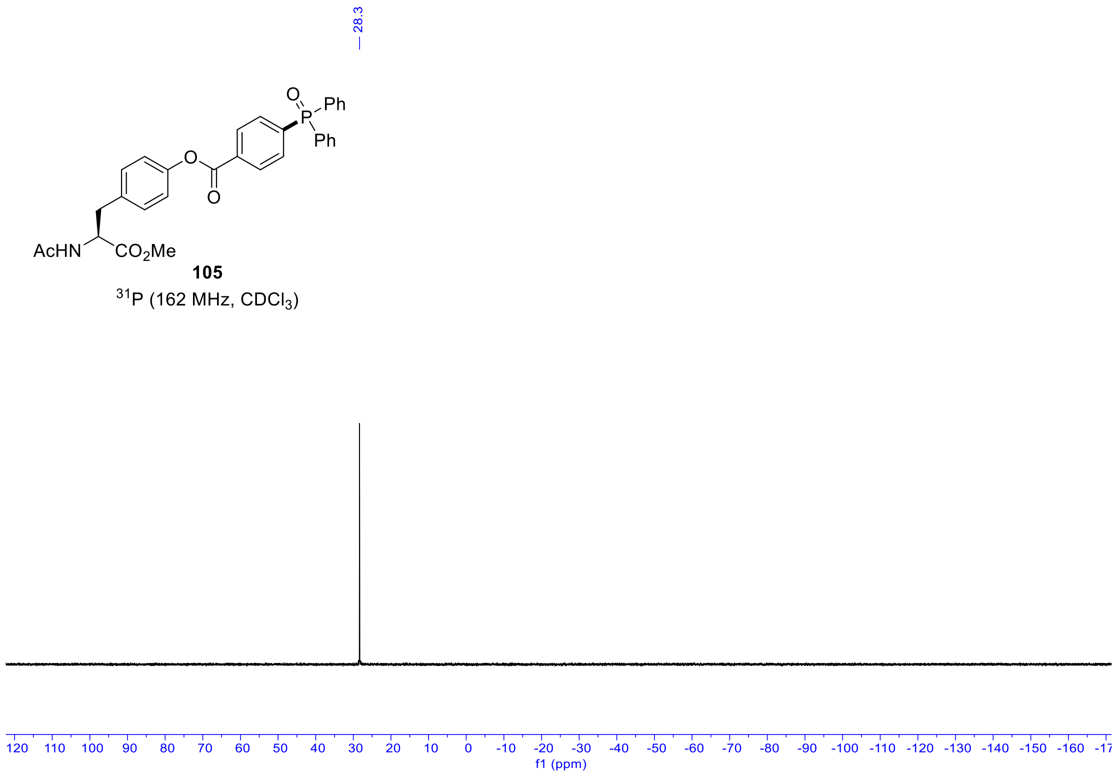

Supplementary Figure 267. ^{31}**P NMR** of compound **105**

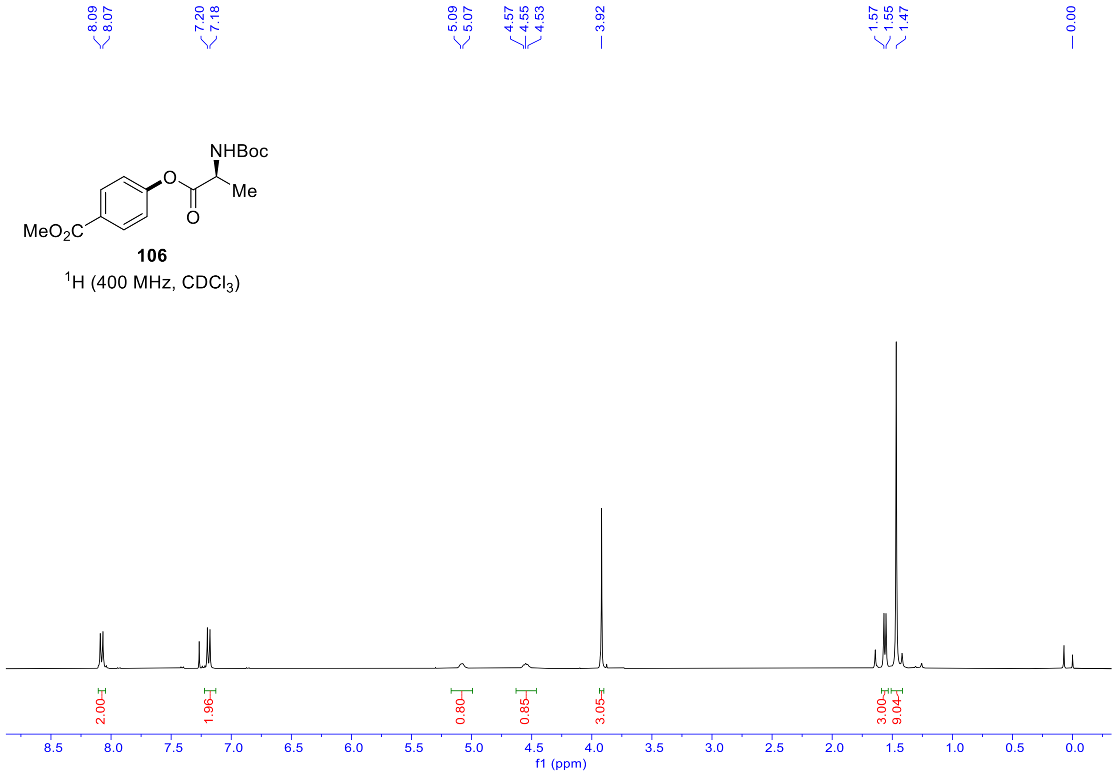

Supplementary Figure 268. ^{1}**H NMR** of compound **106**

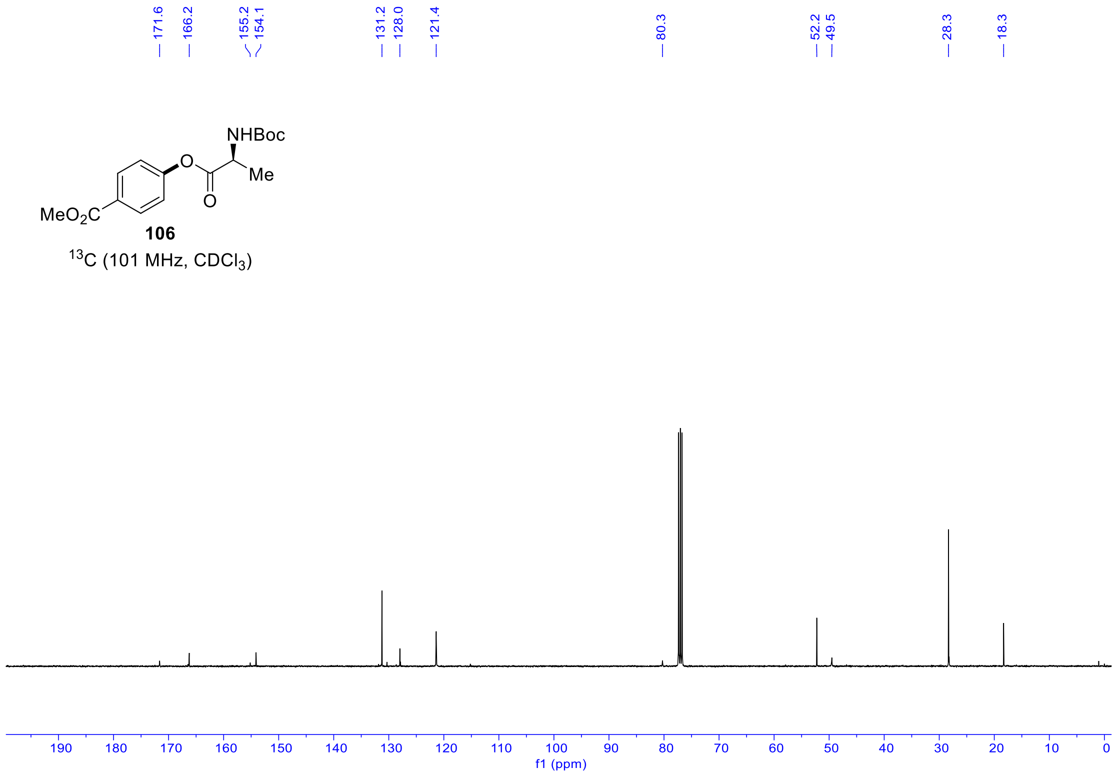

Supplementary Figure 269. ^{13}**C NMR** of compound **106**

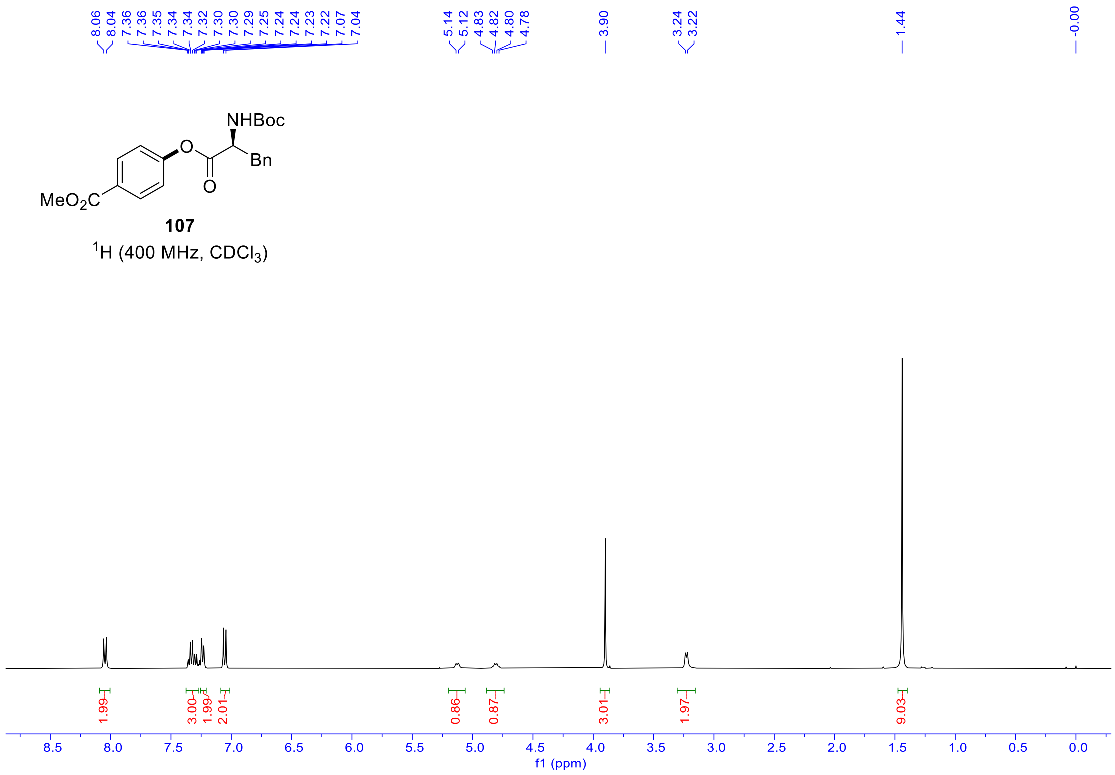

Supplementary Figure 270. ^{1}**H NMR** of compound **107**

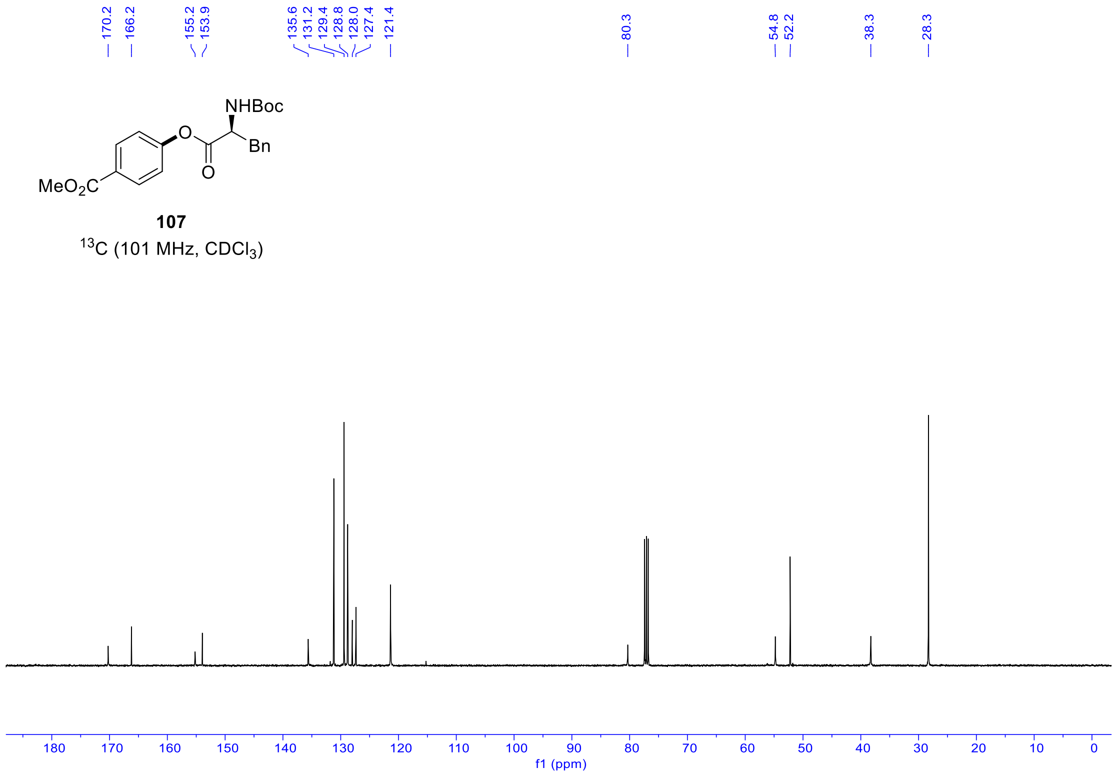

Supplementary Figure 271. ^{13}**C NMR** of compound **107**

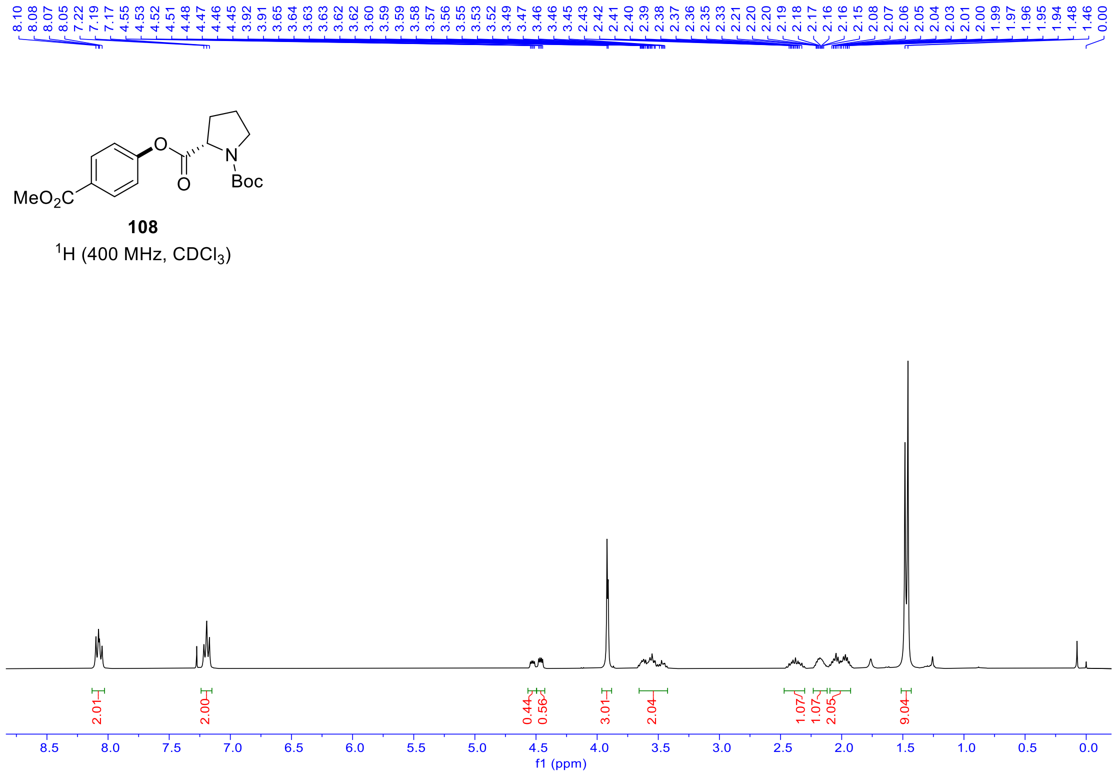

Supplementary Figure 272. ^{1}**H NMR** of compound **108**

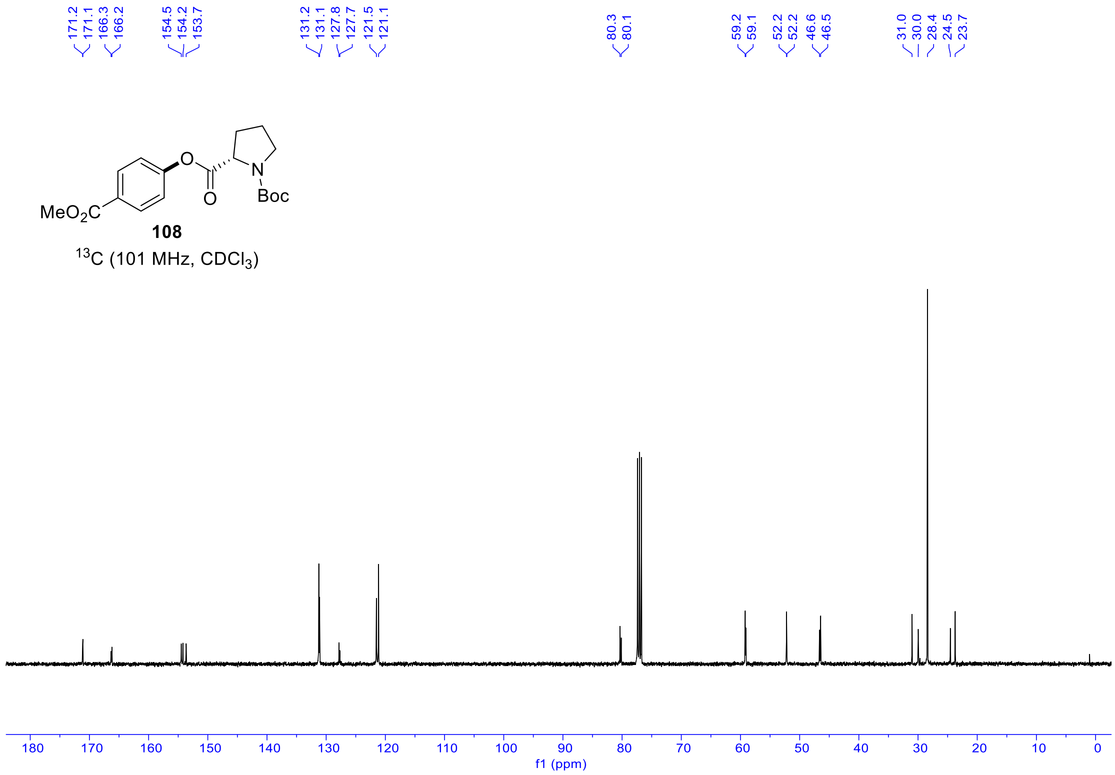

Supplementary Figure 273. ^{13}**C NMR** of compound **108**

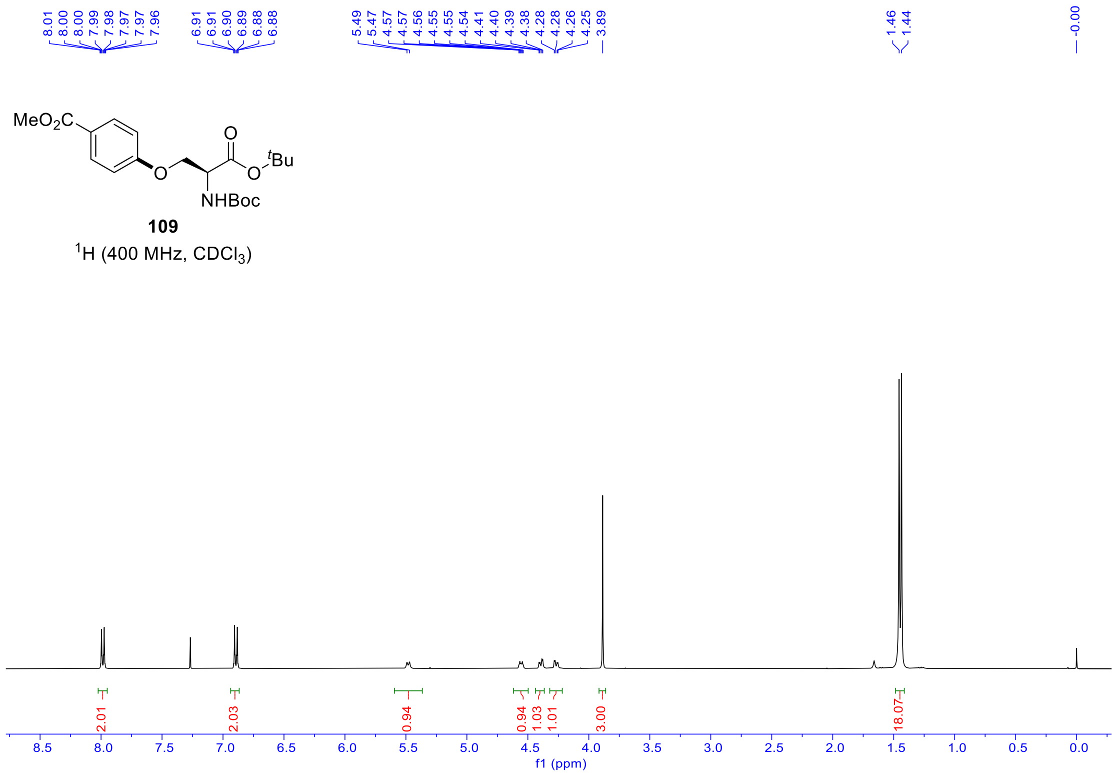

Supplementary Figure 274. ^{1}**H NMR** of compound **109**

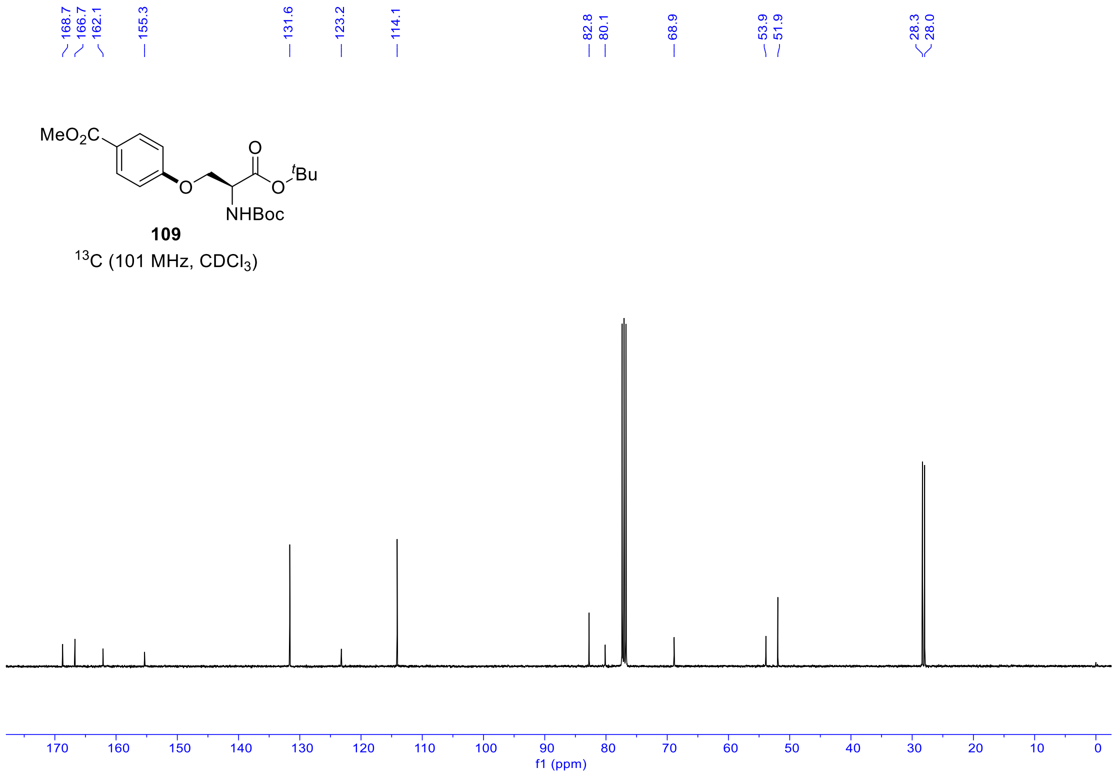

Supplementary Figure 275. ^{13}**C NMR** of compound **109**

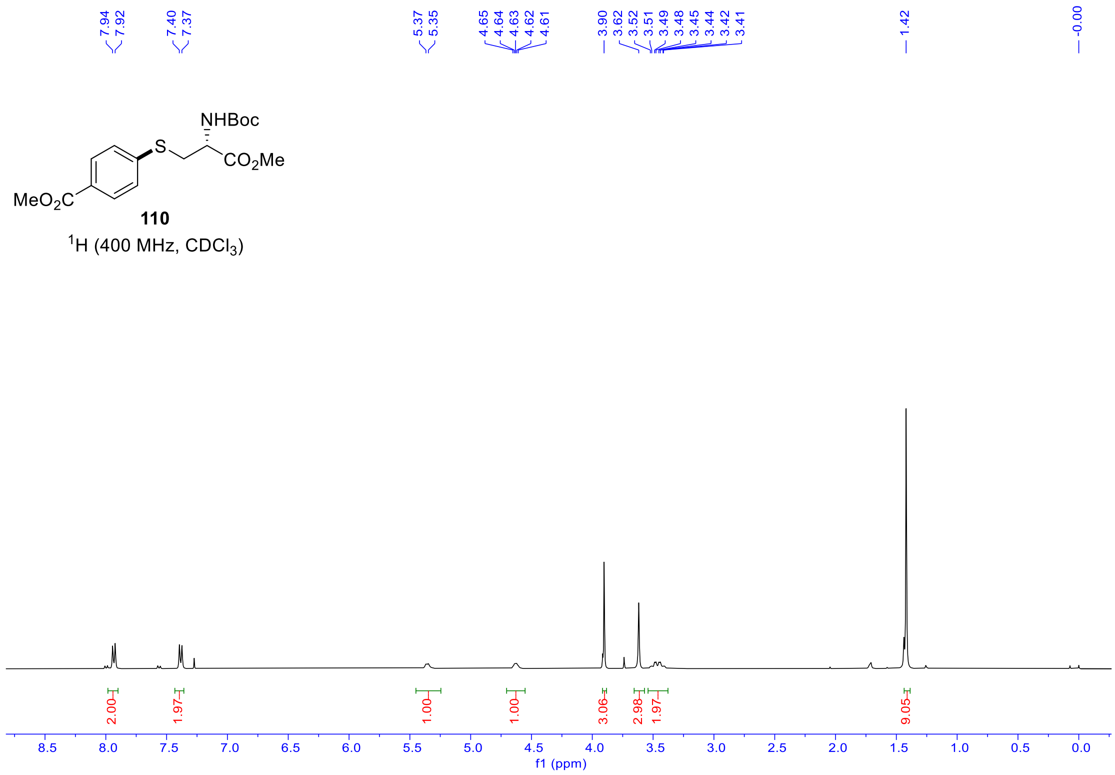

Supplementary Figure 276. ^{1}**H NMR** of compound **110**

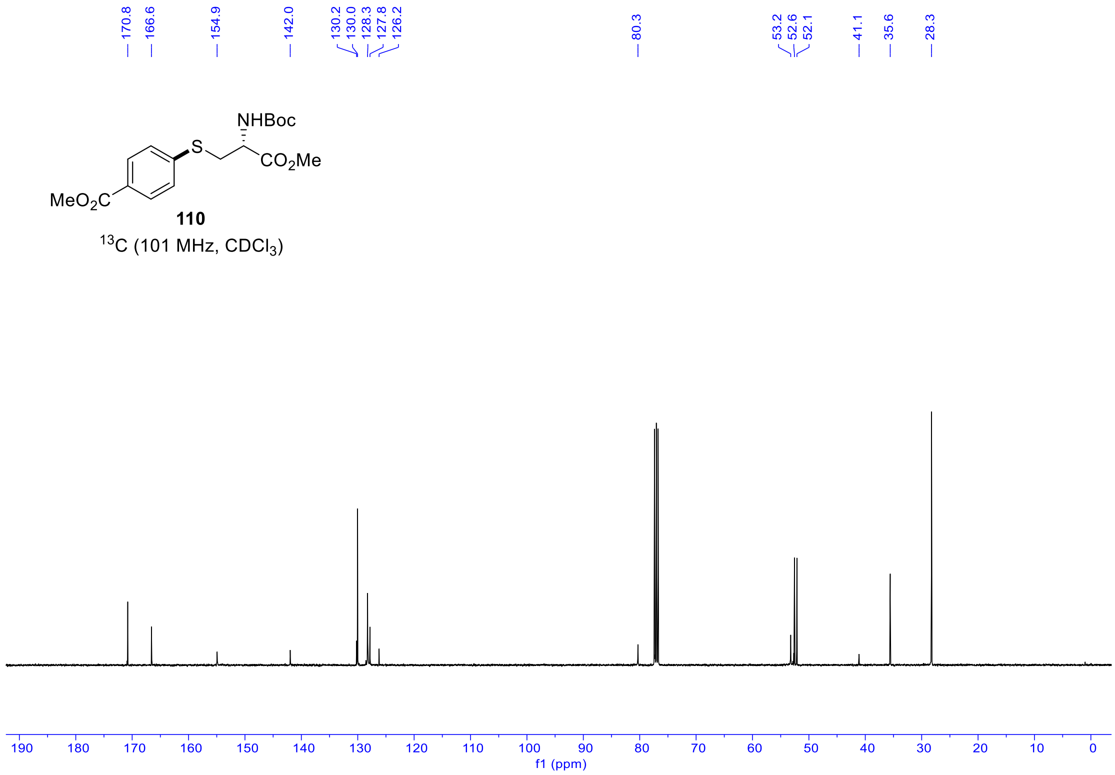

Supplementary Figure 277. ^{13}**C NMR** of compound **110**

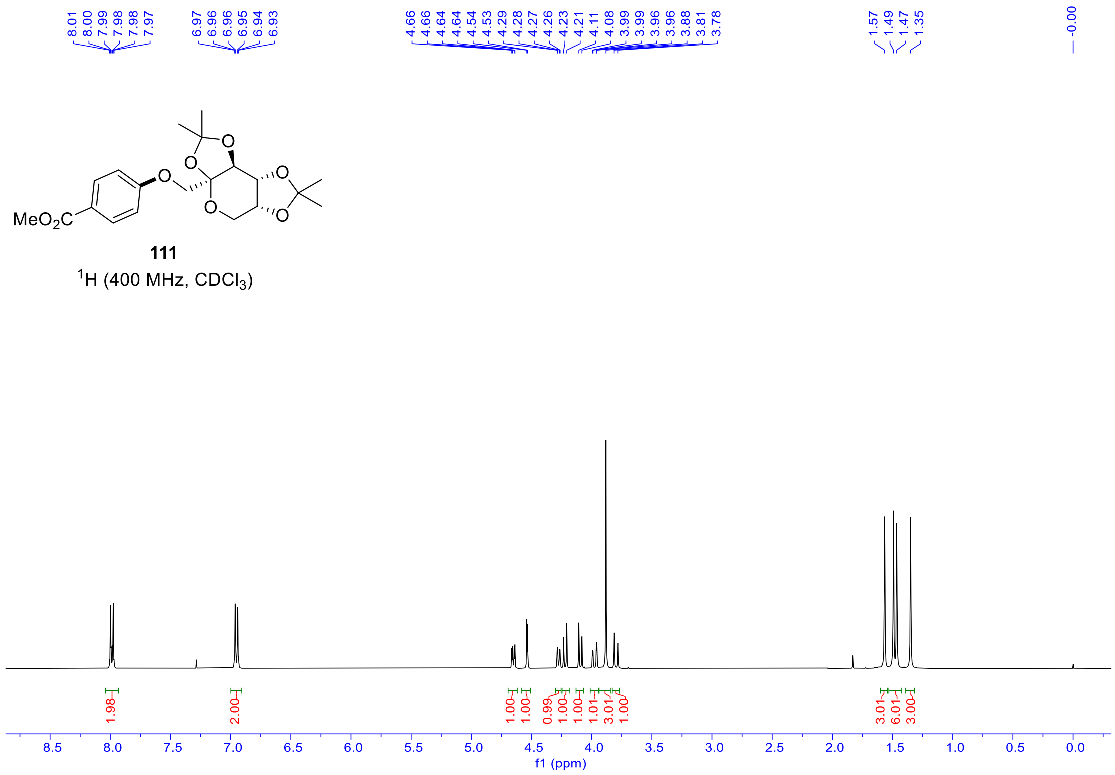

Supplementary Figure 278. ^{1}**H NMR** of compound **111**

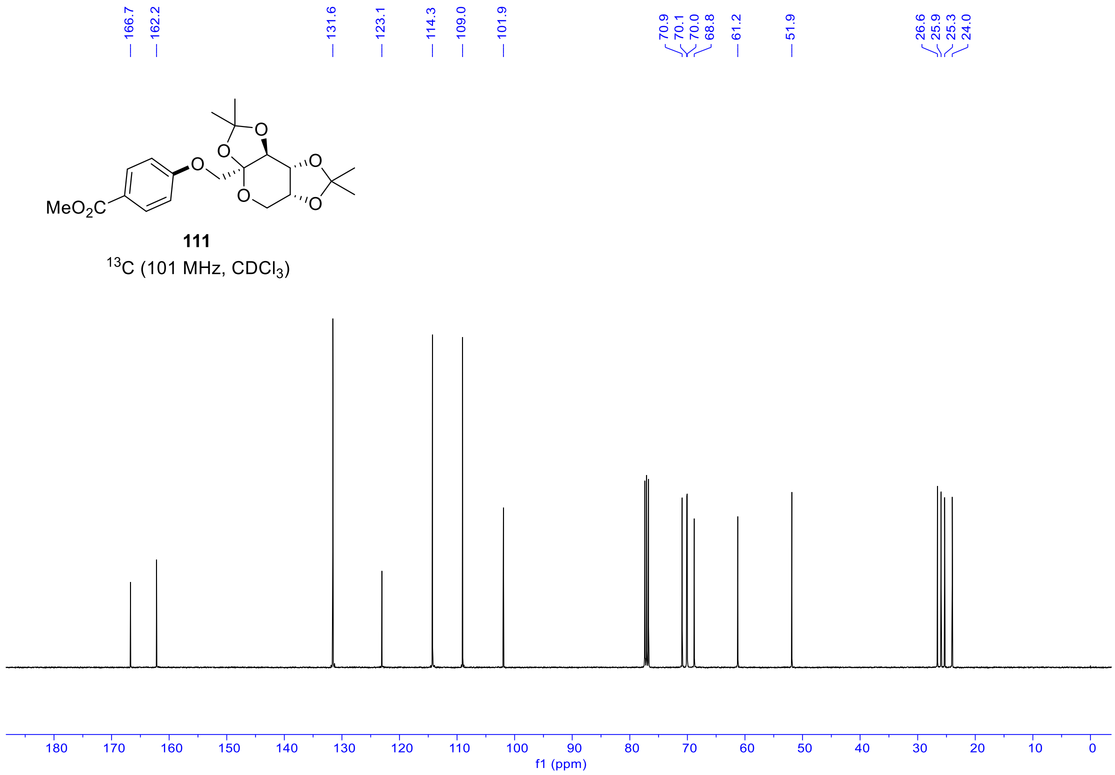

Supplementary Figure 279. ^{13}**C NMR** of compound **111**

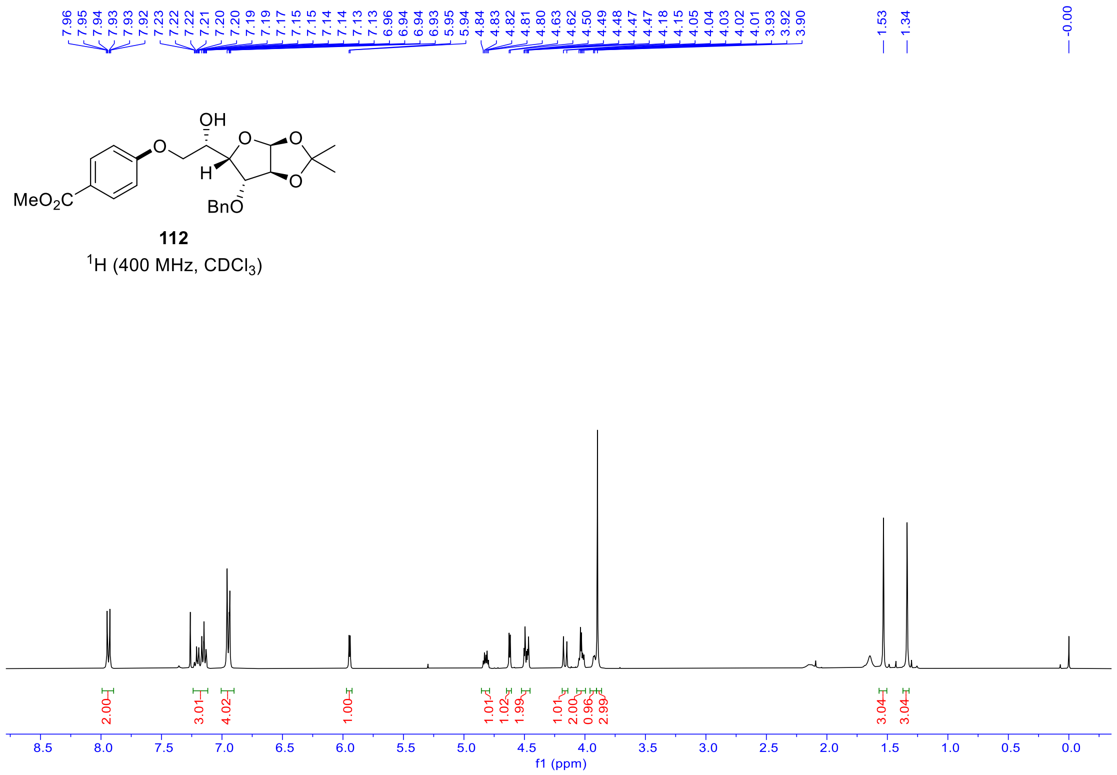

Supplementary Figure 280. ^{1}**H NMR** of compound **112**

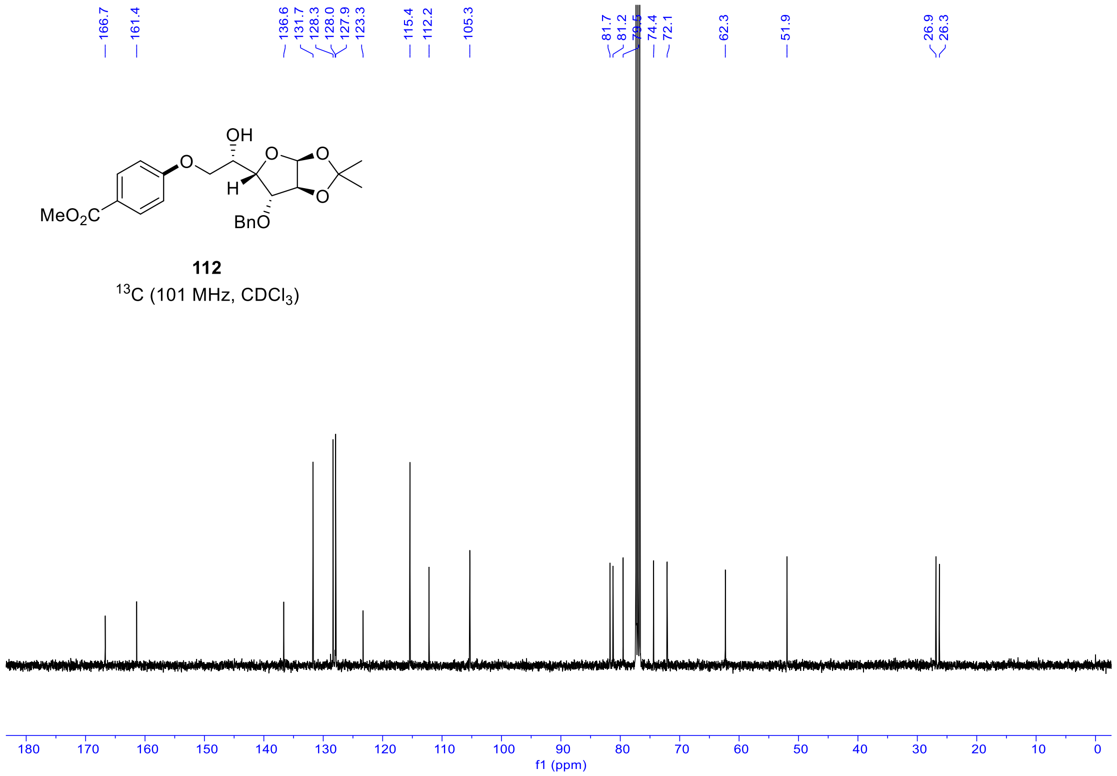

Supplementary Figure 281. ^{13}**C NMR** of compound **112**

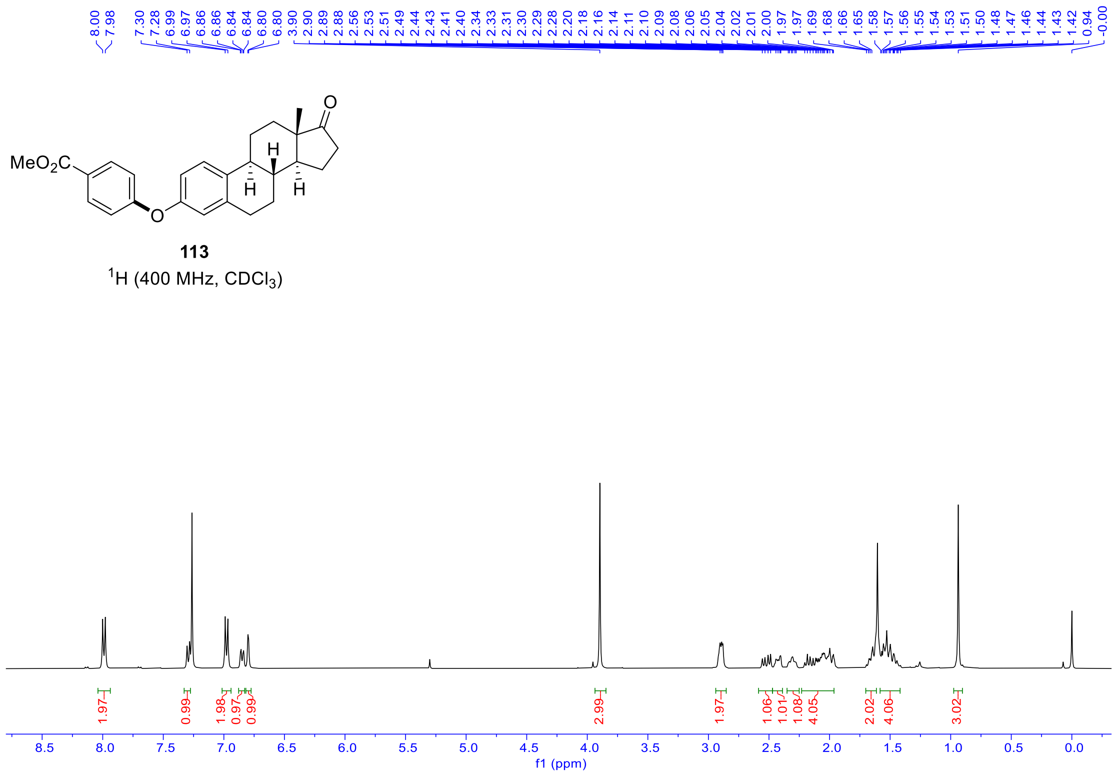

Supplementary Figure 282. ^{1}**H NMR** of compound **113**

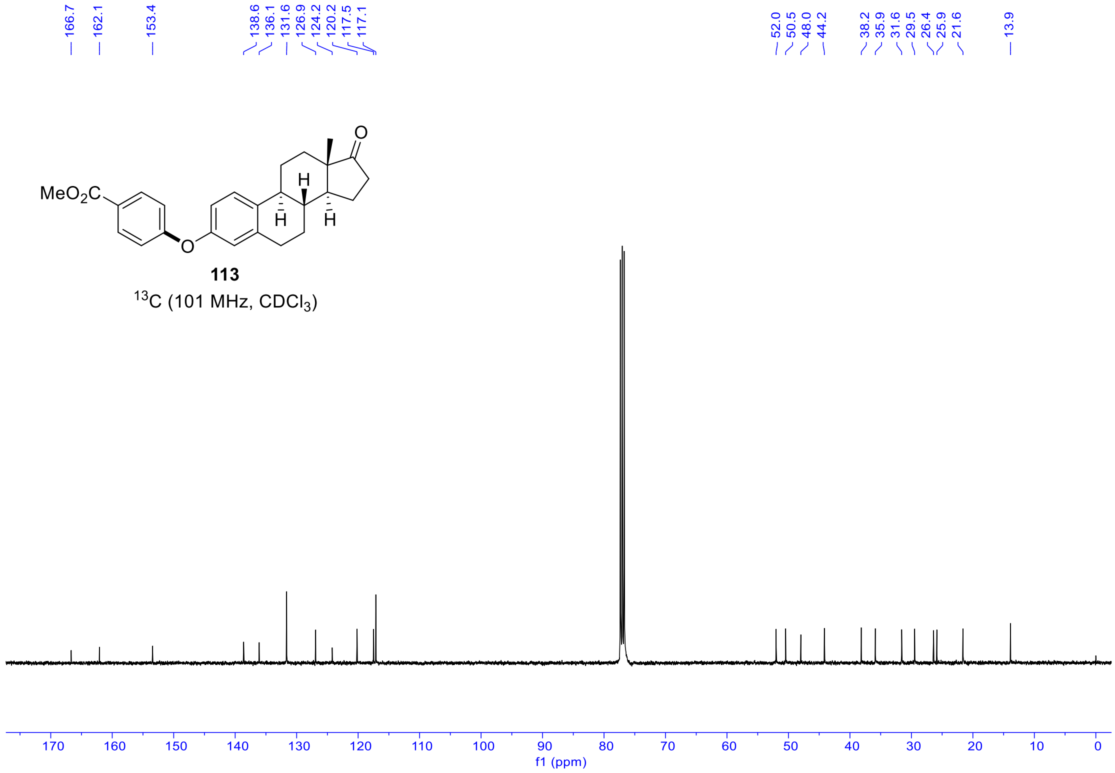

Supplementary Figure 283. ^{13}**C NMR** of compound **113**

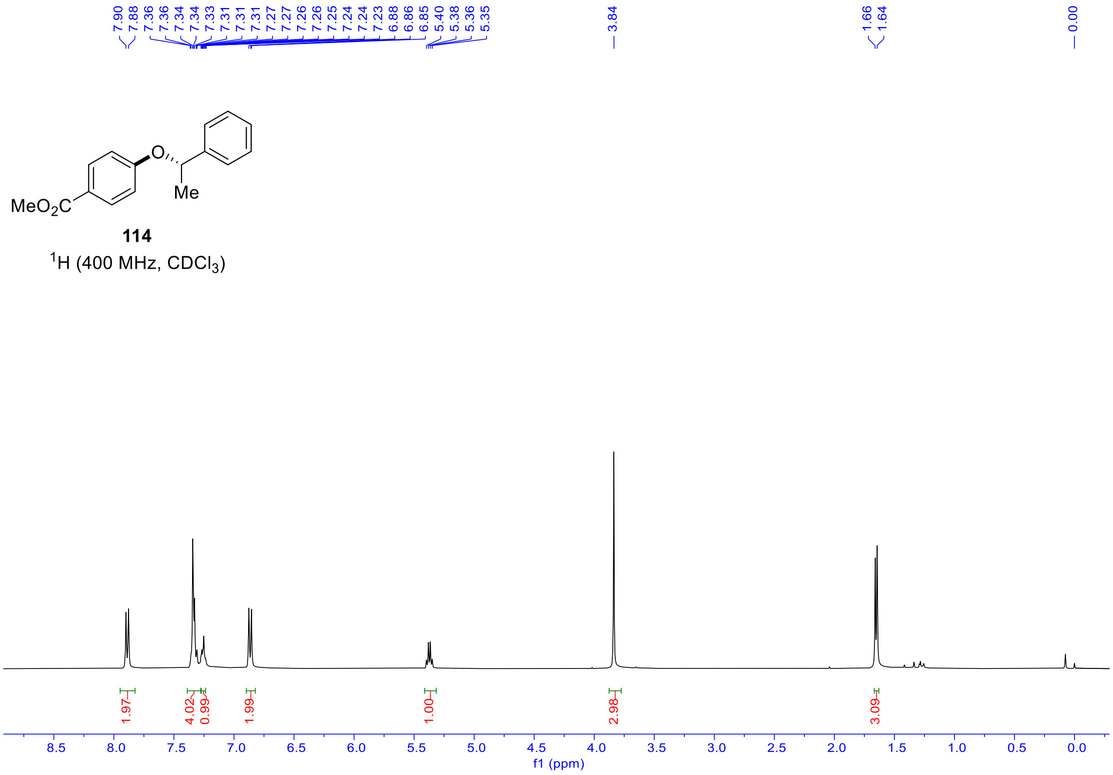

Supplementary Figure 284. ^{1}**H NMR** of compound **114**

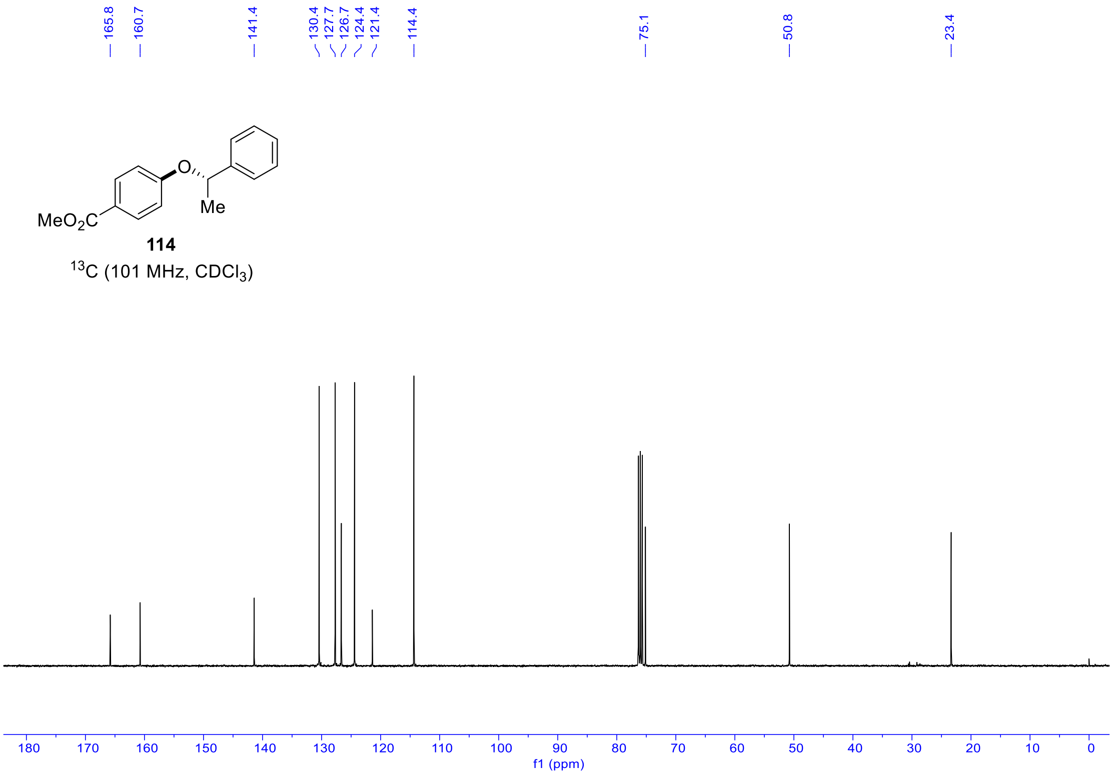

Supplementary Figure 285. ^{13}**C NMR** of compound **114**

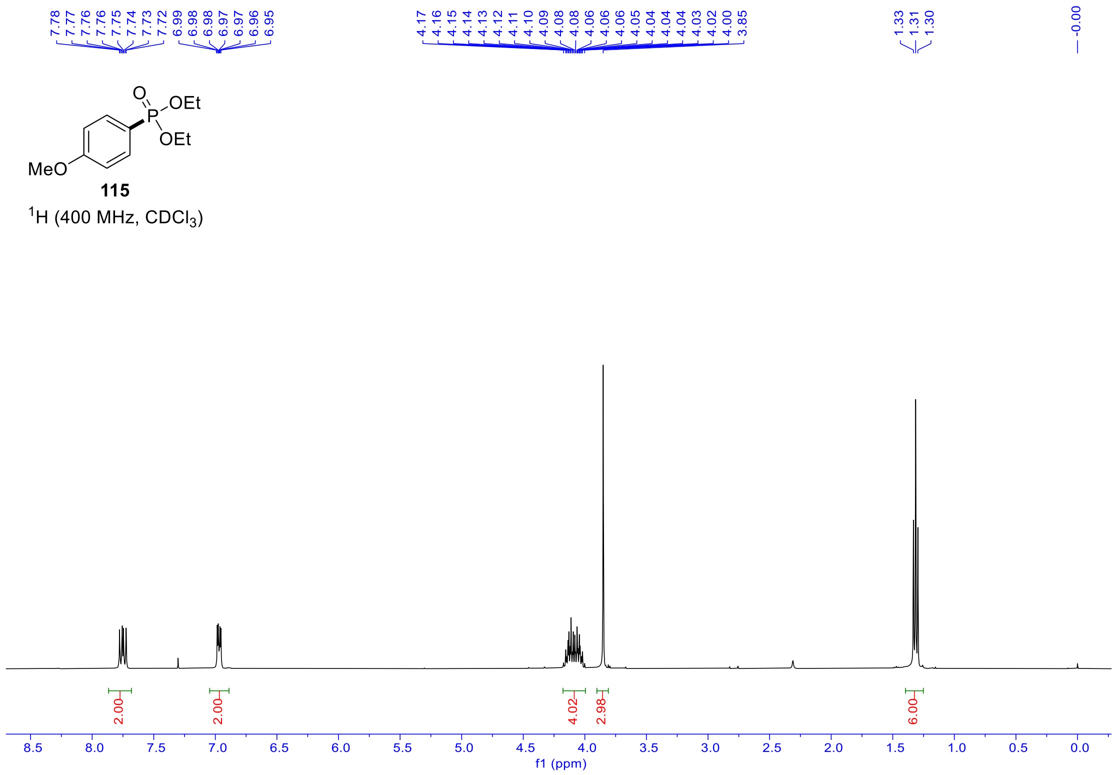

Supplementary Figure 286. ^{1}H NMR of compound **115**

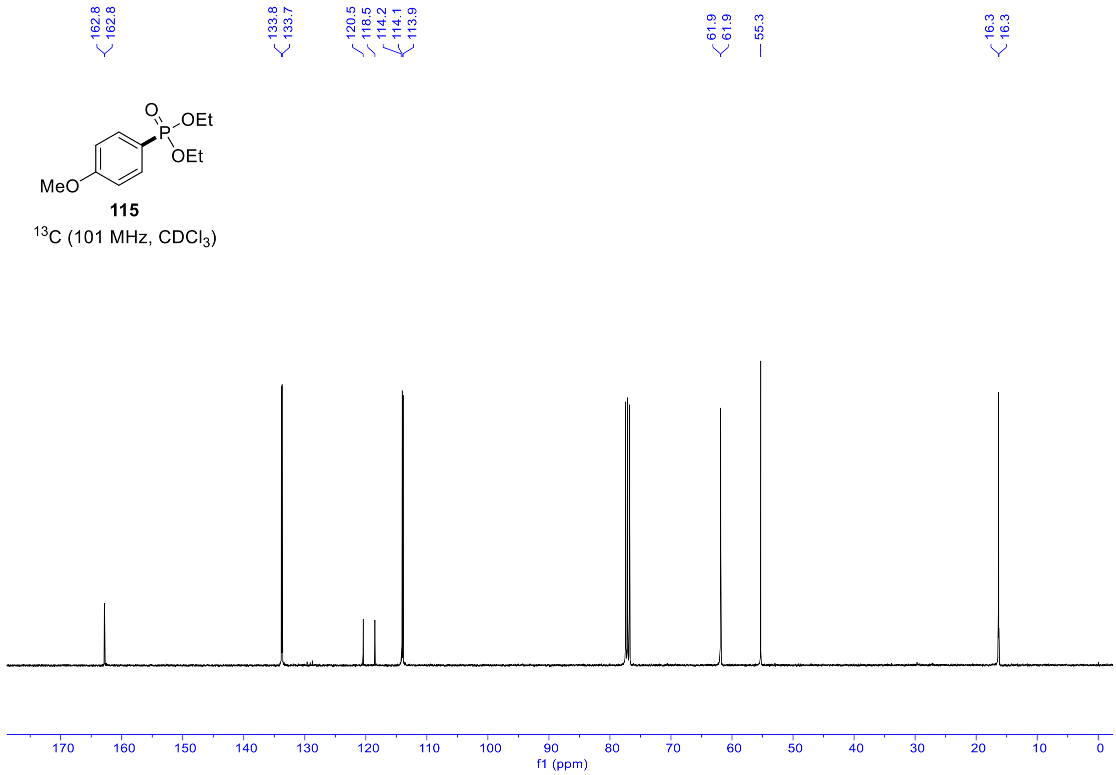

Supplementary Figure 287. ^{13}C NMR of compound **115**

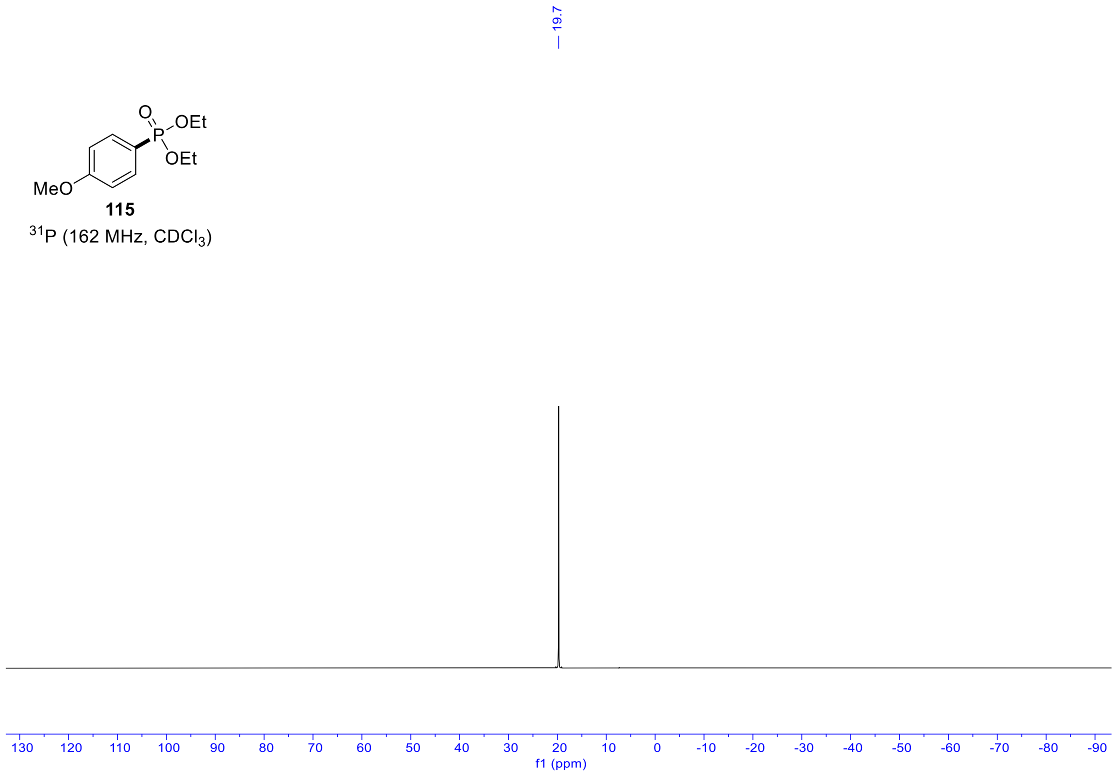

Supplementary Figure 288. ^{31}**P NMR** of compound **115**

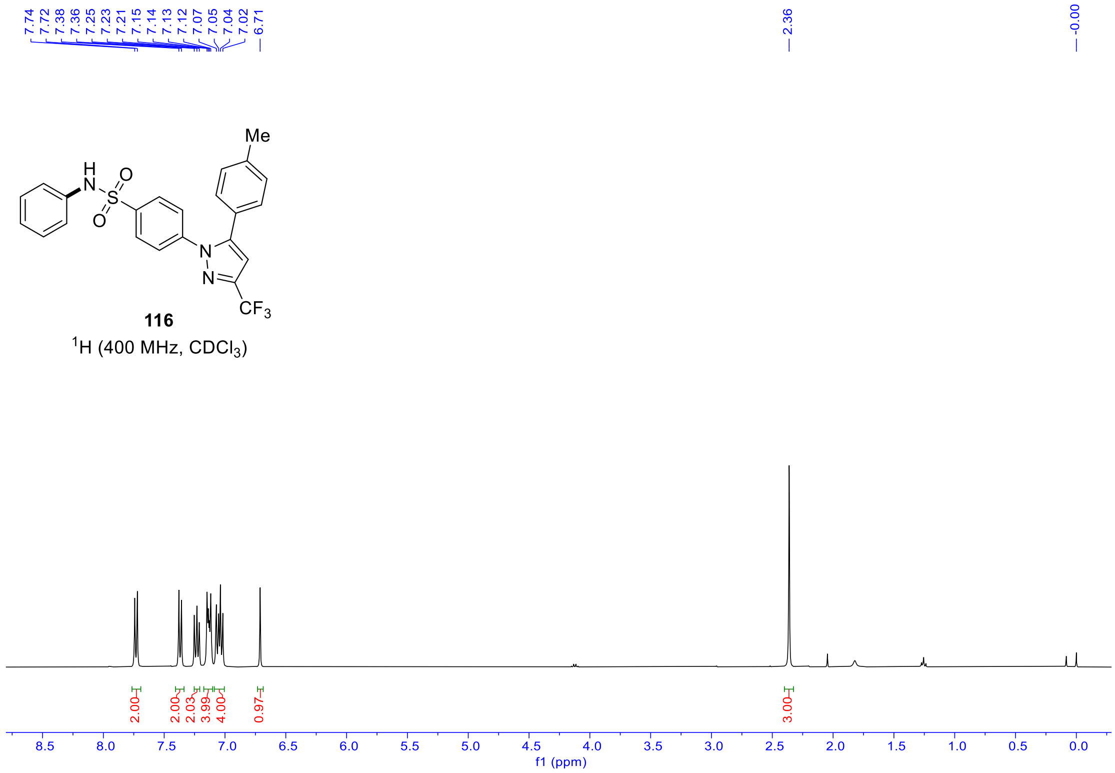

Supplementary Figure 289. ^{1}**H NMR** of compound **116**

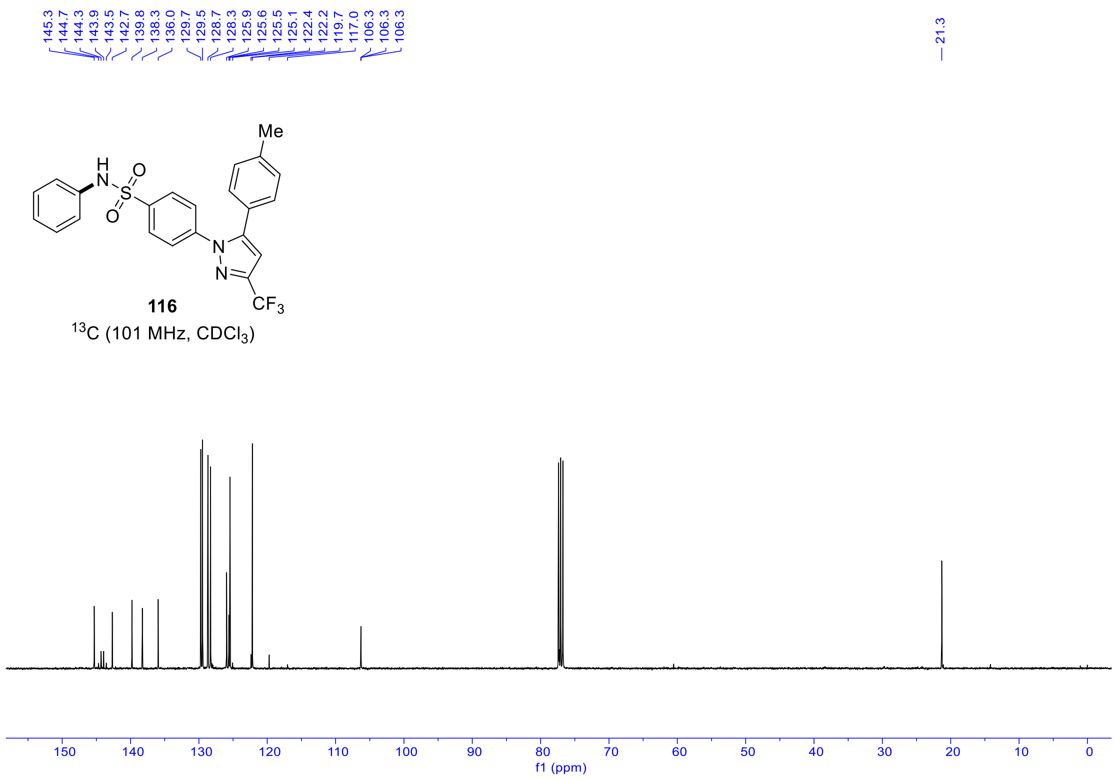

Supplementary Figure 290. ^{13}C NMR of compound **116**

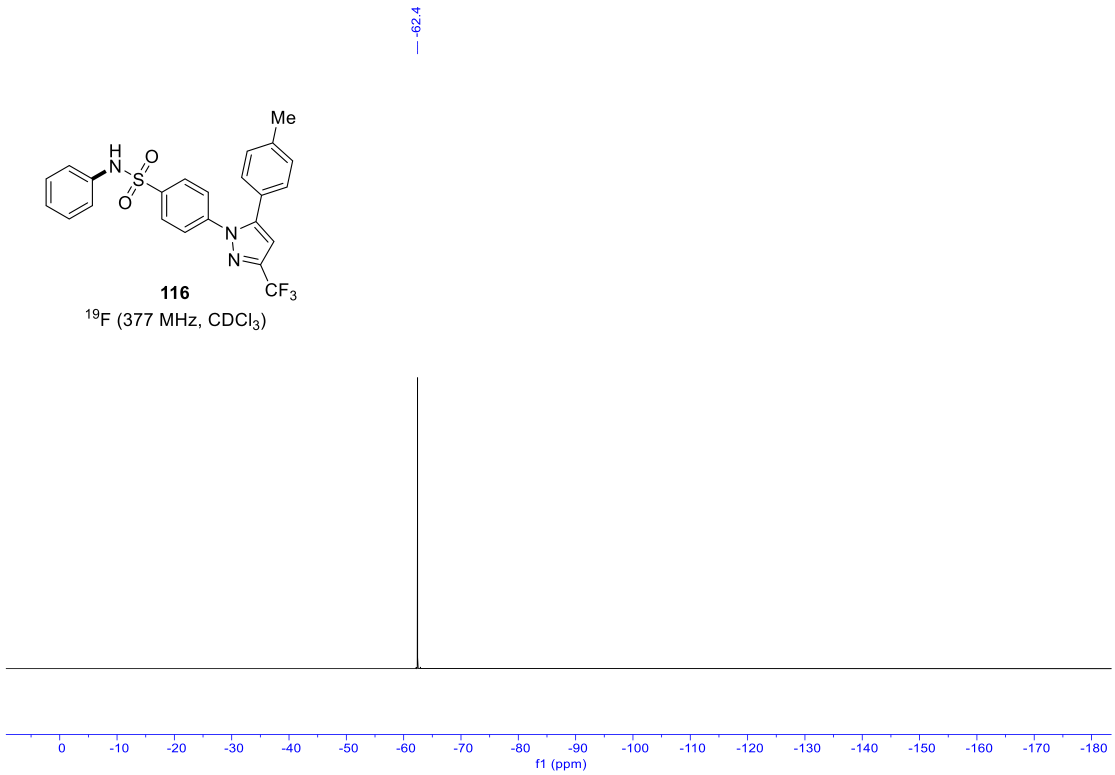

Supplementary Figure 291. ^{19}F NMR of compound **116**

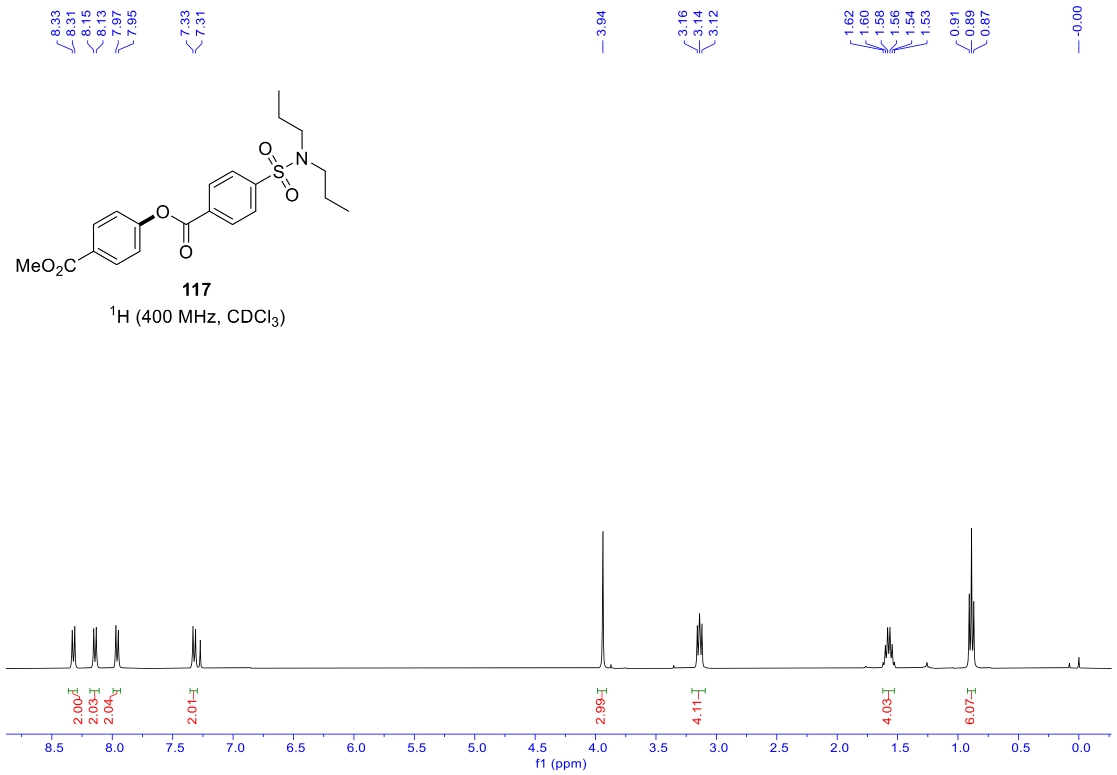

Supplementary Figure 292. ^{1}**H NMR** of compound **117**

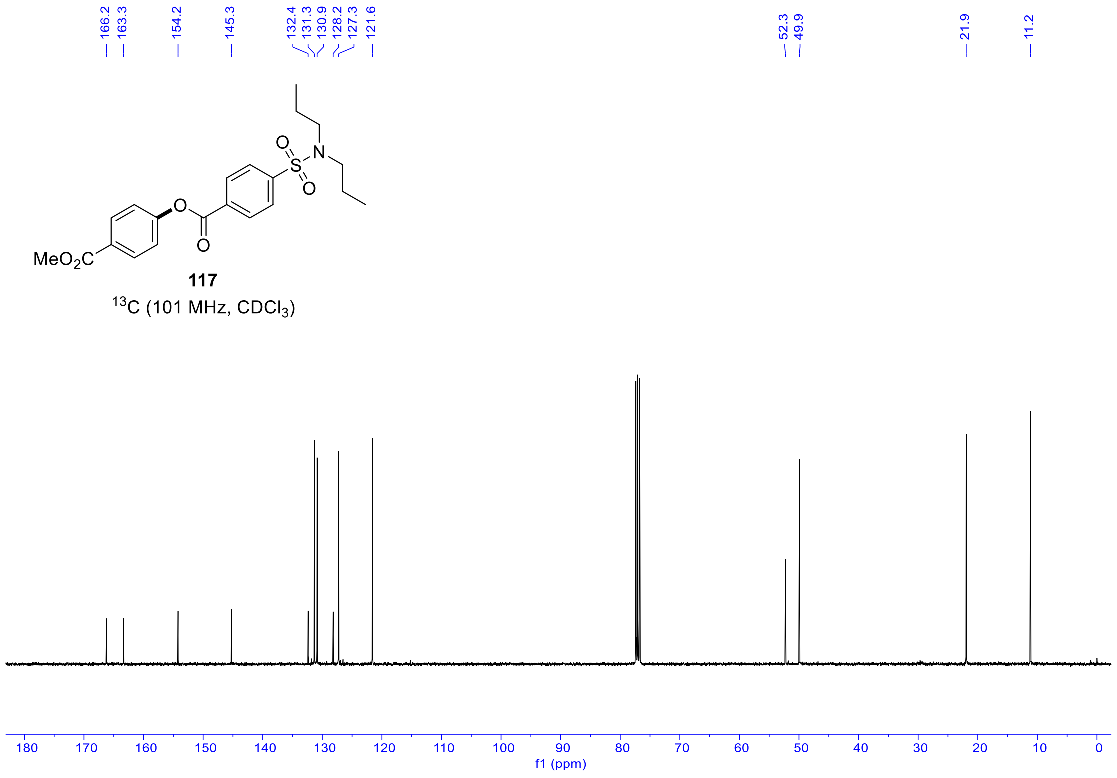

Supplementary Figure 293. ^{13}**C NMR** of compound **117**

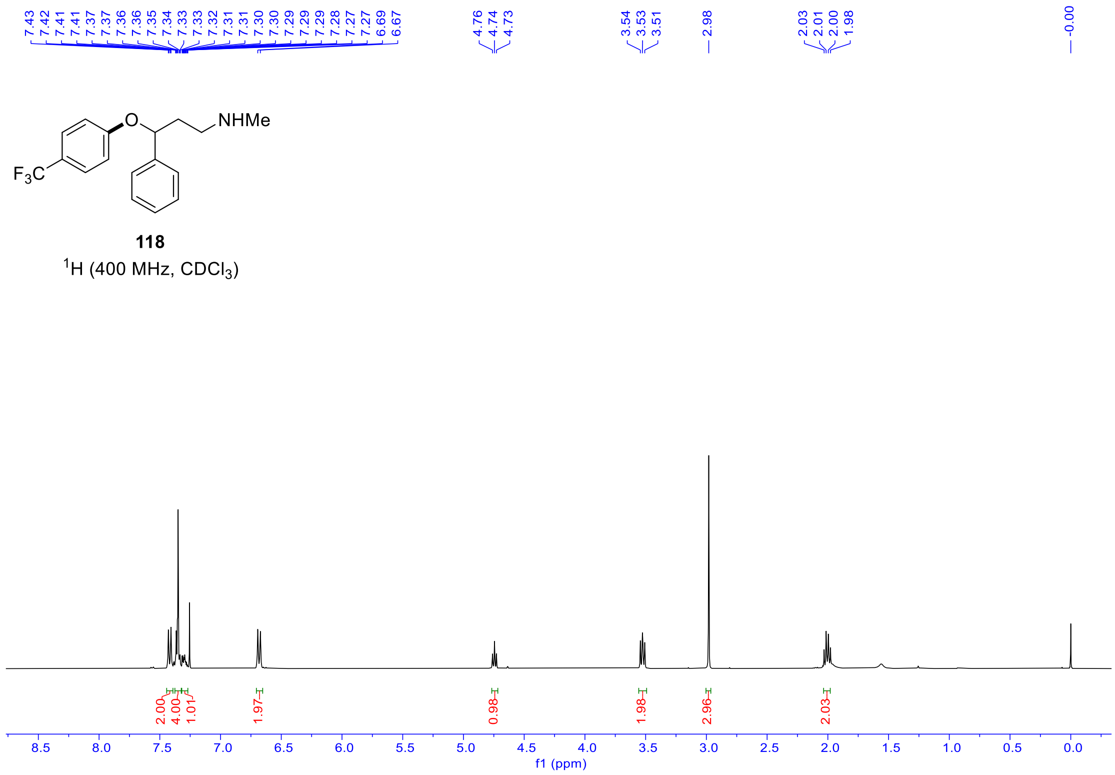

Supplementary Figure 294. **^{1}H NMR** of compound **118**

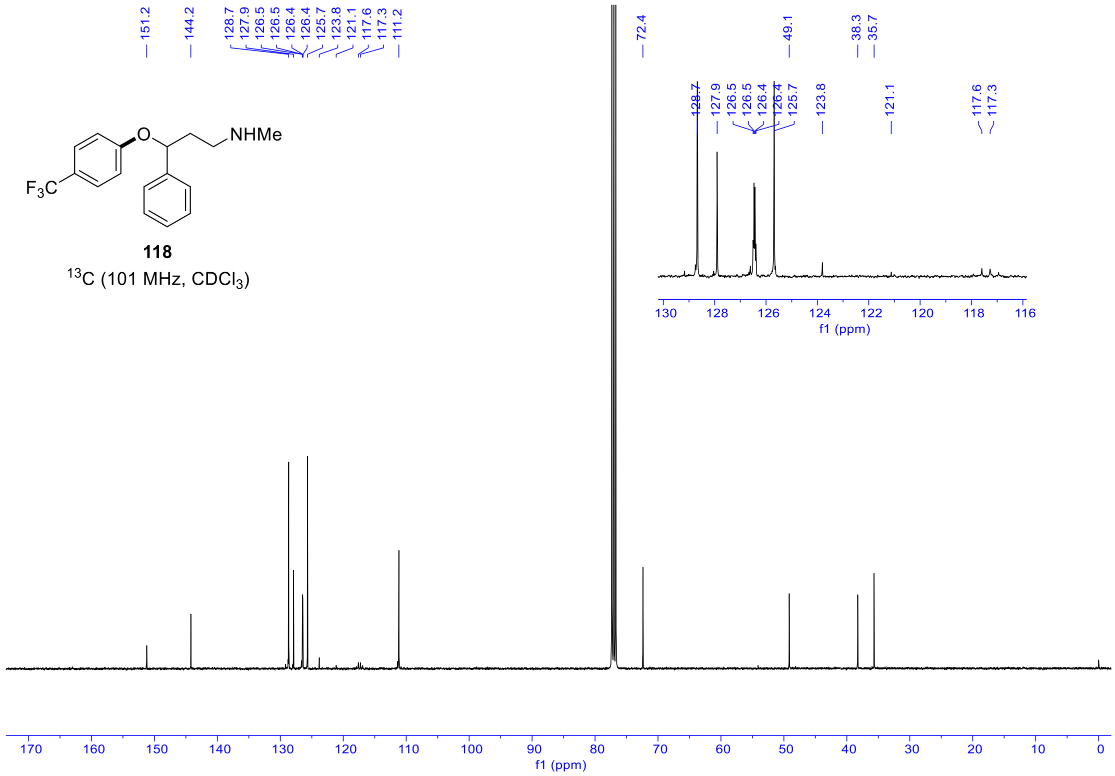

Supplementary Figure 295. **^{13}C NMR** of compound **118**

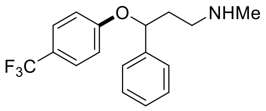

118
^{19}F (377 MHz, CDCl$_3$)

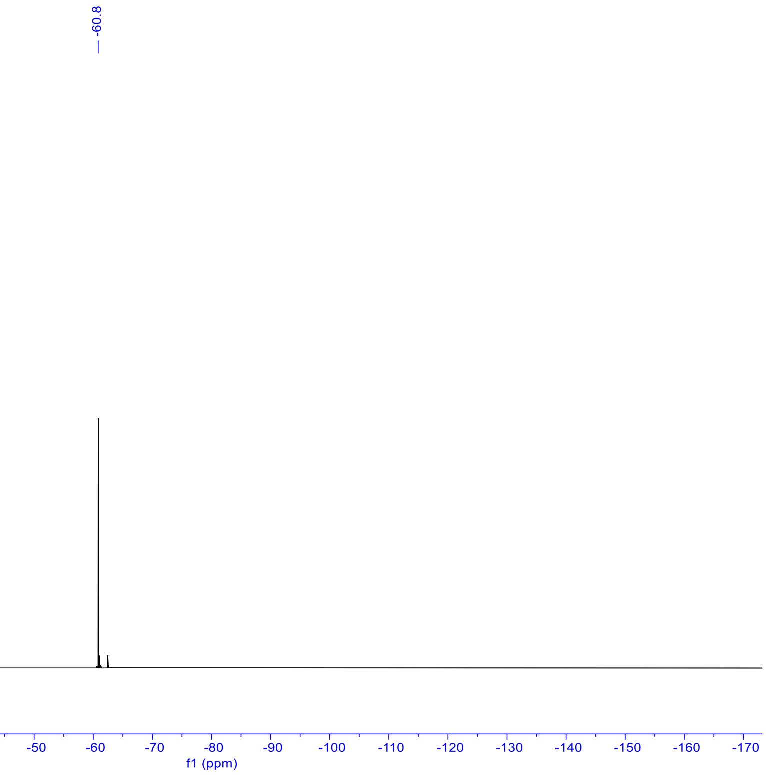

Supplementary Figure 296. ^{19}F NMR of compound **118**

2 Supplementary References

1 a) Yang, Q. *et al*. Remarkable Activity of Potassium-Modified Carbon Nitride for Heterogeneous Photocatalytic Decarboxylative Alkyl/Acyl Radical Addition and Reductive Dimerization of *para*-Quinone Methides. *ACS Sustain. Chem. Eng.* **9**, 2367–2377 (2021). b) He, Y. *et al.* Semi-heterogeneous photocatalytic fluoroalkylation-distal functionalization of unactivated alkenes with $RFSO_2Na$ under air atmosphere. *Green Chem.* **2021**, *23*, 9577–9582.

2 Singh, K., Kabadwal, L. M., Bera, S., Alanthadka, A. & Banerjee, D. Nickel-Catalyzed Synthesis of *N*-Substituted Pyrroles Using Diols with Aryl- and Alkylamines. *J. Org. Chem.* **2018**, *83*, 15406−15414.

3 Wang, X., Blechert, S. & Antonietti, M. Polymeric Graphitic Carbon Nitride for Heterogeneous Photocatalysis. *ACS Catal.* **2012**, *2,* 1596−1606.

4 Goettmann, F., Fischer, A., Antonietti, M. & Thomas, A. Chemical Synthesis of Mesoporous Carbon Nitrides Using Hard Templates and Their Use as a Metal-Free Catalyst for Friedel–Crafts Reaction of Benzene. *Angew. Chem. Int. Ed.* **2006**, *45*, 4467–4471.

5 Li, Y., Zhang, D., Feng, X. & Xiang, Q. Enhanced photocatalytic hydrogen production activity of highly crystalline carbon nitride synthesized by hydrochloric acid treatment. *Chin. J. Catal.* **2020**, *41*, 21–30.

6 Zhao, C. *et al*. Probing supramolecular assembly and charge carrier dynamics toward enhanced photocatalytic hydrogen evolution in 2D graphitic carbon nitride nanosheets. *Appl. Catal. B: Environ.* **2019**, *256*, 117867.

7 Zheng, J. & Zhang, L. Incorporation of CoO nanoparticles in 3D marigold flower-like hierarchical architecture $MnCo_2O_4$ for highly boosting solar light photo-oxidation and reduction ability. *Appl. Catal. B: Environ.* **2018**, *237*, 1–8.

8 Sun, S., Wang, W., Li, D., Zhang, L. & Jiang, D. Solar Light Driven Pure Water Splitting on Quantum Sized $BiVO_4$ without any cocatalyst, *ACS Catal.* **2014**, *4* 3498–3503.

9 Gaussian 16, Revision B.01, M. J. Frisch, G. W. Trucks, H. B. Schlegel, G. E. Scuseria, M. A. Robb, J. R. Cheeseman, G. Scalmani, V. Barone, G. A. Petersson, H. Nakatsuji, X. Li, M. Caricato, A. V. Marenich, J. Bloino, B. G. Janesko, R. Gomperts, B. Mennucci, H. P. Hratchian, J. V. Ortiz, A. F. Izmaylov, J. L. Sonnenberg, D. Williams-Young, F. Ding, F. Lipparini, F. Egidi, J. Goings, B. Peng, A. Petrone, T. Henderson, D. Ranasinghe, V. G. Zakrzewski, J. Gao, N. Rega, G. Zheng, W. Liang, M. Hada, M. Ehara, K. Toyota, R. Fukuda, J. Hasegawa, M. Ishida, T. Nakajima, Y. Honda, O. Kitao, H. Nakai, T. Vreven, K. Throssell, J. A. Montgomery, Jr., J. E. Peralta, F. Ogliaro, M. J. Bearpark, J. J. Heyd, E. N. Brothers, K. N. Kudin, V. N. Staroverov, T. A. Keith, R. Kobayashi, J. Normand, K. Raghavachari, A. P. Rendell, J. C. Burant, S. S. Iyengar, J. Tomasi, M. Cossi, J. M. Millam, M. Klene, C. Adamo, R. Cammi, J. W. Ochterski, R. L. Martin, K. Morokuma, O. Farkas, J. B. Foresman, and D. J. Fox, Gaussian, Inc., Wallingford CT, 2016

10 a). J. P. Perdew, K. Burke, M. Ernzerhof, Generalized Gradient Approximation Made Simple. *Phys. Rev. Lett.* **1996**, *77*, 3865. b). J. P. Perdew, K. Burke, M.

Ernzerhof, Generalized Gradient Approximation Made Simple. *Phys. Rev. Lett.* **1997**, *78*, 1396.

11 Weigend, F. & Ahlrichs, R. Balanced basis sets of split valence, triple zeta valence and quadruple zeta valence quality for H to Rn: Design and assessment of accuracy. *Phys. Chem. Chem. Phys.*, **2005**, *7*, 3297–3305.

12 Zhao, X. *et al*. Nickel-Coordinated Carbon Nitride as a Metallaphotoredox Platform for the Cross-Coupling of Aryl Halides with Alcohols. *ACS Catal.* **2020**, *10*, 15178−15185.

13 Vijeta, A. Casadevall, C. Roy, S. & Reisner, E. Visible-Light Promoted C–O Bond Formation with an Integrated Carbon Nitride–Nickel Heterogeneous Photocatalyst. *Angew. Chem. Int. Ed.* **2021**, *60*, 8494−8499.

14 Zhu, D. *et al*. Visible-Light-Induced Nickel-Catalyzed P(O)–C(sp^2) Coupling Using Thioxanthen-9-one as a Photoredox Catalysis. *Org. Lett*. **2021**, *23*, 160–165.

15 Xie, Y., Liu, S., Liu, Y., Wen, Y. & Deng, G. Palladium-Catalyzed One-Pot Diarylamine Formation from Nitroarenes and Cyclohexanones. *Org. Lett*. **2012**, *14*, 1692–1695.

16 Cavedon, C., Madani, A., Seeberger, P. H. & Pieber, B. Semiheterogeneous Dual Nickel/Photocatalytic (Thio)Etherification Using Carbon Nitrides. *Org. Lett*. **2019**, *21*, 5331–5334.

17 Pieber, B. *et al*. Semi-Heterogeneous Dual Nickel/Photocatalysis using Carbon Nitrides: Esterification of Carboxylic Acids with Aryl Halides. *Angew. Chem. Int. Ed.* **2019**, *58*, 9575–9580.

18 Xuan, J., Zeng, T., Chen, J., Lu, L. & Xiao, W. Room Temperature C-P Bond Formation Enabled by Merging Nickel Catalysis and Visible-Light-Induced Photoredox Catalysis. *Chem. - Eur. J.* **2015**, *21*, 4962–4965.

19 Zhang, X. *et al*. Ni(II)/Zn Catalyzed Reductive Coupling of Aryl Halides with Diphenylphosphine Oxide in Water. *Org. Lett*. **2011**, *13*, 3478–3481.

20 McErlain, H., Riley, L. M. & Sutherland, A. Palladium-Catalyzed C-P Bond-Forming Reactions of Aryl Nonaflates Accelerated by Iodide. *J. Org. Chem.* **2021**, *86*, 17036–17049.

21 Koohgard, M., Karimitabar, H. & Hosseini-Sarvari, M. Visible-Light-Mediated Semi-Heterogeneous Black TiO_2/Nickel Dual Catalytic C(sp^2)-P Bond Formation toward Aryl Phosphonates. Dalton Trans., **2020**, *49*, 17147–17151.

22 Zhuang, R. *et al*. Copper-Catalyzed C−P Bond Construction via Direct Coupling of Phenylboronic Acids with H-Phosphonate Diesters. *Org. Lett.* **2011**, *13*, 2110–2113.

23 Roscales, S. & CsákÿA. G. Synthesis of Di(hetero)arylamines from Nitrosoarenes and Boronic Acids: A General, Mild, and Transition-Metal-Free Coupling. *Org. Lett*. **2018**, *20*, 1667–1671.

24 Akram, M. O., Das, A., Chakrabarty, I. & Patil, N. T. Ligand-Enabled Gold-Catalyzed C (sp^2)-N Cross-Coupling Reactions of Aryl Iodides with Amines. *Org. Lett*. **2019**, *21*, 8101–8105.

25 Yang, F. *et al*. Reductive C-N Coupling of Nitroarenes: Heterogenization of MoO_3 Catalyst by Confinement in Silica. *ChemSusChem*, **2021**, *14*, 3413–3421.
26 Viciu, M. S. *et al*. Synthesis, Characterization, and Catalytic Activity of N-Heterocyclic Carbene (NHC) Palladacycle Complexes. *Org. Lett*. **2003**, *5*, 1479–1482.
27 Chen, K. *et al*. Sequential C–S and S–N Coupling Approach to Sulfonamides. *Org. Lett*. **2020**, *22*, 1841–1845.
28 Moon, S.-Y., Nam, J., Rathwell, K. & Kim, W.-S. Copper-Catalyzed Chan-Lam Coupling between Sulfonyl Azides and Boronic Acids at Room Temperature. *Org. Lett*. **2014**, *16*, 338–341.
29 Ichitsuka, T., Takahashi, I., Koumura, N., Sato, K. & Kobayashi, S. Continuous Synthesis of Aryl Amines from Phenols Utilizing Integrated Packed-Bed Flow Systems. *Angew. Chem., Int. Ed*. **2020**, *59*, 15891–15896.
30 Michel, B. & Greaney, M. F. Continuous-Flow Synthesis of Trimethylsilylphenyl Perfluorosulfonate Benzyne Precursors. *Org. Lett*. **2014**, *16*, 2684–2687.
31 Mackey, K., Jones, D. J., Pardo, L. M. & McGlacken, G. P. Quinoline Ligands Improve the Classic Direct C-H Functionalisation/Intramolecular Cyclisation of Diaryl Ethers to Dibenzofurans. *Eur. J. Org. Chem*. **2021**, *3*, 495–498.
32 Zheng, Z. *et al*. $PhI(OAc)_2$-Mediated Intramolecular Oxidative Aryl-Aldehyde C*sp*^{2}-C*sp*^{2} Bond Formation: Metal-Free Synthesis of Acridone Derivatives. *J. Org. Chem*. **2014**, *79*, 7451–7458.
33 Gisbertz, S., Reischauer, S. & Pieber, B. Overcoming Limitations in Dual Photoredox/Nickel-Catalysed C-N Cross-Couplings Due to Catalyst Deactivation. *Nat. Catal*. **2020**, *3*, 611–620.
34 Hoshimoto, Y., Kinoshita, T., Hazra, S., Ohashi, M. & Ogoshi, S. Main-Group-Catalyzed Reductive Alkylation of Multiply Substituted Amines with Aldehydes Using H_2. *J. Am. Chem. Soc*. **2018**, *140*, 7292–7300.
35 Urgaonkar, S., Nagarajan, M. & Verkade, J. G. $P[N(i\text{-}Bu)CH_2CH_2]_3N$: A Versatile Ligand for the Pd-Catalyzed Amination of Aryl Chlorides. *Org. Lett*. **2003**, *5*, 815–818.
36 Baranwal, S. & Kandasamy, J. Copper Catalyzed *N*-Arylation of Sulfoximines with Aryldiazonium Salts in the Presence of DABCO under Mild Conditions. *Tetrahedron Lett*. **2020**, *61*, 152079.
37 Shirakawa, E., Nishikawa, R., Uchiyama, N., Hata, I. & Hayashi, T. Copper-catalyzed Oxidative C–C, C–O, and C–N Bond Forming Reactions of Arylboronic Acids. *Chem. Lett*. **2013**, *42*, 269–271.
38 Zhang, R. *et al*. A Practical and Sustainable Protocol for Direct Amidation of Unactivated Esters under Transition-Metal-Free and Solvent-Free Conditions. *Green Chem*. **2021**, *23*, 3972–3982.
39 Reddy, L. R. *et al*. *N*-Arylation of Carbamates through Photosensitized Nickel Catalysis. *J. Org. Chem*. **2018**, *83*, 13854–13860.
40 Cao, D. *et al*. Light-Driven Metal-Free Direct Deoxygenation of Alcohols under Mild Conditions. *iScience* **2020**, *23*, No. 101419.

41 Liu, Q. *et al*. Efficient Synthesis of Unsymmetrical Heteroaryl Ethers by a Transition Metal-Free C-O Crosscoupling Reaction of Activated and Unactivated Heteroaryl Chlorides with Alcohols and Phenols. *Chin. J. Chem*. **2013**, *31*, 764–772.

42 Kasai, S. *et al*. Synthesis, Structure-Activity Relationship, and Pharmacological Studies of Novel Melanin-Concentrating Hormone Receptor 1 Antagonists 3-Aminomethylquinolines: Reducing Human Ether-A-Go-Go-Related Gene (hERG)-Associated Liabilities. *J. Med. Chem.* **2012**, *55*, 4336–4351.

43 Davies, J. *et al*. Ni-Catalyzed Carboxylation of Aziridines en Route to β-Amino Acids. *J. Am. Chem. Soc.* **2021**, *143*, 4949–4954.

44 Siera, H., Semleit, N., Kreuzahler, M., Wölper, C. & Haberhauer, G. Gold Catalysis of Non-Conjugated Haloacetylenes. *Synthesis*. **2021**, *53*, 1457–1470.

45 Johann, T., Keth, J., Bros, M. & Frey, H. A General Concept for the Introduction of Hydroxamic Acids into Polymers. *Chem. Sci.* **2019**, *10*, 7009–7022.

46 Welin, E. R., Le, C., Arias-Rotondo, D. M., McCusker, J. K. & MacMillan, D. W. C. Photosensitized, Energy Transfer-Mediated Organometallic Catalysis through Electronically Excited Nickel(II). *Science* **2017**, *355*, 380–385.

47 Becht, J. M., Wagner, A. & Mioskowski, C. Facile Introduction of SH Group on Aromatic Substrates via Electrophilic Substitution Reactions. *J. Org. Chem*. **2003**, *68*, 5758–5761.

48 Ahn, K. C. *et al*. An Immunoassay to Evaluate Human/Environmental Exposure to the Antimicrobial Triclocarban. *Environ. Sci. Technol.* **2012**, *46*, 374–381.

49 Cook, X. A. F. *et al*. Base-Activated Latent Heteroaromatic Sulfinates as Nucleophilic Coupling Partners in Palladium-Catalyzed Cross-Coupling Reactions. *Angew. Chem., Int. Ed*. **2021**, *60*, 22461–22468.

50 Liu, B., Lim, C, H. & Miyake, G. M. Visible-Light-Promoted C-S Cross-Coupling via Intermolecular Charge Transfer. *J. Am. Chem. Soc*. **2017**, *139*, 13616–13619.

51 Ishitobi, K., Muto, K. & Yamaguchi, J. Pd-Catalyzed Alkenyl Thioether Synthesis from Thioesters and *N*-Tosylhydrazones. *ACS Catal*. **2019**, *9*, 11685–11690.

52 Guo, W. *et al*. Metal-Free, Room-Temperature, Radical Alkoxycarbonylation of Aryldiazonium Salts through Visible-Light Photoredox Catalysis. *Angew. Chem., Int. Ed*. **2015**, *54*, 2265–2269.

53 Upadhyay, R., Singh, D. & Maurya, S. K. Highly Efficient Heterogeneous V_2O_5@TiO_2 Catalyzed the Rapid Transformation of Boronic Acids to Phenols. *Eur. J. Org. Chem.* **2021**, *28*, 3925–3931.

54 Tao, R. et al. $Fe(OTf)_3$-Catalyzed Tandem Meyer-Schuster Rearrangement/Intermolecular Hydroamination of 3-Aryl Propargyl Alcohols for the Synthesis of Acyclic β-aminoketones. *Tetrahedron*, **2017**, *73*, 1762–1768.

55 Gallagher, R. T., Basu, S. & Stuart, D. R.; Trimethoxyphenyl (TMP) as a Useful Auxiliary for in situ Formation and Reaction of Aryl(TMP)iodonium Salts: Synthesis of Diaryl Ethers. *Adv. Synth. Catal*. **2020**, *362,* 320–325.

56 Lakemeyer, M. *et al*. Tailored Peptide Phenyl Esters Block Clpxp Proteolysis by an Unusual Breakdown into a Heptamer-Hexamer Assembly. *Angew. Chem. Int. Ed.* **2019**, *58*, 7127–7132.
